# Influence of interaction of cerebral fluids on ventricular deformation: A mathematical approach

**DOI:** 10.1371/journal.pone.0264395

**Published:** 2022-02-28

**Authors:** Galina Valova, Olga Bogomyakova, Andrey Tulupov, Alexander Cherevko

**Affiliations:** 1 Lavrentyev Institute of Hydrodynamics of the Siberian Branch of the Russian Academy of Sciences, Novosibirsk, Russia; 2 International Tomography Center of Siberian Branch of the Russian Academy of Sciences, Novosibirsk, Russia; Tongji University, CHINA

## Abstract

This paper describes the effects of the interaction of cerebral fluids (arterial, capillary and venous blood, cerebrospinal fluid) on ventricular wall displacement and periventricular pressure using a mathematical multiphase poroelasticity model for the cerebral parenchyma. The interaction of cerebral fluids is given by a set of four numerical coefficients. A multiple linear regression with interaction is constructed that allows us to quantify the effect of these coefficients on the average ventricular wall displacement. The prevailing influence of an arterial-liquor component was observed. The sets of coefficients associated with such pathological conditions were found: normal pressure hydrocephalus, intracranial hypertension, and replacement ventriculomegaly under a prolonged hypoperfusion.

## Introduction

There are a large number of pathological conditions of the central nervous system (CNS) characterized by the impaired movement of intracerebral fluids. Infectious diseases, craniocerebral injuries, cerebral circulatory disorders, and neurodegenerative diseases can lead to an imbalance in cerebral hemo- and cerebrospinal fluid dynamics and structural changes in brain tissue. One such condition is hydrocephalus, which is characterized by enlargement of the cerebral ventricles [1]. Its diagnostics is based on clinical findings and neuroimaging results (CT, MRI). An obstructive form is usually distinguished when hydrocephalus develops due to impaired cerebrospinal fluid (CSF) movement and is itself an active process [2]. The non-obstructive form of hydrocephalus is characterized by the absence of obstruction for CSF movement and is most often associated with excessive CSF formation (in a tumor lesion of the vascular plexuses), impaired resorption (absorption) of CSF (e.g., after infectious disease or TBI), or is idiopathic. This category includes normal pressure hydrocephalus [3], characterized by ventricular dilatation without development of a hypertensive syndrome and accompanied by the Hakim-Adams triad and all other variants of communicating hydrocephalus without a known cause. In this group of diseases, the mechanism of hydrocephalus development is not fully understood; moreover, there is still no consensus on reliable diagnostic criteria to suppose its progression. It should be noted that ventricular enlargement may be associated with other pathological processes and do not always cause the existing neurological deficit.

Imaging techniques are now intensively developing and allow us to study more complicated multi-component structures [4, 5]. Using imaging techniques, we can detect ventricular changes and formed structural changes in the brain parenchyma. Using clinical examination methods, we can determine neurological abnormalities. But we cannot always reliably determine the degree of abnormalities in intracranial CSF dynamics, the prognosis for a disease, and the mechanisms of ventricular deformation. The mathematical modeling of pathological processes helps to complete the clinical picture.

The use of lumped-parameter or compartment mathematical models, where the cranial contents consist of interconnected compartments that exchange fluid, allows us to study the effects of model parameters on fluid flow in the brain. Such models provide information about intracranial pressure and the interaction of CSF and blood flow with the brain parenchyma. The mathematical model of CSF pressure-volume compensation provides a theoretical basis for the differential diagnostics of hydrocephalus [6]. In [7], a mathematical model of ventricular volume regulation based on fluid mechanical principles was established. It was shown that in normal pressure hydrocephalus, when the CSF flow between the cerebrospinal and cortical subarachnoid spaces is restricted and the brain becomes more compressible, the volume of the ventricles increases with a minimal increase in intracranial pressure. A more complex multicompartmental model was proposed in [8]. This compartmental model predicts intracranial pressure gradients, blood and CSF flows under both normal conditions and communicating hydrocephalus. The analysis of intracranial pressure and its components to study CSF dynamics both in normal conditions and in various pathologies was discussed in [9]. A new model of water transport through the parenchyma from the microvasculature under the Starling force is proposed in [10]. The model predicts the effect of the osmolarity of the intercellular space, blood and CSF on cerebral water flow and establishes a link between osmotic imbalance and such pathological conditions as hydrocephalus and edema.

In [11], the cerebrovascular system was modeled as a set of resistors and capacitors to study Chiari malformation. The results of this work are also important for understanding the mechanism of spinal cord cyst formation. Another neurological disease caused by the formation of one or more macroscopic fluid-filled cavities in the spinal cord is syringomyelia [12, 13]. Using the compartmental approach, the model of a closed spinal cord system was created to study fluid transport in this pathology [12]. In [14], a model of the electrical circuit of the spinal cord CSF dynamics was created based on computational fluid mechanics techniques to study syringomyelia associated with Chiari malformation. The authors hypothesized that the loss of damping capacity of the cisterna magna and the resulting increase in pressure in the wall of the central channel lead to the formation of syrinx in patients with Chiari anomaly. A mathematical model of global arteriovenous circulation in the human body combined with a refined description of CSF dynamics in the craniospinal cavity is presented in [15]. The main innovation of this study is the connection of blood flow with CSF dynamics in the craniospinal cavity following the ideas presented in [8]. The resulting mathematical model was validated on real MRI data and applied to study transverse sinus stenosis and its association with idiopathic intracranial hypertension.

As can be seen from the above review of models, the information provided by such models is useful for the diagnostics and treatment of intracranial fluid dynamics disorders. However, models with concentrated parameters or divided into compartments do not allow taking into account spatial variations, such as velocity and pressure of fluid in a compartment.

The computational fluid dynamics (CFD) approach can be used to describe the hydrodynamics of cerebral and spinal CSF under normal and pathological conditions. CFD modeling uses a three-dimensional patient-specific geometry that allows us to compare directly simulation results with in-vivo data. In [16], the authors used CFD to investigate the pathogenesis of syringomyelia combined with Chiari malformation. It is shown that abnormal CSF velocities and pressure values are associated with abnormal CNS anatomy. Another study was aimed at determining how Chiari malformation and syringomyelia alter the pressure in the spinal subarachnoid space [17]. Using MRI data for geometry construction and CSF flow information, it was shown that in Chiari malformation, peak pressure may occur earlier and be significantly higher than that in the control group, while in patients with syringomyelia the pressure profiles are similar to those of the control group.

CFD modeling allows predicting pressure and CSF flow rate in the ventricular system and both in the spinal and cerebral subarachnoid space in healthy subjects and patients with acute or chronic hydrocephalus. Thus, in [18], the gradients of intracranial pressure and CSF velocity were determined throughout the craniospinal system of healthy subjects and patients with communicating hydrocephalus. The mathematical model described in this paper also captures the transition from normal intracranial dynamics to acute communicating hydrocephalus. In contrast to [18], in which the simulation was performed in a two-dimensional approximation, in [19], the authors investigated the relationship between vascular pulsations, flow, and pressure waves of CSF in the CNS using a three-dimensional modeling of CFD. The simulation results were in agreement with the clinical values. In [20], using three-dimensional FSI modelling, it was shown that ventricular volume and maximum CSF pressure are important hydrodynamic indicators in patients with non-communicating hydrocephalus and that the type of non-communicating hydrocephalus does not significantly affect the change in these two indicators. But it is worth noting that the CFD approach is quite time-consuming (especially FSI modeling) and does not allow describing a parenchymal component.

It is worth pointing out that modeling CSF flow through brain tissue is crucial for better understanding of various CNS diseases. Poroelastic models are considered as one of the options for modeling the brain parenchyma. This approach is used to simulate the interaction of CSF with the brain parenchyma and to test different hypotheses of the occurrence and development of CNS diseases. Thus, in an early work [21], it was shown that large differences in intracranial pressure between ventricles and subarachnoid space are responsible for the hydrocephalus development. The combination of the poroelastic parenchymal model with the CSF hydrodynamic model in the case of idealized geometry was considered in [22]. The resulting mathematical model allows us to study the onset, development and treatment of hydrocephalus. In [23], the authors focused on studying the effects of variable permeability as a function of strain. The work is focused on the reasonable of using mathematical models that account for strain-dependent permeability for more complex geometric configurations relevant to models of the clinical condition of hydrocephalus. The work [24] is dedicated to the study of acute hydrocephalus caused by stenosis of the cerebral aqueduct using a combination of poroelastic parenchyma model and a three-dimensional CFD model of the aqueduct.

The intensive study of the “cerebrospinal fluid-cerebral blood flow” connection made us realize to consider the intracranial hemo- and CSF dynamics and their influence on changes in cerebral ventricular configuration under physiological and pathological conditions. For example, a spherically symmetric poroelastic model of the brain with multiple fluids (CSF, arterial and venous blood) was developed to determine the spatial and temporal distribution of CSF pressure and brain tissue displacement during an infusion test [25, 26]. The approach described in [27] allowed us to study fluid transport between the blood circulation, CSF and brain parenchyma, as well as to consider hypotheses about the onset and progression of acute and chronic hydrocephalus. In [28] authors investigate the mechanical properties of the aging brain using an improved multi-fluid poroelasticity model [27]. In [29], this mathematical model includes the concept of the glymphatic system and is applied to study cerebral perfusion and neurovascular block in a three-dimensional anatomically accurate brain geometry.

Thus, the study of mathematical models of the interaction of cerebral fluids and the relationship between this interaction with ventricular wall deformation and intracranial pressure allows us to gain deeper understanding of pathological processes and to choose the patient management tactics more consciously.

## Materials and methods

**Fig 1:**
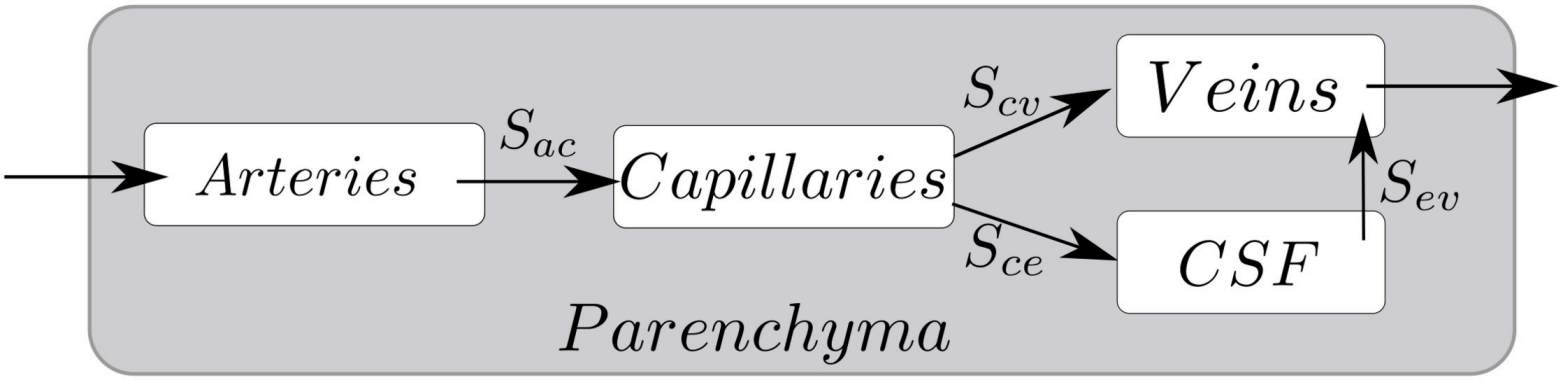
Brain fluid transport scheme. Scheme of blood and CSF transport in the brain parenchyma.

The poroelastic multifluid filtration model is used for mathematical modeling of cerebral hemo- and CSF dynamics [27–30]. The brain parenchyma is modeled by a porous matrix. Fluid phases are present at each point of the pore space and interact with each other. The model considers four phases: arterial blood (values with index *a*), capillary blood (values with index *c*), venous blood (values with index *v*), and cerebrospinal fluid (values with index *e*). The fluid exchange between the phases is shown in Fig 1. Each fluid phase has its own pressure. The model describes the distribution and mutual influence of the pressures in the fluid phases and the displacement of brain parenchyma under the influence of these pressures. Since the subject of the study is conditions developing in adulthood and having chronicity, a stationary mathematical model is considered.

**Fig 2:**
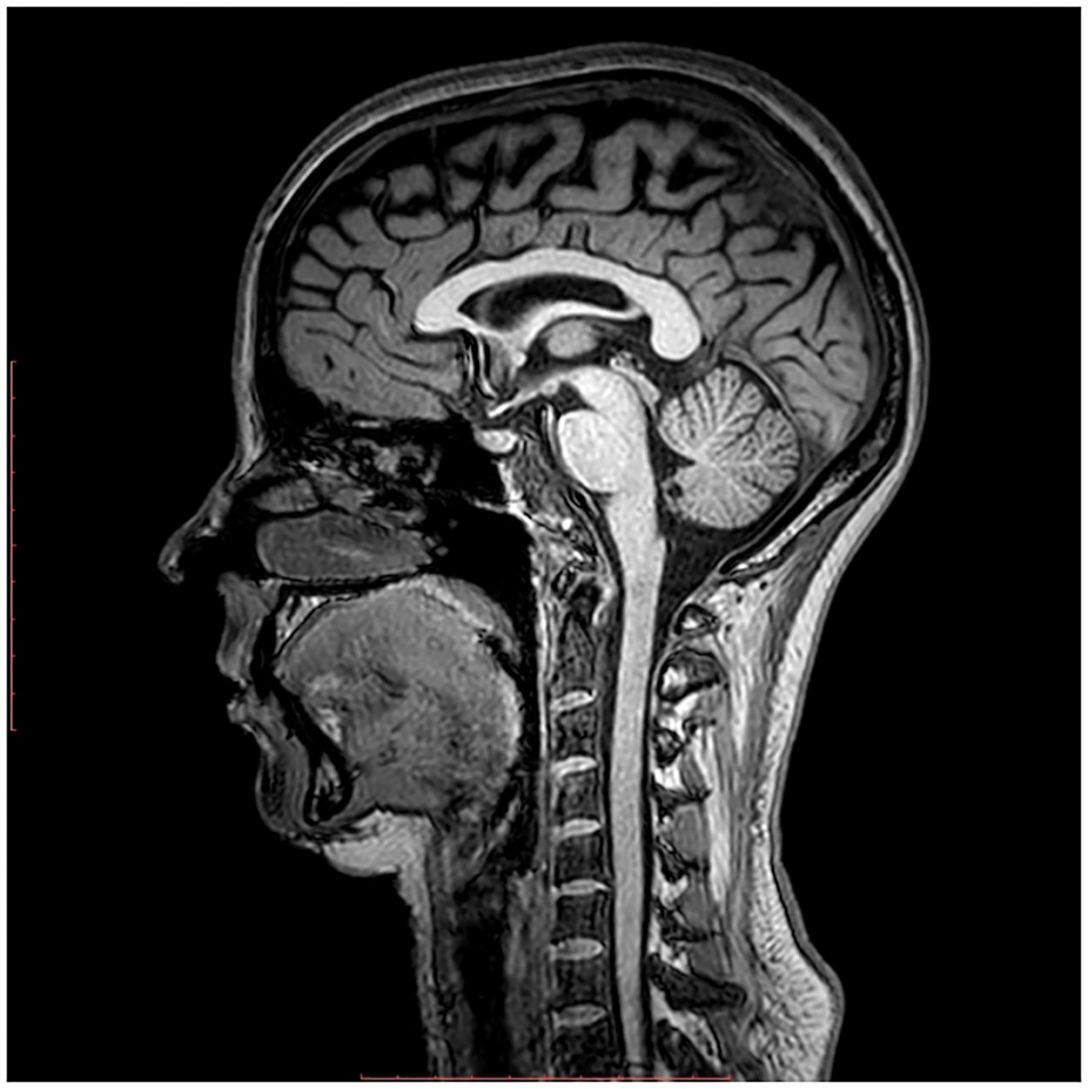
MRI data. Brain MRI saggital scan from one of the volunteer.

**Fig 3:**
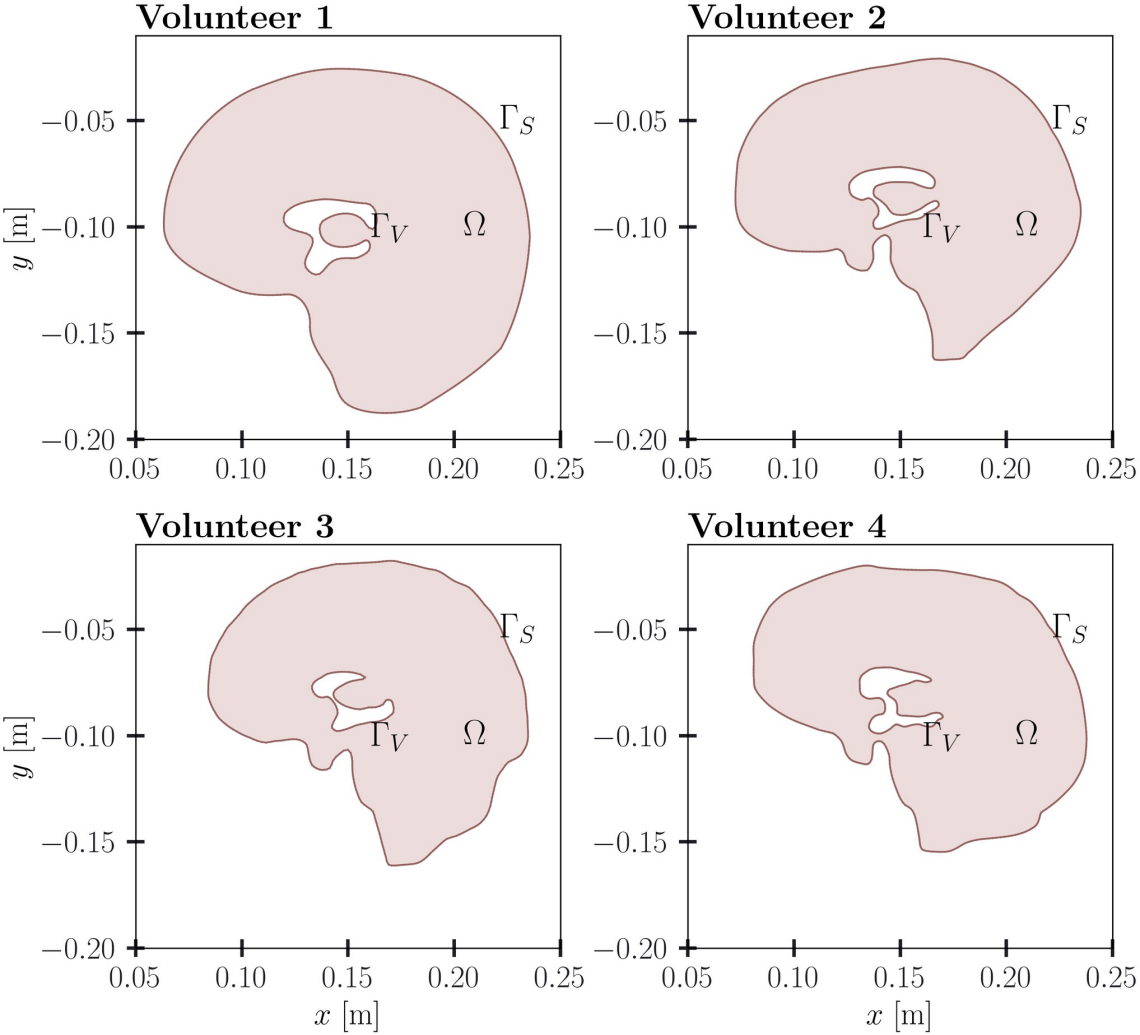
Geometry of the computational domain. Geometries of the computational domain for four volunteers.

Four subject-specific geometries of sagittal slices of the brain obtained from MRI data are included in this paper. The data for the first case was obtained in [31]. For the remaining three cases, real data was taken from volunteers without pathology examined at the ITC SB RAS by using Philips Ingenia tomograph of 3T field strength, Fig 2. This study was conducted in accordance with the Declaration of Helsinki, and approval was granted by the local committee on medical ethics of the International Tomography Center Siberian Branch of the Russian Academy of Sciences. All the volunteers gave their informed consent (in written form). In addition, the studies were supervised by the local ethical committee (ITC SB of RAS). A 3D T1-TFE sequence with a cubic voxel of size 0.55 [*mm*] was used to define the geometry of sagittal slice. The resulting geometry of the computational domain for the four volunteers is shown in Fig 3. Based on these clinical data, the mathematical modeling is performed in a two-dimensional approximation. It is worth noting that modeling brain processes using two-dimensional slices is a widely used approach [31–35]. The choice of the sagittal slice is due to the fact that it passes through the anatomical structures under study and lies in the symmetry plane that minimizes possible differences between two- and three-dimensional deformations (for three-dimensional deformations, this slice remains approximately plane).

This approach allows us to investigate how the interaction of fluid phases affects the displacement of the brain parenchyma and intracranial pressure. It allows us to expand our understanding of the possible mechanisms of hydrocephalus development and some other pathological conditions.

### Mathematical model

The following notation is introduced below. The boundary of the brain ventricles, which is the inner boundary of the computational domain, is denoted by Γ_*V*_. The outer boundary of the computational domain is denoted as Γ_*S*_ and corresponds to the skull. The brain parenchyma is referred to Ω, Fig 2. According to clinical studies, there is a difference between the mechanical properties of white matter and gray matter. However, to date, there are no relevant experimental data to quantify these differences. Therefore, the brain parenchyma is modeled as a homogeneous material [27, 36, 37]. The CSF movement in the subarachnoid space is taken into consideration by introducing averaged values. The intraventricular CSF flow and the CSF flow through the brain aqueduct are not considered directly, but their integral effect is taken into account. Therefore, the mathematical model includes one vector and four scalar equations [27]. The values used in the mathematical model are given in the Table 1.

**Table 1:** Values used in the model Eqs (1)–(13).

Symbol	Name	Model units	Analysis units
***u***	Brain tissue displacement	m	mm
*p*_*a*_, *p*_*v*_, *p*_*e*_, *p*_*c*_	Pore pressure fluids	Pa	mmHg
*E*	Young’s modulus	Pa	Pa
*ν*	Poison’s ratio	–	–
*α*_*a*_, *α*_*v*_, *α*_*e*_, *α*_*c*_	Biot coefficients	–	–
*k*_*a*_, *k*_*v*_, *k*_*e*_, *k*_*c*_	Permeability	m^2^	m^2^
*μ*_*a*_, *μ*_*v*_, *μ*_*e*_, *μ*_*c*_	Dynamic viscosity	$\frac{\text{N}\cdot \text{s}}{{\text{m}}^{2}}$	$\frac{\text{N}\cdot \text{s}}{{\text{m}}^{2}}$
*γ*_*ac*_, *γ*_*cv*_, *γ*_*ev*_, *γ*_*ce*_	Parameters specifying interactions and flows of pore fluids between basins	$\frac{{\text{m}}^{2}}{\text{N}\cdot \text{s}}$	$\frac{{\text{m}}^{2}}{\text{N}\cdot \text{s}}$
*Q*_*p*_	Constant rate of CSF secretion in the brain ventricles	$\frac{{\text{m}}^{3}}{\text{s}}$	$\frac{{\text{m}}^{3}}{\text{s}}$
*Q*_0_	CSF outflow into the venous network	$\frac{{\text{m}}^{3}}{\text{s}}$	$\frac{{\text{m}}^{3}}{\text{s}}$
*d*	Diameter of the brain aqueduct	m	mm
*L*	Length of the brain aqueduct	m	mm
*κ*_*cv*_	Flow resistance from the capillary into the ventricles via the vascular plexus	$\frac{{\text{m}}^{5}\cdot \text{s}}{\text{kg}}$	$\frac{{\text{m}}^{5}\cdot \text{s}}{\text{kg}}$
*R*	Resistance due to the presence of arachnoid granulations	$\frac{1}{m^{3}}$	$\frac{1}{m^{3}}$

The equilibrium vector equation for brain parenchyma is [38]

$$\begin{array}{c} \mu \Delta u+\left(\mu +\lambda \right)\nabla \left(\text{div}\;u\right)-(\alpha_{a}\nabla p_{a}+\alpha_{c}\nabla p_{c}+\alpha_{e}\nabla p_{e}+\alpha_{v}\nabla p_{v})=0. \end{array}$$ (1)

Using the mass conservation law and Darcy’s law for pore fluids, we obtain equations for pressure values

$$\begin{array}{c} -\frac{k_{a}}{\mu_{a}}\Delta p_{a}-\gamma_{ac}\left(p_{c}-p_{a}\right)=0, \end{array}$$ (2)

$$\begin{array}{c} -\frac{k_{v}}{\mu_{v}}\Delta p_{v}+\gamma_{cv}\left(p_{v}-p_{c}\right)+\gamma_{ev}\left(p_{v}-p_{e}\right)=0, \end{array}$$ (3)

$$\begin{array}{c} -\frac{k_{e}}{\mu_{e}}\Delta p_{e}+\gamma_{ce}\left(p_{e}-p_{c}\right)-\gamma_{ev}\left(p_{v}-p_{e}\right)=0, \end{array}$$ (4)

$$\begin{array}{c} -\frac{k_{c}}{\mu_{c}}\Delta p_{c}+\gamma_{ac}\left(p_{c}-p_{a}\right)-\gamma_{ce}\left(p_{e}-p_{c}\right)-\gamma_{cv}\left(p_{v}-p_{c}\right)=0; \end{array}$$ (5)

Here ***u*** is brain parenchyma displacement, λ and *μ* are elastic moduli, *p*_*i*_ is the *i*-th pore fluid pressure, *α*_*i*_ are Biot’s coefficients, *k*_*i*_ are permeability coefficients, *μ*_*i*_ is pore fluid viscosity, *i* = *a*, *c*, *v*, *e*. The terms of the form *S*_*yx*_ = *γ*_*yx*_(*p*_*x*_ − *p*_*y*_) describe the fluid transport from the network *x* to the network *y* due to a hydrostatic pressure gradient. Here *γ*_*yx*_ are parameters specifying interactions and flows of pore fluids between basins. Below, we will refer to *γ*_*yx*_ as interaction parameters.

The system of equations Eqs (1)–(5) is complemented by boundary conditions for displacement and four pore pressure values. At the cerebral ventricular boundary Γ_*V*_ the following conditions are set.

1. Stresses are assumed to be continuous:

$$\begin{array}{c} 2\mu \varepsilon \left(u\right)\cdot n+\lambda \epsilon \left(u\right)n=\sum_{i=a,c,e,v}(\alpha_{i}-1)p_{i}n \end{array}$$ (6)

*ε*(***u***) is a strain tensor; *ϵ*(***u***) = tr*ε*(***u***) = *ε*(***u***)_*ii*_ = div ***u***; ***n*** is an external unit normal vector.

2. There is no flow for the arterial and venous networks:

$$\begin{array}{c} \nabla p_{a}n=\nabla p_{v}n=0; \end{array}$$ (7)

3. CSF is secreted at constant rate *Q*_*p*_ in the brain ventricles. The conservation condition of fluid mass in the ventricular system takes into account the CSF volume produced by the vascular plexuses, the CSF volume that seeps through the ventricular wall, and the CSF outflow through the brain aqueduct

$$\begin{array}{c} Q_{p}=\frac{\pi d^{4}}{128\mu L}(p_{e}\left|\Gamma_{V}-p_{e}\right|\Gamma_{S})-\oint_{\Gamma_{V}}(-\frac{k_{e}}{\mu_{e}}\nabla p_{e})\cdot ndS \end{array}$$ (8)

*d*, *L* are diameter and length of the brain aqueduct.

4. The CSF formation from blood leads to a drop in capillary network pressure:

$$\begin{array}{c} \kappa_{cv}\nabla p_{c}n=Q_{p}, \end{array}$$ (9)

where *κ*_*cv*_ is flow resistance from the capillary network into the ventricles via the vascular plexus.

At the skull boundary Γ_*S*_, the following assumptions are accepted.

1. Since this paper considers the adult brain, the skull is considered rigid. Thus, the displacements of the skull boundary are equal to zero:

$$\begin{array}{c} u=0. \end{array}$$ (10)

2. No capillary flow is at the skull boundary:

$$\begin{array}{c} \nabla p_{c}n=0, \end{array}$$ (11)

3. Arterial and venous pressure values are set:

$$\begin{array}{c} p_{a}=p_{art},\;p_{v}=p_{ven}. \end{array}$$ (12)

4. CSF absorption into the venous network leads to an increase in pressure:

$$\begin{array}{c} p_{e}=p_{v}+\mu_{e}RQ_{0}, \end{array}$$ (13)

where *R* is resistance due to the presence of arachnoid granulations; *Q*_0_ is CSF outflow into the venous network, *μ*_*e*_ is CSF viscosity.

The values of the parameters characterizing the pore fluids and the porous matrix are given in Table 2 according to the literature data [22, 24].

**Table 2:** Parameters used in the model Eqs (1)–(13).

Parameter	Value	Parameter	Value
*E*	584 Pa [39]	*p*_*ven*_	650 Pa
*ν*	0,35	*Q*_*p*_, *Q*_0_	$5,8\cdot 10^{-9}\frac{{\text{m}}^{3}}{\text{s}}$
*k*_*a*,*c*,*v*_	10^−10^ m^2^	*d*	4 mm
*k*_*e*_	1,4 ⋅ 10^−14^ m^2^	*L*	70 mm
*μ*_*a*,*c*,*v*_	$2,67\cdot 10^{-3}\frac{\text{N}\cdot \text{s}}{{\text{m}}^{2}}$	*R*	$8,5\cdot 10^{13}\frac{1}{{\text{m}}^{3}}$
*μ*_*e*_	$8,9\cdot 10^{-4}\frac{\text{N}\cdot \text{s}}{{\text{m}}^{2}}$	*κ*_*cv*_	$6\cdot 10^{-4}\frac{{\text{m}}^{5}\cdot \text{s}}{\text{kg}}$ [24, 40]
*p*_*art*_	8000 Pa	*α*_*a*,*e*,*c*,*v*_	0,99

Given Young’s modulus *E* and Poisson’s ratio *ν*, the elastic moduli λ and *μ* are calculated using the well-known formulas:

$$\begin{array}{c} \lambda =\frac{\nu E}{\left(1+\nu )(1-2\nu \right)} \end{array}$$ (14)

$$\begin{array}{c} \mu =\frac{E}{2(1+\nu )} \end{array}$$ (15)

To solve Eqs (1)–(5) numerically with boundary conditions of Eqs (6)–(13), the finite element method was used. The calculations were performed in the open package FreeFem++ [41]. The weak formulation of the problem is given in S1 Appendix.

Within the framework of the considered model, the celebral hemo- and CSF dynamics is defined by the values of parameters *γ*_*xy*_ describing the interaction and mutual cross-flows of fluid phases. Further in the paper, the effect of *γ*_*xy*_ parameters on ventricular wall displacement and pressure distribution on it is investigated using the numerical solution of the system Eqs (1)–(13). For this purpose, each of the interaction parameters *γ*_*xy*_ was independently provided by 15 values from the range: $10^{-16}\frac{m^{2}}{N\cdot s}:10^{-8}\frac{m^{2}}{N\cdot s}$. The formulation of a weak problem was solved numerically for all 15^4^ = 50625 parameter sets *γ*_*ac*_, *γ*_*cv*_, *γ*_*ce*_, *γ*_*ev*_. It should be noted that these parameter values may cover the entire physiologically acceptable range of periventricular pressure [42].

### Statistical analysis

Based on the results of the numerical calculations, a statistical analysis was performed using the free software environment for statistical computing and graphics *R* [43], R version is 4.0.3.

The goal of the statistical analysis was to investigate the influence of the interaction of cerebral fluids on the mean displacement of the ventricular wall. In selecting a model, we preferred high interpretability over predictive power. For this purpose, a fairly simple statistical model was chosen that had both high interpretability and described the data well. Therefore, to study the effects of the values of the interaction parameters *γ*_*xy*_ on the mean displacement $\bar{u}$ of the ventricular wall, a multiple linear regression with interaction (MLR model) was constructed for each of the four volunteers. To construct the regression, the *γ*_*xy*_ values were logarithmically pre-transformed.

The logarithmic transformation was applied to normalized data and is described as

$$\begin{array}{c} \psi_{**}=\text{log}\;(b+\frac{\gamma_{**}-\text{min}\;\gamma_{**}}{\text{max}\;\gamma_{**}-\text{min}\;\gamma_{**}}), \end{array}$$ (16)

where the choice of parameter *b* is described below. The data obtained was filtered out: only those sets *γ*_*xy*_ for which used to construct the regression, where the capillary pressure value on the ventricular wall ranged from 5 *mmHg* (666.61 *Pa*) up to 40 *mmHg* (5332.88 *Pa*) [42].

This range will be further referred to physiologically permissible. Outside this range, irreversible changes usually leading to a fatal outcome occur.

In order to choose the regression model used later to describe the mean ventricular wall displacement, (2^10^ − 1) linear models with all possible combinations of the following regressors were considered:

$$\begin{array}{c} \psi_{ac},\;\psi_{ce},\;\psi_{ev},\;\psi_{cv},\;\psi_{ac}\cdot \psi_{ce},\;\psi_{ac}\cdot \psi_{ev},\;\psi_{ac}\cdot \psi_{cv},\;\psi_{ce}\cdot \psi_{cv},\;\psi_{cv}\cdot \psi_{ev},\;\psi_{ce}\cdot \psi_{ev}. \end{array}$$ (17)

These regressors allow us to consider the influence of cross-flow between the fluid phases and the interactions between these cross-flows on the mean displacement of the ventricular wall.

For each of the models, the transformation of Eq 16 was optimized in two ways: by finding a value *b* that yields the maximum value of $R_{adj.}^{2}$ and that gives the minimum value of the Akaike AIC information criterion. The optimization was performed using the iterative L-BFGS-B method. This uses a limited-memory modification of the BFGS quasi-Newton method [44]. At each iteration, a linear regression was fitted and the corresponding value of $R_{adj.}^{2}$ or AIC was calculated. In the optimization, *b* ∈ [0, 10] was assumed. It should be noted that for all models the optimal values of *b* found are not on the boundary of this segment.

The resulting models were ordered in descending order $R_{adj.}^{2}$. For convenience, the 25 models with the highest value of the coefficient of determination are given below for each volunteer. The same models have the lowest values of the Akaike information criterion. The corresponding optimal values of the coefficient *b* obtained by two methods for these 25 models differ by less than 0.1%. The S1–S4 Files shows the results of this algorithm for all 1023 models, ordered by decreasing $R_{adj.}^{2}$. This table also contains the optimal values *b* obtained by maximizing $R_{adj.}^{2}$, denoted as *b*_*opt*_.

**Fig 4:**
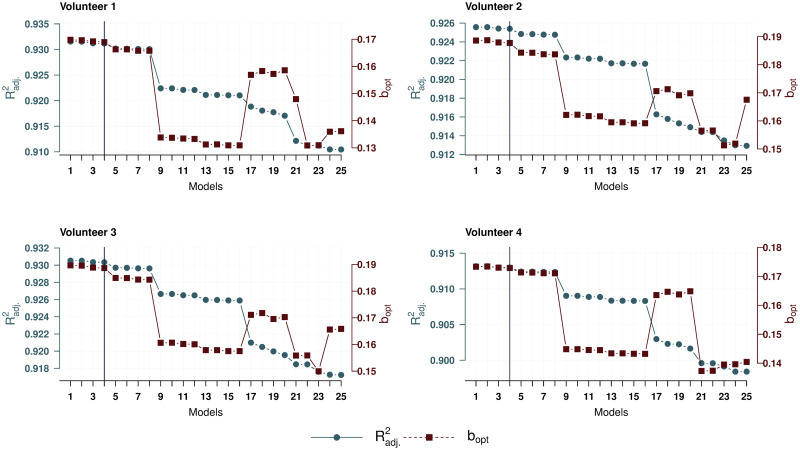
Values of *b*_*opt*_ and $R_{adj.}^{2}$. Values of *b*_*opt*_ and $R_{adj.}^{2}$ of the first 25 models for four volunteers.

**Fig 5:**
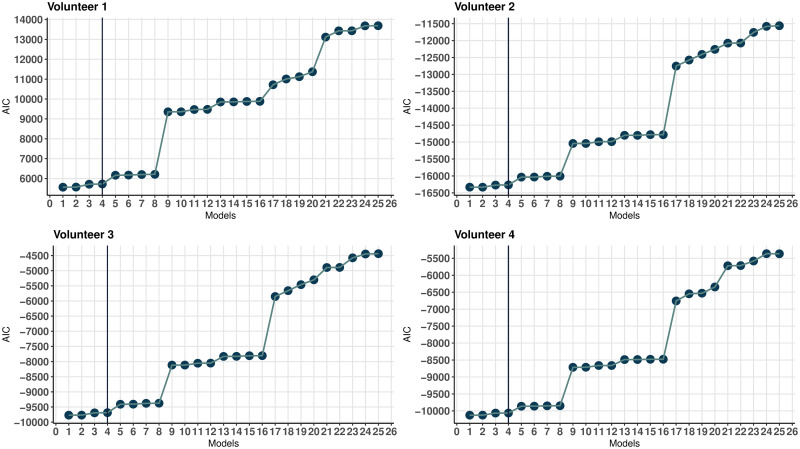
Values of the Akaike information criterion. Values of the Akaike information criterion of the first 25 models for four volunteers.

Based on the above data, let us choose a model that is both simple enough and describes the data well. As can be seen in Fig 4, the optimal values *b*_*opt*_ for the first eight models with the highest coefficients of determination $R_{adj.}^{2}$ differ in the third decimal place. Therefore, it would be correct to compare these eight models for each of the volunteers. Fig 5 shows the values of the Akaike information criterion. From these two figures, we can see that the first four models best represent the dependency under consideration and are qualitatively close to the same. The simplest of these four models is model 4, which will be futher used as a base model. This model is the same for all volunteers, and in contrast to the full model by the absence of the regressors

$$\begin{array}{c} \psi_{cv}\cdot \psi_{ev},\;\psi_{ce}\cdot \psi_{ev}. \end{array}$$ (18)

**Fig 6:**
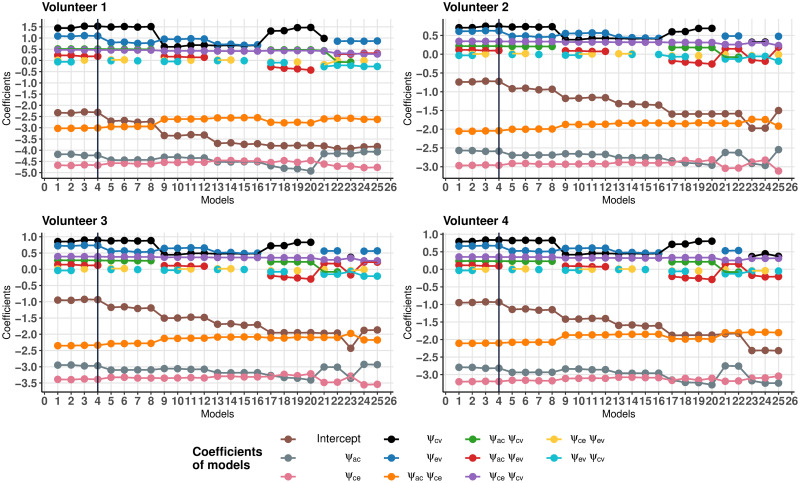
Regression models coefficients. Regression coefficients of the first 25 models for four volunteers.

Fig 6 shows the values of the regression coefficients. The coefficients for the regressors missing in model No. 4 are an order of magnitude smaller than the other regression coefficients. Thus, the excluded regressors make a relatively small contribution to the mean ventricular wall displacement and can be considered unessential.

For each volunteer, the mean ventricular wall displacement has an approximately normal distribution and equals: 2.99 (1.01) for Volunteer 1, 2.34 (0.68) for Volunteer 2, 2.56 (0.78) for Volunteer 3 and 2.58 (0.69) for Volunteer 4. Values are expressed as mean (SD).

For the chosen base model, all regression coefficients $\hat{\beta }$ are statistically significant (*p* < 0.001) for all volunteers. The values of the coefficients, their standard errors and confidence intervals, the values of the dfbeta analysis are given in Table 3. Here dfbeta* is the maximum of the absolute dfbeta analysis values.

**Table 3:** MLR model result for four volunteers.

**MLR model for volunteer 1**					**MLR model for volunteer 2**				
	$\hat{\beta }$	*SE*	*CI*	*dfbeta* *		$\hat{\beta }$	*SE*	*CI*	dfbeta*
*Intercept*	-2.313	0.030	[-2.372, -2.253]	0.0192	*Intercept*	-0.725	0.019	[-0.762, -0.689]	0.0116
*ψ*_*ac*_	-4.226	0.019	[-4.262, -4.19]	0.0111	*ψ*_*ac*_	-2.585	0.012	[-2.609, -2.562]	0.0071
*ψ*_*ce*_	-4.670	0.015	[-4.699, -4.641]	0.0065	*ψ*_*ce*_	-2.963	0.009	[-2.981, -2.945]	0.0037
*ψ*_*ev*_	1.091	0.014	[1.064, 1.118]	0.0052	*ψ*_*ev*_	0.623	0.009	[0.604, 0.641]	0.0038
*ψ*_*cv*_	1.526	0.013	[1.50, 1.552]	0.005	*ψ*_*cv*_	0.747	0.009	[0.730, 0.765]	0.0034
*ψ*_*ac*_⋅*ψ*_*ce*_	-3.009	0.009	[-3.028, -2.991]	0.0036	*ψ*_*ac*_⋅*ψ*_*ce*_	-2.042	0.006	[-2.054, -2.030]	0.0021
*ψ*_*ac*_⋅*ψ*_*ev*_	0.185	0.008	[0.168, 0.201]	0.0031	*ψ*_*ac*_⋅*ψ*_*ev*_	0.098	0.006	[0.086, 0.110]	0.0024
*ψ*_*ac*_⋅*ψ*_*cv*_	0.510	0.008	[0.495, 0.525]	0.0027	*ψ*_*ac*_⋅*ψ*_*cv*_	0.215	0.006	[0.204, 0.226]	0.002
*ψ*_*ce*_⋅*ψ*_*cv*_	0.471	0.004	[0.462, 0.480]	0.0005	*ψ*_*ce*_⋅*ψ*_*cv*_	0.343	0.0030	[0.337, 0.350]	0.0004
**MLR model for volunteer 3**					**MLR model for volunteer 4**				
	$\hat{\beta }$	*SE*	*CI*	dfbeta*		$\hat{\beta }$	*SE*	*CI*	dfbeta*
*Intercept*	-0.936	0.021	[-0.976, -0.895]	0.013	*Intercept*	-0.935	0.022	[-0.978, -0.891]	0.0133
*ψ*_*ac*_	-2.968	0.013	[-2.994, -2.942]	0.0079	*ψ*_*ac*_	-2.816	0.014	[-2.843, -2.789]	0.0078
*ψ*_*ce*_	-3.387	0.010	[-3.408, -3.367]	0.0042	*ψ*_*ce*_	-3.196	0.011	[-3.217, -3.176]	0.0041
*ψ*_*ev*_	0.7300	0.011	[0.709, 0.751]	0.0043	*ψ*_*ev*_	0.6710	0.011	[0.650, 0.692]	0.0041
*ψ*_*cv*_	0.8990	0.010	[0.879, 0.918]	0.0039	*ψ*_*cv*_	0.8350	0.010	[0.816, 0.854]	0.0034
*ψ*_*ac*_⋅*ψ*_*ce*_	-2.338	0.007	[-2.351, -2.324]	0.0024	*ψ*_*ac*_⋅*ψ*_*ce*_	-2.101	0.007	[-2.114, -2.087]	0.0023
*ψ*_*ac*_⋅*ψ*_*ev*_	0.1210	0.007	[0.108, 0.134]	0.0027	*ψ*_*ac*_⋅*ψ*_*ev*_	0.0960	0.006	[0.083, 0.109]	0.0025
*ψ*_*ac*_⋅*ψ*_*cv*_	0.2730	0.006	[0.261, 0.286]	0.0022	*ψ*_*ac*_⋅*ψ*_*cv*_	0.2350	0.006	[0.224, 0.247]	0.0019
*ψ*_*ce*_⋅*ψ*_*cv*_	0.3880	0.004	[0.381, 0.396]	0.0004	*ψ*_*ce*_⋅*ψ*_*cv*_	0.3530	0.004	[0.346, 0.359]	0.0004

Model coefficients ($\hat{\beta }$), standard error estimates (SE), confidence intervals (CI), and values from dfbeta analysis (dfbeta*) of the MLR model for four volunteers.

The estimated explanatory power of regression model $\bar{u}$ for volunteer 1 is 93.0%, F(8, 30250) = 51177.6, p < 0.001. Further, the explanatory power of regression model $\bar{u}$ for volunteer 2 is 92.5%, F(8, 30437) = 47210.16, p < 0.001. The explanatory power of regression model $\bar{u}$ for volunteer 3 is 93.0%, F(8, 30453) = 50850.9, p < 0.001. In addition, the explanatory power of regression model $\bar{u}$ for volunteer 4 is 91.3%, F(8, 30329) = 39791.9, p < 0.001.

The additional characteristics of the base regression model for four volunteers can be found in Table 4. This table provides information about the sample size for each volunteer, the value of *b*_*opt*_, and the value of the maximum correlation coefficient between regressors—*corr*_*max*_. The resulting regression model was validated using 10-fold cross-validation with RMSE cost function (*CV* value in the table) and the value of adjusted *R*^2^.

**Table 4:** Additional characteristics of the base regression model for four volunteers.

	*n*	*b*_*opt*_	*corr*_*max*_	*CV*	$R_{adj.}^{2}$
Volunteer 1	30259	0.1689	0.44	0.266	0.931
Volunteer 2	30446	0.1877	0.46	0.185	0.925
Volunteer 3	30462	0.1886	0.47	0.206	0.930
Volunteer 4	30338	0.1729	0.42	0.205	0.913

Sample size (*n*), optimal *b* corresponding to the model (*b*_*opt*_), value of maximum correlation coefficient between regressors (*corr*_*max*_), 10-fold cross-validation error (*CV*) and adjusted *R*^2^.

To verify the assumptions of the regression analysis for each of the volunteers, the dependence of the residuals on the predicted values and the histogram of the residuals are shown in S5 File. The figures show that the regression assumptions are satisfied.

## Results

### Analysis of calculation results

The computational results allow us to identify and analyze the patterns of influence of the interaction parameters on the pressures and mean displacement of the ventricular wall. From the results of numerical calculations and the constructed regression model, we find that the values of pressure and ventricular wall displacement depend on the interaction parameters in a logarithmic manner. Therefore, we introduce logarithmic interaction parameters *g*_*xy*_ = lg*γ*_*xy*_ for further analysis. Also, in analyzing the results, units are used that are common in medical practice and more convenient for interpretation (column “Analysis units”, Table 1).

#### Analysis of fluid phase pressures

Arterial, venous, and CSF pressure values remain approximately constant at the ventricular boundary within the range of changes in the interaction parameters and equal: *p*_*a*_ = 60.15 mmHg (8019.3183 *Pa*), *p*_*v*_ = 4.88 mmHg (650.61136 *Pa*), *p*_*e*_ = 8.18 mmHg (1090.57396 *Pa*) The variation in these pressure values does not exceed 0.025 mmHg. (3.33305 *Pa*) The pressure values correspond to the physiological norm [42]. Thus, in within the framework of the model, the interaction of the cerebral fluid media with each other does not affect arterial, venous, and CSF pressure values. This results from the fact that this model does not take into account the mechanisms of cerebral autoregulation.

The capillary pressure depends on the values of the interaction parameters, and for a fixed set of parameter values the variation of this pressure at the ventricular wall does not exceed 0.0001 mmHg (0.0133322 *Pa*). When the interaction parameters are changed, the capillary pressure completely covers the physiological range. Further analysis of the calculation results is given on the example of the second volunteer, for whom the plots are the most visual. For the other volunteers, the qualitative conclusions are similar, and the corresponding illustrations are given in the S6 File.

**Fig 7:**
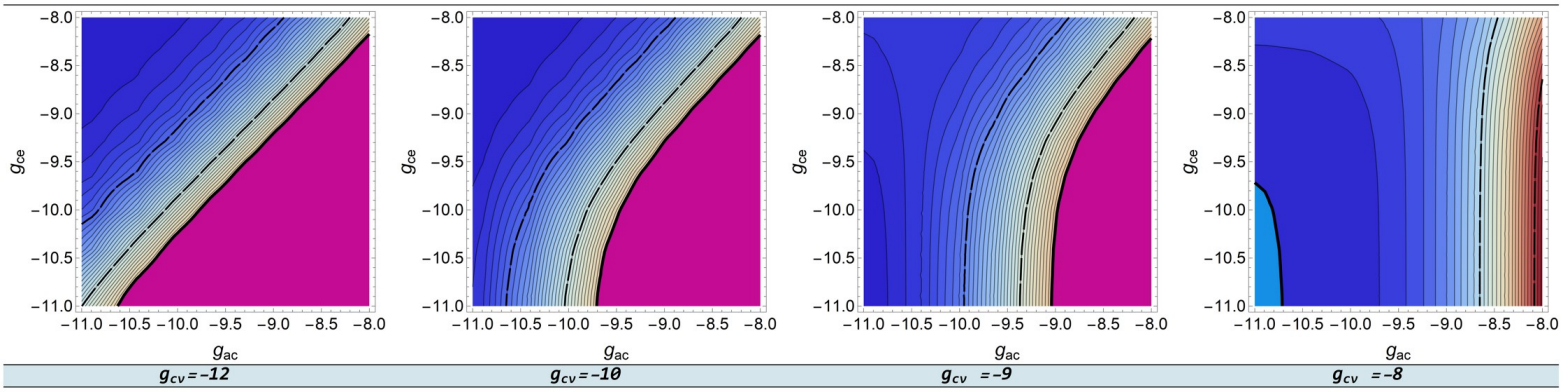
Dependence of *p*_*c*_ on *g*_*xy*_. Capillary pressure dependence on the logarithmic interaction parameters.

The behavior of capillary pressure *p*_*c*_ depending on the interaction parameters can be interpreted in the context of physiology. A change in the logarithmic parameter of the CSF-venous interaction *g*_*ev*_ has almost no effect on the capillary pressure. The influence of the other parameters is shown in Fig 7. Here, capillary pressure level lines are shown in 1 *mmHg* steps: the fill color corresponds to the pressure value, magenta corresponds to *p*_*c*_ > 40 *mmHg*, cyan − *p*_*c*_ < 5 *mmHg*. The dashed lines limit the capillary pressure range to 15 *mmHg*(1999.83 *Pa*) < *p*_*c*_ < 30 *mmHg*(3999.66 *Pa*), which corresponds to the capillary pressure range for a healthy body. At the left of Fig 7 we see that at small values of the logarithmic capillary-venous interaction parameter *g*_*cv*_, the simultaneous increase or decrease of the arterial-capillary *g*_*ac*_ and capillary-CSF *g*_*ce*_ parameters has almost no effect on pressure. This seems to be physiological, because in such a case the capillary blood volume changes slightly. As *g*_*cv*_ increases, the parameter *g*_*ce*_ has less and less influence on the capillary pressure, because both parameters are responsible for the capillary blood drain.

#### Analysis mean ventricular wall displacement

**Fig 8:**
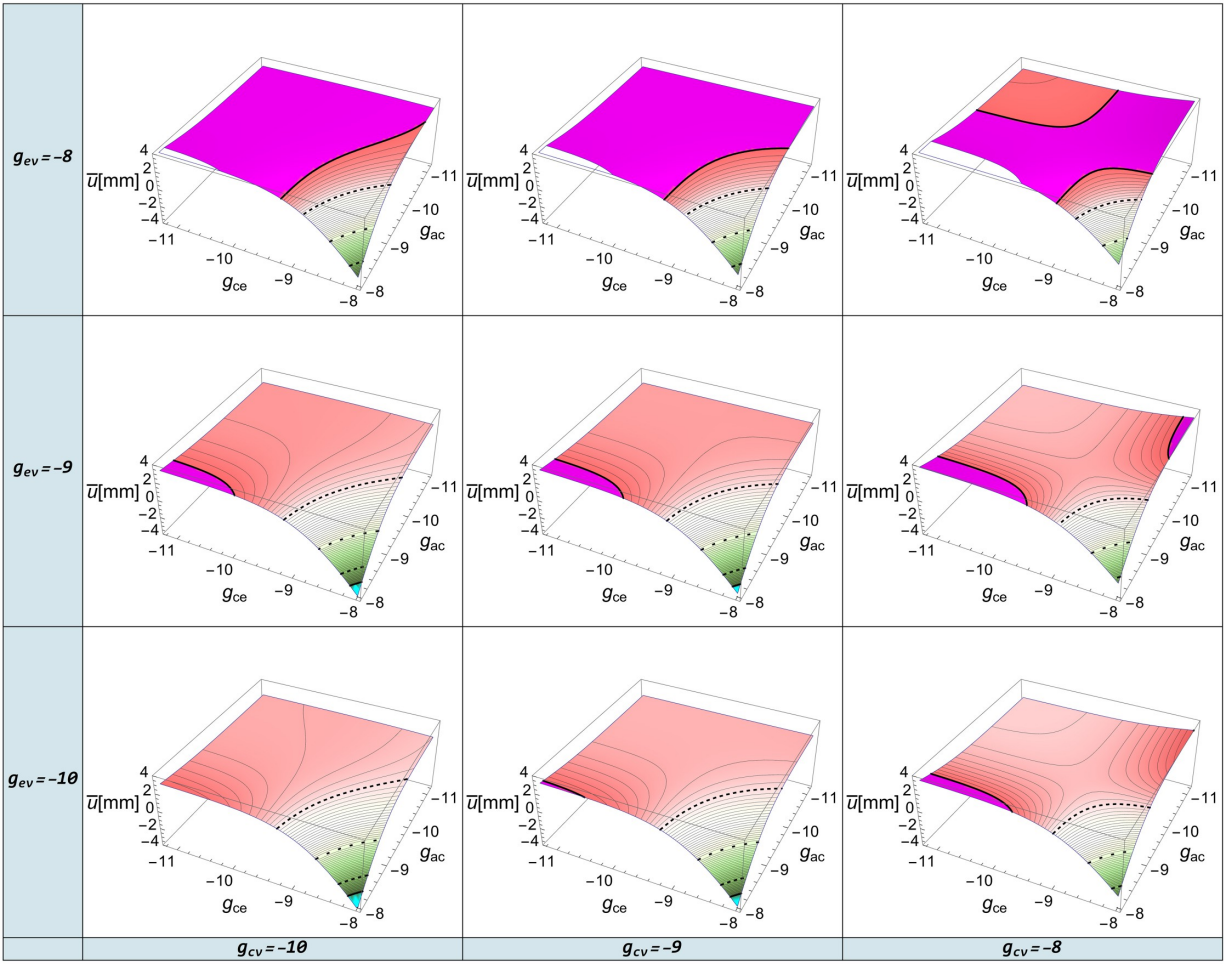
Dependence of $\bar{u}$ on the *g*_*xy*_. Mean ventricular wall displacement dependence on the logarithmic interaction parameters.

The plots of dependence of mean ventricular wall displacement on the logarithmic interaction parameters are shown in Fig 8. The plots are based on data from the second volunteer. Similar plots for the other volunteers are qualitatively identical and are shown in the S6 File. In Fig 8, the lines on the surface correspond to values of mean ventricular wall displacement in increments of 0.1 *mm*. The bold solid lines correspond to values of $\bar{u}=\pm 3\;mm$ (0.003*m*). This range was chosen basing on experience from clinical studies of hydrocephalus [5, 45]. Magenta highlights the area where $\bar{u}>3\;mm$ and cyan illustrates the area where $\bar{u}<-3\;mm$ (-0.003*m*). In the range [−3 *mm*, 3 *mm*], the fill color corresponds to the value of $\bar{u}$. The dashed lines correspond to the values $\bar{u}=\pm 2\;mm$, and the dotted line shows $\bar{u}=0\;mm$.

The plots in Fig 8 are almost symmetrical with respect to the parameters *g*_*ce*_ and *g*_*ac*_, showing their comparable effect on $\bar{u}$. This appears to be physiological, as an increase in either of these parameters increases the CSF content in the parenchyma resulting in ventricular compression and decrease in ventricular size.

A growth in *g*_*ev*_ increases the CSF drain from the parenchyma into the venous channel, resulting in enlargement of the ventricles. As *g*_*cv*_ increases, the ventricles dilate. Such a behavior is consistent with the decrease in *p*_*c*_ at the ventricular boundary.

For small values of *g*_*ce*_ and *g*_*ac*_, there is a complex behavior of mean ventricular wall displacement that is most clearly observed at small values of *g*_*ev*_. In this area of parameters, the surface becomes saddle-shaped. It is difficult to interpret this phenomenon, but, at the same time the variation of the mean ventricular wall displacement at these parameter values is relatively small, i.e. about one millimeter.

### Analysis of regression model of mean ventricular wall displacement

**Fig 9:**
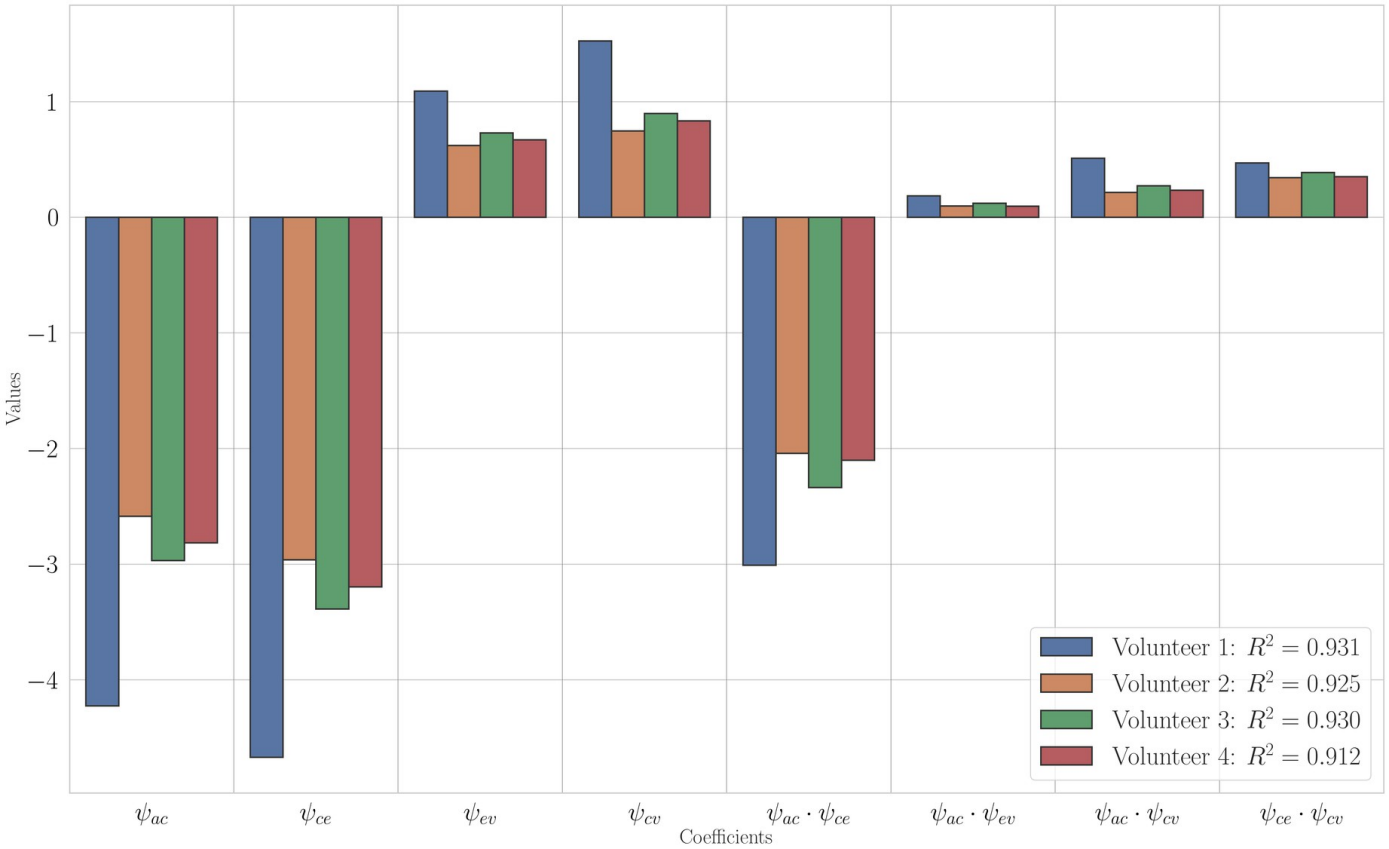
Values of estimation of the regression models. Values of regression coefficients.

The constructed regression model allows us to analyze the comparative effect of the interaction parameters *γ*_*xy*_ on the mean ventricular wall displacement. The values of the regression coefficients for all four volunteers are shown in Fig 9. It should be noted that the values of the regression coefficients for all volunteers show the same pattern.

In what follows the regression coefficients are discussed in descending order of their contribution.

- The largest contribution to the displacement is made by the arterial—CSF component, the influence of which is described by the coefficients at the regressors *ψ*_*ac*_, *ψ*_*ce*_ and *ψ*_*ac*_⋅*ψ*_*ce*_. And the regression coefficient at *ψ*_*ac*_⋅*ψ*_*ce*_, describing the mutual influence of the arterial-capillary and capillary-CSF components is comparable to the influence of each of these components separately. This leads to a strong nonlinearity of the influence of the arterial-CSF component.
- Venous outflow is the next in degree of influence (regression coefficients at *ψ*_*ev*_ and *ψ*_*cv*_).
- The mutual influence of capillary-venous outflow with the arterial-CSF component makes even smaller contribution (regression coefficients at *ψ*_*ac*_⋅*ψ*_*cv*_ and *ψ*_*ce*_⋅*ψ*_*cv*_).
- The smallest contribution comes from the mutual influence of arterial-capillary inflow and CSF-venous outflow (regression coefficient at *ψ*_*ac*_⋅*ψ*_*ev*_).

Thus, the parenchymal arterial-CSF component is crucial for the ventricular wall deformation.

#### The regression model clinical interpretation

The model reveals trends that correlate with the pattern of pathological states. The following plots show the values of the regression terms ($\hat{\psi_{xy}}=\hat{\beta_{xy}}\cdot \psi_{xy}$, where $\hat{\beta_{xy}}$ are regression coefficients) and the corresponding value of the mean ventricular wall displacement ($\bar{u}$).

**Pattern 1: normal pressure hydrocephalus**

**Fig 10:**
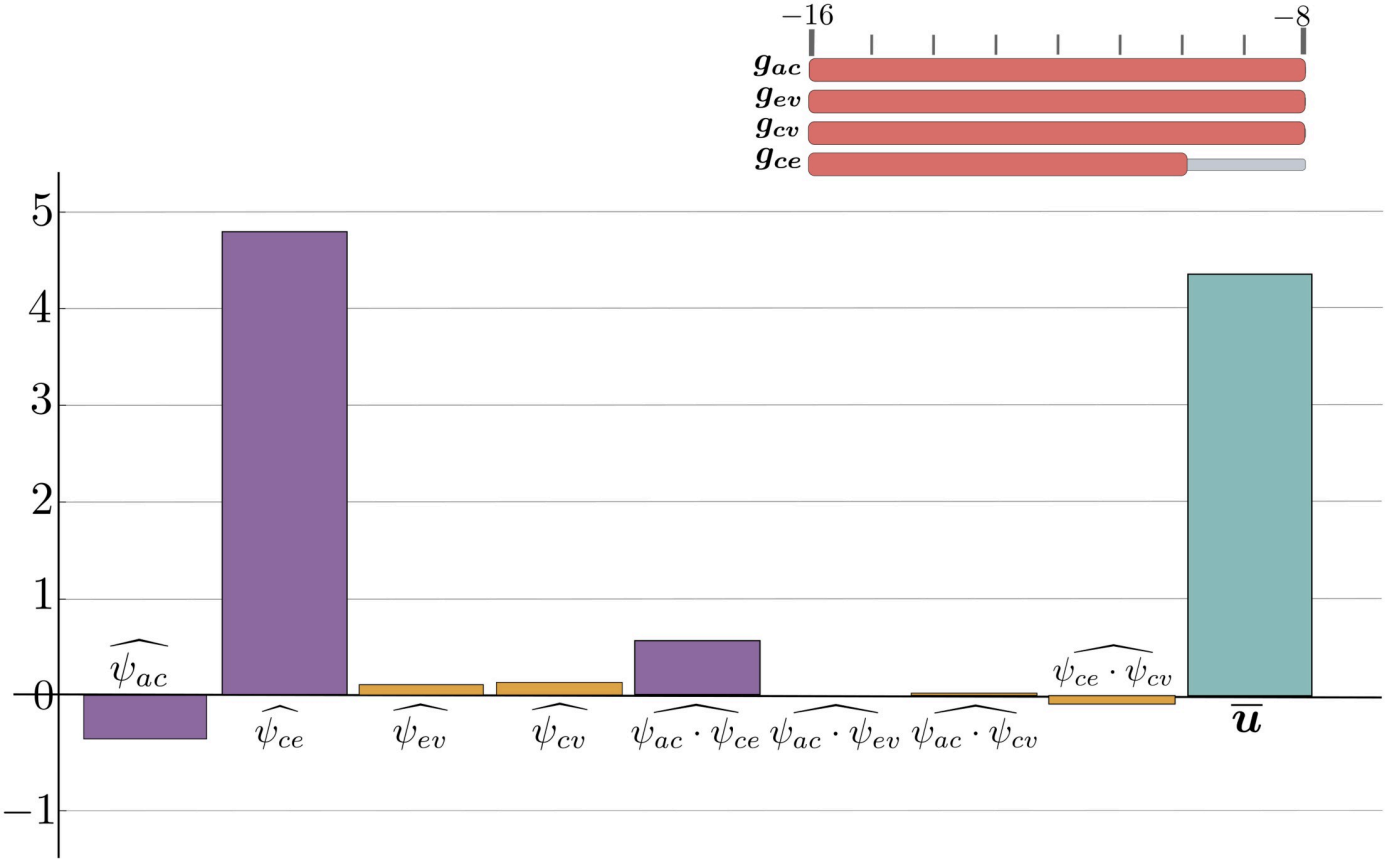
The regression terms for normal pressure hydrocephalus. Possible regression terms for normal pressure hydrocephalus.

When arterial-capillary inflow and capillary-venous outflow are preserved, the decrease in capillary-CSF flow leads to an increase in ventricular size. This is due to a decrease in blood flow to the interstitial and cerebrospinal fluids, which reduces the overall pressure on the ventricular wall and allows them to expand. Such changes may describe normal pressure hydrocephalus, in which ventricular dilation occurs without a significant increase in intracranial pressure Fig 10.

**Pattern 2: intracranial hypertension**

**Fig 11:**
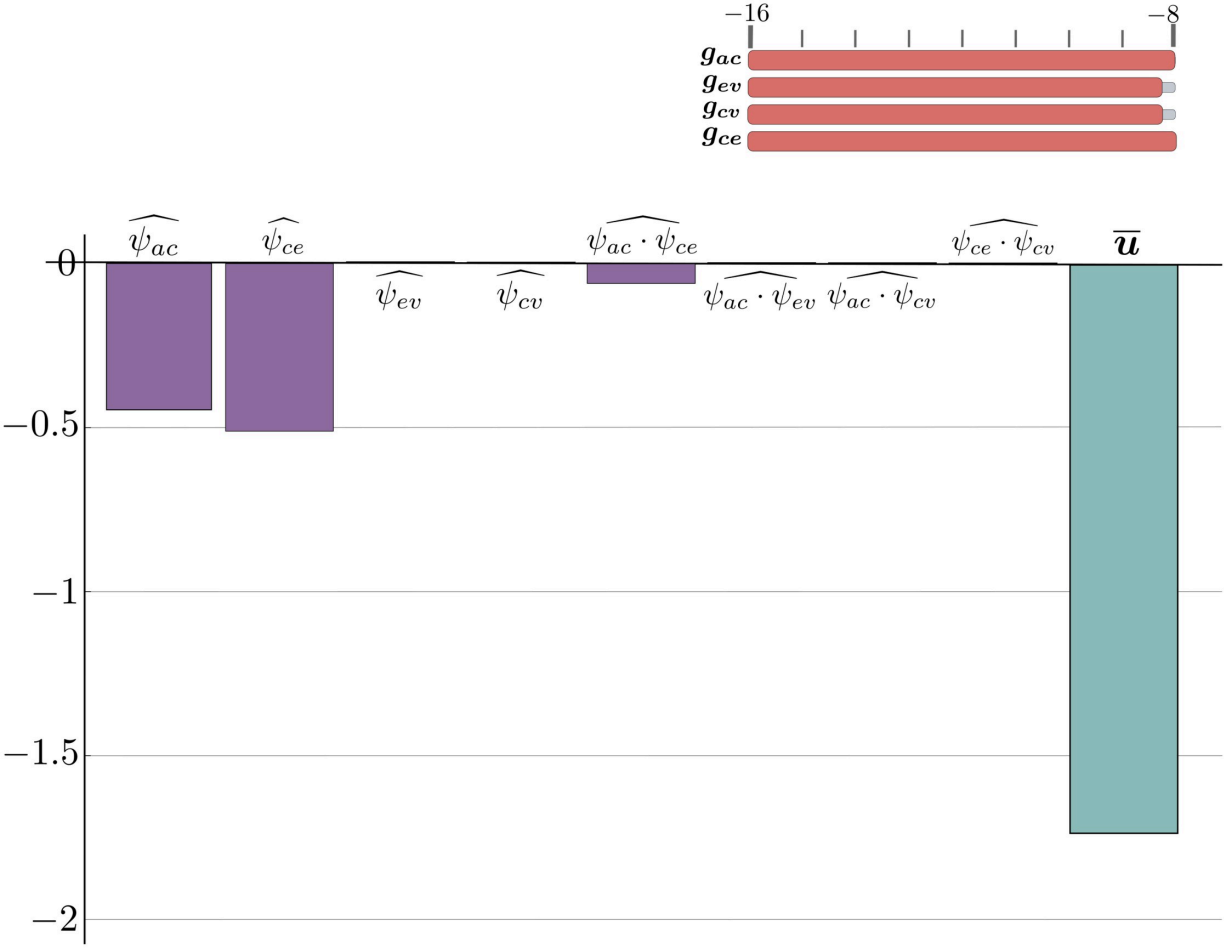
The regression terms for intracranial hypertension. Possible regression terms for intracranial hypertension.

When capillary-venous outflow is disrupted and there is arterial-capillary and arterial-CSF cross-flow, the model describes a situation with intracranial hypertension. In addition, CSF outflow persists with impaired outflow, resulting in edema in the brain parenchyma and increased external pressure on the ventricular wall. The ventricles are compressed under parenchymal pressure. It is worth noting that an increase in any of the parameters describing venous outflow leads to a decrease in ventricular compression, Fig 11.

**Pattern 3: replacement ventriculomegaly under a prolonged hypoperfusion**

**Fig 12:**
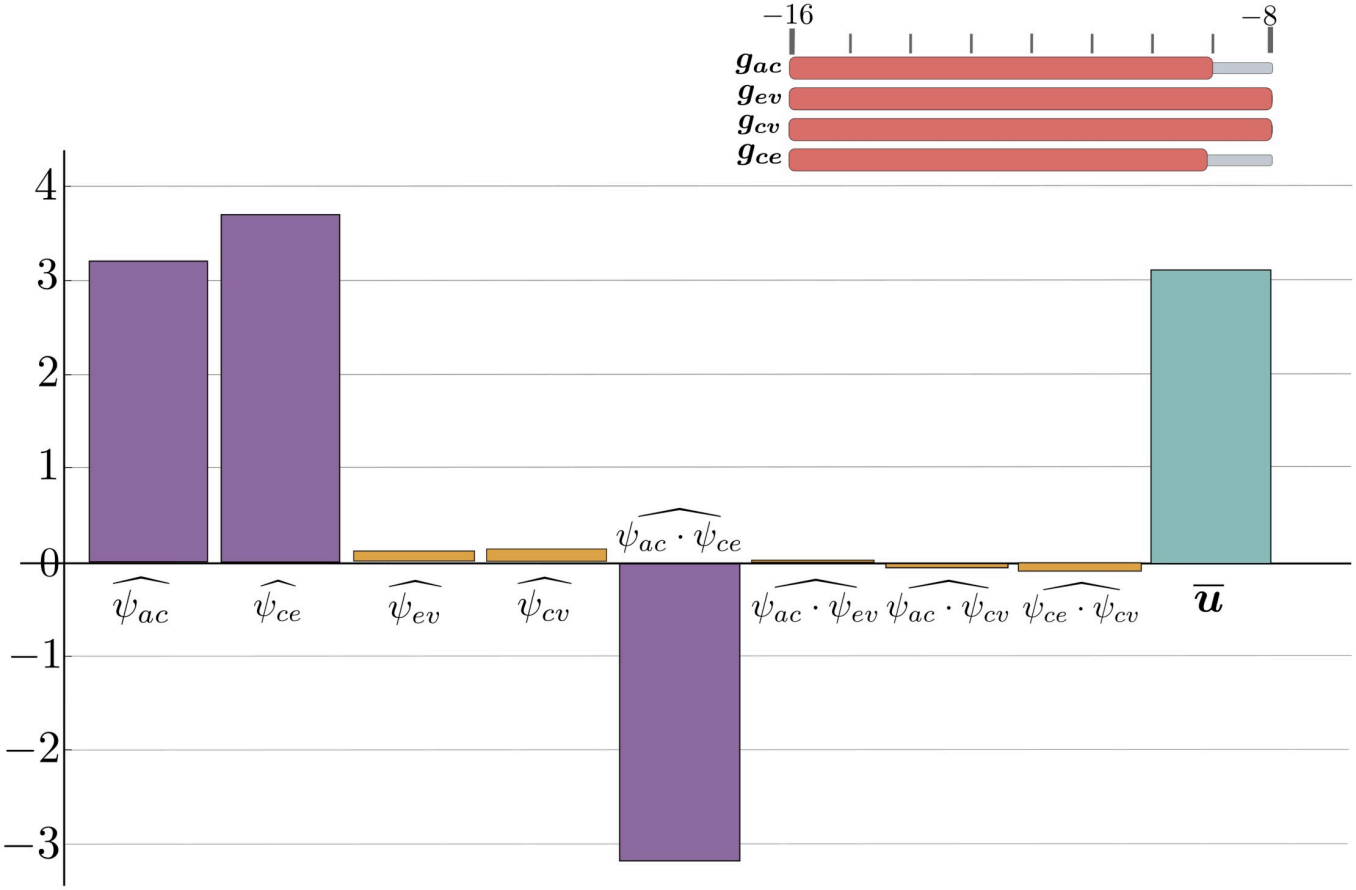
The regression terms for replacement ventriculomegaly on the background of prolonged hypoperfusion. Possible regression terms for replacement ventriculomegaly under a prolonged hypoperfusion.

When arterial-capillary inflow is impaired, the model describes chronic ischemic changes or brain tissue perfusion violation in which atrophic changes and ventricular replacement dilations may form. The decrease in capillary-CSF cross-flow exacerbates ventricular deformations, Fig 12.

## Discussion

The goal of this paper is to study the influence of the interaction of cerebral fluids on the mean displacement of ventricular wall and the pressure of the cerebral fluids. The analysis of numerical calculations for 50625 sets of interaction parameters has revealed the nature of this influence, which has a qualitative physiological justification. For a more detailed quantitative analysis of ventricular wall displacement, a multiple linear regression with a high coefficient of determination ($R_{adj.}^{2}>0.91$) was created for each of the volunteers. This regression accounts for the mutual influence of cross-flows between the cerebral fluids.

The regression describes the dependence of the average value for solving the partial differential equations Eqs (1)–(13) on the interaction parameters. The logarithmic dependence of mean ventricular wall displacement on the interaction parameters was revealed. A determining effect of the arterial-CSF component on the ventricular wall displacement was found.

The model describes the processes in a parenchyma. Since the brain tissue and microcirculatory vascular component prevail at the border with the outer wall of the ventricles, the predominant influence of the arterial-CSF component seems to be physiological. At the same time, the capillary-venous interaction parameter describes the cerebral drainage and its increase reduces the deformation of the ventricles.

The values of interaction parameters were found. The parameters can characterize such pathological states as normal pressure hydrocephalus, intracranial hypertension and replacement ventriculomegaly under a prolonged hypoperfusion, which seems clinically interesting.

Both the numerical analysis and the regression model demonstrate complex nonlinear dependence of the mean ventricular wall displacement on the interaction parameters. Different values of interaction parameters may correspond to both an increase and a decrease in ventricular size. Some aspects of this dependence cannot be easily interpreted physiologically at the moment. Namely the saddle-shaped surface of displacement $\bar{u}$ plots surface at small values of *g*_*ce*_ and *g*_*ac*_, as well as the presence of a term in the regression model responsible for the mutual influence of the arterial-capillary and CSF-venous components. These issues require further investigation.

Due to the difficulty in obtaining experimental data, validation of the numerical model is indeed a difficult task, since access to small vessel structures is not possible under in-vivo conditions. At the same time, efforts in this direction were made in the works on which this paper is based. In result was mainly show qualitative validation, with several limitations that need to be addressed [27, 46].

The natural evolution of the model may be directed to the transition to non-stationary equations, which, will allow us, in particular, to assess the process of hydrocephalus formation and the influence of interaction parameters on this process. Moreover, the transition from a flat geometry to a three-dimensional one is interesting. It is expected that in such a transition, the qualitative conclusions will not change despite the change in the values of the regression coefficients. An important stage of further work is to verify the model using data from a real patient with hydrocephalus.

## Conclusion

This paper describes the effect of the interaction of cerebral fluids (arterial, capillary and venous blood, cerebrospinal fluid) on ventricular wall displacement and periventricular pressure. For this purpose, we used a mathematical model of multiphase poroelasticity for the brain parenchyma similar to that in [27]. The interaction of cerebral fluids is given by a set of four numerical coefficients.

The effect of interaction parameters on mean ventricular wall displacement and periventricular pressure is described qualitatively. The authors constructed a multiple linear regression with interaction that allows us to quantify the effect of these coefficients on the mean ventricular wall displacement. A similar approach to this problem has not previously been found in the literature. Based on the regression model analysis the prevailing influence of the capillary-CSF component was found. The detailed analysis reveals the relationship between the interaction coefficients and the pathological conditions. In particular, sets of interaction parameters were found to be associated with normal pressure hydrocephalus, intracranial hypertension, and replacement ventriculomegaly under a prolonged hypoperfusion.

## Supporting information

S1 Appendix Weak formulation. Weak formulation corresponding to mathematical model.
(PDF)

S1 FileRegression results for volunteer 1.The file provides information about linear regression models for volunteer 1, which were constructed when searching for *b*_*opt*_. For each regression model, the regression formula, the value of *b*_*opt*_, $R_{adj.}^{2}$ and the value of the Akaike information criterion (*AIC*) are given.(PDF)

S2 FileRegression results for volunteer 2.The file provides information about linear regression models for volunteer 1, which were constructed when searching for *b*_*opt*_. For each regression model, the regression formula, the value of *b*_*opt*_, $R_{adj.}^{2}$ and the value of the Akaike information criterion (*AIC*) are given.(PDF)

S3 FileRegression results for volunteer 3.The file provides information about linear regression models for volunteer 1, which were constructed when searching for *b*_*opt*_. For each regression model, the regression formula, the value of *b*_*opt*_, $R_{adj.}^{2}$ and the value of the Akaike information criterion (*AIC*) are given.(PDF)

S4 FileRegression results for volunteer 4.The file provides information about linear regression models for volunteer 1, which were constructed when searching for *b*_*opt*_. For each regression model, the regression formula, the value of *b*_*opt*_, $R_{adj.}^{2}$ and the value of the Akaike information criterion (*AIC*) are given.(PDF)

S5 File Residual plots for regression model. The Residuals vs. Fitted values plot and Density vs. Residuals plot for all volunteers.
(PDF)

S6 FileNumerical results for all four volunteers.The file provides the numerical results: dependence of capillary pressure (*p*_*c*_) and mean wall ventricular displacement ($\bar{u}$) on the logarithm of the interaction parameters (*g*_*xy*_) for all four volunteers.(PDF)

## References

[1] Mori K. Current concept of hydrocephalus: evolution of new classifications. Child’s Nervous System. 1995;11(9):523–531. doi: 10.1007/BF00822842 8529219

[2] Rekate HL. A consensus on the classification of hydrocephalus: its utility in the assessment of abnormalities of cerebrospinal fluid dynamics. Child’s nervous system. 2011;27(10):1535–1541. doi: 10.1007/s00381-011-1558-y 21928019 PMC3175041

[3] Hakim CA, Hakim R, Hakim S. Normal-pressure hydrocephalus. Neurosurgery clinics of North America. 2001;12(4):761–73. doi: 10.1016/S1042-3680(18)30033-0 11524297

[4] Yankova G, Bogomyakova O, Tulupov A. The glymphatic system and meningeal lymphatics of the brain: new understanding of brain clearance. Reviews in the Neurosciences. 2021;. doi: 10.1515/revneuro-2020-0106 33618444

[5] Dalla Corte A, de Souza CF, Anés M, Maeda FK, Lokossou A, Vedolin LM, et al. Correlation of CSF flow using phase-contrast MRI with ventriculomegaly and CSF opening pressure in mucopolysaccharidoses. Fluids and Barriers of the CNS. 2017;14(1):1–12. 28918752 10.1186/s12987-017-0073-2 PMC5603164

[6] Marmarou A. A theoretical model and experimental evaluation of the cerebrospinal fluid system. Thesis Philadelphia: Drexel University. 1973.

[7] Rekate HL, Brodkey JA, Chizeck HJ, El Sakka W, Ko WH. Ventricular volume regulation: a mathematical model and computer simulation. Pediatric Neurosurgery. 1988;14(2):77–84. doi: 10.1159/000120367 3075040

[8] Linninger AA, Xenos M, Sweetman B, Ponkshe S, Guo X, Penn R. A mathematical model of blood, cerebrospinal fluid and brain dynamics. Journal of mathematical biology. 2009;59(6):729–759. doi: 10.1007/s00285-009-0250-2 19219605

[9] Kasprowicz M, Lalou DA, Czosnyka M, Garnett M, Czosnyka Z. Intracranial pressure, its components and cerebrospinal fluid pressure–volume compensation. Acta Neurologica Scandinavica. 2016;134(3):168–180. doi: 10.1111/ane.12541 26666840

[10] Buishas J, Gould IG, Linninger AA. A computational model of cerebrospinal fluid production and reabsorption driven by Starling forces. Croatian medical journal. 2014;55(5):481–497. doi: 10.3325/cmj.2014.55.481 25358881 PMC4228294

[11] Kim M, Cirovic S. A computational model of the cerebrospinal fluid system incorporating lumped-parameter cranial compartment and one-dimensional distributed spinal compartment. Journal of biorheology. 2011;25(1):78–87. doi: 10.1007/s12573-011-0041-4

[12] Elliott N, Lockerby DA, Brodbelt A. A lumped-parameter model of the cerebrospinal system for investigating arterial-driven flow in posttraumatic syringomyelia. Medical engineering & physics. 2011;33(7):874–882. doi: 10.1016/j.medengphy.2010.07.009 20833093

[13] Elliott N, Bertram C, Martin BA, Brodbelt A. Syringomyelia: a review of the biomechanics. Journal of Fluids and Structures. 2013;40:1–24. doi: 10.1016/j.jfluidstructs.2013.01.010

[14] Chang H, Nakagawa H. Hypothesis on the pathophysiology of syringomyelia based on simulation of cerebrospinal fluid dynamics. Journal of Neurology, Neurosurgery & Psychiatry. 2003;74(3):344–347. doi: 10.1136/jnnp.74.3.344 12588922 PMC1738338

[15] Toro EF, Celant M, Zhang Q, Contarino C, Agarwal N, Linninger A, et al. Cerebrospinal fluid dynamics coupled to the global circulation in holistic setting: mathematical models, numerical methods and applications. International Journal for Numerical Methods in Biomedical Engineering. 2021; p. e3532. 34569188 10.1002/cnm.3532 PMC9285081

[16] Clarke EC, Fletcher DF, Stoodley MA, Bilston LE. Computational fluid dynamics modelling of cerebrospinal fluid pressure in Chiari malformation and syringomyelia. Journal of biomechanics. 2013;46(11):1801–1809. doi: 10.1016/j.jbiomech.2013.05.013 23769174

[17] Lloyd RA, Fletcher DF, Clarke EC, Bilston LE. Chiari malformation may increase perivascular cerebrospinal fluid flow into the spinal cord: a subject-specific computational modelling study. Journal of biomechanics. 2017;65:185–193. doi: 10.1016/j.jbiomech.2017.10.007 29096983

[18] Linninger AA, Sweetman B, Penn R. Normal and hydrocephalic brain dynamics: the role of reduced cerebrospinal fluid reabsorption in ventricular enlargement. Annals of biomedical engineering. 2009;37(7):1434–1447. doi: 10.1007/s10439-009-9691-4 19373558

[19] Sweetman B, Linninger AA. Cerebrospinal fluid flow dynamics in the central nervous system. Annals of biomedical engineering. 2011;39(1):484–496. doi: 10.1007/s10439-010-0141-0 20737291

[20] Gholampour S. FSI simulation of CSF hydrodynamic changes in a large population of non-communicating hydrocephalus patients during treatment process with regard to their clinical symptoms. PLoS One. 2018;13(4):e0196216. doi: 10.1371/journal.pone.0196216 29708982 PMC5927404

[21] Hakim S, Venegas JG, Burton JD. The physics of the cranial cavity, hydrocephalus and normal pressure hydrocephalus: mechanical interpretation and mathematical model. Surgical neurology. 1976;5(3):187–210. 1257894

[22] Smillie A, Sobey I, Molnar Z. A hydroelastic model of hydrocephalus. Journal of Fluid Mechanics. 2005;539:417–443. doi: 10.1017/S0022112005005707

[23] Sivaloganathan S, Stastna M, Tenti G, Drake J. Biomechanics of the brain: a theoretical and numerical study of Biot’s equations of consolidation theory with deformation-dependent permeability. International Journal of Non-Linear Mechanics. 2005;40(9):1149–1159. doi: 10.1016/j.ijnonlinmec.2005.04.004

[24] Tully B, Ventikos Y. Coupling poroelasticity and CFD for cerebrospinal fluid hydrodynamics. IEEE Transactions on Biomedical Engineering. 2009;56(6):1644–1651. doi: 10.1109/TBME.2009.2016427 19304478

[25] Eisenträger A, Sobey I. Multi-fluid poroelastic modelling of CSF flow through the brain. In: Poromechanics V: Proceedings of the Fifth Biot Conference on Poromechanics; 2013. p. 2148–2157.

[26] Wirth B, Sobey I. Analytic solution during an infusion test of the linear unsteady poroelastic equations in a spherically symmetric model of the brain. Mathematical medicine and biology: a journal of the IMA. 2009;26(1):25–61. doi: 10.1093/imammb/dqn021 19050059

[27] Tully B, Ventikos Y. Cerebral water transport using multiple-network poroelastic theory: application to normal pressure hydrocephalus. Journal of Fluid Mechanics. 2011;667:188–215. doi: 10.1017/S0022112010004428

[28] Guo L, Vardakis JC, Lassila T, Mitolo M, Ravikumar N, Chou D, et al. Subject-specific multi-poroelastic model for exploring the risk factors associated with the early stages of Alzheimer’s disease. Interface focus. 2018;8(1):20170019. doi: 10.1098/rsfs.2017.0019 29285346 PMC5740222

[29] Vardakis JC, Chou D, Guo L, Ventikos Y. Exploring neurodegenerative disorders using a novel integrated model of cerebral transport: Initial results. Proceedings of the Institution of Mechanical Engineers, Part H: Journal of Engineering in Medicine. 2020;234(11):1223–1234. doi: 10.1177/0954411920964630 33078663 PMC7675777

[30] Yankova G, Cherevko A, Khe A, Bogomyakova O, Tulupov A. Study of Hydrocephalus Using Poroelastic Models. Journal of Applied Mechanics and Technical Physics. 2020;61(1):14–24. doi: 10.1134/S0021894420010022

[31] Masoumi N, Framanzad F, Zamanian B, Seddighi A, Moosavi M, Najarian S, et al. 2D Computational Fluid Dynamic Modeling of Human Ventricle System Based on Fluid-Solid Interaction and Pulsatile Flow. Basic and Clinical Neuroscience. 2013;4(1):64–75. 25337330 PMC4202551

[32] Agapov P, Belotserkovskii OM, Petrov IB. Numerical simulation of the consequences of a mechanical action on a human brain under a skull injury. Computational Mathematics and Mathematical Physics. 2006;46(9):1629–1638. doi: 10.1134/S0965542506090144

[33] Petrov IB. Solution of Deformable Solid Mechanics Dynamical Problems with Use of Mathematical Modeling by Grid-Characteristic Method. In: Continuum Mechanics, Applied Mathematics and Scientific Computing: Godunov’s Legacy. Springer; 2020. p. 299–305.

[34] Cheng S, Jacobson E, Bilston L. Models of the pulsatile hydrodynamics of cerebrospinal fluid flow in the normal and abnormal intracranial system. Computer methods in biomechanics and biomedical engineering. 2007;10(2):151–157. doi: 10.1080/10255840601124753 18651281

[35] Yankova G, Cherevko A, Khe A, Bogomyakova O, Tulupov A. Mathematical modeling of normal-pressure hydrocephalus at different levels of the brain geometry detalization. Journal of Applied Mechanics and Technical Physics. 2021;62(4):654–662. doi: 10.1134/S0021894421040155

[36] Sobey I, Wirth B. Effect of non-linear permeability in a spherically symmetric model of hydrocephalus. Mathematical Medicine and Biology. 2006;23(4):339–361. doi: 10.1093/imammb/dql015 16740628

[37] Vardakis J, Guo L, Peach T, Lassil T, Mitolo M, Chou D, et al. Fluid-structure interaction for highly complex, statistically defined, biological media: Homogenisation and a 3D multi-compartmental poroelastic model for brain biomechanics. Journal of Fluids and Structures. 2019;91:102641. doi: 10.1016/j.jfluidstructs.2019.04.008

[38] Coussy O. Poromechanics. John Wiley & Sons; 2004.

[39] Taylor Z, Miller K. Reassessment of brain elasticity for analysis of biomechanisms of hydrocephalus. Journal of Biomechanics. 2004;37(8):1263–1269. doi: 10.1016/j.jbiomech.2003.11.027 15212932

[40] Vardakis J, Tully B, V Y. Multicompartmental poroelasticity as a platform for the integrative modelling of water transport in the brain. Springer Science & Business Media; 2012.

[41] Hecht F. New development in FreeFem++. Journal of Numerical Mathematics. 2012;20:251–265. doi: 10.1515/jnum-2012-0013

[42] Johanson CE, Duncan JA, Klinge PM, Brinker T, Stopa EG, Silverberg GD. Multiplicity of cerebrospinal fluid functions: new challenges in health and disease. Cerebrospinal fluid research. 2008;5(1):1–32. doi: 10.1186/1743-8454-5-10 18479516 PMC2412840

[43] R Core Team. R: A Language and Environment for Statistical Computing; 2020. Available from: https://www.R-project.org/.

[44] Byrd RH, Lu P, Nocedal J, Zhu C. A limited memory algorithm for bound constrained optimization. SIAM Journal on scientific computing. 1995;16(5):1190–1208. doi: 10.1137/0916069

[45] Johnston I, Teo C. Disorders of CSF hydrodynamics. Child’s nervous system. 2000;16(1011):776–799. doi: 10.1007/s003810000383 11151732

[46] Guo L, Li Z, Lyu J, Mei Y, Vardakis JC, Chen D, et al. On the validation of a multiple-network poroelastic model using arterial spin labeling MRI data. Frontiers in computational neuroscience. 2019;13:60. doi: 10.3389/fncom.2019.00060 31551742 PMC6733888

