# Supplementary material for: Influence of interaction of cerebral fluids on ventricular deformation: A mathematical approach
Source: PLoS One. 2022 Feb 28;17(2):e0264395. doi: 10.1371/journal.pone.0264395 (PMC8884699; doi:10.1371/journal.pone.0264395)
Supplement: S1 Appendix — Weak formulation corresponding to mathematical model. (PDF) [file pone.0264395.s001.pdf]

Mathematical model

The equilibrium vector equation for brain parenchyma is [3]

$$\mu\Delta\boldsymbol{u} + (\mu+\lambda)\nabla(\operatorname{div}\boldsymbol{u}) - (\alpha_a\nabla p_a + \alpha_c\nabla p_c + \alpha_e\nabla p_e + \alpha_v\nabla p_v) = 0. \qquad (1)$$

Using the mass conservation law and Darcy's law for pore fluids we obtain equations for pressures

$$-\frac{k_a}{\mu_a}\Delta p_a - \gamma_{ac}(p_c - p_a) = 0, \qquad (2)$$

$$-\frac{k_v}{\mu_v}\Delta p_v + \gamma_{cv}(p_v - p_c) + \gamma_{ev}(p_v - p_e) = 0, \qquad (3)$$

$$-\frac{k_e}{\mu_e}\Delta p_e + \gamma_{ce}(p_e - p_c) - \gamma_{ev}(p_v - p_e) = 0, \qquad (4)$$

$$-\frac{k_c}{\mu_c}\Delta p_c + \gamma_{ac}(p_c - p_a) - \gamma_{ce}(p_e - p_c) - \gamma_{cv}(p_v - p_c) = 0; \qquad (5)$$

Here $\boldsymbol{u}$ — brain parenchyma displacement, λ and μ — elastic moduli, p_i — the i-th pore fluid pressure, α_i — Biot's coefficients, k_i — permeability coefficients, μ_i — pore fluid viscosity, $i = a, c, v, e$. The terms of the form $S_{yx} = \gamma_{yx}\,(p_x - p_y)$ describe the fluid transport from the x network to the y network due to the hydrostatic pressure gradient. Here γ_{yx} are parameters specifying interactions and flows of pore fluids between basins. Below, we will refer to γ_{yx} as interaction parameters.

Boundary conditions

The system of equations Eq (1) — Eq (5) is complemented by boundary conditions for displacement and four pore pressures. At the cerebral ventricular boundary Γ_V the following conditions are set.

1. Stresses are assumed to be continuous:

$$2\mu\varepsilon(\boldsymbol{u})\cdot\boldsymbol{n} + \lambda\epsilon(\boldsymbol{u})\boldsymbol{n} = \sum_{i=a,c,e,v}(\alpha_i - 1)\,p_i\boldsymbol{n} \qquad (6)$$

$\varepsilon(\boldsymbol{u})$ — strain tensor; $\epsilon(\boldsymbol{u}) = \operatorname{tr}\varepsilon(\boldsymbol{u}) = \varepsilon(\boldsymbol{u})_{ii} = \operatorname{div}\boldsymbol{u}$; $\boldsymbol{n}$ — external unit normal vector.

2. There is no flow for the arterial and venous networks:

$$\nabla p_a\boldsymbol{n} = \nabla p_v\boldsymbol{n} = 0; \qquad (7)$$

3. CSF is secreted at a constant rate Q_p in the brain ventricles. The conservation condition of fluid mass in the ventricular system takes into account the CSF volume produced by the vascular plexuses, the CSF volume that seeps through the ventricular wall, the CSF outflow through the Sylvian aqueduct

$$Q_p = \frac{\pi d^4}{128\mu L}\left(p_e|_{\Gamma_V} - p_e|_{\Gamma_S}\right) - \oint_{\Gamma_V}\left(-\frac{k_e}{\mu_e}\nabla p_e\right)\cdot \boldsymbol{n} dS \tag{8}$$

d, L — diameter and length of the Silvius aqueduct.

4. The CSF formation from blood leads to a drop in capillary network pressure:

$$\kappa_{cv}\nabla p_c \boldsymbol{n} = Q_p, \tag{9}$$

where κ_{cv} — flow resistance from the capillary network into the ventricles via the vascular plexus.

At the skull boundary Γ_S the following assumptions are accepted.

1. Since this paper considers the adult brain, the skull is considered rigid. Thus, the displacements of the skull boundary are equal to zero:

$$\boldsymbol{u} = 0. \tag{10}$$

2. No capillary flow at the skull boundary:

$$\nabla p_c \boldsymbol{n} = 0, \tag{11}$$

3. Arterial and venous pressures are set:

$$p_a = p_{art}, \;\; p_v = p_{ven}. \tag{12}$$

4. CSF absorption into the venous network leads to an increase in pressure:

$$p_e = p_v + \mu_e R Q_0, \tag{13}$$

where R — resistance due to the presence of arachnoid granulations; Q_0 — CSF outflow into the venous network, μ_e — CSF viscosity.

Weak formulation

To use the finite element method, it is necessary to write down a weak formulation of the problem Eq (1) –Eq (5). To do this, we multiply Eq (1) –Eq (5) by the test functions $\boldsymbol{\xi}$, ϕ_a, ϕ_v, ϕ_c, $\phi_e \in H^1(\Omega)$ respectively and integrate them over the domain Ω taking into account the boundary conditions

$$\boldsymbol{\xi}|_{\Gamma_S} = 0, \;\; \phi_a|_{\Gamma_S} = 0, \;\; \phi_v|_{\Gamma_S} = 0, \;\; \phi_e|_{\Gamma_S} = 0. \tag{14}$$

When using the Gauss formula and boundary conditions Eq (6) – Eq (13) the problem has the following formulation: find functions $\boldsymbol{u}$, p_a, p_v, p_c, $p_e \in H^1(\Omega)$, satisfying the boundary conditions Eq (6) – Eq (13) and integral relations

$$\begin{aligned}
&\int\limits_{\Omega}\left(\left(-\lambda\operatorname{div}\boldsymbol{u}+\sum_{i=a,c,e,v}\alpha_i p_i\right)\operatorname{div}\boldsymbol{\xi}-2\mu\varepsilon(\boldsymbol{u}):\varepsilon(\boldsymbol{\xi})\right)\,\mathrm{d}\Omega- \\
&\qquad\qquad\qquad\qquad -\int\limits_{\Gamma_V}\left(\sum_{i=a,c,e,v}p_i\boldsymbol{n}\cdot\boldsymbol{\xi}\right)\,\mathrm{d}s=0, \\
&\int\limits_{\Omega}\left(\frac{k_a}{\mu_a}\nabla p_a\cdot\nabla\phi_a-\gamma_{ac}(p_c-p_a)\phi_a\right)\,\mathrm{d}\Omega=0, \\
&\int\limits_{\Omega}\left(\frac{k_v}{\mu_v}\nabla p_v\cdot\nabla\phi_v+\left(\gamma_{cv}(p_v-p_c)+\gamma_{ev}(p_v-p_e)\right)\phi_v\right)\,\mathrm{d}\Omega=0, \\
&\int\limits_{\Omega}\left(\frac{k_c}{\mu_c}\nabla p_c\cdot\nabla\phi_c+\left(\gamma_{ac}(p_c-p_a)-\gamma_{ce}(p_e-p_c)-\gamma_{cv}(p_v-p_c)\right)\phi_c\right)\,\mathrm{d}\Omega- \\
&-\int\limits_{\Gamma_V}\frac{k_c}{\mu_c}\frac{Q_p}{\kappa_{cv}}\phi_c\,\mathrm{d}s=0, \\
&\int\limits_{\Omega}\left(\frac{k_e}{\mu_e}\nabla p_e\cdot\nabla\phi_e+\left(\gamma_{ce}(p_e-p_c)-\gamma_{ev}(p_v-p_e)\right)\phi_c\right)\,\mathrm{d}\Omega- \\
&\qquad -\int\limits_{\Gamma_V}\left(\frac{Q_p}{4\pi(r_1+u)^2}+\frac{\pi d^4}{4\pi(r_1+u)^2 128L\mu_e}\left(p_{ven}+\mu_e RQ_0-p_e\right)\right)\phi_e\,\mathrm{d}s=0,
\end{aligned} \tag{15}$$

for arbitrary test functions $\boldsymbol{\xi}$, ϕ_a, ϕ_v, ϕ_c, ϕ_e , satisfying the boundary conditions Eq (15). the denominator contains a multiplier of $4\pi(r_1+u)^2$, which is the spherical approximation of the ventricular surface for the second (integral) term in Eq (8). Where $r_1 = 0.03\ m$ corresponds to the average value of the brain ventricle radius [1, 2].

To numerically solve the equations Eq (1)-Eq (5) with boundary conditions Eq (6) – Eq (13) the finite element method was used. The calculations were performed in the open package FreeFem++ [4].

References

[1] Tully B, Ventikos Y. Coupling poroelasticity and CFD for cerebrospinal fluid hydrodynamics. IEEE Transactions on Biomedical Engineering. 2009;56(6):1644–1651.

[2] Wirth B, Sobey I. Analytic solution during an infusion test of the linear unsteady poroelastic equations in a spherically symmetric model of the brain. Mathematical medicine and biology: a journal of the IMA. 2009;26(1):25–61.

[3] Coussy O. Poromechanics. John Wiley & Sons; 2004.

[4] Hecht F. New development in FreeFem++. Journal of Numerical Mathematics. 2012;20:251–265.
