# Supplementary material for: Influence of interaction of cerebral fluids on ventricular deformation: A mathematical approach
Source: PLoS One. 2022 Feb 28;17(2):e0264395. doi: 10.1371/journal.pone.0264395 (PMC8884699; doi:10.1371/journal.pone.0264395)
Supplement: S1 File — The file provides information about linear regression models for volunteer 1, which were constructed when searching for bopt. For each regression model, the regression formula, the value of bopt, Radj.2 and the value of the Akaike information criterion (AIC) are given. (PDF) [file pone.0264395.s002.pdf]

Table 1: Regression results for volunteer 1

N	Formula	Optimal b	$R^2_{adj.}$	AIC
0	$\overline{u} \sim \psi_{ac}+\psi_{ce}+\psi_{ev}+\psi_{cv}+\psi_{ac}\cdot\psi_{ce}+\psi_{ac}\cdot\psi_{ev}+\psi_{ac}\cdot\psi_{cv}+\psi_{ce}\cdot\psi_{ev}+\psi_{ce}\cdot\psi_{cv}+\psi_{ev}\cdot\psi_{cv}$	0.169808	0.93155	5562.31
1	$\overline{u} \sim \psi_{ac}+\psi_{ce}+\psi_{ev}+\psi_{cv}+\psi_{ac}\cdot\psi_{ce}+\psi_{ac}\cdot\psi_{ev}+\psi_{ac}\cdot\psi_{cv}+\psi_{ce}\cdot\psi_{cv}+\psi_{ev}\cdot\psi_{cv}$	0.169632	0.93154	5566.78
2	$\overline{u} \sim \psi_{ac}+\psi_{ce}+\psi_{ev}+\psi_{cv}+\psi_{ac}\cdot\psi_{ce}+\psi_{ac}\cdot\psi_{ev}+\psi_{ac}\cdot\psi_{cv}+\psi_{ce}\cdot\psi_{ev}+\psi_{ce}\cdot\psi_{cv}$	0.169214	0.93121	5712.19
3	$\overline{u} \sim \psi_{ac}+\psi_{ce}+\psi_{ev}+\psi_{cv}+\psi_{ac}\cdot\psi_{ce}+\psi_{ac}\cdot\psi_{ev}+\psi_{ac}\cdot\psi_{cv}+\psi_{ce}\cdot\psi_{cv}$	0.16896	0.93118	5723.46
4	$\overline{u} \sim \psi_{ac}+\psi_{ce}+\psi_{ev}+\psi_{cv}+\psi_{ac}\cdot\psi_{ce}+\psi_{ac}\cdot\psi_{cv}+\psi_{ce}\cdot\psi_{ev}+\psi_{ce}\cdot\psi_{cv}+\psi_{ev}\cdot\psi_{cv}$	0.16627	0.93017	6163.8
5	$\overline{u} \sim \psi_{ac}+\psi_{ce}+\psi_{ev}+\psi_{cv}+\psi_{ac}\cdot\psi_{ce}+\psi_{ac}\cdot\psi_{cv}+\psi_{ce}\cdot\psi_{ev}+\psi_{ce}\cdot\psi_{cv}$	0.166281	0.93015	6172.06
6	$\overline{u} \sim \psi_{ac}+\psi_{ce}+\psi_{ev}+\psi_{cv}+\psi_{ac}\cdot\psi_{ce}+\psi_{ac}\cdot\psi_{cv}+\psi_{ce}\cdot\psi_{cv}+\psi_{ev}\cdot\psi_{cv}$	0.165769	0.93009	6199.97
7	$\overline{u} \sim \psi_{ac}+\psi_{ce}+\psi_{ev}+\psi_{cv}+\psi_{ac}\cdot\psi_{ce}+\psi_{ac}\cdot\psi_{cv}+\psi_{ce}\cdot\psi_{cv}$	0.165773	0.93006	6209.71
8	$\overline{u} \sim \psi_{ac}+\psi_{ce}+\psi_{ev}+\psi_{cv}+\psi_{ac}\cdot\psi_{ce}+\psi_{ac}\cdot\psi_{ev}+\psi_{ce}\cdot\psi_{ev}+\psi_{ce}\cdot\psi_{cv}+\psi_{ev}\cdot\psi_{cv}$	0.133822	0.92241	9355.36
9	$\overline{u} \sim \psi_{ac}+\psi_{ce}+\psi_{ev}+\psi_{cv}+\psi_{ac}\cdot\psi_{ce}+\psi_{ac}\cdot\psi_{ev}+\psi_{ce}\cdot\psi_{cv}+\psi_{ev}\cdot\psi_{cv}$	0.133723	0.9224	9357.23
10	$\overline{u} \sim \psi_{ac}+\psi_{ce}+\psi_{ev}+\psi_{cv}+\psi_{ac}\cdot\psi_{ce}+\psi_{ac}\cdot\psi_{ev}+\psi_{ce}\cdot\psi_{ev}+\psi_{ce}\cdot\psi_{cv}$	0.13343	0.9221	9472.47
11	$\overline{u} \sim \psi_{ac}+\psi_{ce}+\psi_{ev}+\psi_{cv}+\psi_{ac}\cdot\psi_{ce}+\psi_{ac}\cdot\psi_{ev}+\psi_{ce}\cdot\psi_{cv}$	0.133278	0.92208	9479.27
12	$\overline{u} \sim \psi_{ac}+\psi_{ce}+\psi_{ev}+\psi_{cv}+\psi_{ac}\cdot\psi_{ce}+\psi_{ce}\cdot\psi_{ev}+\psi_{ce}\cdot\psi_{cv}+\psi_{ev}\cdot\psi_{cv}$	0.131249	0.92112	9850.65
13	$\overline{u} \sim \psi_{ac}+\psi_{ce}+\psi_{ev}+\psi_{cv}+\psi_{ac}\cdot\psi_{ce}+\psi_{ce}\cdot\psi_{ev}+\psi_{ce}\cdot\psi_{cv}$	0.131266	0.92111	9855.4
14	$\overline{u} \sim \psi_{ac}+\psi_{ce}+\psi_{ev}+\psi_{cv}+\psi_{ac}\cdot\psi_{ce}+\psi_{ce}\cdot\psi_{cv}+\psi_{ev}\cdot\psi_{cv}$	0.130919	0.92105	9877.08
15	$\overline{u} \sim \psi_{ac}+\psi_{ce}+\psi_{ev}+\psi_{cv}+\psi_{ac}\cdot\psi_{ce}+\psi_{ce}\cdot\psi_{cv}$	0.130932	0.92103	9882.92
16	$\overline{u} \sim \psi_{ac}+\psi_{ce}+\psi_{cv}+\psi_{ac}\cdot\psi_{ce}+\psi_{ac}\cdot\psi_{ev}+\psi_{ac}\cdot\psi_{cv}+\psi_{ce}\cdot\psi_{ev}+\psi_{ce}\cdot\psi_{cv}+\psi_{ev}\cdot\psi_{cv}$	0.156888	0.91883	10717.2
17	$\overline{u} \sim \psi_{ac}+\psi_{ce}+\psi_{cv}+\psi_{ac}\cdot\psi_{ce}+\psi_{ac}\cdot\psi_{ev}+\psi_{ac}\cdot\psi_{cv}+\psi_{ce}\cdot\psi_{cv}+\psi_{ev}\cdot\psi_{cv}$	0.158277	0.91805	11007.7
18	$\overline{u} \sim \psi_{ac}+\psi_{ce}+\psi_{cv}+\psi_{ac}\cdot\psi_{ce}+\psi_{ac}\cdot\psi_{ev}+\psi_{ac}\cdot\psi_{cv}+\psi_{ce}\cdot\psi_{ev}+\psi_{ce}\cdot\psi_{cv}$	0.157235	0.91774	11123.2
19	$\overline{u} \sim \psi_{ac}+\psi_{ce}+\psi_{cv}+\psi_{ac}\cdot\psi_{ce}+\psi_{ac}\cdot\psi_{ev}+\psi_{ac}\cdot\psi_{cv}+\psi_{ce}\cdot\psi_{cv}$	0.158563	0.91705	11372.6
20	$\overline{u} \sim \psi_{ac}+\psi_{ce}+\psi_{cv}+\psi_{ac}\cdot\psi_{ce}+\psi_{ac}\cdot\psi_{cv}+\psi_{ce}\cdot\psi_{ev}+\psi_{ce}\cdot\psi_{cv}+\psi_{ev}\cdot\psi_{cv}$	0.147977	0.91214	13115.5
21	$\overline{u} \sim \psi_{ac}+\psi_{ce}+\psi_{ev}+\psi_{ac}\cdot\psi_{ce}+\psi_{ac}\cdot\psi_{ev}+\psi_{ac}\cdot\psi_{cv}+\psi_{ce}\cdot\psi_{ev}+\psi_{ce}\cdot\psi_{cv}+\psi_{ev}\cdot\psi_{cv}$	0.13089	0.91122	13432.2
22	$\overline{u} \sim \psi_{ac}+\psi_{ce}+\psi_{ev}+\psi_{ac}\cdot\psi_{ce}+\psi_{ac}\cdot\psi_{ev}+\psi_{ac}\cdot\psi_{cv}+\psi_{ce}\cdot\psi_{cv}+\psi_{ev}\cdot\psi_{cv}$	0.130983	0.91121	13434.1
23	$\overline{u} \sim \psi_{ac}+\psi_{ce}+\psi_{ev}+\psi_{ac}\cdot\psi_{ce}+\psi_{ac}\cdot\psi_{ev}+\psi_{ce}\cdot\psi_{ev}+\psi_{ce}\cdot\psi_{cv}+\psi_{ev}\cdot\psi_{cv}$	0.135948	0.91047	13683.3

24	$\overline{\boldsymbol{u}} \sim \psi_{ac}+\psi_{ce}+\psi_{ev}+\psi_{ac}\cdot\psi_{ce}+\psi_{ac}\cdot\psi_{ev}+\psi_{ce}\cdot\psi_{cv}+\psi_{ev}\cdot\psi_{cv}$	0.136141	0.91045	13689.2
25	$\overline{\boldsymbol{u}} \sim \psi_{ac}+\psi_{ce}+\psi_{cv}+\psi_{ac}\cdot\psi_{ce}+\psi_{ac}\cdot\psi_{ev}+\psi_{ce}\cdot\psi_{ev}+\psi_{ce}\cdot\psi_{cv}+\psi_{ev}\cdot\psi_{cv}$	0.1265	0.90994	13862.9
26	$\overline{\boldsymbol{u}} \sim \psi_{ac}+\psi_{ce}+\psi_{cv}+\psi_{ac}\cdot\psi_{ce}+\psi_{ac}\cdot\psi_{ev}+\psi_{ce}\cdot\psi_{cv}+\psi_{ev}\cdot\psi_{cv}$	0.127613	0.90909	14146.8
27	$\overline{\boldsymbol{u}} \sim \psi_{ac}+\psi_{ce}+\psi_{cv}+\psi_{ac}\cdot\psi_{ce}+\psi_{ac}\cdot\psi_{ev}+\psi_{ce}\cdot\psi_{ev}+\psi_{ce}\cdot\psi_{cv}$	0.126711	0.90885	14226.3
28	$\overline{\boldsymbol{u}} \sim \psi_{ac}+\psi_{ce}+\psi_{cv}+\psi_{ac}\cdot\psi_{ce}+\psi_{ac}\cdot\psi_{ev}+\psi_{ce}\cdot\psi_{cv}$	0.12778	0.9081	14472.5
29	$\overline{\boldsymbol{u}} \sim \psi_{ac}+\psi_{ce}+\psi_{ev}+\psi_{ac}\cdot\psi_{ce}+\psi_{ac}\cdot\psi_{cv}+\psi_{ce}\cdot\psi_{ev}+\psi_{ce}\cdot\psi_{cv}+\psi_{ev}\cdot\psi_{cv}$	0.124621	0.90743	14694.2
30	$\overline{\boldsymbol{u}} \sim \psi_{ac}+\psi_{ce}+\psi_{ev}+\psi_{ac}\cdot\psi_{ce}+\psi_{ac}\cdot\psi_{cv}+\psi_{ce}\cdot\psi_{cv}+\psi_{ev}\cdot\psi_{cv}$	0.124425	0.9074	14702.1
31	$\overline{\boldsymbol{u}} \sim \psi_{ac}+\psi_{ce}+\psi_{ev}+\psi_{cv}+\psi_{ac}\cdot\psi_{ce}+\psi_{ac}\cdot\psi_{ev}+\psi_{ac}\cdot\psi_{cv}+\psi_{ce}\cdot\psi_{ev}+\psi_{ev}\cdot\psi_{cv}$	0.191084	0.90651	14996.2
32	$\overline{\boldsymbol{u}} \sim \psi_{ac}+\psi_{ce}+\psi_{ev}+\psi_{cv}+\psi_{ac}\cdot\psi_{ce}+\psi_{ac}\cdot\psi_{ev}+\psi_{ac}\cdot\psi_{cv}+\psi_{ev}\cdot\psi_{cv}$	0.190931	0.9065	14997.4
33	$\overline{\boldsymbol{u}} \sim \psi_{ac}+\psi_{ce}+\psi_{ev}+\psi_{cv}+\psi_{ac}\cdot\psi_{ce}+\psi_{ac}\cdot\psi_{ev}+\psi_{ac}\cdot\psi_{cv}+\psi_{ce}\cdot\psi_{ev}$	0.190507	0.9062	15093.2
34	$\overline{\boldsymbol{u}} \sim \psi_{ac}+\psi_{ce}+\psi_{ev}+\psi_{cv}+\psi_{ac}\cdot\psi_{ce}+\psi_{ac}\cdot\psi_{ev}+\psi_{ac}\cdot\psi_{cv}$	0.190276	0.90618	15098.4
35	$\overline{\boldsymbol{u}} \sim \psi_{ac}+\psi_{ce}+\psi_{ev}+\psi_{ac}\cdot\psi_{ce}+\psi_{ce}\cdot\psi_{ev}+\psi_{ce}\cdot\psi_{cv}+\psi_{ev}\cdot\psi_{cv}$	0.131411	0.9056	15285.6
36	$\overline{\boldsymbol{u}} \sim \psi_{ac}+\psi_{ce}+\psi_{ev}+\psi_{ac}\cdot\psi_{ce}+\psi_{ce}\cdot\psi_{cv}+\psi_{ev}\cdot\psi_{cv}$	0.131218	0.90558	15290.5
37	$\overline{\boldsymbol{u}} \sim \psi_{ac}+\psi_{ce}+\psi_{ev}+\psi_{cv}+\psi_{ac}\cdot\psi_{ce}+\psi_{ac}\cdot\psi_{cv}+\psi_{ce}\cdot\psi_{ev}+\psi_{ev}\cdot\psi_{cv}$	0.187451	0.90524	15401.5
38	$\overline{\boldsymbol{u}} \sim \psi_{ac}+\psi_{ce}+\psi_{ev}+\psi_{cv}+\psi_{ac}\cdot\psi_{ce}+\psi_{ac}\cdot\psi_{cv}+\psi_{ce}\cdot\psi_{ev}$	0.187467	0.90523	15405.7
39	$\overline{\boldsymbol{u}} \sim \psi_{ac}+\psi_{ce}+\psi_{ev}+\psi_{cv}+\psi_{ac}\cdot\psi_{ce}+\psi_{ac}\cdot\psi_{cv}+\psi_{ev}\cdot\psi_{cv}$	0.186975	0.90517	15422.3
40	$\overline{\boldsymbol{u}} \sim \psi_{ac}+\psi_{ce}+\psi_{ev}+\psi_{cv}+\psi_{ac}\cdot\psi_{ce}+\psi_{ac}\cdot\psi_{cv}$	0.186985	0.90515	15427.3
41	$\overline{\boldsymbol{u}} \sim \psi_{ac}+\psi_{ce}+\psi_{cv}+\psi_{ac}\cdot\psi_{ce}+\psi_{ce}\cdot\psi_{ev}+\psi_{ce}\cdot\psi_{cv}+\psi_{ev}\cdot\psi_{cv}$	0.121982	0.90354	15939.6
42	$\overline{\boldsymbol{u}} \sim \psi_{ac}+\psi_{ce}+\psi_{cv}+\psi_{ac}\cdot\psi_{ce}+\psi_{ac}\cdot\psi_{cv}+\psi_{ce}\cdot\psi_{cv}+\psi_{ev}\cdot\psi_{cv}$	0.14654	0.90262	16226.7
43	$\overline{\boldsymbol{u}} \sim \psi_{ac}+\psi_{ce}+\psi_{ev}+\psi_{ac}\cdot\psi_{ce}+\psi_{ac}\cdot\psi_{ev}+\psi_{ac}\cdot\psi_{cv}+\psi_{ce}\cdot\psi_{ev}+\psi_{ce}\cdot\psi_{cv}$	0.119697	0.90216	16369.7
44	$\overline{\boldsymbol{u}} \sim \psi_{ac}+\psi_{ce}+\psi_{ev}+\psi_{ac}\cdot\psi_{ce}+\psi_{ac}\cdot\psi_{ev}+\psi_{ac}\cdot\psi_{cv}+\psi_{ce}\cdot\psi_{cv}$	0.119587	0.90215	16372.7
45	$\overline{\boldsymbol{u}} \sim \psi_{ac}+\psi_{ce}+\psi_{ev}+\psi_{ac}\cdot\psi_{ce}+\psi_{ac}\cdot\psi_{cv}+\psi_{ce}\cdot\psi_{ev}+\psi_{ce}\cdot\psi_{cv}$	0.117811	0.90123	16655
46	$\overline{\boldsymbol{u}} \sim \psi_{ac}+\psi_{ce}+\psi_{ev}+\psi_{ac}\cdot\psi_{ce}+\psi_{ac}\cdot\psi_{cv}+\psi_{ce}\cdot\psi_{cv}$	0.117549	0.90117	16672.2
47	$\overline{\boldsymbol{u}} \sim \psi_{ac}+\psi_{ce}+\psi_{ac}\cdot\psi_{ce}+\psi_{ac}\cdot\psi_{ev}+\psi_{ac}\cdot\psi_{cv}+\psi_{ce}\cdot\psi_{ev}+\psi_{ce}\cdot\psi_{cv}+\psi_{ev}\cdot\psi_{cv}$	0.123709	0.9008	16787.9
48	$\overline{\boldsymbol{u}} \sim \psi_{ac}+\psi_{ce}+\psi_{ac}\cdot\psi_{ce}+\psi_{ac}\cdot\psi_{ev}+\psi_{ce}\cdot\psi_{ev}+\psi_{ce}\cdot\psi_{cv}+\psi_{ev}\cdot\psi_{cv}$	0.126651	0.90043	16898.9
49	$\overline{\boldsymbol{u}} \sim \psi_{ac}+\psi_{ce}+\psi_{ac}\cdot\psi_{ce}+\psi_{ac}\cdot\psi_{ev}+\psi_{ac}\cdot\psi_{cv}+\psi_{ce}\cdot\psi_{cv}+\psi_{ev}\cdot\psi_{cv}$	0.124705	0.89971	17117.4
50	$\overline{\boldsymbol{u}} \sim \psi_{ac}+\psi_{ce}+\psi_{ac}\cdot\psi_{ce}+\psi_{ce}\cdot\psi_{ev}+\psi_{ce}\cdot\psi_{cv}+\psi_{ev}\cdot\psi_{cv}$	0.124626	0.89965	17135

51	$\overline{\boldsymbol{u}} \sim \psi_{ac}+\psi_{ce}+\psi_{ac}\cdot\psi_{ce}+\psi_{ac}\cdot\psi_{cv}+\psi_{ce}\cdot\psi_{ev}+\psi_{ce}\cdot\psi_{cv}+\psi_{ev}\cdot\psi_{cv}$	0.124125	0.89965	17134.2
52	$\overline{\boldsymbol{u}} \sim \psi_{ac}+\psi_{ce}+\psi_{ac}\cdot\psi_{ce}+\psi_{ac}\cdot\psi_{ev}+\psi_{ce}\cdot\psi_{cv}+\psi_{ev}\cdot\psi_{cv}$	0.127818	0.8993	17238.6
53	$\overline{\boldsymbol{u}} \sim \psi_{ac}+\psi_{ce}+\psi_{ac}\cdot\psi_{ce}+\psi_{ac}\cdot\psi_{cv}+\psi_{ce}\cdot\psi_{cv}+\psi_{ev}\cdot\psi_{cv}$	0.127492	0.8946	18620.6
54	$\overline{\boldsymbol{u}} \sim \psi_{ac}+\psi_{ce}+\psi_{cv}+\psi_{ac}\cdot\psi_{ce}+\psi_{ac}\cdot\psi_{ev}+\psi_{ac}\cdot\psi_{cv}+\psi_{ce}\cdot\psi_{ev}+\psi_{ev}\cdot\psi_{cv}$	0.174976	0.89404	18781.8
55	$\overline{\boldsymbol{u}} \sim \psi_{ac}+\psi_{ce}+\psi_{cv}+\psi_{ac}\cdot\psi_{ce}+\psi_{ce}\cdot\psi_{cv}+\psi_{ev}\cdot\psi_{cv}$	0.122186	0.894	18791.4
56	$\overline{\boldsymbol{u}} \sim \psi_{ac}+\psi_{ce}+\psi_{ac}\cdot\psi_{ce}+\psi_{ce}\cdot\psi_{cv}+\psi_{ev}\cdot\psi_{cv}$	0.122931	0.89371	18874.4
57	$\overline{\boldsymbol{u}} \sim \psi_{ac}+\psi_{ce}+\psi_{cv}+\psi_{ac}\cdot\psi_{ce}+\psi_{ac}\cdot\psi_{ev}+\psi_{ac}\cdot\psi_{cv}+\psi_{ev}\cdot\psi_{cv}$	0.176339	0.89326	19003.1
58	$\overline{\boldsymbol{u}} \sim \psi_{ac}+\psi_{ce}+\psi_{ev}+\psi_{ac}\cdot\psi_{ce}+\psi_{ac}\cdot\psi_{ev}+\psi_{ac}\cdot\psi_{cv}+\psi_{ce}\cdot\psi_{ev}+\psi_{ev}\cdot\psi_{cv}$	0.153794	0.89307	19059.5
59	$\overline{\boldsymbol{u}} \sim \psi_{ac}+\psi_{ce}+\psi_{ev}+\psi_{ac}\cdot\psi_{ce}+\psi_{ac}\cdot\psi_{ev}+\psi_{ac}\cdot\psi_{cv}+\psi_{ev}\cdot\psi_{cv}$	0.153868	0.89306	19059.2
60	$\overline{\boldsymbol{u}} \sim \psi_{ac}+\psi_{ce}+\psi_{cv}+\psi_{ac}\cdot\psi_{ce}+\psi_{ac}\cdot\psi_{ev}+\psi_{ac}\cdot\psi_{cv}+\psi_{ce}\cdot\psi_{ev}$	0.17538	0.89306	19060.8
61	$\overline{\boldsymbol{u}} \sim \psi_{ac}+\psi_{ce}+\psi_{cv}+\psi_{ac}\cdot\psi_{ce}+\psi_{ac}\cdot\psi_{ev}+\psi_{ac}\cdot\psi_{cv}$	0.176685	0.89237	19252.7
62	$\overline{\boldsymbol{u}} \sim \psi_{ac}+\psi_{ce}+\psi_{ev}+\psi_{ac}\cdot\psi_{ce}+\psi_{ac}\cdot\psi_{cv}+\psi_{ce}\cdot\psi_{ev}+\psi_{ev}\cdot\psi_{cv}$	0.147001	0.8899	19942.5
63	$\overline{\boldsymbol{u}} \sim \psi_{ac}+\psi_{ce}+\psi_{ev}+\psi_{ac}\cdot\psi_{ce}+\psi_{ac}\cdot\psi_{cv}+\psi_{ev}\cdot\psi_{cv}$	0.146781	0.88987	19949
64	$\overline{\boldsymbol{u}} \sim \psi_{ac}+\psi_{ce}+\psi_{ev}+\psi_{cv}+\psi_{ac}\cdot\psi_{ce}+\psi_{ac}\cdot\psi_{ev}+\psi_{ev}\cdot\psi_{cv}$	0.148438	0.88957	20032
65	$\overline{\boldsymbol{u}} \sim \psi_{ac}+\psi_{ce}+\psi_{ev}+\psi_{cv}+\psi_{ac}\cdot\psi_{ce}+\psi_{ac}\cdot\psi_{ev}+\psi_{ce}\cdot\psi_{ev}+\psi_{ev}\cdot\psi_{cv}$	0.148518	0.88957	20032.4
66	$\overline{\boldsymbol{u}} \sim \psi_{ac}+\psi_{ce}+\psi_{ev}+\psi_{ac}\cdot\psi_{ce}+\psi_{ac}\cdot\psi_{ev}+\psi_{ev}\cdot\psi_{cv}$	0.148104	0.8895	20051.2
67	$\overline{\boldsymbol{u}} \sim \psi_{ac}+\psi_{ce}+\psi_{ev}+\psi_{ac}\cdot\psi_{ce}+\psi_{ac}\cdot\psi_{ev}+\psi_{ce}\cdot\psi_{ev}+\psi_{ev}\cdot\psi_{cv}$	0.148155	0.88949	20052.5
68	$\overline{\boldsymbol{u}} \sim \psi_{ac}+\psi_{ce}+\psi_{ev}+\psi_{cv}+\psi_{ac}\cdot\psi_{ce}+\psi_{ac}\cdot\psi_{ev}+\psi_{ce}\cdot\psi_{ev}$	0.148134	0.88932	20101.3
69	$\overline{\boldsymbol{u}} \sim \psi_{ac}+\psi_{ce}+\psi_{ev}+\psi_{cv}+\psi_{ac}\cdot\psi_{ce}+\psi_{ac}\cdot\psi_{ev}$	0.148002	0.8893	20103.5
70	$\overline{\boldsymbol{u}} \sim \psi_{ac}+\psi_{ce}+\psi_{ac}\cdot\psi_{ce}+\psi_{ac}\cdot\psi_{ev}+\psi_{ac}\cdot\psi_{cv}+\psi_{ce}\cdot\psi_{ev}+\psi_{ce}\cdot\psi_{cv}$	0.114458	0.88914	20148.9
71	$\overline{\boldsymbol{u}} \sim \psi_{ac}+\psi_{ce}+\psi_{cv}+\psi_{ac}\cdot\psi_{ce}+\psi_{ac}\cdot\psi_{cv}+\psi_{ce}\cdot\psi_{ev}+\psi_{ce}\cdot\psi_{cv}$	0.134789	0.88896	20197.9
72	$\overline{\boldsymbol{u}} \sim \psi_{ac}+\psi_{ce}+\psi_{ev}+\psi_{cv}+\psi_{ac}\cdot\psi_{ce}+\psi_{ce}\cdot\psi_{ev}+\psi_{ev}\cdot\psi_{cv}$	0.14582	0.8884	20350.2
73	$\overline{\boldsymbol{u}} \sim \psi_{ac}+\psi_{ce}+\psi_{ev}+\psi_{cv}+\psi_{ac}\cdot\psi_{ce}+\psi_{ce}\cdot\psi_{ev}$	0.145837	0.88839	20351.3
74	$\overline{\boldsymbol{u}} \sim \psi_{ac}+\psi_{ce}+\psi_{ev}+\psi_{cv}+\psi_{ac}\cdot\psi_{ce}+\psi_{ev}\cdot\psi_{cv}$	0.145509	0.88835	20363.9
75	$\overline{\boldsymbol{u}} \sim \psi_{ac}+\psi_{ce}+\psi_{ac}\cdot\psi_{ce}+\psi_{ac}\cdot\psi_{ev}+\psi_{ac}\cdot\psi_{cv}+\psi_{ce}\cdot\psi_{cv}$	0.115458	0.88835	20363.2
76	$\overline{\boldsymbol{u}} \sim \psi_{ac}+\psi_{ce}+\psi_{ev}+\psi_{cv}+\psi_{ac}\cdot\psi_{ce}$	0.145523	0.88834	20365.5
77	$\overline{\boldsymbol{u}} \sim \psi_{ac}+\psi_{ce}+\psi_{ev}+\psi_{ac}\cdot\psi_{ce}+\psi_{ce}\cdot\psi_{ev}+\psi_{ev}\cdot\psi_{cv}$	0.144313	0.88791	20483.2

78	$\overline{\boldsymbol{u}} \sim \psi_{ac}+\psi_{ce}+\psi_{ev}+\psi_{ac}\cdot\psi_{ce}+\psi_{ev}\cdot\psi_{cv}$	0.144048	0.88786	20493.7
79	$\overline{\boldsymbol{u}} \sim \psi_{ac}+\psi_{ce}+\psi_{cv}+\psi_{ac}\cdot\psi_{ce}+\psi_{ac}\cdot\psi_{cv}+\psi_{ce}\cdot\psi_{ev}+\psi_{ev}\cdot\psi_{cv}$	0.163361	0.88697	20735.7
80	$\overline{\boldsymbol{u}} \sim \psi_{ac}+\psi_{ce}+\psi_{ev}+\psi_{ac}\cdot\psi_{ce}+\psi_{ac}\cdot\psi_{ev}+\psi_{ac}\cdot\psi_{cv}+\psi_{ce}\cdot\psi_{ev}$	0.141728	0.88639	20891.6
81	$\overline{\boldsymbol{u}} \sim \psi_{ac}+\psi_{ce}+\psi_{ev}+\psi_{ac}\cdot\psi_{ce}+\psi_{ac}\cdot\psi_{ev}+\psi_{ac}\cdot\psi_{cv}$	0.141609	0.88638	20893.4
82	$\overline{\boldsymbol{u}} \sim \psi_{ac}+\psi_{ce}+\psi_{ev}+\psi_{ac}\cdot\psi_{ce}+\psi_{ac}\cdot\psi_{ev}+\psi_{ce}\cdot\psi_{ev}+\psi_{ce}\cdot\psi_{cv}$	0.134263	0.8863	20915.4
83	$\overline{\boldsymbol{u}} \sim \psi_{ac}+\psi_{ce}+\psi_{ev}+\psi_{ac}\cdot\psi_{ce}+\psi_{ac}\cdot\psi_{ev}+\psi_{ce}\cdot\psi_{cv}$	0.134148	0.88629	20917.1
84	$\overline{\boldsymbol{u}} \sim \psi_{ac}+\psi_{ce}+\psi_{ev}+\psi_{ac}\cdot\psi_{ce}+\psi_{ac}\cdot\psi_{cv}+\psi_{ce}\cdot\psi_{ev}$	0.13958	0.88548	21131.9
85	$\overline{\boldsymbol{u}} \sim \psi_{ac}+\psi_{ce}+\psi_{ev}+\psi_{ac}\cdot\psi_{ce}+\psi_{ac}\cdot\psi_{cv}$	0.139291	0.88542	21145.1
86	$\overline{\boldsymbol{u}} \sim \psi_{ac}+\psi_{ce}+\psi_{ev}+\psi_{ac}\cdot\psi_{ce}+\psi_{ce}\cdot\psi_{ev}+\psi_{ce}\cdot\psi_{cv}$	0.132163	0.8854	21153.4
87	$\overline{\boldsymbol{u}} \sim \psi_{ac}+\psi_{ce}+\psi_{ev}+\psi_{ac}\cdot\psi_{ce}+\psi_{ce}\cdot\psi_{cv}$	0.131882	0.88534	21166.4
88	$\overline{\boldsymbol{u}} \sim \psi_{ac}+\psi_{ce}+\psi_{ev}+\psi_{ac}\cdot\psi_{ce}+\psi_{ac}\cdot\psi_{ev}+\psi_{ce}\cdot\psi_{ev}$	0.138961	0.88531	21176.3
89	$\overline{\boldsymbol{u}} \sim \psi_{ac}+\psi_{ce}+\psi_{ev}+\psi_{ac}\cdot\psi_{ce}+\psi_{ac}\cdot\psi_{ev}$	0.138845	0.8853	21178
90	$\overline{\boldsymbol{u}} \sim \psi_{ac}+\psi_{ce}+\psi_{ev}+\psi_{ac}\cdot\psi_{ce}+\psi_{ce}\cdot\psi_{ev}$	0.136854	0.88441	21412.6
91	$\overline{\boldsymbol{u}} \sim \psi_{ac}+\psi_{ce}+\psi_{ev}+\psi_{ac}\cdot\psi_{ce}$	0.136572	0.88435	21425.5
92	$\overline{\boldsymbol{u}} \sim \psi_{ac}+\psi_{ce}+\psi_{ac}\cdot\psi_{ce}+\psi_{ac}\cdot\psi_{ev}+\psi_{ac}\cdot\psi_{cv}+\psi_{ce}\cdot\psi_{ev}+\psi_{ev}\cdot\psi_{cv}$	0.1445	0.88239	21937.1
93	$\overline{\boldsymbol{u}} \sim \psi_{ac}+\psi_{ce}+\psi_{ac}\cdot\psi_{ce}+\psi_{ac}\cdot\psi_{ev}+\psi_{ac}\cdot\psi_{cv}+\psi_{ev}\cdot\psi_{cv}$	0.145488	0.88137	22197.1
94	$\overline{\boldsymbol{u}} \sim \psi_{ac}+\psi_{ce}+\psi_{cv}+\psi_{ac}\cdot\psi_{ce}+\psi_{ce}\cdot\psi_{ev}+\psi_{ce}\cdot\psi_{cv}$	0.114194	0.88099	22295.8
95	$\overline{\boldsymbol{u}} \sim \psi_{ac}+\psi_{ce}+\psi_{ac}\cdot\psi_{ce}+\psi_{ac}\cdot\psi_{cv}+\psi_{ce}\cdot\psi_{ev}+\psi_{ev}\cdot\psi_{cv}$	0.144479	0.88029	22473.6
96	$\overline{\boldsymbol{u}} \sim \psi_{ac}+\psi_{ce}+\psi_{cv}+\psi_{ac}\cdot\psi_{ce}+\psi_{ac}\cdot\psi_{ev}+\psi_{ce}\cdot\psi_{ev}+\psi_{ev}\cdot\psi_{cv}$	0.139691	0.87741	23191.9
97	$\overline{\boldsymbol{u}} \sim \psi_{ac}+\psi_{ce}+\psi_{ac}\cdot\psi_{ce}+\psi_{ac}\cdot\psi_{ev}+\psi_{ce}\cdot\psi_{ev}+\psi_{ev}\cdot\psi_{cv}$	0.139977	0.87739	23195.7
98	$\overline{\boldsymbol{u}} \sim \psi_{ac}+\psi_{ce}+\psi_{cv}+\psi_{ac}\cdot\psi_{ce}+\psi_{ac}\cdot\psi_{cv}+\psi_{ev}\cdot\psi_{cv}$	0.160509	0.87716	23253.2
99	$\overline{\boldsymbol{u}} \sim \psi_{ac}+\psi_{ce}+\psi_{ac}\cdot\psi_{ce}+\psi_{ac}\cdot\psi_{ev}+\psi_{ev}\cdot\psi_{cv}$	0.141008	0.87656	23400.6
100	$\overline{\boldsymbol{u}} \sim \psi_{ac}+\psi_{ce}+\psi_{cv}+\psi_{ac}\cdot\psi_{ce}+\psi_{ac}\cdot\psi_{ev}+\psi_{ev}\cdot\psi_{cv}$	0.140817	0.87656	23400
101	$\overline{\boldsymbol{u}} \sim \psi_{ac}+\psi_{ce}+\psi_{cv}+\psi_{ac}\cdot\psi_{ce}+\psi_{ac}\cdot\psi_{ev}+\psi_{ce}\cdot\psi_{ev}$	0.139922	0.87646	23426.4
102	$\overline{\boldsymbol{u}} \sim \psi_{ac}+\psi_{ce}+\psi_{cv}+\psi_{ac}\cdot\psi_{ce}+\psi_{ac}\cdot\psi_{ev}$	0.141005	0.8757	23608.9
103	$\overline{\boldsymbol{u}} \sim \psi_{ac}+\psi_{ce}+\psi_{ac}\cdot\psi_{ce}+\psi_{ac}\cdot\psi_{cv}+\psi_{ev}\cdot\psi_{cv}$	0.147668	0.87388	24050.8
104	$\overline{\boldsymbol{u}} \sim \psi_{ac}+\psi_{ce}+\psi_{ac}\cdot\psi_{ce}+\psi_{ac}\cdot\psi_{ev}+\psi_{ac}\cdot\psi_{cv}+\psi_{ce}\cdot\psi_{ev}$	0.134397	0.87357	24125.6

105	$\overline{\boldsymbol{u}} \sim \psi_{ac}+\psi_{ce}+\psi_{ac}\cdot\psi_{ce}+\psi_{ac}\cdot\psi_{ev}+\psi_{ce}\cdot\psi_{ev}+\psi_{ce}\cdot\psi_{cv}$	0.127576	0.87349	24145.2
106	$\overline{\boldsymbol{u}} \sim \psi_{ac}+\psi_{ce}+\psi_{ac}\cdot\psi_{ce}+\psi_{ac}\cdot\psi_{ev}+\psi_{ac}\cdot\psi_{cv}$	0.135438	0.8728	24306.7
107	$\overline{\boldsymbol{u}} \sim \psi_{ac}+\psi_{ce}+\psi_{ac}\cdot\psi_{ce}+\psi_{ac}\cdot\psi_{ev}+\psi_{ce}\cdot\psi_{cv}$	0.128644	0.87271	24328.8
108	$\overline{\boldsymbol{u}} \sim \psi_{ac}+\psi_{ce}+\psi_{ac}\cdot\psi_{ce}+\psi_{ac}\cdot\psi_{ev}+\psi_{ce}\cdot\psi_{ev}$	0.131926	0.87251	24376.8
109	$\overline{\boldsymbol{u}} \sim \psi_{ac}+\psi_{ce}+\psi_{ac}\cdot\psi_{ce}+\psi_{ac}\cdot\psi_{ev}$	0.132962	0.87174	24557.5
110	$\overline{\boldsymbol{u}} \sim \psi_{ac}+\psi_{ce}+\psi_{cv}+\psi_{ac}\cdot\psi_{ce}+\psi_{ce}\cdot\psi_{ev}+\psi_{ev}\cdot\psi_{cv}$	0.133834	0.87069	24806.2
111	$\overline{\boldsymbol{u}} \sim \psi_{ac}+\psi_{ce}+\psi_{cv}+\psi_{ac}\cdot\psi_{ce}+\psi_{ac}\cdot\psi_{cv}+\psi_{ce}\cdot\psi_{ev}$	0.147724	0.86388	26360
112	$\overline{\boldsymbol{u}} \sim \psi_{ac}+\psi_{ce}+\psi_{ac}\cdot\psi_{ce}+\psi_{ce}\cdot\psi_{ev}+\psi_{ev}\cdot\psi_{cv}$	0.134296	0.86224	26721.2
113	$\overline{\boldsymbol{u}} \sim \psi_{ac}+\psi_{ce}+\psi_{ac}\cdot\psi_{ce}+\psi_{ac}\cdot\psi_{cv}+\psi_{ce}\cdot\psi_{ev}+\psi_{ce}\cdot\psi_{cv}$	0.104642	0.86171	26837.1
114	$\overline{\boldsymbol{u}} \sim \psi_{ac}+\psi_{ce}+\psi_{cv}+\psi_{ac}\cdot\psi_{ce}+\psi_{ev}\cdot\psi_{cv}$	0.133297	0.86086	27022.1
115	$\overline{\boldsymbol{u}} \sim \psi_{ac}+\psi_{ce}+\psi_{cv}+\psi_{ac}\cdot\psi_{ce}+\psi_{ce}\cdot\psi_{ev}$	0.124553	0.84841	29615.7
116	$\overline{\boldsymbol{u}} \sim \psi_{ac}+\psi_{ce}+\psi_{ac}\cdot\psi_{ce}+\psi_{ce}\cdot\psi_{ev}+\psi_{ce}\cdot\psi_{cv}$	0.114901	0.84579	30135
117	$\overline{\boldsymbol{u}} \sim \psi_{ac}+\psi_{ce}+\psi_{ac}\cdot\psi_{ce}+\psi_{ac}\cdot\psi_{cv}+\psi_{ce}\cdot\psi_{ev}$	0.120618	0.84568	30155
118	$\overline{\boldsymbol{u}} \sim \psi_{ac}+\psi_{ce}+\psi_{ac}\cdot\psi_{ce}+\psi_{ce}\cdot\psi_{ev}$	0.118698	0.8447	30347.3
119	$\overline{\boldsymbol{u}} \sim \psi_{ce}+\psi_{ev}+\psi_{cv}+\psi_{ac}\cdot\psi_{ce}+\psi_{ac}\cdot\psi_{ev}+\psi_{ac}\cdot\psi_{cv}+\psi_{ce}\cdot\psi_{ev}+\psi_{ce}\cdot\psi_{cv}+\psi_{ev}\cdot\psi_{cv}$	0.148827	0.82438	34071.9
120	$\overline{\boldsymbol{u}} \sim \psi_{ce}+\psi_{ev}+\psi_{cv}+\psi_{ac}\cdot\psi_{ce}+\psi_{ac}\cdot\psi_{ev}+\psi_{ac}\cdot\psi_{cv}+\psi_{ce}\cdot\psi_{cv}+\psi_{ev}\cdot\psi_{cv}$	0.149241	0.82313	34286.6
121	$\overline{\boldsymbol{u}} \sim \psi_{ce}+\psi_{ev}+\psi_{cv}+\psi_{ac}\cdot\psi_{ce}+\psi_{ac}\cdot\psi_{ev}+\psi_{ac}\cdot\psi_{cv}+\psi_{ce}\cdot\psi_{ev}+\psi_{ce}\cdot\psi_{cv}$	0.146062	0.81411	35790
122	$\overline{\boldsymbol{u}} \sim \psi_{ce}+\psi_{ev}+\psi_{cv}+\psi_{ac}\cdot\psi_{ce}+\psi_{ac}\cdot\psi_{ev}+\psi_{ac}\cdot\psi_{cv}+\psi_{ce}\cdot\psi_{ev}+\psi_{ev}\cdot\psi_{cv}$	0.149531	0.81345	35897.9
123	$\overline{\boldsymbol{u}} \sim \psi_{ce}+\psi_{ev}+\psi_{cv}+\psi_{ac}\cdot\psi_{ce}+\psi_{ac}\cdot\psi_{ev}+\psi_{ac}\cdot\psi_{cv}+\psi_{ce}\cdot\psi_{cv}$	0.146264	0.81327	35925.5
124	$\overline{\boldsymbol{u}} \sim \psi_{ce}+\psi_{ev}+\psi_{cv}+\psi_{ac}\cdot\psi_{ce}+\psi_{ac}\cdot\psi_{ev}+\psi_{ac}\cdot\psi_{cv}+\psi_{ev}\cdot\psi_{cv}$	0.149797	0.8123	36082.8
125	$\overline{\boldsymbol{u}} \sim \psi_{ac}+\psi_{ce}+\psi_{ac}\cdot\psi_{ce}+\psi_{ev}\cdot\psi_{cv}$	0.139127	0.81186	36150.9
126	$\overline{\boldsymbol{u}} \sim \psi_{ce}+\psi_{ev}+\psi_{cv}+\psi_{ac}\cdot\psi_{ce}+\psi_{ac}\cdot\psi_{ev}+\psi_{ac}\cdot\psi_{cv}+\psi_{ce}\cdot\psi_{ev}$	0.146467	0.80426	37351.9
127	$\overline{\boldsymbol{u}} \sim \psi_{ce}+\psi_{ev}+\psi_{cv}+\psi_{ac}\cdot\psi_{ce}+\psi_{ac}\cdot\psi_{ev}+\psi_{ac}\cdot\psi_{cv}$	0.146585	0.80348	37470.7
128	$\overline{\boldsymbol{u}} \sim \psi_{ce}+\psi_{ev}+\psi_{ac}\cdot\psi_{ce}+\psi_{ac}\cdot\psi_{ev}+\psi_{ac}\cdot\psi_{cv}+\psi_{ce}\cdot\psi_{ev}+\psi_{ce}\cdot\psi_{cv}+\psi_{ev}\cdot\psi_{cv}$	0.122155	0.76241	43216.6
129	$\overline{\boldsymbol{u}} \sim \psi_{ce}+\psi_{ev}+\psi_{ac}\cdot\psi_{ce}+\psi_{ac}\cdot\psi_{ev}+\psi_{ac}\cdot\psi_{cv}+\psi_{ce}\cdot\psi_{ev}+\psi_{ev}\cdot\psi_{cv}$	0.123747	0.76092	43404.5
130	$\overline{\boldsymbol{u}} \sim \psi_{ce}+\psi_{ev}+\psi_{ac}\cdot\psi_{ce}+\psi_{ac}\cdot\psi_{ev}+\psi_{ac}\cdot\psi_{cv}+\psi_{ce}\cdot\psi_{cv}+\psi_{ev}\cdot\psi_{cv}$	0.123193	0.75914	43628.9
131	$\overline{\boldsymbol{u}} \sim \psi_{ce}+\psi_{ev}+\psi_{ac}\cdot\psi_{ce}+\psi_{ac}\cdot\psi_{ev}+\psi_{ac}\cdot\psi_{cv}+\psi_{ev}\cdot\psi_{cv}$	0.124562	0.75783	43792.3

132	$\overline{\boldsymbol{u}} \sim \psi_{ac}+\psi_{ce}+\psi_{cv}+\psi_{ac}\cdot\psi_{ce}+\psi_{ac}\cdot\psi_{cv}+\psi_{ce}\cdot\psi_{cv}$	0.107498	0.7393	46022.4
133	$\overline{\boldsymbol{u}} \sim \psi_{ac}+\psi_{ev}+\psi_{cv}+\psi_{ac}\cdot\psi_{ce}+\psi_{ac}\cdot\psi_{ev}+\psi_{ac}\cdot\psi_{cv}+\psi_{ce}\cdot\psi_{ev}+\psi_{ce}\cdot\psi_{cv}$	0.202106	0.73772	46207.8
134	$\overline{\boldsymbol{u}} \sim \psi_{ac}+\psi_{ev}+\psi_{cv}+\psi_{ac}\cdot\psi_{ce}+\psi_{ac}\cdot\psi_{ev}+\psi_{ac}\cdot\psi_{cv}+\psi_{ce}\cdot\psi_{ev}+\psi_{ce}\cdot\psi_{cv}+\psi_{ev}\cdot\psi_{cv}$	0.201997	0.73772	46209.1
135	$\overline{\boldsymbol{u}} \sim \psi_{ac}+\psi_{ev}+\psi_{cv}+\psi_{ac}\cdot\psi_{ce}+\psi_{ac}\cdot\psi_{cv}+\psi_{ce}\cdot\psi_{ev}+\psi_{ce}\cdot\psi_{cv}$	0.202507	0.73762	46217.8
136	$\overline{\boldsymbol{u}} \sim \psi_{ac}+\psi_{ev}+\psi_{cv}+\psi_{ac}\cdot\psi_{ce}+\psi_{ac}\cdot\psi_{cv}+\psi_{ce}\cdot\psi_{ev}+\psi_{ce}\cdot\psi_{cv}+\psi_{ev}\cdot\psi_{cv}$	0.202541	0.73762	46219.5
137	$\overline{\boldsymbol{u}} \sim \psi_{ac}+\psi_{ce}+\psi_{cv}+\psi_{ac}\cdot\psi_{ce}+\psi_{ce}\cdot\psi_{cv}$	0.0870098	0.73292	46753.2
138	$\overline{\boldsymbol{u}} \sim \psi_{ce}+\psi_{cv}+\psi_{ac}\cdot\psi_{ce}+\psi_{ac}\cdot\psi_{ev}+\psi_{ac}\cdot\psi_{cv}+\psi_{ce}\cdot\psi_{ev}+\psi_{ce}\cdot\psi_{cv}+\psi_{ev}\cdot\psi_{cv}$	0.113728	0.73149	46918.2
139	$\overline{\boldsymbol{u}} \sim \psi_{ce}+\psi_{cv}+\psi_{ac}\cdot\psi_{ce}+\psi_{ac}\cdot\psi_{ev}+\psi_{ac}\cdot\psi_{cv}+\psi_{ce}\cdot\psi_{ev}+\psi_{ev}\cdot\psi_{cv}$	0.114359	0.72659	47464.2
140	$\overline{\boldsymbol{u}} \sim \psi_{ce}+\psi_{ev}+\psi_{cv}+\psi_{ac}\cdot\psi_{ce}+\psi_{ac}\cdot\psi_{cv}+\psi_{ce}\cdot\psi_{ev}+\psi_{ce}\cdot\psi_{cv}+\psi_{ev}\cdot\psi_{cv}$	0.120437	0.72566	47568.4
141	$\overline{\boldsymbol{u}} \sim \psi_{ce}+\psi_{ev}+\psi_{cv}+\psi_{ac}\cdot\psi_{ce}+\psi_{ac}\cdot\psi_{cv}+\psi_{ce}\cdot\psi_{cv}+\psi_{ev}\cdot\psi_{cv}$	0.120428	0.72565	47568.3
142	$\overline{\boldsymbol{u}} \sim \psi_{ce}+\psi_{ev}+\psi_{cv}+\psi_{ac}\cdot\psi_{ce}+\psi_{ac}\cdot\psi_{cv}+\psi_{ce}\cdot\psi_{cv}$	0.120407	0.72561	47572
143	$\overline{\boldsymbol{u}} \sim \psi_{ce}+\psi_{ev}+\psi_{cv}+\psi_{ac}\cdot\psi_{ce}+\psi_{ac}\cdot\psi_{cv}+\psi_{ce}\cdot\psi_{ev}+\psi_{ce}\cdot\psi_{cv}$	0.120416	0.72561	47572.4
144	$\overline{\boldsymbol{u}} \sim \psi_{ce}+\psi_{ev}+\psi_{cv}+\psi_{ac}\cdot\psi_{ce}+\psi_{ac}\cdot\psi_{cv}+\psi_{ce}\cdot\psi_{ev}+\psi_{ev}\cdot\psi_{cv}$	0.12004	0.72294	47865.4
145	$\overline{\boldsymbol{u}} \sim \psi_{ce}+\psi_{ev}+\psi_{cv}+\psi_{ac}\cdot\psi_{ce}+\psi_{ac}\cdot\psi_{cv}+\psi_{ev}\cdot\psi_{cv}$	0.12003	0.72293	47865.4
146	$\overline{\boldsymbol{u}} \sim \psi_{ce}+\psi_{ev}+\psi_{cv}+\psi_{ac}\cdot\psi_{ce}+\psi_{ac}\cdot\psi_{cv}+\psi_{ce}\cdot\psi_{ev}$	0.12002	0.7229	47869
147	$\overline{\boldsymbol{u}} \sim \psi_{ce}+\psi_{ev}+\psi_{cv}+\psi_{ac}\cdot\psi_{ce}+\psi_{ac}\cdot\psi_{cv}$	0.120011	0.72289	47868.7
148	$\overline{\boldsymbol{u}} \sim \psi_{ev}+\psi_{cv}+\psi_{ac}\cdot\psi_{ce}+\psi_{ac}\cdot\psi_{ev}+\psi_{ac}\cdot\psi_{cv}+\psi_{ce}\cdot\psi_{ev}+\psi_{ce}\cdot\psi_{cv}+\psi_{ev}\cdot\psi_{cv}$	0.181269	0.72271	47891.2
149	$\overline{\boldsymbol{u}} \sim \psi_{ev}+\psi_{cv}+\psi_{ac}\cdot\psi_{ce}+\psi_{ac}\cdot\psi_{ev}+\psi_{ac}\cdot\psi_{cv}+\psi_{ce}\cdot\psi_{ev}+\psi_{ce}\cdot\psi_{cv}$	0.178767	0.72135	48039
150	$\overline{\boldsymbol{u}} \sim \psi_{ac}+\psi_{cv}+\psi_{ac}\cdot\psi_{ce}+\psi_{ac}\cdot\psi_{ev}+\psi_{ac}\cdot\psi_{cv}+\psi_{ce}\cdot\psi_{ev}+\psi_{ce}\cdot\psi_{cv}+\psi_{ev}\cdot\psi_{cv}$	0.210601	0.71997	48189.4
151	$\overline{\boldsymbol{u}} \sim \psi_{ac}+\psi_{cv}+\psi_{ac}\cdot\psi_{ce}+\psi_{ac}\cdot\psi_{ev}+\psi_{ac}\cdot\psi_{cv}+\psi_{ce}\cdot\psi_{ev}+\psi_{ce}\cdot\psi_{cv}$	0.210994	0.71981	48205.5
152	$\overline{\boldsymbol{u}} \sim \psi_{ac}+\psi_{ce}+\psi_{ac}\cdot\psi_{ce}+\psi_{ac}\cdot\psi_{cv}+\psi_{ce}\cdot\psi_{cv}$	0.0770066	0.71468	48752.3
153	$\overline{\boldsymbol{u}} \sim \psi_{ac}+\psi_{ce}+\psi_{cv}+\psi_{ac}\cdot\psi_{ce}+\psi_{ac}\cdot\psi_{cv}$	0.125429	0.7134	48888
154	$\overline{\boldsymbol{u}} \sim \psi_{ce}+\psi_{ev}+\psi_{cv}+\psi_{ac}\cdot\psi_{ce}+\psi_{ac}\cdot\psi_{ev}+\psi_{ce}\cdot\psi_{ev}+\psi_{ce}\cdot\psi_{cv}+\psi_{ev}\cdot\psi_{cv}$	0.115868	0.7116	49080.5
155	$\overline{\boldsymbol{u}} \sim \psi_{ce}+\psi_{ev}+\psi_{ac}\cdot\psi_{ce}+\psi_{ac}\cdot\psi_{ev}+\psi_{ce}\cdot\psi_{ev}+\psi_{ce}\cdot\psi_{cv}+\psi_{ev}\cdot\psi_{cv}$	0.115821	0.71132	49108.4
156	$\overline{\boldsymbol{u}} \sim \psi_{ce}+\psi_{cv}+\psi_{ac}\cdot\psi_{ce}+\psi_{ac}\cdot\psi_{cv}+\psi_{ce}\cdot\psi_{ev}+\psi_{ce}\cdot\psi_{cv}+\psi_{ev}\cdot\psi_{cv}$	0.118506	0.71107	49134.7
157	$\overline{\boldsymbol{u}} \sim \psi_{ac}+\psi_{ev}+\psi_{cv}+\psi_{ac}\cdot\psi_{ce}+\psi_{ac}\cdot\psi_{ev}+\psi_{ac}\cdot\psi_{cv}+\psi_{ce}\cdot\psi_{cv}+\psi_{ev}\cdot\psi_{cv}$	0.256011	0.70854	49399.8
158	$\overline{\boldsymbol{u}} \sim \psi_{ac}+\psi_{ev}+\psi_{cv}+\psi_{ac}\cdot\psi_{ce}+\psi_{ac}\cdot\psi_{cv}+\psi_{ce}\cdot\psi_{cv}+\psi_{ev}\cdot\psi_{cv}$	0.256167	0.70846	49407.3

159	$\overline{\boldsymbol{u}} \sim \psi_{ac}+\psi_{ev}+\psi_{cv}+\psi_{ac}\cdot\psi_{ce}+\psi_{ac}\cdot\psi_{ev}+\psi_{ac}\cdot\psi_{cv}+\psi_{ce}\cdot\psi_{cv}$	0.256016	0.70844	49409.4
160	$\overline{\boldsymbol{u}} \sim \psi_{ac}+\psi_{ev}+\psi_{cv}+\psi_{ac}\cdot\psi_{ce}+\psi_{ac}\cdot\psi_{cv}+\psi_{ce}\cdot\psi_{cv}$	0.256119	0.70841	49411.2
161	$\overline{\boldsymbol{u}} \sim \psi_{ce}+\psi_{cv}+\psi_{ac}\cdot\psi_{ce}+\psi_{ac}\cdot\psi_{cv}+\psi_{ce}\cdot\psi_{ev}+\psi_{ev}\cdot\psi_{cv}$	0.118135	0.70834	49418.6
162	$\overline{\boldsymbol{u}} \sim \psi_{ce}+\psi_{ev}+\psi_{cv}+\psi_{ac}\cdot\psi_{ce}+\psi_{ac}\cdot\psi_{ev}+\psi_{ce}\cdot\psi_{cv}+\psi_{ev}\cdot\psi_{cv}$	0.117362	0.70762	49494
163	$\overline{\boldsymbol{u}} \sim \psi_{ce}+\psi_{ev}+\psi_{ac}\cdot\psi_{ce}+\psi_{ac}\cdot\psi_{ev}+\psi_{ce}\cdot\psi_{cv}+\psi_{ev}\cdot\psi_{cv}$	0.117279	0.70742	49513.9
164	$\overline{\boldsymbol{u}} \sim \psi_{ce}+\psi_{cv}+\psi_{ac}\cdot\psi_{ce}+\psi_{ac}\cdot\psi_{ev}+\psi_{ac}\cdot\psi_{cv}+\psi_{ce}\cdot\psi_{cv}+\psi_{ev}\cdot\psi_{cv}$	0.115535	0.70402	49864.8
165	$\overline{\boldsymbol{u}} \sim \psi_{ev}+\psi_{cv}+\psi_{ac}\cdot\psi_{ce}+\psi_{ac}\cdot\psi_{ev}+\psi_{ac}\cdot\psi_{cv}+\psi_{ce}\cdot\psi_{cv}+\psi_{ev}\cdot\psi_{cv}$	0.215951	0.70365	49902.5
166	$\overline{\boldsymbol{u}} \sim \psi_{ac}+\psi_{ev}+\psi_{cv}+\psi_{ac}\cdot\psi_{ce}+\psi_{ac}\cdot\psi_{ev}+\psi_{ac}\cdot\psi_{cv}+\psi_{ce}\cdot\psi_{ev}+\psi_{ev}\cdot\psi_{cv}$	0.229869	0.70313	49956.5
167	$\overline{\boldsymbol{u}} \sim \psi_{ac}+\psi_{ev}+\psi_{cv}+\psi_{ac}\cdot\psi_{ce}+\psi_{ac}\cdot\psi_{ev}+\psi_{ac}\cdot\psi_{cv}+\psi_{ce}\cdot\psi_{ev}$	0.230081	0.70312	49956.4
168	$\overline{\boldsymbol{u}} \sim \psi_{ac}+\psi_{ev}+\psi_{cv}+\psi_{ac}\cdot\psi_{ce}+\psi_{ac}\cdot\psi_{cv}+\psi_{ce}\cdot\psi_{ev}$	0.230649	0.70294	49973.1
169	$\overline{\boldsymbol{u}} \sim \psi_{ac}+\psi_{ev}+\psi_{cv}+\psi_{ac}\cdot\psi_{ce}+\psi_{ac}\cdot\psi_{cv}+\psi_{ce}\cdot\psi_{ev}+\psi_{ev}\cdot\psi_{cv}$	0.230679	0.70294	49975
170	$\overline{\boldsymbol{u}} \sim \psi_{ev}+\psi_{cv}+\psi_{ac}\cdot\psi_{ce}+\psi_{ac}\cdot\psi_{ev}+\psi_{ac}\cdot\psi_{cv}+\psi_{ce}\cdot\psi_{cv}$	0.211994	0.7026	50008.2
171	$\overline{\boldsymbol{u}} \sim \psi_{ac}+\psi_{ce}+\psi_{ev}+\psi_{cv}+\psi_{ac}\cdot\psi_{ev}+\psi_{ac}\cdot\psi_{cv}+\psi_{ce}\cdot\psi_{ev}+\psi_{ce}\cdot\psi_{cv}+\psi_{ev}\cdot\psi_{cv}$	0.360939	0.70173	50099.6
172	$\overline{\boldsymbol{u}} \sim \psi_{ac}+\psi_{ce}+\psi_{ev}+\psi_{cv}+\psi_{ac}\cdot\psi_{ev}+\psi_{ac}\cdot\psi_{cv}+\psi_{ce}\cdot\psi_{ev}+\psi_{ev}\cdot\psi_{cv}$	0.355941	0.70167	50104.7
173	$\overline{\boldsymbol{u}} \sim \psi_{ac}+\psi_{ce}+\psi_{ev}+\psi_{cv}+\psi_{ac}\cdot\psi_{ev}+\psi_{ac}\cdot\psi_{cv}+\psi_{ce}\cdot\psi_{cv}+\psi_{ev}\cdot\psi_{cv}$	0.360845	0.70167	50104.2
174	$\overline{\boldsymbol{u}} \sim \psi_{ac}+\psi_{ce}+\psi_{ev}+\psi_{cv}+\psi_{ac}\cdot\psi_{cv}+\psi_{ce}\cdot\psi_{ev}+\psi_{ce}\cdot\psi_{cv}+\psi_{ev}\cdot\psi_{cv}$	0.361253	0.70167	50104.9
175	$\overline{\boldsymbol{u}} \sim \psi_{ac}+\psi_{ce}+\psi_{ev}+\psi_{cv}+\psi_{ac}\cdot\psi_{ev}+\psi_{ac}\cdot\psi_{cv}+\psi_{ce}\cdot\psi_{ev}+\psi_{ce}\cdot\psi_{cv}$	0.360964	0.70164	50107.5
176	$\overline{\boldsymbol{u}} \sim \psi_{ac}+\psi_{ce}+\psi_{ev}+\psi_{cv}+\psi_{ac}\cdot\psi_{cv}+\psi_{ce}\cdot\psi_{ev}+\psi_{ce}\cdot\psi_{cv}$	0.361157	0.70163	50108.2
177	$\overline{\boldsymbol{u}} \sim \psi_{ac}+\psi_{ce}+\psi_{ev}+\psi_{cv}+\psi_{ac}\cdot\psi_{cv}+\psi_{ce}\cdot\psi_{cv}+\psi_{ev}\cdot\psi_{cv}$	0.36111	0.70163	50107.7
178	$\overline{\boldsymbol{u}} \sim \psi_{ac}+\psi_{ce}+\psi_{ev}+\psi_{cv}+\psi_{ac}\cdot\psi_{ev}+\psi_{ac}\cdot\psi_{cv}+\psi_{ev}\cdot\psi_{cv}$	0.355858	0.70162	50109.2
179	$\overline{\boldsymbol{u}} \sim \psi_{ac}+\psi_{ce}+\psi_{ev}+\psi_{cv}+\psi_{ac}\cdot\psi_{cv}+\psi_{ce}\cdot\psi_{ev}+\psi_{ev}\cdot\psi_{cv}$	0.356209	0.70161	50110.1
180	$\overline{\boldsymbol{u}} \sim \psi_{ac}+\psi_{ce}+\psi_{ev}+\psi_{cv}+\psi_{ac}\cdot\psi_{ev}+\psi_{ac}\cdot\psi_{cv}+\psi_{ce}\cdot\psi_{cv}$	0.360853	0.7016	50110.8
181	$\overline{\boldsymbol{u}} \sim \psi_{ac}+\psi_{ce}+\psi_{ev}+\psi_{cv}+\psi_{ac}\cdot\psi_{cv}+\psi_{ce}\cdot\psi_{cv}$	0.361013	0.70159	50110.7
182	$\overline{\boldsymbol{u}} \sim \psi_{ac}+\psi_{ce}+\psi_{ev}+\psi_{cv}+\psi_{ac}\cdot\psi_{ev}+\psi_{ac}\cdot\psi_{cv}+\psi_{ce}\cdot\psi_{ev}$	0.355977	0.70158	50112.5
183	$\overline{\boldsymbol{u}} \sim \psi_{ac}+\psi_{ce}+\psi_{ev}+\psi_{cv}+\psi_{ac}\cdot\psi_{cv}+\psi_{ce}\cdot\psi_{ev}$	0.356138	0.70157	50113.3
184	$\overline{\boldsymbol{u}} \sim \psi_{ac}+\psi_{ce}+\psi_{ev}+\psi_{cv}+\psi_{ac}\cdot\psi_{cv}+\psi_{ev}\cdot\psi_{cv}$	0.356082	0.70157	50112.9
185	$\overline{\boldsymbol{u}} \sim \psi_{ac}+\psi_{ce}+\psi_{ev}+\psi_{cv}+\psi_{ac}\cdot\psi_{ev}+\psi_{ac}\cdot\psi_{cv}$	0.355877	0.70154	50115.8

186	$\overline{\boldsymbol{u}} \sim \psi_{ac}+\psi_{ce}+\psi_{ev}+\psi_{cv}+\psi_{ac}\cdot\psi_{cv}$	0.35601	0.70153	50115.7
187	$\overline{\boldsymbol{u}} \sim \psi_{ev}+\psi_{cv}+\psi_{ac}\cdot\psi_{ce}+\psi_{ac}\cdot\psi_{cv}+\psi_{ce}\cdot\psi_{ev}+\psi_{ce}\cdot\psi_{cv}+\psi_{ev}\cdot\psi_{cv}$	0.150957	0.70129	50142
188	$\overline{\boldsymbol{u}} \sim \psi_{ev}+\psi_{cv}+\psi_{ac}\cdot\psi_{ce}+\psi_{ac}\cdot\psi_{cv}+\psi_{ce}\cdot\psi_{ev}+\psi_{ce}\cdot\psi_{cv}$	0.150908	0.70128	50142
189	$\overline{\boldsymbol{u}} \sim \psi_{ce}+\psi_{cv}+\psi_{ac}\cdot\psi_{ce}+\psi_{ac}\cdot\psi_{ev}+\psi_{ac}\cdot\psi_{cv}+\psi_{ev}\cdot\psi_{cv}$	0.115561	0.70053	50217.6
190	$\overline{\boldsymbol{u}} \sim \psi_{ce}+\psi_{cv}+\psi_{ac}\cdot\psi_{ce}+\psi_{ac}\cdot\psi_{cv}+\psi_{ce}\cdot\psi_{cv}+\psi_{ev}\cdot\psi_{cv}$	0.117592	0.70022	50249.7
191	$\overline{\boldsymbol{u}} \sim \psi_{ac}+\psi_{cv}+\psi_{ac}\cdot\psi_{ce}+\psi_{ac}\cdot\psi_{ev}+\psi_{ac}\cdot\psi_{cv}+\psi_{ce}\cdot\psi_{cv}+\psi_{ev}\cdot\psi_{cv}$	0.245174	0.70015	50257
192	$\overline{\boldsymbol{u}} \sim \psi_{ac}+\psi_{cv}+\psi_{ac}\cdot\psi_{ce}+\psi_{ac}\cdot\psi_{ev}+\psi_{ac}\cdot\psi_{cv}+\psi_{ce}\cdot\psi_{cv}$	0.245907	0.6998	50291.6
193	$\overline{\boldsymbol{u}} \sim \psi_{ac}+\psi_{ce}+\psi_{cv}+\psi_{ac}\cdot\psi_{ce}$	0.0987034	0.69936	50334.3
194	$\overline{\boldsymbol{u}} \sim \psi_{ce}+\psi_{ev}+\psi_{cv}+\psi_{ac}\cdot\psi_{ev}+\psi_{ac}\cdot\psi_{cv}+\psi_{ce}\cdot\psi_{ev}+\psi_{ce}\cdot\psi_{cv}+\psi_{ev}\cdot\psi_{cv}$	0.252016	0.6981	50464.2
195	$\overline{\boldsymbol{u}} \sim \psi_{ce}+\psi_{ev}+\psi_{cv}+\psi_{ac}\cdot\psi_{ev}+\psi_{ac}\cdot\psi_{cv}+\psi_{ce}\cdot\psi_{ev}+\psi_{ev}\cdot\psi_{cv}$	0.250925	0.69809	50464.7
196	$\overline{\boldsymbol{u}} \sim \psi_{ce}+\psi_{ev}+\psi_{cv}+\psi_{ac}\cdot\psi_{ev}+\psi_{ac}\cdot\psi_{cv}+\psi_{ce}\cdot\psi_{cv}+\psi_{ev}\cdot\psi_{cv}$	0.250594	0.69791	50482.5
197	$\overline{\boldsymbol{u}} \sim \psi_{ce}+\psi_{ev}+\psi_{cv}+\psi_{ac}\cdot\psi_{ev}+\psi_{ac}\cdot\psi_{cv}+\psi_{ev}\cdot\psi_{cv}$	0.24952	0.6979	50483
198	$\overline{\boldsymbol{u}} \sim \psi_{ac}+\psi_{ce}+\psi_{ac}\cdot\psi_{ce}+\psi_{ce}\cdot\psi_{cv}$	0.0871799	0.69766	50505
199	$\overline{\boldsymbol{u}} \sim \psi_{ce}+\psi_{cv}+\psi_{ac}\cdot\psi_{ce}+\psi_{ac}\cdot\psi_{cv}+\psi_{ev}\cdot\psi_{cv}$	0.117219	0.69744	50527.2
200	$\overline{\boldsymbol{u}} \sim \psi_{ce}+\psi_{ev}+\psi_{cv}+\psi_{ac}\cdot\psi_{ev}+\psi_{ac}\cdot\psi_{cv}+\psi_{ce}\cdot\psi_{ev}+\psi_{ce}\cdot\psi_{cv}$	0.24517	0.6974	50533.6
201	$\overline{\boldsymbol{u}} \sim \psi_{ce}+\psi_{ev}+\psi_{cv}+\psi_{ac}\cdot\psi_{ev}+\psi_{ac}\cdot\psi_{cv}+\psi_{ce}\cdot\psi_{ev}$	0.244155	0.69739	50533.9
202	$\overline{\boldsymbol{u}} \sim \psi_{ce}+\psi_{ev}+\psi_{cv}+\psi_{ac}\cdot\psi_{ev}+\psi_{ac}\cdot\psi_{cv}+\psi_{ce}\cdot\psi_{cv}$	0.24414	0.69725	50547.9
203	$\overline{\boldsymbol{u}} \sim \psi_{ce}+\psi_{ev}+\psi_{cv}+\psi_{ac}\cdot\psi_{ev}+\psi_{ac}\cdot\psi_{cv}$	0.243136	0.69723	50548.2
204	$\overline{\boldsymbol{u}} \sim \psi_{ac}+\psi_{ev}+\psi_{cv}+\psi_{ac}\cdot\psi_{cv}+\psi_{ce}\cdot\psi_{ev}+\psi_{ce}\cdot\psi_{cv}+\psi_{ev}\cdot\psi_{cv}$	0.331084	0.69721	50552.8
205	$\overline{\boldsymbol{u}} \sim \psi_{ac}+\psi_{ev}+\psi_{cv}+\psi_{ac}\cdot\psi_{ev}+\psi_{ac}\cdot\psi_{cv}+\psi_{ce}\cdot\psi_{ev}+\psi_{ce}\cdot\psi_{cv}+\psi_{ev}\cdot\psi_{cv}$	0.331026	0.6972	50554.5
206	$\overline{\boldsymbol{u}} \sim \psi_{ac}+\psi_{ev}+\psi_{cv}+\psi_{ac}\cdot\psi_{cv}+\psi_{ce}\cdot\psi_{ev}+\psi_{ce}\cdot\psi_{cv}$	0.331026	0.69718	50554.3
207	$\overline{\boldsymbol{u}} \sim \psi_{ac}+\psi_{ev}+\psi_{cv}+\psi_{ac}\cdot\psi_{ev}+\psi_{ac}\cdot\psi_{cv}+\psi_{ce}\cdot\psi_{ev}+\psi_{ce}\cdot\psi_{cv}$	0.331055	0.69717	50556.2
208	$\overline{\boldsymbol{u}} \sim \psi_{ac}+\psi_{ce}+\psi_{ac}\cdot\psi_{ce}+\psi_{ac}\cdot\psi_{cv}$	0.0947006	0.69695	50575.6
209	$\overline{\boldsymbol{u}} \sim \psi_{ac}+\psi_{ce}+\psi_{ac}\cdot\psi_{ce}$	0.0926186	0.69614	50655.1
210	$\overline{\boldsymbol{u}} \sim \psi_{ev}+\psi_{cv}+\psi_{ac}\cdot\psi_{ce}+\psi_{ac}\cdot\psi_{ev}+\psi_{ac}\cdot\psi_{cv}+\psi_{ce}\cdot\psi_{ev}+\psi_{ev}\cdot\psi_{cv}$	0.195809	0.69599	50674.6
211	$\overline{\boldsymbol{u}} \sim \psi_{ev}+\psi_{cv}+\psi_{ac}\cdot\psi_{ce}+\psi_{ac}\cdot\psi_{ev}+\psi_{ac}\cdot\psi_{cv}+\psi_{ce}\cdot\psi_{ev}$	0.193143	0.69547	50725.3
212	$\overline{\boldsymbol{u}} \sim \psi_{ac}+\psi_{ev}+\psi_{cv}+\psi_{ac}\cdot\psi_{ev}+\psi_{ac}\cdot\psi_{cv}+\psi_{ce}\cdot\psi_{cv}+\psi_{ev}\cdot\psi_{cv}$	0.323027	0.69451	50821.2

213	$\overline{\boldsymbol{u}} \sim \psi_{ac}+\psi_{ev}+\psi_{cv}+\psi_{ac}\cdot\psi_{cv}+\psi_{ce}\cdot\psi_{cv}+\psi_{ev}\cdot\psi_{cv}$	0.323189	0.69448	50823.7
214	$\overline{\boldsymbol{u}} \sim \psi_{ac}+\psi_{ev}+\psi_{cv}+\psi_{ac}\cdot\psi_{cv}+\psi_{ce}\cdot\psi_{cv}$	0.323117	0.69443	50827
215	$\overline{\boldsymbol{u}} \sim \psi_{ac}+\psi_{ev}+\psi_{cv}+\psi_{ac}\cdot\psi_{ev}+\psi_{ac}\cdot\psi_{cv}+\psi_{ce}\cdot\psi_{cv}$	0.323027	0.69443	50827.8
216	$\overline{\boldsymbol{u}} \sim \psi_{ac}+\psi_{ce}+\psi_{cv}+\psi_{ac}\cdot\psi_{ev}+\psi_{ac}\cdot\psi_{cv}+\psi_{ce}\cdot\psi_{ev}+\psi_{ce}\cdot\psi_{cv}+\psi_{ev}\cdot\psi_{cv}$	0.305606	0.69367	50905.9
217	$\overline{\boldsymbol{u}} \sim \psi_{ac}+\psi_{ce}+\psi_{cv}+\psi_{ac}\cdot\psi_{ev}+\psi_{ac}\cdot\psi_{cv}+\psi_{ce}\cdot\psi_{ev}+\psi_{ev}\cdot\psi_{cv}$	0.303574	0.69364	50907.6
218	$\overline{\boldsymbol{u}} \sim \psi_{ev}+\psi_{cv}+\psi_{ac}\cdot\psi_{ev}+\psi_{ac}\cdot\psi_{cv}+\psi_{ce}\cdot\psi_{ev}+\psi_{ce}\cdot\psi_{cv}+\psi_{ev}\cdot\psi_{cv}$	0.249416	0.69358	50913.2
219	$\overline{\boldsymbol{u}} \sim \psi_{ac}+\psi_{ce}+\psi_{cv}+\psi_{ac}\cdot\psi_{ev}+\psi_{ac}\cdot\psi_{cv}+\psi_{ce}\cdot\psi_{ev}+\psi_{ce}\cdot\psi_{cv}$	0.306692	0.69333	50938.4
220	$\overline{\boldsymbol{u}} \sim \psi_{ac}+\psi_{ce}+\psi_{cv}+\psi_{ac}\cdot\psi_{ev}+\psi_{ac}\cdot\psi_{cv}+\psi_{ce}\cdot\psi_{ev}$	0.304647	0.6933	50940.2
221	$\overline{\boldsymbol{u}} \sim \psi_{ev}+\psi_{cv}+\psi_{ac}\cdot\psi_{ev}+\psi_{ac}\cdot\psi_{cv}+\psi_{ce}\cdot\psi_{ev}+\psi_{ce}\cdot\psi_{cv}$	0.243886	0.69304	50965.3
222	$\overline{\boldsymbol{u}} \sim \psi_{ac}+\psi_{ev}+\psi_{cv}+\psi_{ac}\cdot\psi_{ev}+\psi_{ac}\cdot\psi_{cv}+\psi_{ce}\cdot\psi_{ev}$	0.294403	0.69295	50974.8
223	$\overline{\boldsymbol{u}} \sim \psi_{ac}+\psi_{ev}+\psi_{cv}+\psi_{ac}\cdot\psi_{cv}+\psi_{ce}\cdot\psi_{ev}+\psi_{ev}\cdot\psi_{cv}$	0.29421	0.69294	50975.5
224	$\overline{\boldsymbol{u}} \sim \psi_{ac}+\psi_{ev}+\psi_{cv}+\psi_{ac}\cdot\psi_{ev}+\psi_{ac}\cdot\psi_{cv}+\psi_{ce}\cdot\psi_{ev}+\psi_{ev}\cdot\psi_{cv}$	0.294371	0.69294	50976
225	$\overline{\boldsymbol{u}} \sim \psi_{ac}+\psi_{ev}+\psi_{cv}+\psi_{ac}\cdot\psi_{cv}+\psi_{ce}\cdot\psi_{ev}$	0.294194	0.69293	50975.4
226	$\overline{\boldsymbol{u}} \sim \psi_{ac}+\psi_{ce}+\psi_{cv}+\psi_{ac}\cdot\psi_{ev}+\psi_{ac}\cdot\psi_{cv}+\psi_{ce}\cdot\psi_{cv}+\psi_{ev}\cdot\psi_{cv}$	0.305448	0.69274	50996.1
227	$\overline{\boldsymbol{u}} \sim \psi_{ac}+\psi_{ce}+\psi_{cv}+\psi_{ac}\cdot\psi_{ev}+\psi_{ac}\cdot\psi_{cv}+\psi_{ev}\cdot\psi_{cv}$	0.303438	0.69271	50997.8
228	$\overline{\boldsymbol{u}} \sim \psi_{ac}+\psi_{ce}+\psi_{cv}+\psi_{ac}\cdot\psi_{ev}+\psi_{ac}\cdot\psi_{cv}+\psi_{ce}\cdot\psi_{cv}$	0.306498	0.69247	51022
229	$\overline{\boldsymbol{u}} \sim \psi_{ac}+\psi_{ce}+\psi_{cv}+\psi_{ac}\cdot\psi_{ev}+\psi_{ac}\cdot\psi_{cv}$	0.304473	0.69244	51023.8
230	$\overline{\boldsymbol{u}} \sim \psi_{ce}+\psi_{ev}+\psi_{cv}+\psi_{ac}\cdot\psi_{ce}+\psi_{ac}\cdot\psi_{ev}+\psi_{ce}\cdot\psi_{ev}+\psi_{ev}\cdot\psi_{cv}$	0.120944	0.69161	51106.8
231	$\overline{\boldsymbol{u}} \sim \psi_{ev}+\psi_{cv}+\psi_{ac}\cdot\psi_{ev}+\psi_{ac}\cdot\psi_{cv}+\psi_{ce}\cdot\psi_{cv}+\psi_{ev}\cdot\psi_{cv}$	0.254167	0.69158	51108.7
232	$\overline{\boldsymbol{u}} \sim \psi_{ev}+\psi_{cv}+\psi_{ac}\cdot\psi_{ce}+\psi_{ac}\cdot\psi_{cv}+\psi_{ce}\cdot\psi_{cv}+\psi_{ev}\cdot\psi_{cv}$	0.172617	0.69137	51129.6
233	$\overline{\boldsymbol{u}} \sim \psi_{ev}+\psi_{cv}+\psi_{ac}\cdot\psi_{ce}+\psi_{ac}\cdot\psi_{cv}+\psi_{ce}\cdot\psi_{cv}$	0.17259	0.69132	51133.2
234	$\overline{\boldsymbol{u}} \sim \psi_{ce}+\psi_{ev}+\psi_{cv}+\psi_{ac}\cdot\psi_{cv}+\psi_{ce}\cdot\psi_{ev}+\psi_{ce}\cdot\psi_{cv}+\psi_{ev}\cdot\psi_{cv}$	0.188571	0.69104	51163.1
235	$\overline{\boldsymbol{u}} \sim \psi_{ce}+\psi_{ev}+\psi_{cv}+\psi_{ac}\cdot\psi_{cv}+\psi_{ce}\cdot\psi_{ev}+\psi_{ce}\cdot\psi_{cv}$	0.188532	0.691	51166.2
236	$\overline{\boldsymbol{u}} \sim \psi_{ce}+\psi_{ev}+\psi_{cv}+\psi_{ac}\cdot\psi_{cv}+\psi_{ce}\cdot\psi_{ev}+\psi_{ev}\cdot\psi_{cv}$	0.187437	0.691	51165.6
237	$\overline{\boldsymbol{u}} \sim \psi_{ce}+\psi_{ev}+\psi_{cv}+\psi_{ac}\cdot\psi_{cv}+\psi_{ce}\cdot\psi_{cv}+\psi_{ev}\cdot\psi_{cv}$	0.188538	0.691	51165.7
238	$\overline{\boldsymbol{u}} \sim \psi_{ev}+\psi_{cv}+\psi_{ac}\cdot\psi_{ev}+\psi_{ac}\cdot\psi_{cv}+\psi_{ce}\cdot\psi_{cv}$	0.248365	0.69098	51167
239	$\overline{\boldsymbol{u}} \sim \psi_{ce}+\psi_{ev}+\psi_{cv}+\psi_{ac}\cdot\psi_{cv}+\psi_{ev}\cdot\psi_{cv}$	0.187407	0.69097	51168.3

240	$\overline{\boldsymbol{u}} \sim \psi_{ce}+\psi_{ev}+\psi_{cv}+\psi_{ac}\cdot\psi_{cv}+\psi_{ce}\cdot\psi_{ev}$	0.187404	0.69096	51168.8
241	$\overline{\boldsymbol{u}} \sim \psi_{ce}+\psi_{ev}+\psi_{cv}+\psi_{ac}\cdot\psi_{cv}+\psi_{ce}\cdot\psi_{cv}$	0.188501	0.69096	51168.6
242	$\overline{\boldsymbol{u}} \sim \psi_{ce}+\psi_{ev}+\psi_{cv}+\psi_{ac}\cdot\psi_{cv}$	0.187375	0.69093	51171.1
243	$\overline{\boldsymbol{u}} \sim \psi_{ce}+\psi_{cv}+\psi_{ac}\cdot\psi_{ce}+\psi_{ac}\cdot\psi_{ev}+\psi_{ac}\cdot\psi_{cv}+\psi_{ce}\cdot\psi_{ev}+\psi_{ce}\cdot\psi_{cv}$	0.116045	0.69083	51184.1
244	$\overline{\boldsymbol{u}} \sim \psi_{ce}+\psi_{cv}+\psi_{ac}\cdot\psi_{ce}+\psi_{ac}\cdot\psi_{cv}+\psi_{ce}\cdot\psi_{ev}+\psi_{ce}\cdot\psi_{cv}$	0.115708	0.69077	51188.7
245	$\overline{\boldsymbol{u}} \sim \psi_{ev}+\psi_{cv}+\psi_{ac}\cdot\psi_{ev}+\psi_{ac}\cdot\psi_{cv}+\psi_{ce}\cdot\psi_{ev}+\psi_{ev}\cdot\psi_{cv}$	0.231779	0.68937	51324.9
246	$\overline{\boldsymbol{u}} \sim \psi_{ev}+\psi_{cv}+\psi_{ac}\cdot\psi_{ev}+\psi_{ac}\cdot\psi_{cv}+\psi_{ce}\cdot\psi_{ev}$	0.227716	0.68896	51363.8
247	$\overline{\boldsymbol{u}} \sim \psi_{ce}+\psi_{cv}+\psi_{ac}\cdot\psi_{ce}+\psi_{ac}\cdot\psi_{ev}+\psi_{ac}\cdot\psi_{cv}+\psi_{ce}\cdot\psi_{ev}$	0.115869	0.68825	51434.3
248	$\overline{\boldsymbol{u}} \sim \psi_{ce}+\psi_{cv}+\psi_{ac}\cdot\psi_{ce}+\psi_{ac}\cdot\psi_{cv}+\psi_{ce}\cdot\psi_{ev}$	0.115391	0.6881	51447.2
249	$\overline{\boldsymbol{u}} \sim \psi_{ce}+\psi_{ev}+\psi_{cv}+\psi_{ac}\cdot\psi_{ce}+\psi_{ac}\cdot\psi_{ev}+\psi_{ev}\cdot\psi_{cv}$	0.122264	0.68778	51479.3
250	$\overline{\boldsymbol{u}} \sim \psi_{ev}+\psi_{cv}+\psi_{ac}\cdot\psi_{cv}+\psi_{ce}\cdot\psi_{ev}+\psi_{ce}\cdot\psi_{cv}+\psi_{ev}\cdot\psi_{cv}$	0.193114	0.68774	51484
251	$\overline{\boldsymbol{u}} \sim \psi_{ev}+\psi_{cv}+\psi_{ac}\cdot\psi_{cv}+\psi_{ce}\cdot\psi_{ev}+\psi_{ce}\cdot\psi_{cv}$	0.193065	0.68771	51485.5
252	$\overline{\boldsymbol{u}} \sim \psi_{ac}+\psi_{cv}+\psi_{ac}\cdot\psi_{ev}+\psi_{ac}\cdot\psi_{cv}+\psi_{ce}\cdot\psi_{ev}+\psi_{ce}\cdot\psi_{cv}+\psi_{ev}\cdot\psi_{cv}$	0.29649	0.68709	51547.1
253	$\overline{\boldsymbol{u}} \sim \psi_{ev}+\psi_{cv}+\psi_{ac}\cdot\psi_{ce}+\psi_{ac}\cdot\psi_{cv}+\psi_{ce}\cdot\psi_{ev}$	0.163641	0.68705	51549.6
254	$\overline{\boldsymbol{u}} \sim \psi_{ev}+\psi_{cv}+\psi_{ac}\cdot\psi_{ce}+\psi_{ac}\cdot\psi_{cv}+\psi_{ce}\cdot\psi_{ev}+\psi_{ev}\cdot\psi_{cv}$	0.163687	0.68705	51550.7
255	$\overline{\boldsymbol{u}} \sim \psi_{ac}+\psi_{cv}+\psi_{ac}\cdot\psi_{ev}+\psi_{ac}\cdot\psi_{cv}+\psi_{ce}\cdot\psi_{ev}+\psi_{ce}\cdot\psi_{cv}$	0.297329	0.68687	51568.1
256	$\overline{\boldsymbol{u}} \sim \psi_{ac}+\psi_{cv}+\psi_{ac}\cdot\psi_{ce}+\psi_{ac}\cdot\psi_{ev}+\psi_{ac}\cdot\psi_{cv}+\psi_{ce}\cdot\psi_{ev}+\psi_{ev}\cdot\psi_{cv}$	0.237025	0.6862	51633.7
257	$\overline{\boldsymbol{u}} \sim \psi_{ce}+\psi_{ev}+\psi_{cv}+\psi_{ac}\cdot\psi_{ce}+\psi_{ac}\cdot\psi_{ev}+\psi_{ce}\cdot\psi_{ev}+\psi_{ce}\cdot\psi_{cv}$	0.115651	0.68613	51640.5
258	$\overline{\boldsymbol{u}} \sim \psi_{ac}+\psi_{cv}+\psi_{ac}\cdot\psi_{ce}+\psi_{ac}\cdot\psi_{ev}+\psi_{ac}\cdot\psi_{cv}+\psi_{ce}\cdot\psi_{ev}$	0.237384	0.6861	51641.6
259	$\overline{\boldsymbol{u}} \sim \psi_{ac}+\psi_{cv}+\psi_{ac}\cdot\psi_{ev}+\psi_{ac}\cdot\psi_{cv}+\psi_{ce}\cdot\psi_{cv}+\psi_{ev}\cdot\psi_{cv}$	0.294932	0.686	51651.6
260	$\overline{\boldsymbol{u}} \sim \psi_{ac}+\psi_{cv}+\psi_{ac}\cdot\psi_{ev}+\psi_{ac}\cdot\psi_{cv}+\psi_{ce}\cdot\psi_{cv}$	0.29572	0.68572	51677.3
261	$\overline{\boldsymbol{u}} \sim \psi_{ev}+\psi_{cv}+\psi_{ac}\cdot\psi_{cv}+\psi_{ce}\cdot\psi_{cv}+\psi_{ev}\cdot\psi_{cv}$	0.196964	0.68568	51681.2
262	$\overline{\boldsymbol{u}} \sim \psi_{ev}+\psi_{cv}+\psi_{ac}\cdot\psi_{cv}+\psi_{ce}\cdot\psi_{cv}$	0.196928	0.68564	51684.4
263	$\overline{\boldsymbol{u}} \sim \psi_{ev}+\psi_{cv}+\psi_{ac}\cdot\psi_{cv}+\psi_{ce}\cdot\psi_{ev}+\psi_{ev}\cdot\psi_{cv}$	0.18439	0.68415	51828.9
264	$\overline{\boldsymbol{u}} \sim \psi_{ev}+\psi_{cv}+\psi_{ac}\cdot\psi_{cv}+\psi_{ce}\cdot\psi_{ev}$	0.184366	0.68414	51828.9
265	$\overline{\boldsymbol{u}} \sim \psi_{ac}+\psi_{ce}+\psi_{cv}+\psi_{ac}\cdot\psi_{cv}+\psi_{ce}\cdot\psi_{ev}+\psi_{ev}\cdot\psi_{cv}$	0.252813	0.68335	51906.3
266	$\overline{\boldsymbol{u}} \sim \psi_{ac}+\psi_{ce}+\psi_{cv}+\psi_{ac}\cdot\psi_{cv}+\psi_{ce}\cdot\psi_{ev}+\psi_{ce}\cdot\psi_{cv}+\psi_{ev}\cdot\psi_{cv}$	0.253415	0.68334	51907.6

267	$\overline{\boldsymbol{u}} \sim \psi_{ce}+\psi_{ev}+\psi_{cv}+\psi_{ac}\cdot\psi_{ce}+\psi_{ac}\cdot\psi_{ev}+\psi_{ce}\cdot\psi_{cv}$	0.116492	0.68316	51924.5
268	$\overline{\boldsymbol{u}} \sim \psi_{ac}+\psi_{cv}+\psi_{ac}\cdot\psi_{ce}+\psi_{ac}\cdot\psi_{cv}+\psi_{ce}\cdot\psi_{ev}+\psi_{ce}\cdot\psi_{cv}+\psi_{ev}\cdot\psi_{cv}$	0.200566	0.6815	52083.5
269	$\overline{\boldsymbol{u}} \sim \psi_{ac}+\psi_{cv}+\psi_{ac}\cdot\psi_{ev}+\psi_{ac}\cdot\psi_{cv}+\psi_{ce}\cdot\psi_{ev}+\psi_{ev}\cdot\psi_{cv}$	0.270369	0.68086	52143.2
270	$\overline{\boldsymbol{u}} \sim \psi_{ac}+\psi_{cv}+\psi_{ac}\cdot\psi_{ev}+\psi_{ac}\cdot\psi_{cv}+\psi_{ce}\cdot\psi_{ev}$	0.27111	0.68071	52156.4
271	$\overline{\boldsymbol{u}} \sim \psi_{ac}+\psi_{cv}+\psi_{ac}\cdot\psi_{ce}+\psi_{ac}\cdot\psi_{cv}+\psi_{ce}\cdot\psi_{cv}+\psi_{ev}\cdot\psi_{cv}$	0.209413	0.68009	52216.2
272	$\overline{\boldsymbol{u}} \sim \psi_{ce}+\psi_{ev}+\psi_{ac}\cdot\psi_{ce}+\psi_{ac}\cdot\psi_{ev}+\psi_{ce}\cdot\psi_{ev}+\psi_{ev}\cdot\psi_{cv}$	0.123216	0.67812	52402.2
273	$\overline{\boldsymbol{u}} \sim \psi_{ce}+\psi_{cv}+\psi_{ac}\cdot\psi_{ev}+\psi_{ac}\cdot\psi_{cv}+\psi_{ce}\cdot\psi_{ev}+\psi_{ce}\cdot\psi_{cv}+\psi_{ev}\cdot\psi_{cv}$	0.178704	0.67598	52603.7
274	$\overline{\boldsymbol{u}} \sim \psi_{ce}+\psi_{cv}+\psi_{ac}\cdot\psi_{ev}+\psi_{ac}\cdot\psi_{cv}+\psi_{ce}\cdot\psi_{ev}+\psi_{ev}\cdot\psi_{cv}$	0.177861	0.67595	52605.3
275	$\overline{\boldsymbol{u}} \sim \psi_{ce}+\psi_{cv}+\psi_{ac}\cdot\psi_{cv}+\psi_{ce}\cdot\psi_{ev}+\psi_{ce}\cdot\psi_{cv}+\psi_{ev}\cdot\psi_{cv}$	0.180722	0.67593	52606.6
276	$\overline{\boldsymbol{u}} \sim \psi_{ce}+\psi_{cv}+\psi_{ac}\cdot\psi_{cv}+\psi_{ce}\cdot\psi_{ev}+\psi_{ev}\cdot\psi_{cv}$	0.179829	0.67591	52607.8
277	$\overline{\boldsymbol{u}} \sim \psi_{ce}+\psi_{ev}+\psi_{ac}\cdot\psi_{ce}+\psi_{ac}\cdot\psi_{ev}+\psi_{ev}\cdot\psi_{cv}$	0.124058	0.67496	52696
278	$\overline{\boldsymbol{u}} \sim \psi_{ac}+\psi_{ev}+\psi_{cv}+\psi_{ac}\cdot\psi_{ce}+\psi_{ac}\cdot\psi_{ev}+\psi_{ac}\cdot\psi_{cv}+\psi_{ev}\cdot\psi_{cv}$	0.303303	0.67428	52761.4
279	$\overline{\boldsymbol{u}} \sim \psi_{ac}+\psi_{ev}+\psi_{cv}+\psi_{ac}\cdot\psi_{ce}+\psi_{ac}\cdot\psi_{cv}+\psi_{ev}\cdot\psi_{cv}$	0.303452	0.67427	52761.9
280	$\overline{\boldsymbol{u}} \sim \psi_{ac}+\psi_{ev}+\psi_{cv}+\psi_{ac}\cdot\psi_{ce}+\psi_{ac}\cdot\psi_{cv}$	0.303401	0.67423	52764.6
281	$\overline{\boldsymbol{u}} \sim \psi_{ac}+\psi_{ev}+\psi_{cv}+\psi_{ac}\cdot\psi_{ce}+\psi_{ac}\cdot\psi_{ev}+\psi_{ac}\cdot\psi_{cv}$	0.303338	0.67422	52766.1
282	$\overline{\boldsymbol{u}} \sim \psi_{ev}+\psi_{cv}+\psi_{ac}\cdot\psi_{ce}+\psi_{ac}\cdot\psi_{ev}+\psi_{ac}\cdot\psi_{cv}+\psi_{ev}\cdot\psi_{cv}$	0.255584	0.67274	52903.6
283	$\overline{\boldsymbol{u}} \sim \psi_{ev}+\psi_{cv}+\psi_{ac}\cdot\psi_{ce}+\psi_{ac}\cdot\psi_{ev}+\psi_{ac}\cdot\psi_{cv}$	0.25079	0.67236	52937.6
284	$\overline{\boldsymbol{u}} \sim \psi_{ac}+\psi_{ce}+\psi_{cv}+\psi_{ac}\cdot\psi_{cv}+\psi_{ev}\cdot\psi_{cv}$	0.234334	0.67085	53076.9
285	$\overline{\boldsymbol{u}} \sim \psi_{ac}+\psi_{ce}+\psi_{cv}+\psi_{ac}\cdot\psi_{cv}+\psi_{ce}\cdot\psi_{cv}+\psi_{ev}\cdot\psi_{cv}$	0.234605	0.67084	53078.7
286	$\overline{\boldsymbol{u}} \sim \psi_{ev}+\psi_{cv}+\psi_{ac}\cdot\psi_{ce}+\psi_{ac}\cdot\psi_{cv}+\psi_{ev}\cdot\psi_{cv}$	0.212099	0.66986	53167.7
287	$\overline{\boldsymbol{u}} \sim \psi_{ev}+\psi_{cv}+\psi_{ac}\cdot\psi_{ce}+\psi_{ac}\cdot\psi_{cv}$	0.212064	0.66982	53170.3
288	$\overline{\boldsymbol{u}} \sim \psi_{ce}+\psi_{ev}+\psi_{cv}+\psi_{ac}\cdot\psi_{ce}+\psi_{ac}\cdot\psi_{ev}+\psi_{ce}\cdot\psi_{ev}$	0.120214	0.66779	53357.9
289	$\overline{\boldsymbol{u}} \sim \psi_{ce}+\psi_{cv}+\psi_{ac}\cdot\psi_{ev}+\psi_{ac}\cdot\psi_{cv}+\psi_{ce}\cdot\psi_{cv}+\psi_{ev}\cdot\psi_{cv}$	0.165506	0.66752	53382.2
290	$\overline{\boldsymbol{u}} \sim \psi_{ce}+\psi_{cv}+\psi_{ac}\cdot\psi_{ev}+\psi_{ac}\cdot\psi_{cv}+\psi_{ev}\cdot\psi_{cv}$	0.164496	0.66746	53386.5
291	$\overline{\boldsymbol{u}} \sim \psi_{ce}+\psi_{ev}+\psi_{ac}\cdot\psi_{ce}+\psi_{ac}\cdot\psi_{ev}+\psi_{ac}\cdot\psi_{cv}+\psi_{ce}\cdot\psi_{ev}+\psi_{ce}\cdot\psi_{cv}$	0.113343	0.66726	53407
292	$\overline{\boldsymbol{u}} \sim \psi_{cv}+\psi_{ac}\cdot\psi_{ce}+\psi_{ac}\cdot\psi_{ev}+\psi_{ac}\cdot\psi_{cv}+\psi_{ce}\cdot\psi_{cv}+\psi_{ev}\cdot\psi_{cv}$	0.159668	0.66663	53462.8
293	$\overline{\boldsymbol{u}} \sim \psi_{cv}+\psi_{ac}\cdot\psi_{ce}+\psi_{ac}\cdot\psi_{ev}+\psi_{ac}\cdot\psi_{cv}+\psi_{ce}\cdot\psi_{ev}+\psi_{ce}\cdot\psi_{cv}+\psi_{ev}\cdot\psi_{cv}$	0.159467	0.66662	53464.7

294	$\overline{\boldsymbol{u}} \sim \psi_{ac}+\psi_{cv}+\psi_{ac}\cdot\psi_{ce}+\psi_{ac}\cdot\psi_{ev}+\psi_{ac}\cdot\psi_{cv}+\psi_{ev}\cdot\psi_{cv}$	0.282365	0.66619	53502.7
295	$\overline{\boldsymbol{u}} \sim \psi_{ac}+\psi_{cv}+\psi_{ac}\cdot\psi_{ce}+\psi_{ac}\cdot\psi_{ev}+\psi_{ac}\cdot\psi_{cv}$	0.28311	0.66594	53524.6
296	$\overline{\boldsymbol{u}} \sim \psi_{ac}+\psi_{cv}+\psi_{ac}\cdot\psi_{cv}+\psi_{ce}\cdot\psi_{ev}+\psi_{ce}\cdot\psi_{cv}+\psi_{ev}\cdot\psi_{cv}$	0.242205	0.66584	53534.3
297	$\overline{\boldsymbol{u}} \sim \psi_{cv}+\psi_{ac}\cdot\psi_{ce}+\psi_{ac}\cdot\psi_{cv}+\psi_{ce}\cdot\psi_{ev}+\psi_{ce}\cdot\psi_{cv}+\psi_{ev}\cdot\psi_{cv}$	0.164672	0.6652	53592.1
298	$\overline{\boldsymbol{u}} \sim \psi_{cv}+\psi_{ac}\cdot\psi_{ce}+\psi_{ac}\cdot\psi_{cv}+\psi_{ce}\cdot\psi_{cv}+\psi_{ev}\cdot\psi_{cv}$	0.163302	0.66517	53594.5
299	$\overline{\boldsymbol{u}} \sim \psi_{ce}+\psi_{cv}+\psi_{ac}\cdot\psi_{ev}+\psi_{ac}\cdot\psi_{cv}+\psi_{ce}\cdot\psi_{ev}+\psi_{ce}\cdot\psi_{cv}$	0.158958	0.66512	53600
300	$\overline{\boldsymbol{u}} \sim \psi_{ce}+\psi_{cv}+\psi_{ac}\cdot\psi_{ev}+\psi_{ac}\cdot\psi_{cv}+\psi_{ce}\cdot\psi_{ev}$	0.157862	0.66504	53606
301	$\overline{\boldsymbol{u}} \sim \psi_{ce}+\psi_{ev}+\psi_{cv}+\psi_{ac}\cdot\psi_{ce}+\psi_{ac}\cdot\psi_{ev}$	0.12094	0.6649	53618.4
302	$\overline{\boldsymbol{u}} \sim \psi_{ce}+\psi_{ev}+\psi_{ac}\cdot\psi_{ce}+\psi_{ac}\cdot\psi_{ev}+\psi_{ac}\cdot\psi_{cv}+\psi_{ce}\cdot\psi_{ev}$	0.112442	0.66473	53634.8
303	$\overline{\boldsymbol{u}} \sim \psi_{ce}+\psi_{ev}+\psi_{ac}\cdot\psi_{ce}+\psi_{ac}\cdot\psi_{ev}+\psi_{ac}\cdot\psi_{cv}+\psi_{ce}\cdot\psi_{cv}$	0.113837	0.66463	53644
304	$\overline{\boldsymbol{u}} \sim \psi_{ce}+\psi_{cv}+\psi_{ac}\cdot\psi_{cv}+\psi_{ce}\cdot\psi_{cv}+\psi_{ev}\cdot\psi_{cv}$	0.176864	0.66423	53679.5
305	$\overline{\boldsymbol{u}} \sim \psi_{ce}+\psi_{cv}+\psi_{ac}\cdot\psi_{cv}+\psi_{ev}\cdot\psi_{cv}$	0.176101	0.66421	53680
306	$\overline{\boldsymbol{u}} \sim \psi_{ac}+\psi_{cv}+\psi_{ac}\cdot\psi_{cv}+\psi_{ce}\cdot\psi_{cv}+\psi_{ev}\cdot\psi_{cv}$	0.237103	0.66418	53684
307	$\overline{\boldsymbol{u}} \sim \psi_{ce}+\psi_{ev}+\psi_{ac}\cdot\psi_{ce}+\psi_{ac}\cdot\psi_{ev}+\psi_{ce}\cdot\psi_{ev}+\psi_{ce}\cdot\psi_{cv}$	0.115314	0.66335	53759.7
308	$\overline{\boldsymbol{u}} \sim \psi_{ce}+\psi_{ev}+\psi_{ac}\cdot\psi_{ce}+\psi_{ac}\cdot\psi_{ev}+\psi_{ce}\cdot\psi_{ev}$	0.115813	0.66332	53761.2
309	$\overline{\boldsymbol{u}} \sim \psi_{cv}+\psi_{ac}\cdot\psi_{ev}+\psi_{ac}\cdot\psi_{cv}+\psi_{ce}\cdot\psi_{ev}+\psi_{ce}\cdot\psi_{cv}+\psi_{ev}\cdot\psi_{cv}$	0.174948	0.66323	53769.9
310	$\overline{\boldsymbol{u}} \sim \psi_{cv}+\psi_{ac}\cdot\psi_{ev}+\psi_{ac}\cdot\psi_{cv}+\psi_{ce}\cdot\psi_{cv}+\psi_{ev}\cdot\psi_{cv}$	0.171294	0.6627	53816.4
311	$\overline{\boldsymbol{u}} \sim \psi_{ce}+\psi_{ev}+\psi_{ac}\cdot\psi_{ce}+\psi_{ac}\cdot\psi_{ev}+\psi_{ac}\cdot\psi_{cv}$	0.113052	0.6619	53888
312	$\overline{\boldsymbol{u}} \sim \psi_{ac}+\psi_{ce}+\psi_{cv}+\psi_{ac}\cdot\psi_{cv}+\psi_{ce}\cdot\psi_{ev}$	0.217956	0.66113	53957.3
313	$\overline{\boldsymbol{u}} \sim \psi_{ac}+\psi_{ce}+\psi_{cv}+\psi_{ac}\cdot\psi_{cv}+\psi_{ce}\cdot\psi_{ev}+\psi_{ce}\cdot\psi_{cv}$	0.217909	0.66112	53959.3
314	$\overline{\boldsymbol{u}} \sim \psi_{cv}+\psi_{ac}\cdot\psi_{cv}+\psi_{ce}\cdot\psi_{ev}+\psi_{ce}\cdot\psi_{cv}+\psi_{ev}\cdot\psi_{cv}$	0.188657	0.6606	54004.3
315	$\overline{\boldsymbol{u}} \sim \psi_{ce}+\psi_{ev}+\psi_{ac}\cdot\psi_{ce}+\psi_{ac}\cdot\psi_{ev}+\psi_{ce}\cdot\psi_{cv}$	0.116076	0.66044	54018.3
316	$\overline{\boldsymbol{u}} \sim \psi_{ce}+\psi_{ev}+\psi_{ac}\cdot\psi_{ce}+\psi_{ac}\cdot\psi_{ev}$	0.11655	0.66042	54019.4
317	$\overline{\boldsymbol{u}} \sim \psi_{ce}+\psi_{cv}+\psi_{ac}\cdot\psi_{ce}+\psi_{ac}\cdot\psi_{ev}+\psi_{ac}\cdot\psi_{cv}+\psi_{ce}\cdot\psi_{cv}$	0.119254	0.65934	54117.6
318	$\overline{\boldsymbol{u}} \sim \psi_{cv}+\psi_{ac}\cdot\psi_{cv}+\psi_{ce}\cdot\psi_{cv}+\psi_{ev}\cdot\psi_{cv}$	0.184683	0.65877	54166
319	$\overline{\boldsymbol{u}} \sim \psi_{ce}+\psi_{ac}\cdot\psi_{ce}+\psi_{ac}\cdot\psi_{ev}+\psi_{ac}\cdot\psi_{cv}+\psi_{ce}\cdot\psi_{ev}+\psi_{ce}\cdot\psi_{cv}+\psi_{ev}\cdot\psi_{cv}$	0.0820262	0.65858	54185.6
320	$\overline{\boldsymbol{u}} \sim \psi_{ce}+\psi_{ac}\cdot\psi_{ce}+\psi_{ac}\cdot\psi_{ev}+\psi_{ac}\cdot\psi_{cv}+\psi_{ce}\cdot\psi_{ev}+\psi_{ev}\cdot\psi_{cv}$	0.0817705	0.65857	54185.6

321	$\overline{\boldsymbol{u}} \sim \psi_{ce}+\psi_{cv}+\psi_{ac}\cdot\psi_{ce}+\psi_{ac}\cdot\psi_{ev}+\psi_{ac}\cdot\psi_{cv}$	0.118745	0.6579	54244.6
322	$\overline{\boldsymbol{u}} \sim \psi_{ac}+\psi_{ev}+\psi_{cv}+\psi_{ac}\cdot\psi_{ev}+\psi_{ac}\cdot\psi_{cv}+\psi_{ev}\cdot\psi_{cv}$	0.311262	0.65768	54264.6
323	$\overline{\boldsymbol{u}} \sim \psi_{ac}+\psi_{ev}+\psi_{cv}+\psi_{ac}\cdot\psi_{cv}+\psi_{ev}\cdot\psi_{cv}$	0.311481	0.65766	54265.3
324	$\overline{\boldsymbol{u}} \sim \psi_{ac}+\psi_{ev}+\psi_{cv}+\psi_{ac}\cdot\psi_{cv}$	0.311418	0.65762	54267.7
325	$\overline{\boldsymbol{u}} \sim \psi_{ac}+\psi_{ev}+\psi_{cv}+\psi_{ac}\cdot\psi_{ev}+\psi_{ac}\cdot\psi_{cv}$	0.31132	0.65762	54269.2
326	$\overline{\boldsymbol{u}} \sim \psi_{ce}+\psi_{cv}+\psi_{ac}\cdot\psi_{cv}+\psi_{ce}\cdot\psi_{ev}+\psi_{ce}\cdot\psi_{cv}$	0.172062	0.65525	54478
327	$\overline{\boldsymbol{u}} \sim \psi_{ce}+\psi_{cv}+\psi_{ac}\cdot\psi_{cv}+\psi_{ce}\cdot\psi_{ev}$	0.171498	0.65524	54477.4
328	$\overline{\boldsymbol{u}} \sim \psi_{ev}+\psi_{cv}+\psi_{ac}\cdot\psi_{ev}+\psi_{ac}\cdot\psi_{cv}+\psi_{ev}\cdot\psi_{cv}$	0.235391	0.6551	54491.1
329	$\overline{\boldsymbol{u}} \sim \psi_{ev}+\psi_{cv}+\psi_{ac}\cdot\psi_{ev}+\psi_{ac}\cdot\psi_{cv}$	0.229881	0.65458	54535.1
330	$\overline{\boldsymbol{u}} \sim \psi_{cv}+\psi_{ac}\cdot\psi_{ce}+\psi_{ac}\cdot\psi_{ev}+\psi_{ac}\cdot\psi_{cv}+\psi_{ce}\cdot\psi_{ev}+\psi_{ev}\cdot\psi_{cv}$	0.167346	0.6506	54884.5
331	$\overline{\boldsymbol{u}} \sim \psi_{ev}+\psi_{cv}+\psi_{ac}\cdot\psi_{cv}+\psi_{ev}\cdot\psi_{cv}$	0.183594	0.6504	54899.2
332	$\overline{\boldsymbol{u}} \sim \psi_{ev}+\psi_{cv}+\psi_{ac}\cdot\psi_{cv}$	0.183561	0.65036	54901.6
333	$\overline{\boldsymbol{u}} \sim \psi_{ce}+\psi_{cv}+\psi_{ac}\cdot\psi_{ev}+\psi_{ac}\cdot\psi_{cv}+\psi_{ce}\cdot\psi_{cv}$	0.143247	0.65034	54905.5
334	$\overline{\boldsymbol{u}} \sim \psi_{ce}+\psi_{cv}+\psi_{ac}\cdot\psi_{ev}+\psi_{ac}\cdot\psi_{cv}$	0.141791	0.65015	54921.1
335	$\overline{\boldsymbol{u}} \sim \psi_{ac}+\psi_{cv}+\psi_{ac}\cdot\psi_{ev}+\psi_{ac}\cdot\psi_{cv}+\psi_{ev}\cdot\psi_{cv}$	0.279585	0.64953	54975.9
336	$\overline{\boldsymbol{u}} \sim \psi_{cv}+\psi_{ac}\cdot\psi_{ce}+\psi_{ac}\cdot\psi_{ev}+\psi_{ac}\cdot\psi_{cv}+\psi_{ev}\cdot\psi_{cv}$	0.177601	0.64939	54987.7
337	$\overline{\boldsymbol{u}} \sim \psi_{cv}+\psi_{ac}\cdot\psi_{ce}+\psi_{ac}\cdot\psi_{ev}+\psi_{ac}\cdot\psi_{cv}+\psi_{ce}\cdot\psi_{ev}+\psi_{ce}\cdot\psi_{cv}$	0.153603	0.64933	54994.3
338	$\overline{\boldsymbol{u}} \sim \psi_{ac}+\psi_{cv}+\psi_{ac}\cdot\psi_{ev}+\psi_{ac}\cdot\psi_{cv}$	0.280573	0.64927	54997.1
339	$\overline{\boldsymbol{u}} \sim \psi_{cv}+\psi_{ac}\cdot\psi_{ev}+\psi_{ac}\cdot\psi_{cv}+\psi_{ce}\cdot\psi_{ev}+\psi_{ce}\cdot\psi_{cv}$	0.155639	0.64927	54998.3
340	$\overline{\boldsymbol{u}} \sim \psi_{cv}+\psi_{ac}\cdot\psi_{ev}+\psi_{ac}\cdot\psi_{cv}+\psi_{ce}\cdot\psi_{ev}+\psi_{ev}\cdot\psi_{cv}$	0.154077	0.64843	55070.6
341	$\overline{\boldsymbol{u}} \sim \psi_{cv}+\psi_{ac}\cdot\psi_{ce}+\psi_{ac}\cdot\psi_{ev}+\psi_{ac}\cdot\psi_{cv}+\psi_{ce}\cdot\psi_{cv}$	0.144411	0.64708	55186.4
342	$\overline{\boldsymbol{u}} \sim \psi_{cv}+\psi_{ac}\cdot\psi_{ev}+\psi_{ac}\cdot\psi_{cv}+\psi_{ce}\cdot\psi_{cv}$	0.148073	0.64661	55225.7
343	$\overline{\boldsymbol{u}} \sim \psi_{ac}+\psi_{cv}+\psi_{ac}\cdot\psi_{ce}+\psi_{ac}\cdot\psi_{cv}+\psi_{ce}\cdot\psi_{ev}+\psi_{ev}\cdot\psi_{cv}$	0.220402	0.64584	55293.9
344	$\overline{\boldsymbol{u}} \sim \psi_{ac}+\psi_{cv}+\psi_{ac}\cdot\psi_{cv}+\psi_{ce}\cdot\psi_{ev}+\psi_{ev}\cdot\psi_{cv}$	0.211352	0.64498	55365.6
345	$\overline{\boldsymbol{u}} \sim \psi_{ac}+\psi_{cv}+\psi_{ac}\cdot\psi_{ce}+\psi_{ac}\cdot\psi_{cv}+\psi_{ev}\cdot\psi_{cv}$	0.230742	0.6445	55407.2
346	$\overline{\boldsymbol{u}} \sim \psi_{cv}+\psi_{ac}\cdot\psi_{ce}+\psi_{ac}\cdot\psi_{cv}+\psi_{ce}\cdot\psi_{ev}+\psi_{ev}\cdot\psi_{cv}$	0.189465	0.64287	55545
347	$\overline{\boldsymbol{u}} \sim \psi_{cv}+\psi_{ac}\cdot\psi_{ce}+\psi_{ac}\cdot\psi_{cv}+\psi_{ev}\cdot\psi_{cv}$	0.196758	0.64245	55579.5

348	$\overline{\boldsymbol{u}} \sim \psi_{cv}+\psi_{ac}\cdot\psi_{cv}+\psi_{ce}\cdot\psi_{ev}+\psi_{ev}\cdot\psi_{cv}$	0.170599	0.64012	55776
349	$\overline{\boldsymbol{u}} \sim \psi_{cv}+\psi_{ac}\cdot\psi_{ce}+\psi_{ac}\cdot\psi_{ev}+\psi_{ac}\cdot\psi_{cv}+\psi_{ce}\cdot\psi_{ev}$	0.159492	0.63805	55950.9
350	$\overline{\boldsymbol{u}} \sim \psi_{cv}+\psi_{ac}\cdot\psi_{ce}+\psi_{ac}\cdot\psi_{ev}+\psi_{ac}\cdot\psi_{cv}$	0.156659	0.63795	55958
351	$\overline{\boldsymbol{u}} \sim \psi_{ac}+\psi_{ev}+\psi_{cv}+\psi_{ac}\cdot\psi_{ce}+\psi_{ac}\cdot\psi_{ev}+\psi_{ce}\cdot\psi_{ev}+\psi_{ce}\cdot\psi_{cv}+\psi_{ev}\cdot\psi_{cv}$	0.0770629	0.63195	56460
352	$\overline{\boldsymbol{u}} \sim \psi_{ac}+\psi_{ev}+\psi_{cv}+\psi_{ac}\cdot\psi_{ce}+\psi_{ac}\cdot\psi_{ev}+\psi_{ce}\cdot\psi_{ev}+\psi_{ce}\cdot\psi_{cv}$	0.0772698	0.6317	56479
353	$\overline{\boldsymbol{u}} \sim \psi_{ac}+\psi_{ev}+\psi_{cv}+\psi_{ac}\cdot\psi_{ce}+\psi_{ce}\cdot\psi_{ev}+\psi_{ce}\cdot\psi_{cv}+\psi_{ev}\cdot\psi_{cv}$	0.077349	0.63108	56530.3
354	$\overline{\boldsymbol{u}} \sim \psi_{ac}+\psi_{ev}+\psi_{cv}+\psi_{ac}\cdot\psi_{ce}+\psi_{ce}\cdot\psi_{ev}+\psi_{ce}\cdot\psi_{cv}$	0.077398	0.63106	56530.4
355	$\overline{\boldsymbol{u}} \sim \psi_{cv}+\psi_{ac}\cdot\psi_{ev}+\psi_{ac}\cdot\psi_{cv}+\psi_{ev}\cdot\psi_{cv}$	0.158153	0.62841	56745.4
356	$\overline{\boldsymbol{u}} \sim \psi_{cv}+\psi_{ac}\cdot\psi_{ev}+\psi_{ac}\cdot\psi_{cv}+\psi_{ce}\cdot\psi_{ev}$	0.133807	0.62832	56752.2
357	$\overline{\boldsymbol{u}} \sim \psi_{ac}+\psi_{cv}+\psi_{ac}\cdot\psi_{cv}+\psi_{ev}\cdot\psi_{cv}$	0.22114	0.62818	56764.3
358	$\overline{\boldsymbol{u}} \sim \psi_{cv}+\psi_{ac}\cdot\psi_{cv}+\psi_{ev}\cdot\psi_{cv}$	0.172891	0.62385	57113.2
359	$\overline{\boldsymbol{u}} \sim \psi_{ce}+\psi_{ac}\cdot\psi_{ce}+\psi_{ac}\cdot\psi_{ev}+\psi_{ac}\cdot\psi_{cv}+\psi_{ce}\cdot\psi_{cv}+\psi_{ev}\cdot\psi_{cv}$	0.0818611	0.61703	57659.9
360	$\overline{\boldsymbol{u}} \sim \psi_{ce}+\psi_{ac}\cdot\psi_{ce}+\psi_{ac}\cdot\psi_{ev}+\psi_{ac}\cdot\psi_{cv}+\psi_{ev}\cdot\psi_{cv}$	0.080504	0.61626	57719.9
361	$\overline{\boldsymbol{u}} \sim \psi_{cv}+\psi_{ac}\cdot\psi_{ev}+\psi_{ac}\cdot\psi_{cv}$	0.136762	0.61371	57918.3
362	$\overline{\boldsymbol{u}} \sim \psi_{ac}+\psi_{cv}+\psi_{ac}\cdot\psi_{ce}+\psi_{ac}\cdot\psi_{cv}+\psi_{ce}\cdot\psi_{ev}+\psi_{ce}\cdot\psi_{cv}$	0.208818	0.60771	58387.6
363	$\overline{\boldsymbol{u}} \sim \psi_{ac}+\psi_{cv}+\psi_{ac}\cdot\psi_{cv}+\psi_{ce}\cdot\psi_{ev}+\psi_{ce}\cdot\psi_{cv}$	0.210757	0.60765	58391
364	$\overline{\boldsymbol{u}} \sim \psi_{ac}+\psi_{cv}+\psi_{ac}\cdot\psi_{ce}+\psi_{ac}\cdot\psi_{ev}+\psi_{ce}\cdot\psi_{ev}+\psi_{ce}\cdot\psi_{cv}+\psi_{ev}\cdot\psi_{cv}$	0.0881623	0.60708	58436.9
365	$\overline{\boldsymbol{u}} \sim \psi_{ac}+\psi_{cv}+\psi_{ac}\cdot\psi_{ce}+\psi_{ac}\cdot\psi_{ev}+\psi_{ce}\cdot\psi_{ev}+\psi_{ce}\cdot\psi_{cv}$	0.08835	0.60704	58439.4
366	$\overline{\boldsymbol{u}} \sim \psi_{cv}+\psi_{ac}\cdot\psi_{ce}+\psi_{ac}\cdot\psi_{cv}+\psi_{ce}\cdot\psi_{ev}+\psi_{ce}\cdot\psi_{cv}$	0.190753	0.60551	58555.7
367	$\overline{\boldsymbol{u}} \sim \psi_{cv}+\psi_{ac}\cdot\psi_{cv}+\psi_{ce}\cdot\psi_{ev}+\psi_{ce}\cdot\psi_{cv}$	0.185103	0.60528	58572.4
368	$\overline{\boldsymbol{u}} \sim \psi_{ac}+\psi_{ev}+\psi_{ac}\cdot\psi_{ce}+\psi_{ac}\cdot\psi_{ev}+\psi_{ac}\cdot\psi_{cv}+\psi_{ce}\cdot\psi_{ev}+\psi_{ce}\cdot\psi_{cv}+\psi_{ev}\cdot\psi_{cv}$	0.0901366	0.57911	60518.9
369	$\overline{\boldsymbol{u}} \sim \psi_{ac}+\psi_{ev}+\psi_{ac}\cdot\psi_{ce}+\psi_{ac}\cdot\psi_{cv}+\psi_{ce}\cdot\psi_{ev}+\psi_{ce}\cdot\psi_{cv}+\psi_{ev}\cdot\psi_{cv}$	0.0882023	0.57706	60664.8
370	$\overline{\boldsymbol{u}} \sim \psi_{ac}+\psi_{ev}+\psi_{ac}\cdot\psi_{ce}+\psi_{ac}\cdot\psi_{ev}+\psi_{ce}\cdot\psi_{ev}+\psi_{ce}\cdot\psi_{cv}+\psi_{ev}\cdot\psi_{cv}$	0.0814688	0.57671	60689.7
371	$\overline{\boldsymbol{u}} \sim \psi_{ac}+\psi_{ce}+\psi_{ev}+\psi_{ac}\cdot\psi_{ev}+\psi_{ac}\cdot\psi_{cv}+\psi_{ce}\cdot\psi_{ev}+\psi_{ce}\cdot\psi_{cv}+\psi_{ev}\cdot\psi_{cv}$	0.136249	0.57612	60732.8
372	$\overline{\boldsymbol{u}} \sim \psi_{ac}+\psi_{ev}+\psi_{ac}\cdot\psi_{ce}+\psi_{ce}\cdot\psi_{ev}+\psi_{ce}\cdot\psi_{cv}+\psi_{ev}\cdot\psi_{cv}$	0.081471	0.57551	60774.6
373	$\overline{\boldsymbol{u}} \sim \psi_{ac}+\psi_{ce}+\psi_{ev}+\psi_{ac}\cdot\psi_{ev}+\psi_{ac}\cdot\psi_{cv}+\psi_{ce}\cdot\psi_{cv}+\psi_{ev}\cdot\psi_{cv}$	0.135988	0.57541	60782.8
374	$\overline{\boldsymbol{u}} \sim \psi_{ce}+\psi_{ev}+\psi_{ac}\cdot\psi_{ev}+\psi_{ac}\cdot\psi_{cv}+\psi_{ce}\cdot\psi_{ev}+\psi_{ce}\cdot\psi_{cv}+\psi_{ev}\cdot\psi_{cv}$	0.132377	0.57355	60915

375	$\overline{\boldsymbol{u}} \sim \psi_{ac}+\psi_{ce}+\psi_{ac}\cdot\psi_{ev}+\psi_{ac}\cdot\psi_{cv}+\psi_{ce}\cdot\psi_{ev}+\psi_{ce}\cdot\psi_{cv}+\psi_{ev}\cdot\psi_{cv}$	0.135341	0.57331	60932.4
376	$\overline{\boldsymbol{u}} \sim \psi_{ce}+\psi_{ev}+\psi_{ac}\cdot\psi_{ev}+\psi_{ac}\cdot\psi_{cv}+\psi_{ce}\cdot\psi_{cv}+\psi_{ev}\cdot\psi_{cv}$	0.131871	0.57238	60997.3
377	$\overline{\boldsymbol{u}} \sim \psi_{ac}+\psi_{cv}+\psi_{ac}\cdot\psi_{ce}+\psi_{ac}\cdot\psi_{cv}+\psi_{ce}\cdot\psi_{ev}$	0.227184	0.57166	61047.1
378	$\overline{\boldsymbol{u}} \sim \psi_{ac}+\psi_{ce}+\psi_{ac}\cdot\psi_{ev}+\psi_{ac}\cdot\psi_{cv}+\psi_{ce}\cdot\psi_{cv}+\psi_{ev}\cdot\psi_{cv}$	0.135508	0.57146	61061.9
379	$\overline{\boldsymbol{u}} \sim \psi_{ac}+\psi_{ce}+\psi_{ev}+\psi_{ac}\cdot\psi_{cv}+\psi_{ce}\cdot\psi_{ev}+\psi_{ce}\cdot\psi_{cv}+\psi_{ev}\cdot\psi_{cv}$	0.134298	0.57129	61075.2
380	$\overline{\boldsymbol{u}} \sim \psi_{ac}+\psi_{ce}+\psi_{ac}\cdot\psi_{cv}+\psi_{ce}\cdot\psi_{ev}+\psi_{ce}\cdot\psi_{cv}+\psi_{ev}\cdot\psi_{cv}$	0.134055	0.57128	61074.9
381	$\overline{\boldsymbol{u}} \sim \psi_{ac}+\psi_{ce}+\psi_{ev}+\psi_{ac}\cdot\psi_{cv}+\psi_{ce}\cdot\psi_{cv}+\psi_{ev}\cdot\psi_{cv}$	0.134108	0.57101	61093.6
382	$\overline{\boldsymbol{u}} \sim \psi_{ac}+\psi_{ce}+\psi_{ac}\cdot\psi_{cv}+\psi_{ce}\cdot\psi_{cv}+\psi_{ev}\cdot\psi_{cv}$	0.134589	0.57095	61096.7
383	$\overline{\boldsymbol{u}} \sim \psi_{cv}+\psi_{ac}\cdot\psi_{ce}+\psi_{ac}\cdot\psi_{cv}+\psi_{ce}\cdot\psi_{ev}$	0.249884	0.57093	61097.2
384	$\overline{\boldsymbol{u}} \sim \psi_{ac}+\psi_{ev}+\psi_{ac}\cdot\psi_{ce}+\psi_{ac}\cdot\psi_{ev}+\psi_{ac}\cdot\psi_{cv}+\psi_{ce}\cdot\psi_{ev}+\psi_{ev}\cdot\psi_{cv}$	0.106299	0.56419	61572.1
385	$\overline{\boldsymbol{u}} \sim \psi_{ac}+\psi_{ac}\cdot\psi_{ce}+\psi_{ac}\cdot\psi_{ev}+\psi_{ac}\cdot\psi_{cv}+\psi_{ce}\cdot\psi_{ev}+\psi_{ce}\cdot\psi_{cv}+\psi_{ev}\cdot\psi_{cv}$	0.098963	0.56335	61630.2
386	$\overline{\boldsymbol{u}} \sim \psi_{ac}+\psi_{ev}+\psi_{ac}\cdot\psi_{ce}+\psi_{ac}\cdot\psi_{cv}+\psi_{ce}\cdot\psi_{ev}+\psi_{ev}\cdot\psi_{cv}$	0.104244	0.56257	61683.3
387	$\overline{\boldsymbol{u}} \sim \psi_{ac}+\psi_{ev}+\psi_{cv}+\psi_{ac}\cdot\psi_{ce}+\psi_{ce}\cdot\psi_{cv}+\psi_{ev}\cdot\psi_{cv}$	0.0985995	0.56084	61803
388	$\overline{\boldsymbol{u}} \sim \psi_{ac}+\psi_{ev}+\psi_{cv}+\psi_{ac}\cdot\psi_{ce}+\psi_{ce}\cdot\psi_{cv}$	0.0985928	0.56083	61802.5
389	$\overline{\boldsymbol{u}} \sim \psi_{ac}+\psi_{ev}+\psi_{cv}+\psi_{ac}\cdot\psi_{ce}+\psi_{ac}\cdot\psi_{ev}+\psi_{ce}\cdot\psi_{cv}+\psi_{ev}\cdot\psi_{cv}$	0.0985763	0.56082	61804.8
390	$\overline{\boldsymbol{u}} \sim \psi_{ac}+\psi_{ev}+\psi_{cv}+\psi_{ac}\cdot\psi_{ce}+\psi_{ac}\cdot\psi_{ev}+\psi_{ce}\cdot\psi_{cv}$	0.0985945	0.56081	61804.5
391	$\overline{\boldsymbol{u}} \sim \psi_{ce}+\psi_{ac}\cdot\psi_{ev}+\psi_{ac}\cdot\psi_{cv}+\psi_{ce}\cdot\psi_{ev}+\psi_{ce}\cdot\psi_{cv}+\psi_{ev}\cdot\psi_{cv}$	0.118635	0.56002	61859
392	$\overline{\boldsymbol{u}} \sim \psi_{ac}+\psi_{ac}\cdot\psi_{ce}+\psi_{ac}\cdot\psi_{ev}+\psi_{ce}\cdot\psi_{ev}+\psi_{ce}\cdot\psi_{cv}+\psi_{ev}\cdot\psi_{cv}$	0.0884521	0.55904	61926.3
393	$\overline{\boldsymbol{u}} \sim \psi_{ac}+\psi_{ac}\cdot\psi_{ce}+\psi_{ac}\cdot\psi_{cv}+\psi_{ce}\cdot\psi_{ev}+\psi_{ce}\cdot\psi_{cv}+\psi_{ev}\cdot\psi_{cv}$	0.107143	0.55578	62149.7
394	$\overline{\boldsymbol{u}} \sim \psi_{ev}+\psi_{ac}\cdot\psi_{ce}+\psi_{ac}\cdot\psi_{ev}+\psi_{ac}\cdot\psi_{cv}+\psi_{ce}\cdot\psi_{ev}+\psi_{ce}\cdot\psi_{cv}+\psi_{ev}\cdot\psi_{cv}$	0.108694	0.55464	62227.7
395	$\overline{\boldsymbol{u}} \sim \psi_{ac}+\psi_{ce}+\psi_{ev}+\psi_{ac}\cdot\psi_{ev}+\psi_{ac}\cdot\psi_{cv}+\psi_{ce}\cdot\psi_{ev}+\psi_{ev}\cdot\psi_{cv}$	0.14531	0.55435	62247.5
396	$\overline{\boldsymbol{u}} \sim \psi_{ac}+\psi_{cv}+\psi_{ac}\cdot\psi_{ce}+\psi_{ac}\cdot\psi_{ev}+\psi_{ce}\cdot\psi_{cv}+\psi_{ev}\cdot\psi_{cv}$	0.101954	0.55368	62292.3
397	$\overline{\boldsymbol{u}} \sim \psi_{ac}+\psi_{cv}+\psi_{ac}\cdot\psi_{ce}+\psi_{ac}\cdot\psi_{ev}+\psi_{ce}\cdot\psi_{cv}$	0.102393	0.55344	62307.2
398	$\overline{\boldsymbol{u}} \sim \psi_{ac}+\psi_{ce}+\psi_{ev}+\psi_{ac}\cdot\psi_{ev}+\psi_{ac}\cdot\psi_{cv}+\psi_{ev}\cdot\psi_{cv}$	0.145097	0.55343	62309.3
399	$\overline{\boldsymbol{u}} \sim \psi_{ac}+\psi_{ce}+\psi_{ac}\cdot\psi_{ev}+\psi_{ac}\cdot\psi_{cv}+\psi_{ce}\cdot\psi_{ev}+\psi_{ev}\cdot\psi_{cv}$	0.143477	0.55214	62396.1
400	$\overline{\boldsymbol{u}} \sim \psi_{ce}+\psi_{ev}+\psi_{ac}\cdot\psi_{ev}+\psi_{ac}\cdot\psi_{cv}+\psi_{ce}\cdot\psi_{ev}+\psi_{ev}\cdot\psi_{cv}$	0.141528	0.55211	62398.4
401	$\overline{\boldsymbol{u}} \sim \psi_{ac}+\psi_{ev}+\psi_{cv}+\psi_{ac}\cdot\psi_{ce}+\psi_{ac}\cdot\psi_{ev}+\psi_{ce}\cdot\psi_{ev}+\psi_{ev}\cdot\psi_{cv}$	0.0849117	0.55152	62439

402	$\overline{\boldsymbol{u}} \sim \psi_{ac}+\psi_{ev}+\psi_{cv}+\psi_{ac}\cdot\psi_{ce}+\psi_{ac}\cdot\psi_{ev}+\psi_{ce}\cdot\psi_{ev}$	0.0852434	0.55109	62467.4
403	$\overline{\boldsymbol{u}} \sim \psi_{ce}+\psi_{ev}+\psi_{ac}\cdot\psi_{ev}+\psi_{ac}\cdot\psi_{cv}+\psi_{ev}\cdot\psi_{cv}$	0.141067	0.55071	62492.1
404	$\overline{\boldsymbol{u}} \sim \psi_{ac}+\psi_{ce}+\psi_{ac}\cdot\psi_{ev}+\psi_{ac}\cdot\psi_{cv}+\psi_{ev}\cdot\psi_{cv}$	0.143525	0.55013	62530.9
405	$\overline{\boldsymbol{u}} \sim \psi_{ac}+\psi_{ev}+\psi_{cv}+\psi_{ac}\cdot\psi_{ce}+\psi_{ce}\cdot\psi_{ev}+\psi_{ev}\cdot\psi_{cv}$	0.0852222	0.55002	62539.2
406	$\overline{\boldsymbol{u}} \sim \psi_{ac}+\psi_{ev}+\psi_{cv}+\psi_{ac}\cdot\psi_{ce}+\psi_{ce}\cdot\psi_{ev}$	0.0853119	0.54998	62540.6
407	$\overline{\boldsymbol{u}} \sim \psi_{ac}+\psi_{ac}\cdot\psi_{ce}+\psi_{ac}\cdot\psi_{ev}+\psi_{ac}\cdot\psi_{cv}+\psi_{ce}\cdot\psi_{ev}+\psi_{ev}\cdot\psi_{cv}$	0.115783	0.54894	62611.6
408	$\overline{\boldsymbol{u}} \sim \psi_{ac}+\psi_{cv}+\psi_{ac}\cdot\psi_{ce}+\psi_{ce}\cdot\psi_{ev}+\psi_{ce}\cdot\psi_{cv}+\psi_{ev}\cdot\psi_{cv}$	0.0877185	0.5488	62621.2
409	$\overline{\boldsymbol{u}} \sim \psi_{ce}+\psi_{ac}\cdot\psi_{ev}+\psi_{ac}\cdot\psi_{cv}+\psi_{ce}\cdot\psi_{cv}+\psi_{ev}\cdot\psi_{cv}$	0.113676	0.54873	62624.7
410	$\overline{\boldsymbol{u}} \sim \psi_{ev}+\psi_{ac}\cdot\psi_{ce}+\psi_{ac}\cdot\psi_{ev}+\psi_{ac}\cdot\psi_{cv}+\psi_{ce}\cdot\psi_{ev}+\psi_{ev}\cdot\psi_{cv}$	0.114876	0.54824	62658.7
411	$\overline{\boldsymbol{u}} \sim \psi_{ac}+\psi_{ce}+\psi_{ev}+\psi_{ac}\cdot\psi_{cv}+\psi_{ce}\cdot\psi_{ev}+\psi_{ev}\cdot\psi_{cv}$	0.143907	0.54797	62676.5
412	$\overline{\boldsymbol{u}} \sim \psi_{ac}+\psi_{ce}+\psi_{ev}+\psi_{ac}\cdot\psi_{cv}+\psi_{ev}\cdot\psi_{cv}$	0.143677	0.54761	62699.6
413	$\overline{\boldsymbol{u}} \sim \psi_{ac}+\psi_{ce}+\psi_{ac}\cdot\psi_{cv}+\psi_{ev}\cdot\psi_{cv}$	0.143185	0.54704	62736.9
414	$\overline{\boldsymbol{u}} \sim \psi_{ac}+\psi_{ce}+\psi_{ac}\cdot\psi_{cv}+\psi_{ce}\cdot\psi_{ev}+\psi_{ev}\cdot\psi_{cv}$	0.143185	0.54703	62738.6
415	$\overline{\boldsymbol{u}} \sim \psi_{ac}+\psi_{ev}+\psi_{ac}\cdot\psi_{ce}+\psi_{ac}\cdot\psi_{ev}+\psi_{ce}\cdot\psi_{ev}+\psi_{ev}\cdot\psi_{cv}$	0.0861546	0.54237	63049.5
416	$\overline{\boldsymbol{u}} \sim \psi_{ac}+\psi_{ev}+\psi_{ac}\cdot\psi_{ce}+\psi_{ce}\cdot\psi_{ev}+\psi_{ev}\cdot\psi_{cv}$	0.0861734	0.54234	63050.3
417	$\overline{\boldsymbol{u}} \sim \psi_{ev}+\psi_{cv}+\psi_{ac}\cdot\psi_{ce}+\psi_{ac}\cdot\psi_{ev}+\psi_{ce}\cdot\psi_{ev}+\psi_{ce}\cdot\psi_{cv}+\psi_{ev}\cdot\psi_{cv}$	0.095416	0.54229	63055.6
418	$\overline{\boldsymbol{u}} \sim \psi_{ce}+\psi_{cv}+\psi_{ac}\cdot\psi_{ce}+\psi_{ac}\cdot\psi_{cv}+\psi_{ce}\cdot\psi_{cv}$	0.0975829	0.54186	63081.8
419	$\overline{\boldsymbol{u}} \sim \psi_{ce}+\psi_{ac}\cdot\psi_{ev}+\psi_{ac}\cdot\psi_{cv}+\psi_{ce}\cdot\psi_{ev}+\psi_{ev}\cdot\psi_{cv}$	0.126371	0.54089	63146.2
420	$\overline{\boldsymbol{u}} \sim \psi_{ac}+\psi_{ac}\cdot\psi_{ce}+\psi_{ac}\cdot\psi_{cv}+\psi_{ce}\cdot\psi_{ev}+\psi_{ev}\cdot\psi_{cv}$	0.125611	0.53992	63209.8
421	$\overline{\boldsymbol{u}} \sim \psi_{ce}+\psi_{cv}+\psi_{ac}\cdot\psi_{ce}+\psi_{ac}\cdot\psi_{cv}$	0.0972881	0.53922	63254.9
422	$\overline{\boldsymbol{u}} \sim \psi_{ac}+\psi_{ac}\cdot\psi_{ce}+\psi_{ce}\cdot\psi_{ev}+\psi_{ce}\cdot\psi_{cv}+\psi_{ev}\cdot\psi_{cv}$	0.0887442	0.53866	63292.8
423	$\overline{\boldsymbol{u}} \sim \psi_{ac}+\psi_{ev}+\psi_{ac}\cdot\psi_{ev}+\psi_{ac}\cdot\psi_{cv}+\psi_{ce}\cdot\psi_{ev}+\psi_{ce}\cdot\psi_{cv}+\psi_{ev}\cdot\psi_{cv}$	0.137046	0.5378	63351.1
424	$\overline{\boldsymbol{u}} \sim \psi_{ac}+\psi_{ev}+\psi_{ac}\cdot\psi_{ev}+\psi_{ac}\cdot\psi_{cv}+\psi_{ce}\cdot\psi_{ev}+\psi_{ev}\cdot\psi_{cv}$	0.139475	0.53657	63430.4
425	$\overline{\boldsymbol{u}} \sim \psi_{ac}+\psi_{cv}+\psi_{ac}\cdot\psi_{ce}+\psi_{ce}\cdot\psi_{cv}+\psi_{ev}\cdot\psi_{cv}$	0.0966702	0.53524	63516.2
426	$\overline{\boldsymbol{u}} \sim \psi_{ac}+\psi_{ev}+\psi_{ac}\cdot\psi_{cv}+\psi_{ce}\cdot\psi_{ev}+\psi_{ce}\cdot\psi_{cv}+\psi_{ev}\cdot\psi_{cv}$	0.135363	0.53435	63574.8
427	$\overline{\boldsymbol{u}} \sim \psi_{ev}+\psi_{cv}+\psi_{ac}\cdot\psi_{ce}+\psi_{ac}\cdot\psi_{ev}+\psi_{ce}\cdot\psi_{ev}+\psi_{ce}\cdot\psi_{cv}$	0.0967371	0.53427	63580.5
428	$\overline{\boldsymbol{u}} \sim \psi_{ac}+\psi_{ce}+\psi_{ev}+\psi_{ac}\cdot\psi_{cv}+\psi_{ce}\cdot\psi_{ev}+\psi_{ce}\cdot\psi_{cv}$	0.107013	0.53365	63620.7

429	$\overline{\boldsymbol{u}} \sim \psi_{ac}+\psi_{ce}+\psi_{ev}+\psi_{ac}\cdot\psi_{ev}+\psi_{ac}\cdot\psi_{cv}+\psi_{ce}\cdot\psi_{ev}+\psi_{ce}\cdot\psi_{cv}$	0.107027	0.53363	63622.6
430	$\overline{\boldsymbol{u}} \sim \psi_{ac}+\psi_{ce}+\psi_{ev}+\psi_{ac}\cdot\psi_{cv}+\psi_{ce}\cdot\psi_{cv}$	0.106829	0.53353	63627.2
431	$\overline{\boldsymbol{u}} \sim \psi_{ac}+\psi_{ce}+\psi_{ev}+\psi_{ac}\cdot\psi_{ev}+\psi_{ac}\cdot\psi_{cv}+\psi_{ce}\cdot\psi_{cv}$	0.106872	0.53352	63628.8
432	$\overline{\boldsymbol{u}} \sim \psi_{ev}+\psi_{ac}\cdot\psi_{ev}+\psi_{ac}\cdot\psi_{cv}+\psi_{ce}\cdot\psi_{ev}+\psi_{ce}\cdot\psi_{cv}+\psi_{ev}\cdot\psi_{cv}$	0.133814	0.53324	63647
433	$\overline{\boldsymbol{u}} \sim \psi_{ac}+\psi_{ev}+\psi_{ac}\cdot\psi_{cv}+\psi_{ce}\cdot\psi_{ev}+\psi_{ev}\cdot\psi_{cv}$	0.138343	0.53249	63694.4
434	$\overline{\boldsymbol{u}} \sim \psi_{ev}+\psi_{ac}\cdot\psi_{ev}+\psi_{ac}\cdot\psi_{cv}+\psi_{ce}\cdot\psi_{ev}+\psi_{ev}\cdot\psi_{cv}$	0.135817	0.53242	63699.3
435	$\overline{\boldsymbol{u}} \sim \psi_{ac}+\psi_{ac}\cdot\psi_{ev}+\psi_{ac}\cdot\psi_{cv}+\psi_{ce}\cdot\psi_{ev}+\psi_{ce}\cdot\psi_{cv}+\psi_{ev}\cdot\psi_{cv}$	0.138482	0.53025	63840.3
436	$\overline{\boldsymbol{u}} \sim \psi_{ce}+\psi_{ac}\cdot\psi_{ev}+\psi_{ac}\cdot\psi_{cv}+\psi_{ev}\cdot\psi_{cv}$	0.120868	0.53011	63847.6
437	$\overline{\boldsymbol{u}} \sim \psi_{ac}+\psi_{ac}\cdot\psi_{ev}+\psi_{ac}\cdot\psi_{cv}+\psi_{ce}\cdot\psi_{ev}+\psi_{ev}\cdot\psi_{cv}$	0.139411	0.52989	63862.8
438	$\overline{\boldsymbol{u}} \sim \psi_{ac}+\psi_{ac}\cdot\psi_{cv}+\psi_{ce}\cdot\psi_{ev}+\psi_{ce}\cdot\psi_{cv}+\psi_{ev}\cdot\psi_{cv}$	0.139028	0.5297	63874.6
439	$\overline{\boldsymbol{u}} \sim \psi_{ac}+\psi_{ac}\cdot\psi_{cv}+\psi_{ce}\cdot\psi_{ev}+\psi_{ev}\cdot\psi_{cv}$	0.139369	0.52964	63877.3
440	$\overline{\boldsymbol{u}} \sim \psi_{ac}+\psi_{ce}+\psi_{ev}+\psi_{cv}+\psi_{ce}\cdot\psi_{ev}+\psi_{ce}\cdot\psi_{cv}$	0.114323	0.52937	63896.7
441	$\overline{\boldsymbol{u}} \sim \psi_{ac}+\psi_{ce}+\psi_{ev}+\psi_{cv}+\psi_{ce}\cdot\psi_{ev}+\psi_{ce}\cdot\psi_{cv}+\psi_{ev}\cdot\psi_{cv}$	0.114333	0.52937	63897.7
442	$\overline{\boldsymbol{u}} \sim \psi_{ac}+\psi_{ev}+\psi_{ac}\cdot\psi_{ce}+\psi_{ac}\cdot\psi_{ev}+\psi_{ac}\cdot\psi_{cv}+\psi_{ce}\cdot\psi_{ev}+\psi_{ce}\cdot\psi_{cv}$	0.0656541	0.52937	63897.9
443	$\overline{\boldsymbol{u}} \sim \psi_{ac}+\psi_{ce}+\psi_{ev}+\psi_{cv}+\psi_{ac}\cdot\psi_{ev}+\psi_{ce}\cdot\psi_{ev}+\psi_{ce}\cdot\psi_{cv}$	0.114375	0.52936	63898.4
444	$\overline{\boldsymbol{u}} \sim \psi_{ac}+\psi_{ce}+\psi_{ev}+\psi_{cv}+\psi_{ac}\cdot\psi_{ev}+\psi_{ce}\cdot\psi_{ev}+\psi_{ce}\cdot\psi_{cv}+\psi_{ev}\cdot\psi_{cv}$	0.11435	0.52936	63899.7
445	$\overline{\boldsymbol{u}} \sim \psi_{ac}+\psi_{ce}+\psi_{ev}+\psi_{cv}+\psi_{ce}\cdot\psi_{ev}$	0.1158	0.5293	63900.4
446	$\overline{\boldsymbol{u}} \sim \psi_{ac}+\psi_{ce}+\psi_{ev}+\psi_{cv}+\psi_{ce}\cdot\psi_{ev}+\psi_{ev}\cdot\psi_{cv}$	0.115808	0.5293	63901.4
447	$\overline{\boldsymbol{u}} \sim \psi_{ac}+\psi_{ce}+\psi_{ev}+\psi_{cv}+\psi_{ac}\cdot\psi_{ev}+\psi_{ce}\cdot\psi_{ev}$	0.115853	0.52929	63902.1
448	$\overline{\boldsymbol{u}} \sim \psi_{ac}+\psi_{ce}+\psi_{ev}+\psi_{cv}+\psi_{ac}\cdot\psi_{ev}+\psi_{ce}\cdot\psi_{ev}+\psi_{ev}\cdot\psi_{cv}$	0.115826	0.52928	63903.4
449	$\overline{\boldsymbol{u}} \sim \psi_{ac}+\psi_{ce}+\psi_{ev}+\psi_{cv}+\psi_{ce}\cdot\psi_{cv}$	0.114057	0.52924	63904.1
450	$\overline{\boldsymbol{u}} \sim \psi_{ac}+\psi_{ce}+\psi_{ev}+\psi_{cv}+\psi_{ac}\cdot\psi_{ev}+\psi_{ce}\cdot\psi_{cv}$	0.114157	0.52924	63905.3
451	$\overline{\boldsymbol{u}} \sim \psi_{ac}+\psi_{ce}+\psi_{ev}+\psi_{cv}+\psi_{ce}\cdot\psi_{cv}+\psi_{ev}\cdot\psi_{cv}$	0.114066	0.52924	63905.4
452	$\overline{\boldsymbol{u}} \sim \psi_{ac}+\psi_{ce}+\psi_{ev}+\psi_{cv}+\psi_{ac}\cdot\psi_{ev}+\psi_{ce}\cdot\psi_{cv}+\psi_{ev}\cdot\psi_{cv}$	0.114138	0.52923	63907
453	$\overline{\boldsymbol{u}} \sim \psi_{ac}+\psi_{ce}+\psi_{ev}+\psi_{cv}$	0.115539	0.52917	63907.9
454	$\overline{\boldsymbol{u}} \sim \psi_{ac}+\psi_{ce}+\psi_{ev}+\psi_{cv}+\psi_{ac}\cdot\psi_{ev}$	0.115637	0.52917	63909
455	$\overline{\boldsymbol{u}} \sim \psi_{ac}+\psi_{ce}+\psi_{ev}+\psi_{cv}+\psi_{ev}\cdot\psi_{cv}$	0.115545	0.52916	63909.1

456	$\overline{\boldsymbol{u}} \sim \psi_{ac}+\psi_{ce}+\psi_{ev}+\psi_{cv}+\psi_{ac}\cdot\psi_{ev}+\psi_{ev}\cdot\psi_{cv}$	0.115618	0.52915	63910.7
457	$\overline{\boldsymbol{u}} \sim \psi_{ac}+\psi_{ev}+\psi_{ac}\cdot\psi_{ce}+\psi_{ac}\cdot\psi_{cv}+\psi_{ce}\cdot\psi_{ev}+\psi_{ce}\cdot\psi_{cv}$	0.065635	0.52804	63982.4
458	$\overline{\boldsymbol{u}} \sim \psi_{ac}+\psi_{ce}+\psi_{ac}\cdot\psi_{ev}+\psi_{ac}\cdot\psi_{cv}+\psi_{ce}\cdot\psi_{ev}+\psi_{ce}\cdot\psi_{cv}$	0.110866	0.5277	64004
459	$\overline{\boldsymbol{u}} \sim \psi_{ac}+\psi_{cv}+\psi_{ac}\cdot\psi_{ce}+\psi_{ac}\cdot\psi_{ev}+\psi_{ce}\cdot\psi_{ev}$	0.101354	0.52694	64051.8
460	$\overline{\boldsymbol{u}} \sim \psi_{ac}+\psi_{cv}+\psi_{ac}\cdot\psi_{ce}+\psi_{ac}\cdot\psi_{ev}+\psi_{ce}\cdot\psi_{ev}+\psi_{ev}\cdot\psi_{cv}$	0.101305	0.52692	64053.7
461	$\overline{\boldsymbol{u}} \sim \psi_{ac}+\psi_{ce}+\psi_{ac}\cdot\psi_{ev}+\psi_{ac}\cdot\psi_{cv}+\psi_{ce}\cdot\psi_{cv}$	0.111498	0.52659	64074
462	$\overline{\boldsymbol{u}} \sim \psi_{ac}+\psi_{ce}+\psi_{ev}+\psi_{ac}\cdot\psi_{ev}+\psi_{ce}\cdot\psi_{ev}+\psi_{ce}\cdot\psi_{cv}+\psi_{ev}\cdot\psi_{cv}$	0.1094	0.52591	64119.4
463	$\overline{\boldsymbol{u}} \sim \psi_{ac}+\psi_{ce}+\psi_{ev}+\psi_{ac}\cdot\psi_{ev}+\psi_{ce}\cdot\psi_{cv}+\psi_{ev}\cdot\psi_{cv}$	0.109106	0.52566	64134.3
464	$\overline{\boldsymbol{u}} \sim \psi_{ac}+\psi_{ce}+\psi_{ev}+\psi_{ce}\cdot\psi_{ev}+\psi_{ce}\cdot\psi_{cv}+\psi_{ev}\cdot\psi_{cv}$	0.109469	0.52563	64136.5
465	$\overline{\boldsymbol{u}} \sim \psi_{ac}+\psi_{ce}+\psi_{ev}+\psi_{ce}\cdot\psi_{cv}+\psi_{ev}\cdot\psi_{cv}$	0.109194	0.52545	64147
466	$\overline{\boldsymbol{u}} \sim \psi_{ev}+\psi_{ac}\cdot\psi_{ce}+\psi_{ac}\cdot\psi_{ev}+\psi_{ce}\cdot\psi_{ev}+\psi_{ce}\cdot\psi_{cv}+\psi_{ev}\cdot\psi_{cv}$	0.0950009	0.52502	64175.5
467	$\overline{\boldsymbol{u}} \sim \psi_{ac}+\psi_{ce}+\psi_{cv}+\psi_{ac}\cdot\psi_{ev}+\psi_{ce}\cdot\psi_{ev}+\psi_{ce}\cdot\psi_{cv}+\psi_{ev}\cdot\psi_{cv}$	0.118401	0.52386	64249.8
468	$\overline{\boldsymbol{u}} \sim \psi_{ac}+\psi_{ce}+\psi_{cv}+\psi_{ac}\cdot\psi_{ev}+\psi_{ce}\cdot\psi_{ev}+\psi_{ev}\cdot\psi_{cv}$	0.119704	0.52381	64252.4
469	$\overline{\boldsymbol{u}} \sim \psi_{ac}+\psi_{ce}+\psi_{cv}+\psi_{ac}\cdot\psi_{ev}+\psi_{ce}\cdot\psi_{ev}+\psi_{ce}\cdot\psi_{cv}$	0.119046	0.52368	64260.3
470	$\overline{\boldsymbol{u}} \sim \psi_{ac}+\psi_{ce}+\psi_{cv}+\psi_{ac}\cdot\psi_{ev}+\psi_{ce}\cdot\psi_{ev}$	0.120343	0.52363	64262.8
471	$\overline{\boldsymbol{u}} \sim \psi_{ac}+\psi_{ce}+\psi_{ev}+\psi_{ac}\cdot\psi_{ev}+\psi_{ce}\cdot\psi_{ev}+\psi_{ev}\cdot\psi_{cv}$	0.115743	0.52319	64291.7
472	$\overline{\boldsymbol{u}} \sim \psi_{ac}+\psi_{ce}+\psi_{ev}+\psi_{ac}\cdot\psi_{ev}+\psi_{ev}\cdot\psi_{cv}$	0.115551	0.52284	64312.9
473	$\overline{\boldsymbol{u}} \sim \psi_{ac}+\psi_{ce}+\psi_{cv}+\psi_{ac}\cdot\psi_{ev}+\psi_{ce}\cdot\psi_{cv}+\psi_{ev}\cdot\psi_{cv}$	0.11922	0.52271	64322
474	$\overline{\boldsymbol{u}} \sim \psi_{ac}+\psi_{ce}+\psi_{cv}+\psi_{ac}\cdot\psi_{ev}+\psi_{ev}\cdot\psi_{cv}$	0.120504	0.52266	64324.4
475	$\overline{\boldsymbol{u}} \sim \psi_{ac}+\psi_{ce}+\psi_{cv}+\psi_{ac}\cdot\psi_{ev}+\psi_{ce}\cdot\psi_{cv}$	0.11978	0.52258	64329.2
476	$\overline{\boldsymbol{u}} \sim \psi_{ac}+\psi_{ce}+\psi_{cv}+\psi_{ac}\cdot\psi_{ev}$	0.121058	0.52253	64331.6
477	$\overline{\boldsymbol{u}} \sim \psi_{ac}+\psi_{ce}+\psi_{ev}+\psi_{ce}\cdot\psi_{ev}+\psi_{ev}\cdot\psi_{cv}$	0.116627	0.5224	64341
478	$\overline{\boldsymbol{u}} \sim \psi_{ac}+\psi_{ce}+\psi_{ev}+\psi_{ev}\cdot\psi_{cv}$	0.116386	0.52217	64354.2
479	$\overline{\boldsymbol{u}} \sim \psi_{ac}+\psi_{ce}+\psi_{ac}\cdot\psi_{ev}+\psi_{ce}\cdot\psi_{ev}+\psi_{ce}\cdot\psi_{cv}+\psi_{ev}\cdot\psi_{cv}$	0.112709	0.52126	64414
480	$\overline{\boldsymbol{u}} \sim \psi_{ac}+\psi_{ac}\cdot\psi_{ce}+\psi_{ac}\cdot\psi_{ev}+\psi_{ce}\cdot\psi_{ev}+\psi_{ev}\cdot\psi_{cv}$	0.0990671	0.52108	64424.3
481	$\overline{\boldsymbol{u}} \sim \psi_{ac}+\psi_{ce}+\psi_{ev}+\psi_{ce}\cdot\psi_{ev}+\psi_{ce}\cdot\psi_{cv}$	0.103463	0.52033	64471.9
482	$\overline{\boldsymbol{u}} \sim \psi_{ac}+\psi_{ce}+\psi_{ev}+\psi_{ac}\cdot\psi_{ev}+\psi_{ce}\cdot\psi_{ev}+\psi_{ce}\cdot\psi_{cv}$	0.103502	0.52031	64473.7

483	$\overline{\boldsymbol{u}} \sim \psi_{ac}+\psi_{ce}+\psi_{ev}+\psi_{ce}\cdot\psi_{cv}$	0.103238	0.5202	64479.1
484	$\overline{\boldsymbol{u}} \sim \psi_{ac}+\psi_{ce}+\psi_{ev}+\psi_{ac}\cdot\psi_{ev}+\psi_{ce}\cdot\psi_{cv}$	0.103315	0.52019	64480.4
485	$\overline{\boldsymbol{u}} \sim \psi_{ac}+\psi_{ev}+\psi_{cv}+\psi_{ac}\cdot\psi_{ev}+\psi_{ce}\cdot\psi_{ev}+\psi_{ce}\cdot\psi_{cv}$	0.122348	0.52	64493.3
486	$\overline{\boldsymbol{u}} \sim \psi_{ac}+\psi_{ev}+\psi_{cv}+\psi_{ac}\cdot\psi_{ev}+\psi_{ce}\cdot\psi_{ev}+\psi_{ce}\cdot\psi_{cv}+\psi_{ev}\cdot\psi_{cv}$	0.122336	0.51999	64494.8
487	$\overline{\boldsymbol{u}} \sim \psi_{ac}+\psi_{ce}+\psi_{ac}\cdot\psi_{ev}+\psi_{ce}\cdot\psi_{cv}+\psi_{ev}\cdot\psi_{cv}$	0.113312	0.51994	64496.4
488	$\overline{\boldsymbol{u}} \sim \psi_{ac}+\psi_{ev}+\psi_{cv}+\psi_{ce}\cdot\psi_{ev}+\psi_{ce}\cdot\psi_{cv}$	0.122167	0.51988	64499.9
489	$\overline{\boldsymbol{u}} \sim \psi_{ac}+\psi_{ev}+\psi_{cv}+\psi_{ce}\cdot\psi_{ev}+\psi_{ce}\cdot\psi_{cv}+\psi_{ev}\cdot\psi_{cv}$	0.122181	0.51987	64501.7
490	$\overline{\boldsymbol{u}} \sim \psi_{ac}+\psi_{ev}+\psi_{ac}\cdot\psi_{ce}+\psi_{ac}\cdot\psi_{ev}+\psi_{ac}\cdot\psi_{cv}+\psi_{ce}\cdot\psi_{ev}$	0.0783389	0.51914	64547.5
491	$\overline{\boldsymbol{u}} \sim \psi_{ac}+\psi_{ce}+\psi_{ac}\cdot\psi_{ev}+\psi_{ce}\cdot\psi_{ev}+\psi_{ev}\cdot\psi_{cv}$	0.117464	0.51911	64548.4
492	$\overline{\boldsymbol{u}} \sim \psi_{ac}+\psi_{cv}+\psi_{ac}\cdot\psi_{ce}+\psi_{ac}\cdot\psi_{cv}+\psi_{ce}\cdot\psi_{cv}$	0.206862	0.51821	64605.2
493	$\overline{\boldsymbol{u}} \sim \psi_{ac}+\psi_{ev}+\psi_{ac}\cdot\psi_{ce}+\psi_{ac}\cdot\psi_{cv}+\psi_{ce}\cdot\psi_{ev}$	0.07837	0.51777	64632.6
494	$\overline{\boldsymbol{u}} \sim \psi_{ac}+\psi_{ce}+\psi_{ac}\cdot\psi_{ev}+\psi_{ev}\cdot\psi_{cv}$	0.118122	0.51769	64636.7
495	$\overline{\boldsymbol{u}} \sim \psi_{ac}+\psi_{ce}+\psi_{ce}\cdot\psi_{ev}+\psi_{ce}\cdot\psi_{cv}+\psi_{ev}\cdot\psi_{cv}$	0.111683	0.51569	64762.7
496	$\overline{\boldsymbol{u}} \sim \psi_{ac}+\psi_{ce}+\psi_{cv}+\psi_{ce}\cdot\psi_{ev}+\psi_{ce}\cdot\psi_{cv}+\psi_{ev}\cdot\psi_{cv}$	0.111459	0.51569	64764
497	$\overline{\boldsymbol{u}} \sim \psi_{ac}+\psi_{ce}+\psi_{cv}+\psi_{ce}\cdot\psi_{ev}+\psi_{ev}\cdot\psi_{cv}$	0.112702	0.51562	64767.4
498	$\overline{\boldsymbol{u}} \sim \psi_{ac}+\psi_{ce}+\psi_{ce}\cdot\psi_{ev}+\psi_{ev}\cdot\psi_{cv}$	0.11288	0.51561	64766.8
499	$\overline{\boldsymbol{u}} \sim \psi_{ac}+\psi_{ev}+\psi_{cv}+\psi_{ac}\cdot\psi_{ev}+\psi_{ce}\cdot\psi_{ev}+\psi_{ev}\cdot\psi_{cv}$	0.112883	0.51459	64832.6
500	$\overline{\boldsymbol{u}} \sim \psi_{ac}+\psi_{ce}+\psi_{ac}\cdot\psi_{ev}+\psi_{ce}\cdot\psi_{ev}+\psi_{ce}\cdot\psi_{cv}$	0.107959	0.51455	64833.8
501	$\overline{\boldsymbol{u}} \sim \psi_{ac}+\psi_{ev}+\psi_{cv}+\psi_{ac}\cdot\psi_{ev}+\psi_{ce}\cdot\psi_{ev}$	0.112884	0.51455	64834.2
502	$\overline{\boldsymbol{u}} \sim \psi_{ac}+\psi_{ev}+\psi_{cv}+\psi_{ce}\cdot\psi_{ev}$	0.112526	0.51426	64851
503	$\overline{\boldsymbol{u}} \sim \psi_{ac}+\psi_{ev}+\psi_{cv}+\psi_{ce}\cdot\psi_{ev}+\psi_{ev}\cdot\psi_{cv}$	0.112522	0.51425	64853
504	$\overline{\boldsymbol{u}} \sim \psi_{ac}+\psi_{ev}+\psi_{cv}+\psi_{ac}\cdot\psi_{ev}+\psi_{ce}\cdot\psi_{cv}$	0.130151	0.51416	64858.5
505	$\overline{\boldsymbol{u}} \sim \psi_{ac}+\psi_{ev}+\psi_{cv}+\psi_{ce}\cdot\psi_{cv}$	0.130002	0.51415	64858.2
506	$\overline{\boldsymbol{u}} \sim \psi_{ac}+\psi_{ev}+\psi_{cv}+\psi_{ce}\cdot\psi_{cv}+\psi_{ev}\cdot\psi_{cv}$	0.130013	0.51415	64859.2
507	$\overline{\boldsymbol{u}} \sim \psi_{ac}+\psi_{ev}+\psi_{cv}+\psi_{ac}\cdot\psi_{ev}+\psi_{ce}\cdot\psi_{cv}+\psi_{ev}\cdot\psi_{cv}$	0.130133	0.51415	64860.2
508	$\overline{\boldsymbol{u}} \sim \psi_{ac}+\psi_{ce}+\psi_{ac}\cdot\psi_{ev}+\psi_{ce}\cdot\psi_{cv}$	0.108643	0.51343	64902.8
509	$\overline{\boldsymbol{u}} \sim \psi_{ac}+\psi_{ev}+\psi_{ac}\cdot\psi_{ce}+\psi_{ac}\cdot\psi_{ev}+\psi_{ac}\cdot\psi_{cv}+\psi_{ce}\cdot\psi_{cv}+\psi_{ev}\cdot\psi_{cv}$	0.131955	0.51246	64966.1

510	$\overline{\boldsymbol{u}} \sim \psi_{ev}+\psi_{cv}+\psi_{ac}\cdot\psi_{ce}+\psi_{ac}\cdot\psi_{ev}+\psi_{ce}\cdot\psi_{cv}+\psi_{ev}\cdot\psi_{cv}$	0.106652	0.5119	64999.9
511	$\overline{\boldsymbol{u}} \sim \psi_{ac}+\psi_{cv}+\psi_{ac}\cdot\psi_{ev}+\psi_{ce}\cdot\psi_{ev}+\psi_{ce}\cdot\psi_{cv}+\psi_{ev}\cdot\psi_{cv}$	0.130538	0.51128	65037.9
512	$\overline{\boldsymbol{u}} \sim \psi_{ac}+\psi_{cv}+\psi_{ac}\cdot\psi_{ev}+\psi_{ce}\cdot\psi_{ev}+\psi_{ce}\cdot\psi_{cv}$	0.130935	0.51122	65041.1
513	$\overline{\boldsymbol{u}} \sim \psi_{ac}+\psi_{ev}+\psi_{ac}\cdot\psi_{ev}+\psi_{ac}\cdot\psi_{cv}+\psi_{ce}\cdot\psi_{cv}+\psi_{ev}\cdot\psi_{cv}$	0.142024	0.51077	65069.6
514	$\overline{\boldsymbol{u}} \sim \psi_{ac}+\psi_{ev}+\psi_{ac}\cdot\psi_{ce}+\psi_{ac}\cdot\psi_{ev}+\psi_{ce}\cdot\psi_{ev}+\psi_{ce}\cdot\psi_{cv}$	0.0809161	0.51043	65090.6
515	$\overline{\boldsymbol{u}} \sim \psi_{ac}+\psi_{ac}\cdot\psi_{ce}+\psi_{ac}\cdot\psi_{ev}+\psi_{ac}\cdot\psi_{cv}+\psi_{ce}\cdot\psi_{cv}+\psi_{ev}\cdot\psi_{cv}$	0.130929	0.50984	65127.5
516	$\overline{\boldsymbol{u}} \sim \psi_{ac}+\psi_{ce}+\psi_{cv}+\psi_{ac}\cdot\psi_{cv}$	0.266881	0.50981	65127.3
517	$\overline{\boldsymbol{u}} \sim \psi_{ac}+\psi_{ce}+\psi_{cv}+\psi_{ac}\cdot\psi_{cv}+\psi_{ce}\cdot\psi_{cv}$	0.268756	0.5098	65128.5
518	$\overline{\boldsymbol{u}} \sim \psi_{ac}+\psi_{ev}+\psi_{ac}\cdot\psi_{ce}+\psi_{ce}\cdot\psi_{ev}+\psi_{ce}\cdot\psi_{cv}$	0.080994	0.509	65178.2
519	$\overline{\boldsymbol{u}} \sim \psi_{ev}+\psi_{ac}\cdot\psi_{ce}+\psi_{ac}\cdot\psi_{ev}+\psi_{ac}\cdot\psi_{cv}+\psi_{ce}\cdot\psi_{cv}+\psi_{ev}\cdot\psi_{cv}$	0.134248	0.50897	65180.8
520	$\overline{\boldsymbol{u}} \sim \psi_{ac}+\psi_{ev}+\psi_{ac}\cdot\psi_{ce}+\psi_{ac}\cdot\psi_{ev}+\psi_{ce}\cdot\psi_{ev}$	0.0770476	0.50863	65200.9
521	$\overline{\boldsymbol{u}} \sim \psi_{ce}+\psi_{ev}+\psi_{ac}\cdot\psi_{ev}+\psi_{ac}\cdot\psi_{cv}+\psi_{ce}\cdot\psi_{ev}+\psi_{ce}\cdot\psi_{cv}$	0.104239	0.5085	65209.8
522	$\overline{\boldsymbol{u}} \sim \psi_{ev}+\psi_{ac}\cdot\psi_{ev}+\psi_{ac}\cdot\psi_{cv}+\psi_{ce}\cdot\psi_{cv}+\psi_{ev}\cdot\psi_{cv}$	0.139318	0.50839	65215.7
523	$\overline{\boldsymbol{u}} \sim \psi_{ac}+\psi_{ac}\cdot\psi_{ev}+\psi_{ac}\cdot\psi_{cv}+\psi_{ce}\cdot\psi_{cv}+\psi_{ev}\cdot\psi_{cv}$	0.140741	0.50811	65232.7
524	$\overline{\boldsymbol{u}} \sim \psi_{ac}+\psi_{cv}+\psi_{ac}\cdot\psi_{ev}+\psi_{ce}\cdot\psi_{cv}+\psi_{ev}\cdot\psi_{cv}$	0.134133	0.50794	65243.3
525	$\overline{\boldsymbol{u}} \sim \psi_{ac}+\psi_{cv}+\psi_{ac}\cdot\psi_{ev}+\psi_{ce}\cdot\psi_{cv}$	0.134624	0.50783	65249.3
526	$\overline{\boldsymbol{u}} \sim \psi_{ce}+\psi_{ev}+\psi_{ac}\cdot\psi_{ev}+\psi_{ac}\cdot\psi_{cv}+\psi_{ce}\cdot\psi_{cv}$	0.103887	0.50757	65266
527	$\overline{\boldsymbol{u}} \sim \psi_{ac}+\psi_{ev}+\psi_{ac}\cdot\psi_{ev}+\psi_{ce}\cdot\psi_{ev}+\psi_{ce}\cdot\psi_{cv}+\psi_{ev}\cdot\psi_{cv}$	0.116738	0.50731	65283
528	$\overline{\boldsymbol{u}} \sim \psi_{ce}+\psi_{ev}+\psi_{ac}\cdot\psi_{ce}+\psi_{ac}\cdot\psi_{cv}+\psi_{ce}\cdot\psi_{ev}+\psi_{ce}\cdot\psi_{cv}+\psi_{ev}\cdot\psi_{cv}$	0.0784363	0.50727	65286.5
529	$\overline{\boldsymbol{u}} \sim \psi_{ac}+\psi_{ev}+\psi_{ac}\cdot\psi_{ce}+\psi_{ce}\cdot\psi_{ev}$	0.0770921	0.50717	65289.7
530	$\overline{\boldsymbol{u}} \sim \psi_{ac}+\psi_{ev}+\psi_{ce}\cdot\psi_{ev}+\psi_{ce}\cdot\psi_{cv}+\psi_{ev}\cdot\psi_{cv}$	0.116766	0.50696	65303.6
531	$\overline{\boldsymbol{u}} \sim \psi_{ce}+\psi_{ev}+\psi_{ac}\cdot\psi_{ce}+\psi_{ac}\cdot\psi_{cv}+\psi_{ce}\cdot\psi_{cv}+\psi_{ev}\cdot\psi_{cv}$	0.078356	0.5068	65314.5
532	$\overline{\boldsymbol{u}} \sim \psi_{ac}+\psi_{ev}+\psi_{ac}\cdot\psi_{ev}+\psi_{ce}\cdot\psi_{ev}+\psi_{ev}\cdot\psi_{cv}$	0.113641	0.50645	65334.5
533	$\overline{\boldsymbol{u}} \sim \psi_{ac}+\psi_{ev}+\psi_{ce}\cdot\psi_{ev}+\psi_{ev}\cdot\psi_{cv}$	0.113963	0.50631	65342.2
534	$\overline{\boldsymbol{u}} \sim \psi_{ev}+\psi_{cv}+\psi_{ac}\cdot\psi_{ce}+\psi_{ac}\cdot\psi_{ev}+\psi_{ce}\cdot\psi_{cv}$	0.108269	0.50515	65414.4
535	$\overline{\boldsymbol{u}} \sim \psi_{ac}+\psi_{ev}+\psi_{ac}\cdot\psi_{ce}+\psi_{ac}\cdot\psi_{cv}+\psi_{ce}\cdot\psi_{cv}+\psi_{ev}\cdot\psi_{cv}$	0.132483	0.50486	65433.1
536	$\overline{\boldsymbol{u}} \sim \psi_{cv}+\psi_{ac}\cdot\psi_{ce}+\psi_{ac}\cdot\psi_{cv}+\psi_{ce}\cdot\psi_{cv}$	0.149818	0.50447	65455.1

537	$\overline{\boldsymbol{u}} \sim \psi_{ac}+\psi_{ev}+\psi_{ac}\cdot\psi_{cv}+\psi_{ce}\cdot\psi_{cv}+\psi_{ev}\cdot\psi_{cv}$	0.141499	0.50358	65510.4
538	$\overline{\boldsymbol{u}} \sim \psi_{ce}+\psi_{cv}+\psi_{ac}\cdot\psi_{cv}$	0.165312	0.50339	65520.1
539	$\overline{\boldsymbol{u}} \sim \psi_{ce}+\psi_{cv}+\psi_{ac}\cdot\psi_{cv}+\psi_{ce}\cdot\psi_{cv}$	0.165855	0.50338	65521.5
540	$\overline{\boldsymbol{u}} \sim \psi_{ac}+\psi_{ev}+\psi_{ac}\cdot\psi_{ce}+\psi_{ac}\cdot\psi_{ev}+\psi_{ce}\cdot\psi_{cv}+\psi_{ev}\cdot\psi_{cv}$	0.110969	0.50325	65531.4
541	$\overline{\boldsymbol{u}} \sim \psi_{ac}+\psi_{ce}+\psi_{cv}+\psi_{ce}\cdot\psi_{cv}+\psi_{ev}\cdot\psi_{cv}$	0.108488	0.50323	65531.3
542	$\overline{\boldsymbol{u}} \sim \psi_{ac}+\psi_{ce}+\psi_{cv}+\psi_{ev}\cdot\psi_{cv}$	0.109667	0.50316	65534.9
543	$\overline{\boldsymbol{u}} \sim \psi_{ac}+\psi_{cv}+\psi_{ac}\cdot\psi_{cv}+\psi_{ce}\cdot\psi_{cv}$	0.264915	0.50316	65535.1
544	$\overline{\boldsymbol{u}} \sim \psi_{ac}+\psi_{cv}+\psi_{ac}\cdot\psi_{ev}+\psi_{ce}\cdot\psi_{ev}+\psi_{ev}\cdot\psi_{cv}$	0.121634	0.50274	65561.3
545	$\overline{\boldsymbol{u}} \sim \psi_{ac}+\psi_{cv}+\psi_{ac}\cdot\psi_{ev}+\psi_{ce}\cdot\psi_{ev}$	0.121901	0.50272	65561.5
546	$\overline{\boldsymbol{u}} \sim \psi_{ac}+\psi_{ce}+\psi_{ac}\cdot\psi_{cv}+\psi_{ce}\cdot\psi_{ev}+\psi_{ce}\cdot\psi_{cv}$	0.103009	0.50207	65601.9
547	$\overline{\boldsymbol{u}} \sim \psi_{ac}+\psi_{ac}\cdot\psi_{ce}+\psi_{ac}\cdot\psi_{ev}+\psi_{ac}\cdot\psi_{cv}+\psi_{ce}\cdot\psi_{ev}+\psi_{ce}\cdot\psi_{cv}$	0.0816312	0.50201	65606.6
548	$\overline{\boldsymbol{u}} \sim \psi_{ce}+\psi_{ev}+\psi_{ac}\cdot\psi_{cv}+\psi_{ce}\cdot\psi_{ev}+\psi_{ce}\cdot\psi_{cv}+\psi_{ev}\cdot\psi_{cv}$	0.0904723	0.50179	65620.2
549	$\overline{\boldsymbol{u}} \sim \psi_{ac}+\psi_{ac}\cdot\psi_{ce}+\psi_{ac}\cdot\psi_{cv}+\psi_{ce}\cdot\psi_{cv}+\psi_{ev}\cdot\psi_{cv}$	0.132732	0.50137	65644.7
550	$\overline{\boldsymbol{u}} \sim \psi_{ce}+\psi_{ev}+\psi_{ac}\cdot\psi_{cv}+\psi_{ce}\cdot\psi_{cv}+\psi_{ev}\cdot\psi_{cv}$	0.0903461	0.50131	65648.1
551	$\overline{\boldsymbol{u}} \sim \psi_{ce}+\psi_{ac}\cdot\psi_{ce}+\psi_{ac}\cdot\psi_{cv}+\psi_{ce}\cdot\psi_{ev}+\psi_{ce}\cdot\psi_{cv}+\psi_{ev}\cdot\psi_{cv}$	0.076379	0.50089	65674.9
552	$\overline{\boldsymbol{u}} \sim \psi_{ac}+\psi_{ac}\cdot\psi_{cv}+\psi_{ce}\cdot\psi_{cv}+\psi_{ev}\cdot\psi_{cv}$	0.141016	0.50056	65692.5
553	$\overline{\boldsymbol{u}} \sim \psi_{ac}+\psi_{ev}+\psi_{ac}\cdot\psi_{ce}+\psi_{ac}\cdot\psi_{ev}+\psi_{ac}\cdot\psi_{cv}+\psi_{ev}\cdot\psi_{cv}$	0.154229	0.50025	65713.6
554	$\overline{\boldsymbol{u}} \sim \psi_{ac}+\psi_{ac}\cdot\psi_{ce}+\psi_{ac}\cdot\psi_{ev}+\psi_{ce}\cdot\psi_{cv}+\psi_{ev}\cdot\psi_{cv}$	0.110693	0.49996	65730.1
555	$\overline{\boldsymbol{u}} \sim \psi_{ev}+\psi_{ac}\cdot\psi_{ce}+\psi_{ac}\cdot\psi_{ev}+\psi_{ac}\cdot\psi_{cv}+\psi_{ev}\cdot\psi_{cv}$	0.151331	0.49938	65765
556	$\overline{\boldsymbol{u}} \sim \psi_{ac}+\psi_{ac}\cdot\psi_{ev}+\psi_{ce}\cdot\psi_{ev}+\psi_{ce}\cdot\psi_{cv}+\psi_{ev}\cdot\psi_{cv}$	0.122359	0.49915	65778.9
557	$\overline{\boldsymbol{u}} \sim \psi_{ac}\cdot\psi_{ce}+\psi_{ac}\cdot\psi_{ev}+\psi_{ac}\cdot\psi_{cv}+\psi_{ce}\cdot\psi_{ev}+\psi_{ce}\cdot\psi_{cv}+\psi_{ev}\cdot\psi_{cv}$	0.114688	0.49905	65786.2
558	$\overline{\boldsymbol{u}} \sim \psi_{ce}+\psi_{ev}+\psi_{cv}+\psi_{ac}\cdot\psi_{ev}+\psi_{ce}\cdot\psi_{ev}+\psi_{ev}\cdot\psi_{cv}$	0.113775	0.49903	65787.2
559	$\overline{\boldsymbol{u}} \sim \psi_{ce}+\psi_{ev}+\psi_{cv}+\psi_{ac}\cdot\psi_{ev}+\psi_{ce}\cdot\psi_{ev}+\psi_{ce}\cdot\psi_{cv}+\psi_{ev}\cdot\psi_{cv}$	0.114074	0.49902	65788.7
560	$\overline{\boldsymbol{u}} \sim \psi_{ce}+\psi_{ac}\cdot\psi_{ce}+\psi_{ac}\cdot\psi_{cv}+\psi_{ce}\cdot\psi_{cv}+\psi_{ev}\cdot\psi_{cv}$	0.0756115	0.49894	65791.6
561	$\overline{\boldsymbol{u}} \sim \psi_{ce}+\psi_{ev}+\psi_{ac}\cdot\psi_{ev}+\psi_{ce}\cdot\psi_{ev}+\psi_{ce}\cdot\psi_{cv}+\psi_{ev}\cdot\psi_{cv}$	0.113196	0.49889	65796
562	$\overline{\boldsymbol{u}} \sim \psi_{ce}+\psi_{ev}+\psi_{ac}\cdot\psi_{ev}+\psi_{ce}\cdot\psi_{ev}+\psi_{ev}\cdot\psi_{cv}$	0.113722	0.49886	65796.3
563	$\overline{\boldsymbol{u}} \sim \psi_{ac}+\psi_{ce}+\psi_{ce}\cdot\psi_{cv}+\psi_{ev}\cdot\psi_{cv}$	0.1123	0.49882	65798.1

564	$\overline{\boldsymbol{u}} \sim \psi_{ce}+\psi_{cv}+\psi_{ac}\cdot\psi_{ce}+\psi_{ac}\cdot\psi_{ev}+\psi_{ce}\cdot\psi_{ev}+\psi_{ce}\cdot\psi_{cv}+\psi_{ev}\cdot\psi_{cv}$	0.0569888	0.49882	65800.9
565	$\overline{\boldsymbol{u}} \sim \psi_{ac}\cdot\psi_{ev}+\psi_{ac}\cdot\psi_{cv}+\psi_{ce}\cdot\psi_{ev}+\psi_{ce}\cdot\psi_{cv}+\psi_{ev}\cdot\psi_{cv}$	0.117805	0.49872	65805.2
566	$\overline{\boldsymbol{u}} \sim \psi_{ac}+\psi_{ev}+\psi_{ac}\cdot\psi_{ce}+\psi_{ce}\cdot\psi_{cv}+\psi_{ev}\cdot\psi_{cv}$	0.114233	0.49849	65818.9
567	$\overline{\boldsymbol{u}} \sim \psi_{ac}+\psi_{ac}\cdot\psi_{ce}+\psi_{ce}\cdot\psi_{cv}+\psi_{ev}\cdot\psi_{cv}$	0.11629	0.4982	65835.1
568	$\overline{\boldsymbol{u}} \sim \psi_{ac}+\psi_{ce}+\psi_{cv}+\psi_{ce}\cdot\psi_{ev}+\psi_{ce}\cdot\psi_{cv}$	0.108137	0.49794	65852.4
569	$\overline{\boldsymbol{u}} \sim \psi_{ac}+\psi_{ce}+\psi_{cv}+\psi_{ce}\cdot\psi_{ev}$	0.10929	0.49785	65856.2
570	$\overline{\boldsymbol{u}} \sim \psi_{cv}+\psi_{ac}\cdot\psi_{cv}+\psi_{ce}\cdot\psi_{cv}$	0.176553	0.49773	65862.6
571	$\overline{\boldsymbol{u}} \sim \psi_{ac}+\psi_{ac}\cdot\psi_{ce}+\psi_{ac}\cdot\psi_{ev}+\psi_{ac}\cdot\psi_{cv}+\psi_{ev}\cdot\psi_{cv}$	0.152459	0.49757	65874.4
572	$\overline{\boldsymbol{u}} \sim \psi_{ce}+\psi_{ev}+\psi_{cv}+\psi_{ac}\cdot\psi_{ev}+\psi_{ev}\cdot\psi_{cv}$	0.113549	0.49756	65875
573	$\overline{\boldsymbol{u}} \sim \psi_{ce}+\psi_{ev}+\psi_{cv}+\psi_{ac}\cdot\psi_{ev}+\psi_{ce}\cdot\psi_{cv}+\psi_{ev}\cdot\psi_{cv}$	0.113882	0.49755	65876.4
574	$\overline{\boldsymbol{u}} \sim \psi_{ac}\cdot\psi_{ce}+\psi_{ac}\cdot\psi_{ev}+\psi_{ac}\cdot\psi_{cv}+\psi_{ce}\cdot\psi_{ev}+\psi_{ev}\cdot\psi_{cv}$	0.117587	0.49749	65878.9
575	$\overline{\boldsymbol{u}} \sim \psi_{ce}+\psi_{ev}+\psi_{ac}\cdot\psi_{ev}+\psi_{ce}\cdot\psi_{cv}+\psi_{ev}\cdot\psi_{cv}$	0.112938	0.49738	65885.7
576	$\overline{\boldsymbol{u}} \sim \psi_{ce}+\psi_{ev}+\psi_{ac}\cdot\psi_{ev}+\psi_{ev}\cdot\psi_{cv}$	0.113516	0.49735	65886.5
577	$\overline{\boldsymbol{u}} \sim \psi_{ac}\cdot\psi_{ev}+\psi_{ac}\cdot\psi_{cv}+\psi_{ce}\cdot\psi_{ev}+\psi_{ev}\cdot\psi_{cv}$	0.115213	0.4973	65889.9
578	$\overline{\boldsymbol{u}} \sim \psi_{ce}+\psi_{ac}\cdot\psi_{cv}+\psi_{ce}\cdot\psi_{ev}+\psi_{ce}\cdot\psi_{cv}+\psi_{ev}\cdot\psi_{cv}$	0.0871561	0.49679	65921.1
579	$\overline{\boldsymbol{u}} \sim \psi_{ac}+\psi_{ac}\cdot\psi_{ev}+\psi_{ce}\cdot\psi_{ev}+\psi_{ev}\cdot\psi_{cv}$	0.119085	0.49666	65928
580	$\overline{\boldsymbol{u}} \sim \psi_{ce}+\psi_{ac}\cdot\psi_{cv}+\psi_{ce}\cdot\psi_{cv}+\psi_{ev}\cdot\psi_{cv}$	0.0858999	0.49548	65998.9
581	$\overline{\boldsymbol{u}} \sim \psi_{ce}+\psi_{ev}+\psi_{cv}+\psi_{ac}\cdot\psi_{ev}+\psi_{ce}\cdot\psi_{ev}+\psi_{ce}\cdot\psi_{cv}$	0.115052	0.4943	66071.8
582	$\overline{\boldsymbol{u}} \sim \psi_{ce}+\psi_{ev}+\psi_{cv}+\psi_{ac}\cdot\psi_{ev}+\psi_{ce}\cdot\psi_{ev}$	0.114578	0.49429	66071
583	$\overline{\boldsymbol{u}} \sim \psi_{ac}+\psi_{ev}+\psi_{ac}\cdot\psi_{ce}+\psi_{ac}\cdot\psi_{cv}+\psi_{ev}\cdot\psi_{cv}$	0.154529	0.49369	66107.1
584	$\overline{\boldsymbol{u}} \sim \psi_{ac}+\psi_{ac}\cdot\psi_{ce}+\psi_{ac}\cdot\psi_{ev}+\psi_{ac}\cdot\psi_{cv}+\psi_{ce}\cdot\psi_{ev}$	0.0951345	0.49343	66122.6
585	$\overline{\boldsymbol{u}} \sim \psi_{ce}+\psi_{ev}+\psi_{cv}+\psi_{ac}\cdot\psi_{ev}$	0.114215	0.49305	66144.6
586	$\overline{\boldsymbol{u}} \sim \psi_{ce}+\psi_{ev}+\psi_{cv}+\psi_{ac}\cdot\psi_{ev}+\psi_{ce}\cdot\psi_{cv}$	0.114711	0.49305	66145.3
587	$\overline{\boldsymbol{u}} \sim \psi_{ac}+\psi_{ce}+\psi_{ev}\cdot\psi_{cv}$	0.101566	0.49296	66148.5
588	$\overline{\boldsymbol{u}} \sim \psi_{ac}+\psi_{ce}+\psi_{ev}+\psi_{ac}\cdot\psi_{cv}+\psi_{ce}\cdot\psi_{ev}$	0.109879	0.49207	66203.7
589	$\overline{\boldsymbol{u}} \sim \psi_{ac}+\psi_{ce}+\psi_{ev}+\psi_{ac}\cdot\psi_{ev}+\psi_{ac}\cdot\psi_{cv}+\psi_{ce}\cdot\psi_{ev}$	0.109977	0.49207	66204.8
590	$\overline{\boldsymbol{u}} \sim \psi_{ac}+\psi_{ce}+\psi_{ev}+\psi_{ac}\cdot\psi_{ev}+\psi_{ac}\cdot\psi_{cv}$	0.109736	0.49192	66212.4

591	$\overline{\boldsymbol{u}} \sim \psi_{ac}+\psi_{ce}+\psi_{ev}+\psi_{ac}\cdot\psi_{cv}$	0.109582	0.49191	66212.2
592	$\overline{\boldsymbol{u}} \sim \psi_{ac}+\psi_{ev}+\psi_{ac}\cdot\psi_{ev}+\psi_{ce}\cdot\psi_{cv}+\psi_{ev}\cdot\psi_{cv}$	0.126236	0.49158	66232.8
593	$\overline{\boldsymbol{u}} \sim \psi_{ac}+\psi_{ac}\cdot\psi_{ce}+\psi_{ac}\cdot\psi_{cv}+\psi_{ev}\cdot\psi_{cv}$	0.156691	0.49149	66237.4
594	$\overline{\boldsymbol{u}} \sim \psi_{ac}\cdot\psi_{ce}+\psi_{ac}\cdot\psi_{ev}+\psi_{ac}\cdot\psi_{cv}+\psi_{ce}\cdot\psi_{cv}+\psi_{ev}\cdot\psi_{cv}$	0.125522	0.49132	66248.4
595	$\overline{\boldsymbol{u}} \sim \psi_{ac}\cdot\psi_{ev}+\psi_{ac}\cdot\psi_{cv}+\psi_{ce}\cdot\psi_{cv}+\psi_{ev}\cdot\psi_{cv}$	0.122832	0.49108	66261.7
596	$\overline{\boldsymbol{u}} \sim \psi_{ac}+\psi_{ev}+\psi_{ce}\cdot\psi_{cv}+\psi_{ev}\cdot\psi_{cv}$	0.127556	0.48927	66369.2
597	$\overline{\boldsymbol{u}} \sim \psi_{ac}+\psi_{ce}+\psi_{ce}\cdot\psi_{ev}+\psi_{ce}\cdot\psi_{cv}$	0.100115	0.48904	66382.6
598	$\overline{\boldsymbol{u}} \sim \psi_{ac}+\psi_{cv}+\psi_{ce}\cdot\psi_{ev}+\psi_{ce}\cdot\psi_{cv}+\psi_{ev}\cdot\psi_{cv}$	0.123126	0.48839	66421.9
599	$\overline{\boldsymbol{u}} \sim \psi_{ac}+\psi_{cv}+\psi_{ce}\cdot\psi_{cv}+\psi_{ev}\cdot\psi_{cv}$	0.12171	0.48812	66436.9
600	$\overline{\boldsymbol{u}} \sim \psi_{ac}+\psi_{ac}\cdot\psi_{ev}+\psi_{ce}\cdot\psi_{cv}+\psi_{ev}\cdot\psi_{cv}$	0.126878	0.48773	66460.4
601	$\overline{\boldsymbol{u}} \sim \psi_{ac}\cdot\psi_{ce}+\psi_{ac}\cdot\psi_{ev}+\psi_{ac}\cdot\psi_{cv}+\psi_{ev}\cdot\psi_{cv}$	0.134376	0.48735	66482.5
602	$\overline{\boldsymbol{u}} \sim \psi_{ev}+\psi_{cv}+\psi_{ac}\cdot\psi_{ev}+\psi_{ce}\cdot\psi_{ev}+\psi_{ce}\cdot\psi_{cv}+\psi_{ev}\cdot\psi_{cv}$	0.121177	0.48691	66510.6
603	$\overline{\boldsymbol{u}} \sim \psi_{ac}+\psi_{ce}\cdot\psi_{ev}+\psi_{ce}\cdot\psi_{cv}+\psi_{ev}\cdot\psi_{cv}$	0.121397	0.4869	66509.5
604	$\overline{\boldsymbol{u}} \sim \psi_{ce}+\psi_{cv}+\psi_{ac}\cdot\psi_{ce}+\psi_{ac}\cdot\psi_{ev}+\psi_{ce}\cdot\psi_{ev}+\psi_{ev}\cdot\psi_{cv}$	0.0612309	0.48682	66515.7
605	$\overline{\boldsymbol{u}} \sim \psi_{ac}+\psi_{ce}+\psi_{ac}\cdot\psi_{ev}+\psi_{ac}\cdot\psi_{cv}+\psi_{ce}\cdot\psi_{ev}$	0.114594	0.48669	66522.4
606	$\overline{\boldsymbol{u}} \sim \psi_{ac}+\psi_{ce}\cdot\psi_{cv}+\psi_{ev}\cdot\psi_{cv}$	0.123588	0.48625	66546.8
607	$\overline{\boldsymbol{u}} \sim \psi_{ev}+\psi_{cv}+\psi_{ac}\cdot\psi_{ce}+\psi_{ac}\cdot\psi_{ev}+\psi_{ce}\cdot\psi_{ev}+\psi_{ev}\cdot\psi_{cv}$	0.101572	0.48594	66567.7
608	$\overline{\boldsymbol{u}} \sim \psi_{ev}+\psi_{ac}\cdot\psi_{ce}+\psi_{ac}\cdot\psi_{ev}+\psi_{ce}\cdot\psi_{ev}+\psi_{ev}\cdot\psi_{cv}$	0.101476	0.48591	66568.3
609	$\overline{\boldsymbol{u}} \sim \psi_{ac}+\psi_{ce}+\psi_{ac}\cdot\psi_{ev}+\psi_{ac}\cdot\psi_{cv}$	0.115253	0.48556	66588.1
610	$\overline{\boldsymbol{u}} \sim \psi_{ce}+\psi_{ev}+\psi_{ac}\cdot\psi_{ev}+\psi_{ce}\cdot\psi_{ev}+\psi_{ce}\cdot\psi_{cv}$	0.107194	0.48526	66607
611	$\overline{\boldsymbol{u}} \sim \psi_{ev}+\psi_{ac}\cdot\psi_{ce}+\psi_{ac}\cdot\psi_{ev}+\psi_{ce}\cdot\psi_{cv}+\psi_{ev}\cdot\psi_{cv}$	0.11181	0.48512	66615.2
612	$\overline{\boldsymbol{u}} \sim \psi_{ac}+\psi_{ac}\cdot\psi_{ce}+\psi_{ac}\cdot\psi_{ev}+\psi_{ce}\cdot\psi_{ev}+\psi_{ce}\cdot\psi_{cv}$	0.0995808	0.48486	66630.3
613	$\overline{\boldsymbol{u}} \sim \psi_{ev}+\psi_{cv}+\psi_{ac}\cdot\psi_{ev}+\psi_{ce}\cdot\psi_{cv}+\psi_{ev}\cdot\psi_{cv}$	0.124826	0.48417	66670.7
614	$\overline{\boldsymbol{u}} \sim \psi_{ce}+\psi_{ev}+\psi_{ac}\cdot\psi_{ev}+\psi_{ce}\cdot\psi_{cv}$	0.106906	0.48401	66679.4
615	$\overline{\boldsymbol{u}} \sim \psi_{ac}+\psi_{cv}+\psi_{ac}\cdot\psi_{ce}+\psi_{ac}\cdot\psi_{cv}$	0.249763	0.48367	66698.9
616	$\overline{\boldsymbol{u}} \sim \psi_{ac}+\psi_{ev}+\psi_{ac}\cdot\psi_{ev}+\psi_{ce}\cdot\psi_{ev}+\psi_{ce}\cdot\psi_{cv}$	0.106347	0.48289	66745.6
617	$\overline{\boldsymbol{u}} \sim \psi_{ac}+\psi_{ev}+\psi_{ac}\cdot\psi_{ev}+\psi_{ac}\cdot\psi_{cv}+\psi_{ce}\cdot\psi_{ev}+\psi_{ce}\cdot\psi_{cv}$	0.106316	0.48288	66747.4

618	$\overline{\boldsymbol{u}} \sim \psi_{ev}+\psi_{cv}+\psi_{ac}\cdot\psi_{ev}+\psi_{ce}\cdot\psi_{ev}+\psi_{ce}\cdot\psi_{cv}$	0.121376	0.4828	66751
619	$\overline{\boldsymbol{u}} \sim \psi_{ac}+\psi_{ac}\cdot\psi_{ce}+\psi_{ac}\cdot\psi_{ev}+\psi_{ce}\cdot\psi_{ev}$	0.0958327	0.48248	66768.6
620	$\overline{\boldsymbol{u}} \sim \psi_{ev}+\psi_{ac}\cdot\psi_{ev}+\psi_{ce}\cdot\psi_{ev}+\psi_{ce}\cdot\psi_{cv}+\psi_{ev}\cdot\psi_{cv}$	0.118455	0.48245	66771.5
621	$\overline{\boldsymbol{u}} \sim \psi_{ac}+\psi_{ev}+\psi_{ce}\cdot\psi_{ev}+\psi_{ce}\cdot\psi_{cv}$	0.10599	0.48225	66781.9
622	$\overline{\boldsymbol{u}} \sim \psi_{ac}+\psi_{ev}+\psi_{ac}\cdot\psi_{cv}+\psi_{ce}\cdot\psi_{ev}+\psi_{ce}\cdot\psi_{cv}$	0.105932	0.48225	66783.4
623	$\overline{\boldsymbol{u}} \sim \psi_{cv}+\psi_{ac}\cdot\psi_{ce}+\psi_{ac}\cdot\psi_{cv}$	0.19199	0.48154	66822.7
624	$\overline{\boldsymbol{u}} \sim \psi_{ev}+\psi_{cv}+\psi_{ac}\cdot\psi_{ce}+\psi_{ac}\cdot\psi_{ev}+\psi_{ce}\cdot\psi_{ev}$	0.102485	0.48092	66860.7
625	$\overline{\boldsymbol{u}} \sim \psi_{ce}+\psi_{ev}+\psi_{ac}\cdot\psi_{ce}+\psi_{ac}\cdot\psi_{cv}+\psi_{ce}\cdot\psi_{ev}+\psi_{ev}\cdot\psi_{cv}$	0.0745778	0.48043	66890.2
626	$\overline{\boldsymbol{u}} \sim \psi_{ev}+\psi_{cv}+\psi_{ac}\cdot\psi_{ev}+\psi_{ce}\cdot\psi_{cv}$	0.125136	0.47992	66918.1
627	$\overline{\boldsymbol{u}} \sim \psi_{ce}+\psi_{ev}+\psi_{ac}\cdot\psi_{ce}+\psi_{ac}\cdot\psi_{cv}+\psi_{ev}\cdot\psi_{cv}$	0.0744819	0.47983	66924.1
628	$\overline{\boldsymbol{u}} \sim \psi_{ac}+\psi_{cv}+\psi_{ac}\cdot\psi_{cv}+\psi_{ce}\cdot\psi_{ev}$	0.21533	0.4793	66954.4
629	$\overline{\boldsymbol{u}} \sim \psi_{ac}+\psi_{ce}+\psi_{ev}+\psi_{ce}\cdot\psi_{ev}$	0.119747	0.47918	66961.1
630	$\overline{\boldsymbol{u}} \sim \psi_{ac}+\psi_{ce}+\psi_{ev}+\psi_{ac}\cdot\psi_{ev}+\psi_{ce}\cdot\psi_{ev}$	0.119902	0.47918	66961.9
631	$\overline{\boldsymbol{u}} \sim \psi_{ac}+\psi_{ce}+\psi_{ev}+\psi_{ac}\cdot\psi_{ev}$	0.119573	0.47903	66969.9
632	$\overline{\boldsymbol{u}} \sim \psi_{ac}+\psi_{ce}+\psi_{ev}$	0.119344	0.47901	66970.2
633	$\overline{\boldsymbol{u}} \sim \psi_{cv}+\psi_{ac}\cdot\psi_{cv}+\psi_{ce}\cdot\psi_{ev}$	0.160907	0.47641	67120.9
634	$\overline{\boldsymbol{u}} \sim \psi_{ev}+\psi_{cv}+\psi_{ac}\cdot\psi_{ev}+\psi_{ce}\cdot\psi_{ev}+\psi_{ev}\cdot\psi_{cv}$	0.112354	0.47613	67138.7
635	$\overline{\boldsymbol{u}} \sim \psi_{ev}+\psi_{ac}\cdot\psi_{ev}+\psi_{ce}\cdot\psi_{ev}+\psi_{ev}\cdot\psi_{cv}$	0.112416	0.47577	67158.6
636	$\overline{\boldsymbol{u}} \sim \psi_{ac}+\psi_{ev}+\psi_{ac}\cdot\psi_{ev}+\psi_{ac}\cdot\psi_{cv}+\psi_{ce}\cdot\psi_{ev}$	0.108451	0.47558	67170.5
637	$\overline{\boldsymbol{u}} \sim \psi_{ac}+\psi_{ev}+\psi_{ac}\cdot\psi_{cv}+\psi_{ce}\cdot\psi_{ev}$	0.108034	0.4752	67191.6
638	$\overline{\boldsymbol{u}} \sim \psi_{ev}+\psi_{ac}\cdot\psi_{ev}+\psi_{ce}\cdot\psi_{cv}+\psi_{ev}\cdot\psi_{cv}$	0.123411	0.47473	67218.8
639	$\overline{\boldsymbol{u}} \sim \psi_{ac}+\psi_{ce}+\psi_{ac}\cdot\psi_{ev}+\psi_{ce}\cdot\psi_{ev}$	0.125069	0.47399	67261.3
640	$\overline{\boldsymbol{u}} \sim \psi_{ac}+\psi_{ce}+\psi_{ac}\cdot\psi_{ev}$	0.12579	0.47286	67325.1
641	$\overline{\boldsymbol{u}} \sim \psi_{ev}+\psi_{cv}+\psi_{ac}\cdot\psi_{ev}+\psi_{ce}\cdot\psi_{ev}$	0.112667	0.4721	67369.9
642	$\overline{\boldsymbol{u}} \sim \psi_{ac}+\psi_{ev}+\psi_{ac}\cdot\psi_{ev}+\psi_{ac}\cdot\psi_{cv}+\psi_{ev}\cdot\psi_{cv}$	0.134008	0.47196	67378.6
643	$\overline{\boldsymbol{u}} \sim \psi_{ev}+\psi_{ac}\cdot\psi_{ev}+\psi_{ac}\cdot\psi_{cv}+\psi_{ev}\cdot\psi_{cv}$	0.130168	0.46964	67510.1
644	$\overline{\boldsymbol{u}} \sim \psi_{ac}+\psi_{ac}\cdot\psi_{ev}+\psi_{ac}\cdot\psi_{cv}+\psi_{ev}\cdot\psi_{cv}$	0.132905	0.46946	67520.4

645	$\overline{\boldsymbol{u}} \sim \psi_{ac}+\psi_{ev}+\psi_{cv}+\psi_{ac}\cdot\psi_{ce}+\psi_{ac}\cdot\psi_{ev}$	0.131779	0.46926	67532.8
646	$\overline{\boldsymbol{u}} \sim \psi_{ac}+\psi_{ev}+\psi_{cv}+\psi_{ac}\cdot\psi_{ce}+\psi_{ac}\cdot\psi_{ev}+\psi_{ev}\cdot\psi_{cv}$	0.131773	0.46925	67534.8
647	$\overline{\boldsymbol{u}} \sim \psi_{ac}+\psi_{ev}+\psi_{cv}+\psi_{ac}\cdot\psi_{ce}$	0.131511	0.46922	67534.2
648	$\overline{\boldsymbol{u}} \sim \psi_{ac}+\psi_{ev}+\psi_{cv}+\psi_{ac}\cdot\psi_{ce}+\psi_{ev}\cdot\psi_{cv}$	0.131518	0.46922	67535.5
649	$\overline{\boldsymbol{u}} \sim \psi_{ce}+\psi_{ev}+\psi_{ac}\cdot\psi_{cv}+\psi_{ce}\cdot\psi_{ev}+\psi_{ev}\cdot\psi_{cv}$	0.0984722	0.4691	67542.4
650	$\overline{\boldsymbol{u}} \sim \psi_{ce}+\psi_{ev}+\psi_{ac}\cdot\psi_{cv}+\psi_{ev}\cdot\psi_{cv}$	0.0983224	0.46847	67577
651	$\overline{\boldsymbol{u}} \sim \psi_{ac}+\psi_{ac}\cdot\psi_{ev}+\psi_{ac}\cdot\psi_{cv}+\psi_{ce}\cdot\psi_{ev}+\psi_{ce}\cdot\psi_{cv}$	0.117454	0.46814	67596.7
652	$\overline{\boldsymbol{u}} \sim \psi_{ac}+\psi_{ac}\cdot\psi_{ev}+\psi_{ce}\cdot\psi_{ev}+\psi_{ce}\cdot\psi_{cv}$	0.118039	0.46786	67611.5
653	$\overline{\boldsymbol{u}} \sim \psi_{ac}+\psi_{cv}+\psi_{ac}\cdot\psi_{cv}$	0.232566	0.46722	67646.9
654	$\overline{\boldsymbol{u}} \sim \psi_{ce}+\psi_{ac}\cdot\psi_{ce}+\psi_{ac}\cdot\psi_{cv}+\psi_{ce}\cdot\psi_{ev}+\psi_{ev}\cdot\psi_{cv}$	0.0703004	0.46629	67701.7
655	$\overline{\boldsymbol{u}} \sim \psi_{ac}+\psi_{ev}+\psi_{ac}\cdot\psi_{cv}+\psi_{ev}\cdot\psi_{cv}$	0.13437	0.46475	67788.2
656	$\overline{\boldsymbol{u}} \sim \psi_{ac}+\psi_{ac}\cdot\psi_{ev}+\psi_{ac}\cdot\psi_{cv}+\psi_{ce}\cdot\psi_{ev}$	0.117908	0.46379	67842.2
657	$\overline{\boldsymbol{u}} \sim \psi_{cv}+\psi_{ac}\cdot\psi_{cv}$	0.161289	0.46343	67860.8
658	$\overline{\boldsymbol{u}} \sim \psi_{ac}+\psi_{cv}+\psi_{ac}\cdot\psi_{ce}+\psi_{ac}\cdot\psi_{ev}+\psi_{ev}\cdot\psi_{cv}$	0.136057	0.46342	67864.3
659	$\overline{\boldsymbol{u}} \sim \psi_{ac}+\psi_{cv}+\psi_{ac}\cdot\psi_{ce}+\psi_{ac}\cdot\psi_{ev}$	0.136492	0.46335	67867.4
660	$\overline{\boldsymbol{u}} \sim \psi_{ac}+\psi_{ev}+\psi_{ac}\cdot\psi_{ev}+\psi_{ce}\cdot\psi_{ev}$	0.116813	0.4624	67920.6
661	$\overline{\boldsymbol{u}} \sim \psi_{ac}+\psi_{ev}+\psi_{ce}\cdot\psi_{ev}$	0.116273	0.46198	67943.2
662	$\overline{\boldsymbol{u}} \sim \psi_{ac}+\psi_{ev}+\psi_{ac}\cdot\psi_{ce}+\psi_{ac}\cdot\psi_{ev}+\psi_{ev}\cdot\psi_{cv}$	0.131677	0.46146	67974.6
663	$\overline{\boldsymbol{u}} \sim \psi_{ac}+\psi_{ac}\cdot\psi_{cv}+\psi_{ev}\cdot\psi_{cv}$	0.134237	0.46134	67979.1
664	$\overline{\boldsymbol{u}} \sim \psi_{ac}+\psi_{ce}+\psi_{ac}\cdot\psi_{cv}+\psi_{ce}\cdot\psi_{ev}$	0.105271	0.46117	67990
665	$\overline{\boldsymbol{u}} \sim \psi_{ac}+\psi_{ev}+\psi_{ac}\cdot\psi_{ce}+\psi_{ev}\cdot\psi_{cv}$	0.13269	0.46087	68006.7
666	$\overline{\boldsymbol{u}} \sim \psi_{ac}+\psi_{cv}+\psi_{ac}\cdot\psi_{ce}+\psi_{ce}\cdot\psi_{ev}+\psi_{ev}\cdot\psi_{cv}$	0.102905	0.46023	68043.2
667	$\overline{\boldsymbol{u}} \sim \psi_{ac}\cdot\psi_{ce}+\psi_{ac}\cdot\psi_{cv}+\psi_{ce}\cdot\psi_{ev}+\psi_{ce}\cdot\psi_{cv}+\psi_{ev}\cdot\psi_{cv}$	0.105939	0.46001	68056
668	$\overline{\boldsymbol{u}} \sim \psi_{ev}+\psi_{ac}\cdot\psi_{ce}+\psi_{ac}\cdot\psi_{cv}+\psi_{ce}\cdot\psi_{ev}+\psi_{ce}\cdot\psi_{cv}+\psi_{ev}\cdot\psi_{cv}$	0.106184	0.46	68057.4
669	$\overline{\boldsymbol{u}} \sim \psi_{ce}+\psi_{ev}+\psi_{ac}\cdot\psi_{ev}+\psi_{ac}\cdot\psi_{cv}+\psi_{ce}\cdot\psi_{ev}$	0.114428	0.45991	68061.6
670	$\overline{\boldsymbol{u}} \sim \psi_{ce}+\psi_{ac}\cdot\psi_{ce}+\psi_{ac}\cdot\psi_{cv}+\psi_{ev}\cdot\psi_{cv}$	0.0677443	0.45982	68065.4
671	$\overline{\boldsymbol{u}} \sim \psi_{ac}+\psi_{cv}+\psi_{ce}\cdot\psi_{ev}+\psi_{ev}\cdot\psi_{cv}$	0.107166	0.45937	68090.7

672	$\overline{\boldsymbol{u}} \sim \psi_{ce}+\psi_{ac}\cdot\psi_{ce}+\psi_{ac}\cdot\psi_{ev}+\psi_{ce}\cdot\psi_{ev}+\psi_{ce}\cdot\psi_{cv}+\psi_{ev}\cdot\psi_{cv}$	0.0564851	0.45909	68108.1
673	$\overline{\boldsymbol{u}} \sim \psi_{ce}+\psi_{ev}+\psi_{ac}\cdot\psi_{ev}+\psi_{ac}\cdot\psi_{cv}$	0.114052	0.45869	68128.5
674	$\overline{\boldsymbol{u}} \sim \psi_{ce}+\psi_{ac}\cdot\psi_{cv}+\psi_{ce}\cdot\psi_{ev}+\psi_{ev}\cdot\psi_{cv}$	0.0953776	0.45714	68215.3
675	$\overline{\boldsymbol{u}} \sim \psi_{ac}+\psi_{ac}\cdot\psi_{ce}+\psi_{ac}\cdot\psi_{ev}+\psi_{ev}\cdot\psi_{cv}$	0.133551	0.45701	68222.6
676	$\overline{\boldsymbol{u}} \sim \psi_{ev}+\psi_{ac}\cdot\psi_{cv}+\psi_{ce}\cdot\psi_{ev}+\psi_{ce}\cdot\psi_{cv}+\psi_{ev}\cdot\psi_{cv}$	0.0947222	0.45504	68333.2
677	$\overline{\boldsymbol{u}} \sim \psi_{ac}\cdot\psi_{cv}+\psi_{ce}\cdot\psi_{ev}+\psi_{ce}\cdot\psi_{cv}+\psi_{ev}\cdot\psi_{cv}$	0.0949693	0.45503	68332.4
678	$\overline{\boldsymbol{u}} \sim \psi_{ce}+\psi_{ev}+\psi_{ac}\cdot\psi_{ev}+\psi_{ce}\cdot\psi_{ev}$	0.116909	0.45477	68346.8
679	$\overline{\boldsymbol{u}} \sim \psi_{ce}+\psi_{ev}+\psi_{ac}\cdot\psi_{ev}$	0.116365	0.4537	68405.4
680	$\overline{\boldsymbol{u}} \sim \psi_{ac}\cdot\psi_{ev}+\psi_{ac}\cdot\psi_{cv}+\psi_{ev}\cdot\psi_{cv}$	0.113037	0.45343	68420.5
681	$\overline{\boldsymbol{u}} \sim \psi_{ce}+\psi_{ac}\cdot\psi_{cv}+\psi_{ev}\cdot\psi_{cv}$	0.0948745	0.45208	68494.9
682	$\overline{\boldsymbol{u}} \sim \psi_{ev}+\psi_{ac}\cdot\psi_{ce}+\psi_{ac}\cdot\psi_{cv}+\psi_{ce}\cdot\psi_{ev}+\psi_{ev}\cdot\psi_{cv}$	0.102086	0.45202	68500.4
683	$\overline{\boldsymbol{u}} \sim \psi_{ev}+\psi_{ac}\cdot\psi_{cv}+\psi_{ce}\cdot\psi_{ev}+\psi_{ev}\cdot\psi_{cv}$	0.0975537	0.45135	68536.2
684	$\overline{\boldsymbol{u}} \sim \psi_{ac}+\psi_{ac}\cdot\psi_{ev}+\psi_{ce}\cdot\psi_{ev}$	0.127416	0.45088	68561.3
685	$\overline{\boldsymbol{u}} \sim \psi_{ac}\cdot\psi_{ce}+\psi_{ac}\cdot\psi_{cv}+\psi_{ce}\cdot\psi_{ev}+\psi_{ev}\cdot\psi_{cv}$	0.09772	0.45016	68602.1
686	$\overline{\boldsymbol{u}} \sim \psi_{ac}\cdot\psi_{cv}+\psi_{ce}\cdot\psi_{ev}+\psi_{ev}\cdot\psi_{cv}$	0.0965135	0.45011	68603.7
687	$\overline{\boldsymbol{u}} \sim \psi_{ev}+\psi_{ac}\cdot\psi_{ce}+\psi_{ac}\cdot\psi_{ev}+\psi_{ac}\cdot\psi_{cv}+\psi_{ce}\cdot\psi_{ev}+\psi_{ce}\cdot\psi_{cv}$	0.0991338	0.44941	68645.3
688	$\overline{\boldsymbol{u}} \sim \psi_{ev}+\psi_{ac}\cdot\psi_{ce}+\psi_{ac}\cdot\psi_{ev}+\psi_{ac}\cdot\psi_{cv}+\psi_{ce}\cdot\psi_{ev}$	0.0985306	0.4493	68650.2
689	$\overline{\boldsymbol{u}} \sim \psi_{ev}+\psi_{ac}\cdot\psi_{ce}+\psi_{ac}\cdot\psi_{ev}+\psi_{ce}\cdot\psi_{ev}+\psi_{ce}\cdot\psi_{cv}$	0.0995994	0.44917	68657.2
690	$\overline{\boldsymbol{u}} \sim \psi_{ac}+\psi_{ce}+\psi_{ce}\cdot\psi_{ev}$	0.111836	0.44832	68701.8
691	$\overline{\boldsymbol{u}} \sim \psi_{ev}+\psi_{ac}\cdot\psi_{ce}+\psi_{ac}\cdot\psi_{ev}+\psi_{ce}\cdot\psi_{ev}$	0.0963382	0.44688	68781.6
692	$\overline{\boldsymbol{u}} \sim \psi_{ev}+\psi_{ac}\cdot\psi_{ce}+\psi_{ac}\cdot\psi_{cv}+\psi_{ce}\cdot\psi_{cv}+\psi_{ev}\cdot\psi_{cv}$	0.124801	0.44442	68917
693	$\overline{\boldsymbol{u}} \sim \psi_{ev}+\psi_{ac}\cdot\psi_{ev}+\psi_{ac}\cdot\psi_{cv}+\psi_{ce}\cdot\psi_{ev}+\psi_{ce}\cdot\psi_{cv}$	0.108789	0.44341	68971.8
694	$\overline{\boldsymbol{u}} \sim \psi_{ac}+\psi_{cv}+\psi_{ac}\cdot\psi_{ce}+\psi_{ev}\cdot\psi_{cv}$	0.122859	0.44312	68987.1
695	$\overline{\boldsymbol{u}} \sim \psi_{ev}+\psi_{ac}\cdot\psi_{ev}+\psi_{ce}\cdot\psi_{ev}+\psi_{ce}\cdot\psi_{cv}$	0.109664	0.44264	69012.9
696	$\overline{\boldsymbol{u}} \sim \psi_{ev}+\psi_{cv}+\psi_{ac}\cdot\psi_{ce}+\psi_{ac}\cdot\psi_{ev}+\psi_{ev}\cdot\psi_{cv}$	0.127907	0.44241	69026.2
697	$\overline{\boldsymbol{u}} \sim \psi_{ac}+\psi_{cv}+\psi_{ac}\cdot\psi_{ce}+\psi_{ce}\cdot\psi_{ev}+\psi_{ce}\cdot\psi_{cv}$	0.102576	0.44166	69067.3
698	$\overline{\boldsymbol{u}} \sim \psi_{ev}+\psi_{ac}\cdot\psi_{ce}+\psi_{ac}\cdot\psi_{cv}+\psi_{ev}\cdot\psi_{cv}$	0.11929	0.44152	69073.6

699	$\overline{\boldsymbol{u}} \sim \psi_{ev}+\psi_{ac}\cdot\psi_{ce}+\psi_{ac}\cdot\psi_{ev}+\psi_{ev}\cdot\psi_{cv}$	0.128053	0.44148	69075.6
700	$\overline{\boldsymbol{u}} \sim \psi_{ev}+\psi_{cv}+\psi_{ac}\cdot\psi_{ce}+\psi_{ac}\cdot\psi_{ev}$	0.128415	0.43889	69215.9
701	$\overline{\boldsymbol{u}} \sim \psi_{ac}+\psi_{ev}+\psi_{cv}+\psi_{ac}\cdot\psi_{ev}$	0.106002	0.43717	69308.4
702	$\overline{\boldsymbol{u}} \sim \psi_{ac}+\psi_{ev}+\psi_{cv}+\psi_{ac}\cdot\psi_{ev}+\psi_{ev}\cdot\psi_{cv}$	0.105994	0.43715	69310.4
703	$\overline{\boldsymbol{u}} \sim \psi_{ac}+\psi_{ev}+\psi_{cv}$	0.105753	0.43714	69309.1
704	$\overline{\boldsymbol{u}} \sim \psi_{ac}+\psi_{ev}+\psi_{cv}+\psi_{ev}\cdot\psi_{cv}$	0.105759	0.43713	69310.6
705	$\overline{\boldsymbol{u}} \sim \psi_{ac}+\psi_{ac}\cdot\psi_{ce}+\psi_{ce}\cdot\psi_{ev}+\psi_{ev}\cdot\psi_{cv}$	0.108478	0.43664	69336.9
706	$\overline{\boldsymbol{u}} \sim \psi_{ev}+\psi_{ac}\cdot\psi_{ev}+\psi_{ac}\cdot\psi_{cv}+\psi_{ce}\cdot\psi_{ev}$	0.113396	0.43622	69359.2
707	$\overline{\boldsymbol{u}} \sim \psi_{ac}+\psi_{ac}\cdot\psi_{ce}+\psi_{ev}\cdot\psi_{cv}$	0.114699	0.4356	69391.7
708	$\overline{\boldsymbol{u}} \sim \psi_{ac}\cdot\psi_{ce}+\psi_{ac}\cdot\psi_{cv}+\psi_{ce}\cdot\psi_{cv}+\psi_{ev}\cdot\psi_{cv}$	0.131007	0.4339	69483.9
709	$\overline{\boldsymbol{u}} \sim \psi_{ev}+\psi_{ac}\cdot\psi_{ev}+\psi_{ce}\cdot\psi_{ev}$	0.11499	0.43162	69604.2
710	$\overline{\boldsymbol{u}} \sim \psi_{ac}+\psi_{cv}+\psi_{ac}\cdot\psi_{ev}+\psi_{ev}\cdot\psi_{cv}$	0.111798	0.43115	69630.2
711	$\overline{\boldsymbol{u}} \sim \psi_{ac}+\psi_{cv}+\psi_{ac}\cdot\psi_{ev}$	0.112305	0.43107	69633.7
712	$\overline{\boldsymbol{u}} \sim \psi_{ac}+\psi_{ce}\cdot\psi_{ev}+\psi_{ev}\cdot\psi_{cv}$	0.09279	0.43041	69668.8
713	$\overline{\boldsymbol{u}} \sim \psi_{ac}+\psi_{ev}+\psi_{ac}\cdot\psi_{ev}+\psi_{ac}\cdot\psi_{cv}+\psi_{ce}\cdot\psi_{cv}$	0.114139	0.42955	69716
714	$\overline{\boldsymbol{u}} \sim \psi_{ac}+\psi_{ev}+\psi_{ac}\cdot\psi_{ce}+\psi_{ac}\cdot\psi_{ev}+\psi_{ac}\cdot\psi_{cv}+\psi_{ce}\cdot\psi_{cv}$	0.113703	0.42954	69717.9
715	$\overline{\boldsymbol{u}} \sim \psi_{ac}+\psi_{ev}+\psi_{ac}\cdot\psi_{cv}+\psi_{ce}\cdot\psi_{cv}$	0.113877	0.4295	69718.1
716	$\overline{\boldsymbol{u}} \sim \psi_{ac}+\psi_{ev}+\psi_{ac}\cdot\psi_{ce}+\psi_{ac}\cdot\psi_{cv}+\psi_{ce}\cdot\psi_{cv}$	0.113403	0.42948	69719.9
717	$\overline{\boldsymbol{u}} \sim \psi_{ac}+\psi_{ev}+\psi_{ac}\cdot\psi_{ev}+\psi_{ev}\cdot\psi_{cv}$	0.107254	0.42898	69745.8
718	$\overline{\boldsymbol{u}} \sim \psi_{ac}+\psi_{ev}+\psi_{ev}\cdot\psi_{cv}$	0.10846	0.42832	69779.6
719	$\overline{\boldsymbol{u}} \sim \psi_{ac}+\psi_{cv}+\psi_{ce}\cdot\psi_{ev}+\psi_{ce}\cdot\psi_{cv}$	0.122458	0.42795	69799.9
720	$\overline{\boldsymbol{u}} \sim \psi_{ac}\cdot\psi_{ce}+\psi_{ac}\cdot\psi_{cv}+\psi_{ev}\cdot\psi_{cv}$	0.124916	0.42627	69887.6
721	$\overline{\boldsymbol{u}} \sim \psi_{ac}+\psi_{ac}\cdot\psi_{ev}+\psi_{ev}\cdot\psi_{cv}$	0.110254	0.42449	69981.6
722	$\overline{\boldsymbol{u}} \sim \psi_{ac}+\psi_{ev}+\psi_{ac}\cdot\psi_{ce}+\psi_{ac}\cdot\psi_{ev}+\psi_{ac}\cdot\psi_{cv}$	0.132029	0.42393	70013.1
723	$\overline{\boldsymbol{u}} \sim \psi_{ac}+\psi_{ev}+\psi_{ac}\cdot\psi_{ce}+\psi_{ac}\cdot\psi_{cv}$	0.131672	0.42384	70016.5
724	$\overline{\boldsymbol{u}} \sim \psi_{ac}+\psi_{ac}\cdot\psi_{ev}+\psi_{ac}\cdot\psi_{cv}+\psi_{ce}\cdot\psi_{cv}$	0.118924	0.42375	70021.4
725	$\overline{\boldsymbol{u}} \sim \psi_{ac}+\psi_{ac}\cdot\psi_{ce}+\psi_{ac}\cdot\psi_{ev}+\psi_{ac}\cdot\psi_{cv}+\psi_{ce}\cdot\psi_{cv}$	0.119014	0.42373	70023.4

726	$\overline{\boldsymbol{u}} \sim \psi_{ce}+\psi_{cv}+\psi_{ac}\cdot\psi_{ev}+\psi_{ce}\cdot\psi_{ev}+\psi_{ce}\cdot\psi_{cv}+\psi_{ev}\cdot\psi_{cv}$	0.0911699	0.42139	70146.9
727	$\overline{\boldsymbol{u}} \sim \psi_{ce}+\psi_{cv}+\psi_{ac}\cdot\psi_{ev}+\psi_{ce}\cdot\psi_{ev}+\psi_{ev}\cdot\psi_{cv}$	0.0896067	0.42093	70170.2
728	$\overline{\boldsymbol{u}} \sim \psi_{ac}+\psi_{ac}\cdot\psi_{ce}+\psi_{ac}\cdot\psi_{ev}+\psi_{ac}\cdot\psi_{cv}$	0.13644	0.41839	70301.5
729	$\overline{\boldsymbol{u}} \sim \psi_{ev}+\psi_{ac}\cdot\psi_{cv}+\psi_{ce}\cdot\psi_{cv}+\psi_{ev}\cdot\psi_{cv}$	0.102075	0.41753	70346.3
730	$\overline{\boldsymbol{u}} \sim \psi_{ac}+\psi_{ev}+\psi_{ac}\cdot\psi_{ce}+\psi_{ac}\cdot\psi_{ev}+\psi_{ce}\cdot\psi_{cv}$	0.14715	0.41471	70493.4
731	$\overline{\boldsymbol{u}} \sim \psi_{ac}+\psi_{ev}+\psi_{ac}\cdot\psi_{ce}+\psi_{ce}\cdot\psi_{cv}$	0.146714	0.41461	70497.8
732	$\overline{\boldsymbol{u}} \sim \psi_{ac}+\psi_{cv}+\psi_{ev}\cdot\psi_{cv}$	0.101771	0.41131	70666.9
733	$\overline{\boldsymbol{u}} \sim \psi_{ac}+\psi_{ev}+\psi_{ac}\cdot\psi_{ce}+\psi_{ac}\cdot\psi_{ev}$	0.149695	0.40984	70742.9
734	$\overline{\boldsymbol{u}} \sim \psi_{ce}+\psi_{ev}+\psi_{ac}\cdot\psi_{ce}+\psi_{ac}\cdot\psi_{cv}+\psi_{ce}\cdot\psi_{ev}+\psi_{ce}\cdot\psi_{cv}$	0.0750587	0.40976	70749.5
735	$\overline{\boldsymbol{u}} \sim \psi_{ac}+\psi_{ev}+\psi_{ac}\cdot\psi_{ce}$	0.149187	0.40974	70747.4
736	$\overline{\boldsymbol{u}} \sim \psi_{ce}+\psi_{ev}+\psi_{ac}\cdot\psi_{cv}+\psi_{ce}\cdot\psi_{ev}+\psi_{ce}\cdot\psi_{cv}$	0.0761395	0.40973	70749.8
737	$\overline{\boldsymbol{u}} \sim \psi_{ce}+\psi_{ev}+\psi_{ac}\cdot\psi_{ce}+\psi_{ac}\cdot\psi_{cv}+\psi_{ce}\cdot\psi_{cv}$	0.0748001	0.40956	70758.6
738	$\overline{\boldsymbol{u}} \sim \psi_{ce}+\psi_{ev}+\psi_{ac}\cdot\psi_{cv}+\psi_{ce}\cdot\psi_{cv}$	0.0759023	0.40953	70759.1
739	$\overline{\boldsymbol{u}} \sim \psi_{ac}+\psi_{ac}\cdot\psi_{ce}+\psi_{ac}\cdot\psi_{ev}+\psi_{ce}\cdot\psi_{cv}$	0.150969	0.40937	70767.2
740	$\overline{\boldsymbol{u}} \sim \psi_{ce}+\psi_{ac}\cdot\psi_{ev}+\psi_{ce}\cdot\psi_{ev}+\psi_{ce}\cdot\psi_{cv}+\psi_{ev}\cdot\psi_{cv}$	0.0947112	0.40546	70967.7
741	$\overline{\boldsymbol{u}} \sim \psi_{ac}+\psi_{ev}\cdot\psi_{cv}$	0.0937237	0.40484	70996.6
742	$\overline{\boldsymbol{u}} \sim \psi_{ac}+\psi_{ac}\cdot\psi_{ce}+\psi_{ac}\cdot\psi_{ev}$	0.153909	0.40452	71013.8
743	$\overline{\boldsymbol{u}} \sim \psi_{ce}+\psi_{ac}\cdot\psi_{ce}+\psi_{ac}\cdot\psi_{ev}+\psi_{ac}\cdot\psi_{cv}+\psi_{ce}\cdot\psi_{ev}+\psi_{ce}\cdot\psi_{cv}$	0.0689211	0.40048	71221.3
744	$\overline{\boldsymbol{u}} \sim \psi_{ce}+\psi_{cv}+\psi_{ac}\cdot\psi_{ce}+\psi_{ac}\cdot\psi_{ev}+\psi_{ce}\cdot\psi_{cv}+\psi_{ev}\cdot\psi_{cv}$	0.0586585	0.39908	71291.6
745	$\overline{\boldsymbol{u}} \sim \psi_{ev}+\psi_{cv}+\psi_{ac}\cdot\psi_{ev}+\psi_{ev}\cdot\psi_{cv}$	0.106906	0.39854	71316.8
746	$\overline{\boldsymbol{u}} \sim \psi_{ev}+\psi_{ac}\cdot\psi_{ev}+\psi_{ev}\cdot\psi_{cv}$	0.106993	0.39818	71334
747	$\overline{\boldsymbol{u}} \sim \psi_{ev}+\psi_{ac}\cdot\psi_{ce}+\psi_{ac}\cdot\psi_{ev}+\psi_{ac}\cdot\psi_{cv}+\psi_{ce}\cdot\psi_{cv}$	0.133529	0.39672	71409.4
748	$\overline{\boldsymbol{u}} \sim \psi_{ev}+\psi_{ac}\cdot\psi_{ce}+\psi_{ac}\cdot\psi_{ev}+\psi_{ac}\cdot\psi_{cv}$	0.134353	0.39669	71410.1
749	$\overline{\boldsymbol{u}} \sim \psi_{ev}+\psi_{ac}\cdot\psi_{ce}+\psi_{ac}\cdot\psi_{ev}+\psi_{ce}\cdot\psi_{cv}$	0.140296	0.39441	71524
750	$\overline{\boldsymbol{u}} \sim \psi_{ev}+\psi_{cv}+\psi_{ac}\cdot\psi_{ev}$	0.1082	0.3933	71578.4
751	$\overline{\boldsymbol{u}} \sim \psi_{ce}+\psi_{ac}\cdot\psi_{ev}+\psi_{ac}\cdot\psi_{cv}+\psi_{ce}\cdot\psi_{ev}+\psi_{ce}\cdot\psi_{cv}$	0.0805546	0.3929	71600.3
752	$\overline{\boldsymbol{u}} \sim \psi_{ce}+\psi_{cv}+\psi_{ac}\cdot\psi_{ce}+\psi_{ac}\cdot\psi_{ev}+\psi_{ev}\cdot\psi_{cv}$	0.0626892	0.39057	71716.4

753	$\overline{\boldsymbol{u}} \sim \psi_{ev}+\psi_{ac}\cdot\psi_{ev}+\psi_{ac}\cdot\psi_{cv}+\psi_{ce}\cdot\psi_{cv}$	0.122384	0.39019	71734.5
754	$\overline{\boldsymbol{u}} \sim \psi_{ev}+\psi_{ac}\cdot\psi_{ce}+\psi_{ac}\cdot\psi_{ev}$	0.142134	0.38996	71744.8
755	$\overline{\boldsymbol{u}} \sim \psi_{ac}\cdot\psi_{cv}+\psi_{ce}\cdot\psi_{cv}+\psi_{ev}\cdot\psi_{cv}$	0.105557	0.38898	71793
756	$\overline{\boldsymbol{u}} \sim \psi_{ac}+\psi_{ev}+\psi_{ac}\cdot\psi_{ev}+\psi_{ac}\cdot\psi_{cv}$	0.108256	0.38883	71801.6
757	$\overline{\boldsymbol{u}} \sim \psi_{ac}+\psi_{ev}+\psi_{ac}\cdot\psi_{cv}$	0.107883	0.38876	71804.2
758	$\overline{\boldsymbol{u}} \sim \psi_{ce}+\psi_{ac}\cdot\psi_{ce}+\psi_{ac}\cdot\psi_{ev}+\psi_{ce}\cdot\psi_{ev}+\psi_{ev}\cdot\psi_{cv}$	0.070916	0.38565	71959.6
759	$\overline{\boldsymbol{u}} \sim \psi_{ac}+\psi_{ev}+\psi_{ac}\cdot\psi_{ev}+\psi_{ce}\cdot\psi_{cv}$	0.132703	0.38417	72031.6
760	$\overline{\boldsymbol{u}} \sim \psi_{ac}+\psi_{ev}+\psi_{ce}\cdot\psi_{cv}$	0.13204	0.38407	72035.5
761	$\overline{\boldsymbol{u}} \sim \psi_{ac}+\psi_{ac}\cdot\psi_{ev}+\psi_{ac}\cdot\psi_{cv}$	0.11448	0.38314	72080.9
762	$\overline{\boldsymbol{u}} \sim \psi_{ac}+\psi_{cv}+\psi_{ac}\cdot\psi_{ce}+\psi_{ce}\cdot\psi_{cv}$	0.0651826	0.38274	72101.6
763	$\overline{\boldsymbol{u}} \sim \psi_{ev}+\psi_{ac}\cdot\psi_{cv}+\psi_{ev}\cdot\psi_{cv}$	0.0890312	0.38066	72202.4
764	$\overline{\boldsymbol{u}} \sim \psi_{ce}+\psi_{ac}\cdot\psi_{ce}+\psi_{ac}\cdot\psi_{cv}+\psi_{ce}\cdot\psi_{ev}+\psi_{ce}\cdot\psi_{cv}$	0.0769883	0.37987	72243.2
765	$\overline{\boldsymbol{u}} \sim \psi_{ce}+\psi_{ac}\cdot\psi_{cv}+\psi_{ce}\cdot\psi_{ev}+\psi_{ce}\cdot\psi_{cv}$	0.077768	0.37986	72242.6
766	$\overline{\boldsymbol{u}} \sim \psi_{ac}+\psi_{ac}\cdot\psi_{ev}+\psi_{ce}\cdot\psi_{cv}$	0.139952	0.37879	72293.8
767	$\overline{\boldsymbol{u}} \sim \psi_{ce}+\psi_{ac}\cdot\psi_{ev}+\psi_{ce}\cdot\psi_{ev}+\psi_{ev}\cdot\psi_{cv}$	0.0835137	0.37848	72309.8
768	$\overline{\boldsymbol{u}} \sim \psi_{ac}+\psi_{ev}+\psi_{ac}\cdot\psi_{ev}$	0.120935	0.37456	72498.8
769	$\overline{\boldsymbol{u}} \sim \psi_{ac}+\psi_{ev}$	0.120355	0.37447	72502.4
770	$\overline{\boldsymbol{u}} \sim \psi_{cv}+\psi_{ac}\cdot\psi_{ce}+\psi_{ac}\cdot\psi_{ev}+\psi_{ce}\cdot\psi_{ev}+\psi_{ce}\cdot\psi_{cv}+\psi_{ev}\cdot\psi_{cv}$	0.100709	0.37256	72598.8
771	$\overline{\boldsymbol{u}} \sim \psi_{cv}+\psi_{ac}\cdot\psi_{ev}+\psi_{ce}\cdot\psi_{ev}+\psi_{ce}\cdot\psi_{cv}+\psi_{ev}\cdot\psi_{cv}$	0.101936	0.37252	72599.7
772	$\overline{\boldsymbol{u}} \sim \psi_{ac}\cdot\psi_{ce}+\psi_{ac}\cdot\psi_{ev}+\psi_{ce}\cdot\psi_{ev}+\psi_{ce}\cdot\psi_{cv}+\psi_{ev}\cdot\psi_{cv}$	0.100953	0.36954	72742.9
773	$\overline{\boldsymbol{u}} \sim \psi_{ac}\cdot\psi_{ev}+\psi_{ce}\cdot\psi_{ev}+\psi_{ce}\cdot\psi_{cv}+\psi_{ev}\cdot\psi_{cv}$	0.102732	0.36944	72746.6
774	$\overline{\boldsymbol{u}} \sim \psi_{ac}+\psi_{ac}\cdot\psi_{ev}$	0.1284	0.36912	72760.3
775	$\overline{\boldsymbol{u}} \sim \psi_{ce}+\psi_{cv}+\psi_{ac}\cdot\psi_{ev}+\psi_{ce}\cdot\psi_{cv}+\psi_{ev}\cdot\psi_{cv}$	0.0887761	0.36729	72850.8
776	$\overline{\boldsymbol{u}} \sim \psi_{ce}+\psi_{cv}+\psi_{ac}\cdot\psi_{ev}+\psi_{ev}\cdot\psi_{cv}$	0.0864609	0.36594	72914.3
777	$\overline{\boldsymbol{u}} \sim \psi_{cv}+\psi_{ac}\cdot\psi_{ce}+\psi_{ac}\cdot\psi_{ev}+\psi_{ce}\cdot\psi_{cv}+\psi_{ev}\cdot\psi_{cv}$	0.0906417	0.3606	73168.9
778	$\overline{\boldsymbol{u}} \sim \psi_{ev}+\psi_{ac}\cdot\psi_{ev}+\psi_{ce}\cdot\psi_{cv}$	0.128089	0.35872	73255.8
779	$\overline{\boldsymbol{u}} \sim \psi_{cv}+\psi_{ac}\cdot\psi_{ev}+\psi_{ce}\cdot\psi_{cv}+\psi_{ev}\cdot\psi_{cv}$	0.0958147	0.35862	73261.4

780	$\overline{\boldsymbol{u}} \sim \psi_{ev}+\psi_{ac}\cdot\psi_{ce}+\psi_{ac}\cdot\psi_{cv}+\psi_{ce}\cdot\psi_{ev}+\psi_{ce}\cdot\psi_{cv}$	0.111023	0.35741	73319.6
781	$\overline{\boldsymbol{u}} \sim \psi_{ac}+\psi_{ce}+\psi_{ac}\cdot\psi_{cv}+\psi_{ce}\cdot\psi_{cv}$	0.0645306	0.35547	73409.6
782	$\overline{\boldsymbol{u}} \sim \psi_{ac}+\psi_{ce}+\psi_{cv}+\psi_{ce}\cdot\psi_{cv}$	0.0644059	0.35101	73618.3
783	$\overline{\boldsymbol{u}} \sim \psi_{ac}\cdot\psi_{cv}+\psi_{ev}\cdot\psi_{cv}$	0.0835527	0.35086	73623.3
784	$\overline{\boldsymbol{u}} \sim \psi_{ac}+\psi_{ce}+\psi_{cv}$	0.06638	0.35073	73630.7
785	$\overline{\boldsymbol{u}} \sim \psi_{ev}+\psi_{ac}\cdot\psi_{ev}+\psi_{ac}\cdot\psi_{cv}$	0.114968	0.34886	73717.4
786	$\overline{\boldsymbol{u}} \sim \psi_{ce}+\psi_{ac}\cdot\psi_{ce}+\psi_{ac}\cdot\psi_{ev}+\psi_{ac}\cdot\psi_{cv}+\psi_{ce}\cdot\psi_{ev}$	0.0545419	0.34784	73766.8
787	$\overline{\boldsymbol{u}} \sim \psi_{ac}+\psi_{cv}+\psi_{ac}\cdot\psi_{ce}+\psi_{ce}\cdot\psi_{ev}$	0.133178	0.34582	73859.7
788	$\overline{\boldsymbol{u}} \sim \psi_{ev}+\psi_{ac}\cdot\psi_{ev}$	0.11801	0.34364	73958.1
789	$\overline{\boldsymbol{u}} \sim \psi_{ce}+\psi_{cv}+\psi_{ac}\cdot\psi_{ce}+\psi_{ac}\cdot\psi_{ev}+\psi_{ce}\cdot\psi_{ev}+\psi_{ce}\cdot\psi_{cv}$	0.0768234	0.34355	73966.2
790	$\overline{\boldsymbol{u}} \sim \psi_{ac}+\psi_{ce}+\psi_{ce}\cdot\psi_{cv}$	0.0586451	0.34311	73983.4
791	$\overline{\boldsymbol{u}} \sim \psi_{ce}+\psi_{cv}+\psi_{ac}\cdot\psi_{ce}+\psi_{ac}\cdot\psi_{ev}+\psi_{ce}\cdot\psi_{ev}$	0.0797765	0.33796	74222
792	$\overline{\boldsymbol{u}} \sim \psi_{ev}+\psi_{ac}\cdot\psi_{ce}+\psi_{ac}\cdot\psi_{cv}+\psi_{ce}\cdot\psi_{cv}$	0.139657	0.33794	74221.8
793	$\overline{\boldsymbol{u}} \sim \psi_{cv}+\psi_{ac}\cdot\psi_{ce}+\psi_{ac}\cdot\psi_{ev}+\psi_{ce}\cdot\psi_{ev}+\psi_{ev}\cdot\psi_{cv}$	0.11463	0.33671	74279.2
794	$\overline{\boldsymbol{u}} \sim \psi_{ce}+\psi_{cv}+\psi_{ac}\cdot\psi_{ev}+\psi_{ce}\cdot\psi_{ev}+\psi_{ce}\cdot\psi_{cv}$	0.095523	0.33549	74334.4
795	$\overline{\boldsymbol{u}} \sim \psi_{ce}+\psi_{ac}\cdot\psi_{ce}+\psi_{ac}\cdot\psi_{ev}+\psi_{ce}\cdot\psi_{cv}+\psi_{ev}\cdot\psi_{cv}$	0.071387	0.3344	74384.1
796	$\overline{\boldsymbol{u}} \sim \psi_{ac}+\psi_{cv}+\psi_{ce}\cdot\psi_{cv}$	0.0820102	0.33417	74392.4
797	$\overline{\boldsymbol{u}} \sim \psi_{ac}\cdot\psi_{ce}+\psi_{ac}\cdot\psi_{ev}+\psi_{ce}\cdot\psi_{cv}+\psi_{ev}\cdot\psi_{cv}$	0.0773754	0.33393	74404.6
798	$\overline{\boldsymbol{u}} \sim \psi_{ce}+\psi_{ac}\cdot\psi_{ce}+\psi_{ac}\cdot\psi_{ev}+\psi_{ce}\cdot\psi_{ev}+\psi_{ce}\cdot\psi_{cv}$	0.0773345	0.33331	74433.6
799	$\overline{\boldsymbol{u}} \sim \psi_{ce}+\psi_{cv}+\psi_{ac}\cdot\psi_{ev}+\psi_{ce}\cdot\psi_{ev}$	0.0927915	0.33309	74442.8
800	$\overline{\boldsymbol{u}} \sim \psi_{cv}+\psi_{ac}\cdot\psi_{ce}+\psi_{ac}\cdot\psi_{ev}+\psi_{ev}\cdot\psi_{cv}$	0.107092	0.33303	74445.5
801	$\overline{\boldsymbol{u}} \sim \psi_{ce}+\psi_{ac}\cdot\psi_{ce}+\psi_{ac}\cdot\psi_{ev}+\psi_{ce}\cdot\psi_{ev}$	0.0746062	0.33257	74466.4
802	$\overline{\boldsymbol{u}} \sim \psi_{ce}+\psi_{ev}+\psi_{ac}\cdot\psi_{ce}+\psi_{ac}\cdot\psi_{cv}+\psi_{ce}\cdot\psi_{ev}$	0.0968845	0.33225	74481.6
803	$\overline{\boldsymbol{u}} \sim \psi_{ce}+\psi_{ev}+\psi_{ac}\cdot\psi_{ce}+\psi_{ac}\cdot\psi_{cv}$	0.0947911	0.33193	74495
804	$\overline{\boldsymbol{u}} \sim \psi_{ce}+\psi_{ev}+\psi_{ac}\cdot\psi_{cv}+\psi_{ce}\cdot\psi_{ev}$	0.119318	0.33123	74526.8
805	$\overline{\boldsymbol{u}} \sim \psi_{ce}+\psi_{ev}+\psi_{ac}\cdot\psi_{cv}$	0.117696	0.33088	74541.8
806	$\overline{\boldsymbol{u}} \sim \psi_{ev}+\psi_{ac}\cdot\psi_{cv}+\psi_{ce}\cdot\psi_{ev}+\psi_{ce}\cdot\psi_{cv}$	0.0863129	0.32746	74697.2

807	$\overline{\boldsymbol{u}} \sim \psi_{ce}+\psi_{ac}\cdot\psi_{ev}+\psi_{ce}\cdot\psi_{ev}+\psi_{ce}\cdot\psi_{cv}$	0.0926289	0.32691	74721.9
808	$\overline{\boldsymbol{u}} \sim \psi_{ce}+\psi_{ac}\cdot\psi_{ev}+\psi_{ce}\cdot\psi_{cv}+\psi_{ev}\cdot\psi_{cv}$	0.0962857	0.3267	74731.1
809	$\overline{\boldsymbol{u}} \sim \psi_{ce}+\psi_{ac}\cdot\psi_{ev}+\psi_{ac}\cdot\psi_{cv}+\psi_{ce}\cdot\psi_{ev}$	0.0949506	0.32525	74796.2
810	$\overline{\boldsymbol{u}} \sim \psi_{ce}+\psi_{ev}+\psi_{cv}+\psi_{ac}\cdot\psi_{ce}+\psi_{ce}\cdot\psi_{ev}+\psi_{ce}\cdot\psi_{cv}$	0.163455	0.32503	74808.3
811	$\overline{\boldsymbol{u}} \sim \psi_{ce}+\psi_{ev}+\psi_{cv}+\psi_{ac}\cdot\psi_{ce}+\psi_{ce}\cdot\psi_{ev}+\psi_{ce}\cdot\psi_{cv}+\psi_{ev}\cdot\psi_{cv}$	0.163489	0.32502	74809.6
812	$\overline{\boldsymbol{u}} \sim \psi_{ce}+\psi_{ev}+\psi_{cv}+\psi_{ac}\cdot\psi_{ce}+\psi_{ce}\cdot\psi_{cv}$	0.162134	0.32459	74827
813	$\overline{\boldsymbol{u}} \sim \psi_{ce}+\psi_{ev}+\psi_{cv}+\psi_{ac}\cdot\psi_{ce}+\psi_{ce}\cdot\psi_{cv}+\psi_{ev}\cdot\psi_{cv}$	0.162161	0.32458	74828.5
814	$\overline{\boldsymbol{u}} \sim \psi_{ev}+\psi_{cv}+\psi_{ac}\cdot\psi_{ce}+\psi_{ce}\cdot\psi_{ev}+\psi_{ce}\cdot\psi_{cv}$	0.161073	0.32451	74830.4
815	$\overline{\boldsymbol{u}} \sim \psi_{ev}+\psi_{cv}+\psi_{ac}\cdot\psi_{ce}+\psi_{ce}\cdot\psi_{ev}+\psi_{ce}\cdot\psi_{cv}+\psi_{ev}\cdot\psi_{cv}$	0.161099	0.3245	74831.7
816	$\overline{\boldsymbol{u}} \sim \psi_{ev}+\psi_{cv}+\psi_{ac}\cdot\psi_{ce}+\psi_{ce}\cdot\psi_{cv}$	0.160974	0.32444	74832.4
817	$\overline{\boldsymbol{u}} \sim \psi_{ev}+\psi_{cv}+\psi_{ac}\cdot\psi_{ce}+\psi_{ce}\cdot\psi_{cv}+\psi_{ev}\cdot\psi_{cv}$	0.161	0.32443	74833.9
818	$\overline{\boldsymbol{u}} \sim \psi_{ce}+\psi_{ev}+\psi_{cv}+\psi_{ce}\cdot\psi_{ev}+\psi_{ce}\cdot\psi_{cv}$	0.205756	0.32203	74941.2
819	$\overline{\boldsymbol{u}} \sim \psi_{ce}+\psi_{ev}+\psi_{cv}+\psi_{ce}\cdot\psi_{ev}+\psi_{ce}\cdot\psi_{cv}+\psi_{ev}\cdot\psi_{cv}$	0.205845	0.32203	74942.3
820	$\overline{\boldsymbol{u}} \sim \psi_{ac}\cdot\psi_{ev}+\psi_{ce}\cdot\psi_{cv}+\psi_{ev}\cdot\psi_{cv}$	0.0902928	0.32178	74950.5
821	$\overline{\boldsymbol{u}} \sim \psi_{ce}+\psi_{ev}+\psi_{ac}\cdot\psi_{ce}+\psi_{ce}\cdot\psi_{ev}+\psi_{ce}\cdot\psi_{cv}+\psi_{ev}\cdot\psi_{cv}$	0.160196	0.32172	74956.4
822	$\overline{\boldsymbol{u}} \sim \psi_{ce}+\psi_{ev}+\psi_{cv}+\psi_{ce}\cdot\psi_{cv}$	0.202322	0.32157	74960.9
823	$\overline{\boldsymbol{u}} \sim \psi_{ce}+\psi_{ev}+\psi_{cv}+\psi_{ce}\cdot\psi_{cv}+\psi_{ev}\cdot\psi_{cv}$	0.202394	0.32156	74962.3
824	$\overline{\boldsymbol{u}} \sim \psi_{ce}+\psi_{ev}+\psi_{cv}+\psi_{ac}\cdot\psi_{ce}+\psi_{ce}\cdot\psi_{ev}$	0.155254	0.3215	74964.8
825	$\overline{\boldsymbol{u}} \sim \psi_{ce}+\psi_{ev}+\psi_{cv}+\psi_{ac}\cdot\psi_{ce}+\psi_{ce}\cdot\psi_{ev}+\psi_{ev}\cdot\psi_{cv}$	0.155277	0.3215	74966.1
826	$\overline{\boldsymbol{u}} \sim \psi_{ce}+\psi_{ac}\cdot\psi_{ev}+\psi_{ce}\cdot\psi_{ev}$	0.0960645	0.32133	74970.8
827	$\overline{\boldsymbol{u}} \sim \psi_{ce}+\psi_{ev}+\psi_{ac}\cdot\psi_{ce}+\psi_{ce}\cdot\psi_{cv}+\psi_{ev}\cdot\psi_{cv}$	0.158881	0.3212	74978.3
828	$\overline{\boldsymbol{u}} \sim \psi_{ce}+\psi_{ev}+\psi_{cv}+\psi_{ac}\cdot\psi_{ce}$	0.154103	0.32107	74983.3
829	$\overline{\boldsymbol{u}} \sim \psi_{ce}+\psi_{ev}+\psi_{cv}+\psi_{ac}\cdot\psi_{ce}+\psi_{ev}\cdot\psi_{cv}$	0.154121	0.32105	74984.9
830	$\overline{\boldsymbol{u}} \sim \psi_{ce}+\psi_{ev}+\psi_{ac}\cdot\psi_{ce}+\psi_{ce}\cdot\psi_{ev}+\psi_{ev}\cdot\psi_{cv}$	0.155982	0.32073	74999.3
831	$\overline{\boldsymbol{u}} \sim \psi_{ce}+\psi_{ev}+\psi_{ac}\cdot\psi_{ce}+\psi_{ev}\cdot\psi_{cv}$	0.154821	0.32024	75019.9
832	$\overline{\boldsymbol{u}} \sim \psi_{ev}+\psi_{ac}\cdot\psi_{ce}+\psi_{ce}\cdot\psi_{ev}+\psi_{ce}\cdot\psi_{cv}+\psi_{ev}\cdot\psi_{cv}$	0.156435	0.31965	75047.4
833	$\overline{\boldsymbol{u}} \sim \psi_{ev}+\psi_{ac}\cdot\psi_{ce}+\psi_{ce}\cdot\psi_{cv}+\psi_{ev}\cdot\psi_{cv}$	0.156685	0.31962	75047.7

834	$\overline{\boldsymbol{u}} \sim \psi_{ev}+\psi_{cv}+\psi_{ac}\cdot\psi_{ce}+\psi_{ce}\cdot\psi_{ev}$	0.15164	0.31901	75074.8
835	$\overline{\boldsymbol{u}} \sim \psi_{ev}+\psi_{cv}+\psi_{ac}\cdot\psi_{ce}+\psi_{ce}\cdot\psi_{ev}+\psi_{ev}\cdot\psi_{cv}$	0.151657	0.319	75076.4
836	$\overline{\boldsymbol{u}} \sim \psi_{ev}+\psi_{cv}+\psi_{ac}\cdot\psi_{ce}$	0.15227	0.31881	75082.6
837	$\overline{\boldsymbol{u}} \sim \psi_{ev}+\psi_{cv}+\psi_{ac}\cdot\psi_{ce}+\psi_{ev}\cdot\psi_{cv}$	0.152284	0.3188	75084.2
838	$\overline{\boldsymbol{u}} \sim \psi_{ce}+\psi_{ev}+\psi_{ce}\cdot\psi_{ev}+\psi_{ce}\cdot\psi_{cv}+\psi_{ev}\cdot\psi_{cv}$	0.202317	0.3184	75102.8
839	$\overline{\boldsymbol{u}} \sim \psi_{ev}+\psi_{cv}+\psi_{ce}\cdot\psi_{ev}+\psi_{ce}\cdot\psi_{cv}$	0.185136	0.31828	75107.1
840	$\overline{\boldsymbol{u}} \sim \psi_{ev}+\psi_{cv}+\psi_{ce}\cdot\psi_{ev}+\psi_{ce}\cdot\psi_{cv}+\psi_{ev}\cdot\psi_{cv}$	0.185184	0.31827	75108.7
841	$\overline{\boldsymbol{u}} \sim \psi_{ce}+\psi_{ev}+\psi_{ce}\cdot\psi_{cv}+\psi_{ev}\cdot\psi_{cv}$	0.198984	0.31786	75125.9
842	$\overline{\boldsymbol{u}} \sim \psi_{ev}+\psi_{ac}\cdot\psi_{ce}+\psi_{ce}\cdot\psi_{ev}+\psi_{ev}\cdot\psi_{cv}$	0.151962	0.3177	75132.9
843	$\overline{\boldsymbol{u}} \sim \psi_{ev}+\psi_{cv}+\psi_{ce}\cdot\psi_{cv}$	0.183674	0.31749	75141.4
844	$\overline{\boldsymbol{u}} \sim \psi_{ev}+\psi_{cv}+\psi_{ce}\cdot\psi_{cv}+\psi_{ev}\cdot\psi_{cv}$	0.183724	0.31748	75142.7
845	$\overline{\boldsymbol{u}} \sim \psi_{ev}+\psi_{ac}\cdot\psi_{ce}+\psi_{ev}\cdot\psi_{cv}$	0.15275	0.3174	75145.4
846	$\overline{\boldsymbol{u}} \sim \psi_{ce}+\psi_{ev}+\psi_{ac}\cdot\psi_{ce}+\psi_{ce}\cdot\psi_{ev}+\psi_{ce}\cdot\psi_{cv}$	0.155573	0.31689	75169.7
847	$\overline{\boldsymbol{u}} \sim \psi_{ev}+\psi_{ac}\cdot\psi_{ce}+\psi_{ac}\cdot\psi_{cv}+\psi_{ce}\cdot\psi_{ev}$	0.139835	0.31656	75183.3
848	$\overline{\boldsymbol{u}} \sim \psi_{ce}+\psi_{ev}+\psi_{ac}\cdot\psi_{ce}+\psi_{ce}\cdot\psi_{cv}$	0.154343	0.31646	75188.1
849	$\overline{\boldsymbol{u}} \sim \psi_{ce}+\psi_{ev}+\psi_{cv}+\psi_{ce}\cdot\psi_{ev}$	0.2046	0.31542	75233.8
850	$\overline{\boldsymbol{u}} \sim \psi_{ce}+\psi_{ev}+\psi_{cv}+\psi_{ce}\cdot\psi_{ev}+\psi_{ev}\cdot\psi_{cv}$	0.204674	0.31542	75235.1
851	$\overline{\boldsymbol{u}} \sim \psi_{ce}+\psi_{ev}+\psi_{ac}\cdot\psi_{ce}+\psi_{ce}\cdot\psi_{ev}$	0.164428	0.31528	75240.1
852	$\overline{\boldsymbol{u}} \sim \psi_{ce}+\psi_{ev}+\psi_{ce}\cdot\psi_{ev}+\psi_{ev}\cdot\psi_{cv}$	0.20311	0.31523	75242.2
853	$\overline{\boldsymbol{u}} \sim \psi_{ce}+\psi_{ev}+\psi_{cv}$	0.201079	0.31495	75253.5
854	$\overline{\boldsymbol{u}} \sim \psi_{ce}+\psi_{ev}+\psi_{cv}+\psi_{ev}\cdot\psi_{cv}$	0.201138	0.31494	75255.1
855	$\overline{\boldsymbol{u}} \sim \psi_{ce}+\psi_{ev}+\psi_{ac}\cdot\psi_{ce}$	0.162858	0.31484	75258.6
856	$\overline{\boldsymbol{u}} \sim \psi_{ce}+\psi_{ev}+\psi_{ev}\cdot\psi_{cv}$	0.199607	0.31474	75263.1
857	$\overline{\boldsymbol{u}} \sim \psi_{ac}+\psi_{ce}+\psi_{ac}\cdot\psi_{cv}$	0.0614812	0.31463	75267.7
858	$\overline{\boldsymbol{u}} \sim \psi_{ce}+\psi_{ev}+\psi_{ce}\cdot\psi_{ev}+\psi_{ce}\cdot\psi_{cv}$	0.198633	0.31311	75335.8
859	$\overline{\boldsymbol{u}} \sim \psi_{ce}+\psi_{ev}+\psi_{ce}\cdot\psi_{ev}$	0.199621	0.31307	75336.7
860	$\overline{\boldsymbol{u}} \sim \psi_{ce}+\psi_{ev}+\psi_{ce}\cdot\psi_{cv}$	0.195301	0.31265	75355.3

861	$\overline{\boldsymbol{u}} \sim \psi_{ce}+\psi_{ev}$	0.196193	0.3126	75356.2
862	$\overline{\boldsymbol{u}} \sim \psi_{ce}+\psi_{ac}\cdot\psi_{ce}+\psi_{ce}\cdot\psi_{ev}+\psi_{ce}\cdot\psi_{cv}+\psi_{ev}\cdot\psi_{cv}$	0.151137	0.31245	75366.1
863	$\overline{\boldsymbol{u}} \sim \psi_{ce}+\psi_{cv}+\psi_{ac}\cdot\psi_{ce}+\psi_{ce}\cdot\psi_{ev}+\psi_{ce}\cdot\psi_{cv}+\psi_{ev}\cdot\psi_{cv}$	0.151157	0.31244	75367.2
864	$\overline{\boldsymbol{u}} \sim \psi_{ev}+\psi_{ac}\cdot\psi_{ce}+\psi_{ce}\cdot\psi_{ev}+\psi_{ce}\cdot\psi_{cv}$	0.150594	0.311	75428.6
865	$\overline{\boldsymbol{u}} \sim \psi_{ev}+\psi_{ac}\cdot\psi_{ce}+\psi_{ac}\cdot\psi_{cv}$	0.15667	0.31084	75434.6
866	$\overline{\boldsymbol{u}} \sim \psi_{ev}+\psi_{ce}\cdot\psi_{ev}+\psi_{ce}\cdot\psi_{cv}+\psi_{ev}\cdot\psi_{cv}$	0.176085	0.3093	75503.4
867	$\overline{\boldsymbol{u}} \sim \psi_{ev}+\psi_{ac}\cdot\psi_{ce}+\psi_{ce}\cdot\psi_{cv}$	0.153874	0.30914	75509
868	$\overline{\boldsymbol{u}} \sim \psi_{ce}+\psi_{ce}\cdot\psi_{ev}+\psi_{ce}\cdot\psi_{cv}+\psi_{ev}\cdot\psi_{cv}$	0.164712	0.30914	75510.4
869	$\overline{\boldsymbol{u}} \sim \psi_{ce}+\psi_{cv}+\psi_{ce}\cdot\psi_{ev}+\psi_{ce}\cdot\psi_{cv}+\psi_{ev}\cdot\psi_{cv}$	0.164859	0.30912	75512.3
870	$\overline{\boldsymbol{u}} \sim \psi_{ce}+\psi_{cv}+\psi_{ac}\cdot\psi_{ce}+\psi_{ce}\cdot\psi_{ev}+\psi_{ev}\cdot\psi_{cv}$	0.145976	0.30897	75518.9
871	$\overline{\boldsymbol{u}} \sim \psi_{ev}+\psi_{ac}\cdot\psi_{cv}+\psi_{ce}\cdot\psi_{ev}$	0.113615	0.30885	75522.1
872	$\overline{\boldsymbol{u}} \sim \psi_{ev}+\psi_{ac}\cdot\psi_{ce}+\psi_{ce}\cdot\psi_{ev}$	0.155888	0.30873	75527
873	$\overline{\boldsymbol{u}} \sim \psi_{cv}+\psi_{ac}\cdot\psi_{ce}+\psi_{ce}\cdot\psi_{ev}+\psi_{ce}\cdot\psi_{cv}+\psi_{ev}\cdot\psi_{cv}$	0.150006	0.30751	75582.7
874	$\overline{\boldsymbol{u}} \sim \psi_{ac}\cdot\psi_{ce}+\psi_{ce}\cdot\psi_{ev}+\psi_{ce}\cdot\psi_{cv}+\psi_{ev}\cdot\psi_{cv}$	0.149992	0.30746	75583.6
875	$\overline{\boldsymbol{u}} \sim \psi_{ev}+\psi_{ac}\cdot\psi_{ce}$	0.159835	0.30651	75623.2
876	$\overline{\boldsymbol{u}} \sim \psi_{ce}+\psi_{ac}\cdot\psi_{ce}+\psi_{ce}\cdot\psi_{ev}+\psi_{ev}\cdot\psi_{cv}$	0.1421	0.30487	75696.7
877	$\overline{\boldsymbol{u}} \sim \psi_{ev}+\psi_{ce}\cdot\psi_{cv}+\psi_{ev}\cdot\psi_{cv}$	0.175149	0.30393	75736.6
878	$\overline{\boldsymbol{u}} \sim \psi_{ce}+\psi_{ac}\cdot\psi_{ce}+\psi_{ac}\cdot\psi_{cv}+\psi_{ce}\cdot\psi_{ev}$	0.0964946	0.30354	75754.4
879	$\overline{\boldsymbol{u}} \sim \psi_{ac}\cdot\psi_{ce}+\psi_{ac}\cdot\psi_{ev}+\psi_{ce}\cdot\psi_{ev}+\psi_{ev}\cdot\psi_{cv}$	0.128804	0.30278	75787.8
880	$\overline{\boldsymbol{u}} \sim \psi_{ce}+\psi_{ac}\cdot\psi_{cv}+\psi_{ce}\cdot\psi_{ev}$	0.110398	0.30252	75798
881	$\overline{\boldsymbol{u}} \sim \psi_{ce}+\psi_{cv}+\psi_{ce}\cdot\psi_{ev}+\psi_{ev}\cdot\psi_{cv}$	0.163687	0.30248	75800.6
882	$\overline{\boldsymbol{u}} \sim \psi_{ce}+\psi_{ac}\cdot\psi_{ce}+\psi_{ac}\cdot\psi_{ev}+\psi_{ac}\cdot\psi_{cv}+\psi_{ce}\cdot\psi_{cv}$	0.0823681	0.30228	75810.3
883	$\overline{\boldsymbol{u}} \sim \psi_{ac}+\psi_{ce}$	0.0626002	0.30132	75849
884	$\overline{\boldsymbol{u}} \sim \psi_{ac}+\psi_{ac}\cdot\psi_{ce}+\psi_{ac}\cdot\psi_{cv}+\psi_{ce}\cdot\psi_{ev}+\psi_{ce}\cdot\psi_{cv}$	0.121449	0.30041	75891.3
885	$\overline{\boldsymbol{u}} \sim \psi_{ce}+\psi_{cv}+\psi_{ac}\cdot\psi_{ce}+\psi_{ce}\cdot\psi_{cv}+\psi_{ev}\cdot\psi_{cv}$	0.141173	0.29841	75977.4
886	$\overline{\boldsymbol{u}} \sim \psi_{cv}+\psi_{ac}\cdot\psi_{ce}+\psi_{ce}\cdot\psi_{cv}+\psi_{ev}\cdot\psi_{cv}$	0.141551	0.29829	75981.9
887	$\overline{\boldsymbol{u}} \sim \psi_{cv}+\psi_{ac}\cdot\psi_{ce}+\psi_{ce}\cdot\psi_{ev}+\psi_{ev}\cdot\psi_{cv}$	0.145373	0.29785	76000.7

888	$\overline{\boldsymbol{u}} \sim \psi_{ce}+\psi_{ac}\cdot\psi_{ev}+\psi_{ac}\cdot\psi_{cv}+\psi_{ce}\cdot\psi_{cv}$	0.0672149	0.29611	76075.8
889	$\overline{\boldsymbol{u}} \sim \psi_{ce}+\psi_{cv}+\psi_{ac}\cdot\psi_{ce}+\psi_{ce}\cdot\psi_{ev}+\psi_{ce}\cdot\psi_{cv}$	0.141623	0.29607	76078.5
890	$\overline{\boldsymbol{u}} \sim \psi_{ac}+\psi_{ac}\cdot\psi_{ce}+\psi_{ac}\cdot\psi_{cv}+\psi_{ce}\cdot\psi_{ev}$	0.135029	0.29567	76094.4
891	$\overline{\boldsymbol{u}} \sim \psi_{ce}+\psi_{cv}+\psi_{ce}\cdot\psi_{cv}+\psi_{ev}\cdot\psi_{cv}$	0.147436	0.29501	76122.9
892	$\overline{\boldsymbol{u}} \sim \psi_{ce}+\psi_{cv}+\psi_{ac}\cdot\psi_{ce}+\psi_{ev}\cdot\psi_{cv}$	0.137287	0.29493	76126.3
893	$\overline{\boldsymbol{u}} \sim \psi_{ce}+\psi_{ce}\cdot\psi_{ev}+\psi_{ev}\cdot\psi_{cv}$	0.156613	0.29489	76127.2
894	$\overline{\boldsymbol{u}} \sim \psi_{cv}+\psi_{ce}\cdot\psi_{ev}+\psi_{ce}\cdot\psi_{cv}+\psi_{ev}\cdot\psi_{cv}$	0.151657	0.29441	76148.7
895	$\overline{\boldsymbol{u}} \sim \psi_{ce}\cdot\psi_{ev}+\psi_{ce}\cdot\psi_{cv}+\psi_{ev}\cdot\psi_{cv}$	0.152391	0.29358	76183
896	$\overline{\boldsymbol{u}} \sim \psi_{ce}+\psi_{ac}\cdot\psi_{ce}+\psi_{ce}\cdot\psi_{cv}+\psi_{ev}\cdot\psi_{cv}$	0.13927	0.29336	76193.7
897	$\overline{\boldsymbol{u}} \sim \psi_{ac}+\psi_{ac}\cdot\psi_{cv}+\psi_{ce}\cdot\psi_{ev}+\psi_{ce}\cdot\psi_{cv}$	0.0976745	0.29334	76194.7
898	$\overline{\boldsymbol{u}} \sim \psi_{cv}+\psi_{ac}\cdot\psi_{ev}+\psi_{ce}\cdot\psi_{ev}+\psi_{ev}\cdot\psi_{cv}$	0.0899368	0.29284	76216
899	$\overline{\boldsymbol{u}} \sim \psi_{ce}+\psi_{cv}+\psi_{ac}\cdot\psi_{ce}+\psi_{ce}\cdot\psi_{ev}$	0.138182	0.29271	76221.6
900	$\overline{\boldsymbol{u}} \sim \psi_{cv}+\psi_{ac}\cdot\psi_{ce}+\psi_{ev}\cdot\psi_{cv}$	0.138966	0.29269	76221.2
901	$\overline{\boldsymbol{u}} \sim \psi_{ce}+\psi_{cv}+\psi_{ce}\cdot\psi_{ev}+\psi_{ce}\cdot\psi_{cv}$	0.147005	0.29259	76226.7
902	$\overline{\boldsymbol{u}} \sim \psi_{ac}\cdot\psi_{ce}+\psi_{ce}\cdot\psi_{cv}+\psi_{ev}\cdot\psi_{cv}$	0.137862	0.29223	76241.1
903	$\overline{\boldsymbol{u}} \sim \psi_{ev}+\psi_{cv}+\psi_{ce}\cdot\psi_{ev}$	0.162194	0.29169	76264
904	$\overline{\boldsymbol{u}} \sim \psi_{ev}+\psi_{cv}+\psi_{ce}\cdot\psi_{ev}+\psi_{ev}\cdot\psi_{cv}$	0.162166	0.29167	76266
905	$\overline{\boldsymbol{u}} \sim \psi_{ev}+\psi_{ce}\cdot\psi_{ev}+\psi_{ev}\cdot\psi_{cv}$	0.161888	0.29131	76280.3
906	$\overline{\boldsymbol{u}} \sim \psi_{ev}+\psi_{ce}\cdot\psi_{ev}+\psi_{ce}\cdot\psi_{cv}$	0.165479	0.29129	76281.3
907	$\overline{\boldsymbol{u}} \sim \psi_{cv}+\psi_{ce}\cdot\psi_{cv}+\psi_{ev}\cdot\psi_{cv}$	0.147142	0.29115	76287.1
908	$\overline{\boldsymbol{u}} \sim \psi_{ac}\cdot\psi_{ce}+\psi_{ce}\cdot\psi_{ev}+\psi_{ev}\cdot\psi_{cv}$	0.144224	0.29059	76310.9
909	$\overline{\boldsymbol{u}} \sim \psi_{ce}+\psi_{ce}\cdot\psi_{cv}+\psi_{ev}\cdot\psi_{cv}$	0.141988	0.29043	76317.9
910	$\overline{\boldsymbol{u}} \sim \psi_{ce}\cdot\psi_{cv}+\psi_{ev}\cdot\psi_{cv}$	0.143305	0.29021	76326.2
911	$\overline{\boldsymbol{u}} \sim \psi_{ac}+\psi_{cv}+\psi_{ac}\cdot\psi_{ce}$	0.0826103	0.28972	76348.3
912	$\overline{\boldsymbol{u}} \sim \psi_{ev}+\psi_{ce}\cdot\psi_{ev}$	0.16	0.28918	76369.9
913	$\overline{\boldsymbol{u}} \sim \psi_{ce}+\psi_{cv}+\psi_{ev}\cdot\psi_{cv}$	0.146216	0.28832	76407.6
914	$\overline{\boldsymbol{u}} \sim \psi_{ce}+\psi_{ac}\cdot\psi_{ce}+\psi_{ce}\cdot\psi_{ev}+\psi_{ce}\cdot\psi_{cv}$	0.13748	0.28811	76417.5

915	$\overline{\boldsymbol{u}} \sim \psi_{ce}+\psi_{ac}\cdot\psi_{ce}+\psi_{ce}\cdot\psi_{ev}$	0.139808	0.28638	76490.2
916	$\overline{\boldsymbol{u}} \sim \psi_{ac}+\psi_{ac}\cdot\psi_{ce}+\psi_{ce}\cdot\psi_{ev}+\psi_{ce}\cdot\psi_{cv}$	0.147359	0.28619	76499
917	$\overline{\boldsymbol{u}} \sim \psi_{ce}+\psi_{cv}+\psi_{ce}\cdot\psi_{ev}$	0.146099	0.28605	76504
918	$\overline{\boldsymbol{u}} \sim \psi_{ce}+\psi_{ce}\cdot\psi_{ev}+\psi_{ce}\cdot\psi_{cv}$	0.144494	0.28383	76598.1
919	$\overline{\boldsymbol{u}} \sim \psi_{ce}+\psi_{ce}\cdot\psi_{ev}$	0.144434	0.28378	76599.1
920	$\overline{\boldsymbol{u}} \sim \psi_{ac}+\psi_{ac}\cdot\psi_{ce}+\psi_{ce}\cdot\psi_{ev}$	0.149931	0.28026	76748.5
921	$\overline{\boldsymbol{u}} \sim \psi_{cv}+\psi_{ac}\cdot\psi_{ce}+\psi_{ac}\cdot\psi_{ev}+\psi_{ce}\cdot\psi_{ev}+\psi_{ce}\cdot\psi_{cv}$	0.139457	0.2794	76786.6
922	$\overline{\boldsymbol{u}} \sim \psi_{cv}+\psi_{ac}\cdot\psi_{ce}+\psi_{ce}\cdot\psi_{ev}+\psi_{ce}\cdot\psi_{cv}$	0.145791	0.27771	76856.4
923	$\overline{\boldsymbol{u}} \sim \psi_{ac}\cdot\psi_{ce}+\psi_{ac}\cdot\psi_{ev}+\psi_{ac}\cdot\psi_{cv}+\psi_{ce}\cdot\psi_{ev}+\psi_{ce}\cdot\psi_{cv}$	0.145075	0.26602	77343.3
924	$\overline{\boldsymbol{u}} \sim \psi_{ac}+\psi_{cv}+\psi_{ce}\cdot\psi_{ev}$	0.0596504	0.26165	77520.9
925	$\overline{\boldsymbol{u}} \sim \psi_{ac}+\psi_{cv}$	0.0585538	0.26086	77552.4
926	$\overline{\boldsymbol{u}} \sim \psi_{ac}\cdot\psi_{ce}+\psi_{ac}\cdot\psi_{ev}+\psi_{ac}\cdot\psi_{cv}+\psi_{ce}\cdot\psi_{cv}$	0.134746	0.25985	77595.5
927	$\overline{\boldsymbol{u}} \sim \psi_{cv}+\psi_{ac}\cdot\psi_{ce}+\psi_{ac}\cdot\psi_{ev}+\psi_{ce}\cdot\psi_{ev}$	0.147939	0.25921	77621.9
928	$\overline{\boldsymbol{u}} \sim \psi_{cv}+\psi_{ac}\cdot\psi_{ce}+\psi_{ce}\cdot\psi_{ev}$	0.146203	0.25891	77632.8
929	$\overline{\boldsymbol{u}} \sim \psi_{ce}+\psi_{ac}\cdot\psi_{ce}+\psi_{ev}\cdot\psi_{cv}$	0.121985	0.25482	77799.4
930	$\overline{\boldsymbol{u}} \sim \psi_{ce}+\psi_{ac}\cdot\psi_{ce}+\psi_{ac}\cdot\psi_{ev}+\psi_{ev}\cdot\psi_{cv}$	0.120707	0.25481	77800.8
931	$\overline{\boldsymbol{u}} \sim \psi_{ac}\cdot\psi_{ce}+\psi_{ac}\cdot\psi_{ev}+\psi_{ev}\cdot\psi_{cv}$	0.117381	0.25455	77810.3
932	$\overline{\boldsymbol{u}} \sim \psi_{ac}\cdot\psi_{ce}+\psi_{ev}\cdot\psi_{cv}$	0.119968	0.25451	77811
933	$\overline{\boldsymbol{u}} \sim \psi_{cv}+\psi_{ac}\cdot\psi_{ev}+\psi_{ev}\cdot\psi_{cv}$	0.0828013	0.25278	77882.3
934	$\overline{\boldsymbol{u}} \sim \psi_{ac}+\psi_{ac}\cdot\psi_{ce}+\psi_{ac}\cdot\psi_{cv}+\psi_{ce}\cdot\psi_{cv}$	0.0670147	0.25226	77904.4
935	$\overline{\boldsymbol{u}} \sim \psi_{ac}+\psi_{ac}\cdot\psi_{cv}+\psi_{ce}\cdot\psi_{cv}$	0.0702977	0.25204	77912.4
936	$\overline{\boldsymbol{u}} \sim \psi_{cv}+\psi_{ac}\cdot\psi_{ev}+\psi_{ce}\cdot\psi_{ev}+\psi_{ce}\cdot\psi_{cv}$	0.120359	0.25156	77932.7
937	$\overline{\boldsymbol{u}} \sim \psi_{cv}+\psi_{ce}\cdot\psi_{ev}+\psi_{ce}\cdot\psi_{cv}$	0.134452	0.24797	78076.3
938	$\overline{\boldsymbol{u}} \sim \psi_{ev}+\psi_{ac}\cdot\psi_{cv}+\psi_{ce}\cdot\psi_{cv}$	0.438545	0.24771	78086.9
939	$\overline{\boldsymbol{u}} \sim \psi_{ev}+\psi_{ce}\cdot\psi_{cv}$	0.487845	0.24704	78112.7
940	$\overline{\boldsymbol{u}} \sim \psi_{ac}+\psi_{ac}\cdot\psi_{ce}+\psi_{ac}\cdot\psi_{cv}$	0.084898	0.24478	78204.4
941	$\overline{\boldsymbol{u}} \sim \psi_{ac}\cdot\psi_{ce}+\psi_{ac}\cdot\psi_{cv}+\psi_{ce}\cdot\psi_{ev}+\psi_{ce}\cdot\psi_{cv}$	0.149872	0.24294	78279.2

942	$\overline{\boldsymbol{u}} \sim \psi_{ac}{\cdot}\psi_{ce}{+}\psi_{ac}{\cdot}\psi_{ev}{+}\psi_{ce}{\cdot}\psi_{ev}{+}\psi_{ce}{\cdot}\psi_{cv}$	0.156561	0.23955	78414.5
943	$\overline{\boldsymbol{u}} \sim \psi_{ce}{+}\psi_{ac}{\cdot}\psi_{ce}{+}\psi_{ac}{\cdot}\psi_{cv}{+}\psi_{ce}{\cdot}\psi_{cv}$	0.0402935	0.23794	78478.4
944	$\overline{\boldsymbol{u}} \sim \psi_{ce}{+}\psi_{ac}{\cdot}\psi_{cv}{+}\psi_{ce}{\cdot}\psi_{cv}$	0.0437858	0.23741	78498.1
945	$\overline{\boldsymbol{u}} \sim \psi_{ac}{\cdot}\psi_{ce}{+}\psi_{ac}{\cdot}\psi_{ev}{+}\psi_{ac}{\cdot}\psi_{cv}{+}\psi_{ce}{\cdot}\psi_{ev}$	0.16343	0.23696	78517.4
946	$\overline{\boldsymbol{u}} \sim \psi_{ac}{\cdot}\psi_{ce}{+}\psi_{ac}{\cdot}\psi_{ev}{+}\psi_{ce}{\cdot}\psi_{ev}$	0.161821	0.23565	78568
947	$\overline{\boldsymbol{u}} \sim \psi_{ac}{\cdot}\psi_{ce}{+}\psi_{ce}{\cdot}\psi_{ev}{+}\psi_{ce}{\cdot}\psi_{cv}$	0.151572	0.23503	78592.8
948	$\overline{\boldsymbol{u}} \sim \psi_{ac}{+}\psi_{ac}{\cdot}\psi_{ce}{+}\psi_{ce}{\cdot}\psi_{cv}$	0.0974183	0.23449	78614.1
949	$\overline{\boldsymbol{u}} \sim \psi_{ce}{+}\psi_{ac}{\cdot}\psi_{ev}{+}\psi_{ev}{\cdot}\psi_{cv}$	0.0839301	0.23169	78724.6
950	$\overline{\boldsymbol{u}} \sim \psi_{ac}{\cdot}\psi_{ce}{+}\psi_{ce}{\cdot}\psi_{ev}$	0.155577	0.23048	78771.1
951	$\overline{\boldsymbol{u}} \sim \psi_{ac}{\cdot}\psi_{ce}{+}\psi_{ac}{\cdot}\psi_{cv}{+}\psi_{ce}{\cdot}\psi_{ev}$	0.155621	0.23046	78772.9
952	$\overline{\boldsymbol{u}} \sim \psi_{ac}{+}\psi_{ac}{\cdot}\psi_{ce}$	0.0924464	0.2298	78798
953	$\overline{\boldsymbol{u}} \sim \psi_{cv}{+}\psi_{ce}{\cdot}\psi_{ev}{+}\psi_{ev}{\cdot}\psi_{cv}$	0.138571	0.22939	78815
954	$\overline{\boldsymbol{u}} \sim \psi_{ce}{+}\psi_{ev}{\cdot}\psi_{cv}$	0.124923	0.22937	78814.7
955	$\overline{\boldsymbol{u}} \sim \psi_{ac}{+}\psi_{ac}{\cdot}\psi_{cv}{+}\psi_{ce}{\cdot}\psi_{ev}$	0.0595615	0.21304	79450.3
956	$\overline{\boldsymbol{u}} \sim \psi_{ac}{+}\psi_{ac}{\cdot}\psi_{cv}$	0.0588044	0.21289	79454.9
957	$\overline{\boldsymbol{u}} \sim \psi_{ac}{+}\psi_{ce}{\cdot}\psi_{ev}{+}\psi_{ce}{\cdot}\psi_{cv}$	0.0758776	0.2124	79474.7
958	$\overline{\boldsymbol{u}} \sim \psi_{ce}{+}\psi_{ac}{\cdot}\psi_{ce}{+}\psi_{ac}{\cdot}\psi_{ev}{+}\psi_{ac}{\cdot}\psi_{cv}$	0.157673	0.20966	79580.9
959	$\overline{\boldsymbol{u}} \sim \psi_{ce}{+}\psi_{ac}{\cdot}\psi_{ev}{+}\psi_{ac}{\cdot}\psi_{cv}$	0.126781	0.20712	79676.8
960	$\overline{\boldsymbol{u}} \sim \psi_{ac}{+}\psi_{ce}{\cdot}\psi_{cv}$	0.0645558	0.20671	79691.5
961	$\overline{\boldsymbol{u}} \sim \psi_{ac}{\cdot}\psi_{ce}{+}\psi_{ac}{\cdot}\psi_{ev}{+}\psi_{ac}{\cdot}\psi_{cv}$	0.170682	0.20545	79740.6
962	$\overline{\boldsymbol{u}} \sim \psi_{ce}{+}\psi_{cv}{+}\psi_{ac}{\cdot}\psi_{ev}{+}\psi_{ce}{\cdot}\psi_{cv}$	0.819412	0.20202	79872
963	$\overline{\boldsymbol{u}} \sim \psi_{ev}{+}\psi_{ac}{\cdot}\psi_{cv}$	0.258339	0.20183	79877.2
964	$\overline{\boldsymbol{u}} \sim \psi_{ce}{+}\psi_{ac}{\cdot}\psi_{ev}{+}\psi_{ce}{\cdot}\psi_{cv}$	0.826212	0.20035	79934.3
965	$\overline{\boldsymbol{u}} \sim \psi_{ce}{+}\psi_{cv}{+}\psi_{ac}{\cdot}\psi_{ce}{+}\psi_{ac}{\cdot}\psi_{ev}{+}\psi_{ce}{\cdot}\psi_{cv}$	0.177807	0.19982	79956.3
966	$\overline{\boldsymbol{u}} \sim \psi_{ac}{+}\psi_{ce}{\cdot}\psi_{ev}$	0.0599743	0.19824	80012.8
967	$\overline{\boldsymbol{u}} \sim \psi_{ac}$	0.059216	0.19814	80015.8
968	$\overline{\boldsymbol{u}} \sim \psi_{cv}{+}\psi_{ac}{\cdot}\psi_{ce}{+}\psi_{ac}{\cdot}\psi_{ev}{+}\psi_{ce}{\cdot}\psi_{cv}$	0.184086	0.19733	80049.4

969	$\overline{\boldsymbol{u}} \sim \psi_{ce}+\psi_{cv}+\psi_{ac}\cdot\psi_{ce}+\psi_{ac}\cdot\psi_{ev}$	0.171442	0.19716	80055.7
970	$\overline{\boldsymbol{u}} \sim \psi_{cv}+\psi_{ac}\cdot\psi_{ce}+\psi_{ac}\cdot\psi_{ev}$	0.174944	0.19627	80088.2
971	$\overline{\boldsymbol{u}} \sim \psi_{ce}+\psi_{cv}+\psi_{ac}\cdot\psi_{ev}$	0.825277	0.19405	80171.7
972	$\overline{\boldsymbol{u}} \sim \psi_{ce}+\psi_{ac}\cdot\psi_{ev}$	0.820234	0.19322	80201.9
973	$\overline{\boldsymbol{u}} \sim \psi_{ac}\cdot\psi_{ce}+\psi_{ac}\cdot\psi_{ev}+\psi_{ce}\cdot\psi_{cv}$	0.1726	0.19285	80216.7
974	$\overline{\boldsymbol{u}} \sim \psi_{ce}+\psi_{ac}\cdot\psi_{ce}+\psi_{ac}\cdot\psi_{ev}+\psi_{ce}\cdot\psi_{cv}$	0.172397	0.19283	80218.4
975	$\overline{\boldsymbol{u}} \sim \psi_{ac}\cdot\psi_{ce}+\psi_{ac}\cdot\psi_{ev}$	0.181795	0.19095	80286.9
976	$\overline{\boldsymbol{u}} \sim \psi_{ce}+\psi_{ac}\cdot\psi_{ce}+\psi_{ac}\cdot\psi_{ev}$	0.182188	0.19093	80288.5
977	$\overline{\boldsymbol{u}} \sim \psi_{ev}+\psi_{cv}$	1.83677	0.19069	80296.5
978	$\overline{\boldsymbol{u}} \sim \psi_{ev}+\psi_{cv}+\psi_{ev}\cdot\psi_{cv}$	1.83943	0.19068	80298.1
979	$\overline{\boldsymbol{u}} \sim \psi_{ev}+\psi_{ev}\cdot\psi_{cv}$	0.579492	0.18857	80375.6
980	$\overline{\boldsymbol{u}} \sim \psi_{ev}$	1.91918	0.18706	80431.2
981	$\overline{\boldsymbol{u}} \sim \psi_{cv}+\psi_{ac}\cdot\psi_{ev}+\psi_{ce}\cdot\psi_{cv}$	0.337459	0.18149	80639.7
982	$\overline{\boldsymbol{u}} \sim \psi_{ce}+\psi_{ac}\cdot\psi_{ce}+\psi_{ac}\cdot\psi_{cv}$	0.00542261	0.17224	80979.7
983	$\overline{\boldsymbol{u}} \sim \psi_{ce}+\psi_{ac}\cdot\psi_{cv}$	0.0267583	0.15956	81438.8
984	$\overline{\boldsymbol{u}} \sim \psi_{ac}\cdot\psi_{ce}+\psi_{ac}\cdot\psi_{cv}+\psi_{ce}\cdot\psi_{cv}$	0.104656	0.15916	81454
985	$\overline{\boldsymbol{u}} \sim \psi_{cv}+\psi_{ev}\cdot\psi_{cv}$	0.206022	0.15723	81522.3
986	$\overline{\boldsymbol{u}} \sim \psi_{ce}+\psi_{cv}+\psi_{ac}\cdot\psi_{ce}+\psi_{ce}\cdot\psi_{cv}$	0.0769911	0.14629	81914.6
987	$\overline{\boldsymbol{u}} \sim \psi_{cv}+\psi_{ac}\cdot\psi_{ce}+\psi_{ce}\cdot\psi_{cv}$	0.0851568	0.14593	81926.6
988	$\overline{\boldsymbol{u}} \sim \psi_{ce}+\psi_{cv}+\psi_{ce}\cdot\psi_{cv}$	0.0510963	0.14394	81996.9
989	$\overline{\boldsymbol{u}} \sim \psi_{ce}+\psi_{cv}+\psi_{ac}\cdot\psi_{ce}$	0.0785648	0.14305	82028.2
990	$\overline{\boldsymbol{u}} \sim \psi_{cv}+\psi_{ac}\cdot\psi_{ce}$	0.0969567	0.14023	82126.6
991	$\overline{\boldsymbol{u}} \sim \psi_{cv}+\psi_{ce}\cdot\psi_{cv}$	0.0655384	0.13928	82160
992	$\overline{\boldsymbol{u}} \sim \psi_{ce}+\psi_{ac}\cdot\psi_{ce}+\psi_{ce}\cdot\psi_{cv}$	0.0731028	0.1387	82181.5
993	$\overline{\boldsymbol{u}} \sim \psi_{ce}+\psi_{cv}$	0.0480642	0.13765	82217.3
994	$\overline{\boldsymbol{u}} \sim \psi_{ce}+\psi_{ac}\cdot\psi_{ce}$	0.0629765	0.13729	82230.1
995	$\overline{\boldsymbol{u}} \sim \psi_{ac}\cdot\psi_{ev}+\psi_{ac}\cdot\psi_{cv}+\psi_{ce}\cdot\psi_{ev}+\psi_{ce}\cdot\psi_{cv}$	0.670704	0.13608	82274.5

996	$\overline{\boldsymbol{u}} \sim \psi_{ce}$	0.0457927	0.13566	82286.3
997	$\overline{\boldsymbol{u}} \sim \psi_{ce}+\psi_{ce}\cdot\psi_{cv}$	0.0461795	0.13565	82287.3
998	$\overline{\boldsymbol{u}} \sim \psi_{ac}\cdot\psi_{ev}+\psi_{ce}\cdot\psi_{ev}+\psi_{ce}\cdot\psi_{cv}$	0.659871	0.13549	82294
999	$\overline{\boldsymbol{u}} \sim \psi_{ac}\cdot\psi_{ev}+\psi_{ac}\cdot\psi_{cv}+\psi_{ce}\cdot\psi_{cv}$	0.642889	0.13452	82328.1
1000	$\overline{\boldsymbol{u}} \sim \psi_{ac}\cdot\psi_{ev}+\psi_{ce}\cdot\psi_{cv}$	0.636502	0.13439	82331.5
1001	$\overline{\boldsymbol{u}} \sim \psi_{ac}\cdot\psi_{ce}+\psi_{ac}\cdot\psi_{cv}$	0.0973709	0.13208	82412.4
1002	$\overline{\boldsymbol{u}} \sim \psi_{ac}\cdot\psi_{ce}+\psi_{ce}\cdot\psi_{cv}$	0.105103	0.13031	82473.7
1003	$\overline{\boldsymbol{u}} \sim \psi_{ac}\cdot\psi_{ce}$	0.104131	0.12751	82570.1
1004	$\overline{\boldsymbol{u}} \sim \psi_{ce}\cdot\psi_{ev}+\psi_{ev}\cdot\psi_{cv}$	0.155886	0.12243	82746.8
1005	$\overline{\boldsymbol{u}} \sim \psi_{ac}\cdot\psi_{ev}+\psi_{ce}\cdot\psi_{ev}+\psi_{ev}\cdot\psi_{cv}$	0.162414	0.12241	82748.5
1006	$\overline{\boldsymbol{u}} \sim \psi_{ac}\cdot\psi_{ev}+\psi_{ev}\cdot\psi_{cv}$	0.0604901	0.10765	83252.1
1007	$\overline{\boldsymbol{u}} \sim \psi_{ac}\cdot\psi_{ev}+\psi_{ac}\cdot\psi_{cv}+\psi_{ce}\cdot\psi_{ev}$	0.396298	0.10571	83318.8
1008	$\overline{\boldsymbol{u}} \sim \psi_{ev}\cdot\psi_{cv}$	0.228486	0.10294	83410.3
1009	$\overline{\boldsymbol{u}} \sim \psi_{ac}\cdot\psi_{cv}+\psi_{ce}\cdot\psi_{ev}+\psi_{ce}\cdot\psi_{cv}$	0.234124	0.09575	83654.2
1010	$\overline{\boldsymbol{u}} \sim \psi_{cv}+\psi_{ac}\cdot\psi_{ev}+\psi_{ce}\cdot\psi_{ev}$	0.766434	0.08422	84037.6
1011	$\overline{\boldsymbol{u}} \sim \psi_{ce}\cdot\psi_{ev}+\psi_{ce}\cdot\psi_{cv}$	0.149627	0.08421	84036.6
1012	$\overline{\boldsymbol{u}} \sim \psi_{ac}\cdot\psi_{ev}+\psi_{ce}\cdot\psi_{ev}$	0.750522	0.08354	84059
1013	$\overline{\boldsymbol{u}} \sim \psi_{ac}\cdot\psi_{ev}+\psi_{ac}\cdot\psi_{cv}$	0.587546	0.07964	84187.3
1014	$\overline{\boldsymbol{u}} \sim \psi_{cv}+\psi_{ac}\cdot\psi_{ev}$	0.845929	0.07618	84301
1015	$\overline{\boldsymbol{u}} \sim \psi_{ac}\cdot\psi_{ev}$	0.840011	0.07435	84359.8
1016	$\overline{\boldsymbol{u}} \sim \psi_{ce}\cdot\psi_{cv}$	0.0463237	0.0658	84638
1017	$\overline{\boldsymbol{u}} \sim \psi_{ac}\cdot\psi_{cv}+\psi_{ce}\cdot\psi_{cv}$	0.0430303	0.06579	84639.3
1018	$\overline{\boldsymbol{u}} \sim \psi_{ac}\cdot\psi_{cv}+\psi_{ce}\cdot\psi_{ev}$	0.00548484	0.035	85620.5
1019	$\overline{\boldsymbol{u}} \sim \psi_{ac}\cdot\psi_{cv}$	0.00616805	0.03402	85650.4
1020	$\overline{\boldsymbol{u}} \sim \psi_{cv}+\psi_{ce}\cdot\psi_{ev}$	0.00158602	0.00513	86543
1021	$\overline{\boldsymbol{u}} \sim \psi_{cv}$	0.100029	0.00368	86585.9
1022	$\overline{\boldsymbol{u}} \sim \psi_{ce}\cdot\psi_{ev}$	0.000685411	0.002	86637
