# Supplementary material for: Influence of interaction of cerebral fluids on ventricular deformation: A mathematical approach
Source: PLoS One. 2022 Feb 28;17(2):e0264395. doi: 10.1371/journal.pone.0264395 (PMC8884699; doi:10.1371/journal.pone.0264395)
Supplement: S2 File — The file provides information about linear regression models for volunteer 1, which were constructed when searching for bopt. For each regression model, the regression formula, the value of bopt, Radj.2 and the value of the Akaike information criterion (AIC) are given. (PDF) [file pone.0264395.s003.pdf]

Table 1: Regression results for volunteer 2

| N | Formula | Optimal b | $R^2_{adj.}$ | AIC |
|---|---|---|---|---|
| 0 | $\overline{u} \sim \psi_{ac}+\psi_{ce}+\psi_{ev}+\psi_{cv}+\psi_{ac}\cdot\psi_{ce}+\psi_{ac}\cdot\psi_{ev}+\psi_{ac}\cdot\psi_{cv}+\psi_{ce}\cdot\psi_{cv}+\psi_{ev}\cdot\psi_{cv}$ | 0.188603 | 0.92557 | -16332.9 |
| 1 | $\overline{u} \sim \psi_{ac}+\psi_{ce}+\psi_{ev}+\psi_{cv}+\psi_{ac}\cdot\psi_{ce}+\psi_{ac}\cdot\psi_{ev}+\psi_{ac}\cdot\psi_{cv}+\psi_{ce}\cdot\psi_{ev}+\psi_{ce}\cdot\psi_{cv}+\psi_{ev}\cdot\psi_{cv}$ | 0.188712 | 0.92557 | -16334 |
| 2 | $\overline{u} \sim \psi_{ac}+\psi_{ce}+\psi_{ev}+\psi_{cv}+\psi_{ac}\cdot\psi_{ce}+\psi_{ac}\cdot\psi_{ev}+\psi_{ac}\cdot\psi_{cv}+\psi_{ce}\cdot\psi_{ev}+\psi_{ce}\cdot\psi_{cv}$ | 0.187928 | 0.92542 | -16271.2 |
| 3 | $\overline{u} \sim \psi_{ac}+\psi_{ce}+\psi_{ev}+\psi_{cv}+\psi_{ac}\cdot\psi_{ce}+\psi_{ac}\cdot\psi_{ev}+\psi_{ac}\cdot\psi_{cv}+\psi_{ce}\cdot\psi_{cv}$ | 0.187745 | 0.9254 | -16266.4 |
| 4 | $\overline{u} \sim \psi_{ac}+\psi_{ce}+\psi_{ev}+\psi_{cv}+\psi_{ac}\cdot\psi_{ce}+\psi_{ac}\cdot\psi_{cv}+\psi_{ce}\cdot\psi_{ev}+\psi_{ce}\cdot\psi_{cv}+\psi_{ev}\cdot\psi_{cv}$ | 0.184301 | 0.92484 | -16036.3 |
| 5 | $\overline{u} \sim \psi_{ac}+\psi_{ce}+\psi_{ev}+\psi_{cv}+\psi_{ac}\cdot\psi_{ce}+\psi_{ac}\cdot\psi_{cv}+\psi_{ce}\cdot\psi_{ev}+\psi_{ce}\cdot\psi_{cv}$ | 0.184274 | 0.92483 | -16034.1 |
| 6 | $\overline{u} \sim \psi_{ac}+\psi_{ce}+\psi_{ev}+\psi_{cv}+\psi_{ac}\cdot\psi_{ce}+\psi_{ac}\cdot\psi_{cv}+\psi_{ce}\cdot\psi_{cv}+\psi_{ev}\cdot\psi_{cv}$ | 0.183726 | 0.92477 | -16010.9 |
| 7 | $\overline{u} \sim \psi_{ac}+\psi_{ce}+\psi_{ev}+\psi_{cv}+\psi_{ac}\cdot\psi_{ce}+\psi_{ac}\cdot\psi_{cv}+\psi_{ce}\cdot\psi_{cv}$ | 0.183689 | 0.92476 | -16007.8 |
| 8 | $\overline{u} \sim \psi_{ac}+\psi_{ce}+\psi_{ev}+\psi_{cv}+\psi_{ac}\cdot\psi_{ce}+\psi_{ac}\cdot\psi_{ev}+\psi_{ce}\cdot\psi_{cv}+\psi_{ev}\cdot\psi_{cv}$ | 0.162128 | 0.92234 | -15041.6 |
| 9 | $\overline{u} \sim \psi_{ac}+\psi_{ce}+\psi_{ev}+\psi_{cv}+\psi_{ac}\cdot\psi_{ce}+\psi_{ac}\cdot\psi_{ev}+\psi_{ce}\cdot\psi_{ev}+\psi_{ce}\cdot\psi_{cv}+\psi_{ev}\cdot\psi_{cv}$ | 0.162188 | 0.92234 | -15041.6 |
| 10 | $\overline{u} \sim \psi_{ac}+\psi_{ce}+\psi_{ev}+\psi_{cv}+\psi_{ac}\cdot\psi_{ce}+\psi_{ac}\cdot\psi_{ev}+\psi_{ce}\cdot\psi_{ev}+\psi_{ce}\cdot\psi_{cv}$ | 0.161713 | 0.92221 | -14990.5 |
| 11 | $\overline{u} \sim \psi_{ac}+\psi_{ce}+\psi_{ev}+\psi_{cv}+\psi_{ac}\cdot\psi_{ce}+\psi_{ac}\cdot\psi_{ev}+\psi_{ce}\cdot\psi_{cv}$ | 0.161608 | 0.9222 | -14987.7 |
| 12 | $\overline{u} \sim \psi_{ac}+\psi_{ce}+\psi_{ev}+\psi_{cv}+\psi_{ac}\cdot\psi_{ce}+\psi_{ce}\cdot\psi_{ev}+\psi_{ce}\cdot\psi_{cv}$ | 0.159496 | 0.92172 | -14800 |
| 13 | $\overline{u} \sim \psi_{ac}+\psi_{ce}+\psi_{ev}+\psi_{cv}+\psi_{ac}\cdot\psi_{ce}+\psi_{ce}\cdot\psi_{ev}+\psi_{ce}\cdot\psi_{cv}+\psi_{ev}\cdot\psi_{cv}$ | 0.159513 | 0.92172 | -14801.6 |
| 14 | $\overline{u} \sim \psi_{ac}+\psi_{ce}+\psi_{ev}+\psi_{cv}+\psi_{ac}\cdot\psi_{ce}+\psi_{ce}\cdot\psi_{cv}$ | 0.159143 | 0.92167 | -14780.6 |
| 15 | $\overline{u} \sim \psi_{ac}+\psi_{ce}+\psi_{ev}+\psi_{cv}+\psi_{ac}\cdot\psi_{ce}+\psi_{ce}\cdot\psi_{cv}+\psi_{ev}\cdot\psi_{cv}$ | 0.159165 | 0.92167 | -14782.9 |
| 16 | $\overline{u} \sim \psi_{ac}+\psi_{ce}+\psi_{cv}+\psi_{ac}\cdot\psi_{ce}+\psi_{ac}\cdot\psi_{ev}+\psi_{ac}\cdot\psi_{cv}+\psi_{ce}\cdot\psi_{ev}+\psi_{ce}\cdot\psi_{cv}+\psi_{ev}\cdot\psi_{cv}$ | 0.170588 | 0.91628 | -12754.3 |
| 17 | $\overline{u} \sim \psi_{ac}+\psi_{ce}+\psi_{cv}+\psi_{ac}\cdot\psi_{ce}+\psi_{ac}\cdot\psi_{ev}+\psi_{ac}\cdot\psi_{cv}+\psi_{ce}\cdot\psi_{cv}+\psi_{ev}\cdot\psi_{cv}$ | 0.171269 | 0.91579 | -12575.5 |
| 18 | $\overline{u} \sim \psi_{ac}+\psi_{ce}+\psi_{cv}+\psi_{ac}\cdot\psi_{ce}+\psi_{ac}\cdot\psi_{ev}+\psi_{ac}\cdot\psi_{cv}+\psi_{ce}\cdot\psi_{ev}+\psi_{ce}\cdot\psi_{cv}$ | 0.169103 | 0.91532 | -12408.6 |
| 19 | $\overline{u} \sim \psi_{ac}+\psi_{ce}+\psi_{cv}+\psi_{ac}\cdot\psi_{ce}+\psi_{ac}\cdot\psi_{ev}+\psi_{ac}\cdot\psi_{cv}+\psi_{ce}\cdot\psi_{cv}$ | 0.16982 | 0.91491 | -12260 |
| 20 | $\overline{u} \sim \psi_{ac}+\psi_{ce}+\psi_{ev}+\psi_{ac}\cdot\psi_{ce}+\psi_{ac}\cdot\psi_{ev}+\psi_{ac}\cdot\psi_{cv}+\psi_{ce}\cdot\psi_{ev}+\psi_{ce}\cdot\psi_{cv}+\psi_{ev}\cdot\psi_{cv}$ | 0.156502 | 0.9144 | -12076 |
| 21 | $\overline{u} \sim \psi_{ac}+\psi_{ce}+\psi_{ev}+\psi_{ac}\cdot\psi_{ce}+\psi_{ac}\cdot\psi_{ev}+\psi_{ac}\cdot\psi_{cv}+\psi_{ce}\cdot\psi_{cv}+\psi_{ev}\cdot\psi_{cv}$ | 0.156563 | 0.91439 | -12073.5 |
| 22 | $\overline{u} \sim \psi_{ac}+\psi_{ce}+\psi_{cv}+\psi_{ac}\cdot\psi_{ce}+\psi_{ac}\cdot\psi_{ev}+\psi_{ce}\cdot\psi_{ev}+\psi_{ce}\cdot\psi_{cv}+\psi_{ev}\cdot\psi_{cv}$ | 0.151311 | 0.9135 | -11758.7 |
| 23 | $\overline{u} \sim \psi_{ac}+\psi_{ce}+\psi_{cv}+\psi_{ac}\cdot\psi_{ce}+\psi_{ac}\cdot\psi_{ev}+\psi_{ce}\cdot\psi_{cv}+\psi_{ev}\cdot\psi_{cv}$ | 0.151901 | 0.91299 | -11580.3 |

| | | | | |
|---|---|---|---|---|
| 24 | $\overline{\boldsymbol{u}} \sim \psi_{ac}+\psi_{ce}+\psi_{ev}+\psi_{ac}\cdot\psi_{ce}+\psi_{ac}\cdot\psi_{ev}+\psi_{ce}\cdot\psi_{ev}+\psi_{ce}\cdot\psi_{cv}+\psi_{ev}\cdot\psi_{cv}$ | 0.167547 | 0.91293 | -11561 |
| 25 | $\overline{\boldsymbol{u}} \sim \psi_{ac}+\psi_{ce}+\psi_{ev}+\psi_{ac}\cdot\psi_{ce}+\psi_{ac}\cdot\psi_{ev}+\psi_{ce}\cdot\psi_{cv}+\psi_{ev}\cdot\psi_{cv}$ | 0.167769 | 0.9129 | -11551.2 |
| 26 | $\overline{\boldsymbol{u}} \sim \psi_{ac}+\psi_{ce}+\psi_{ev}+\psi_{ac}\cdot\psi_{ce}+\psi_{ac}\cdot\psi_{cv}+\psi_{ce}\cdot\psi_{ev}+\psi_{ce}\cdot\psi_{cv}+\psi_{ev}\cdot\psi_{cv}$ | 0.150697 | 0.9126 | -11444.4 |
| 27 | $\overline{\boldsymbol{u}} \sim \psi_{ac}+\psi_{ce}+\psi_{cv}+\psi_{ac}\cdot\psi_{ce}+\psi_{ac}\cdot\psi_{ev}+\psi_{ce}\cdot\psi_{ev}+\psi_{ce}\cdot\psi_{cv}$ | 0.150335 | 0.91259 | -11442.2 |
| 28 | $\overline{\boldsymbol{u}} \sim \psi_{ac}+\psi_{ce}+\psi_{ev}+\psi_{ac}\cdot\psi_{ce}+\psi_{ac}\cdot\psi_{cv}+\psi_{ce}\cdot\psi_{cv}+\psi_{ev}\cdot\psi_{cv}$ | 0.150503 | 0.91258 | -11439 |
| 29 | $\overline{\boldsymbol{u}} \sim \psi_{ac}+\psi_{ce}+\psi_{cv}+\psi_{ac}\cdot\psi_{ce}+\psi_{ac}\cdot\psi_{ev}+\psi_{ce}\cdot\psi_{cv}$ | 0.150945 | 0.91216 | -11292.4 |
| 30 | $\overline{\boldsymbol{u}} \sim \psi_{ac}+\psi_{ce}+\psi_{cv}+\psi_{ac}\cdot\psi_{ce}+\psi_{ac}\cdot\psi_{cv}+\psi_{ce}\cdot\psi_{ev}+\psi_{ce}\cdot\psi_{cv}+\psi_{ev}\cdot\psi_{cv}$ | 0.165486 | 0.91139 | -11024.3 |
| 31 | $\overline{\boldsymbol{u}} \sim \psi_{ac}+\psi_{ce}+\psi_{ev}+\psi_{ac}\cdot\psi_{ce}+\psi_{ce}\cdot\psi_{ev}+\psi_{ce}\cdot\psi_{cv}+\psi_{ev}\cdot\psi_{cv}$ | 0.162991 | 0.91019 | -10617 |
| 32 | $\overline{\boldsymbol{u}} \sim \psi_{ac}+\psi_{ce}+\psi_{ev}+\psi_{ac}\cdot\psi_{ce}+\psi_{ce}\cdot\psi_{cv}+\psi_{ev}\cdot\psi_{cv}$ | 0.162786 | 0.91018 | -10614 |
| 33 | $\overline{\boldsymbol{u}} \sim \psi_{ac}+\psi_{ce}+\psi_{ev}+\psi_{ac}\cdot\psi_{ce}+\psi_{ac}\cdot\psi_{ev}+\psi_{ac}\cdot\psi_{cv}+\psi_{ce}\cdot\psi_{cv}$ | 0.1445 | 0.90922 | -10289.3 |
| 34 | $\overline{\boldsymbol{u}} \sim \psi_{ac}+\psi_{ce}+\psi_{ev}+\psi_{ac}\cdot\psi_{ce}+\psi_{ac}\cdot\psi_{ev}+\psi_{ac}\cdot\psi_{cv}+\psi_{ce}\cdot\psi_{ev}+\psi_{ce}\cdot\psi_{cv}$ | 0.144571 | 0.90922 | -10290 |
| 35 | $\overline{\boldsymbol{u}} \sim \psi_{ac}+\psi_{ce}+\psi_{ev}+\psi_{ac}\cdot\psi_{ce}+\psi_{ac}\cdot\psi_{cv}+\psi_{ce}\cdot\psi_{ev}+\psi_{ce}\cdot\psi_{cv}$ | 0.142962 | 0.90883 | -10158.3 |
| 36 | $\overline{\boldsymbol{u}} \sim \psi_{ac}+\psi_{ce}+\psi_{ev}+\psi_{ac}\cdot\psi_{ce}+\psi_{ac}\cdot\psi_{cv}+\psi_{ce}\cdot\psi_{cv}$ | 0.142711 | 0.90879 | -10146.7 |
| 37 | $\overline{\boldsymbol{u}} \sim \psi_{ac}+\psi_{ce}+\psi_{cv}+\psi_{ac}\cdot\psi_{ce}+\psi_{ce}\cdot\psi_{ev}+\psi_{ce}\cdot\psi_{cv}+\psi_{ev}\cdot\psi_{cv}$ | 0.148126 | 0.90867 | -10106.1 |
| 38 | $\overline{\boldsymbol{u}} \sim \psi_{ac}+\psi_{ce}+\psi_{ac}\cdot\psi_{ce}+\psi_{ac}\cdot\psi_{ev}+\psi_{ac}\cdot\psi_{cv}+\psi_{ce}\cdot\psi_{ev}+\psi_{ce}\cdot\psi_{cv}+\psi_{ev}\cdot\psi_{cv}$ | 0.148069 | 0.90744 | -9696.95 |
| 39 | $\overline{\boldsymbol{u}} \sim \psi_{ac}+\psi_{ce}+\psi_{ac}\cdot\psi_{ce}+\psi_{ac}\cdot\psi_{ev}+\psi_{ac}\cdot\psi_{cv}+\psi_{ce}\cdot\psi_{cv}+\psi_{ev}\cdot\psi_{cv}$ | 0.148542 | 0.90679 | -9485.75 |
| 40 | $\overline{\boldsymbol{u}} \sim \psi_{ac}+\psi_{ce}+\psi_{ac}\cdot\psi_{ce}+\psi_{ac}\cdot\psi_{ev}+\psi_{ce}\cdot\psi_{ev}+\psi_{ce}\cdot\psi_{cv}+\psi_{ev}\cdot\psi_{cv}$ | 0.155571 | 0.9065 | -9389.89 |
| 41 | $\overline{\boldsymbol{u}} \sim \psi_{ac}+\psi_{ce}+\psi_{ac}\cdot\psi_{ce}+\psi_{ac}\cdot\psi_{cv}+\psi_{ce}\cdot\psi_{ev}+\psi_{ce}\cdot\psi_{cv}+\psi_{ev}\cdot\psi_{cv}$ | 0.149244 | 0.90605 | -9246.1 |
| 42 | $\overline{\boldsymbol{u}} \sim \psi_{ac}+\psi_{ce}+\psi_{ac}\cdot\psi_{ce}+\psi_{ce}\cdot\psi_{ev}+\psi_{ce}\cdot\psi_{cv}+\psi_{ev}\cdot\psi_{cv}$ | 0.152983 | 0.90584 | -9177.41 |
| 43 | $\overline{\boldsymbol{u}} \sim \psi_{ac}+\psi_{ce}+\psi_{ac}\cdot\psi_{ce}+\psi_{ac}\cdot\psi_{ev}+\psi_{ce}\cdot\psi_{cv}+\psi_{ev}\cdot\psi_{cv}$ | 0.156253 | 0.9058 | -9164.55 |
| 44 | $\overline{\boldsymbol{u}} \sim \psi_{ac}+\psi_{ce}+\psi_{cv}+\psi_{ac}\cdot\psi_{ce}+\psi_{ac}\cdot\psi_{cv}+\psi_{ce}\cdot\psi_{cv}+\psi_{ev}\cdot\psi_{cv}$ | 0.164537 | 0.90456 | -8765.44 |
| 45 | $\overline{\boldsymbol{u}} \sim \psi_{ac}+\psi_{ce}+\psi_{cv}+\psi_{ac}\cdot\psi_{ce}+\psi_{ce}\cdot\psi_{cv}+\psi_{ev}\cdot\psi_{cv}$ | 0.147987 | 0.90183 | -7907.32 |
| 46 | $\overline{\boldsymbol{u}} \sim \psi_{ac}+\psi_{ce}+\psi_{ac}\cdot\psi_{ce}+\psi_{ac}\cdot\psi_{cv}+\psi_{ce}\cdot\psi_{cv}+\psi_{ev}\cdot\psi_{cv}$ | 0.152625 | 0.90179 | -7895.28 |
| 47 | $\overline{\boldsymbol{u}} \sim \psi_{ac}+\psi_{ce}+\psi_{ac}\cdot\psi_{ce}+\psi_{ce}\cdot\psi_{cv}+\psi_{ev}\cdot\psi_{cv}$ | 0.149376 | 0.9016 | -7839.1 |
| 48 | $\overline{\boldsymbol{u}} \sim \psi_{ac}+\psi_{ce}+\psi_{ev}+\psi_{cv}+\psi_{ac}\cdot\psi_{ce}+\psi_{ac}\cdot\psi_{ev}+\psi_{ac}\cdot\psi_{cv}+\psi_{ev}\cdot\psi_{cv}$ | 0.230367 | 0.90123 | -7719.87 |
| 49 | $\overline{\boldsymbol{u}} \sim \psi_{ac}+\psi_{ce}+\psi_{ev}+\psi_{cv}+\psi_{ac}\cdot\psi_{ce}+\psi_{ac}\cdot\psi_{ev}+\psi_{ac}\cdot\psi_{cv}+\psi_{ce}\cdot\psi_{ev}+\psi_{ev}\cdot\psi_{cv}$ | 0.230507 | 0.90123 | -7720.43 |
| 50 | $\overline{\boldsymbol{u}} \sim \psi_{ac}+\psi_{ce}+\psi_{ev}+\psi_{cv}+\psi_{ac}\cdot\psi_{ce}+\psi_{ac}\cdot\psi_{ev}+\psi_{ac}\cdot\psi_{cv}+\psi_{ce}\cdot\psi_{ev}$ | 0.22954 | 0.90108 | -7673.88 |

| | | | | |
|---|---|---|---|---|
| 51 | $\overline{\boldsymbol{u}} \sim \psi_{ac}+\psi_{ce}+\psi_{ev}+\psi_{cv}+\psi_{ac}\cdot\psi_{ce}+\psi_{ac}\cdot\psi_{ev}+\psi_{ac}\cdot\psi_{cv}$ | 0.229309 | 0.90106 | -7670.44 |
| 52 | $\overline{\boldsymbol{u}} \sim \psi_{ac}+\psi_{ce}+\psi_{ev}+\psi_{cv}+\psi_{ac}\cdot\psi_{ce}+\psi_{ac}\cdot\psi_{cv}+\psi_{ce}\cdot\psi_{ev}+\psi_{ev}\cdot\psi_{cv}$ | 0.225001 | 0.90049 | -7493.17 |
| 53 | $\overline{\boldsymbol{u}} \sim \psi_{ac}+\psi_{ce}+\psi_{ev}+\psi_{cv}+\psi_{ac}\cdot\psi_{ce}+\psi_{ac}\cdot\psi_{cv}+\psi_{ce}\cdot\psi_{ev}$ | 0.224966 | 0.90048 | -7492.06 |
| 54 | $\overline{\boldsymbol{u}} \sim \psi_{ac}+\psi_{ce}+\psi_{ev}+\psi_{cv}+\psi_{ac}\cdot\psi_{ce}+\psi_{ac}\cdot\psi_{cv}+\psi_{ev}\cdot\psi_{cv}$ | 0.22428 | 0.90042 | -7473.57 |
| 55 | $\overline{\boldsymbol{u}} \sim \psi_{ac}+\psi_{ce}+\psi_{ev}+\psi_{cv}+\psi_{ac}\cdot\psi_{ce}+\psi_{ac}\cdot\psi_{cv}$ | 0.224233 | 0.90041 | -7471.8 |
| 56 | $\overline{\boldsymbol{u}} \sim \psi_{ac}+\psi_{ce}+\psi_{ac}\cdot\psi_{ce}+\psi_{ac}\cdot\psi_{ev}+\psi_{ac}\cdot\psi_{cv}+\psi_{ce}\cdot\psi_{ev}+\psi_{ce}\cdot\psi_{cv}$ | 0.136282 | 0.90009 | -7371.45 |
| 57 | $\overline{\boldsymbol{u}} \sim \psi_{ac}+\psi_{ce}+\psi_{ac}\cdot\psi_{ce}+\psi_{ac}\cdot\psi_{ev}+\psi_{ac}\cdot\psi_{cv}+\psi_{ce}\cdot\psi_{cv}$ | 0.136869 | 0.89964 | -7236 |
| 58 | $\overline{\boldsymbol{u}} \sim \psi_{ac}+\psi_{ce}+\psi_{ev}+\psi_{ac}\cdot\psi_{ce}+\psi_{ac}\cdot\psi_{ev}+\psi_{ce}\cdot\psi_{cv}$ | 0.168657 | 0.89515 | -5903.91 |
| 59 | $\overline{\boldsymbol{u}} \sim \psi_{ac}+\psi_{ce}+\psi_{ev}+\psi_{ac}\cdot\psi_{ce}+\psi_{ac}\cdot\psi_{ev}+\psi_{ce}\cdot\psi_{ev}+\psi_{ce}\cdot\psi_{cv}$ | 0.168754 | 0.89515 | -5904.64 |
| 60 | $\overline{\boldsymbol{u}} \sim \psi_{ac}+\psi_{ce}+\psi_{ev}+\psi_{ac}\cdot\psi_{ce}+\psi_{ce}\cdot\psi_{ev}+\psi_{ce}\cdot\psi_{cv}$ | 0.166592 | 0.89473 | -5783.29 |
| 61 | $\overline{\boldsymbol{u}} \sim \psi_{ac}+\psi_{ce}+\psi_{ev}+\psi_{ac}\cdot\psi_{ce}+\psi_{ce}\cdot\psi_{cv}$ | 0.166284 | 0.89469 | -5772.23 |
| 62 | $\overline{\boldsymbol{u}} \sim \psi_{ac}+\psi_{ce}+\psi_{ev}+\psi_{ac}\cdot\psi_{ce}+\psi_{ac}\cdot\psi_{ev}+\psi_{ac}\cdot\psi_{cv}+\psi_{ev}\cdot\psi_{cv}$ | 0.195216 | 0.8944 | -5685.89 |
| 63 | $\overline{\boldsymbol{u}} \sim \psi_{ac}+\psi_{ce}+\psi_{ev}+\psi_{ac}\cdot\psi_{ce}+\psi_{ac}\cdot\psi_{ev}+\psi_{ac}\cdot\psi_{cv}+\psi_{ce}\cdot\psi_{ev}+\psi_{ev}\cdot\psi_{cv}$ | 0.195178 | 0.8944 | -5685.13 |
| 64 | $\overline{\boldsymbol{u}} \sim \psi_{ac}+\psi_{ce}+\psi_{cv}+\psi_{ac}\cdot\psi_{ce}+\psi_{ac}\cdot\psi_{cv}+\psi_{ce}\cdot\psi_{ev}+\psi_{ce}\cdot\psi_{cv}$ | 0.150012 | 0.89402 | -5578.06 |
| 65 | $\overline{\boldsymbol{u}} \sim \psi_{ac}+\psi_{ce}+\psi_{ev}+\psi_{ac}\cdot\psi_{ce}+\psi_{ac}\cdot\psi_{cv}+\psi_{ce}\cdot\psi_{ev}+\psi_{ev}\cdot\psi_{cv}$ | 0.188023 | 0.89287 | -5248.2 |
| 66 | $\overline{\boldsymbol{u}} \sim \psi_{ac}+\psi_{ce}+\psi_{ev}+\psi_{ac}\cdot\psi_{ce}+\psi_{ac}\cdot\psi_{cv}+\psi_{ev}\cdot\psi_{cv}$ | 0.187739 | 0.89284 | -5241.61 |
| 67 | $\overline{\boldsymbol{u}} \sim \psi_{ac}+\psi_{ce}+\psi_{cv}+\psi_{ac}\cdot\psi_{ce}+\psi_{ce}\cdot\psi_{ev}+\psi_{ce}\cdot\psi_{cv}$ | 0.137192 | 0.8917 | -4918.9 |
| 68 | $\overline{\boldsymbol{u}} \sim \psi_{ac}+\psi_{ce}+\psi_{cv}+\psi_{ac}\cdot\psi_{ce}+\psi_{ac}\cdot\psi_{ev}+\psi_{ac}\cdot\psi_{cv}+\psi_{ce}\cdot\psi_{ev}+\psi_{ev}\cdot\psi_{cv}$ | 0.203645 | 0.8917 | -4916.73 |
| 69 | $\overline{\boldsymbol{u}} \sim \psi_{ac}+\psi_{ce}+\psi_{ev}+\psi_{ac}\cdot\psi_{ce}+\psi_{ac}\cdot\psi_{ev}+\psi_{ev}\cdot\psi_{cv}$ | 0.186655 | 0.89152 | -4868.41 |
| 70 | $\overline{\boldsymbol{u}} \sim \psi_{ac}+\psi_{ce}+\psi_{ev}+\psi_{cv}+\psi_{ac}\cdot\psi_{ce}+\psi_{ac}\cdot\psi_{ev}+\psi_{ev}\cdot\psi_{cv}$ | 0.186603 | 0.89152 | -4866.76 |
| 71 | $\overline{\boldsymbol{u}} \sim \psi_{ac}+\psi_{ce}+\psi_{ev}+\psi_{ac}\cdot\psi_{ce}+\psi_{ac}\cdot\psi_{ev}+\psi_{ce}\cdot\psi_{ev}+\psi_{ev}\cdot\psi_{cv}$ | 0.186709 | 0.89152 | -4867.72 |
| 72 | $\overline{\boldsymbol{u}} \sim \psi_{ac}+\psi_{ce}+\psi_{ev}+\psi_{cv}+\psi_{ac}\cdot\psi_{ce}+\psi_{ac}\cdot\psi_{ev}+\psi_{ce}\cdot\psi_{ev}+\psi_{ev}\cdot\psi_{cv}$ | 0.186664 | 0.89152 | -4865.96 |
| 73 | $\overline{\boldsymbol{u}} \sim \psi_{ac}+\psi_{ce}+\psi_{ev}+\psi_{cv}+\psi_{ac}\cdot\psi_{ce}+\psi_{ac}\cdot\psi_{ev}$ | 0.186057 | 0.8914 | -4833.2 |
| 74 | $\overline{\boldsymbol{u}} \sim \psi_{ac}+\psi_{ce}+\psi_{ev}+\psi_{cv}+\psi_{ac}\cdot\psi_{ce}+\psi_{ac}\cdot\psi_{ev}+\psi_{ce}\cdot\psi_{ev}$ | 0.186166 | 0.8914 | -4834.13 |
| 75 | $\overline{\boldsymbol{u}} \sim \psi_{ac}+\psi_{ce}+\psi_{cv}+\psi_{ac}\cdot\psi_{ce}+\psi_{ac}\cdot\psi_{ev}+\psi_{ac}\cdot\psi_{cv}+\psi_{ev}\cdot\psi_{cv}$ | 0.204307 | 0.89123 | -4785.73 |
| 76 | $\overline{\boldsymbol{u}} \sim \psi_{ac}+\psi_{ce}+\psi_{ev}+\psi_{cv}+\psi_{ac}\cdot\psi_{ce}+\psi_{ce}\cdot\psi_{ev}+\psi_{ev}\cdot\psi_{cv}$ | 0.18386 | 0.89097 | -4711.73 |
| 77 | $\overline{\boldsymbol{u}} \sim \psi_{ac}+\psi_{ce}+\psi_{ev}+\psi_{cv}+\psi_{ac}\cdot\psi_{ce}+\psi_{ce}\cdot\psi_{ev}$ | 0.183842 | 0.89096 | -4711.46 |

| | | | | |
|---|---|---|---|---|
| 78 | $\overline{\boldsymbol{u}} \sim \psi_{ac}+\psi_{ce}+\psi_{ev}+\psi_{cv}+\psi_{ac}\cdot\psi_{ce}$ | 0.183473 | 0.89092 | -4699.85 |
| 79 | $\overline{\boldsymbol{u}} \sim \psi_{ac}+\psi_{ce}+\psi_{ev}+\psi_{cv}+\psi_{ac}\cdot\psi_{ce}+\psi_{ev}\cdot\psi_{cv}$ | 0.183499 | 0.89092 | -4700.57 |
| 80 | $\overline{\boldsymbol{u}} \sim \psi_{ac}+\psi_{ce}+\psi_{ev}+\psi_{ac}\cdot\psi_{ce}+\psi_{ce}\cdot\psi_{ev}+\psi_{ev}\cdot\psi_{cv}$ | 0.183265 | 0.89092 | -4699.97 |
| 81 | $\overline{\boldsymbol{u}} \sim \psi_{ac}+\psi_{ce}+\psi_{ev}+\psi_{ac}\cdot\psi_{ce}+\psi_{ev}\cdot\psi_{cv}$ | 0.182938 | 0.89088 | -4689.8 |
| 82 | $\overline{\boldsymbol{u}} \sim \psi_{ac}+\psi_{ce}+\psi_{ev}+\psi_{ac}\cdot\psi_{ce}+\psi_{ac}\cdot\psi_{ev}$ | 0.181066 | 0.89083 | -4675.15 |
| 83 | $\overline{\boldsymbol{u}} \sim \psi_{ac}+\psi_{ce}+\psi_{ev}+\psi_{ac}\cdot\psi_{ce}+\psi_{ac}\cdot\psi_{ev}+\psi_{ce}\cdot\psi_{ev}$ | 0.181165 | 0.89083 | -4675.92 |
| 84 | $\overline{\boldsymbol{u}} \sim \psi_{ac}+\psi_{ce}+\psi_{ev}+\psi_{ac}\cdot\psi_{ce}+\psi_{ac}\cdot\psi_{ev}+\psi_{ac}\cdot\psi_{cv}+\psi_{ce}\cdot\psi_{ev}$ | 0.181168 | 0.89083 | -4673.92 |
| 85 | $\overline{\boldsymbol{u}} \sim \psi_{ac}+\psi_{ce}+\psi_{ev}+\psi_{ac}\cdot\psi_{ce}+\psi_{ac}\cdot\psi_{ev}+\psi_{ac}\cdot\psi_{cv}$ | 0.181068 | 0.89082 | -4673.15 |
| 86 | $\overline{\boldsymbol{u}} \sim \psi_{ac}+\psi_{ce}+\psi_{cv}+\psi_{ac}\cdot\psi_{ce}+\psi_{ac}\cdot\psi_{ev}+\psi_{ac}\cdot\psi_{cv}+\psi_{ce}\cdot\psi_{ev}$ | 0.201881 | 0.89077 | -4657.98 |
| 87 | $\overline{\boldsymbol{u}} \sim \psi_{ac}+\psi_{ce}+\psi_{ev}+\psi_{ac}\cdot\psi_{ce}+\psi_{ce}\cdot\psi_{ev}$ | 0.17901 | 0.8904 | -4556.95 |
| 88 | $\overline{\boldsymbol{u}} \sim \psi_{ac}+\psi_{ce}+\psi_{ev}+\psi_{ac}\cdot\psi_{ce}+\psi_{ac}\cdot\psi_{cv}+\psi_{ce}\cdot\psi_{ev}$ | 0.179006 | 0.8904 | -4554.95 |
| 89 | $\overline{\boldsymbol{u}} \sim \psi_{ac}+\psi_{ce}+\psi_{cv}+\psi_{ac}\cdot\psi_{ce}+\psi_{ac}\cdot\psi_{ev}+\psi_{ac}\cdot\psi_{cv}$ | 0.2026 | 0.89038 | -4549.47 |
| 90 | $\overline{\boldsymbol{u}} \sim \psi_{ac}+\psi_{ce}+\psi_{ev}+\psi_{ac}\cdot\psi_{ce}$ | 0.178669 | 0.89036 | -4545.9 |
| 91 | $\overline{\boldsymbol{u}} \sim \psi_{ac}+\psi_{ce}+\psi_{ev}+\psi_{ac}\cdot\psi_{ce}+\psi_{ac}\cdot\psi_{cv}$ | 0.178664 | 0.89036 | -4543.9 |
| 92 | $\overline{\boldsymbol{u}} \sim \psi_{ac}+\psi_{ce}+\psi_{ac}\cdot\psi_{ce}+\psi_{ac}\cdot\psi_{ev}+\psi_{ac}\cdot\psi_{cv}+\psi_{ce}\cdot\psi_{ev}+\psi_{ev}\cdot\psi_{cv}$ | 0.181774 | 0.88678 | -3566.19 |
| 93 | $\overline{\boldsymbol{u}} \sim \psi_{ac}+\psi_{ce}+\psi_{cv}+\psi_{ac}\cdot\psi_{ce}+\psi_{ac}\cdot\psi_{cv}+\psi_{ce}\cdot\psi_{ev}+\psi_{ev}\cdot\psi_{cv}$ | 0.194638 | 0.88643 | -3471.79 |
| 94 | $\overline{\boldsymbol{u}} \sim \psi_{ac}+\psi_{ce}+\psi_{ac}\cdot\psi_{ce}+\psi_{ac}\cdot\psi_{ev}+\psi_{ac}\cdot\psi_{cv}+\psi_{ev}\cdot\psi_{cv}$ | 0.182183 | 0.8862 | -3411.1 |
| 95 | $\overline{\boldsymbol{u}} \sim \psi_{ac}+\psi_{ce}+\psi_{ac}\cdot\psi_{ce}+\psi_{ac}\cdot\psi_{ev}+\psi_{ce}\cdot\psi_{ev}+\psi_{ce}\cdot\psi_{cv}$ | 0.156655 | 0.88587 | -3320.94 |
| 96 | $\overline{\boldsymbol{u}} \sim \psi_{ac}+\psi_{ce}+\psi_{ac}\cdot\psi_{ce}+\psi_{ac}\cdot\psi_{ev}+\psi_{ce}\cdot\psi_{cv}$ | 0.157291 | 0.88544 | -3208.05 |
| 97 | $\overline{\boldsymbol{u}} \sim \psi_{ac}+\psi_{ce}+\psi_{ac}\cdot\psi_{ce}+\psi_{ac}\cdot\psi_{cv}+\psi_{ce}\cdot\psi_{ev}+\psi_{ev}\cdot\psi_{cv}$ | 0.182453 | 0.88437 | -2923.68 |
| 98 | $\overline{\boldsymbol{u}} \sim \psi_{ce}+\psi_{ev}+\psi_{cv}+\psi_{ac}\cdot\psi_{ce}+\psi_{ce}\cdot\psi_{ev}+\psi_{ce}\cdot\psi_{cv}+\psi_{ev}\cdot\psi_{cv}$ | 0.927641 | 0.88421 | -2880.41 |
| 99 | $\overline{\boldsymbol{u}} \sim \psi_{ce}+\psi_{ev}+\psi_{cv}+\psi_{ac}\cdot\psi_{ce}+\psi_{ce}\cdot\psi_{ev}+\psi_{ce}\cdot\psi_{cv}$ | 0.927641 | 0.88419 | -2877.97 |
| 100 | $\overline{\boldsymbol{u}} \sim \psi_{ce}+\psi_{ev}+\psi_{cv}+\psi_{ac}\cdot\psi_{ce}+\psi_{ce}\cdot\psi_{cv}+\psi_{ev}\cdot\psi_{cv}$ | 0.927645 | 0.88394 | -2812.74 |
| 101 | $\overline{\boldsymbol{u}} \sim \psi_{ce}+\psi_{ev}+\psi_{cv}+\psi_{ac}\cdot\psi_{ce}+\psi_{ce}\cdot\psi_{cv}$ | 0.927646 | 0.88393 | -2809.09 |
| 102 | $\overline{\boldsymbol{u}} \sim \psi_{ce}+\psi_{ev}+\psi_{ac}\cdot\psi_{ce}+\psi_{ce}\cdot\psi_{ev}+\psi_{ce}\cdot\psi_{cv}+\psi_{ev}\cdot\psi_{cv}$ | 0.929702 | 0.8838 | -2775.81 |
| 103 | $\overline{\boldsymbol{u}} \sim \psi_{ce}+\psi_{ev}+\psi_{ac}\cdot\psi_{ce}+\psi_{ce}\cdot\psi_{ev}+\psi_{ce}\cdot\psi_{cv}$ | 0.929615 | 0.88376 | -2765.15 |
| 104 | $\overline{\boldsymbol{u}} \sim \psi_{ce}+\psi_{ev}+\psi_{ac}\cdot\psi_{ce}+\psi_{ce}\cdot\psi_{cv}+\psi_{ev}\cdot\psi_{cv}$ | 0.929711 | 0.88355 | -2709.51 |

| | | | | |
|---|---|---|---|---|
| 105 | $\overline{\boldsymbol{u}} \sim \psi_{ce}+\psi_{ev}+\psi_{ac}\cdot\psi_{ce}+\psi_{ce}\cdot\psi_{cv}$ | 0.929618 | 0.88349 | -2696.9 |
| 106 | $\overline{\boldsymbol{u}} \sim \psi_{ac}+\psi_{ce}+\psi_{cv}+\psi_{ac}\cdot\psi_{ce}+\psi_{ac}\cdot\psi_{ev}+\psi_{ce}\cdot\psi_{ev}+\psi_{ev}\cdot\psi_{cv}$ | 0.172991 | 0.8828 | -2513.09 |
| 107 | $\overline{\boldsymbol{u}} \sim \psi_{ac}+\psi_{ce}+\psi_{ac}\cdot\psi_{ce}+\psi_{ac}\cdot\psi_{ev}+\psi_{ce}\cdot\psi_{ev}+\psi_{ev}\cdot\psi_{cv}$ | 0.173717 | 0.88248 | -2431.57 |
| 108 | $\overline{\boldsymbol{u}} \sim \psi_{ac}+\psi_{ce}+\psi_{cv}+\psi_{ac}\cdot\psi_{ce}+\psi_{ac}\cdot\psi_{ev}+\psi_{ev}\cdot\psi_{cv}$ | 0.173577 | 0.88231 | -2387.61 |
| 109 | $\overline{\boldsymbol{u}} \sim \psi_{ac}+\psi_{ce}+\psi_{ac}\cdot\psi_{ce}+\psi_{ac}\cdot\psi_{ev}+\psi_{ev}\cdot\psi_{cv}$ | 0.174247 | 0.88204 | -2317.57 |
| 110 | $\overline{\boldsymbol{u}} \sim \psi_{ac}+\psi_{ce}+\psi_{cv}+\psi_{ac}\cdot\psi_{ce}+\psi_{ac}\cdot\psi_{ev}+\psi_{ce}\cdot\psi_{ev}$ | 0.171938 | 0.88196 | -2297.86 |
| 111 | $\overline{\boldsymbol{u}} \sim \psi_{ac}+\psi_{ce}+\psi_{cv}+\psi_{ac}\cdot\psi_{ce}+\psi_{ac}\cdot\psi_{ev}$ | 0.172553 | 0.88155 | -2192.15 |
| 112 | $\overline{\boldsymbol{u}} \sim \psi_{ac}+\psi_{ce}+\psi_{ac}\cdot\psi_{ce}+\psi_{ac}\cdot\psi_{ev}+\psi_{ce}\cdot\psi_{ev}$ | 0.168097 | 0.88146 | -2169.79 |
| 113 | $\overline{\boldsymbol{u}} \sim \psi_{ac}+\psi_{ce}+\psi_{ac}\cdot\psi_{ce}+\psi_{ac}\cdot\psi_{ev}+\psi_{ac}\cdot\psi_{cv}+\psi_{ce}\cdot\psi_{ev}$ | 0.168058 | 0.88146 | -2167.84 |
| 114 | $\overline{\boldsymbol{u}} \sim \psi_{ac}+\psi_{ce}+\psi_{ac}\cdot\psi_{ce}+\psi_{ac}\cdot\psi_{ev}$ | 0.168694 | 0.88105 | -2063.72 |
| 115 | $\overline{\boldsymbol{u}} \sim \psi_{ac}+\psi_{ce}+\psi_{ac}\cdot\psi_{ce}+\psi_{ac}\cdot\psi_{ev}+\psi_{ac}\cdot\psi_{cv}$ | 0.168659 | 0.88104 | -2061.76 |
| 116 | $\overline{\boldsymbol{u}} \sim \psi_{ac}+\psi_{ce}+\psi_{ac}\cdot\psi_{ce}+\psi_{ac}\cdot\psi_{cv}+\psi_{ce}\cdot\psi_{ev}+\psi_{ce}\cdot\psi_{cv}$ | 0.125891 | 0.8795 | -1669.86 |
| 117 | $\overline{\boldsymbol{u}} \sim \psi_{ac}+\psi_{ce}+\psi_{cv}+\psi_{ac}\cdot\psi_{ce}+\psi_{ac}\cdot\psi_{cv}+\psi_{ev}\cdot\psi_{cv}$ | 0.191606 | 0.87942 | -1649.41 |
| 118 | $\overline{\boldsymbol{u}} \sim \psi_{ac}+\psi_{ce}+\psi_{ac}\cdot\psi_{ce}+\psi_{ac}\cdot\psi_{cv}+\psi_{ev}\cdot\psi_{cv}$ | 0.185505 | 0.87889 | -1516.24 |
| 119 | $\overline{\boldsymbol{u}} \sim \psi_{ac}+\psi_{ce}+\psi_{cv}+\psi_{ac}\cdot\psi_{ce}+\psi_{ce}\cdot\psi_{ev}+\psi_{ev}\cdot\psi_{cv}$ | 0.167899 | 0.87766 | -1207.08 |
| 120 | $\overline{\boldsymbol{u}} \sim \psi_{ce}+\psi_{ev}+\psi_{ac}\cdot\psi_{ce}+\psi_{ac}\cdot\psi_{cv}+\psi_{ce}\cdot\psi_{ev}$ | 1.04449 | 0.87437 | -400.94 |
| 121 | $\overline{\boldsymbol{u}} \sim \psi_{ce}+\psi_{ev}+\psi_{ac}\cdot\psi_{ce}+\psi_{ac}\cdot\psi_{cv}$ | 1.04447 | 0.8741 | -336.806 |
| 122 | $\overline{\boldsymbol{u}} \sim \psi_{ac}+\psi_{ce}+\psi_{cv}+\psi_{ac}\cdot\psi_{ce}+\psi_{ev}\cdot\psi_{cv}$ | 0.16666 | 0.87065 | 489.286 |
| 123 | $\overline{\boldsymbol{u}} \sim \psi_{ac}+\psi_{ce}+\psi_{cv}+\psi_{ac}\cdot\psi_{ce}+\psi_{ac}\cdot\psi_{cv}+\psi_{ce}\cdot\psi_{ev}$ | 0.17382 | 0.86865 | 956.841 |
| 124 | $\overline{\boldsymbol{u}} \sim \psi_{ac}+\psi_{ce}+\psi_{ac}\cdot\psi_{ce}+\psi_{ce}\cdot\psi_{ev}+\psi_{ev}\cdot\psi_{cv}$ | 0.167826 | 0.86808 | 1087.21 |
| 125 | $\overline{\boldsymbol{u}} \sim \psi_{ac}+\psi_{ce}+\psi_{ac}\cdot\psi_{ce}+\psi_{ce}\cdot\psi_{ev}+\psi_{ce}\cdot\psi_{cv}$ | 0.141754 | 0.86484 | 1825.79 |
| 126 | $\overline{\boldsymbol{u}} \sim \psi_{ac}+\psi_{ce}+\psi_{cv}+\psi_{ac}\cdot\psi_{ce}+\psi_{ce}\cdot\psi_{ev}$ | 0.154151 | 0.86057 | 2773.55 |
| 127 | $\overline{\boldsymbol{u}} \sim \psi_{ac}+\psi_{ce}+\psi_{ac}\cdot\psi_{ce}+\psi_{ce}\cdot\psi_{ev}$ | 0.151533 | 0.86015 | 2862.42 |
| 128 | $\overline{\boldsymbol{u}} \sim \psi_{ac}+\psi_{ce}+\psi_{ac}\cdot\psi_{ce}+\psi_{ac}\cdot\psi_{cv}+\psi_{ce}\cdot\psi_{ev}$ | 0.151416 | 0.86015 | 2863.88 |
| 129 | $\overline{\boldsymbol{u}} \sim \psi_{ev}+\psi_{cv}+\psi_{ac}\cdot\psi_{ce}+\psi_{ce}\cdot\psi_{ev}+\psi_{ce}\cdot\psi_{cv}+\psi_{ev}\cdot\psi_{cv}$ | 1.01692 | 0.8588 | 3157 |
| 130 | $\overline{\boldsymbol{u}} \sim \psi_{ev}+\psi_{cv}+\psi_{ac}\cdot\psi_{ce}+\psi_{ce}\cdot\psi_{ev}+\psi_{ce}\cdot\psi_{cv}$ | 1.01694 | 0.85878 | 3160.22 |
| 131 | $\overline{\boldsymbol{u}} \sim \psi_{ce}+\psi_{ev}+\psi_{cv}+\psi_{ac}\cdot\psi_{ce}+\psi_{ce}\cdot\psi_{ev}+\psi_{ev}\cdot\psi_{cv}$ | 0.949976 | 0.85854 | 3214.12 |

| | | | | |
|---|---|---|---|---|
| 132 | $\overline{\boldsymbol{u}} \sim \psi_{ce}+\psi_{ev}+\psi_{cv}+\psi_{ac}\cdot\psi_{ce}+\psi_{ce}\cdot\psi_{ev}$ | 0.949976 | 0.85853 | 3215.33 |
| 133 | $\overline{\boldsymbol{u}} \sim \psi_{ce}+\psi_{ev}+\psi_{cv}+\psi_{ac}\cdot\psi_{ce}+\psi_{ev}\cdot\psi_{cv}$ | 0.949986 | 0.85832 | 3259.33 |
| 134 | $\overline{\boldsymbol{u}} \sim \psi_{ce}+\psi_{ev}+\psi_{cv}+\psi_{ac}\cdot\psi_{ce}$ | 0.949986 | 0.85831 | 3261.4 |
| 135 | $\overline{\boldsymbol{u}} \sim \psi_{ce}+\psi_{ev}+\psi_{ac}\cdot\psi_{ce}+\psi_{ce}\cdot\psi_{ev}+\psi_{ev}\cdot\psi_{cv}$ | 0.939389 | 0.8564 | 3670.94 |
| 136 | $\overline{\boldsymbol{u}} \sim \psi_{ce}+\psi_{ev}+\psi_{ac}\cdot\psi_{ce}+\psi_{ce}\cdot\psi_{ev}$ | 0.938966 | 0.85638 | 3673.1 |
| 137 | $\overline{\boldsymbol{u}} \sim \psi_{ce}+\psi_{ev}+\psi_{ac}\cdot\psi_{ce}+\psi_{ev}\cdot\psi_{cv}$ | 0.939345 | 0.85618 | 3716.69 |
| 138 | $\overline{\boldsymbol{u}} \sim \psi_{ce}+\psi_{ev}+\psi_{ac}\cdot\psi_{ce}$ | 0.938969 | 0.85616 | 3717.98 |
| 139 | $\overline{\boldsymbol{u}} \sim \psi_{ce}+\psi_{ac}\cdot\psi_{ce}+\psi_{ac}\cdot\psi_{ev}+\psi_{ac}\cdot\psi_{cv}$ | 1.18704 | 0.84967 | 5063.97 |
| 140 | $\overline{\boldsymbol{u}} \sim \psi_{ce}+\psi_{cv}+\psi_{ac}\cdot\psi_{ce}+\psi_{ac}\cdot\psi_{ev}+\psi_{ce}\cdot\psi_{cv}$ | 1.12845 | 0.82964 | 8872.02 |
| 141 | $\overline{\boldsymbol{u}} \sim \psi_{ce}+\psi_{ac}\cdot\psi_{ce}+\psi_{ac}\cdot\psi_{ev}+\psi_{ce}\cdot\psi_{cv}$ | 1.13023 | 0.82853 | 9068.7 |
| 142 | $\overline{\boldsymbol{u}} \sim \psi_{ac}+\psi_{ce}+\psi_{ac}\cdot\psi_{ce}+\psi_{ev}\cdot\psi_{cv}$ | 0.184921 | 0.82601 | 9513.4 |
| 143 | $\overline{\boldsymbol{u}} \sim \psi_{ce}+\psi_{ev}+\psi_{cv}+\psi_{ac}\cdot\psi_{ce}+\psi_{ac}\cdot\psi_{ev}+\psi_{ac}\cdot\psi_{cv}+\psi_{ce}\cdot\psi_{ev}+\psi_{ce}\cdot\psi_{cv}+\psi_{ev}\cdot\psi_{cv}$ | 0.208468 | 0.82233 | 10155.9 |
| 144 | $\overline{\boldsymbol{u}} \sim \psi_{ce}+\psi_{ev}+\psi_{cv}+\psi_{ac}\cdot\psi_{ce}+\psi_{ac}\cdot\psi_{ev}+\psi_{ac}\cdot\psi_{cv}+\psi_{ce}\cdot\psi_{cv}+\psi_{ev}\cdot\psi_{cv}$ | 0.208378 | 0.82023 | 10512.5 |
| 145 | $\overline{\boldsymbol{u}} \sim \psi_{ce}+\psi_{ac}\cdot\psi_{ce}+\psi_{ac}\cdot\psi_{cv}+\psi_{ce}\cdot\psi_{ev}$ | 1.14614 | 0.81585 | 11242.1 |
| 146 | $\overline{\boldsymbol{u}} \sim \psi_{ce}+\psi_{cv}+\psi_{ac}\cdot\psi_{ce}+\psi_{ac}\cdot\psi_{ev}$ | 1.14087 | 0.8142 | 11513.8 |
| 147 | $\overline{\boldsymbol{u}} \sim \psi_{ce}+\psi_{ev}+\psi_{cv}+\psi_{ac}\cdot\psi_{ce}+\psi_{ac}\cdot\psi_{ev}+\psi_{ac}\cdot\psi_{cv}+\psi_{ce}\cdot\psi_{ev}+\psi_{ce}\cdot\psi_{cv}$ | 0.205025 | 0.81385 | 11575.1 |
| 148 | $\overline{\boldsymbol{u}} \sim \psi_{ce}+\psi_{ev}+\psi_{cv}+\psi_{ac}\cdot\psi_{ce}+\psi_{ac}\cdot\psi_{ev}+\psi_{ac}\cdot\psi_{cv}+\psi_{ce}\cdot\psi_{cv}$ | 0.204816 | 0.81231 | 11825.1 |
| 149 | $\overline{\boldsymbol{u}} \sim \psi_{ce}+\psi_{ev}+\psi_{cv}+\psi_{ac}\cdot\psi_{ce}+\psi_{ac}\cdot\psi_{ev}+\psi_{ac}\cdot\psi_{cv}+\psi_{ce}\cdot\psi_{ev}+\psi_{ev}\cdot\psi_{cv}$ | 0.209293 | 0.81088 | 12056.9 |
| 150 | $\overline{\boldsymbol{u}} \sim \psi_{ce}+\psi_{ev}+\psi_{cv}+\psi_{ac}\cdot\psi_{ce}+\psi_{ac}\cdot\psi_{ev}+\psi_{ac}\cdot\psi_{cv}+\psi_{ev}\cdot\psi_{cv}$ | 0.208952 | 0.80898 | 12359.6 |
| 151 | $\overline{\boldsymbol{u}} \sim \psi_{ce}+\psi_{ev}+\psi_{cv}+\psi_{ac}\cdot\psi_{ce}+\psi_{ac}\cdot\psi_{ev}+\psi_{ac}\cdot\psi_{cv}+\psi_{ce}\cdot\psi_{ev}$ | 0.205411 | 0.80325 | 13260.1 |
| 152 | $\overline{\boldsymbol{u}} \sim \psi_{ce}+\psi_{ac}\cdot\psi_{ce}+\psi_{ac}\cdot\psi_{ev}$ | 1.12699 | 0.80301 | 13292.1 |
| 153 | $\overline{\boldsymbol{u}} \sim \psi_{ce}+\psi_{ev}+\psi_{cv}+\psi_{ac}\cdot\psi_{ce}+\psi_{ac}\cdot\psi_{ev}+\psi_{ac}\cdot\psi_{cv}$ | 0.20506 | 0.80185 | 13474.6 |
| 154 | $\overline{\boldsymbol{u}} \sim \psi_{ce}+\psi_{cv}+\psi_{ac}\cdot\psi_{ce}+\psi_{ce}\cdot\psi_{ev}+\psi_{ce}\cdot\psi_{cv}+\psi_{ev}\cdot\psi_{cv}$ | 1.03323 | 0.79438 | 14601.4 |
| 155 | $\overline{\boldsymbol{u}} \sim \psi_{ce}+\psi_{ac}\cdot\psi_{ce}+\psi_{ce}\cdot\psi_{ev}+\psi_{ce}\cdot\psi_{cv}+\psi_{ev}\cdot\psi_{cv}$ | 1.03158 | 0.79191 | 14963.3 |
| 156 | $\overline{\boldsymbol{u}} \sim \psi_{ac}+\psi_{ce}+\psi_{cv}+\psi_{ac}\cdot\psi_{ce}+\psi_{ac}\cdot\psi_{cv}+\psi_{ce}\cdot\psi_{cv}$ | 0.135669 | 0.78692 | 15685.6 |
| 157 | $\overline{\boldsymbol{u}} \sim \psi_{ac}+\psi_{ce}+\psi_{cv}+\psi_{ac}\cdot\psi_{ce}+\psi_{ce}\cdot\psi_{cv}$ | 0.121289 | 0.785 | 15957.7 |
| 158 | $\overline{\boldsymbol{u}} \sim \psi_{ce}+\psi_{cv}+\psi_{ac}\cdot\psi_{ce}+\psi_{ce}\cdot\psi_{ev}+\psi_{ev}\cdot\psi_{cv}$ | 1.05046 | 0.78053 | 16584.5 |

| | | | | |
|---|---|---|---|---|
| 159 | $\overline{\boldsymbol{u}} \sim \psi_{ce}+\psi_{ac}\cdot\psi_{ce}+\psi_{ce}\cdot\psi_{ev}+\psi_{ev}\cdot\psi_{cv}$ | 1.05118 | 0.7801 | 16643.3 |
| 160 | $\overline{\boldsymbol{u}} \sim \psi_{ce}+\psi_{ev}+\psi_{ac}\cdot\psi_{ce}+\psi_{ac}\cdot\psi_{ev}+\psi_{ac}\cdot\psi_{cv}+\psi_{ce}\cdot\psi_{ev}+\psi_{ce}\cdot\psi_{cv}+\psi_{ev}\cdot\psi_{cv}$ | 0.19406 | 0.77725 | 17040.1 |
| 161 | $\overline{\boldsymbol{u}} \sim \psi_{ce}+\psi_{cv}+\psi_{ac}\cdot\psi_{ce}+\psi_{ce}\cdot\psi_{ev}+\psi_{ce}\cdot\psi_{cv}$ | 1.01902 | 0.77507 | 17332.5 |
| 162 | $\overline{\boldsymbol{u}} \sim \psi_{ce}+\psi_{ac}\cdot\psi_{ce}+\psi_{ce}\cdot\psi_{ev}+\psi_{ce}\cdot\psi_{cv}$ | 1.01809 | 0.77504 | 17336.6 |
| 163 | $\overline{\boldsymbol{u}} \sim \psi_{ce}+\psi_{ev}+\psi_{ac}\cdot\psi_{ce}+\psi_{ac}\cdot\psi_{ev}+\psi_{ac}\cdot\psi_{cv}+\psi_{ce}\cdot\psi_{ev}+\psi_{ev}\cdot\psi_{cv}$ | 0.194074 | 0.77422 | 17449.6 |
| 164 | $\overline{\boldsymbol{u}} \sim \psi_{ac}+\psi_{ce}+\psi_{ac}\cdot\psi_{ce}+\psi_{ac}\cdot\psi_{cv}+\psi_{ce}\cdot\psi_{cv}$ | 0.108023 | 0.77331 | 17569.9 |
| 165 | $\overline{\boldsymbol{u}} \sim \psi_{ce}+\psi_{ev}+\psi_{ac}\cdot\psi_{ce}+\psi_{ac}\cdot\psi_{ev}+\psi_{ac}\cdot\psi_{cv}+\psi_{ce}\cdot\psi_{cv}+\psi_{ev}\cdot\psi_{cv}$ | 0.194837 | 0.77257 | 17672 |
| 166 | $\overline{\boldsymbol{u}} \sim \psi_{ce}+\psi_{ev}+\psi_{ac}\cdot\psi_{ce}+\psi_{ac}\cdot\psi_{ev}+\psi_{ac}\cdot\psi_{cv}+\psi_{ev}\cdot\psi_{cv}$ | 0.194423 | 0.7699 | 18025.4 |
| 167 | $\overline{\boldsymbol{u}} \sim \psi_{ac}+\psi_{ce}+\psi_{cv}+\psi_{ac}\cdot\psi_{ce}+\psi_{ac}\cdot\psi_{cv}$ | 0.170318 | 0.76115 | 19160.7 |
| 168 | $\overline{\boldsymbol{u}} \sim \psi_{ce}+\psi_{cv}+\psi_{ac}\cdot\psi_{ce}+\psi_{ce}\cdot\psi_{ev}$ | 1.04447 | 0.76029 | 19270.1 |
| 169 | $\overline{\boldsymbol{u}} \sim \psi_{ce}+\psi_{cv}+\psi_{ac}\cdot\psi_{ce}+\psi_{ce}\cdot\psi_{cv}+\psi_{ev}\cdot\psi_{cv}$ | 1.04526 | 0.75885 | 19453.1 |
| 170 | $\overline{\boldsymbol{u}} \sim \psi_{ac}+\psi_{ce}+\psi_{ac}\cdot\psi_{ce}+\psi_{ce}\cdot\psi_{cv}$ | 0.126292 | 0.75798 | 19562.1 |
| 171 | $\overline{\boldsymbol{u}} \sim \psi_{ac}+\psi_{ce}+\psi_{cv}+\psi_{ac}\cdot\psi_{ce}$ | 0.14291 | 0.75334 | 20139.1 |
| 172 | $\overline{\boldsymbol{u}} \sim \psi_{ac}+\psi_{ce}+\psi_{ac}\cdot\psi_{ce}$ | 0.139761 | 0.75299 | 20181.4 |
| 173 | $\overline{\boldsymbol{u}} \sim \psi_{ac}+\psi_{ce}+\psi_{ac}\cdot\psi_{ce}+\psi_{ac}\cdot\psi_{cv}$ | 0.139518 | 0.75299 | 20182.6 |
| 174 | $\overline{\boldsymbol{u}} \sim \psi_{ce}+\psi_{ac}\cdot\psi_{ce}+\psi_{ce}\cdot\psi_{cv}+\psi_{ev}\cdot\psi_{cv}$ | 1.01978 | 0.75265 | 20224.2 |
| 175 | $\overline{\boldsymbol{u}} \sim \psi_{ce}+\psi_{ac}\cdot\psi_{ce}+\psi_{ce}\cdot\psi_{ev}$ | 1.01239 | 0.74889 | 20682.5 |
| 176 | $\overline{\boldsymbol{u}} \sim \psi_{ce}+\psi_{cv}+\psi_{ac}\cdot\psi_{ce}+\psi_{ev}\cdot\psi_{cv}$ | 1.07111 | 0.74661 | 20959.7 |
| 177 | $\overline{\boldsymbol{u}} \sim \psi_{ce}+\psi_{ev}+\psi_{cv}+\psi_{ac}\cdot\psi_{ce}+\psi_{ac}\cdot\psi_{ev}+\psi_{ce}\cdot\psi_{ev}+\psi_{ce}\cdot\psi_{cv}+\psi_{ev}\cdot\psi_{cv}$ | 0.21344 | 0.737 | 22097 |
| 178 | $\overline{\boldsymbol{u}} \sim \psi_{ce}+\psi_{ev}+\psi_{ac}\cdot\psi_{ce}+\psi_{ac}\cdot\psi_{ev}+\psi_{ce}\cdot\psi_{ev}+\psi_{ce}\cdot\psi_{cv}+\psi_{ev}\cdot\psi_{cv}$ | 0.214026 | 0.73688 | 22109.7 |
| 179 | $\overline{\boldsymbol{u}} \sim \psi_{ce}+\psi_{cv}+\psi_{ac}\cdot\psi_{ce}+\psi_{ce}\cdot\psi_{cv}$ | 0.927512 | 0.73673 | 22124 |
| 180 | $\overline{\boldsymbol{u}} \sim \psi_{ce}+\psi_{ac}\cdot\psi_{ce}+\psi_{ce}\cdot\psi_{cv}$ | 0.929461 | 0.73632 | 22170.7 |
| 181 | $\overline{\boldsymbol{u}} \sim \psi_{ce}+\psi_{cv}+\psi_{ac}\cdot\psi_{ce}+\psi_{ac}\cdot\psi_{ev}+\psi_{ac}\cdot\psi_{cv}+\psi_{ce}\cdot\psi_{ev}+\psi_{ce}\cdot\psi_{cv}+\psi_{ev}\cdot\psi_{cv}$ | 0.144755 | 0.73266 | 22594.5 |
| 182 | $\overline{\boldsymbol{u}} \sim \psi_{ce}+\psi_{ev}+\psi_{cv}+\psi_{ac}\cdot\psi_{ce}+\psi_{ac}\cdot\psi_{ev}+\psi_{ce}\cdot\psi_{cv}+\psi_{ev}\cdot\psi_{cv}$ | 0.217248 | 0.73113 | 22767.6 |
| 183 | $\overline{\boldsymbol{u}} \sim \psi_{ce}+\psi_{ev}+\psi_{ac}\cdot\psi_{ce}+\psi_{ac}\cdot\psi_{ev}+\psi_{ce}\cdot\psi_{cv}+\psi_{ev}\cdot\psi_{cv}$ | 0.217645 | 0.73107 | 22773.2 |
| 184 | $\overline{\boldsymbol{u}} \sim \psi_{ce}+\psi_{cv}+\psi_{ac}\cdot\psi_{ce}+\psi_{ac}\cdot\psi_{ev}+\psi_{ac}\cdot\psi_{cv}+\psi_{ce}\cdot\psi_{ev}+\psi_{ev}\cdot\psi_{cv}$ | 0.146629 | 0.72707 | 23224 |
| 185 | $\overline{\boldsymbol{u}} \sim \psi_{ac}+\psi_{ev}+\psi_{cv}+\psi_{ac}\cdot\psi_{ce}+\psi_{ac}\cdot\psi_{ev}+\psi_{ac}\cdot\psi_{cv}+\psi_{ce}\cdot\psi_{ev}+\psi_{ce}\cdot\psi_{cv}+\psi_{ev}\cdot\psi_{cv}$ | 0.278266 | 0.72389 | 23578.2 |

| | | | | |
|---|---|---|---|---|
| 186 | $\overline{\boldsymbol{u}} \sim \psi_{ac}+\psi_{ev}+\psi_{cv}+\psi_{ac}\cdot\psi_{ce}+\psi_{ac}\cdot\psi_{ev}+\psi_{ac}\cdot\psi_{cv}+\psi_{ce}\cdot\psi_{ev}+\psi_{ce}\cdot\psi_{cv}$ | 0.279167 | 0.72373 | 23595.8 |
| 187 | $\overline{\boldsymbol{u}} \sim \psi_{ac}+\psi_{ev}+\psi_{cv}+\psi_{ac}\cdot\psi_{ce}+\psi_{ac}\cdot\psi_{cv}+\psi_{ce}\cdot\psi_{ev}+\psi_{ce}\cdot\psi_{cv}+\psi_{ev}\cdot\psi_{cv}$ | 0.281784 | 0.72314 | 23660.6 |
| 188 | $\overline{\boldsymbol{u}} \sim \psi_{ac}+\psi_{ev}+\psi_{cv}+\psi_{ac}\cdot\psi_{ce}+\psi_{ac}\cdot\psi_{cv}+\psi_{ce}\cdot\psi_{ev}+\psi_{ce}\cdot\psi_{cv}$ | 0.281871 | 0.72313 | 23660.5 |
| 189 | $\overline{\boldsymbol{u}} \sim \psi_{ce}+\psi_{ev}+\psi_{cv}+\psi_{ac}\cdot\psi_{ce}+\psi_{ac}\cdot\psi_{ev}+\psi_{ce}\cdot\psi_{ev}+\psi_{ce}\cdot\psi_{cv}$ | 0.224856 | 0.71748 | 24275.3 |
| 190 | $\overline{\boldsymbol{u}} \sim \psi_{ce}+\psi_{ev}+\psi_{cv}+\psi_{ac}\cdot\psi_{ce}+\psi_{ac}\cdot\psi_{ev}+\psi_{ce}\cdot\psi_{ev}+\psi_{ev}\cdot\psi_{cv}$ | 0.212614 | 0.71388 | 24660.7 |
| 191 | $\overline{\boldsymbol{u}} \sim \psi_{ce}+\psi_{ev}+\psi_{cv}+\psi_{ac}\cdot\psi_{ce}+\psi_{ac}\cdot\psi_{ev}+\psi_{ce}\cdot\psi_{cv}$ | 0.227594 | 0.71276 | 24778.7 |
| 192 | $\overline{\boldsymbol{u}} \sim \psi_{ce}+\psi_{ev}+\psi_{cv}+\psi_{ac}\cdot\psi_{ce}+\psi_{ac}\cdot\psi_{cv}+\psi_{ce}\cdot\psi_{cv}$ | 0.14605 | 0.71169 | 24892.1 |
| 193 | $\overline{\boldsymbol{u}} \sim \psi_{ce}+\psi_{ev}+\psi_{cv}+\psi_{ac}\cdot\psi_{ce}+\psi_{ac}\cdot\psi_{cv}+\psi_{ce}\cdot\psi_{cv}+\psi_{ev}\cdot\psi_{cv}$ | 0.14605 | 0.71169 | 24893.3 |
| 194 | $\overline{\boldsymbol{u}} \sim \psi_{ce}+\psi_{ev}+\psi_{cv}+\psi_{ac}\cdot\psi_{ce}+\psi_{ac}\cdot\psi_{cv}+\psi_{ce}\cdot\psi_{ev}+\psi_{ce}\cdot\psi_{cv}$ | 0.146043 | 0.71168 | 24894 |
| 195 | $\overline{\boldsymbol{u}} \sim \psi_{ce}+\psi_{ev}+\psi_{cv}+\psi_{ac}\cdot\psi_{ce}+\psi_{ac}\cdot\psi_{cv}+\psi_{ce}\cdot\psi_{ev}+\psi_{ce}\cdot\psi_{cv}+\psi_{ev}\cdot\psi_{cv}$ | 0.146043 | 0.71168 | 24895.2 |
| 196 | $\overline{\boldsymbol{u}} \sim \psi_{ce}+\psi_{cv}+\psi_{ac}\cdot\psi_{ce}$ | 0.949876 | 0.71157 | 24901.8 |
| 197 | $\overline{\boldsymbol{u}} \sim \psi_{ce}+\psi_{ac}\cdot\psi_{ce}$ | 0.938823 | 0.70946 | 25122.8 |
| 198 | $\overline{\boldsymbol{u}} \sim \psi_{ce}+\psi_{ev}+\psi_{cv}+\psi_{ac}\cdot\psi_{ce}+\psi_{ac}\cdot\psi_{cv}$ | 0.146603 | 0.70913 | 25160 |
| 199 | $\overline{\boldsymbol{u}} \sim \psi_{ce}+\psi_{ev}+\psi_{cv}+\psi_{ac}\cdot\psi_{ce}+\psi_{ac}\cdot\psi_{cv}+\psi_{ev}\cdot\psi_{cv}$ | 0.146604 | 0.70913 | 25161.2 |
| 200 | $\overline{\boldsymbol{u}} \sim \psi_{ce}+\psi_{ev}+\psi_{cv}+\psi_{ac}\cdot\psi_{ce}+\psi_{ac}\cdot\psi_{cv}+\psi_{ce}\cdot\psi_{ev}$ | 0.146596 | 0.70912 | 25161.9 |
| 201 | $\overline{\boldsymbol{u}} \sim \psi_{ce}+\psi_{ev}+\psi_{cv}+\psi_{ac}\cdot\psi_{ce}+\psi_{ac}\cdot\psi_{cv}+\psi_{ce}\cdot\psi_{ev}+\psi_{ev}\cdot\psi_{cv}$ | 0.146596 | 0.70912 | 25163.1 |
| 202 | $\overline{\boldsymbol{u}} \sim \psi_{ce}+\psi_{ev}+\psi_{cv}+\psi_{ac}\cdot\psi_{ce}+\psi_{ac}\cdot\psi_{ev}+\psi_{ev}\cdot\psi_{cv}$ | 0.21479 | 0.70815 | 25264.1 |
| 203 | $\overline{\boldsymbol{u}} \sim \psi_{ac}+\psi_{cv}+\psi_{ac}\cdot\psi_{ce}+\psi_{ac}\cdot\psi_{ev}+\psi_{ac}\cdot\psi_{cv}+\psi_{ce}\cdot\psi_{ev}+\psi_{ce}\cdot\psi_{cv}+\psi_{ev}\cdot\psi_{cv}$ | 0.267797 | 0.70716 | 25369.1 |
| 204 | $\overline{\boldsymbol{u}} \sim \psi_{ac}+\psi_{cv}+\psi_{ac}\cdot\psi_{ce}+\psi_{ac}\cdot\psi_{ev}+\psi_{ac}\cdot\psi_{cv}+\psi_{ce}\cdot\psi_{ev}+\psi_{ce}\cdot\psi_{cv}$ | 0.267597 | 0.70708 | 25375.8 |
| 205 | $\overline{\boldsymbol{u}} \sim \psi_{ce}+\psi_{cv}+\psi_{ac}\cdot\psi_{ce}+\psi_{ac}\cdot\psi_{ev}+\psi_{ac}\cdot\psi_{cv}+\psi_{ce}\cdot\psi_{cv}+\psi_{ev}\cdot\psi_{cv}$ | 0.142323 | 0.7028 | 25817.9 |
| 206 | $\overline{\boldsymbol{u}} \sim \psi_{ce}+\psi_{ev}+\psi_{ac}\cdot\psi_{ce}+\psi_{ac}\cdot\psi_{ev}+\psi_{ac}\cdot\psi_{cv}+\psi_{ce}\cdot\psi_{ev}+\psi_{ce}\cdot\psi_{cv}$ | 0.215446 | 0.70062 | 26040 |
| 207 | $\overline{\boldsymbol{u}} \sim \psi_{ce}+\psi_{ev}+\psi_{ac}\cdot\psi_{ce}+\psi_{ac}\cdot\psi_{ev}+\psi_{ac}\cdot\psi_{cv}+\psi_{ce}\cdot\psi_{ev}$ | 0.221396 | 0.7004 | 26061.8 |
| 208 | $\overline{\boldsymbol{u}} \sim \psi_{ce}+\psi_{ev}+\psi_{ac}\cdot\psi_{ce}+\psi_{ac}\cdot\psi_{ev}+\psi_{ce}\cdot\psi_{ev}+\psi_{ev}\cdot\psi_{cv}$ | 0.22359 | 0.70039 | 26062.7 |
| 209 | $\overline{\boldsymbol{u}} \sim \psi_{ce}+\psi_{cv}+\psi_{ac}\cdot\psi_{ce}+\psi_{ac}\cdot\psi_{cv}+\psi_{ce}\cdot\psi_{ev}+\psi_{ce}\cdot\psi_{cv}+\psi_{ev}\cdot\psi_{cv}$ | 0.142086 | 0.70002 | 26101.4 |
| 210 | $\overline{\boldsymbol{u}} \sim \psi_{ce}+\psi_{cv}+\psi_{ac}\cdot\psi_{ce}+\psi_{ac}\cdot\psi_{ev}+\psi_{ac}\cdot\psi_{cv}+\psi_{ev}\cdot\psi_{cv}$ | 0.143346 | 0.69892 | 26211.4 |
| 211 | $\overline{\boldsymbol{u}} \sim \psi_{ev}+\psi_{cv}+\psi_{ac}\cdot\psi_{ce}+\psi_{ac}\cdot\psi_{ev}+\psi_{ac}\cdot\psi_{cv}+\psi_{ce}\cdot\psi_{ev}+\psi_{ce}\cdot\psi_{cv}+\psi_{ev}\cdot\psi_{cv}$ | 0.228074 | 0.69892 | 26213.5 |
| 212 | $\overline{\boldsymbol{u}} \sim \psi_{ev}+\psi_{cv}+\psi_{ac}\cdot\psi_{ce}+\psi_{ac}\cdot\psi_{ev}+\psi_{ac}\cdot\psi_{cv}+\psi_{ce}\cdot\psi_{ev}+\psi_{ce}\cdot\psi_{cv}$ | 0.225452 | 0.69794 | 26311.4 |

| | | | | |
|---|---|---|---|---|
| 213 | $\overline{\boldsymbol{u}} \sim \psi_{ce}+\psi_{ev}+\psi_{ac}\cdot\psi_{ce}+\psi_{ac}\cdot\psi_{ev}+\psi_{ce}\cdot\psi_{ev}+\psi_{ce}\cdot\psi_{cv}$ | 0.231268 | 0.69791 | 26313.5 |
| 214 | $\overline{\boldsymbol{u}} \sim \psi_{ce}+\psi_{cv}+\psi_{ac}\cdot\psi_{ce}+\psi_{ac}\cdot\psi_{cv}+\psi_{ce}\cdot\psi_{ev}+\psi_{ev}\cdot\psi_{cv}$ | 0.142664 | 0.69745 | 26359.4 |
| 215 | $\overline{\boldsymbol{u}} \sim \psi_{ce}+\psi_{ev}+\psi_{ac}\cdot\psi_{ce}+\psi_{ac}\cdot\psi_{ev}+\psi_{ac}\cdot\psi_{cv}+\psi_{ce}\cdot\psi_{cv}$ | 0.21592 | 0.69638 | 26466.9 |
| 216 | $\overline{\boldsymbol{u}} \sim \psi_{ce}+\psi_{ev}+\psi_{ac}\cdot\psi_{ce}+\psi_{ac}\cdot\psi_{ev}+\psi_{ac}\cdot\psi_{cv}$ | 0.223658 | 0.69606 | 26498.3 |
| 217 | $\overline{\boldsymbol{u}} \sim \psi_{ce}+\psi_{ev}+\psi_{ac}\cdot\psi_{ce}+\psi_{ac}\cdot\psi_{ev}+\psi_{ev}\cdot\psi_{cv}$ | 0.224469 | 0.69551 | 26553.6 |
| 218 | $\overline{\boldsymbol{u}} \sim \psi_{ce}+\psi_{ev}+\psi_{cv}+\psi_{ac}\cdot\psi_{ce}+\psi_{ac}\cdot\psi_{ev}+\psi_{ce}\cdot\psi_{ev}$ | 0.219592 | 0.69527 | 26578.5 |
| 219 | $\overline{\boldsymbol{u}} \sim \psi_{ce}+\psi_{ev}+\psi_{ac}\cdot\psi_{ce}+\psi_{ac}\cdot\psi_{ev}+\psi_{ce}\cdot\psi_{ev}$ | 0.21965 | 0.6948 | 26624.3 |
| 220 | $\overline{\boldsymbol{u}} \sim \psi_{ce}+\psi_{ev}+\psi_{ac}\cdot\psi_{ce}+\psi_{ac}\cdot\psi_{ev}+\psi_{ce}\cdot\psi_{cv}$ | 0.234415 | 0.69329 | 26774.6 |
| 221 | $\overline{\boldsymbol{u}} \sim \psi_{ce}+\psi_{cv}+\psi_{ac}\cdot\psi_{ce}+\psi_{ac}\cdot\psi_{cv}+\psi_{ce}\cdot\psi_{cv}+\psi_{ev}\cdot\psi_{cv}$ | 0.14134 | 0.69227 | 26877.1 |
| 222 | $\overline{\boldsymbol{u}} \sim \psi_{ce}+\psi_{ev}+\psi_{cv}+\psi_{ac}\cdot\psi_{ce}+\psi_{ac}\cdot\psi_{ev}$ | 0.220817 | 0.69063 | 27037.5 |
| 223 | $\overline{\boldsymbol{u}} \sim \psi_{ce}+\psi_{ev}+\psi_{ac}\cdot\psi_{ce}+\psi_{ac}\cdot\psi_{ev}$ | 0.22106 | 0.69017 | 27082 |
| 224 | $\overline{\boldsymbol{u}} \sim \psi_{ce}+\psi_{cv}+\psi_{ac}\cdot\psi_{ce}+\psi_{ac}\cdot\psi_{cv}+\psi_{ev}\cdot\psi_{cv}$ | 0.141901 | 0.68969 | 27129.8 |
| 225 | $\overline{\boldsymbol{u}} \sim \psi_{ce}+\psi_{cv}+\psi_{ac}\cdot\psi_{ce}+\psi_{ac}\cdot\psi_{ev}+\psi_{ac}\cdot\psi_{cv}+\psi_{ce}\cdot\psi_{ev}+\psi_{ce}\cdot\psi_{cv}$ | 0.136955 | 0.68602 | 27489.5 |
| 226 | $\overline{\boldsymbol{u}} \sim \psi_{ce}+\psi_{cv}+\psi_{ac}\cdot\psi_{ce}+\psi_{ac}\cdot\psi_{cv}+\psi_{ce}\cdot\psi_{ev}+\psi_{ce}\cdot\psi_{cv}$ | 0.137573 | 0.6847 | 27616.5 |
| 227 | $\overline{\boldsymbol{u}} \sim \psi_{ce}+\psi_{ac}\cdot\psi_{ce}+\psi_{ac}\cdot\psi_{ev}+\psi_{ac}\cdot\psi_{cv}+\psi_{ce}\cdot\psi_{ev}+\psi_{ce}\cdot\psi_{cv}+\psi_{ev}\cdot\psi_{cv}$ | 0.125418 | 0.68322 | 27760.4 |
| 228 | $\overline{\boldsymbol{u}} \sim \psi_{ce}+\psi_{cv}+\psi_{ac}\cdot\psi_{ce}+\psi_{ac}\cdot\psi_{ev}+\psi_{ac}\cdot\psi_{cv}+\psi_{ce}\cdot\psi_{ev}$ | 0.137781 | 0.68313 | 27767.5 |
| 229 | $\overline{\boldsymbol{u}} \sim \psi_{ce}+\psi_{ac}\cdot\psi_{ce}+\psi_{ac}\cdot\psi_{ev}+\psi_{ac}\cdot\psi_{cv}+\psi_{ce}\cdot\psi_{ev}+\psi_{ev}\cdot\psi_{cv}$ | 0.126376 | 0.68292 | 27787.6 |
| 230 | $\overline{\boldsymbol{u}} \sim \psi_{ac}+\psi_{ev}+\psi_{cv}+\psi_{ac}\cdot\psi_{ce}+\psi_{ac}\cdot\psi_{ev}+\psi_{ac}\cdot\psi_{cv}+\psi_{ce}\cdot\psi_{ev}+\psi_{ev}\cdot\psi_{cv}$ | 0.326689 | 0.68234 | 27845.2 |
| 231 | $\overline{\boldsymbol{u}} \sim \psi_{ce}+\psi_{cv}+\psi_{ac}\cdot\psi_{ce}+\psi_{ac}\cdot\psi_{cv}+\psi_{ce}\cdot\psi_{ev}$ | 0.138175 | 0.68215 | 27860.9 |
| 232 | $\overline{\boldsymbol{u}} \sim \psi_{ac}+\psi_{ev}+\psi_{cv}+\psi_{ac}\cdot\psi_{ce}+\psi_{ac}\cdot\psi_{ev}+\psi_{ac}\cdot\psi_{cv}+\psi_{ce}\cdot\psi_{ev}$ | 0.327851 | 0.68212 | 27865.7 |
| 233 | $\overline{\boldsymbol{u}} \sim \psi_{ac}+\psi_{ev}+\psi_{cv}+\psi_{ac}\cdot\psi_{ce}+\psi_{ac}\cdot\psi_{cv}+\psi_{ce}\cdot\psi_{ev}+\psi_{ev}\cdot\psi_{cv}$ | 0.331215 | 0.68128 | 27945.9 |
| 234 | $\overline{\boldsymbol{u}} \sim \psi_{ac}+\psi_{ev}+\psi_{cv}+\psi_{ac}\cdot\psi_{ce}+\psi_{ac}\cdot\psi_{cv}+\psi_{ce}\cdot\psi_{ev}$ | 0.331326 | 0.68127 | 27945.8 |
| 235 | $\overline{\boldsymbol{u}} \sim \psi_{ac}+\psi_{ev}+\psi_{cv}+\psi_{ac}\cdot\psi_{ce}+\psi_{ac}\cdot\psi_{ev}+\psi_{ac}\cdot\psi_{cv}+\psi_{ce}\cdot\psi_{cv}+\psi_{ev}\cdot\psi_{cv}$ | 0.334263 | 0.6808 | 27992.5 |
| 236 | $\overline{\boldsymbol{u}} \sim \psi_{ac}+\psi_{ev}+\psi_{cv}+\psi_{ac}\cdot\psi_{ce}+\psi_{ac}\cdot\psi_{ev}+\psi_{ac}\cdot\psi_{cv}+\psi_{ce}\cdot\psi_{cv}$ | 0.334134 | 0.68079 | 27992.8 |
| 237 | $\overline{\boldsymbol{u}} \sim \psi_{ac}+\psi_{ev}+\psi_{cv}+\psi_{ac}\cdot\psi_{ce}+\psi_{ac}\cdot\psi_{cv}+\psi_{ce}\cdot\psi_{cv}$ | 0.333755 | 0.68077 | 27993.6 |
| 238 | $\overline{\boldsymbol{u}} \sim \psi_{ac}+\psi_{ev}+\psi_{cv}+\psi_{ac}\cdot\psi_{ce}+\psi_{ac}\cdot\psi_{cv}+\psi_{ce}\cdot\psi_{cv}+\psi_{ev}\cdot\psi_{cv}$ | 0.333764 | 0.68077 | 27994.9 |
| 239 | $\overline{\boldsymbol{u}} \sim \psi_{ac}+\psi_{ce}+\psi_{ev}+\psi_{cv}+\psi_{ac}\cdot\psi_{ev}+\psi_{ac}\cdot\psi_{cv}+\psi_{ev}\cdot\psi_{cv}$ | 0.964741 | 0.6796 | 28106 |

| | | | | |
|---|---|---|---|---|
| 240 | $\overline{\boldsymbol{u}} \sim \psi_{ac}+\psi_{ce}+\psi_{ev}+\psi_{cv}+\psi_{ac}\cdot\psi_{ev}+\psi_{ac}\cdot\psi_{cv}+\psi_{ce}\cdot\psi_{ev}+\psi_{ev}\cdot\psi_{cv}$ | 0.964695 | 0.67959 | 28108 |
| 241 | $\overline{\boldsymbol{u}} \sim \psi_{ac}+\psi_{ce}+\psi_{ev}+\psi_{cv}+\psi_{ac}\cdot\psi_{ev}+\psi_{ac}\cdot\psi_{cv}+\psi_{ce}\cdot\psi_{cv}+\psi_{ev}\cdot\psi_{cv}$ | 0.96431 | 0.67959 | 28108 |
| 242 | $\overline{\boldsymbol{u}} \sim \psi_{ac}+\psi_{ce}+\psi_{ev}+\psi_{cv}+\psi_{ac}\cdot\psi_{ev}+\psi_{ac}\cdot\psi_{cv}$ | 0.962403 | 0.67958 | 28106.8 |
| 243 | $\overline{\boldsymbol{u}} \sim \psi_{ac}+\psi_{ce}+\psi_{ev}+\psi_{cv}+\psi_{ac}\cdot\psi_{ev}+\psi_{ac}\cdot\psi_{cv}+\psi_{ce}\cdot\psi_{ev}+\psi_{ce}\cdot\psi_{cv}+\psi_{ev}\cdot\psi_{cv}$ | 0.964264 | 0.67958 | 28110 |
| 244 | $\overline{\boldsymbol{u}} \sim \psi_{ac}+\psi_{ce}+\psi_{ev}+\psi_{cv}+\psi_{ac}\cdot\psi_{ev}+\psi_{ac}\cdot\psi_{cv}+\psi_{ce}\cdot\psi_{ev}$ | 0.962537 | 0.67957 | 28108.8 |
| 245 | $\overline{\boldsymbol{u}} \sim \psi_{ac}+\psi_{ce}+\psi_{ev}+\psi_{cv}+\psi_{ac}\cdot\psi_{ev}+\psi_{ac}\cdot\psi_{cv}+\psi_{ce}\cdot\psi_{cv}$ | 0.961937 | 0.67957 | 28108.8 |
| 246 | $\overline{\boldsymbol{u}} \sim \psi_{ac}+\psi_{ce}+\psi_{ev}+\psi_{cv}+\psi_{ac}\cdot\psi_{ev}+\psi_{ac}\cdot\psi_{cv}+\psi_{ce}\cdot\psi_{ev}+\psi_{ce}\cdot\psi_{cv}$ | 0.962073 | 0.67956 | 28110.8 |
| 247 | $\overline{\boldsymbol{u}} \sim \psi_{ac}+\psi_{ce}+\psi_{ev}+\psi_{cv}+\psi_{ac}\cdot\psi_{cv}$ | 0.954047 | 0.67954 | 28110.1 |
| 248 | $\overline{\boldsymbol{u}} \sim \psi_{ac}+\psi_{ce}+\psi_{ev}+\psi_{cv}+\psi_{ac}\cdot\psi_{cv}+\psi_{ce}\cdot\psi_{ev}$ | 0.954941 | 0.67953 | 28111.8 |
| 249 | $\overline{\boldsymbol{u}} \sim \psi_{ac}+\psi_{ce}+\psi_{ev}+\psi_{cv}+\psi_{ac}\cdot\psi_{cv}+\psi_{ce}\cdot\psi_{cv}$ | 0.953473 | 0.67953 | 28112.1 |
| 250 | $\overline{\boldsymbol{u}} \sim \psi_{ac}+\psi_{ce}+\psi_{ev}+\psi_{cv}+\psi_{ac}\cdot\psi_{cv}+\psi_{ev}\cdot\psi_{cv}$ | 0.954219 | 0.67953 | 28111.4 |
| 251 | $\overline{\boldsymbol{u}} \sim \psi_{ac}+\psi_{ce}+\psi_{ev}+\psi_{cv}+\psi_{ac}\cdot\psi_{cv}+\psi_{ce}\cdot\psi_{ev}+\psi_{ev}\cdot\psi_{cv}$ | 0.955066 | 0.67953 | 28113.1 |
| 252 | $\overline{\boldsymbol{u}} \sim \psi_{ac}+\psi_{ce}+\psi_{ev}+\psi_{cv}+\psi_{ac}\cdot\psi_{cv}+\psi_{ce}\cdot\psi_{ev}+\psi_{ce}\cdot\psi_{cv}$ | 0.954379 | 0.67952 | 28113.8 |
| 253 | $\overline{\boldsymbol{u}} \sim \psi_{ac}+\psi_{ce}+\psi_{ev}+\psi_{cv}+\psi_{ac}\cdot\psi_{cv}+\psi_{ce}\cdot\psi_{cv}+\psi_{ev}\cdot\psi_{cv}$ | 0.95365 | 0.67952 | 28113.4 |
| 254 | $\overline{\boldsymbol{u}} \sim \psi_{ac}+\psi_{ce}+\psi_{ev}+\psi_{cv}+\psi_{ac}\cdot\psi_{cv}+\psi_{ce}\cdot\psi_{ev}+\psi_{ce}\cdot\psi_{cv}+\psi_{ev}\cdot\psi_{cv}$ | 0.954507 | 0.67952 | 28115.1 |
| 255 | $\overline{\boldsymbol{u}} \sim \psi_{ev}+\psi_{cv}+\psi_{ac}\cdot\psi_{ce}+\psi_{ac}\cdot\psi_{cv}+\psi_{ce}\cdot\psi_{ev}+\psi_{ce}\cdot\psi_{cv}$ | 0.180434 | 0.67401 | 28631.7 |
| 256 | $\overline{\boldsymbol{u}} \sim \psi_{ev}+\psi_{cv}+\psi_{ac}\cdot\psi_{ce}+\psi_{ac}\cdot\psi_{cv}+\psi_{ce}\cdot\psi_{ev}+\psi_{ce}\cdot\psi_{cv}+\psi_{ev}\cdot\psi_{cv}$ | 0.180421 | 0.674 | 28633.4 |
| 257 | $\overline{\boldsymbol{u}} \sim \psi_{ac}+\psi_{cv}+\psi_{ac}\cdot\psi_{ce}+\psi_{ac}\cdot\psi_{ev}+\psi_{ac}\cdot\psi_{cv}+\psi_{ce}\cdot\psi_{cv}+\psi_{ev}\cdot\psi_{cv}$ | 0.30704 | 0.67327 | 28701.9 |
| 258 | $\overline{\boldsymbol{u}} \sim \psi_{ac}+\psi_{cv}+\psi_{ac}\cdot\psi_{ce}+\psi_{ac}\cdot\psi_{ev}+\psi_{ac}\cdot\psi_{cv}+\psi_{ce}\cdot\psi_{cv}$ | 0.306533 | 0.67295 | 28730.8 |
| 259 | $\overline{\boldsymbol{u}} \sim \psi_{ev}+\psi_{cv}+\psi_{ac}\cdot\psi_{ce}+\psi_{ac}\cdot\psi_{ev}+\psi_{ac}\cdot\psi_{cv}+\psi_{ce}\cdot\psi_{cv}+\psi_{ev}\cdot\psi_{cv}$ | 0.26361 | 0.67184 | 28834.3 |
| 260 | $\overline{\boldsymbol{u}} \sim \psi_{ev}+\psi_{cv}+\psi_{ac}\cdot\psi_{ce}+\psi_{ac}\cdot\psi_{ev}+\psi_{ac}\cdot\psi_{cv}+\psi_{ce}\cdot\psi_{cv}$ | 0.259758 | 0.67099 | 28912.1 |
| 261 | $\overline{\boldsymbol{u}} \sim \psi_{ac}+\psi_{ev}+\psi_{cv}+\psi_{ac}\cdot\psi_{ev}+\psi_{ac}\cdot\psi_{cv}+\psi_{ce}\cdot\psi_{ev}+\psi_{ce}\cdot\psi_{cv}$ | 0.46673 | 0.66816 | 29174.5 |
| 262 | $\overline{\boldsymbol{u}} \sim \psi_{ac}+\psi_{ev}+\psi_{cv}+\psi_{ac}\cdot\psi_{ev}+\psi_{ac}\cdot\psi_{cv}+\psi_{ce}\cdot\psi_{ev}+\psi_{ce}\cdot\psi_{cv}+\psi_{ev}\cdot\psi_{cv}$ | 0.466749 | 0.66815 | 29176.1 |
| 263 | $\overline{\boldsymbol{u}} \sim \psi_{ac}+\psi_{ev}+\psi_{cv}+\psi_{ac}\cdot\psi_{cv}+\psi_{ce}\cdot\psi_{ev}+\psi_{ce}\cdot\psi_{cv}$ | 0.466716 | 0.66811 | 29177.5 |
| 264 | $\overline{\boldsymbol{u}} \sim \psi_{ac}+\psi_{ev}+\psi_{cv}+\psi_{ac}\cdot\psi_{cv}+\psi_{ce}\cdot\psi_{ev}+\psi_{ce}\cdot\psi_{cv}+\psi_{ev}\cdot\psi_{cv}$ | 0.466712 | 0.6681 | 29179.5 |
| 265 | $\overline{\boldsymbol{u}} \sim \psi_{ev}+\psi_{cv}+\psi_{ac}\cdot\psi_{ce}+\psi_{ac}\cdot\psi_{ev}+\psi_{ac}\cdot\psi_{cv}+\psi_{ce}\cdot\psi_{ev}+\psi_{ev}\cdot\psi_{cv}$ | 0.243806 | 0.66707 | 29274.5 |
| 266 | $\overline{\boldsymbol{u}} \sim \psi_{ac}+\psi_{ce}+\psi_{cv}+\psi_{ac}\cdot\psi_{ev}+\psi_{ac}\cdot\psi_{cv}+\psi_{ce}\cdot\psi_{ev}+\psi_{ce}\cdot\psi_{cv}+\psi_{ev}\cdot\psi_{cv}$ | 0.436917 | 0.66691 | 29290.1 |

| | | | | |
|---|---|---|---|---|
| 267 | $\overline{\boldsymbol{u}} \sim \psi_{ac}+\psi_{ce}+\psi_{cv}+\psi_{ac}\cdot\psi_{ev}+\psi_{ac}\cdot\psi_{cv}+\psi_{ce}\cdot\psi_{ev}+\psi_{ev}\cdot\psi_{cv}$ | 0.438685 | 0.66684 | 29295.1 |
| 268 | $\overline{\boldsymbol{u}} \sim \psi_{ev}+\psi_{cv}+\psi_{ac}\cdot\psi_{ce}+\psi_{ac}\cdot\psi_{ev}+\psi_{ac}\cdot\psi_{cv}+\psi_{ce}\cdot\psi_{ev}$ | 0.24141 | 0.66675 | 29302.7 |
| 269 | $\overline{\boldsymbol{u}} \sim \psi_{ac}+\psi_{ce}+\psi_{cv}+\psi_{ac}\cdot\psi_{ev}+\psi_{ac}\cdot\psi_{cv}+\psi_{ce}\cdot\psi_{ev}+\psi_{ce}\cdot\psi_{cv}$ | 0.435956 | 0.66658 | 29319.3 |
| 270 | $\overline{\boldsymbol{u}} \sim \psi_{ac}+\psi_{ce}+\psi_{cv}+\psi_{ac}\cdot\psi_{ev}+\psi_{ac}\cdot\psi_{cv}+\psi_{ce}\cdot\psi_{ev}$ | 0.437734 | 0.66651 | 29324.3 |
| 271 | $\overline{\boldsymbol{u}} \sim \psi_{ac}+\psi_{ce}+\psi_{cv}+\psi_{ac}\cdot\psi_{ev}+\psi_{ac}\cdot\psi_{cv}+\psi_{ce}\cdot\psi_{cv}+\psi_{ev}\cdot\psi_{cv}$ | 0.437058 | 0.6665 | 29326.2 |
| 272 | $\overline{\boldsymbol{u}} \sim \psi_{ac}+\psi_{ce}+\psi_{cv}+\psi_{ac}\cdot\psi_{ev}+\psi_{ac}\cdot\psi_{cv}+\psi_{ev}\cdot\psi_{cv}$ | 0.438829 | 0.66643 | 29331.2 |
| 273 | $\overline{\boldsymbol{u}} \sim \psi_{ac}+\psi_{ce}+\psi_{cv}+\psi_{ac}\cdot\psi_{ev}+\psi_{ac}\cdot\psi_{cv}+\psi_{ce}\cdot\psi_{cv}$ | 0.436278 | 0.66622 | 29350.8 |
| 274 | $\overline{\boldsymbol{u}} \sim \psi_{ac}+\psi_{ce}+\psi_{cv}+\psi_{ac}\cdot\psi_{ev}+\psi_{ac}\cdot\psi_{cv}$ | 0.438059 | 0.66615 | 29355.8 |
| 275 | $\overline{\boldsymbol{u}} \sim \psi_{ce}+\psi_{ev}+\psi_{cv}+\psi_{ac}\cdot\psi_{ev}+\psi_{ac}\cdot\psi_{cv}+\psi_{ce}\cdot\psi_{ev}+\psi_{ce}\cdot\psi_{cv}+\psi_{ev}\cdot\psi_{cv}$ | 0.315032 | 0.66599 | 29373.5 |
| 276 | $\overline{\boldsymbol{u}} \sim \psi_{ce}+\psi_{ev}+\psi_{cv}+\psi_{ac}\cdot\psi_{ev}+\psi_{ac}\cdot\psi_{cv}+\psi_{ce}\cdot\psi_{ev}+\psi_{ev}\cdot\psi_{cv}$ | 0.316459 | 0.66591 | 29380.4 |
| 277 | $\overline{\boldsymbol{u}} \sim \psi_{ce}+\psi_{ev}+\psi_{cv}+\psi_{ac}\cdot\psi_{ev}+\psi_{ac}\cdot\psi_{cv}+\psi_{ce}\cdot\psi_{cv}+\psi_{ev}\cdot\psi_{cv}$ | 0.313036 | 0.66579 | 29391 |
| 278 | $\overline{\boldsymbol{u}} \sim \psi_{ac}+\psi_{cv}+\psi_{ac}\cdot\psi_{ce}+\psi_{ac}\cdot\psi_{ev}+\psi_{ac}\cdot\psi_{cv}+\psi_{ce}\cdot\psi_{ev}+\psi_{ev}\cdot\psi_{cv}$ | 0.309363 | 0.66575 | 29394.1 |
| 279 | $\overline{\boldsymbol{u}} \sim \psi_{ac}+\psi_{cv}+\psi_{ac}\cdot\psi_{ce}+\psi_{ac}\cdot\psi_{ev}+\psi_{ac}\cdot\psi_{cv}+\psi_{ce}\cdot\psi_{ev}$ | 0.309245 | 0.66573 | 29395.7 |
| 280 | $\overline{\boldsymbol{u}} \sim \psi_{ce}+\psi_{ev}+\psi_{cv}+\psi_{ac}\cdot\psi_{ev}+\psi_{ac}\cdot\psi_{cv}+\psi_{ev}\cdot\psi_{cv}$ | 0.314458 | 0.6657 | 29398 |
| 281 | $\overline{\boldsymbol{u}} \sim \psi_{ce}+\psi_{ev}+\psi_{cv}+\psi_{ac}\cdot\psi_{ev}+\psi_{ac}\cdot\psi_{cv}+\psi_{ce}\cdot\psi_{ev}+\psi_{ce}\cdot\psi_{cv}$ | 0.308679 | 0.66538 | 29428.5 |
| 282 | $\overline{\boldsymbol{u}} \sim \psi_{ce}+\psi_{ev}+\psi_{cv}+\psi_{ac}\cdot\psi_{ev}+\psi_{ac}\cdot\psi_{cv}+\psi_{ce}\cdot\psi_{ev}$ | 0.310097 | 0.66529 | 29435.5 |
| 283 | $\overline{\boldsymbol{u}} \sim \psi_{ce}+\psi_{ev}+\psi_{cv}+\psi_{ac}\cdot\psi_{ev}+\psi_{ac}\cdot\psi_{cv}+\psi_{ce}\cdot\psi_{cv}$ | 0.307192 | 0.66522 | 29441.8 |
| 284 | $\overline{\boldsymbol{u}} \sim \psi_{ce}+\psi_{ev}+\psi_{cv}+\psi_{ac}\cdot\psi_{ev}+\psi_{ac}\cdot\psi_{cv}$ | 0.308606 | 0.66513 | 29448.8 |
| 285 | $\overline{\boldsymbol{u}} \sim \psi_{ac}+\psi_{ev}+\psi_{cv}+\psi_{ac}\cdot\psi_{ev}+\psi_{ac}\cdot\psi_{cv}+\psi_{ce}\cdot\psi_{ev}+\psi_{ev}\cdot\psi_{cv}$ | 0.428068 | 0.66443 | 29514.1 |
| 286 | $\overline{\boldsymbol{u}} \sim \psi_{ac}+\psi_{ev}+\psi_{cv}+\psi_{ac}\cdot\psi_{ev}+\psi_{ac}\cdot\psi_{cv}+\psi_{ce}\cdot\psi_{ev}$ | 0.427991 | 0.66442 | 29514.8 |
| 287 | $\overline{\boldsymbol{u}} \sim \psi_{ac}+\psi_{ev}+\psi_{cv}+\psi_{ac}\cdot\psi_{cv}+\psi_{ce}\cdot\psi_{ev}$ | 0.427772 | 0.66425 | 29528.8 |
| 288 | $\overline{\boldsymbol{u}} \sim \psi_{ac}+\psi_{ev}+\psi_{cv}+\psi_{ac}\cdot\psi_{cv}+\psi_{ce}\cdot\psi_{ev}+\psi_{ev}\cdot\psi_{cv}$ | 0.42778 | 0.66424 | 29530.7 |
| 289 | $\overline{\boldsymbol{u}} \sim \psi_{ac}+\psi_{cv}+\psi_{ac}\cdot\psi_{ce}+\psi_{ac}\cdot\psi_{cv}+\psi_{ce}\cdot\psi_{ev}+\psi_{ce}\cdot\psi_{cv}+\psi_{ev}\cdot\psi_{cv}$ | 0.250828 | 0.66363 | 29587.2 |
| 290 | $\overline{\boldsymbol{u}} \sim \psi_{ac}+\psi_{ev}+\psi_{cv}+\psi_{ac}\cdot\psi_{cv}+\psi_{ce}\cdot\psi_{cv}$ | 0.428747 | 0.66168 | 29761.3 |
| 291 | $\overline{\boldsymbol{u}} \sim \psi_{ac}+\psi_{ev}+\psi_{cv}+\psi_{ac}\cdot\psi_{ev}+\psi_{ac}\cdot\psi_{cv}+\psi_{ce}\cdot\psi_{cv}+\psi_{ev}\cdot\psi_{cv}$ | 0.429076 | 0.66168 | 29763.4 |
| 292 | $\overline{\boldsymbol{u}} \sim \psi_{ac}+\psi_{ev}+\psi_{cv}+\psi_{ac}\cdot\psi_{ev}+\psi_{ac}\cdot\psi_{cv}+\psi_{ce}\cdot\psi_{cv}$ | 0.428963 | 0.66167 | 29762.6 |
| 293 | $\overline{\boldsymbol{u}} \sim \psi_{ac}+\psi_{ev}+\psi_{cv}+\psi_{ac}\cdot\psi_{cv}+\psi_{ce}\cdot\psi_{cv}+\psi_{ev}\cdot\psi_{cv}$ | 0.428758 | 0.66167 | 29762.7 |

| | | | | |
|---|---|---|---|---|
| 294 | $\overline{\boldsymbol{u}} \sim \psi_{ev}+\psi_{cv}+\psi_{ac}\cdot\psi_{ev}+\psi_{ac}\cdot\psi_{cv}+\psi_{ce}\cdot\psi_{ev}+\psi_{ce}\cdot\psi_{cv}+\psi_{ev}\cdot\psi_{cv}$ | 0.303081 | 0.65869 | 30030.8 |
| 295 | $\overline{\boldsymbol{u}} \sim \psi_{ev}+\psi_{cv}+\psi_{ac}\cdot\psi_{ev}+\psi_{ac}\cdot\psi_{cv}+\psi_{ce}\cdot\psi_{ev}+\psi_{ce}\cdot\psi_{cv}$ | 0.298746 | 0.65832 | 30063.2 |
| 296 | $\overline{\boldsymbol{u}} \sim \psi_{ac}+\psi_{cv}+\psi_{ac}\cdot\psi_{ev}+\psi_{ac}\cdot\psi_{cv}+\psi_{ce}\cdot\psi_{ev}+\psi_{ce}\cdot\psi_{cv}+\psi_{ev}\cdot\psi_{cv}$ | 0.390348 | 0.65717 | 30166.4 |
| 297 | $\overline{\boldsymbol{u}} \sim \psi_{ac}+\psi_{cv}+\psi_{ac}\cdot\psi_{ev}+\psi_{ac}\cdot\psi_{cv}+\psi_{ce}\cdot\psi_{ev}+\psi_{ce}\cdot\psi_{cv}$ | 0.390164 | 0.657 | 30180.5 |
| 298 | $\overline{\boldsymbol{u}} \sim \psi_{ac}+\psi_{cv}+\psi_{ac}\cdot\psi_{ce}+\psi_{ac}\cdot\psi_{cv}+\psi_{ce}\cdot\psi_{cv}+\psi_{ev}\cdot\psi_{cv}$ | 0.268807 | 0.65683 | 30195.2 |
| 299 | $\overline{\boldsymbol{u}} \sim \psi_{ce}+\psi_{ev}+\psi_{cv}+\psi_{ac}\cdot\psi_{cv}+\psi_{ce}\cdot\psi_{cv}$ | 0.232476 | 0.65669 | 30207.1 |
| 300 | $\overline{\boldsymbol{u}} \sim \psi_{ce}+\psi_{ev}+\psi_{cv}+\psi_{ac}\cdot\psi_{cv}+\psi_{ce}\cdot\psi_{ev}+\psi_{ce}\cdot\psi_{cv}$ | 0.232458 | 0.65668 | 30208.6 |
| 301 | $\overline{\boldsymbol{u}} \sim \psi_{ce}+\psi_{ev}+\psi_{cv}+\psi_{ac}\cdot\psi_{cv}+\psi_{ce}\cdot\psi_{cv}+\psi_{ev}\cdot\psi_{cv}$ | 0.232477 | 0.65668 | 30208.6 |
| 302 | $\overline{\boldsymbol{u}} \sim \psi_{ce}+\psi_{ev}+\psi_{cv}+\psi_{ac}\cdot\psi_{cv}+\psi_{ce}\cdot\psi_{ev}+\psi_{ce}\cdot\psi_{cv}+\psi_{ev}\cdot\psi_{cv}$ | 0.232459 | 0.65668 | 30210 |
| 303 | $\overline{\boldsymbol{u}} \sim \psi_{ce}+\psi_{ev}+\psi_{cv}+\psi_{ac}\cdot\psi_{cv}$ | 0.233618 | 0.65662 | 30212.1 |
| 304 | $\overline{\boldsymbol{u}} \sim \psi_{ce}+\psi_{ev}+\psi_{cv}+\psi_{ac}\cdot\psi_{cv}+\psi_{ce}\cdot\psi_{ev}$ | 0.2336 | 0.65661 | 30213.6 |
| 305 | $\overline{\boldsymbol{u}} \sim \psi_{ce}+\psi_{ev}+\psi_{cv}+\psi_{ac}\cdot\psi_{cv}+\psi_{ev}\cdot\psi_{cv}$ | 0.233619 | 0.65661 | 30213.6 |
| 306 | $\overline{\boldsymbol{u}} \sim \psi_{ce}+\psi_{ev}+\psi_{cv}+\psi_{ac}\cdot\psi_{cv}+\psi_{ce}\cdot\psi_{ev}+\psi_{ev}\cdot\psi_{cv}$ | 0.233601 | 0.65661 | 30215 |
| 307 | $\overline{\boldsymbol{u}} \sim \psi_{ev}+\psi_{cv}+\psi_{ac}\cdot\psi_{ce}+\psi_{ac}\cdot\psi_{cv}+\psi_{ce}\cdot\psi_{cv}$ | 0.204913 | 0.65641 | 30232.1 |
| 308 | $\overline{\boldsymbol{u}} \sim \psi_{ev}+\psi_{cv}+\psi_{ac}\cdot\psi_{ce}+\psi_{ac}\cdot\psi_{cv}+\psi_{ce}\cdot\psi_{cv}+\psi_{ev}\cdot\psi_{cv}$ | 0.204913 | 0.6564 | 30233.5 |
| 309 | $\overline{\boldsymbol{u}} \sim \psi_{ev}+\psi_{cv}+\psi_{ac}\cdot\psi_{ce}+\psi_{ac}\cdot\psi_{cv}+\psi_{ce}\cdot\psi_{ev}$ | 0.197111 | 0.65631 | 30240.7 |
| 310 | $\overline{\boldsymbol{u}} \sim \psi_{ev}+\psi_{cv}+\psi_{ac}\cdot\psi_{ce}+\psi_{ac}\cdot\psi_{cv}+\psi_{ce}\cdot\psi_{ev}+\psi_{ev}\cdot\psi_{cv}$ | 0.197079 | 0.6563 | 30242 |
| 311 | $\overline{\boldsymbol{u}} \sim \psi_{ac}+\psi_{ce}+\psi_{cv}+\psi_{ac}\cdot\psi_{cv}+\psi_{ce}\cdot\psi_{ev}+\psi_{ce}\cdot\psi_{cv}+\psi_{ev}\cdot\psi_{cv}$ | 0.353293 | 0.65602 | 30268.5 |
| 312 | $\overline{\boldsymbol{u}} \sim \psi_{ac}+\psi_{ce}+\psi_{cv}+\psi_{ac}\cdot\psi_{cv}+\psi_{ce}\cdot\psi_{ev}+\psi_{ev}\cdot\psi_{cv}$ | 0.355026 | 0.65589 | 30278.7 |
| 313 | $\overline{\boldsymbol{u}} \sim \psi_{ev}+\psi_{cv}+\psi_{ac}\cdot\psi_{ev}+\psi_{ac}\cdot\psi_{cv}+\psi_{ce}\cdot\psi_{ev}+\psi_{ev}\cdot\psi_{cv}$ | 0.288272 | 0.65525 | 30335.3 |
| 314 | $\overline{\boldsymbol{u}} \sim \psi_{ev}+\psi_{cv}+\psi_{ac}\cdot\psi_{ev}+\psi_{ac}\cdot\psi_{cv}+\psi_{ce}\cdot\psi_{ev}$ | 0.284957 | 0.65498 | 30358.2 |
| 315 | $\overline{\boldsymbol{u}} \sim \psi_{ev}+\psi_{cv}+\psi_{ac}\cdot\psi_{ev}+\psi_{ac}\cdot\psi_{cv}+\psi_{ce}\cdot\psi_{cv}+\psi_{ev}\cdot\psi_{cv}$ | 0.307152 | 0.65481 | 30374 |
| 316 | $\overline{\boldsymbol{u}} \sim \psi_{ev}+\psi_{cv}+\psi_{ac}\cdot\psi_{ev}+\psi_{ac}\cdot\psi_{cv}+\psi_{ce}\cdot\psi_{cv}$ | 0.30237 | 0.65434 | 30414.1 |
| 317 | $\overline{\boldsymbol{u}} \sim \psi_{ac}+\psi_{cv}+\psi_{ac}\cdot\psi_{ev}+\psi_{ac}\cdot\psi_{cv}+\psi_{ce}\cdot\psi_{cv}+\psi_{ev}\cdot\psi_{cv}$ | 0.376912 | 0.65336 | 30501.6 |
| 318 | $\overline{\boldsymbol{u}} \sim \psi_{ac}+\psi_{cv}+\psi_{ac}\cdot\psi_{ev}+\psi_{ac}\cdot\psi_{cv}+\psi_{ce}\cdot\psi_{cv}$ | 0.376335 | 0.6531 | 30523.5 |
| 319 | $\overline{\boldsymbol{u}} \sim \psi_{ac}+\psi_{cv}+\psi_{ac}\cdot\psi_{ev}+\psi_{ac}\cdot\psi_{cv}+\psi_{ce}\cdot\psi_{ev}+\psi_{ev}\cdot\psi_{cv}$ | 0.366314 | 0.65255 | 30573 |
| 320 | $\overline{\boldsymbol{u}} \sim \psi_{ac}+\psi_{cv}+\psi_{ac}\cdot\psi_{ev}+\psi_{ac}\cdot\psi_{cv}+\psi_{ce}\cdot\psi_{ev}$ | 0.366301 | 0.65244 | 30581.2 |

| | | | | |
|---|---|---|---|---|
| 321 | $\overline{\boldsymbol{u}} \sim \psi_{ev}+\psi_{cv}+\psi_{ac}\cdot\psi_{cv}+\psi_{ce}\cdot\psi_{ev}+\psi_{ce}\cdot\psi_{cv}$ | 0.23541 | 0.65153 | 30661.2 |
| 322 | $\overline{\boldsymbol{u}} \sim \psi_{ev}+\psi_{cv}+\psi_{ac}\cdot\psi_{cv}+\psi_{ce}\cdot\psi_{ev}+\psi_{ce}\cdot\psi_{cv}+\psi_{ev}\cdot\psi_{cv}$ | 0.235412 | 0.65152 | 30663.1 |
| 323 | $\overline{\boldsymbol{u}} \sim \psi_{ce}+\psi_{cv}+\psi_{ac}\cdot\psi_{ce}+\psi_{ac}\cdot\psi_{ev}+\psi_{ac}\cdot\psi_{cv}+\psi_{ce}\cdot\psi_{cv}$ | 0.134969 | 0.64979 | 30814.1 |
| 324 | $\overline{\boldsymbol{u}} \sim \psi_{ev}+\psi_{cv}+\psi_{ac}\cdot\psi_{cv}+\psi_{ce}\cdot\psi_{ev}$ | 0.229182 | 0.64897 | 30882.8 |
| 325 | $\overline{\boldsymbol{u}} \sim \psi_{ev}+\psi_{cv}+\psi_{ac}\cdot\psi_{cv}+\psi_{ce}\cdot\psi_{ev}+\psi_{ev}\cdot\psi_{cv}$ | 0.229182 | 0.64896 | 30884.8 |
| 326 | $\overline{\boldsymbol{u}} \sim \psi_{ce}+\psi_{cv}+\psi_{ac}\cdot\psi_{ce}+\psi_{ac}\cdot\psi_{ev}+\psi_{ac}\cdot\psi_{cv}$ | 0.135221 | 0.64834 | 30938.6 |
| 327 | $\overline{\boldsymbol{u}} \sim \psi_{ev}+\psi_{cv}+\psi_{ac}\cdot\psi_{cv}+\psi_{ce}\cdot\psi_{cv}$ | 0.237755 | 0.64725 | 31031.4 |
| 328 | $\overline{\boldsymbol{u}} \sim \psi_{ev}+\psi_{cv}+\psi_{ac}\cdot\psi_{cv}+\psi_{ce}\cdot\psi_{cv}+\psi_{ev}\cdot\psi_{cv}$ | 0.237756 | 0.64725 | 31032.9 |
| 329 | $\overline{\boldsymbol{u}} \sim \psi_{ac}+\psi_{ce}+\psi_{cv}+\psi_{ac}\cdot\psi_{cv}+\psi_{ce}\cdot\psi_{cv}+\psi_{ev}\cdot\psi_{cv}$ | 0.328427 | 0.6462 | 31124 |
| 330 | $\overline{\boldsymbol{u}} \sim \psi_{ac}+\psi_{ce}+\psi_{cv}+\psi_{ac}\cdot\psi_{cv}+\psi_{ev}\cdot\psi_{cv}$ | 0.330183 | 0.64605 | 31136.2 |
| 331 | $\overline{\boldsymbol{u}} \sim \psi_{ce}+\psi_{cv}+\psi_{ac}\cdot\psi_{ev}+\psi_{ac}\cdot\psi_{cv}+\psi_{ce}\cdot\psi_{ev}+\psi_{ce}\cdot\psi_{cv}+\psi_{ev}\cdot\psi_{cv}$ | 0.224603 | 0.64395 | 31318 |
| 332 | $\overline{\boldsymbol{u}} \sim \psi_{ce}+\psi_{cv}+\psi_{ac}\cdot\psi_{cv}+\psi_{ce}\cdot\psi_{ev}+\psi_{ce}\cdot\psi_{cv}+\psi_{ev}\cdot\psi_{cv}$ | 0.222434 | 0.64393 | 31318.9 |
| 333 | $\overline{\boldsymbol{u}} \sim \psi_{ce}+\psi_{cv}+\psi_{ac}\cdot\psi_{ev}+\psi_{ac}\cdot\psi_{cv}+\psi_{ce}\cdot\psi_{ev}+\psi_{ev}\cdot\psi_{cv}$ | 0.22572 | 0.64386 | 31325.1 |
| 334 | $\overline{\boldsymbol{u}} \sim \psi_{ce}+\psi_{cv}+\psi_{ac}\cdot\psi_{cv}+\psi_{ce}\cdot\psi_{ev}+\psi_{ev}\cdot\psi_{cv}$ | 0.223648 | 0.64384 | 31325.6 |
| 335 | $\overline{\boldsymbol{u}} \sim \psi_{ce}+\psi_{ac}\cdot\psi_{ce}+\psi_{ac}\cdot\psi_{ev}+\psi_{ac}\cdot\psi_{cv}+\psi_{ce}\cdot\psi_{cv}+\psi_{ev}\cdot\psi_{cv}$ | 0.121658 | 0.64138 | 31536.3 |
| 336 | $\overline{\boldsymbol{u}} \sim \psi_{ce}+\psi_{ac}\cdot\psi_{ce}+\psi_{ac}\cdot\psi_{ev}+\psi_{ac}\cdot\psi_{cv}+\psi_{ev}\cdot\psi_{cv}$ | 0.121368 | 0.64135 | 31537.6 |
| 337 | $\overline{\boldsymbol{u}} \sim \psi_{ac}+\psi_{ev}+\psi_{cv}+\psi_{ac}\cdot\psi_{ce}+\psi_{ac}\cdot\psi_{ev}+\psi_{ce}\cdot\psi_{ev}+\psi_{ce}\cdot\psi_{cv}+\psi_{ev}\cdot\psi_{cv}$ | 0.139594 | 0.6393 | 31714.1 |
| 338 | $\overline{\boldsymbol{u}} \sim \psi_{ac}+\psi_{ev}+\psi_{cv}+\psi_{ac}\cdot\psi_{ce}+\psi_{ac}\cdot\psi_{ev}+\psi_{ce}\cdot\psi_{ev}+\psi_{ce}\cdot\psi_{cv}$ | 0.140122 | 0.6386 | 31772.3 |
| 339 | $\overline{\boldsymbol{u}} \sim \psi_{cv}+\psi_{ac}\cdot\psi_{ce}+\psi_{ac}\cdot\psi_{ev}+\psi_{ac}\cdot\psi_{cv}+\psi_{ce}\cdot\psi_{ev}+\psi_{ce}\cdot\psi_{cv}+\psi_{ev}\cdot\psi_{cv}$ | 0.184346 | 0.63752 | 31862.8 |
| 340 | $\overline{\boldsymbol{u}} \sim \psi_{cv}+\psi_{ac}\cdot\psi_{ce}+\psi_{ac}\cdot\psi_{cv}+\psi_{ce}\cdot\psi_{ev}+\psi_{ce}\cdot\psi_{cv}+\psi_{ev}\cdot\psi_{cv}$ | 0.188247 | 0.63699 | 31906.3 |
| 341 | $\overline{\boldsymbol{u}} \sim \psi_{ce}+\psi_{cv}+\psi_{ac}\cdot\psi_{ev}+\psi_{ac}\cdot\psi_{cv}+\psi_{ce}\cdot\psi_{cv}+\psi_{ev}\cdot\psi_{cv}$ | 0.207882 | 0.63698 | 31907.4 |
| 342 | $\overline{\boldsymbol{u}} \sim \psi_{ce}+\psi_{cv}+\psi_{ac}\cdot\psi_{ev}+\psi_{ac}\cdot\psi_{cv}+\psi_{ev}\cdot\psi_{cv}$ | 0.208822 | 0.63691 | 31912.6 |
| 343 | $\overline{\boldsymbol{u}} \sim \psi_{ac}+\psi_{ev}+\psi_{cv}+\psi_{ac}\cdot\psi_{ce}+\psi_{ac}\cdot\psi_{cv}$ | 0.414517 | 0.63687 | 31915.6 |
| 344 | $\overline{\boldsymbol{u}} \sim \psi_{ac}+\psi_{ev}+\psi_{cv}+\psi_{ac}\cdot\psi_{ce}+\psi_{ac}\cdot\psi_{ev}+\psi_{ac}\cdot\psi_{cv}$ | 0.414635 | 0.63686 | 31917.5 |
| 345 | $\overline{\boldsymbol{u}} \sim \psi_{ac}+\psi_{ev}+\psi_{cv}+\psi_{ac}\cdot\psi_{ce}+\psi_{ac}\cdot\psi_{cv}+\psi_{ev}\cdot\psi_{cv}$ | 0.414528 | 0.63686 | 31917.1 |
| 346 | $\overline{\boldsymbol{u}} \sim \psi_{ac}+\psi_{ev}+\psi_{cv}+\psi_{ac}\cdot\psi_{ce}+\psi_{ac}\cdot\psi_{ev}+\psi_{ac}\cdot\psi_{cv}+\psi_{ev}\cdot\psi_{cv}$ | 0.414735 | 0.63686 | 31918.7 |
| 347 | $\overline{\boldsymbol{u}} \sim \psi_{ac}+\psi_{ce}+\psi_{cv}+\psi_{ac}\cdot\psi_{cv}+\psi_{ce}\cdot\psi_{ev}+\psi_{ce}\cdot\psi_{cv}$ | 0.294574 | 0.63651 | 31947 |

| | | | | |
|---|---|---|---|---|
| 348 | $\overline{\boldsymbol{u}} \sim \psi_{ac}+\psi_{ev}+\psi_{cv}+\psi_{ac}\cdot\psi_{ce}+\psi_{ce}\cdot\psi_{ev}+\psi_{ce}\cdot\psi_{cv}+\psi_{ev}\cdot\psi_{cv}$ | 0.14022 | 0.63641 | 31956.4 |
| 349 | $\overline{\boldsymbol{u}} \sim \psi_{ac}+\psi_{ev}+\psi_{cv}+\psi_{ac}\cdot\psi_{ce}+\psi_{ce}\cdot\psi_{ev}+\psi_{ce}\cdot\psi_{cv}$ | 0.14032 | 0.63635 | 31960.4 |
| 350 | $\overline{\boldsymbol{u}} \sim \psi_{ac}+\psi_{ce}+\psi_{cv}+\psi_{ac}\cdot\psi_{cv}+\psi_{ce}\cdot\psi_{ev}$ | 0.29631 | 0.63632 | 31961.8 |
| 351 | $\overline{\boldsymbol{u}} \sim \psi_{cv}+\psi_{ac}\cdot\psi_{ce}+\psi_{ac}\cdot\psi_{ev}+\psi_{ac}\cdot\psi_{cv}+\psi_{ce}\cdot\psi_{cv}+\psi_{ev}\cdot\psi_{cv}$ | 0.19211 | 0.63608 | 31983.1 |
| 352 | $\overline{\boldsymbol{u}} \sim \psi_{cv}+\psi_{ac}\cdot\psi_{ce}+\psi_{ac}\cdot\psi_{cv}+\psi_{ce}\cdot\psi_{cv}+\psi_{ev}\cdot\psi_{cv}$ | 0.194145 | 0.63587 | 31999.7 |
| 353 | $\overline{\boldsymbol{u}} \sim \psi_{ce}+\psi_{cv}+\psi_{ac}\cdot\psi_{cv}+\psi_{ce}\cdot\psi_{cv}+\psi_{ev}\cdot\psi_{cv}$ | 0.219041 | 0.63539 | 32039.2 |
| 354 | $\overline{\boldsymbol{u}} \sim \psi_{ce}+\psi_{cv}+\psi_{ac}\cdot\psi_{cv}+\psi_{ev}\cdot\psi_{cv}$ | 0.220281 | 0.63529 | 32046.6 |
| 355 | $\overline{\boldsymbol{u}} \sim \psi_{ac}+\psi_{cv}+\psi_{ac}\cdot\psi_{cv}+\psi_{ce}\cdot\psi_{ev}+\psi_{ce}\cdot\psi_{cv}+\psi_{ev}\cdot\psi_{cv}$ | 0.313193 | 0.63461 | 32105.5 |
| 356 | $\overline{\boldsymbol{u}} \sim \psi_{ac}+\psi_{cv}+\psi_{ac}\cdot\psi_{cv}+\psi_{ce}\cdot\psi_{cv}+\psi_{ev}\cdot\psi_{cv}$ | 0.31232 | 0.63445 | 32117.7 |
| 357 | $\overline{\boldsymbol{u}} \sim \psi_{ce}+\psi_{cv}+\psi_{ac}\cdot\psi_{ev}+\psi_{ac}\cdot\psi_{cv}+\psi_{ce}\cdot\psi_{ev}+\psi_{ce}\cdot\psi_{cv}$ | 0.197306 | 0.63397 | 32159.1 |
| 358 | $\overline{\boldsymbol{u}} \sim \psi_{ce}+\psi_{cv}+\psi_{ac}\cdot\psi_{ev}+\psi_{ac}\cdot\psi_{cv}+\psi_{ce}\cdot\psi_{ev}$ | 0.198151 | 0.63391 | 32163.2 |
| 359 | $\overline{\boldsymbol{u}} \sim \psi_{ev}+\psi_{cv}+\psi_{ac}\cdot\psi_{ce}+\psi_{ac}\cdot\psi_{ev}+\psi_{ac}\cdot\psi_{cv}+\psi_{ev}\cdot\psi_{cv}$ | 0.306283 | 0.63201 | 32321.3 |
| 360 | $\overline{\boldsymbol{u}} \sim \psi_{ev}+\psi_{cv}+\psi_{ac}\cdot\psi_{ce}+\psi_{ac}\cdot\psi_{ev}+\psi_{ac}\cdot\psi_{cv}$ | 0.302271 | 0.63171 | 32345.4 |
| 361 | $\overline{\boldsymbol{u}} \sim \psi_{ac}+\psi_{cv}+\psi_{ac}\cdot\psi_{ce}+\psi_{ac}\cdot\psi_{ev}+\psi_{ac}\cdot\psi_{cv}+\psi_{ev}\cdot\psi_{cv}$ | 0.364656 | 0.62901 | 32568.3 |
| 362 | $\overline{\boldsymbol{u}} \sim \psi_{ac}+\psi_{cv}+\psi_{ac}\cdot\psi_{ce}+\psi_{ac}\cdot\psi_{ev}+\psi_{ac}\cdot\psi_{cv}$ | 0.364135 | 0.62878 | 32586.7 |
| 363 | $\overline{\boldsymbol{u}} \sim \psi_{cv}+\psi_{ac}\cdot\psi_{ev}+\psi_{ac}\cdot\psi_{cv}+\psi_{ce}\cdot\psi_{cv}+\psi_{ev}\cdot\psi_{cv}$ | 0.211652 | 0.62788 | 32660.1 |
| 364 | $\overline{\boldsymbol{u}} \sim \psi_{ac}+\psi_{ev}+\psi_{cv}+\psi_{ac}\cdot\psi_{ev}+\psi_{ac}\cdot\psi_{cv}+\psi_{ev}\cdot\psi_{cv}$ | 0.764406 | 0.62787 | 32661.7 |
| 365 | $\overline{\boldsymbol{u}} \sim \psi_{cv}+\psi_{ac}\cdot\psi_{ev}+\psi_{ac}\cdot\psi_{cv}+\psi_{ce}\cdot\psi_{ev}+\psi_{ce}\cdot\psi_{cv}+\psi_{ev}\cdot\psi_{cv}$ | 0.211476 | 0.62787 | 32662 |
| 366 | $\overline{\boldsymbol{u}} \sim \psi_{ac}+\psi_{ev}+\psi_{cv}+\psi_{ac}\cdot\psi_{ev}+\psi_{ac}\cdot\psi_{cv}$ | 0.762867 | 0.62786 | 32662 |
| 367 | $\overline{\boldsymbol{u}} \sim \psi_{ac}+\psi_{ev}+\psi_{cv}+\psi_{ac}\cdot\psi_{cv}$ | 0.757617 | 0.62782 | 32663.9 |
| 368 | $\overline{\boldsymbol{u}} \sim \psi_{ac}+\psi_{ev}+\psi_{cv}+\psi_{ac}\cdot\psi_{cv}+\psi_{ev}\cdot\psi_{cv}$ | 0.75773 | 0.62782 | 32665.3 |
| 369 | $\overline{\boldsymbol{u}} \sim \psi_{ce}+\psi_{cv}+\psi_{ac}\cdot\psi_{cv}+\psi_{ce}\cdot\psi_{ev}+\psi_{ce}\cdot\psi_{cv}$ | 0.212166 | 0.62767 | 32677.3 |
| 370 | $\overline{\boldsymbol{u}} \sim \psi_{ev}+\psi_{cv}+\psi_{ac}\cdot\psi_{ce}+\psi_{ac}\cdot\psi_{cv}$ | 0.251246 | 0.62764 | 32679.2 |
| 371 | $\overline{\boldsymbol{u}} \sim \psi_{ev}+\psi_{cv}+\psi_{ac}\cdot\psi_{ce}+\psi_{ac}\cdot\psi_{cv}+\psi_{ev}\cdot\psi_{cv}$ | 0.251246 | 0.62763 | 32680.8 |
| 372 | $\overline{\boldsymbol{u}} \sim \psi_{ce}+\psi_{cv}+\psi_{ac}\cdot\psi_{cv}+\psi_{ce}\cdot\psi_{ev}$ | 0.213449 | 0.62755 | 32685.9 |
| 373 | $\overline{\boldsymbol{u}} \sim \psi_{cv}+\psi_{ac}\cdot\psi_{cv}+\psi_{ce}\cdot\psi_{ev}+\psi_{ce}\cdot\psi_{cv}+\psi_{ev}\cdot\psi_{cv}$ | 0.22536 | 0.62603 | 32811.1 |
| 374 | $\overline{\boldsymbol{u}} \sim \psi_{cv}+\psi_{ac}\cdot\psi_{cv}+\psi_{ce}\cdot\psi_{cv}+\psi_{ev}\cdot\psi_{cv}$ | 0.224434 | 0.62582 | 32827.6 |

| | | | | |
|---|---|---|---|---|
| 375 | $\overline{\boldsymbol{u}} \sim \psi_{ce}+\psi_{cv}+\psi_{ac}\cdot\psi_{ev}+\psi_{ac}\cdot\psi_{cv}+\psi_{ce}\cdot\psi_{cv}$ | 0.177195 | 0.62213 | 33126.8 |
| 376 | $\overline{\boldsymbol{u}} \sim \psi_{ce}+\psi_{cv}+\psi_{ac}\cdot\psi_{ev}+\psi_{ac}\cdot\psi_{cv}$ | 0.177726 | 0.62211 | 33127.7 |
| 377 | $\overline{\boldsymbol{u}} \sim \psi_{ac}+\psi_{cv}+\psi_{ac}\cdot\psi_{ce}+\psi_{ac}\cdot\psi_{ev}+\psi_{ce}\cdot\psi_{ev}+\psi_{ce}\cdot\psi_{cv}$ | 0.149471 | 0.6203 | 33275.4 |
| 378 | $\overline{\boldsymbol{u}} \sim \psi_{ac}+\psi_{cv}+\psi_{ac}\cdot\psi_{ce}+\psi_{ac}\cdot\psi_{ev}+\psi_{ce}\cdot\psi_{ev}+\psi_{ce}\cdot\psi_{cv}+\psi_{ev}\cdot\psi_{cv}$ | 0.149478 | 0.62029 | 33277.4 |
| 379 | $\overline{\boldsymbol{u}} \sim \psi_{ac}+\psi_{cv}+\psi_{ac}\cdot\psi_{ce}+\psi_{ac}\cdot\psi_{cv}+\psi_{ce}\cdot\psi_{ev}+\psi_{ev}\cdot\psi_{cv}$ | 0.280202 | 0.61794 | 33463.9 |
| 380 | $\overline{\boldsymbol{u}} \sim \psi_{ac}+\psi_{cv}+\psi_{ac}\cdot\psi_{cv}+\psi_{ce}\cdot\psi_{ev}+\psi_{ev}\cdot\psi_{cv}$ | 0.284613 | 0.61774 | 33478.9 |
| 381 | $\overline{\boldsymbol{u}} \sim \psi_{cv}+\psi_{ac}\cdot\psi_{ce}+\psi_{ac}\cdot\psi_{ev}+\psi_{ac}\cdot\psi_{cv}+\psi_{ce}\cdot\psi_{ev}+\psi_{ce}\cdot\psi_{cv}$ | 0.172469 | 0.61711 | 33530.1 |
| 382 | $\overline{\boldsymbol{u}} \sim \psi_{cv}+\psi_{ac}\cdot\psi_{ce}+\psi_{ac}\cdot\psi_{ev}+\psi_{ac}\cdot\psi_{cv}+\psi_{ce}\cdot\psi_{cv}$ | 0.17075 | 0.61704 | 33534.3 |
| 383 | $\overline{\boldsymbol{u}} \sim \psi_{ev}+\psi_{cv}+\psi_{ac}\cdot\psi_{ev}+\psi_{ac}\cdot\psi_{cv}+\psi_{ev}\cdot\psi_{cv}$ | 0.300549 | 0.61671 | 33560.5 |
| 384 | $\overline{\boldsymbol{u}} \sim \psi_{ac}+\psi_{cv}+\psi_{ac}\cdot\psi_{ev}+\psi_{ac}\cdot\psi_{cv}+\psi_{ev}\cdot\psi_{cv}$ | 0.416583 | 0.61641 | 33584.6 |
| 385 | $\overline{\boldsymbol{u}} \sim \psi_{cv}+\psi_{ac}\cdot\psi_{ce}+\psi_{ac}\cdot\psi_{ev}+\psi_{ac}\cdot\psi_{cv}+\psi_{ce}\cdot\psi_{ev}+\psi_{ev}\cdot\psi_{cv}$ | 0.193374 | 0.61637 | 33588.8 |
| 386 | $\overline{\boldsymbol{u}} \sim \psi_{cv}+\psi_{ac}\cdot\psi_{ev}+\psi_{ac}\cdot\psi_{cv}+\psi_{ce}\cdot\psi_{ev}+\psi_{ev}\cdot\psi_{cv}$ | 0.191305 | 0.61633 | 33590.6 |
| 387 | $\overline{\boldsymbol{u}} \sim \psi_{ev}+\psi_{cv}+\psi_{ac}\cdot\psi_{ev}+\psi_{ac}\cdot\psi_{cv}$ | 0.295058 | 0.61623 | 33598.2 |
| 388 | $\overline{\boldsymbol{u}} \sim \psi_{ac}+\psi_{cv}+\psi_{ac}\cdot\psi_{ev}+\psi_{ac}\cdot\psi_{cv}$ | 0.415675 | 0.61613 | 33606.1 |
| 389 | $\overline{\boldsymbol{u}} \sim \psi_{cv}+\psi_{ac}\cdot\psi_{ev}+\psi_{ac}\cdot\psi_{cv}+\psi_{ce}\cdot\psi_{ev}+\psi_{ce}\cdot\psi_{cv}$ | 0.185417 | 0.61465 | 33724.1 |
| 390 | $\overline{\boldsymbol{u}} \sim \psi_{cv}+\psi_{ac}\cdot\psi_{ev}+\psi_{ac}\cdot\psi_{cv}+\psi_{ce}\cdot\psi_{cv}$ | 0.181716 | 0.61413 | 33764.2 |
| 391 | $\overline{\boldsymbol{u}} \sim \psi_{ac}+\psi_{cv}+\psi_{ac}\cdot\psi_{ce}+\psi_{ac}\cdot\psi_{cv}+\psi_{ev}\cdot\psi_{cv}$ | 0.303596 | 0.61036 | 34061.3 |
| 392 | $\overline{\boldsymbol{u}} \sim \psi_{cv}+\psi_{ac}\cdot\psi_{ce}+\psi_{ac}\cdot\psi_{cv}+\psi_{ce}\cdot\psi_{ev}+\psi_{ev}\cdot\psi_{cv}$ | 0.215945 | 0.61015 | 34077 |
| 393 | $\overline{\boldsymbol{u}} \sim \psi_{cv}+\psi_{ac}\cdot\psi_{cv}+\psi_{ce}\cdot\psi_{ev}+\psi_{ev}\cdot\psi_{cv}$ | 0.211144 | 0.60997 | 34090.3 |
| 394 | $\overline{\boldsymbol{u}} \sim \psi_{cv}+\psi_{ac}\cdot\psi_{ce}+\psi_{ac}\cdot\psi_{ev}+\psi_{ac}\cdot\psi_{cv}+\psi_{ev}\cdot\psi_{cv}$ | 0.217422 | 0.60992 | 34095.3 |
| 395 | $\overline{\boldsymbol{u}} \sim \psi_{ev}+\psi_{cv}+\psi_{ac}\cdot\psi_{cv}$ | 0.227092 | 0.60939 | 34134.8 |
| 396 | $\overline{\boldsymbol{u}} \sim \psi_{ev}+\psi_{cv}+\psi_{ac}\cdot\psi_{cv}+\psi_{ev}\cdot\psi_{cv}$ | 0.227093 | 0.60938 | 34136.4 |
| 397 | $\overline{\boldsymbol{u}} \sim \psi_{cv}+\psi_{ac}\cdot\psi_{ce}+\psi_{ac}\cdot\psi_{cv}+\psi_{ev}\cdot\psi_{cv}$ | 0.236421 | 0.60578 | 34415.4 |
| 398 | $\overline{\boldsymbol{u}} \sim \psi_{cv}+\psi_{ac}\cdot\psi_{ce}+\psi_{ac}\cdot\psi_{ev}+\psi_{ac}\cdot\psi_{cv}+\psi_{ce}\cdot\psi_{ev}$ | 0.179714 | 0.60161 | 34737.3 |
| 399 | $\overline{\boldsymbol{u}} \sim \psi_{cv}+\psi_{ac}\cdot\psi_{ce}+\psi_{ac}\cdot\psi_{ev}+\psi_{ac}\cdot\psi_{cv}$ | 0.19083 | 0.60014 | 34848.6 |
| 400 | $\overline{\boldsymbol{u}} \sim \psi_{cv}+\psi_{ac}\cdot\psi_{ev}+\psi_{ac}\cdot\psi_{cv}+\psi_{ce}\cdot\psi_{ev}$ | 0.163927 | 0.59848 | 34974.1 |
| 401 | $\overline{\boldsymbol{u}} \sim \psi_{ac}+\psi_{cv}+\psi_{ac}\cdot\psi_{cv}+\psi_{ev}\cdot\psi_{cv}$ | 0.317081 | 0.59688 | 35095.5 |

| | | | | |
|---|---|---|---|---|
| 402 | $\overline{\boldsymbol{u}} \sim \psi_{cv}+\psi_{ac}\cdot\psi_{ev}+\psi_{ac}\cdot\psi_{cv}+\psi_{ev}\cdot\psi_{cv}$ | 0.200637 | 0.59052 | 35571.9 |
| 403 | $\overline{\boldsymbol{u}} \sim \psi_{cv}+\psi_{ac}\cdot\psi_{cv}+\psi_{ev}\cdot\psi_{cv}$ | 0.215025 | 0.58825 | 35739.4 |
| 404 | $\overline{\boldsymbol{u}} \sim \psi_{ac}+\psi_{cv}+\psi_{ac}\cdot\psi_{ce}+\psi_{ac}\cdot\psi_{cv}+\psi_{ce}\cdot\psi_{ev}+\psi_{ce}\cdot\psi_{cv}$ | 0.247766 | 0.58619 | 35894.1 |
| 405 | $\overline{\boldsymbol{u}} \sim \psi_{ac}+\psi_{cv}+\psi_{ac}\cdot\psi_{cv}+\psi_{ce}\cdot\psi_{ev}+\psi_{ce}\cdot\psi_{cv}$ | 0.263359 | 0.58204 | 36197.4 |
| 406 | $\overline{\boldsymbol{u}} \sim \psi_{ac}+\psi_{ev}+\psi_{ac}\cdot\psi_{ce}+\psi_{ac}\cdot\psi_{ev}+\psi_{ac}\cdot\psi_{cv}+\psi_{ce}\cdot\psi_{ev}+\psi_{ce}\cdot\psi_{cv}+\psi_{ev}\cdot\psi_{cv}$ | 0.151932 | 0.57941 | 36391.2 |
| 407 | $\overline{\boldsymbol{u}} \sim \psi_{ac}+\psi_{ev}+\psi_{ac}\cdot\psi_{ce}+\psi_{ac}\cdot\psi_{cv}+\psi_{ce}\cdot\psi_{ev}+\psi_{ce}\cdot\psi_{cv}+\psi_{ev}\cdot\psi_{cv}$ | 0.151627 | 0.57931 | 36397.6 |
| 408 | $\overline{\boldsymbol{u}} \sim \psi_{ac}+\psi_{ev}+\psi_{ac}\cdot\psi_{ce}+\psi_{ce}\cdot\psi_{ev}+\psi_{ce}\cdot\psi_{cv}+\psi_{ev}\cdot\psi_{cv}$ | 0.144689 | 0.57853 | 36452.9 |
| 409 | $\overline{\boldsymbol{u}} \sim \psi_{ac}+\psi_{ev}+\psi_{ac}\cdot\psi_{ce}+\psi_{ac}\cdot\psi_{ev}+\psi_{ce}\cdot\psi_{ev}+\psi_{ce}\cdot\psi_{cv}+\psi_{ev}\cdot\psi_{cv}$ | 0.144576 | 0.57853 | 36453.5 |
| 410 | $\overline{\boldsymbol{u}} \sim \psi_{cv}+\psi_{ac}\cdot\psi_{ce}+\psi_{ac}\cdot\psi_{cv}+\psi_{ce}\cdot\psi_{ev}+\psi_{ce}\cdot\psi_{cv}$ | 0.206461 | 0.57831 | 36467.3 |
| 411 | $\overline{\boldsymbol{u}} \sim \psi_{ce}+\psi_{cv}+\psi_{ac}\cdot\psi_{ce}+\psi_{ac}\cdot\psi_{cv}+\psi_{ce}\cdot\psi_{cv}$ | 0.127895 | 0.57768 | 36512.8 |
| 412 | $\overline{\boldsymbol{u}} \sim \psi_{cv}+\psi_{ac}\cdot\psi_{ev}+\psi_{ac}\cdot\psi_{cv}$ | 0.170925 | 0.57764 | 36514.2 |
| 413 | $\overline{\boldsymbol{u}} \sim \psi_{cv}+\psi_{ac}\cdot\psi_{cv}+\psi_{ce}\cdot\psi_{ev}+\psi_{ce}\cdot\psi_{cv}$ | 0.216358 | 0.57754 | 36522.1 |
| 414 | $\overline{\boldsymbol{u}} \sim \psi_{ce}+\psi_{cv}+\psi_{ac}\cdot\psi_{ce}+\psi_{ac}\cdot\psi_{cv}$ | 0.128709 | 0.57512 | 36696.3 |
| 415 | $\overline{\boldsymbol{u}} \sim \psi_{ac}+\psi_{ac}\cdot\psi_{ce}+\psi_{ac}\cdot\psi_{ev}+\psi_{ac}\cdot\psi_{cv}+\psi_{ce}\cdot\psi_{ev}+\psi_{ce}\cdot\psi_{cv}+\psi_{ev}\cdot\psi_{cv}$ | 0.159933 | 0.56961 | 37091.2 |
| 416 | $\overline{\boldsymbol{u}} \sim \psi_{ac}+\psi_{ac}\cdot\psi_{ce}+\psi_{ac}\cdot\psi_{ev}+\psi_{ce}\cdot\psi_{ev}+\psi_{ce}\cdot\psi_{cv}+\psi_{ev}\cdot\psi_{cv}$ | 0.150628 | 0.56775 | 37221.8 |
| 417 | $\overline{\boldsymbol{u}} \sim \psi_{ac}+\psi_{ac}\cdot\psi_{ce}+\psi_{ac}\cdot\psi_{cv}+\psi_{ce}\cdot\psi_{ev}+\psi_{ce}\cdot\psi_{cv}+\psi_{ev}\cdot\psi_{cv}$ | 0.166376 | 0.55788 | 37909 |
| 418 | $\overline{\boldsymbol{u}} \sim \psi_{ac}+\psi_{cv}+\psi_{ac}\cdot\psi_{ce}+\psi_{ce}\cdot\psi_{ev}+\psi_{ce}\cdot\psi_{cv}+\psi_{ev}\cdot\psi_{cv}$ | 0.141526 | 0.55683 | 37981.5 |
| 419 | $\overline{\boldsymbol{u}} \sim \psi_{ac}+\psi_{ce}+\psi_{ev}+\psi_{ac}\cdot\psi_{ev}+\psi_{ac}\cdot\psi_{cv}+\psi_{ce}\cdot\psi_{ev}+\psi_{ce}\cdot\psi_{cv}+\psi_{ev}\cdot\psi_{cv}$ | 0.248422 | 0.55534 | 38085.6 |
| 420 | $\overline{\boldsymbol{u}} \sim \psi_{ac}+\psi_{ev}+\psi_{ac}\cdot\psi_{ce}+\psi_{ac}\cdot\psi_{ev}+\psi_{ac}\cdot\psi_{cv}+\psi_{ce}\cdot\psi_{ev}+\psi_{ev}\cdot\psi_{cv}$ | 0.177404 | 0.55506 | 38104 |
| 421 | $\overline{\boldsymbol{u}} \sim \psi_{ac}+\psi_{ev}+\psi_{ac}\cdot\psi_{ce}+\psi_{ac}\cdot\psi_{cv}+\psi_{ce}\cdot\psi_{ev}+\psi_{ev}\cdot\psi_{cv}$ | 0.177282 | 0.55505 | 38103 |
| 422 | $\overline{\boldsymbol{u}} \sim \psi_{ac}+\psi_{ce}+\psi_{ev}+\psi_{ac}\cdot\psi_{ev}+\psi_{ac}\cdot\psi_{cv}+\psi_{ce}\cdot\psi_{cv}+\psi_{ev}\cdot\psi_{cv}$ | 0.248494 | 0.55479 | 38122 |
| 423 | $\overline{\boldsymbol{u}} \sim \psi_{ac}+\psi_{ce}+\psi_{ac}\cdot\psi_{ev}+\psi_{ac}\cdot\psi_{cv}+\psi_{ce}\cdot\psi_{ev}+\psi_{ce}\cdot\psi_{cv}+\psi_{ev}\cdot\psi_{cv}$ | 0.24314 | 0.55438 | 38149.9 |
| 424 | $\overline{\boldsymbol{u}} \sim \psi_{ac}+\psi_{ce}+\psi_{ac}\cdot\psi_{ev}+\psi_{ac}\cdot\psi_{cv}+\psi_{ce}\cdot\psi_{cv}+\psi_{ev}\cdot\psi_{cv}$ | 0.242808 | 0.55339 | 38216.5 |
| 425 | $\overline{\boldsymbol{u}} \sim \psi_{ac}+\psi_{ce}+\psi_{ev}+\psi_{ac}\cdot\psi_{cv}+\psi_{ce}\cdot\psi_{ev}+\psi_{ce}\cdot\psi_{cv}+\psi_{ev}\cdot\psi_{cv}$ | 0.248366 | 0.55275 | 38261.1 |
| 426 | $\overline{\boldsymbol{u}} \sim \psi_{ac}+\psi_{ev}+\psi_{cv}+\psi_{ac}\cdot\psi_{ce}+\psi_{ac}\cdot\psi_{ev}+\psi_{ce}\cdot\psi_{cv}$ | 0.166344 | 0.55258 | 38271.7 |
| 427 | $\overline{\boldsymbol{u}} \sim \psi_{ac}+\psi_{ce}+\psi_{ev}+\psi_{ac}\cdot\psi_{cv}+\psi_{ce}\cdot\psi_{cv}+\psi_{ev}\cdot\psi_{cv}$ | 0.24841 | 0.55257 | 38272.6 |
| 428 | $\overline{\boldsymbol{u}} \sim \psi_{ac}+\psi_{ev}+\psi_{cv}+\psi_{ac}\cdot\psi_{ce}+\psi_{ac}\cdot\psi_{ev}+\psi_{ce}\cdot\psi_{cv}+\psi_{ev}\cdot\psi_{cv}$ | 0.166357 | 0.55257 | 38273.4 |

| | | | | |
|---|---|---|---|---|
| 429 | $\overline{\boldsymbol{u}} \sim \psi_{ac}+\psi_{ce}+\psi_{ac}\cdot\psi_{cv}+\psi_{ce}\cdot\psi_{ev}+\psi_{ce}\cdot\psi_{cv}+\psi_{ev}\cdot\psi_{cv}$ | 0.252696 | 0.55252 | 38276.3 |
| 430 | $\overline{\boldsymbol{u}} \sim \psi_{ac}+\psi_{ce}+\psi_{ac}\cdot\psi_{cv}+\psi_{ce}\cdot\psi_{cv}+\psi_{ev}\cdot\psi_{cv}$ | 0.251473 | 0.55251 | 38276 |
| 431 | $\overline{\boldsymbol{u}} \sim \psi_{ac}+\psi_{ev}+\psi_{cv}+\psi_{ac}\cdot\psi_{ce}+\psi_{ce}\cdot\psi_{cv}$ | 0.166171 | 0.5525 | 38276.3 |
| 432 | $\overline{\boldsymbol{u}} \sim \psi_{ac}+\psi_{ev}+\psi_{cv}+\psi_{ac}\cdot\psi_{ce}+\psi_{ce}\cdot\psi_{cv}+\psi_{ev}\cdot\psi_{cv}$ | 0.166168 | 0.55249 | 38278.1 |
| 433 | $\overline{\boldsymbol{u}} \sim \psi_{ce}+\psi_{ev}+\psi_{ac}\cdot\psi_{ev}+\psi_{ac}\cdot\psi_{cv}+\psi_{ce}\cdot\psi_{ev}+\psi_{ce}\cdot\psi_{cv}+\psi_{ev}\cdot\psi_{cv}$ | 0.211331 | 0.55014 | 38438.6 |
| 434 | $\overline{\boldsymbol{u}} \sim \psi_{ce}+\psi_{ev}+\psi_{ac}\cdot\psi_{ev}+\psi_{ac}\cdot\psi_{cv}+\psi_{ce}\cdot\psi_{cv}+\psi_{ev}\cdot\psi_{cv}$ | 0.209004 | 0.54879 | 38528.8 |
| 435 | $\overline{\boldsymbol{u}} \sim \psi_{ac}+\psi_{cv}+\psi_{ac}\cdot\psi_{ce}+\psi_{ac}\cdot\psi_{ev}+\psi_{ce}\cdot\psi_{cv}+\psi_{ev}\cdot\psi_{cv}$ | 0.166266 | 0.54817 | 38570.3 |
| 436 | $\overline{\boldsymbol{u}} \sim \psi_{ac}+\psi_{cv}+\psi_{ac}\cdot\psi_{ce}+\psi_{ac}\cdot\psi_{ev}+\psi_{ce}\cdot\psi_{cv}$ | 0.166453 | 0.54809 | 38574.6 |
| 437 | $\overline{\boldsymbol{u}} \sim \psi_{ac}+\psi_{ac}\cdot\psi_{ce}+\psi_{ac}\cdot\psi_{ev}+\psi_{ac}\cdot\psi_{cv}+\psi_{ce}\cdot\psi_{ev}+\psi_{ev}\cdot\psi_{cv}$ | 0.184996 | 0.54527 | 38765.3 |
| 438 | $\overline{\boldsymbol{u}} \sim \psi_{ac}+\psi_{ac}\cdot\psi_{ce}+\psi_{ce}\cdot\psi_{ev}+\psi_{ce}\cdot\psi_{cv}+\psi_{ev}\cdot\psi_{cv}$ | 0.14395 | 0.54496 | 38785.2 |
| 439 | $\overline{\boldsymbol{u}} \sim \psi_{ac}+\psi_{ev}+\psi_{cv}+\psi_{ac}\cdot\psi_{ce}+\psi_{ac}\cdot\psi_{ev}+\psi_{ce}\cdot\psi_{ev}+\psi_{ev}\cdot\psi_{cv}$ | 0.156158 | 0.54221 | 38970.3 |
| 440 | $\overline{\boldsymbol{u}} \sim \psi_{ac}+\psi_{cv}+\psi_{ac}\cdot\psi_{ce}+\psi_{ac}\cdot\psi_{cv}+\psi_{ce}\cdot\psi_{cv}$ | 0.298681 | 0.54147 | 39017.6 |
| 441 | $\overline{\boldsymbol{u}} \sim \psi_{ac}+\psi_{ev}+\psi_{cv}+\psi_{ac}\cdot\psi_{ce}+\psi_{ac}\cdot\psi_{ev}+\psi_{ce}\cdot\psi_{ev}$ | 0.156738 | 0.54107 | 39045.2 |
| 442 | $\overline{\boldsymbol{u}} \sim \psi_{ce}+\psi_{ac}\cdot\psi_{ev}+\psi_{ac}\cdot\psi_{cv}+\psi_{ce}\cdot\psi_{ev}+\psi_{ce}\cdot\psi_{cv}+\psi_{ev}\cdot\psi_{cv}$ | 0.179529 | 0.54106 | 39045.7 |
| 443 | $\overline{\boldsymbol{u}} \sim \psi_{ev}+\psi_{ac}\cdot\psi_{ce}+\psi_{ac}\cdot\psi_{ev}+\psi_{ac}\cdot\psi_{cv}+\psi_{ce}\cdot\psi_{ev}+\psi_{ce}\cdot\psi_{cv}+\psi_{ev}\cdot\psi_{cv}$ | 0.158435 | 0.53993 | 39121.8 |
| 444 | $\overline{\boldsymbol{u}} \sim \psi_{ce}+\psi_{cv}+\psi_{ac}\cdot\psi_{ce}+\psi_{ac}\cdot\psi_{ev}+\psi_{ce}\cdot\psi_{ev}+\psi_{ce}\cdot\psi_{cv}+\psi_{ev}\cdot\psi_{cv}$ | 0.0982713 | 0.5389 | 39189.8 |
| 445 | $\overline{\boldsymbol{u}} \sim \psi_{ac}+\psi_{ev}+\psi_{cv}+\psi_{ac}\cdot\psi_{ce}+\psi_{ce}\cdot\psi_{ev}+\psi_{ev}\cdot\psi_{cv}$ | 0.155232 | 0.53733 | 39292.1 |
| 446 | $\overline{\boldsymbol{u}} \sim \psi_{ac}+\psi_{ev}+\psi_{cv}+\psi_{ac}\cdot\psi_{ce}+\psi_{ce}\cdot\psi_{ev}$ | 0.155388 | 0.53724 | 39297.3 |
| 447 | $\overline{\boldsymbol{u}} \sim \psi_{ac}+\psi_{cv}+\psi_{ac}\cdot\psi_{ce}+\psi_{ac}\cdot\psi_{cv}+\psi_{ce}\cdot\psi_{ev}$ | 0.276461 | 0.53712 | 39305 |
| 448 | $\overline{\boldsymbol{u}} \sim \psi_{ac}+\psi_{ce}+\psi_{cv}+\psi_{ac}\cdot\psi_{cv}$ | 0.754059 | 0.53709 | 39306.3 |
| 449 | $\overline{\boldsymbol{u}} \sim \psi_{ac}+\psi_{ce}+\psi_{cv}+\psi_{ac}\cdot\psi_{cv}+\psi_{ce}\cdot\psi_{cv}$ | 0.749978 | 0.53708 | 39307.9 |
| 450 | $\overline{\boldsymbol{u}} \sim \psi_{cv}+\psi_{ac}\cdot\psi_{ce}+\psi_{ac}\cdot\psi_{cv}+\psi_{ce}\cdot\psi_{ev}$ | 0.264583 | 0.53699 | 39312.7 |
| 451 | $\overline{\boldsymbol{u}} \sim \psi_{ac}+\psi_{ev}+\psi_{ac}\cdot\psi_{ce}+\psi_{ac}\cdot\psi_{ev}+\psi_{ac}\cdot\psi_{cv}+\psi_{ce}\cdot\psi_{ev}+\psi_{ce}\cdot\psi_{cv}$ | 0.11874 | 0.5342 | 39498.7 |
| 452 | $\overline{\boldsymbol{u}} \sim \psi_{ev}+\psi_{cv}+\psi_{ac}\cdot\psi_{ce}+\psi_{ac}\cdot\psi_{ev}+\psi_{ce}\cdot\psi_{ev}+\psi_{ce}\cdot\psi_{cv}+\psi_{ev}\cdot\psi_{cv}$ | 0.144436 | 0.5333 | 39557.3 |
| 453 | $\overline{\boldsymbol{u}} \sim \psi_{ac}+\psi_{ce}+\psi_{ev}+\psi_{ac}\cdot\psi_{ev}+\psi_{ac}\cdot\psi_{cv}+\psi_{ce}\cdot\psi_{ev}+\psi_{ev}\cdot\psi_{cv}$ | 0.265224 | 0.53317 | 39565.9 |
| 454 | $\overline{\boldsymbol{u}} \sim \psi_{ac}+\psi_{ev}+\psi_{ac}\cdot\psi_{ce}+\psi_{ac}\cdot\psi_{ev}+\psi_{ce}\cdot\psi_{ev}+\psi_{ev}\cdot\psi_{cv}$ | 0.153277 | 0.53294 | 39579.6 |
| 455 | $\overline{\boldsymbol{u}} \sim \psi_{ac}+\psi_{cv}+\psi_{ac}\cdot\psi_{ce}+\psi_{ce}\cdot\psi_{cv}+\psi_{ev}\cdot\psi_{cv}$ | 0.154614 | 0.53276 | 39590.5 |

| | | | | |
|---|---|---|---|---|
| 456 | $\overline{\boldsymbol{u}} \sim \psi_{ce}+\psi_{ac}\cdot\psi_{ev}+\psi_{ac}\cdot\psi_{cv}+\psi_{ce}\cdot\psi_{cv}+\psi_{ev}\cdot\psi_{cv}$ | 0.167574 | 0.53267 | 39596.2 |
| 457 | $\overline{\boldsymbol{u}} \sim \psi_{ac}+\psi_{ce}+\psi_{ac}\cdot\psi_{ev}+\psi_{ac}\cdot\psi_{cv}+\psi_{ce}\cdot\psi_{ev}+\psi_{ev}\cdot\psi_{cv}$ | 0.259334 | 0.53263 | 39600.1 |
| 458 | $\overline{\boldsymbol{u}} \sim \psi_{ac}+\psi_{ce}+\psi_{ev}+\psi_{ac}\cdot\psi_{ev}+\psi_{ac}\cdot\psi_{cv}+\psi_{ev}\cdot\psi_{cv}$ | 0.265564 | 0.53243 | 39613.3 |
| 459 | $\overline{\boldsymbol{u}} \sim \psi_{ac}+\psi_{ce}+\psi_{ac}\cdot\psi_{ev}+\psi_{ac}\cdot\psi_{cv}+\psi_{ev}\cdot\psi_{cv}$ | 0.25868 | 0.53151 | 39672.1 |
| 460 | $\overline{\boldsymbol{u}} \sim \psi_{ac}+\psi_{ev}+\psi_{ac}\cdot\psi_{ce}+\psi_{ce}\cdot\psi_{ev}+\psi_{ev}\cdot\psi_{cv}$ | 0.152631 | 0.53118 | 39693.4 |
| 461 | $\overline{\boldsymbol{u}} \sim \psi_{ac}+\psi_{ac}\cdot\psi_{ce}+\psi_{ac}\cdot\psi_{cv}+\psi_{ce}\cdot\psi_{ev}+\psi_{ev}\cdot\psi_{cv}$ | 0.191154 | 0.53045 | 39741 |
| 462 | $\overline{\boldsymbol{u}} \sim \psi_{ac}+\psi_{ev}+\psi_{ac}\cdot\psi_{ce}+\psi_{ac}\cdot\psi_{cv}+\psi_{ce}\cdot\psi_{ev}+\psi_{ce}\cdot\psi_{cv}$ | 0.117095 | 0.52996 | 39773.8 |
| 463 | $\overline{\boldsymbol{u}} \sim \psi_{ac}+\psi_{ce}+\psi_{ev}+\psi_{ac}\cdot\psi_{cv}+\psi_{ce}\cdot\psi_{ev}+\psi_{ev}\cdot\psi_{cv}$ | 0.267739 | 0.5297 | 39790 |
| 464 | $\overline{\boldsymbol{u}} \sim \psi_{ac}+\psi_{ce}+\psi_{ev}+\psi_{ac}\cdot\psi_{cv}+\psi_{ev}\cdot\psi_{cv}$ | 0.267809 | 0.52945 | 39805.6 |
| 465 | $\overline{\boldsymbol{u}} \sim \psi_{ev}+\psi_{ac}\cdot\psi_{ce}+\psi_{ac}\cdot\psi_{ev}+\psi_{ac}\cdot\psi_{cv}+\psi_{ce}\cdot\psi_{ev}+\psi_{ev}\cdot\psi_{cv}$ | 0.165614 | 0.52824 | 39884.9 |
| 466 | $\overline{\boldsymbol{u}} \sim \psi_{ac}+\psi_{ce}+\psi_{ac}\cdot\psi_{cv}+\psi_{ce}\cdot\psi_{ev}+\psi_{ev}\cdot\psi_{cv}$ | 0.29055 | 0.52818 | 39887.9 |
| 467 | $\overline{\boldsymbol{u}} \sim \psi_{ac}+\psi_{ce}+\psi_{ac}\cdot\psi_{cv}+\psi_{ev}\cdot\psi_{cv}$ | 0.298199 | 0.52808 | 39893.2 |
| 468 | $\overline{\boldsymbol{u}} \sim \psi_{ce}+\psi_{ev}+\psi_{ac}\cdot\psi_{ev}+\psi_{ac}\cdot\psi_{cv}+\psi_{ce}\cdot\psi_{ev}+\psi_{ev}\cdot\psi_{cv}$ | 0.223616 | 0.5279 | 39906.5 |
| 469 | $\overline{\boldsymbol{u}} \sim \psi_{ev}+\psi_{cv}+\psi_{ac}\cdot\psi_{ce}+\psi_{ac}\cdot\psi_{ev}+\psi_{ce}\cdot\psi_{ev}+\psi_{ce}\cdot\psi_{cv}$ | 0.145976 | 0.52715 | 39954.9 |
| 470 | $\overline{\boldsymbol{u}} \sim \psi_{ce}+\psi_{ev}+\psi_{ac}\cdot\psi_{ev}+\psi_{ac}\cdot\psi_{cv}+\psi_{ev}\cdot\psi_{cv}$ | 0.221032 | 0.52624 | 40012.6 |
| 471 | $\overline{\boldsymbol{u}} \sim \psi_{ac}+\psi_{cv}+\psi_{ac}\cdot\psi_{ce}+\psi_{ac}\cdot\psi_{ev}+\psi_{ce}\cdot\psi_{ev}+\psi_{ev}\cdot\psi_{cv}$ | 0.172474 | 0.52315 | 40211.1 |
| 472 | $\overline{\boldsymbol{u}} \sim \psi_{ac}+\psi_{cv}+\psi_{ac}\cdot\psi_{ce}+\psi_{ac}\cdot\psi_{ev}+\psi_{ce}\cdot\psi_{ev}$ | 0.172194 | 0.5231 | 40213.5 |
| 473 | $\overline{\boldsymbol{u}} \sim \psi_{ce}+\psi_{cv}+\psi_{ac}\cdot\psi_{ce}+\psi_{ac}\cdot\psi_{ev}+\psi_{ce}\cdot\psi_{ev}+\psi_{ev}\cdot\psi_{cv}$ | 0.10427 | 0.52249 | 40253.7 |
| 474 | $\overline{\boldsymbol{u}} \sim \psi_{ac}+\psi_{cv}+\psi_{ac}\cdot\psi_{cv}+\psi_{ce}\cdot\psi_{cv}$ | 0.395921 | 0.52162 | 40307.2 |
| 475 | $\overline{\boldsymbol{u}} \sim \psi_{ce}+\psi_{ac}\cdot\psi_{ev}+\psi_{ac}\cdot\psi_{cv}+\psi_{ce}\cdot\psi_{ev}+\psi_{ev}\cdot\psi_{cv}$ | 0.19041 | 0.52061 | 40372 |
| 476 | $\overline{\boldsymbol{u}} \sim \psi_{cv}+\psi_{ac}\cdot\psi_{ce}+\psi_{ac}\cdot\psi_{cv}+\psi_{ce}\cdot\psi_{cv}$ | 0.189059 | 0.52035 | 40387.7 |
| 477 | $\overline{\boldsymbol{u}} \sim \psi_{ce}+\psi_{cv}+\psi_{ac}\cdot\psi_{cv}+\psi_{ce}\cdot\psi_{cv}$ | 0.218738 | 0.51996 | 40412.6 |
| 478 | $\overline{\boldsymbol{u}} \sim \psi_{ce}+\psi_{cv}+\psi_{ac}\cdot\psi_{cv}$ | 0.220216 | 0.51986 | 40417.7 |
| 479 | $\overline{\boldsymbol{u}} \sim \psi_{ac}+\psi_{ac}\cdot\psi_{ce}+\psi_{ac}\cdot\psi_{ev}+\psi_{ce}\cdot\psi_{ev}+\psi_{ev}\cdot\psi_{cv}$ | 0.16661 | 0.51759 | 40563.1 |
| 480 | $\overline{\boldsymbol{u}} \sim \psi_{ac}+\psi_{ev}+\psi_{ac}\cdot\psi_{ce}+\psi_{ac}\cdot\psi_{ev}+\psi_{ac}\cdot\psi_{cv}+\psi_{ce}\cdot\psi_{ev}$ | 0.142257 | 0.51545 | 40698.9 |
| 481 | $\overline{\boldsymbol{u}} \sim \psi_{ce}+\psi_{ev}+\psi_{ac}\cdot\psi_{ce}+\psi_{ac}\cdot\psi_{cv}+\psi_{ce}\cdot\psi_{ev}+\psi_{ce}\cdot\psi_{cv}+\psi_{ev}\cdot\psi_{cv}$ | 0.108037 | 0.51508 | 40723.5 |
| 482 | $\overline{\boldsymbol{u}} \sim \psi_{ce}+\psi_{ev}+\psi_{ac}\cdot\psi_{ce}+\psi_{ac}\cdot\psi_{cv}+\psi_{ce}\cdot\psi_{cv}+\psi_{ev}\cdot\psi_{cv}$ | 0.108081 | 0.51473 | 40744.4 |

| | | | | |
|---|---|---|---|---|
| 483 | $\overline{\boldsymbol{u}} \sim \psi_{ac}+\psi_{ev}+\psi_{ac}\cdot\psi_{ev}+\psi_{ac}\cdot\psi_{cv}+\psi_{ce}\cdot\psi_{ev}+\psi_{ce}\cdot\psi_{cv}+\psi_{ev}\cdot\psi_{cv}$ | 0.223039 | 0.51428 | 40773.3 |
| 484 | $\overline{\boldsymbol{u}} \sim \psi_{ac}+\psi_{ac}\cdot\psi_{ce}+\psi_{ac}\cdot\psi_{ev}+\psi_{ac}\cdot\psi_{cv}+\psi_{ce}\cdot\psi_{ev}+\psi_{ce}\cdot\psi_{cv}$ | 0.136147 | 0.51411 | 40783.3 |
| 485 | $\overline{\boldsymbol{u}} \sim \psi_{ac}+\psi_{ce}+\psi_{ev}+\psi_{ac}\cdot\psi_{ev}+\psi_{ac}\cdot\psi_{cv}+\psi_{ce}\cdot\psi_{ev}+\psi_{ce}\cdot\psi_{cv}$ | 0.205922 | 0.51344 | 40825.9 |
| 486 | $\overline{\boldsymbol{u}} \sim \psi_{ac}+\psi_{ce}+\psi_{ev}+\psi_{ac}\cdot\psi_{ev}+\psi_{ac}\cdot\psi_{cv}+\psi_{ce}\cdot\psi_{cv}$ | 0.205925 | 0.51342 | 40826.6 |
| 487 | $\overline{\boldsymbol{u}} \sim \psi_{ac}+\psi_{ev}+\psi_{ac}\cdot\psi_{cv}+\psi_{ce}\cdot\psi_{ev}+\psi_{ce}\cdot\psi_{cv}+\psi_{ev}\cdot\psi_{cv}$ | 0.223655 | 0.51332 | 40832.5 |
| 488 | $\overline{\boldsymbol{u}} \sim \psi_{ac}+\psi_{ce}+\psi_{ev}+\psi_{ac}\cdot\psi_{cv}+\psi_{ce}\cdot\psi_{ev}+\psi_{ce}\cdot\psi_{cv}$ | 0.20502 | 0.51324 | 40837.7 |
| 489 | $\overline{\boldsymbol{u}} \sim \psi_{ac}+\psi_{ce}+\psi_{ev}+\psi_{ac}\cdot\psi_{cv}+\psi_{ce}\cdot\psi_{cv}$ | 0.204919 | 0.51317 | 40841.1 |
| 490 | $\overline{\boldsymbol{u}} \sim \psi_{ac}+\psi_{ev}+\psi_{ac}\cdot\psi_{ev}+\psi_{ac}\cdot\psi_{cv}+\psi_{ce}\cdot\psi_{ev}+\psi_{ev}\cdot\psi_{cv}$ | 0.227832 | 0.51309 | 40847.1 |
| 491 | $\overline{\boldsymbol{u}} \sim \psi_{ce}+\psi_{ac}\cdot\psi_{ev}+\psi_{ac}\cdot\psi_{cv}+\psi_{ev}\cdot\psi_{cv}$ | 0.177183 | 0.51242 | 40886.8 |
| 492 | $\overline{\boldsymbol{u}} \sim \psi_{ac}+\psi_{ev}+\psi_{ac}\cdot\psi_{cv}+\psi_{ce}\cdot\psi_{ev}+\psi_{ev}\cdot\psi_{cv}$ | 0.229599 | 0.51177 | 40928.7 |
| 493 | $\overline{\boldsymbol{u}} \sim \psi_{ev}+\psi_{ac}\cdot\psi_{ce}+\psi_{ac}\cdot\psi_{ev}+\psi_{ce}\cdot\psi_{ev}+\psi_{ce}\cdot\psi_{cv}+\psi_{ev}\cdot\psi_{cv}$ | 0.140898 | 0.51162 | 40938.7 |
| 494 | $\overline{\boldsymbol{u}} \sim \psi_{ac}+\psi_{ac}\cdot\psi_{ev}+\psi_{ac}\cdot\psi_{cv}+\psi_{ce}\cdot\psi_{ev}+\psi_{ce}\cdot\psi_{cv}+\psi_{ev}\cdot\psi_{cv}$ | 0.221092 | 0.51108 | 40972.4 |
| 495 | $\overline{\boldsymbol{u}} \sim \psi_{ac}+\psi_{ev}+\psi_{ac}\cdot\psi_{ce}+\psi_{ac}\cdot\psi_{cv}+\psi_{ce}\cdot\psi_{ev}$ | 0.140397 | 0.51098 | 40977.5 |
| 496 | $\overline{\boldsymbol{u}} \sim \psi_{ac}+\psi_{ev}+\psi_{ac}\cdot\psi_{ce}+\psi_{ac}\cdot\psi_{ev}+\psi_{ce}\cdot\psi_{ev}$ | 0.144712 | 0.51059 | 41001.8 |
| 497 | $\overline{\boldsymbol{u}} \sim \psi_{ac}+\psi_{ev}+\psi_{ac}\cdot\psi_{ce}+\psi_{ac}\cdot\psi_{ev}+\psi_{ce}\cdot\psi_{ev}+\psi_{ce}\cdot\psi_{cv}$ | 0.144589 | 0.51059 | 41002.9 |
| 498 | $\overline{\boldsymbol{u}} \sim \psi_{ac}+\psi_{ac}\cdot\psi_{ev}+\psi_{ac}\cdot\psi_{cv}+\psi_{ce}\cdot\psi_{ev}+\psi_{ev}\cdot\psi_{cv}$ | 0.224058 | 0.51044 | 41011.1 |
| 499 | $\overline{\boldsymbol{u}} \sim \psi_{cv}+\psi_{ac}\cdot\psi_{cv}+\psi_{ce}\cdot\psi_{cv}$ | 0.225127 | 0.5104 | 41011.7 |
| 500 | $\overline{\boldsymbol{u}} \sim \psi_{ac}+\psi_{ac}\cdot\psi_{cv}+\psi_{ce}\cdot\psi_{ev}+\psi_{ce}\cdot\psi_{cv}+\psi_{ev}\cdot\psi_{cv}$ | 0.218231 | 0.51038 | 41015.1 |
| 501 | $\overline{\boldsymbol{u}} \sim \psi_{ac}+\psi_{ac}\cdot\psi_{cv}+\psi_{ce}\cdot\psi_{ev}+\psi_{ev}\cdot\psi_{cv}$ | 0.220721 | 0.5102 | 41025.3 |
| 502 | $\overline{\boldsymbol{u}} \sim \psi_{ac}+\psi_{ce}+\psi_{ac}\cdot\psi_{ev}+\psi_{ac}\cdot\psi_{cv}+\psi_{ce}\cdot\psi_{ev}+\psi_{ce}\cdot\psi_{cv}$ | 0.204388 | 0.51007 | 41035.6 |
| 503 | $\overline{\boldsymbol{u}} \sim \psi_{ac}+\psi_{ce}+\psi_{ac}\cdot\psi_{ev}+\psi_{ac}\cdot\psi_{cv}+\psi_{ce}\cdot\psi_{cv}$ | 0.204935 | 0.50966 | 41059.7 |
| 504 | $\overline{\boldsymbol{u}} \sim \psi_{ac}+\psi_{ev}+\psi_{ac}\cdot\psi_{ce}+\psi_{ce}\cdot\psi_{ev}$ | 0.142812 | 0.50598 | 41286.2 |
| 505 | $\overline{\boldsymbol{u}} \sim \psi_{ac}+\psi_{ev}+\psi_{ac}\cdot\psi_{ce}+\psi_{ce}\cdot\psi_{ev}+\psi_{ce}\cdot\psi_{cv}$ | 0.142701 | 0.50598 | 41287.5 |
| 506 | $\overline{\boldsymbol{u}} \sim \psi_{ev}+\psi_{ac}\cdot\psi_{ev}+\psi_{ac}\cdot\psi_{cv}+\psi_{ce}\cdot\psi_{ev}+\psi_{ce}\cdot\psi_{cv}+\psi_{ev}\cdot\psi_{cv}$ | 0.193402 | 0.50525 | 41333.2 |
| 507 | $\overline{\boldsymbol{u}} \sim \psi_{ce}+\psi_{ac}\cdot\psi_{ce}+\psi_{ac}\cdot\psi_{cv}+\psi_{ce}\cdot\psi_{ev}+\psi_{ce}\cdot\psi_{cv}+\psi_{ev}\cdot\psi_{cv}$ | 0.107302 | 0.50522 | 41335.4 |
| 508 | $\overline{\boldsymbol{u}} \sim \psi_{ac}+\psi_{ce}+\psi_{ev}+\psi_{cv}+\psi_{ac}\cdot\psi_{ev}+\psi_{ce}\cdot\psi_{ev}+\psi_{ev}\cdot\psi_{cv}$ | 0.272375 | 0.50513 | 41341.6 |
| 509 | $\overline{\boldsymbol{u}} \sim \psi_{ac}+\psi_{ce}+\psi_{ev}+\psi_{cv}+\psi_{ac}\cdot\psi_{ev}+\psi_{ce}\cdot\psi_{ev}$ | 0.272107 | 0.50512 | 41341.4 |

| | | | | |
|---|---|---|---|---|
| 510 | $\overline{\boldsymbol{u}} \sim \psi_{ac}+\psi_{ce}+\psi_{ev}+\psi_{cv}+\psi_{ac}\cdot\psi_{ev}+\psi_{ce}\cdot\psi_{ev}+\psi_{ce}\cdot\psi_{cv}+\psi_{ev}\cdot\psi_{cv}$ | 0.271591 | 0.50512 | 41343.4 |
| 511 | $\overline{\boldsymbol{u}} \sim \psi_{ac}+\psi_{ce}+\psi_{ev}+\psi_{cv}+\psi_{ac}\cdot\psi_{ev}+\psi_{ev}\cdot\psi_{cv}$ | 0.272414 | 0.50511 | 41342.1 |
| 512 | $\overline{\boldsymbol{u}} \sim \psi_{ac}+\psi_{ce}+\psi_{ev}+\psi_{cv}+\psi_{ac}\cdot\psi_{ev}+\psi_{ce}\cdot\psi_{ev}+\psi_{ce}\cdot\psi_{cv}$ | 0.271319 | 0.50511 | 41343.1 |
| 513 | $\overline{\boldsymbol{u}} \sim \psi_{ac}+\psi_{ce}+\psi_{ev}+\psi_{cv}+\psi_{ac}\cdot\psi_{ev}+\psi_{ce}\cdot\psi_{cv}+\psi_{ev}\cdot\psi_{cv}$ | 0.271628 | 0.5051 | 41343.9 |
| 514 | $\overline{\boldsymbol{u}} \sim \psi_{ac}+\psi_{ce}+\psi_{ev}+\psi_{cv}+\psi_{ac}\cdot\psi_{ev}$ | 0.272122 | 0.50509 | 41342.4 |
| 515 | $\overline{\boldsymbol{u}} \sim \psi_{ac}+\psi_{ce}+\psi_{ev}+\psi_{cv}+\psi_{ac}\cdot\psi_{ev}+\psi_{ce}\cdot\psi_{cv}$ | 0.271332 | 0.50508 | 41344.1 |
| 516 | $\overline{\boldsymbol{u}} \sim \psi_{ac}+\psi_{ce}+\psi_{ev}+\psi_{cv}+\psi_{ce}\cdot\psi_{ev}$ | 0.269599 | 0.50482 | 41358.7 |
| 517 | $\overline{\boldsymbol{u}} \sim \psi_{ac}+\psi_{ce}+\psi_{ev}+\psi_{cv}+\psi_{ce}\cdot\psi_{ev}+\psi_{ce}\cdot\psi_{cv}$ | 0.268766 | 0.50481 | 41360.4 |
| 518 | $\overline{\boldsymbol{u}} \sim \psi_{ac}+\psi_{ce}+\psi_{ev}+\psi_{cv}+\psi_{ce}\cdot\psi_{ev}+\psi_{ev}\cdot\psi_{cv}$ | 0.269593 | 0.50481 | 41360.6 |
| 519 | $\overline{\boldsymbol{u}} \sim \psi_{ac}+\psi_{ce}+\psi_{ev}+\psi_{cv}+\psi_{ce}\cdot\psi_{ev}+\psi_{ce}\cdot\psi_{cv}+\psi_{ev}\cdot\psi_{cv}$ | 0.26876 | 0.5048 | 41362.3 |
| 520 | $\overline{\boldsymbol{u}} \sim \psi_{ac}+\psi_{ce}+\psi_{ev}+\psi_{cv}$ | 0.269351 | 0.50473 | 41363.2 |
| 521 | $\overline{\boldsymbol{u}} \sim \psi_{ac}+\psi_{ce}+\psi_{ev}+\psi_{cv}+\psi_{ce}\cdot\psi_{cv}$ | 0.26851 | 0.50472 | 41364.9 |
| 522 | $\overline{\boldsymbol{u}} \sim \psi_{ac}+\psi_{ce}+\psi_{ev}+\psi_{cv}+\psi_{ev}\cdot\psi_{cv}$ | 0.269347 | 0.50472 | 41365.2 |
| 523 | $\overline{\boldsymbol{u}} \sim \psi_{ac}+\psi_{ce}+\psi_{ev}+\psi_{cv}+\psi_{ce}\cdot\psi_{cv}+\psi_{ev}\cdot\psi_{cv}$ | 0.268506 | 0.50471 | 41366.9 |
| 524 | $\overline{\boldsymbol{u}} \sim \psi_{ev}+\psi_{ac}\cdot\psi_{ev}+\psi_{ac}\cdot\psi_{cv}+\psi_{ce}\cdot\psi_{ev}+\psi_{ev}\cdot\psi_{cv}$ | 0.196491 | 0.50458 | 41373.5 |
| 525 | $\overline{\boldsymbol{u}} \sim \psi_{ac}+\psi_{ce}+\psi_{cv}+\psi_{ac}\cdot\psi_{ev}+\psi_{ce}\cdot\psi_{ev}+\psi_{ev}\cdot\psi_{cv}$ | 0.257316 | 0.50181 | 41544.2 |
| 526 | $\overline{\boldsymbol{u}} \sim \psi_{ac}+\psi_{ce}+\psi_{cv}+\psi_{ac}\cdot\psi_{ev}+\psi_{ce}\cdot\psi_{ev}$ | 0.257571 | 0.5018 | 41543.9 |
| 527 | $\overline{\boldsymbol{u}} \sim \psi_{ac}+\psi_{ce}+\psi_{cv}+\psi_{ac}\cdot\psi_{ev}+\psi_{ce}\cdot\psi_{ev}+\psi_{ce}\cdot\psi_{cv}+\psi_{ev}\cdot\psi_{cv}$ | 0.256574 | 0.5018 | 41545.7 |
| 528 | $\overline{\boldsymbol{u}} \sim \psi_{ac}+\psi_{ce}+\psi_{cv}+\psi_{ac}\cdot\psi_{ev}+\psi_{ce}\cdot\psi_{ev}+\psi_{ce}\cdot\psi_{cv}$ | 0.256835 | 0.50179 | 41545.4 |
| 529 | $\overline{\boldsymbol{u}} \sim \psi_{ac}+\psi_{ce}+\psi_{cv}+\psi_{ac}\cdot\psi_{ev}$ | 0.258104 | 0.50141 | 41566.6 |
| 530 | $\overline{\boldsymbol{u}} \sim \psi_{ac}+\psi_{ce}+\psi_{cv}+\psi_{ac}\cdot\psi_{ev}+\psi_{ev}\cdot\psi_{cv}$ | 0.257905 | 0.50141 | 41567.7 |
| 531 | $\overline{\boldsymbol{u}} \sim \psi_{ac}+\psi_{ce}+\psi_{cv}+\psi_{ac}\cdot\psi_{ev}+\psi_{ce}\cdot\psi_{cv}$ | 0.25738 | 0.5014 | 41568.2 |
| 532 | $\overline{\boldsymbol{u}} \sim \psi_{ac}+\psi_{ce}+\psi_{cv}+\psi_{ac}\cdot\psi_{ev}+\psi_{ce}\cdot\psi_{cv}+\psi_{ev}\cdot\psi_{cv}$ | 0.257177 | 0.5014 | 41569.3 |
| 533 | $\overline{\boldsymbol{u}} \sim \psi_{ac}+\psi_{ce}+\psi_{ev}+\psi_{ce}\cdot\psi_{ev}+\psi_{ce}\cdot\psi_{cv}+\psi_{ev}\cdot\psi_{cv}$ | 0.237424 | 0.50084 | 41603.5 |
| 534 | $\overline{\boldsymbol{u}} \sim \psi_{ac}+\psi_{ce}+\psi_{ev}+\psi_{ac}\cdot\psi_{ev}+\psi_{ce}\cdot\psi_{ev}+\psi_{ce}\cdot\psi_{cv}+\psi_{ev}\cdot\psi_{cv}$ | 0.237451 | 0.50083 | 41605.4 |
| 535 | $\overline{\boldsymbol{u}} \sim \psi_{ce}+\psi_{ac}\cdot\psi_{ce}+\psi_{ac}\cdot\psi_{cv}+\psi_{ce}\cdot\psi_{cv}+\psi_{ev}\cdot\psi_{cv}$ | 0.107304 | 0.50078 | 41606.4 |
| 536 | $\overline{\boldsymbol{u}} \sim \psi_{ac}+\psi_{ce}+\psi_{ev}+\psi_{ce}\cdot\psi_{cv}+\psi_{ev}\cdot\psi_{cv}$ | 0.237109 | 0.50071 | 41610.4 |

| | | | | |
|---|---|---|---|---|
| 537 | $\overline{\boldsymbol{u}} \sim \psi_{ac}+\psi_{ce}+\psi_{ev}+\psi_{ac}\cdot\psi_{ev}+\psi_{ce}\cdot\psi_{cv}+\psi_{ev}\cdot\psi_{cv}$ | 0.237211 | 0.50071 | 41611.8 |
| 538 | $\overline{\boldsymbol{u}} \sim \psi_{ac}+\psi_{ce}+\psi_{ac}\cdot\psi_{ev}+\psi_{ce}\cdot\psi_{ev}+\psi_{ce}\cdot\psi_{cv}+\psi_{ev}\cdot\psi_{cv}$ | 0.233103 | 0.49854 | 41743.7 |
| 539 | $\overline{\boldsymbol{u}} \sim \psi_{ac}+\psi_{ce}+\psi_{ev}+\psi_{ac}\cdot\psi_{ev}+\psi_{ce}\cdot\psi_{ev}+\psi_{ev}\cdot\psi_{cv}$ | 0.242121 | 0.49853 | 41744 |
| 540 | $\overline{\boldsymbol{u}} \sim \psi_{ac}+\psi_{ce}+\psi_{ev}+\psi_{ce}\cdot\psi_{ev}+\psi_{ev}\cdot\psi_{cv}$ | 0.2426 | 0.49849 | 41745.7 |
| 541 | $\overline{\boldsymbol{u}} \sim \psi_{ac}+\psi_{ce}+\psi_{ev}+\psi_{ac}\cdot\psi_{ev}+\psi_{ev}\cdot\psi_{cv}$ | 0.242005 | 0.49834 | 41754.7 |
| 542 | $\overline{\boldsymbol{u}} \sim \psi_{ac}+\psi_{ce}+\psi_{ev}+\psi_{ev}\cdot\psi_{cv}$ | 0.242345 | 0.49833 | 41754.3 |
| 543 | $\overline{\boldsymbol{u}} \sim \psi_{ac}+\psi_{ce}+\psi_{ac}\cdot\psi_{ev}+\psi_{ce}\cdot\psi_{cv}+\psi_{ev}\cdot\psi_{cv}$ | 0.233317 | 0.49801 | 41774.8 |
| 544 | $\overline{\boldsymbol{u}} \sim \psi_{ac}+\psi_{cv}+\psi_{ac}\cdot\psi_{ce}+\psi_{ac}\cdot\psi_{cv}$ | 0.377282 | 0.49716 | 41825 |
| 545 | $\overline{\boldsymbol{u}} \sim \psi_{ac}+\psi_{ce}+\psi_{ac}\cdot\psi_{ev}+\psi_{ce}\cdot\psi_{ev}+\psi_{ev}\cdot\psi_{cv}$ | 0.236654 | 0.49667 | 41856.1 |
| 546 | $\overline{\boldsymbol{u}} \sim \psi_{ac}+\psi_{ac}\cdot\psi_{ce}+\psi_{ac}\cdot\psi_{ev}+\psi_{ac}\cdot\psi_{cv}+\psi_{ce}\cdot\psi_{ev}$ | 0.158935 | 0.49652 | 41865.2 |
| 547 | $\overline{\boldsymbol{u}} \sim \psi_{ac}+\psi_{ce}+\psi_{ev}+\psi_{ac}\cdot\psi_{ev}+\psi_{ce}\cdot\psi_{ev}+\psi_{ce}\cdot\psi_{cv}$ | 0.215847 | 0.49622 | 41884 |
| 548 | $\overline{\boldsymbol{u}} \sim \psi_{ac}+\psi_{ce}+\psi_{ev}+\psi_{ac}\cdot\psi_{ev}+\psi_{ce}\cdot\psi_{cv}$ | 0.215835 | 0.49619 | 41885.1 |
| 549 | $\overline{\boldsymbol{u}} \sim \psi_{ac}+\psi_{ce}+\psi_{ac}\cdot\psi_{ev}+\psi_{ev}\cdot\psi_{cv}$ | 0.23685 | 0.49607 | 41891.3 |
| 550 | $\overline{\boldsymbol{u}} \sim \psi_{ac}+\psi_{ce}+\psi_{ev}+\psi_{ce}\cdot\psi_{ev}+\psi_{ce}\cdot\psi_{cv}$ | 0.214281 | 0.49594 | 41900 |
| 551 | $\overline{\boldsymbol{u}} \sim \psi_{ac}+\psi_{ce}+\psi_{ev}+\psi_{ce}\cdot\psi_{cv}$ | 0.214088 | 0.49585 | 41904.5 |
| 552 | $\overline{\boldsymbol{u}} \sim \psi_{ac}+\psi_{ev}+\psi_{cv}+\psi_{ac}\cdot\psi_{ev}+\psi_{ce}\cdot\psi_{ev}+\psi_{ce}\cdot\psi_{cv}+\psi_{ev}\cdot\psi_{cv}$ | 0.244202 | 0.49516 | 41948.9 |
| 553 | $\overline{\boldsymbol{u}} \sim \psi_{ac}+\psi_{ev}+\psi_{cv}+\psi_{ac}\cdot\psi_{ev}+\psi_{ce}\cdot\psi_{ev}+\psi_{ce}\cdot\psi_{cv}$ | 0.243798 | 0.49502 | 41956.5 |
| 554 | $\overline{\boldsymbol{u}} \sim \psi_{ce}+\psi_{ac}\cdot\psi_{ce}+\psi_{ac}\cdot\psi_{ev}+\psi_{ce}\cdot\psi_{ev}+\psi_{ce}\cdot\psi_{cv}+\psi_{ev}\cdot\psi_{cv}$ | 0.0931784 | 0.49486 | 41965.9 |
| 555 | $\overline{\boldsymbol{u}} \sim \psi_{ev}+\psi_{cv}+\psi_{ac}\cdot\psi_{ce}+\psi_{ac}\cdot\psi_{ev}+\psi_{ce}\cdot\psi_{cv}+\psi_{ev}\cdot\psi_{cv}$ | 0.153915 | 0.49427 | 42001.8 |
| 556 | $\overline{\boldsymbol{u}} \sim \psi_{ac}+\psi_{ev}+\psi_{cv}+\psi_{ce}\cdot\psi_{ev}+\psi_{ce}\cdot\psi_{cv}$ | 0.240629 | 0.49412 | 42009.5 |
| 557 | $\overline{\boldsymbol{u}} \sim \psi_{ac}+\psi_{ev}+\psi_{cv}+\psi_{ce}\cdot\psi_{ev}+\psi_{ce}\cdot\psi_{cv}+\psi_{ev}\cdot\psi_{cv}$ | 0.240632 | 0.49411 | 42011.3 |
| 558 | $\overline{\boldsymbol{u}} \sim \psi_{ac}+\psi_{ce}+\psi_{ac}\cdot\psi_{ev}+\psi_{ce}\cdot\psi_{ev}+\psi_{ce}\cdot\psi_{cv}$ | 0.213693 | 0.49311 | 42070.4 |
| 559 | $\overline{\boldsymbol{u}} \sim \psi_{ac}+\psi_{ce}+\psi_{ce}\cdot\psi_{ev}+\psi_{ce}\cdot\psi_{cv}+\psi_{ev}\cdot\psi_{cv}$ | 0.215505 | 0.49287 | 42084.6 |
| 560 | $\overline{\boldsymbol{u}} \sim \psi_{ac}+\psi_{ce}+\psi_{cv}+\psi_{ce}\cdot\psi_{ev}+\psi_{ce}\cdot\psi_{cv}+\psi_{ev}\cdot\psi_{cv}$ | 0.215607 | 0.49286 | 42086.5 |
| 561 | $\overline{\boldsymbol{u}} \sim \psi_{ac}+\psi_{ce}+\psi_{cv}+\psi_{ce}\cdot\psi_{ev}+\psi_{ev}\cdot\psi_{cv}$ | 0.216513 | 0.49285 | 42085.9 |
| 562 | $\overline{\boldsymbol{u}} \sim \psi_{ac}+\psi_{ce}+\psi_{ce}\cdot\psi_{ev}+\psi_{ev}\cdot\psi_{cv}$ | 0.216741 | 0.49283 | 42086 |
| 563 | $\overline{\boldsymbol{u}} \sim \psi_{ac}+\psi_{ce}+\psi_{ac}\cdot\psi_{ev}+\psi_{ce}\cdot\psi_{cv}$ | 0.214307 | 0.49271 | 42093.5 |

| | | | | |
|---|---|---|---|---|
| 564 | $\overline{\boldsymbol{u}} \sim \psi_{ce}+\psi_{ev}+\psi_{ac}\cdot\psi_{ce}+\psi_{ac}\cdot\psi_{cv}+\psi_{ce}\cdot\psi_{ev}+\psi_{ev}\cdot\psi_{cv}$ | 0.104722 | 0.49257 | 42103.9 |
| 565 | $\overline{\boldsymbol{u}} \sim \psi_{ce}+\psi_{ev}+\psi_{ac}\cdot\psi_{ce}+\psi_{ac}\cdot\psi_{cv}+\psi_{ev}\cdot\psi_{cv}$ | 0.104793 | 0.49211 | 42130.4 |
| 566 | $\overline{\boldsymbol{u}} \sim \psi_{ac}+\psi_{ac}\cdot\psi_{ce}+\psi_{ac}\cdot\psi_{ev}+\psi_{ce}\cdot\psi_{ev}$ | 0.16339 | 0.49154 | 42163.8 |
| 567 | $\overline{\boldsymbol{u}} \sim \psi_{ac}+\psi_{ac}\cdot\psi_{ce}+\psi_{ac}\cdot\psi_{ev}+\psi_{ce}\cdot\psi_{ev}+\psi_{ce}\cdot\psi_{cv}$ | 0.163408 | 0.49152 | 42165.6 |
| 568 | $\overline{\boldsymbol{u}} \sim \psi_{ce}+\psi_{ev}+\psi_{ac}\cdot\psi_{cv}+\psi_{ce}\cdot\psi_{ev}+\psi_{ce}\cdot\psi_{cv}+\psi_{ev}\cdot\psi_{cv}$ | 0.130599 | 0.49076 | 42212.2 |
| 569 | $\overline{\boldsymbol{u}} \sim \psi_{cv}+\psi_{ac}\cdot\psi_{ce}+\psi_{ac}\cdot\psi_{cv}$ | 0.239638 | 0.49057 | 42220.9 |
| 570 | $\overline{\boldsymbol{u}} \sim \psi_{ce}+\psi_{ev}+\psi_{ac}\cdot\psi_{cv}+\psi_{ce}\cdot\psi_{cv}+\psi_{ev}\cdot\psi_{cv}$ | 0.130556 | 0.49041 | 42232 |
| 571 | $\overline{\boldsymbol{u}} \sim \psi_{ac}+\psi_{ev}+\psi_{ac}\cdot\psi_{ce}+\psi_{ac}\cdot\psi_{ev}+\psi_{ac}\cdot\psi_{cv}+\psi_{ce}\cdot\psi_{cv}+\psi_{ev}\cdot\psi_{cv}$ | 0.193899 | 0.49037 | 42236.8 |
| 572 | $\overline{\boldsymbol{u}} \sim \psi_{ac}+\psi_{ac}\cdot\psi_{ce}+\psi_{ac}\cdot\psi_{ev}+\psi_{ac}\cdot\psi_{cv}+\psi_{ce}\cdot\psi_{cv}+\psi_{ev}\cdot\psi_{cv}$ | 0.192939 | 0.49 | 42257.7 |
| 573 | $\overline{\boldsymbol{u}} \sim \psi_{ac}+\psi_{cv}+\psi_{ac}\cdot\psi_{ev}+\psi_{ce}\cdot\psi_{ev}+\psi_{ce}\cdot\psi_{cv}$ | 0.239178 | 0.48971 | 42274.1 |
| 574 | $\overline{\boldsymbol{u}} \sim \psi_{ac}+\psi_{cv}+\psi_{ac}\cdot\psi_{ev}+\psi_{ce}\cdot\psi_{ev}+\psi_{ce}\cdot\psi_{cv}+\psi_{ev}\cdot\psi_{cv}$ | 0.23916 | 0.48969 | 42276.1 |
| 575 | $\overline{\boldsymbol{u}} \sim \psi_{ev}+\psi_{cv}+\psi_{ac}\cdot\psi_{ce}+\psi_{ac}\cdot\psi_{ev}+\psi_{ce}\cdot\psi_{cv}$ | 0.15514 | 0.4888 | 42327.9 |
| 576 | $\overline{\boldsymbol{u}} \sim \psi_{ac}+\psi_{ev}+\psi_{cv}+\psi_{ac}\cdot\psi_{ev}+\psi_{ce}\cdot\psi_{ev}+\psi_{ev}\cdot\psi_{cv}$ | 0.217175 | 0.4887 | 42335.5 |
| 577 | $\overline{\boldsymbol{u}} \sim \psi_{ac}+\psi_{ev}+\psi_{cv}+\psi_{ac}\cdot\psi_{ev}+\psi_{ce}\cdot\psi_{ev}$ | 0.216585 | 0.4884 | 42351.9 |
| 578 | $\overline{\boldsymbol{u}} \sim \psi_{ac}+\psi_{ce}+\psi_{ac}\cdot\psi_{cv}+\psi_{ce}\cdot\psi_{ev}+\psi_{ce}\cdot\psi_{cv}$ | 0.17257 | 0.48796 | 42378.4 |
| 579 | $\overline{\boldsymbol{u}} \sim \psi_{ac}+\psi_{ev}+\psi_{cv}+\psi_{ac}\cdot\psi_{ev}+\psi_{ce}\cdot\psi_{cv}+\psi_{ev}\cdot\psi_{cv}$ | 0.23561 | 0.48744 | 42409.8 |
| 580 | $\overline{\boldsymbol{u}} \sim \psi_{ac}+\psi_{ev}+\psi_{cv}+\psi_{ac}\cdot\psi_{ev}+\psi_{ce}\cdot\psi_{cv}$ | 0.235461 | 0.48742 | 42410 |
| 581 | $\overline{\boldsymbol{u}} \sim \psi_{ac}+\psi_{cv}+\psi_{ac}\cdot\psi_{cv}+\psi_{ce}\cdot\psi_{ev}$ | 0.461675 | 0.4874 | 42410.3 |
| 582 | $\overline{\boldsymbol{u}} \sim \psi_{ac}+\psi_{ev}+\psi_{cv}+\psi_{ce}\cdot\psi_{cv}$ | 0.234087 | 0.48706 | 42430.4 |
| 583 | $\overline{\boldsymbol{u}} \sim \psi_{ac}+\psi_{ev}+\psi_{cv}+\psi_{ce}\cdot\psi_{cv}+\psi_{ev}\cdot\psi_{cv}$ | 0.234085 | 0.48705 | 42432.4 |
| 584 | $\overline{\boldsymbol{u}} \sim \psi_{ac}+\psi_{ev}+\psi_{cv}+\psi_{ce}\cdot\psi_{ev}$ | 0.212436 | 0.48693 | 42438.2 |
| 585 | $\overline{\boldsymbol{u}} \sim \psi_{ac}+\psi_{ev}+\psi_{cv}+\psi_{ce}\cdot\psi_{ev}+\psi_{ev}\cdot\psi_{cv}$ | 0.212444 | 0.48693 | 42439.4 |
| 586 | $\overline{\boldsymbol{u}} \sim \psi_{ce}+\psi_{ev}+\psi_{ac}\cdot\psi_{ev}+\psi_{ac}\cdot\psi_{cv}+\psi_{ce}\cdot\psi_{ev}+\psi_{ce}\cdot\psi_{cv}$ | 0.162079 | 0.48605 | 42492.5 |
| 587 | $\overline{\boldsymbol{u}} \sim \psi_{ac}+\psi_{cv}+\psi_{ac}\cdot\psi_{cv}$ | 0.595634 | 0.48593 | 42496.9 |
| 588 | $\overline{\boldsymbol{u}} \sim \psi_{ac}+\psi_{ev}+\psi_{ac}\cdot\psi_{ce}+\psi_{ac}\cdot\psi_{cv}+\psi_{ce}\cdot\psi_{cv}+\psi_{ev}\cdot\psi_{cv}$ | 0.196126 | 0.48568 | 42514.2 |
| 589 | $\overline{\boldsymbol{u}} \sim \psi_{ac}\cdot\psi_{ce}+\psi_{ac}\cdot\psi_{ev}+\psi_{ac}\cdot\psi_{cv}+\psi_{ce}\cdot\psi_{ev}+\psi_{ce}\cdot\psi_{cv}+\psi_{ev}\cdot\psi_{cv}$ | 0.147808 | 0.48543 | 42529.4 |
| 590 | $\overline{\boldsymbol{u}} \sim \psi_{ce}+\psi_{ev}+\psi_{ac}\cdot\psi_{ev}+\psi_{ac}\cdot\psi_{cv}+\psi_{ce}\cdot\psi_{cv}$ | 0.160954 | 0.48495 | 42556.7 |

| | | | | |
|---|---|---|---|---|
| 591 | $\overline{\boldsymbol{u}} \sim \psi_{ac}+\psi_{ev}+\psi_{ac}\cdot\psi_{ce}+\psi_{ac}\cdot\psi_{ev}+\psi_{ce}\cdot\psi_{cv}+\psi_{ev}\cdot\psi_{cv}$ | 0.17475 | 0.4844 | 42589.9 |
| 592 | $\overline{\boldsymbol{u}} \sim \psi_{ce}+\psi_{ac}\cdot\psi_{cv}+\psi_{ce}\cdot\psi_{ev}+\psi_{ce}\cdot\psi_{cv}+\psi_{ev}\cdot\psi_{cv}$ | 0.127908 | 0.48414 | 42604.5 |
| 593 | $\overline{\boldsymbol{u}} \sim \psi_{ac}+\psi_{cv}+\psi_{ac}\cdot\psi_{ev}+\psi_{ce}\cdot\psi_{cv}$ | 0.231989 | 0.48394 | 42615.2 |
| 594 | $\overline{\boldsymbol{u}} \sim \psi_{ac}+\psi_{cv}+\psi_{ac}\cdot\psi_{ev}+\psi_{ce}\cdot\psi_{cv}+\psi_{ev}\cdot\psi_{cv}$ | 0.231855 | 0.48394 | 42616.4 |
| 595 | $\overline{\boldsymbol{u}} \sim \psi_{ac}+\psi_{ac}\cdot\psi_{ce}+\psi_{ac}\cdot\psi_{ev}+\psi_{ce}\cdot\psi_{cv}+\psi_{ev}\cdot\psi_{cv}$ | 0.173828 | 0.48376 | 42626.7 |
| 596 | $\overline{\boldsymbol{u}} \sim \psi_{ac}+\psi_{ce}+\psi_{cv}+\psi_{ce}\cdot\psi_{cv}+\psi_{ev}\cdot\psi_{cv}$ | 0.201574 | 0.48332 | 42653.2 |
| 597 | $\overline{\boldsymbol{u}} \sim \psi_{ac}+\psi_{ce}+\psi_{cv}+\psi_{ev}\cdot\psi_{cv}$ | 0.202496 | 0.4833 | 42653 |
| 598 | $\overline{\boldsymbol{u}} \sim \psi_{ac}+\psi_{ev}+\psi_{ac}\cdot\psi_{ev}+\psi_{ac}\cdot\psi_{cv}+\psi_{ce}\cdot\psi_{cv}+\psi_{ev}\cdot\psi_{cv}$ | 0.215845 | 0.48313 | 42665 |
| 599 | $\overline{\boldsymbol{u}} \sim \psi_{ac}+\psi_{ac}\cdot\psi_{ev}+\psi_{ac}\cdot\psi_{cv}+\psi_{ce}\cdot\psi_{cv}+\psi_{ev}\cdot\psi_{cv}$ | 0.214484 | 0.48273 | 42687.7 |
| 600 | $\overline{\boldsymbol{u}} \sim \psi_{ev}+\psi_{ac}\cdot\psi_{ce}+\psi_{ac}\cdot\psi_{ev}+\psi_{ac}\cdot\psi_{cv}+\psi_{ce}\cdot\psi_{cv}+\psi_{ev}\cdot\psi_{cv}$ | 0.183096 | 0.48169 | 42749.9 |
| 601 | $\overline{\boldsymbol{u}} \sim \psi_{ce}+\psi_{ac}\cdot\psi_{cv}+\psi_{ce}\cdot\psi_{cv}+\psi_{ev}\cdot\psi_{cv}$ | 0.127057 | 0.48161 | 42752.6 |
| 602 | $\overline{\boldsymbol{u}} \sim \psi_{ac}+\psi_{ev}+\psi_{ac}\cdot\psi_{ce}+\psi_{ce}\cdot\psi_{cv}+\psi_{ev}\cdot\psi_{cv}$ | 0.179001 | 0.48139 | 42766.2 |
| 603 | $\overline{\boldsymbol{u}} \sim \psi_{ac}+\psi_{cv}+\psi_{ac}\cdot\psi_{ev}+\psi_{ce}\cdot\psi_{ev}$ | 0.216325 | 0.48122 | 42775.6 |
| 604 | $\overline{\boldsymbol{u}} \sim \psi_{ac}+\psi_{cv}+\psi_{ac}\cdot\psi_{ev}+\psi_{ce}\cdot\psi_{ev}+\psi_{ev}\cdot\psi_{cv}$ | 0.216493 | 0.48121 | 42777.1 |
| 605 | $\overline{\boldsymbol{u}} \sim \psi_{ac}+\psi_{ev}+\psi_{ac}\cdot\psi_{ev}+\psi_{ce}\cdot\psi_{ev}+\psi_{ce}\cdot\psi_{cv}+\psi_{ev}\cdot\psi_{cv}$ | 0.209937 | 0.48118 | 42779.5 |
| 606 | $\overline{\boldsymbol{u}} \sim \psi_{ac}+\psi_{ev}+\psi_{ce}\cdot\psi_{ev}+\psi_{ce}\cdot\psi_{cv}+\psi_{ev}\cdot\psi_{cv}$ | 0.209672 | 0.48117 | 42779.4 |
| 607 | $\overline{\boldsymbol{u}} \sim \psi_{ac}+\psi_{ce}+\psi_{ce}\cdot\psi_{cv}+\psi_{ev}\cdot\psi_{cv}$ | 0.206285 | 0.48048 | 42818.8 |
| 608 | $\overline{\boldsymbol{u}} \sim \psi_{ac}+\psi_{ev}+\psi_{ac}\cdot\psi_{ev}+\psi_{ce}\cdot\psi_{ev}+\psi_{ev}\cdot\psi_{cv}$ | 0.204151 | 0.48005 | 42844.9 |
| 609 | $\overline{\boldsymbol{u}} \sim \psi_{ac}\cdot\psi_{ce}+\psi_{ac}\cdot\psi_{ev}+\psi_{ac}\cdot\psi_{cv}+\psi_{ce}\cdot\psi_{ev}+\psi_{ev}\cdot\psi_{cv}$ | 0.15198 | 0.4799 | 42853.8 |
| 610 | $\overline{\boldsymbol{u}} \sim \psi_{ac}+\psi_{ev}+\psi_{ce}\cdot\psi_{ev}+\psi_{ev}\cdot\psi_{cv}$ | 0.20299 | 0.47986 | 42854.8 |
| 611 | $\overline{\boldsymbol{u}} \sim \psi_{ac}\cdot\psi_{ev}+\psi_{ac}\cdot\psi_{cv}+\psi_{ce}\cdot\psi_{ev}+\psi_{ce}\cdot\psi_{cv}+\psi_{ev}\cdot\psi_{cv}$ | 0.161294 | 0.4795 | 42876.9 |
| 612 | $\overline{\boldsymbol{u}} \sim \psi_{ac}+\psi_{ev}+\psi_{ac}\cdot\psi_{cv}+\psi_{ce}\cdot\psi_{cv}+\psi_{ev}\cdot\psi_{cv}$ | 0.217409 | 0.47908 | 42902 |
| 613 | $\overline{\boldsymbol{u}} \sim \psi_{ac}\cdot\psi_{ev}+\psi_{ac}\cdot\psi_{cv}+\psi_{ce}\cdot\psi_{ev}+\psi_{ev}\cdot\psi_{cv}$ | 0.158346 | 0.47877 | 42918.7 |
| 614 | $\overline{\boldsymbol{u}} \sim \psi_{ac}+\psi_{ce}+\psi_{cv}+\psi_{ce}\cdot\psi_{ev}+\psi_{ce}\cdot\psi_{cv}$ | 0.191001 | 0.47862 | 42928.8 |
| 615 | $\overline{\boldsymbol{u}} \sim \psi_{ac}+\psi_{ce}+\psi_{cv}+\psi_{ce}\cdot\psi_{ev}$ | 0.191892 | 0.47859 | 42929 |
| 616 | $\overline{\boldsymbol{u}} \sim \psi_{ev}+\psi_{ac}\cdot\psi_{ev}+\psi_{ac}\cdot\psi_{cv}+\psi_{ce}\cdot\psi_{cv}+\psi_{ev}\cdot\psi_{cv}$ | 0.196079 | 0.4779 | 42970.4 |
| 617 | $\overline{\boldsymbol{u}} \sim \psi_{ac}+\psi_{ac}\cdot\psi_{ce}+\psi_{ac}\cdot\psi_{cv}+\psi_{ce}\cdot\psi_{cv}+\psi_{ev}\cdot\psi_{cv}$ | 0.21258 | 0.47766 | 42984.7 |

| | | | | |
|---|---|---|---|---|
| 618 | $\overline{\boldsymbol{u}} \sim \psi_{ac}+\psi_{ac}\cdot\psi_{ce}+\psi_{ce}\cdot\psi_{cv}+\psi_{ev}\cdot\psi_{cv}$ | 0.200518 | 0.47723 | 43008.6 |
| 619 | $\overline{\boldsymbol{u}} \sim \psi_{ac}+\psi_{ac}\cdot\psi_{ev}+\psi_{ce}\cdot\psi_{ev}+\psi_{ce}\cdot\psi_{cv}+\psi_{ev}\cdot\psi_{cv}$ | 0.210586 | 0.4771 | 43017.2 |
| 620 | $\overline{\boldsymbol{u}} \sim \psi_{ac}+\psi_{ce}+\psi_{ev}\cdot\psi_{cv}$ | 0.203419 | 0.47659 | 43044.6 |
| 621 | $\overline{\boldsymbol{u}} \sim \psi_{cv}+\psi_{ac}\cdot\psi_{cv}+\psi_{ce}\cdot\psi_{ev}$ | 0.212096 | 0.47635 | 43058.6 |
| 622 | $\overline{\boldsymbol{u}} \sim \psi_{ac}+\psi_{ac}\cdot\psi_{ev}+\psi_{ce}\cdot\psi_{ev}+\psi_{ev}\cdot\psi_{cv}$ | 0.204712 | 0.47481 | 43149.1 |
| 623 | $\overline{\boldsymbol{u}} \sim \psi_{ce}+\psi_{ac}\cdot\psi_{ce}+\psi_{ac}\cdot\psi_{cv}+\psi_{ce}\cdot\psi_{ev}+\psi_{ev}\cdot\psi_{cv}$ | 0.104476 | 0.4735 | 43226.2 |
| 624 | $\overline{\boldsymbol{u}} \sim \psi_{cv}+\psi_{ac}\cdot\psi_{cv}$ | 0.213836 | 0.47281 | 43262.8 |
| 625 | $\overline{\boldsymbol{u}} \sim \psi_{ac}+\psi_{ac}\cdot\psi_{cv}+\psi_{ce}\cdot\psi_{cv}+\psi_{ev}\cdot\psi_{cv}$ | 0.235318 | 0.47277 | 43267.3 |
| 626 | $\overline{\boldsymbol{u}} \sim \psi_{ac}+\psi_{ce}+\psi_{ev}+\psi_{ac}\cdot\psi_{ev}+\psi_{ac}\cdot\psi_{cv}+\psi_{ce}\cdot\psi_{ev}$ | 0.225676 | 0.47132 | 43353.1 |
| 627 | $\overline{\boldsymbol{u}} \sim \psi_{ac}+\psi_{ce}+\psi_{ev}+\psi_{ac}\cdot\psi_{ev}+\psi_{ac}\cdot\psi_{cv}$ | 0.225643 | 0.47127 | 43354.7 |
| 628 | $\overline{\boldsymbol{u}} \sim \psi_{ac}+\psi_{ce}+\psi_{ev}+\psi_{ac}\cdot\psi_{cv}+\psi_{ce}\cdot\psi_{ev}$ | 0.22339 | 0.47093 | 43374.1 |
| 629 | $\overline{\boldsymbol{u}} \sim \psi_{ac}+\psi_{ce}+\psi_{ce}\cdot\psi_{ev}+\psi_{ce}\cdot\psi_{cv}$ | 0.17399 | 0.47086 | 43377.4 |
| 630 | $\overline{\boldsymbol{u}} \sim \psi_{ac}+\psi_{ce}+\psi_{ev}+\psi_{ac}\cdot\psi_{cv}$ | 0.223094 | 0.47082 | 43379.8 |
| 631 | $\overline{\boldsymbol{u}} \sim \psi_{ac}+\psi_{ce}+\psi_{ac}\cdot\psi_{ev}+\psi_{ac}\cdot\psi_{cv}+\psi_{ce}\cdot\psi_{ev}$ | 0.223066 | 0.4685 | 43513.8 |
| 632 | $\overline{\boldsymbol{u}} \sim \psi_{ac}\cdot\psi_{ce}+\psi_{ac}\cdot\psi_{ev}+\psi_{ac}\cdot\psi_{cv}+\psi_{ce}\cdot\psi_{cv}+\psi_{ev}\cdot\psi_{cv}$ | 0.164137 | 0.46837 | 43521.2 |
| 633 | $\overline{\boldsymbol{u}} \sim \psi_{ac}+\psi_{ce}+\psi_{ac}\cdot\psi_{ev}+\psi_{ac}\cdot\psi_{cv}$ | 0.223654 | 0.4681 | 43535.7 |
| 634 | $\overline{\boldsymbol{u}} \sim \psi_{ac}\cdot\psi_{ev}+\psi_{ac}\cdot\psi_{cv}+\psi_{ce}\cdot\psi_{cv}+\psi_{ev}\cdot\psi_{cv}$ | 0.169909 | 0.46742 | 43574.6 |
| 635 | $\overline{\boldsymbol{u}} \sim \psi_{ce}+\psi_{ev}+\psi_{cv}+\psi_{ac}\cdot\psi_{ev}+\psi_{ce}\cdot\psi_{ev}+\psi_{ce}\cdot\psi_{cv}+\psi_{ev}\cdot\psi_{cv}$ | 0.178723 | 0.46724 | 43588.1 |
| 636 | $\overline{\boldsymbol{u}} \sim \psi_{ce}+\psi_{ev}+\psi_{cv}+\psi_{ac}\cdot\psi_{ev}+\psi_{ce}\cdot\psi_{ev}+\psi_{ev}\cdot\psi_{cv}$ | 0.178001 | 0.46723 | 43587.7 |
| 637 | $\overline{\boldsymbol{u}} \sim \psi_{ce}+\psi_{ev}+\psi_{ac}\cdot\psi_{ev}+\psi_{ce}\cdot\psi_{ev}+\psi_{ev}\cdot\psi_{cv}$ | 0.177721 | 0.46709 | 43594.4 |
| 638 | $\overline{\boldsymbol{u}} \sim \psi_{ce}+\psi_{ev}+\psi_{ac}\cdot\psi_{ev}+\psi_{ce}\cdot\psi_{ev}+\psi_{ce}\cdot\psi_{cv}+\psi_{ev}\cdot\psi_{cv}$ | 0.17739 | 0.46709 | 43595.8 |
| 639 | $\overline{\boldsymbol{u}} \sim \psi_{ac}+\psi_{cv}+\psi_{ce}\cdot\psi_{cv}+\psi_{ev}\cdot\psi_{cv}$ | 0.198377 | 0.46607 | 43651.5 |
| 640 | $\overline{\boldsymbol{u}} \sim \psi_{ac}+\psi_{cv}+\psi_{ce}\cdot\psi_{ev}+\psi_{ce}\cdot\psi_{cv}+\psi_{ev}\cdot\psi_{cv}$ | 0.198312 | 0.46606 | 43653.4 |
| 641 | $\overline{\boldsymbol{u}} \sim \psi_{ac}+\psi_{ce}+\psi_{ev}+\psi_{ac}\cdot\psi_{ev}+\psi_{ce}\cdot\psi_{ev}$ | 0.289049 | 0.46576 | 43670.3 |
| 642 | $\overline{\boldsymbol{u}} \sim \psi_{ac}+\psi_{ce}+\psi_{ev}+\psi_{ac}\cdot\psi_{ev}$ | 0.289017 | 0.46572 | 43671.9 |
| 643 | $\overline{\boldsymbol{u}} \sim \psi_{ac}+\psi_{ev}+\psi_{ac}\cdot\psi_{ce}+\psi_{ac}\cdot\psi_{ev}+\psi_{ac}\cdot\psi_{cv}+\psi_{ev}\cdot\psi_{cv}$ | 0.222281 | 0.4654 | 43691.7 |
| 644 | $\overline{\boldsymbol{u}} \sim \psi_{ce}+\psi_{ev}+\psi_{cv}+\psi_{ac}\cdot\psi_{ev}+\psi_{ce}\cdot\psi_{cv}+\psi_{ev}\cdot\psi_{cv}$ | 0.177039 | 0.46533 | 43696.1 |

| | | | | |
|---|---|---|---|---|
| 645 | $\overline{\boldsymbol{u}} \sim \psi_{ac}+\psi_{ce}+\psi_{ev}+\psi_{ce}\cdot\psi_{ev}$ | 0.284499 | 0.46531 | 43695.1 |
| 646 | $\overline{\boldsymbol{u}} \sim \psi_{ce}+\psi_{ev}+\psi_{cv}+\psi_{ac}\cdot\psi_{ev}+\psi_{ev}\cdot\psi_{cv}$ | 0.176274 | 0.46531 | 43695.8 |
| 647 | $\overline{\boldsymbol{u}} \sim \psi_{ac}+\psi_{ce}+\psi_{ev}$ | 0.283986 | 0.46518 | 43701.3 |
| 648 | $\overline{\boldsymbol{u}} \sim \psi_{ce}+\psi_{ev}+\psi_{ac}\cdot\psi_{ev}+\psi_{ev}\cdot\psi_{cv}$ | 0.175978 | 0.46514 | 43705 |
| 649 | $\overline{\boldsymbol{u}} \sim \psi_{ce}+\psi_{ev}+\psi_{ac}\cdot\psi_{ev}+\psi_{ce}\cdot\psi_{cv}+\psi_{ev}\cdot\psi_{cv}$ | 0.175586 | 0.46513 | 43706.1 |
| 650 | $\overline{\boldsymbol{u}} \sim \psi_{ac}+\psi_{ac}\cdot\psi_{ce}+\psi_{ac}\cdot\psi_{ev}+\psi_{ac}\cdot\psi_{cv}+\psi_{ev}\cdot\psi_{cv}$ | 0.220816 | 0.46499 | 43714.2 |
| 651 | $\overline{\boldsymbol{u}} \sim \psi_{ac}+\psi_{ce}\cdot\psi_{ev}+\psi_{ce}\cdot\psi_{cv}+\psi_{ev}\cdot\psi_{cv}$ | 0.195168 | 0.46446 | 43743.6 |
| 652 | $\overline{\boldsymbol{u}} \sim \psi_{ce}+\psi_{ev}+\psi_{cv}+\psi_{ac}\cdot\psi_{ev}+\psi_{ce}\cdot\psi_{ev}+\psi_{ce}\cdot\psi_{cv}$ | 0.178039 | 0.46368 | 43789.9 |
| 653 | $\overline{\boldsymbol{u}} \sim \psi_{ce}+\psi_{ev}+\psi_{cv}+\psi_{ac}\cdot\psi_{ev}+\psi_{ce}\cdot\psi_{ev}$ | 0.177175 | 0.46365 | 43790.3 |
| 654 | $\overline{\boldsymbol{u}} \sim \psi_{ac}+\psi_{ce}+\psi_{ac}\cdot\psi_{ev}+\psi_{ce}\cdot\psi_{ev}$ | 0.270867 | 0.46284 | 43835.4 |
| 655 | $\overline{\boldsymbol{u}} \sim \psi_{ce}+\psi_{ac}\cdot\psi_{ce}+\psi_{ac}\cdot\psi_{cv}+\psi_{ev}\cdot\psi_{cv}$ | 0.108967 | 0.46264 | 43846.9 |
| 656 | $\overline{\boldsymbol{u}} \sim \psi_{ac}+\psi_{ev}+\psi_{ac}\cdot\psi_{ev}+\psi_{ce}\cdot\psi_{cv}+\psi_{ev}\cdot\psi_{cv}$ | 0.202947 | 0.46256 | 43852.2 |
| 657 | $\overline{\boldsymbol{u}} \sim \psi_{ev}+\psi_{ac}\cdot\psi_{ce}+\psi_{ac}\cdot\psi_{ev}+\psi_{ac}\cdot\psi_{cv}+\psi_{ev}\cdot\psi_{cv}$ | 0.204404 | 0.46246 | 43857.7 |
| 658 | $\overline{\boldsymbol{u}} \sim \psi_{ac}+\psi_{ce}+\psi_{ac}\cdot\psi_{ev}$ | 0.271275 | 0.46245 | 43856.5 |
| 659 | $\overline{\boldsymbol{u}} \sim \psi_{ce}+\psi_{ev}+\psi_{cv}+\psi_{ac}\cdot\psi_{ev}+\psi_{ce}\cdot\psi_{cv}$ | 0.176433 | 0.46201 | 43883.4 |
| 660 | $\overline{\boldsymbol{u}} \sim \psi_{ce}+\psi_{ev}+\psi_{cv}+\psi_{ac}\cdot\psi_{ev}$ | 0.175537 | 0.46198 | 43884 |
| 661 | $\overline{\boldsymbol{u}} \sim \psi_{ac}+\psi_{ev}+\psi_{ce}\cdot\psi_{cv}+\psi_{ev}\cdot\psi_{cv}$ | 0.204253 | 0.4618 | 43894.3 |
| 662 | $\overline{\boldsymbol{u}} \sim \psi_{ac}+\psi_{ev}+\psi_{ac}\cdot\psi_{ce}+\psi_{ac}\cdot\psi_{cv}+\psi_{ev}\cdot\psi_{cv}$ | 0.224269 | 0.46171 | 43900.5 |
| 663 | $\overline{\boldsymbol{u}} \sim \psi_{ac}+\psi_{ac}\cdot\psi_{ev}+\psi_{ce}\cdot\psi_{cv}+\psi_{ev}\cdot\psi_{cv}$ | 0.202197 | 0.46141 | 43916.1 |
| 664 | $\overline{\boldsymbol{u}} \sim \psi_{ac}+\psi_{ce}\cdot\psi_{cv}+\psi_{ev}\cdot\psi_{cv}$ | 0.199211 | 0.46121 | 43926.6 |
| 665 | $\overline{\boldsymbol{u}} \sim \psi_{ac}+\psi_{ev}+\psi_{ac}\cdot\psi_{ev}+\psi_{ac}\cdot\psi_{cv}+\psi_{ce}\cdot\psi_{ev}+\psi_{ce}\cdot\psi_{cv}$ | 0.185121 | 0.46073 | 43956.5 |
| 666 | $\overline{\boldsymbol{u}} \sim \psi_{ac}+\psi_{ev}+\psi_{ac}\cdot\psi_{ev}+\psi_{ce}\cdot\psi_{ev}+\psi_{ce}\cdot\psi_{cv}$ | 0.185446 | 0.46046 | 43971.2 |
| 667 | $\overline{\boldsymbol{u}} \sim \psi_{ev}+\psi_{ac}\cdot\psi_{ce}+\psi_{ac}\cdot\psi_{ev}+\psi_{ce}\cdot\psi_{cv}+\psi_{ev}\cdot\psi_{cv}$ | 0.156014 | 0.45971 | 44013.5 |
| 668 | $\overline{\boldsymbol{u}} \sim \psi_{ac}+\psi_{ev}+\psi_{ac}\cdot\psi_{cv}+\psi_{ce}\cdot\psi_{ev}+\psi_{ce}\cdot\psi_{cv}$ | 0.181598 | 0.45838 | 44088.3 |
| 669 | $\overline{\boldsymbol{u}} \sim \psi_{ac}+\psi_{ev}+\psi_{ce}\cdot\psi_{ev}+\psi_{ce}\cdot\psi_{cv}$ | 0.181838 | 0.4582 | 44097.1 |
| 670 | $\overline{\boldsymbol{u}} \sim \psi_{ev}+\psi_{ac}\cdot\psi_{ce}+\psi_{ac}\cdot\psi_{ev}+\psi_{ce}\cdot\psi_{ev}+\psi_{ev}\cdot\psi_{cv}$ | 0.143032 | 0.45711 | 44159.6 |
| 671 | $\overline{\boldsymbol{u}} \sim \psi_{ev}+\psi_{cv}+\psi_{ac}\cdot\psi_{ce}+\psi_{ac}\cdot\psi_{ev}+\psi_{ce}\cdot\psi_{ev}+\psi_{ev}\cdot\psi_{cv}$ | 0.143126 | 0.4571 | 44161 |

| | | | | |
|---|---|---|---|---|
| 672 | $\overline{\boldsymbol{u}} \sim \psi_{ce}+\psi_{ev}+\psi_{ac}\cdot\psi_{cv}+\psi_{ce}\cdot\psi_{ev}+\psi_{ev}\cdot\psi_{cv}$ | 0.139112 | 0.45656 | 44190.1 |
| 673 | $\overline{\boldsymbol{u}} \sim \psi_{ac}+\psi_{ac}\cdot\psi_{ce}+\psi_{ac}\cdot\psi_{cv}+\psi_{ev}\cdot\psi_{cv}$ | 0.243066 | 0.45639 | 44198.8 |
| 674 | $\overline{\boldsymbol{u}} \sim \psi_{ce}+\psi_{ev}+\psi_{ac}\cdot\psi_{ev}+\psi_{ce}\cdot\psi_{ev}+\psi_{ce}\cdot\psi_{cv}$ | 0.16544 | 0.45615 | 44212.9 |
| 675 | $\overline{\boldsymbol{u}} \sim \psi_{ce}+\psi_{ev}+\psi_{ac}\cdot\psi_{cv}+\psi_{ev}\cdot\psi_{cv}$ | 0.13907 | 0.45607 | 44216.5 |
| 676 | $\overline{\boldsymbol{u}} \sim \psi_{ac}\cdot\psi_{ce}+\psi_{ac}\cdot\psi_{ev}+\psi_{ac}\cdot\psi_{cv}+\psi_{ev}\cdot\psi_{cv}$ | 0.1803 | 0.45566 | 44239.4 |
| 677 | $\overline{\boldsymbol{u}} \sim \psi_{ce}+\psi_{ev}+\psi_{ac}\cdot\psi_{ev}+\psi_{ce}\cdot\psi_{cv}$ | 0.164111 | 0.4545 | 44304.3 |
| 678 | $\overline{\boldsymbol{u}} \sim \psi_{ev}+\psi_{cv}+\psi_{ac}\cdot\psi_{ce}+\psi_{ac}\cdot\psi_{ev}+\psi_{ce}\cdot\psi_{ev}$ | 0.143607 | 0.45338 | 44367.9 |
| 679 | $\overline{\boldsymbol{u}} \sim \psi_{ac}+\psi_{ev}+\psi_{ac}\cdot\psi_{ev}+\psi_{ac}\cdot\psi_{cv}+\psi_{ce}\cdot\psi_{ev}$ | 0.194097 | 0.45332 | 44371.5 |
| 680 | $\overline{\boldsymbol{u}} \sim \psi_{ev}+\psi_{cv}+\psi_{ac}\cdot\psi_{ev}+\psi_{ce}\cdot\psi_{ev}+\psi_{ce}\cdot\psi_{cv}+\psi_{ev}\cdot\psi_{cv}$ | 0.176031 | 0.45275 | 44404.1 |
| 681 | $\overline{\boldsymbol{u}} \sim \psi_{ac}+\psi_{ac}\cdot\psi_{ev}+\psi_{ac}\cdot\psi_{cv}+\psi_{ce}\cdot\psi_{ev}+\psi_{ce}\cdot\psi_{cv}$ | 0.192111 | 0.4517 | 44461.1 |
| 682 | $\overline{\boldsymbol{u}} \sim \psi_{ac}+\psi_{ac}\cdot\psi_{ev}+\psi_{ce}\cdot\psi_{ev}+\psi_{ce}\cdot\psi_{cv}$ | 0.192089 | 0.45168 | 44461.3 |
| 683 | $\overline{\boldsymbol{u}} \sim \psi_{ac}+\psi_{ev}+\psi_{ac}\cdot\psi_{cv}+\psi_{ce}\cdot\psi_{ev}$ | 0.189914 | 0.45157 | 44467.8 |
| 684 | $\overline{\boldsymbol{u}} \sim \psi_{ev}+\psi_{cv}+\psi_{ac}\cdot\psi_{ev}+\psi_{ce}\cdot\psi_{ev}+\psi_{ce}\cdot\psi_{cv}$ | 0.175295 | 0.44977 | 44568.4 |
| 685 | $\overline{\boldsymbol{u}} \sim \psi_{ev}+\psi_{cv}+\psi_{ac}\cdot\psi_{ev}+\psi_{ce}\cdot\psi_{cv}+\psi_{ev}\cdot\psi_{cv}$ | 0.178064 | 0.44966 | 44574.5 |
| 686 | $\overline{\boldsymbol{u}} \sim \psi_{ac}+\psi_{cv}+\psi_{ac}\cdot\psi_{ce}+\psi_{ce}\cdot\psi_{ev}+\psi_{ce}\cdot\psi_{cv}$ | 0.146291 | 0.44955 | 44580.7 |
| 687 | $\overline{\boldsymbol{u}} \sim \psi_{ev}+\psi_{ac}\cdot\psi_{ev}+\psi_{ce}\cdot\psi_{ev}+\psi_{ce}\cdot\psi_{cv}+\psi_{ev}\cdot\psi_{cv}$ | 0.170089 | 0.44775 | 44679.8 |
| 688 | $\overline{\boldsymbol{u}} \sim \psi_{ac}+\psi_{ev}+\psi_{ac}\cdot\psi_{ev}+\psi_{ce}\cdot\psi_{ev}$ | 0.21787 | 0.44673 | 44734.8 |
| 689 | $\overline{\boldsymbol{u}} \sim \psi_{ev}+\psi_{cv}+\psi_{ac}\cdot\psi_{ev}+\psi_{ce}\cdot\psi_{cv}$ | 0.177368 | 0.44651 | 44747.1 |
| 690 | $\overline{\boldsymbol{u}} \sim \psi_{ac}+\psi_{ac}\cdot\psi_{ev}+\psi_{ac}\cdot\psi_{cv}+\psi_{ce}\cdot\psi_{ev}$ | 0.197709 | 0.44643 | 44751.7 |
| 691 | $\overline{\boldsymbol{u}} \sim \psi_{ac}+\psi_{ce}+\psi_{ac}\cdot\psi_{cv}+\psi_{ce}\cdot\psi_{ev}$ | 0.178335 | 0.44604 | 44772.9 |
| 692 | $\overline{\boldsymbol{u}} \sim \psi_{ac}+\psi_{ev}+\psi_{ce}\cdot\psi_{ev}$ | 0.212286 | 0.44486 | 44836.8 |
| 693 | $\overline{\boldsymbol{u}} \sim \psi_{ac}+\psi_{cv}+\psi_{ac}\cdot\psi_{ce}+\psi_{ce}\cdot\psi_{ev}+\psi_{ev}\cdot\psi_{cv}$ | 0.157793 | 0.4441 | 44880.4 |
| 694 | $\overline{\boldsymbol{u}} \sim \psi_{ce}+\psi_{ac}\cdot\psi_{cv}+\psi_{ce}\cdot\psi_{ev}+\psi_{ev}\cdot\psi_{cv}$ | 0.140754 | 0.44138 | 45028 |
| 695 | $\overline{\boldsymbol{u}} \sim \psi_{ev}+\psi_{ac}\cdot\psi_{ce}+\psi_{ac}\cdot\psi_{cv}+\psi_{ce}\cdot\psi_{ev}+\psi_{ce}\cdot\psi_{cv}+\psi_{ev}\cdot\psi_{cv}$ | 0.131092 | 0.44101 | 45050.4 |
| 696 | $\overline{\boldsymbol{u}} \sim \psi_{ev}+\psi_{ac}\cdot\psi_{cv}+\psi_{ce}\cdot\psi_{ev}+\psi_{ce}\cdot\psi_{cv}+\psi_{ev}\cdot\psi_{cv}$ | 0.130354 | 0.441 | 45049.8 |
| 697 | $\overline{\boldsymbol{u}} \sim \psi_{ac}\cdot\psi_{cv}+\psi_{ce}\cdot\psi_{ev}+\psi_{ce}\cdot\psi_{cv}+\psi_{ev}\cdot\psi_{cv}$ | 0.130132 | 0.44098 | 45049.9 |
| 698 | $\overline{\boldsymbol{u}} \sim \psi_{ac}\cdot\psi_{ce}+\psi_{ac}\cdot\psi_{cv}+\psi_{ce}\cdot\psi_{ev}+\psi_{ce}\cdot\psi_{cv}+\psi_{ev}\cdot\psi_{cv}$ | 0.130735 | 0.44098 | 45050.9 |

| | | | | |
|---|---|---|---|---|
| 699 | $\overline{\boldsymbol{u}} \sim \psi_{ac}+\psi_{ev}+\psi_{ac}\cdot\psi_{ev}+\psi_{ac}\cdot\psi_{cv}+\psi_{ev}\cdot\psi_{cv}$ | 0.23118 | 0.44045 | 45079.6 |
| 700 | $\overline{\boldsymbol{u}} \sim \psi_{ac}+\psi_{ac}\cdot\psi_{ev}+\psi_{ac}\cdot\psi_{cv}+\psi_{ev}\cdot\psi_{cv}$ | 0.228288 | 0.44004 | 45100.8 |
| 701 | $\overline{\boldsymbol{u}} \sim \psi_{ac}+\psi_{ac}\cdot\psi_{ev}+\psi_{ce}\cdot\psi_{ev}$ | 0.219021 | 0.43993 | 45105.8 |
| 702 | $\overline{\boldsymbol{u}} \sim \psi_{ev}+\psi_{cv}+\psi_{ac}\cdot\psi_{ev}+\psi_{ce}\cdot\psi_{ev}+\psi_{ev}\cdot\psi_{cv}$ | 0.158997 | 0.43963 | 45124.2 |
| 703 | $\overline{\boldsymbol{u}} \sim \psi_{ac}+\psi_{ce}+\psi_{ce}\cdot\psi_{ev}$ | 0.194084 | 0.43941 | 45134 |
| 704 | $\overline{\boldsymbol{u}} \sim \psi_{ev}+\psi_{ac}\cdot\psi_{ev}+\psi_{ce}\cdot\psi_{ev}+\psi_{ev}\cdot\psi_{cv}$ | 0.158779 | 0.4393 | 45141.4 |
| 705 | $\overline{\boldsymbol{u}} \sim \psi_{ev}+\psi_{ac}\cdot\psi_{ev}+\psi_{ce}\cdot\psi_{cv}+\psi_{ev}\cdot\psi_{cv}$ | 0.171993 | 0.4393 | 45141 |
| 706 | $\overline{\boldsymbol{u}} \sim \psi_{ev}+\psi_{ac}\cdot\psi_{ce}+\psi_{ac}\cdot\psi_{cv}+\psi_{ce}\cdot\psi_{ev}+\psi_{ev}\cdot\psi_{cv}$ | 0.129167 | 0.43804 | 45210.5 |
| 707 | $\overline{\boldsymbol{u}} \sim \psi_{ac}+\psi_{cv}+\psi_{ce}\cdot\psi_{ev}+\psi_{ev}\cdot\psi_{cv}$ | 0.170406 | 0.43797 | 45213.4 |
| 708 | $\overline{\boldsymbol{u}} \sim \psi_{ev}+\psi_{ac}\cdot\psi_{cv}+\psi_{ce}\cdot\psi_{ev}+\psi_{ev}\cdot\psi_{cv}$ | 0.13385 | 0.43717 | 45256.7 |
| 709 | $\overline{\boldsymbol{u}} \sim \psi_{ac}\cdot\psi_{ce}+\psi_{ac}\cdot\psi_{cv}+\psi_{ce}\cdot\psi_{ev}+\psi_{ev}\cdot\psi_{cv}$ | 0.127225 | 0.43707 | 45262.1 |
| 710 | $\overline{\boldsymbol{u}} \sim \psi_{ev}+\psi_{cv}+\psi_{ac}\cdot\psi_{ev}+\psi_{ce}\cdot\psi_{ev}$ | 0.158644 | 0.43676 | 45278.5 |
| 711 | $\overline{\boldsymbol{u}} \sim \psi_{ac}+\psi_{ev}+\psi_{ac}\cdot\psi_{cv}+\psi_{ev}\cdot\psi_{cv}$ | 0.234687 | 0.43621 | 45308.6 |
| 712 | $\overline{\boldsymbol{u}} \sim \psi_{ce}+\psi_{ev}+\psi_{ac}\cdot\psi_{ev}+\psi_{ac}\cdot\psi_{cv}+\psi_{ce}\cdot\psi_{ev}$ | 0.177479 | 0.43494 | 45377.8 |
| 713 | $\overline{\boldsymbol{u}} \sim \psi_{ev}+\psi_{ac}\cdot\psi_{ev}+\psi_{ac}\cdot\psi_{cv}+\psi_{ev}\cdot\psi_{cv}$ | 0.19684 | 0.43492 | 45378.2 |
| 714 | $\overline{\boldsymbol{u}} \sim \psi_{ac}\cdot\psi_{cv}+\psi_{ce}\cdot\psi_{ev}+\psi_{ev}\cdot\psi_{cv}$ | 0.134644 | 0.43474 | 45386.7 |
| 715 | $\overline{\boldsymbol{u}} \sim \psi_{ce}+\psi_{ac}\cdot\psi_{cv}+\psi_{ev}\cdot\psi_{cv}$ | 0.148806 | 0.43366 | 45444.9 |
| 716 | $\overline{\boldsymbol{u}} \sim \psi_{ce}+\psi_{ev}+\psi_{ac}\cdot\psi_{ev}+\psi_{ce}\cdot\psi_{ev}$ | 0.1855 | 0.43358 | 45450.1 |
| 717 | $\overline{\boldsymbol{u}} \sim \psi_{ce}+\psi_{cv}+\psi_{ac}\cdot\psi_{ce}+\psi_{ac}\cdot\psi_{ev}+\psi_{ce}\cdot\psi_{cv}+\psi_{ev}\cdot\psi_{cv}$ | 0.0944355 | 0.43355 | 45454.1 |
| 718 | $\overline{\boldsymbol{u}} \sim \psi_{ce}+\psi_{ev}+\psi_{ac}\cdot\psi_{ev}+\psi_{ac}\cdot\psi_{cv}$ | 0.17607 | 0.43341 | 45459.2 |
| 719 | $\overline{\boldsymbol{u}} \sim \psi_{ce}+\psi_{ev}+\psi_{ac}\cdot\psi_{ev}$ | 0.183583 | 0.43215 | 45525.8 |
| 720 | $\overline{\boldsymbol{u}} \sim \psi_{ce}+\psi_{ac}\cdot\psi_{ce}+\psi_{ac}\cdot\psi_{ev}+\psi_{ac}\cdot\psi_{cv}+\psi_{ce}\cdot\psi_{ev}+\psi_{ce}\cdot\psi_{cv}$ | 0.0976593 | 0.431 | 45590.8 |
| 721 | $\overline{\boldsymbol{u}} \sim \psi_{ev}+\psi_{ac}\cdot\psi_{ce}+\psi_{ac}\cdot\psi_{ev}+\psi_{ac}\cdot\psi_{cv}+\psi_{ce}\cdot\psi_{ev}+\psi_{ce}\cdot\psi_{cv}$ | 0.138191 | 0.43053 | 45615.6 |
| 722 | $\overline{\boldsymbol{u}} \sim \psi_{ev}+\psi_{ac}\cdot\psi_{ce}+\psi_{ac}\cdot\psi_{ev}+\psi_{ac}\cdot\psi_{cv}+\psi_{ce}\cdot\psi_{ev}$ | 0.138639 | 0.43002 | 45641.9 |
| 723 | $\overline{\boldsymbol{u}} \sim \psi_{ac}+\psi_{ac}\cdot\psi_{cv}+\psi_{ev}\cdot\psi_{cv}$ | 0.296579 | 0.42997 | 45642.6 |
| 724 | $\overline{\boldsymbol{u}} \sim \psi_{ev}+\psi_{ac}\cdot\psi_{ce}+\psi_{ac}\cdot\psi_{ev}+\psi_{ce}\cdot\psi_{ev}$ | 0.138778 | 0.42994 | 45645.2 |
| 725 | $\overline{\boldsymbol{u}} \sim \psi_{ev}+\psi_{ac}\cdot\psi_{ce}+\psi_{ac}\cdot\psi_{ev}+\psi_{ce}\cdot\psi_{ev}+\psi_{ce}\cdot\psi_{cv}$ | 0.138789 | 0.42993 | 45646.6 |

| | | | | |
|---|---|---|---|---|
| 726 | $\overline{\boldsymbol{u}} \sim \psi_{ac}+\psi_{ev}+\psi_{cv}+\psi_{ac}\cdot\psi_{ce}+\psi_{ac}\cdot\psi_{ev}+\psi_{ev}\cdot\psi_{cv}$ | 0.210842 | 0.42986 | 45651.4 |
| 727 | $\overline{\boldsymbol{u}} \sim \psi_{ac}+\psi_{ev}+\psi_{cv}+\psi_{ac}\cdot\psi_{ce}+\psi_{ac}\cdot\psi_{ev}$ | 0.210638 | 0.42983 | 45652.4 |
| 728 | $\overline{\boldsymbol{u}} \sim \psi_{ac}+\psi_{ev}+\psi_{cv}+\psi_{ac}\cdot\psi_{ce}$ | 0.208931 | 0.42935 | 45676.7 |
| 729 | $\overline{\boldsymbol{u}} \sim \psi_{ac}+\psi_{ev}+\psi_{cv}+\psi_{ac}\cdot\psi_{ce}+\psi_{ev}\cdot\psi_{cv}$ | 0.208929 | 0.42933 | 45678.7 |
| 730 | $\overline{\boldsymbol{u}} \sim \psi_{ac}+\psi_{cv}+\psi_{ac}\cdot\psi_{ce}+\psi_{ac}\cdot\psi_{ev}$ | 0.210617 | 0.42677 | 45814.2 |
| 731 | $\overline{\boldsymbol{u}} \sim \psi_{ac}+\psi_{cv}+\psi_{ac}\cdot\psi_{ce}+\psi_{ac}\cdot\psi_{ev}+\psi_{ev}\cdot\psi_{cv}$ | 0.210532 | 0.42675 | 45816 |
| 732 | $\overline{\boldsymbol{u}} \sim \psi_{ac}\cdot\psi_{ev}+\psi_{ac}\cdot\psi_{cv}+\psi_{ev}\cdot\psi_{cv}$ | 0.163512 | 0.42477 | 45919.1 |
| 733 | $\overline{\boldsymbol{u}} \sim \psi_{ac}+\psi_{ev}+\psi_{ac}\cdot\psi_{ce}+\psi_{ac}\cdot\psi_{ev}+\psi_{ev}\cdot\psi_{cv}$ | 0.200366 | 0.42157 | 46090.2 |
| 734 | $\overline{\boldsymbol{u}} \sim \psi_{ac}+\psi_{ev}+\psi_{ac}\cdot\psi_{ce}+\psi_{ev}\cdot\psi_{cv}$ | 0.20067 | 0.42156 | 46089.5 |
| 735 | $\overline{\boldsymbol{u}} \sim \psi_{ce}+\psi_{cv}+\psi_{ac}\cdot\psi_{ce}+\psi_{ac}\cdot\psi_{ev}+\psi_{ev}\cdot\psi_{cv}$ | 0.0996297 | 0.42055 | 46143.9 |
| 736 | $\overline{\boldsymbol{u}} \sim \psi_{ac}+\psi_{ac}\cdot\psi_{ce}+\psi_{ac}\cdot\psi_{ev}+\psi_{ev}\cdot\psi_{cv}$ | 0.200466 | 0.41986 | 46179 |
| 737 | $\overline{\boldsymbol{u}} \sim \psi_{ac}+\psi_{cv}+\psi_{ac}\cdot\psi_{ce}+\psi_{ce}\cdot\psi_{cv}$ | 0.134935 | 0.41982 | 46180.9 |
| 738 | $\overline{\boldsymbol{u}} \sim \psi_{ac}+\psi_{cv}+\psi_{ce}\cdot\psi_{ev}+\psi_{ce}\cdot\psi_{cv}$ | 0.176893 | 0.41495 | 46435.7 |
| 739 | $\overline{\boldsymbol{u}} \sim \psi_{ev}+\psi_{ac}\cdot\psi_{ev}+\psi_{ac}\cdot\psi_{cv}+\psi_{ce}\cdot\psi_{ev}+\psi_{ce}\cdot\psi_{cv}$ | 0.150893 | 0.41395 | 46488.6 |
| 740 | $\overline{\boldsymbol{u}} \sim \psi_{ce}+\psi_{ev}+\psi_{ac}\cdot\psi_{ce}+\psi_{ac}\cdot\psi_{cv}+\psi_{ce}\cdot\psi_{ev}+\psi_{ce}\cdot\psi_{cv}$ | 0.103047 | 0.41388 | 46493.2 |
| 741 | $\overline{\boldsymbol{u}} \sim \psi_{ce}+\psi_{ev}+\psi_{ac}\cdot\psi_{ce}+\psi_{ac}\cdot\psi_{cv}+\psi_{ce}\cdot\psi_{cv}$ | 0.102883 | 0.41376 | 46498.3 |
| 742 | $\overline{\boldsymbol{u}} \sim \psi_{ac}+\psi_{ac}\cdot\psi_{ce}+\psi_{ce}\cdot\psi_{ev}+\psi_{ev}\cdot\psi_{cv}$ | 0.177166 | 0.4133 | 46521.1 |
| 743 | $\overline{\boldsymbol{u}} \sim \psi_{ac}+\psi_{ce}\cdot\psi_{ev}+\psi_{ev}\cdot\psi_{cv}$ | 0.174245 | 0.41219 | 46577.6 |
| 744 | $\overline{\boldsymbol{u}} \sim \psi_{ev}+\psi_{ac}\cdot\psi_{ev}+\psi_{ce}\cdot\psi_{ev}+\psi_{ce}\cdot\psi_{cv}$ | 0.152071 | 0.41164 | 46607.1 |
| 745 | $\overline{\boldsymbol{u}} \sim \psi_{ev}+\psi_{ac}\cdot\psi_{ce}+\psi_{ac}\cdot\psi_{cv}+\psi_{ev}\cdot\psi_{cv}$ | 0.148438 | 0.41118 | 46630.9 |
| 746 | $\overline{\boldsymbol{u}} \sim \psi_{ev}+\psi_{ac}\cdot\psi_{ce}+\psi_{ac}\cdot\psi_{cv}+\psi_{ce}\cdot\psi_{cv}+\psi_{ev}\cdot\psi_{cv}$ | 0.148673 | 0.41117 | 46632.5 |
| 747 | $\overline{\boldsymbol{u}} \sim \psi_{ac}+\psi_{cv}+\psi_{ac}\cdot\psi_{ce}+\psi_{ev}\cdot\psi_{cv}$ | 0.183899 | 0.40885 | 46751.3 |
| 748 | $\overline{\boldsymbol{u}} \sim \psi_{ev}+\psi_{ac}\cdot\psi_{ev}+\psi_{ac}\cdot\psi_{cv}+\psi_{ce}\cdot\psi_{ev}$ | 0.158389 | 0.40676 | 46858.9 |
| 749 | $\overline{\boldsymbol{u}} \sim \psi_{ev}+\psi_{ac}\cdot\psi_{ev}+\psi_{ce}\cdot\psi_{ev}$ | 0.162385 | 0.40567 | 46913.7 |
| 750 | $\overline{\boldsymbol{u}} \sim \psi_{ac}+\psi_{ac}\cdot\psi_{ce}+\psi_{ev}\cdot\psi_{cv}$ | 0.182604 | 0.40497 | 46949.6 |
| 751 | $\overline{\boldsymbol{u}} \sim \psi_{ce}+\psi_{ev}+\psi_{ac}\cdot\psi_{cv}+\psi_{ce}\cdot\psi_{ev}+\psi_{ce}\cdot\psi_{cv}$ | 0.112105 | 0.40416 | 46992.7 |
| 752 | $\overline{\boldsymbol{u}} \sim \psi_{ce}+\psi_{ev}+\psi_{ac}\cdot\psi_{cv}+\psi_{ce}\cdot\psi_{cv}$ | 0.11194 | 0.40403 | 46998.5 |

| | | | | |
|---|---|---|---|---|
| 753 | $\overline{\boldsymbol{u}} \sim \psi_{ac}+\psi_{ev}+\psi_{ac}\cdot\psi_{ce}+\psi_{ac}\cdot\psi_{ev}+\psi_{ac}\cdot\psi_{cv}+\psi_{ce}\cdot\psi_{cv}$ | 0.167129 | 0.40316 | 47044.7 |
| 754 | $\overline{\boldsymbol{u}} \sim \psi_{ac}+\psi_{ev}+\psi_{ac}\cdot\psi_{ce}+\psi_{ac}\cdot\psi_{cv}+\psi_{ce}\cdot\psi_{cv}$ | 0.165417 | 0.40265 | 47069.9 |
| 755 | $\overline{\boldsymbol{u}} \sim \psi_{ce}+\psi_{cv}+\psi_{ac}\cdot\psi_{ev}+\psi_{ce}\cdot\psi_{ev}+\psi_{ce}\cdot\psi_{cv}+\psi_{ev}\cdot\psi_{cv}$ | 0.128871 | 0.4019 | 47109.4 |
| 756 | $\overline{\boldsymbol{u}} \sim \psi_{ce}+\psi_{cv}+\psi_{ac}\cdot\psi_{ev}+\psi_{ce}\cdot\psi_{ev}+\psi_{ev}\cdot\psi_{cv}$ | 0.12741 | 0.40159 | 47124 |
| 757 | $\overline{\boldsymbol{u}} \sim \psi_{ac}+\psi_{ev}+\psi_{ac}\cdot\psi_{ev}+\psi_{ac}\cdot\psi_{cv}+\psi_{ce}\cdot\psi_{cv}$ | 0.180598 | 0.40069 | 47169.5 |
| 758 | $\overline{\boldsymbol{u}} \sim \psi_{ac}+\psi_{ac}\cdot\psi_{ce}+\psi_{ac}\cdot\psi_{ev}+\psi_{ac}\cdot\psi_{cv}+\psi_{ce}\cdot\psi_{cv}$ | 0.169917 | 0.40031 | 47188.9 |
| 759 | $\overline{\boldsymbol{u}} \sim \psi_{ac}+\psi_{ev}+\psi_{cv}+\psi_{ac}\cdot\psi_{ev}+\psi_{ev}\cdot\psi_{cv}$ | 0.227776 | 0.40016 | 47196.7 |
| 760 | $\overline{\boldsymbol{u}} \sim \psi_{ac}+\psi_{ev}+\psi_{cv}+\psi_{ac}\cdot\psi_{ev}$ | 0.22743 | 0.40013 | 47197.3 |
| 761 | $\overline{\boldsymbol{u}} \sim \psi_{ac}+\psi_{ev}+\psi_{ac}\cdot\psi_{cv}+\psi_{ce}\cdot\psi_{cv}$ | 0.178808 | 0.40013 | 47197.2 |
| 762 | $\overline{\boldsymbol{u}} \sim \psi_{ac}+\psi_{ev}+\psi_{cv}$ | 0.224441 | 0.39968 | 47218.9 |
| 763 | $\overline{\boldsymbol{u}} \sim \psi_{ac}+\psi_{ev}+\psi_{cv}+\psi_{ev}\cdot\psi_{cv}$ | 0.224438 | 0.39966 | 47220.9 |
| 764 | $\overline{\boldsymbol{u}} \sim \psi_{ev}+\psi_{ac}\cdot\psi_{cv}+\psi_{ce}\cdot\psi_{cv}+\psi_{ev}\cdot\psi_{cv}$ | 0.133394 | 0.39806 | 47302.1 |
| 765 | $\overline{\boldsymbol{u}} \sim \psi_{ac}+\psi_{ac}\cdot\psi_{ev}+\psi_{ac}\cdot\psi_{cv}+\psi_{ce}\cdot\psi_{cv}$ | 0.182681 | 0.39793 | 47308.3 |
| 766 | $\overline{\boldsymbol{u}} \sim \psi_{ce}+\psi_{ac}\cdot\psi_{ce}+\psi_{ac}\cdot\psi_{ev}+\psi_{ce}\cdot\psi_{ev}+\psi_{ev}\cdot\psi_{cv}$ | 0.105562 | 0.39772 | 47320.1 |
| 767 | $\overline{\boldsymbol{u}} \sim \psi_{ac}+\psi_{cv}+\psi_{ac}\cdot\psi_{ev}$ | 0.22405 | 0.39694 | 47357.6 |
| 768 | $\overline{\boldsymbol{u}} \sim \psi_{ac}+\psi_{cv}+\psi_{ac}\cdot\psi_{ev}+\psi_{ev}\cdot\psi_{cv}$ | 0.223894 | 0.39693 | 47359.3 |
| 769 | $\overline{\boldsymbol{u}} \sim \psi_{ev}+\psi_{cv}+\psi_{ac}\cdot\psi_{ce}+\psi_{ac}\cdot\psi_{ev}+\psi_{ev}\cdot\psi_{cv}$ | 0.165275 | 0.39426 | 47494.4 |
| 770 | $\overline{\boldsymbol{u}} \sim \psi_{ce}+\psi_{ac}\cdot\psi_{ev}+\psi_{ac}\cdot\psi_{cv}+\psi_{ce}\cdot\psi_{ev}+\psi_{ce}\cdot\psi_{cv}$ | 0.113125 | 0.39421 | 47497.2 |
| 771 | $\overline{\boldsymbol{u}} \sim \psi_{ev}+\psi_{ac}\cdot\psi_{ce}+\psi_{ac}\cdot\psi_{ev}+\psi_{ev}\cdot\psi_{cv}$ | 0.16499 | 0.39341 | 47536.1 |
| 772 | $\overline{\boldsymbol{u}} \sim \psi_{ev}+\psi_{cv}+\psi_{ac}\cdot\psi_{ce}+\psi_{ac}\cdot\psi_{ev}$ | 0.164878 | 0.39151 | 47631.5 |
| 773 | $\overline{\boldsymbol{u}} \sim \psi_{ac}+\psi_{ev}+\psi_{ac}\cdot\psi_{ev}+\psi_{ev}\cdot\psi_{cv}$ | 0.206531 | 0.39148 | 47633 |
| 774 | $\overline{\boldsymbol{u}} \sim \psi_{ac}+\psi_{ev}+\psi_{ev}\cdot\psi_{cv}$ | 0.207108 | 0.39146 | 47633.1 |
| 775 | $\overline{\boldsymbol{u}} \sim \psi_{ce}+\psi_{ac}\cdot\psi_{ce}+\psi_{ac}\cdot\psi_{cv}+\psi_{ce}\cdot\psi_{ev}+\psi_{ce}\cdot\psi_{cv}$ | 0.100626 | 0.39143 | 47636.4 |
| 776 | $\overline{\boldsymbol{u}} \sim \psi_{ac}+\psi_{ac}\cdot\psi_{ev}+\psi_{ev}\cdot\psi_{cv}$ | 0.205673 | 0.38971 | 47720.2 |
| 777 | $\overline{\boldsymbol{u}} \sim \psi_{ce}+\psi_{ac}\cdot\psi_{ce}+\psi_{ac}\cdot\psi_{ev}+\psi_{ac}\cdot\psi_{cv}+\psi_{ce}\cdot\psi_{ev}$ | 0.0875761 | 0.38949 | 47733.3 |
| 778 | $\overline{\boldsymbol{u}} \sim \psi_{ac}\cdot\psi_{ce}+\psi_{ac}\cdot\psi_{cv}+\psi_{ce}\cdot\psi_{cv}+\psi_{ev}\cdot\psi_{cv}$ | 0.157919 | 0.38893 | 47760.3 |
| 779 | $\overline{\boldsymbol{u}} \sim \psi_{ce}+\psi_{ac}\cdot\psi_{ev}+\psi_{ce}\cdot\psi_{ev}+\psi_{ce}\cdot\psi_{cv}+\psi_{ev}\cdot\psi_{cv}$ | 0.129868 | 0.38716 | 47849.6 |

| | | | | |
|---|---|---|---|---|
| 780 | $\overline{\boldsymbol{u}} \sim \psi_{ac}{+}\psi_{ev}{+}\psi_{ac}{\cdot}\psi_{ce}{+}\psi_{ac}{\cdot}\psi_{ev}{+}\psi_{ac}{\cdot}\psi_{cv}$ | 0.193481 | 0.38687 | 47863.6 |
| 781 | $\overline{\boldsymbol{u}} \sim \psi_{ac}{\cdot}\psi_{ce}{+}\psi_{ac}{\cdot}\psi_{cv}{+}\psi_{ev}{\cdot}\psi_{cv}$ | 0.1575 | 0.38684 | 47863.4 |
| 782 | $\overline{\boldsymbol{u}} \sim \psi_{ac}{+}\psi_{ev}{+}\psi_{ac}{\cdot}\psi_{ce}{+}\psi_{ac}{\cdot}\psi_{cv}$ | 0.191494 | 0.38625 | 47893.8 |
| 783 | $\overline{\boldsymbol{u}} \sim \psi_{ac}{+}\psi_{ac}{\cdot}\psi_{ce}{+}\psi_{ac}{\cdot}\psi_{ev}{+}\psi_{ac}{\cdot}\psi_{cv}$ | 0.195088 | 0.38422 | 47994 |
| 784 | $\overline{\boldsymbol{u}} \sim \psi_{ce}{+}\psi_{ac}{\cdot}\psi_{cv}{+}\psi_{ce}{\cdot}\psi_{ev}{+}\psi_{ce}{\cdot}\psi_{cv}$ | 0.10913 | 0.38178 | 48114.7 |
| 785 | $\overline{\boldsymbol{u}} \sim \psi_{ac}{+}\psi_{ce}{+}\psi_{ac}{\cdot}\psi_{cv}{+}\psi_{ce}{\cdot}\psi_{cv}$ | 0.160912 | 0.37996 | 48204 |
| 786 | $\overline{\boldsymbol{u}} \sim \psi_{ac}{+}\psi_{ev}{+}\psi_{ac}{\cdot}\psi_{ce}{+}\psi_{ac}{\cdot}\psi_{ev}{+}\psi_{ce}{\cdot}\psi_{cv}$ | 0.213659 | 0.37917 | 48243.7 |
| 787 | $\overline{\boldsymbol{u}} \sim \psi_{ac}{+}\psi_{cv}{+}\psi_{ev}{\cdot}\psi_{cv}$ | 0.180944 | 0.37908 | 48246 |
| 788 | $\overline{\boldsymbol{u}} \sim \psi_{ac}{+}\psi_{ev}{+}\psi_{ac}{\cdot}\psi_{ce}{+}\psi_{ac}{\cdot}\psi_{ev}$ | 0.218021 | 0.37899 | 48251.6 |
| 789 | $\overline{\boldsymbol{u}} \sim \psi_{ac}{+}\psi_{ev}{+}\psi_{ac}{\cdot}\psi_{ce}{+}\psi_{ce}{\cdot}\psi_{cv}$ | 0.211215 | 0.37848 | 48276.6 |
| 790 | $\overline{\boldsymbol{u}} \sim \psi_{ac}{+}\psi_{ev}{+}\psi_{ac}{\cdot}\psi_{ce}$ | 0.215508 | 0.37829 | 48284.8 |
| 791 | $\overline{\boldsymbol{u}} \sim \psi_{ac}{+}\psi_{ac}{\cdot}\psi_{ce}{+}\psi_{ac}{\cdot}\psi_{ev}{+}\psi_{ce}{\cdot}\psi_{cv}$ | 0.214206 | 0.37659 | 48369.1 |
| 792 | $\overline{\boldsymbol{u}} \sim \psi_{ac}{+}\psi_{ac}{\cdot}\psi_{ce}{+}\psi_{ac}{\cdot}\psi_{ev}$ | 0.218274 | 0.37641 | 48376.9 |
| 793 | $\overline{\boldsymbol{u}} \sim \psi_{ac}{+}\psi_{ev}{\cdot}\psi_{cv}$ | 0.178468 | 0.37567 | 48412.1 |
| 794 | $\overline{\boldsymbol{u}} \sim \psi_{ac}{+}\psi_{ce}{+}\psi_{cv}{+}\psi_{ce}{\cdot}\psi_{cv}$ | 0.190928 | 0.37045 | 48667.6 |
| 795 | $\overline{\boldsymbol{u}} \sim \psi_{ac}{+}\psi_{ce}{+}\psi_{cv}$ | 0.192877 | 0.37043 | 48667.5 |
| 796 | $\overline{\boldsymbol{u}} \sim \psi_{ce}{+}\psi_{cv}{+}\psi_{ac}{\cdot}\psi_{ce}{+}\psi_{ac}{\cdot}\psi_{ev}{+}\psi_{ce}{\cdot}\psi_{ev}{+}\psi_{ce}{\cdot}\psi_{cv}$ | 0.104421 | 0.37031 | 48676.4 |
| 797 | $\overline{\boldsymbol{u}} \sim \psi_{ce}{+}\psi_{ac}{\cdot}\psi_{ev}{+}\psi_{ce}{\cdot}\psi_{ev}{+}\psi_{ev}{\cdot}\psi_{cv}$ | 0.117492 | 0.36436 | 48960.6 |
| 798 | $\overline{\boldsymbol{u}} \sim \psi_{ac}{+}\psi_{ev}{+}\psi_{ac}{\cdot}\psi_{ev}{+}\psi_{ce}{\cdot}\psi_{cv}$ | 0.320765 | 0.36416 | 48970.3 |
| 799 | $\overline{\boldsymbol{u}} \sim \psi_{ac}{+}\psi_{ev}{+}\psi_{ce}{\cdot}\psi_{cv}$ | 0.310708 | 0.36335 | 49008.1 |
| 800 | $\overline{\boldsymbol{u}} \sim \psi_{ac}{+}\psi_{ce}{+}\psi_{ce}{\cdot}\psi_{cv}$ | 0.160223 | 0.36283 | 49032.8 |
| 801 | $\overline{\boldsymbol{u}} \sim \psi_{ac}{+}\psi_{ac}{\cdot}\psi_{ev}{+}\psi_{ce}{\cdot}\psi_{cv}$ | 0.292446 | 0.36132 | 49105 |
| 802 | $\overline{\boldsymbol{u}} \sim \psi_{ce}{+}\psi_{ac}{\cdot}\psi_{ce}{+}\psi_{ac}{\cdot}\psi_{ev}{+}\psi_{ce}{\cdot}\psi_{ev}{+}\psi_{ce}{\cdot}\psi_{cv}$ | 0.10589 | 0.36064 | 49139 |
| 803 | $\overline{\boldsymbol{u}} \sim \psi_{ce}{+}\psi_{cv}{+}\psi_{ac}{\cdot}\psi_{ce}{+}\psi_{ac}{\cdot}\psi_{ev}{+}\psi_{ce}{\cdot}\psi_{ev}$ | 0.10792 | 0.3601 | 49164.9 |
| 804 | $\overline{\boldsymbol{u}} \sim \psi_{ce}{+}\psi_{ac}{\cdot}\psi_{ce}{+}\psi_{ac}{\cdot}\psi_{ev}{+}\psi_{ce}{\cdot}\psi_{ev}$ | 0.107389 | 0.35969 | 49183.2 |
| 805 | $\overline{\boldsymbol{u}} \sim \psi_{cv}{+}\psi_{ac}{\cdot}\psi_{ce}{+}\psi_{ac}{\cdot}\psi_{ev}{+}\psi_{ce}{\cdot}\psi_{ev}{+}\psi_{ce}{\cdot}\psi_{cv}{+}\psi_{ev}{\cdot}\psi_{cv}$ | 0.120084 | 0.35849 | 49242.4 |
| 806 | $\overline{\boldsymbol{u}} \sim \psi_{ac}{\cdot}\psi_{cv}{+}\psi_{ce}{\cdot}\psi_{cv}{+}\psi_{ev}{\cdot}\psi_{cv}$ | 0.150015 | 0.3582 | 49253.4 |

| | | | | |
|---|---|---|---|---|
| 807 | $\overline{\boldsymbol{u}} \sim \psi_{ce}+\psi_{ac}\cdot\psi_{ce}+\psi_{ac}\cdot\psi_{ev}+\psi_{ce}\cdot\psi_{cv}+\psi_{ev}\cdot\psi_{cv}$ | 0.0945711 | 0.3568 | 49321.5 |
| 808 | $\overline{\boldsymbol{u}} \sim \psi_{ac}\cdot\psi_{ce}+\psi_{ac}\cdot\psi_{ev}+\psi_{ce}\cdot\psi_{ev}+\psi_{ce}\cdot\psi_{cv}+\psi_{ev}\cdot\psi_{cv}$ | 0.119758 | 0.35629 | 49345.8 |
| 809 | $\overline{\boldsymbol{u}} \sim \psi_{ev}+\psi_{ac}\cdot\psi_{ce}+\psi_{ac}\cdot\psi_{ev}+\psi_{ac}\cdot\psi_{cv}+\psi_{ce}\cdot\psi_{cv}$ | 0.163032 | 0.35619 | 49350.6 |
| 810 | $\overline{\boldsymbol{u}} \sim \psi_{ce}+\psi_{cv}+\psi_{ac}\cdot\psi_{ev}+\psi_{ce}\cdot\psi_{cv}+\psi_{ev}\cdot\psi_{cv}$ | 0.120399 | 0.35496 | 49408.5 |
| 811 | $\overline{\boldsymbol{u}} \sim \psi_{ev}+\psi_{ac}\cdot\psi_{ev}+\psi_{ac}\cdot\psi_{cv}+\psi_{ce}\cdot\psi_{cv}$ | 0.159255 | 0.35482 | 49413.9 |
| 812 | $\overline{\boldsymbol{u}} \sim \psi_{ev}+\psi_{ac}\cdot\psi_{cv}+\psi_{ev}\cdot\psi_{cv}$ | 0.118601 | 0.35465 | 49420.9 |
| 813 | $\overline{\boldsymbol{u}} \sim \psi_{ac}+\psi_{ev}+\psi_{ac}\cdot\psi_{ev}+\psi_{ac}\cdot\psi_{cv}$ | 0.199247 | 0.35441 | 49433.5 |
| 814 | $\overline{\boldsymbol{u}} \sim \psi_{cv}+\psi_{ac}\cdot\psi_{ce}+\psi_{ac}\cdot\psi_{ev}+\psi_{ce}\cdot\psi_{cv}+\psi_{ev}\cdot\psi_{cv}$ | 0.115773 | 0.3544 | 49435.1 |
| 815 | $\overline{\boldsymbol{u}} \sim \psi_{ce}+\psi_{cv}+\psi_{ac}\cdot\psi_{ev}+\psi_{ev}\cdot\psi_{cv}$ | 0.118301 | 0.35407 | 49449.5 |
| 816 | $\overline{\boldsymbol{u}} \sim \psi_{ac}+\psi_{ev}+\psi_{ac}\cdot\psi_{cv}$ | 0.196291 | 0.35382 | 49460.3 |
| 817 | $\overline{\boldsymbol{u}} \sim \psi_{ac}+\psi_{cv}+\psi_{ce}\cdot\psi_{cv}$ | 0.189929 | 0.35331 | 49484.5 |
| 818 | $\overline{\boldsymbol{u}} \sim \psi_{ev}+\psi_{ac}\cdot\psi_{ce}+\psi_{ac}\cdot\psi_{ev}+\psi_{ac}\cdot\psi_{cv}$ | 0.166308 | 0.35319 | 49490.7 |
| 819 | $\overline{\boldsymbol{u}} \sim \psi_{cv}+\psi_{ac}\cdot\psi_{ev}+\psi_{ce}\cdot\psi_{ev}+\psi_{ce}\cdot\psi_{cv}+\psi_{ev}\cdot\psi_{cv}$ | 0.129608 | 0.35176 | 49559.1 |
| 820 | $\overline{\boldsymbol{u}} \sim \psi_{ac}+\psi_{ac}\cdot\psi_{ev}+\psi_{ac}\cdot\psi_{cv}$ | 0.200819 | 0.35167 | 49561.4 |
| 821 | $\overline{\boldsymbol{u}} \sim \psi_{ev}+\psi_{cv}+\psi_{ac}\cdot\psi_{ev}+\psi_{ev}\cdot\psi_{cv}$ | 0.152077 | 0.35129 | 49580.1 |
| 822 | $\overline{\boldsymbol{u}} \sim \psi_{ev}+\psi_{ac}\cdot\psi_{ev}+\psi_{ev}\cdot\psi_{cv}$ | 0.151854 | 0.35096 | 49594.6 |
| 823 | $\overline{\boldsymbol{u}} \sim \psi_{ev}+\psi_{ac}\cdot\psi_{ce}+\psi_{ac}\cdot\psi_{ev}+\psi_{ce}\cdot\psi_{cv}$ | 0.172311 | 0.35089 | 49598.9 |
| 824 | $\overline{\boldsymbol{u}} \sim \psi_{ev}+\psi_{ac}\cdot\psi_{ce}+\psi_{ac}\cdot\psi_{ev}$ | 0.174094 | 0.35072 | 49606.1 |
| 825 | $\overline{\boldsymbol{u}} \sim \psi_{ac}\cdot\psi_{ev}+\psi_{ce}\cdot\psi_{ev}+\psi_{ce}\cdot\psi_{cv}+\psi_{ev}\cdot\psi_{cv}$ | 0.129877 | 0.34901 | 49686.9 |
| 826 | $\overline{\boldsymbol{u}} \sim \psi_{ac}+\psi_{ev}+\psi_{ac}\cdot\psi_{ev}$ | 0.261164 | 0.34716 | 49772.2 |
| 827 | $\overline{\boldsymbol{u}} \sim \psi_{ev}+\psi_{cv}+\psi_{ac}\cdot\psi_{ev}$ | 0.152098 | 0.3471 | 49775 |
| 828 | $\overline{\boldsymbol{u}} \sim \psi_{ac}+\psi_{ev}$ | 0.254823 | 0.34646 | 49804.1 |
| 829 | $\overline{\boldsymbol{u}} \sim \psi_{ac}+\psi_{ac}\cdot\psi_{ev}$ | 0.252451 | 0.34443 | 49898.3 |
| 830 | $\overline{\boldsymbol{u}} \sim \psi_{cv}+\psi_{ac}\cdot\psi_{ev}+\psi_{ce}\cdot\psi_{cv}+\psi_{ev}\cdot\psi_{cv}$ | 0.124965 | 0.34281 | 49975.7 |
| 831 | $\overline{\boldsymbol{u}} \sim \psi_{ac}\cdot\psi_{ce}+\psi_{ac}\cdot\psi_{ev}+\psi_{ce}\cdot\psi_{cv}+\psi_{ev}\cdot\psi_{cv}$ | 0.109944 | 0.34142 | 50040.2 |
| 832 | $\overline{\boldsymbol{u}} \sim \psi_{ac}+\psi_{ce}+\psi_{ac}\cdot\psi_{cv}$ | 0.166626 | 0.3379 | 50201.4 |
| 833 | $\overline{\boldsymbol{u}} \sim \psi_{ac}+\psi_{ce}$ | 0.196836 | 0.33117 | 50508.4 |

| | | | | |
|---|---|---|---|---|
| 834 | $\overline{\boldsymbol{u}} \sim \psi_{ev}+\psi_{ac}\cdot\psi_{ev}+\psi_{ce}\cdot\psi_{cv}$ | 0.186806 | 0.32957 | 50582.1 |
| 835 | $\overline{\boldsymbol{u}} \sim \psi_{ce}+\psi_{cv}+\psi_{ac}\cdot\psi_{ev}+\psi_{ce}\cdot\psi_{ev}+\psi_{ce}\cdot\psi_{cv}$ | 0.123047 | 0.3277 | 50668.5 |
| 836 | $\overline{\boldsymbol{u}} \sim \psi_{ce}+\psi_{cv}+\psi_{ac}\cdot\psi_{ev}+\psi_{ce}\cdot\psi_{ev}$ | 0.120637 | 0.3261 | 50740 |
| 837 | $\overline{\boldsymbol{u}} \sim \psi_{ev}+\psi_{ac}\cdot\psi_{ce}+\psi_{ac}\cdot\psi_{cv}+\psi_{ce}\cdot\psi_{ev}+\psi_{ce}\cdot\psi_{cv}$ | 0.124585 | 0.32582 | 50753.8 |
| 838 | $\overline{\boldsymbol{u}} \sim \psi_{ce}+\psi_{ac}\cdot\psi_{ev}+\psi_{ac}\cdot\psi_{cv}+\psi_{ce}\cdot\psi_{ev}$ | 0.128947 | 0.32302 | 50879 |
| 839 | $\overline{\boldsymbol{u}} \sim \psi_{ce}+\psi_{ev}+\psi_{ac}\cdot\psi_{cv}+\psi_{ce}\cdot\psi_{ev}$ | 0.230799 | 0.3216 | 50942.7 |
| 840 | $\overline{\boldsymbol{u}} \sim \psi_{ce}+\psi_{ac}\cdot\psi_{ev}+\psi_{ce}\cdot\psi_{ev}+\psi_{ce}\cdot\psi_{cv}$ | 0.120226 | 0.3214 | 50951.6 |
| 841 | $\overline{\boldsymbol{u}} \sim \psi_{ce}+\psi_{ev}+\psi_{ac}\cdot\psi_{cv}$ | 0.22789 | 0.32131 | 50954.9 |
| 842 | $\overline{\boldsymbol{u}} \sim \psi_{ac}+\psi_{cv}+\psi_{ac}\cdot\psi_{ce}+\psi_{ce}\cdot\psi_{ev}$ | 0.171483 | 0.31911 | 51054.1 |
| 843 | $\overline{\boldsymbol{u}} \sim \psi_{ce}+\psi_{ac}\cdot\psi_{ce}+\psi_{ac}\cdot\psi_{ev}+\psi_{ac}\cdot\psi_{cv}+\psi_{ce}\cdot\psi_{cv}$ | 0.0936399 | 0.31888 | 51065.4 |
| 844 | $\overline{\boldsymbol{u}} \sim \psi_{ce}+\psi_{ac}\cdot\psi_{ev}+\psi_{ac}\cdot\psi_{cv}+\psi_{ce}\cdot\psi_{cv}$ | 0.0977306 | 0.3175 | 51126.2 |
| 845 | $\overline{\boldsymbol{u}} \sim \psi_{ce}+\psi_{ac}\cdot\psi_{ev}+\psi_{ce}\cdot\psi_{cv}+\psi_{ev}\cdot\psi_{cv}$ | 0.124939 | 0.31738 | 51131.4 |
| 846 | $\overline{\boldsymbol{u}} \sim \psi_{ce}+\psi_{ac}\cdot\psi_{ev}+\psi_{ce}\cdot\psi_{ev}$ | 0.124891 | 0.31688 | 51152.9 |
| 847 | $\overline{\boldsymbol{u}} \sim \psi_{ac}\cdot\psi_{ev}+\psi_{ce}\cdot\psi_{cv}+\psi_{ev}\cdot\psi_{cv}$ | 0.124968 | 0.31619 | 51183.5 |
| 848 | $\overline{\boldsymbol{u}} \sim \psi_{ev}+\psi_{ac}\cdot\psi_{cv}+\psi_{ce}\cdot\psi_{ev}+\psi_{ce}\cdot\psi_{cv}$ | 0.116501 | 0.3151 | 51233.1 |
| 849 | $\overline{\boldsymbol{u}} \sim \psi_{ac}\cdot\psi_{cv}+\psi_{ev}\cdot\psi_{cv}$ | 0.117102 | 0.31165 | 51383.9 |
| 850 | $\overline{\boldsymbol{u}} \sim \psi_{ev}+\psi_{ac}\cdot\psi_{ev}+\psi_{ac}\cdot\psi_{cv}$ | 0.156073 | 0.30714 | 51583.9 |
| 851 | $\overline{\boldsymbol{u}} \sim \psi_{ev}+\psi_{ac}\cdot\psi_{ev}$ | 0.162761 | 0.30554 | 51653.3 |
| 852 | $\overline{\boldsymbol{u}} \sim \psi_{ce}+\psi_{ev}+\psi_{cv}+\psi_{ce}\cdot\psi_{ev}+\psi_{ce}\cdot\psi_{cv}$ | 0.395263 | 0.3044 | 51705.9 |
| 853 | $\overline{\boldsymbol{u}} \sim \psi_{ce}+\psi_{ev}+\psi_{cv}+\psi_{ce}\cdot\psi_{ev}+\psi_{ce}\cdot\psi_{cv}+\psi_{ev}\cdot\psi_{cv}$ | 0.395268 | 0.30438 | 51707.9 |
| 854 | $\overline{\boldsymbol{u}} \sim \psi_{ce}+\psi_{ev}+\psi_{cv}+\psi_{ce}\cdot\psi_{cv}$ | 0.387953 | 0.30401 | 51722 |
| 855 | $\overline{\boldsymbol{u}} \sim \psi_{ce}+\psi_{ev}+\psi_{cv}+\psi_{ce}\cdot\psi_{cv}+\psi_{ev}\cdot\psi_{cv}$ | 0.388 | 0.30399 | 51724 |
| 856 | $\overline{\boldsymbol{u}} \sim \psi_{ce}+\psi_{ev}+\psi_{ce}\cdot\psi_{ev}+\psi_{ce}\cdot\psi_{cv}+\psi_{ev}\cdot\psi_{cv}$ | 0.383037 | 0.30158 | 51829.2 |
| 857 | $\overline{\boldsymbol{u}} \sim \psi_{ce}+\psi_{ev}+\psi_{ce}\cdot\psi_{cv}+\psi_{ev}\cdot\psi_{cv}$ | 0.376238 | 0.30113 | 51847.9 |
| 858 | $\overline{\boldsymbol{u}} \sim \psi_{ce}+\psi_{ev}+\psi_{cv}+\psi_{ce}\cdot\psi_{ev}$ | 0.403085 | 0.29943 | 51922 |
| 859 | $\overline{\boldsymbol{u}} \sim \psi_{ce}+\psi_{ev}+\psi_{cv}+\psi_{ce}\cdot\psi_{ev}+\psi_{ev}\cdot\psi_{cv}$ | 0.403094 | 0.2994 | 51924 |
| 860 | $\overline{\boldsymbol{u}} \sim \psi_{ce}+\psi_{ev}+\psi_{ce}\cdot\psi_{ev}+\psi_{ev}\cdot\psi_{cv}$ | 0.387385 | 0.29919 | 51932.3 |

| | | | | |
|---|---|---|---|---|
| 861 | $\overline{\boldsymbol{u}} \sim \psi_{ce}+\psi_{ac}\cdot\psi_{cv}+\psi_{ce}\cdot\psi_{ev}$ | 0.159699 | 0.29905 | 51937.1 |
| 862 | $\overline{\boldsymbol{u}} \sim \psi_{ce}+\psi_{ev}+\psi_{cv}$ | 0.395198 | 0.29902 | 51938.4 |
| 863 | $\overline{\boldsymbol{u}} \sim \psi_{cv}+\psi_{ac}\cdot\psi_{ce}+\psi_{ac}\cdot\psi_{ev}+\psi_{ce}\cdot\psi_{ev}+\psi_{ev}\cdot\psi_{cv}$ | 0.124672 | 0.29902 | 51940.8 |
| 864 | $\overline{\boldsymbol{u}} \sim \psi_{cv}+\psi_{ac}\cdot\psi_{ce}+\psi_{ac}\cdot\psi_{ev}+\psi_{ev}\cdot\psi_{cv}$ | 0.125103 | 0.29901 | 51940 |
| 865 | $\overline{\boldsymbol{u}} \sim \psi_{ce}+\psi_{ev}+\psi_{cv}+\psi_{ev}\cdot\psi_{cv}$ | 0.395251 | 0.299 | 51940.4 |
| 866 | $\overline{\boldsymbol{u}} \sim \psi_{ce}+\psi_{ev}+\psi_{ev}\cdot\psi_{cv}$ | 0.380208 | 0.29877 | 51949.5 |
| 867 | $\overline{\boldsymbol{u}} \sim \psi_{ce}+\psi_{ev}+\psi_{ce}\cdot\psi_{ev}+\psi_{ce}\cdot\psi_{cv}$ | 0.439013 | 0.29825 | 51973 |
| 868 | $\overline{\boldsymbol{u}} \sim \psi_{ce}+\psi_{ev}+\psi_{ce}\cdot\psi_{ev}$ | 0.396488 | 0.2982 | 51974 |
| 869 | $\overline{\boldsymbol{u}} \sim \psi_{ce}+\psi_{ev}+\psi_{ce}\cdot\psi_{cv}$ | 0.419727 | 0.29784 | 51989.8 |
| 870 | $\overline{\boldsymbol{u}} \sim \psi_{ce}+\psi_{ev}$ | 0.388734 | 0.2978 | 51990.4 |
| 871 | $\overline{\boldsymbol{u}} \sim \psi_{ev}+\psi_{cv}+\psi_{ce}\cdot\psi_{ev}+\psi_{ce}\cdot\psi_{cv}$ | 0.239824 | 0.29691 | 52031.3 |
| 872 | $\overline{\boldsymbol{u}} \sim \psi_{ev}+\psi_{cv}+\psi_{ce}\cdot\psi_{ev}+\psi_{ce}\cdot\psi_{cv}+\psi_{ev}\cdot\psi_{cv}$ | 0.239869 | 0.29689 | 52033.1 |
| 873 | $\overline{\boldsymbol{u}} \sim \psi_{ac}+\psi_{cv}+\psi_{ac}\cdot\psi_{ce}$ | 0.170567 | 0.29674 | 52037.6 |
| 874 | $\overline{\boldsymbol{u}} \sim \psi_{ev}+\psi_{ac}\cdot\psi_{ce}+\psi_{ac}\cdot\psi_{cv}+\psi_{ce}\cdot\psi_{ev}$ | 0.159758 | 0.2954 | 52096.3 |
| 875 | $\overline{\boldsymbol{u}} \sim \psi_{ev}+\psi_{ac}\cdot\psi_{cv}+\psi_{ce}\cdot\psi_{ev}$ | 0.158048 | 0.29504 | 52110.9 |
| 876 | $\overline{\boldsymbol{u}} \sim \psi_{ev}+\psi_{cv}+\psi_{ac}\cdot\psi_{ce}+\psi_{ce}\cdot\psi_{cv}$ | 0.213127 | 0.29436 | 52141.5 |
| 877 | $\overline{\boldsymbol{u}} \sim \psi_{ev}+\psi_{cv}+\psi_{ac}\cdot\psi_{ce}+\psi_{ce}\cdot\psi_{cv}+\psi_{ev}\cdot\psi_{cv}$ | 0.213133 | 0.29433 | 52143.5 |
| 878 | $\overline{\boldsymbol{u}} \sim \psi_{ev}+\psi_{cv}+\psi_{ce}\cdot\psi_{cv}$ | 0.22314 | 0.29426 | 52144.7 |
| 879 | $\overline{\boldsymbol{u}} \sim \psi_{ev}+\psi_{cv}+\psi_{ce}\cdot\psi_{cv}+\psi_{ev}\cdot\psi_{cv}$ | 0.223146 | 0.29424 | 52146.7 |
| 880 | $\overline{\boldsymbol{u}} \sim \psi_{ce}+\psi_{cv}+\psi_{ce}\cdot\psi_{ev}+\psi_{ce}\cdot\psi_{cv}+\psi_{ev}\cdot\psi_{cv}$ | 0.227205 | 0.29281 | 52209.3 |
| 881 | $\overline{\boldsymbol{u}} \sim \psi_{ce}+\psi_{ce}\cdot\psi_{ev}+\psi_{ce}\cdot\psi_{cv}+\psi_{ev}\cdot\psi_{cv}$ | 0.225809 | 0.2928 | 52208.6 |
| 882 | $\overline{\boldsymbol{u}} \sim \psi_{ac}+\psi_{ac}\cdot\psi_{cv}+\psi_{ce}\cdot\psi_{ev}+\psi_{ce}\cdot\psi_{cv}$ | 0.143507 | 0.28994 | 52331.4 |
| 883 | $\overline{\boldsymbol{u}} \sim \psi_{ac}+\psi_{ac}\cdot\psi_{ce}+\psi_{ac}\cdot\psi_{cv}+\psi_{ce}\cdot\psi_{ev}+\psi_{ce}\cdot\psi_{cv}$ | 0.143601 | 0.28992 | 52333.4 |
| 884 | $\overline{\boldsymbol{u}} \sim \psi_{ev}+\psi_{ac}\cdot\psi_{ce}+\psi_{ce}\cdot\psi_{ev}+\psi_{ce}\cdot\psi_{cv}+\psi_{ev}\cdot\psi_{cv}$ | 0.205698 | 0.28807 | 52412.6 |
| 885 | $\overline{\boldsymbol{u}} \sim \psi_{ev}+\psi_{ce}\cdot\psi_{ev}+\psi_{ce}\cdot\psi_{cv}+\psi_{ev}\cdot\psi_{cv}$ | 0.220583 | 0.28794 | 52417.2 |
| 886 | $\overline{\boldsymbol{u}} \sim \psi_{ce}+\psi_{cv}+\psi_{ce}\cdot\psi_{ev}+\psi_{ev}\cdot\psi_{cv}$ | 0.227793 | 0.2878 | 52423.2 |
| 887 | $\overline{\boldsymbol{u}} \sim \psi_{ce}+\psi_{ac}\cdot\psi_{ce}+\psi_{ac}\cdot\psi_{cv}+\psi_{ce}\cdot\psi_{cv}$ | 0.0736028 | 0.28701 | 52456.9 |

| | | | | |
|---|---|---|---|---|
| 888 | $\overline{\boldsymbol{u}} \sim \psi_{ev}+\psi_{ac}\cdot\psi_{ce}+\psi_{ac}\cdot\psi_{cv}+\psi_{ce}\cdot\psi_{cv}$ | 0.149086 | 0.28517 | 52535.3 |
| 889 | $\overline{\boldsymbol{u}} \sim \psi_{ev}+\psi_{ac}\cdot\psi_{ce}+\psi_{ce}\cdot\psi_{cv}+\psi_{ev}\cdot\psi_{cv}$ | 0.178235 | 0.28312 | 52622.3 |
| 890 | $\overline{\boldsymbol{u}} \sim \psi_{ce}+\psi_{cv}+\psi_{ce}\cdot\psi_{cv}+\psi_{ev}\cdot\psi_{cv}$ | 0.198368 | 0.28195 | 52672.2 |
| 891 | $\overline{\boldsymbol{u}} \sim \psi_{ce}+\psi_{ce}\cdot\psi_{ev}+\psi_{ev}\cdot\psi_{cv}$ | 0.212244 | 0.2815 | 52690 |
| 892 | $\overline{\boldsymbol{u}} \sim \psi_{ce}+\psi_{cv}+\psi_{ce}\cdot\psi_{ev}+\psi_{ce}\cdot\psi_{cv}$ | 0.192742 | 0.28018 | 52746.9 |
| 893 | $\overline{\boldsymbol{u}} \sim \psi_{ev}+\psi_{cv}+\psi_{ac}\cdot\psi_{ce}+\psi_{ce}\cdot\psi_{ev}$ | 0.164196 | 0.27949 | 52776.1 |
| 894 | $\overline{\boldsymbol{u}} \sim \psi_{ev}+\psi_{cv}+\psi_{ac}\cdot\psi_{ce}+\psi_{ce}\cdot\psi_{ev}+\psi_{ev}\cdot\psi_{cv}$ | 0.164228 | 0.27948 | 52777.7 |
| 895 | $\overline{\boldsymbol{u}} \sim \psi_{ev}+\psi_{ce}\cdot\psi_{cv}+\psi_{ev}\cdot\psi_{cv}$ | 0.201116 | 0.27875 | 52806.6 |
| 896 | $\overline{\boldsymbol{u}} \sim \psi_{ev}+\psi_{ac}\cdot\psi_{ce}+\psi_{ce}\cdot\psi_{ev}+\psi_{ev}\cdot\psi_{cv}$ | 0.164401 | 0.27869 | 52810.2 |
| 897 | $\overline{\boldsymbol{u}} \sim \psi_{ce}+\psi_{ce}\cdot\psi_{cv}+\psi_{ev}\cdot\psi_{cv}$ | 0.186626 | 0.27811 | 52833.5 |
| 898 | $\overline{\boldsymbol{u}} \sim \psi_{ce}+\psi_{cv}+\psi_{ev}\cdot\psi_{cv}$ | 0.198496 | 0.27691 | 52884.2 |
| 899 | $\overline{\boldsymbol{u}} \sim \psi_{ce}+\psi_{ac}\cdot\psi_{cv}+\psi_{ce}\cdot\psi_{cv}$ | 0.0868739 | 0.27643 | 52904.5 |
| 900 | $\overline{\boldsymbol{u}} \sim \psi_{cv}+\psi_{ac}\cdot\psi_{ev}+\psi_{ce}\cdot\psi_{ev}+\psi_{ev}\cdot\psi_{cv}$ | 0.110394 | 0.27598 | 52924.4 |
| 901 | $\overline{\boldsymbol{u}} \sim \psi_{cv}+\psi_{ac}\cdot\psi_{ce}+\psi_{ce}\cdot\psi_{ev}+\psi_{ce}\cdot\psi_{cv}+\psi_{ev}\cdot\psi_{cv}$ | 0.170084 | 0.27545 | 52947.3 |
| 902 | $\overline{\boldsymbol{u}} \sim \psi_{ce}+\psi_{cv}+\psi_{ce}\cdot\psi_{ev}$ | 0.19299 | 0.27521 | 52955.7 |
| 903 | $\overline{\boldsymbol{u}} \sim \psi_{ev}+\psi_{ac}\cdot\psi_{ce}+\psi_{ce}\cdot\psi_{ev}+\psi_{ce}\cdot\psi_{cv}$ | 0.173168 | 0.27515 | 52959.2 |
| 904 | $\overline{\boldsymbol{u}} \sim \psi_{ev}+\psi_{ac}\cdot\psi_{ce}+\psi_{ce}\cdot\psi_{ev}$ | 0.16945 | 0.27503 | 52963 |
| 905 | $\overline{\boldsymbol{u}} \sim \psi_{ac}\cdot\psi_{ce}+\psi_{ce}\cdot\psi_{ev}+\psi_{ce}\cdot\psi_{cv}+\psi_{ev}\cdot\psi_{cv}$ | 0.169547 | 0.27493 | 52968.1 |
| 906 | $\overline{\boldsymbol{u}} \sim \psi_{cv}+\psi_{ce}\cdot\psi_{ev}+\psi_{ce}\cdot\psi_{cv}+\psi_{ev}\cdot\psi_{cv}$ | 0.178862 | 0.27453 | 52985 |
| 907 | $\overline{\boldsymbol{u}} \sim \psi_{ac}\cdot\psi_{ce}+\psi_{ce}\cdot\psi_{cv}+\psi_{ev}\cdot\psi_{cv}$ | 0.173276 | 0.27409 | 53002.5 |
| 908 | $\overline{\boldsymbol{u}} \sim \psi_{ce}+\psi_{ce}\cdot\psi_{ev}$ | 0.192004 | 0.27407 | 53002.3 |
| 909 | $\overline{\boldsymbol{u}} \sim \psi_{cv}+\psi_{ac}\cdot\psi_{ce}+\psi_{ce}\cdot\psi_{cv}+\psi_{ev}\cdot\psi_{cv}$ | 0.172922 | 0.27407 | 53004.2 |
| 910 | $\overline{\boldsymbol{u}} \sim \psi_{ce}+\psi_{ce}\cdot\psi_{ev}+\psi_{ce}\cdot\psi_{cv}$ | 0.192036 | 0.27405 | 53004.2 |
| 911 | $\overline{\boldsymbol{u}} \sim \psi_{cv}+\psi_{ce}\cdot\psi_{cv}+\psi_{ev}\cdot\psi_{cv}$ | 0.178505 | 0.27379 | 53015 |
| 912 | $\overline{\boldsymbol{u}} \sim \psi_{ce}\cdot\psi_{ev}+\psi_{ce}\cdot\psi_{cv}+\psi_{ev}\cdot\psi_{cv}$ | 0.179954 | 0.27371 | 53018.4 |
| 913 | $\overline{\boldsymbol{u}} \sim \psi_{ce}\cdot\psi_{cv}+\psi_{ev}\cdot\psi_{cv}$ | 0.17935 | 0.27369 | 53018.3 |
| 914 | $\overline{\boldsymbol{u}} \sim \psi_{ac}+\psi_{ac}\cdot\psi_{ce}+\psi_{ac}\cdot\psi_{cv}+\psi_{ce}\cdot\psi_{ev}$ | 0.16353 | 0.27301 | 53048.9 |

| | | | | |
|---|---|---|---|---|
| 915 | $\overline{\boldsymbol{u}} \sim \psi_{ac}+\psi_{ac}\cdot\psi_{ce}+\psi_{ac}\cdot\psi_{cv}+\psi_{ce}\cdot\psi_{cv}$ | 0.129261 | 0.27196 | 53092.6 |
| 916 | $\overline{\boldsymbol{u}} \sim \psi_{ev}+\psi_{ce}\cdot\psi_{ev}+\psi_{ce}\cdot\psi_{cv}$ | 0.207824 | 0.27184 | 53096.9 |
| 917 | $\overline{\boldsymbol{u}} \sim \psi_{ev}+\psi_{cv}+\psi_{ac}\cdot\psi_{ce}$ | 0.155084 | 0.27158 | 53107.7 |
| 918 | $\overline{\boldsymbol{u}} \sim \psi_{ev}+\psi_{cv}+\psi_{ac}\cdot\psi_{ce}+\psi_{ev}\cdot\psi_{cv}$ | 0.155087 | 0.27156 | 53109.7 |
| 919 | $\overline{\boldsymbol{u}} \sim \psi_{ev}+\psi_{ac}\cdot\psi_{ce}+\psi_{ac}\cdot\psi_{cv}$ | 0.163988 | 0.27127 | 53120.5 |
| 920 | $\overline{\boldsymbol{u}} \sim \psi_{ev}+\psi_{ac}\cdot\psi_{ce}+\psi_{ev}\cdot\psi_{cv}$ | 0.155152 | 0.27026 | 53162.9 |
| 921 | $\overline{\boldsymbol{u}} \sim \psi_{ev}+\psi_{cv}+\psi_{ce}\cdot\psi_{ev}+\psi_{ev}\cdot\psi_{cv}$ | 0.191848 | 0.26939 | 53200.1 |
| 922 | $\overline{\boldsymbol{u}} \sim \psi_{ev}+\psi_{cv}+\psi_{ce}\cdot\psi_{ev}$ | 0.191813 | 0.26938 | 53199.6 |
| 923 | $\overline{\boldsymbol{u}} \sim \psi_{ev}+\psi_{ce}\cdot\psi_{ev}+\psi_{ev}\cdot\psi_{cv}$ | 0.19114 | 0.26905 | 53213.1 |
| 924 | $\overline{\boldsymbol{u}} \sim \psi_{ac}+\psi_{ac}\cdot\psi_{cv}+\psi_{ce}\cdot\psi_{cv}$ | 0.144402 | 0.26891 | 53219.2 |
| 925 | $\overline{\boldsymbol{u}} \sim \psi_{ev}+\psi_{ce}\cdot\psi_{ev}$ | 0.190591 | 0.26806 | 53253.2 |
| 926 | $\overline{\boldsymbol{u}} \sim \psi_{ac}+\psi_{cv}+\psi_{ce}\cdot\psi_{ev}$ | 0.157314 | 0.26757 | 53275 |
| 927 | $\overline{\boldsymbol{u}} \sim \psi_{ac}+\psi_{cv}$ | 0.156383 | 0.26702 | 53296.8 |
| 928 | $\overline{\boldsymbol{u}} \sim \psi_{ac}+\psi_{ac}\cdot\psi_{ce}+\psi_{ce}\cdot\psi_{ev}+\psi_{ce}\cdot\psi_{cv}$ | 0.175381 | 0.26407 | 53420.8 |
| 929 | $\overline{\boldsymbol{u}} \sim \psi_{ac}+\psi_{ac}\cdot\psi_{ce}+\psi_{ce}\cdot\psi_{ev}$ | 0.178069 | 0.26362 | 53438.8 |
| 930 | $\overline{\boldsymbol{u}} \sim \psi_{ev}+\psi_{ac}\cdot\psi_{ce}$ | 0.16066 | 0.26194 | 53506.7 |
| 931 | $\overline{\boldsymbol{u}} \sim \psi_{ev}+\psi_{ac}\cdot\psi_{ce}+\psi_{ce}\cdot\psi_{cv}$ | 0.160813 | 0.26192 | 53508.7 |
| 932 | $\overline{\boldsymbol{u}} \sim \psi_{ac}+\psi_{ac}\cdot\psi_{ce}+\psi_{ac}\cdot\psi_{cv}$ | 0.158557 | 0.25463 | 53808 |
| 933 | $\overline{\boldsymbol{u}} \sim \psi_{ac}\cdot\psi_{ce}+\psi_{ac}\cdot\psi_{ev}+\psi_{ce}\cdot\psi_{ev}+\psi_{ev}\cdot\psi_{cv}$ | 0.12835 | 0.25211 | 53911.9 |
| 934 | $\overline{\boldsymbol{u}} \sim \psi_{cv}+\psi_{ac}\cdot\psi_{ce}+\psi_{ev}\cdot\psi_{cv}$ | 0.144586 | 0.2521 | 53911.4 |
| 935 | $\overline{\boldsymbol{u}} \sim \psi_{cv}+\psi_{ac}\cdot\psi_{ce}+\psi_{ce}\cdot\psi_{ev}+\psi_{ev}\cdot\psi_{cv}$ | 0.144559 | 0.25208 | 53913 |
| 936 | $\overline{\boldsymbol{u}} \sim \psi_{cv}+\psi_{ac}\cdot\psi_{ce}+\psi_{ac}\cdot\psi_{ev}+\psi_{ce}\cdot\psi_{ev}+\psi_{ce}\cdot\psi_{cv}$ | 0.138708 | 0.24738 | 54104.6 |
| 937 | $\overline{\boldsymbol{u}} \sim \psi_{ac}+\psi_{ac}\cdot\psi_{ce}+\psi_{ce}\cdot\psi_{cv}$ | 0.174644 | 0.24634 | 54144.8 |
| 938 | $\overline{\boldsymbol{u}} \sim \psi_{ac}+\psi_{ac}\cdot\psi_{ce}$ | 0.177771 | 0.24604 | 54156 |
| 939 | $\overline{\boldsymbol{u}} \sim \psi_{cv}+\psi_{ac}\cdot\psi_{ce}+\psi_{ce}\cdot\psi_{ev}+\psi_{ce}\cdot\psi_{cv}$ | 0.148826 | 0.24253 | 54299.3 |
| 940 | $\overline{\boldsymbol{u}} \sim \psi_{cv}+\psi_{ac}\cdot\psi_{ev}+\psi_{ce}\cdot\psi_{ev}+\psi_{ce}\cdot\psi_{cv}$ | 0.133627 | 0.24028 | 54389.7 |
| 941 | $\overline{\boldsymbol{u}} \sim \psi_{ac}\cdot\psi_{ce}+\psi_{ce}\cdot\psi_{ev}+\psi_{ev}\cdot\psi_{cv}$ | 0.138957 | 0.23873 | 54450.8 |

| | | | | |
|---|---|---|---|---|
| 942 | $\overline{\boldsymbol{u}} \sim \psi_{ce}+\psi_{ac}\cdot\psi_{ce}+\psi_{ac}\cdot\psi_{ev}+\psi_{ev}\cdot\psi_{cv}$ | 0.128571 | 0.23851 | 54460.4 |
| 943 | $\overline{\boldsymbol{u}} \sim \psi_{ce}+\psi_{ac}\cdot\psi_{ev}+\psi_{ev}\cdot\psi_{cv}$ | 0.14121 | 0.23712 | 54515 |
| 944 | $\overline{\boldsymbol{u}} \sim \psi_{ce}+\psi_{ac}\cdot\psi_{ce}+\psi_{ev}\cdot\psi_{cv}$ | 0.152745 | 0.23473 | 54610.3 |
| 945 | $\overline{\boldsymbol{u}} \sim \psi_{cv}+\psi_{ce}\cdot\psi_{ev}+\psi_{ce}\cdot\psi_{cv}$ | 0.151299 | 0.23434 | 54625.7 |
| 946 | $\overline{\boldsymbol{u}} \sim \psi_{ce}+\psi_{ev}\cdot\psi_{cv}$ | 0.216547 | 0.23289 | 54682.3 |
| 947 | $\overline{\boldsymbol{u}} \sim \psi_{ac}+\psi_{ce}\cdot\psi_{ev}+\psi_{ce}\cdot\psi_{cv}$ | 0.208344 | 0.23076 | 54767.9 |
| 948 | $\overline{\boldsymbol{u}} \sim \psi_{ac}+\psi_{ce}\cdot\psi_{cv}$ | 0.204657 | 0.22965 | 54810.5 |
| 949 | $\overline{\boldsymbol{u}} \sim \psi_{ac}\cdot\psi_{ce}+\psi_{ac}\cdot\psi_{ev}+\psi_{ev}\cdot\psi_{cv}$ | 0.124014 | 0.22804 | 54875.3 |
| 950 | $\overline{\boldsymbol{u}} \sim \psi_{ac}\cdot\psi_{ce}+\psi_{ev}\cdot\psi_{cv}$ | 0.132773 | 0.22591 | 54958.2 |
| 951 | $\overline{\boldsymbol{u}} \sim \psi_{ce}+\psi_{ac}\cdot\psi_{ev}+\psi_{ac}\cdot\psi_{cv}$ | 0.256067 | 0.22428 | 55023.3 |
| 952 | $\overline{\boldsymbol{u}} \sim \psi_{ac}+\psi_{ac}\cdot\psi_{cv}+\psi_{ce}\cdot\psi_{ev}$ | 0.143715 | 0.22385 | 55040.1 |
| 953 | $\overline{\boldsymbol{u}} \sim \psi_{ac}+\psi_{ac}\cdot\psi_{cv}$ | 0.143605 | 0.22234 | 55098.2 |
| 954 | $\overline{\boldsymbol{u}} \sim \psi_{cv}+\psi_{ac}\cdot\psi_{ev}+\psi_{ev}\cdot\psi_{cv}$ | 0.100092 | 0.22234 | 55099.2 |
| 955 | $\overline{\boldsymbol{u}} \sim \psi_{ev}+\psi_{ac}\cdot\psi_{cv}+\psi_{ce}\cdot\psi_{cv}$ | 0.418538 | 0.21895 | 55231.5 |
| 956 | $\overline{\boldsymbol{u}} \sim \psi_{ev}+\psi_{ce}\cdot\psi_{cv}$ | 0.430279 | 0.21891 | 55232.4 |
| 957 | $\overline{\boldsymbol{u}} \sim \psi_{ce}+\psi_{ac}\cdot\psi_{ce}+\psi_{ac}\cdot\psi_{cv}$ | 0.0146899 | 0.21869 | 55241.6 |
| 958 | $\overline{\boldsymbol{u}} \sim \psi_{ce}+\psi_{cv}+\psi_{ac}\cdot\psi_{ev}+\psi_{ce}\cdot\psi_{cv}$ | 0.861591 | 0.21794 | 55272.1 |
| 959 | $\overline{\boldsymbol{u}} \sim \psi_{ce}+\psi_{ac}\cdot\psi_{ev}+\psi_{ce}\cdot\psi_{cv}$ | 0.862595 | 0.21748 | 55288.9 |
| 960 | $\overline{\boldsymbol{u}} \sim \psi_{ac}\cdot\psi_{ce}+\psi_{ac}\cdot\psi_{ev}+\psi_{ac}\cdot\psi_{cv}+\psi_{ce}\cdot\psi_{ev}+\psi_{ce}\cdot\psi_{cv}$ | 0.138743 | 0.21635 | 55334.9 |
| 961 | $\overline{\boldsymbol{u}} \sim \psi_{ac}\cdot\psi_{ce}+\psi_{ac}\cdot\psi_{ev}+\psi_{ac}\cdot\psi_{cv}+\psi_{ce}\cdot\psi_{cv}$ | 0.136696 | 0.2159 | 55351.2 |
| 962 | $\overline{\boldsymbol{u}} \sim \psi_{ac}+\psi_{ce}\cdot\psi_{ev}$ | 0.170812 | 0.2154 | 55368.8 |
| 963 | $\overline{\boldsymbol{u}} \sim \psi_{ac}$ | 0.170157 | 0.21375 | 55431.8 |
| 964 | $\overline{\boldsymbol{u}} \sim \psi_{ce}+\psi_{cv}+\psi_{ac}\cdot\psi_{ev}$ | 0.865403 | 0.21278 | 55471.3 |
| 965 | $\overline{\boldsymbol{u}} \sim \psi_{ce}+\psi_{ac}\cdot\psi_{ev}$ | 0.862058 | 0.21215 | 55494.4 |
| 966 | $\overline{\boldsymbol{u}} \sim \psi_{cv}+\psi_{ce}\cdot\psi_{ev}+\psi_{ev}\cdot\psi_{cv}$ | 0.151122 | 0.21181 | 55508.6 |
| 967 | $\overline{\boldsymbol{u}} \sim \psi_{cv}+\psi_{ac}\cdot\psi_{ce}+\psi_{ac}\cdot\psi_{ev}+\psi_{ce}\cdot\psi_{ev}$ | 0.138629 | 0.20535 | 55758.4 |
| 968 | $\overline{\boldsymbol{u}} \sim \psi_{cv}+\psi_{ac}\cdot\psi_{ce}+\psi_{ce}\cdot\psi_{ev}$ | 0.137961 | 0.20528 | 55760 |

| | | | | |
|---|---|---|---|---|
| 969 | $\overline{\boldsymbol{u}} \sim \psi_{ac}{\cdot}\psi_{ce}{+}\psi_{ac}{\cdot}\psi_{ev}{+}\psi_{ac}{\cdot}\psi_{cv}{+}\psi_{ce}{\cdot}\psi_{ev}$ | 0.153756 | 0.19572 | 56124.8 |
| 970 | $\overline{\boldsymbol{u}} \sim \psi_{ac}{\cdot}\psi_{ce}{+}\psi_{ac}{\cdot}\psi_{cv}{+}\psi_{ce}{\cdot}\psi_{ev}{+}\psi_{ce}{\cdot}\psi_{cv}$ | 0.143405 | 0.19543 | 56136 |
| 971 | $\overline{\boldsymbol{u}} \sim \psi_{ce}{+}\psi_{ac}{\cdot}\psi_{cv}$ | 0.0963399 | 0.19173 | 56273.5 |
| 972 | $\overline{\boldsymbol{u}} \sim \psi_{ac}{\cdot}\psi_{ce}{+}\psi_{ac}{\cdot}\psi_{ev}{+}\psi_{ce}{\cdot}\psi_{ev}{+}\psi_{ce}{\cdot}\psi_{cv}$ | 0.14733 | 0.18858 | 56393.9 |
| 973 | $\overline{\boldsymbol{u}} \sim \psi_{ac}{\cdot}\psi_{ce}{+}\psi_{ac}{\cdot}\psi_{ev}{+}\psi_{ce}{\cdot}\psi_{ev}$ | 0.148151 | 0.18855 | 56394.3 |
| 974 | $\overline{\boldsymbol{u}} \sim \psi_{ac}{\cdot}\psi_{ce}{+}\psi_{ac}{\cdot}\psi_{cv}{+}\psi_{ce}{\cdot}\psi_{ev}$ | 0.147489 | 0.18834 | 56402 |
| 975 | $\overline{\boldsymbol{u}} \sim \psi_{cv}{+}\psi_{ac}{\cdot}\psi_{ce}{+}\psi_{ac}{\cdot}\psi_{ev}{+}\psi_{ce}{\cdot}\psi_{cv}$ | 0.205221 | 0.18671 | 56464.1 |
| 976 | $\overline{\boldsymbol{u}} \sim \psi_{ac}{\cdot}\psi_{ce}{+}\psi_{ac}{\cdot}\psi_{ev}{+}\psi_{ac}{\cdot}\psi_{cv}$ | 0.158962 | 0.18599 | 56490.2 |
| 977 | $\overline{\boldsymbol{u}} \sim \psi_{ac}{\cdot}\psi_{ce}{+}\psi_{ce}{\cdot}\psi_{ev}{+}\psi_{ce}{\cdot}\psi_{cv}$ | 0.143985 | 0.18572 | 56500 |
| 978 | $\overline{\boldsymbol{u}} \sim \psi_{ac}{\cdot}\psi_{ce}{+}\psi_{ce}{\cdot}\psi_{ev}$ | 0.14496 | 0.18561 | 56503.3 |
| 979 | $\overline{\boldsymbol{u}} \sim \psi_{cv}{+}\psi_{ac}{\cdot}\psi_{ev}{+}\psi_{ce}{\cdot}\psi_{cv}$ | 0.265356 | 0.18503 | 56525.9 |
| 980 | $\overline{\boldsymbol{u}} \sim \psi_{cv}{+}\psi_{ac}{\cdot}\psi_{ce}{+}\psi_{ac}{\cdot}\psi_{ev}$ | 0.15365 | 0.17182 | 57015.6 |
| 981 | $\overline{\boldsymbol{u}} \sim \psi_{ce}{+}\psi_{cv}{+}\psi_{ce}{\cdot}\psi_{cv}$ | 0.177581 | 0.17172 | 57019.2 |
| 982 | $\overline{\boldsymbol{u}} \sim \psi_{ce}{+}\psi_{cv}$ | 0.177627 | 0.16675 | 57200.3 |
| 983 | $\overline{\boldsymbol{u}} \sim \psi_{ac}{\cdot}\psi_{ce}{+}\psi_{ac}{\cdot}\psi_{ev}$ | 0.158778 | 0.16658 | 57206.4 |
| 984 | $\overline{\boldsymbol{u}} \sim \psi_{ac}{\cdot}\psi_{ce}{+}\psi_{ac}{\cdot}\psi_{ev}{+}\psi_{ce}{\cdot}\psi_{cv}$ | 0.159768 | 0.16658 | 57207.5 |
| 985 | $\overline{\boldsymbol{u}} \sim \psi_{ce}$ | 0.1733 | 0.16562 | 57240.4 |
| 986 | $\overline{\boldsymbol{u}} \sim \psi_{ce}{+}\psi_{ce}{\cdot}\psi_{cv}$ | 0.173352 | 0.1656 | 57242.4 |
| 987 | $\overline{\boldsymbol{u}} \sim \psi_{cv}{+}\psi_{ac}{\cdot}\psi_{ce}{+}\psi_{ce}{\cdot}\psi_{cv}$ | 0.142269 | 0.16426 | 57292.1 |
| 988 | $\overline{\boldsymbol{u}} \sim \psi_{cv}{+}\psi_{ce}{\cdot}\psi_{cv}$ | 0.148594 | 0.16385 | 57305.9 |
| 989 | $\overline{\boldsymbol{u}} \sim \psi_{ev}{+}\psi_{ac}{\cdot}\psi_{cv}$ | 0.218887 | 0.16311 | 57333 |
| 990 | $\overline{\boldsymbol{u}} \sim \psi_{ac}{\cdot}\psi_{ce}{+}\psi_{ac}{\cdot}\psi_{cv}{+}\psi_{ce}{\cdot}\psi_{cv}$ | 0.12547 | 0.15648 | 57574.2 |
| 991 | $\overline{\boldsymbol{u}} \sim \psi_{cv}{+}\psi_{ac}{\cdot}\psi_{ce}$ | 0.122747 | 0.1432 | 58048.7 |
| 992 | $\overline{\boldsymbol{u}} \sim \psi_{ac}{\cdot}\psi_{ce}{+}\psi_{ac}{\cdot}\psi_{cv}$ | 0.129373 | 0.14239 | 58077.6 |
| 993 | $\overline{\boldsymbol{u}} \sim \psi_{ev}{+}\psi_{cv}$ | 1.88438 | 0.13649 | 58286.3 |
| 994 | $\overline{\boldsymbol{u}} \sim \psi_{ev}{+}\psi_{cv}{+}\psi_{ev}{\cdot}\psi_{cv}$ | 1.8863 | 0.13647 | 58288.2 |
| 995 | $\overline{\boldsymbol{u}} \sim \psi_{ev}{+}\psi_{ev}{\cdot}\psi_{cv}$ | 0.588536 | 0.13484 | 58344.7 |

| | | | | |
|---|---|---|---|---|
| 996 | $\overline{\boldsymbol{u}} \sim \psi_{ev}$ | 2.00275 | 0.13424 | 58364.5 |
| 997 | $\overline{\boldsymbol{u}} \sim \psi_{ac}{\cdot}\psi_{ce}$ | 0.128582 | 0.13336 | 58395.4 |
| 998 | $\overline{\boldsymbol{u}} \sim \psi_{ac}{\cdot}\psi_{ce}+\psi_{ce}{\cdot}\psi_{cv}$ | 0.128379 | 0.13334 | 58397.3 |
| 999 | $\overline{\boldsymbol{u}} \sim \psi_{ac}{\cdot}\psi_{ev}+\psi_{ac}{\cdot}\psi_{cv}+\psi_{ce}{\cdot}\psi_{ev}+\psi_{ce}{\cdot}\psi_{cv}$ | 0.648982 | 0.13003 | 58515.4 |
| 1000 | $\overline{\boldsymbol{u}} \sim \psi_{ac}{\cdot}\psi_{ev}+\psi_{ce}{\cdot}\psi_{ev}+\psi_{ce}{\cdot}\psi_{cv}$ | 0.622069 | 0.1265 | 58637.7 |
| 1001 | $\overline{\boldsymbol{u}} \sim \psi_{ac}{\cdot}\psi_{ev}+\psi_{ac}{\cdot}\psi_{cv}+\psi_{ce}{\cdot}\psi_{cv}$ | 0.590805 | 0.12358 | 58739.3 |
| 1002 | $\overline{\boldsymbol{u}} \sim \psi_{ac}{\cdot}\psi_{ev}+\psi_{ce}{\cdot}\psi_{cv}$ | 0.566417 | 0.12283 | 58764.1 |
| 1003 | $\overline{\boldsymbol{u}} \sim \psi_{ac}{\cdot}\psi_{ev}+\psi_{ce}{\cdot}\psi_{ev}+\psi_{ev}{\cdot}\psi_{cv}$ | 0.130951 | 0.11329 | 59094.7 |
| 1004 | $\overline{\boldsymbol{u}} \sim \psi_{ce}{\cdot}\psi_{ev}+\psi_{ev}{\cdot}\psi_{cv}$ | 0.171193 | 0.11273 | 59112.7 |
| 1005 | $\overline{\boldsymbol{u}} \sim \psi_{cv}+\psi_{ev}{\cdot}\psi_{cv}$ | 0.202863 | 0.11152 | 59154.3 |
| 1006 | $\overline{\boldsymbol{u}} \sim \psi_{ac}{\cdot}\psi_{ev}+\psi_{ac}{\cdot}\psi_{cv}+\psi_{ce}{\cdot}\psi_{ev}$ | 0.3449 | 0.10744 | 59294.7 |
| 1007 | $\overline{\boldsymbol{u}} \sim \psi_{ac}{\cdot}\psi_{cv}+\psi_{ce}{\cdot}\psi_{ev}+\psi_{ce}{\cdot}\psi_{cv}$ | 0.246736 | 0.09568 | 59693.4 |
| 1008 | $\overline{\boldsymbol{u}} \sim \psi_{ce}{\cdot}\psi_{ev}+\psi_{ce}{\cdot}\psi_{cv}$ | 0.233743 | 0.09242 | 59802 |
| 1009 | $\overline{\boldsymbol{u}} \sim \psi_{ac}{\cdot}\psi_{ev}+\psi_{ev}{\cdot}\psi_{cv}$ | 0.0498846 | 0.08883 | 59922 |
| 1010 | $\overline{\boldsymbol{u}} \sim \psi_{ac}{\cdot}\psi_{cv}+\psi_{ce}{\cdot}\psi_{cv}$ | 0.276076 | 0.08612 | 60012.3 |
| 1011 | $\overline{\boldsymbol{u}} \sim \psi_{ce}{\cdot}\psi_{cv}$ | 0.258605 | 0.08597 | 60016.3 |
| 1012 | $\overline{\boldsymbol{u}} \sim \psi_{ev}{\cdot}\psi_{cv}$ | 0.218984 | 0.07215 | 60473.3 |
| 1013 | $\overline{\boldsymbol{u}} \sim \psi_{cv}+\psi_{ac}{\cdot}\psi_{ev}+\psi_{ce}{\cdot}\psi_{ev}$ | 0.680673 | 0.0667 | 60653.8 |
| 1014 | $\overline{\boldsymbol{u}} \sim \psi_{ac}{\cdot}\psi_{ev}+\psi_{ce}{\cdot}\psi_{ev}$ | 0.676916 | 0.06661 | 60655.5 |
| 1015 | $\overline{\boldsymbol{u}} \sim \psi_{ac}{\cdot}\psi_{ev}+\psi_{ac}{\cdot}\psi_{cv}$ | 0.555832 | 0.06123 | 60830.5 |
| 1016 | $\overline{\boldsymbol{u}} \sim \psi_{cv}+\psi_{ac}{\cdot}\psi_{ev}$ | 0.889376 | 0.05345 | 61081.8 |
| 1017 | $\overline{\boldsymbol{u}} \sim \psi_{ac}{\cdot}\psi_{ev}$ | 0.884849 | 0.05161 | 61140 |
| 1018 | $\overline{\boldsymbol{u}} \sim \psi_{ac}{\cdot}\psi_{cv}+\psi_{ce}{\cdot}\psi_{ev}$ | 0.0204519 | 0.0469 | 61291.8 |
| 1019 | $\overline{\boldsymbol{u}} \sim \psi_{ac}{\cdot}\psi_{cv}$ | 0.0197091 | 0.03986 | 61514.9 |
| 1020 | $\overline{\boldsymbol{u}} \sim \psi_{cv}+\psi_{ce}{\cdot}\psi_{ev}$ | 0.0232651 | 0.01116 | 62412.6 |
| 1021 | $\overline{\boldsymbol{u}} \sim \psi_{ce}{\cdot}\psi_{ev}$ | 0.021264 | 0.00905 | 62476.5 |
| 1022 | $\overline{\boldsymbol{u}} \sim \psi_{cv}$ | 0.0119828 | 0.00246 | 62678.4 |
