# Supplementary material for: Influence of interaction of cerebral fluids on ventricular deformation: A mathematical approach
Source: PLoS One. 2022 Feb 28;17(2):e0264395. doi: 10.1371/journal.pone.0264395 (PMC8884699; doi:10.1371/journal.pone.0264395)
Supplement: S3 File — The file provides information about linear regression models for volunteer 1, which were constructed when searching for bopt. For each regression model, the regression formula, the value of bopt, Radj.2 and the value of the Akaike information criterion (AIC) are given. (PDF) [file pone.0264395.s004.pdf]

Table 1: Regression results for volunteer 3

N	Formula	Optimal b	$R^2_{adj.}$	AIC
0	$\overline{u} \sim \psi_{ac}+\psi_{ce}+\psi_{ev}+\psi_{cv}+\psi_{ac}\cdot\psi_{ce}+\psi_{ac}\cdot\psi_{ev}+\psi_{ac}\cdot\psi_{cv}+\psi_{ce}\cdot\psi_{cv}+\psi_{ev}\cdot\psi_{cv}$	0.173365	0.91317	-10126.4
1	$\overline{u} \sim \psi_{ac}+\psi_{ce}+\psi_{ev}+\psi_{cv}+\psi_{ac}\cdot\psi_{ce}+\psi_{ac}\cdot\psi_{ev}+\psi_{ac}\cdot\psi_{cv}+\psi_{ce}\cdot\psi_{ev}+\psi_{ce}\cdot\psi_{cv}+\psi_{ev}\cdot\psi_{cv}$	0.173427	0.91317	-10125.6
2	$\overline{u} \sim \psi_{ac}+\psi_{ce}+\psi_{ev}+\psi_{cv}+\psi_{ac}\cdot\psi_{ce}+\psi_{ac}\cdot\psi_{ev}+\psi_{ac}\cdot\psi_{cv}+\psi_{ce}\cdot\psi_{ev}+\psi_{ce}\cdot\psi_{cv}$	0.173003	0.913	-10064.8
3	$\overline{u} \sim \psi_{ac}+\psi_{ce}+\psi_{ev}+\psi_{cv}+\psi_{ac}\cdot\psi_{ce}+\psi_{ac}\cdot\psi_{ev}+\psi_{ac}\cdot\psi_{cv}+\psi_{ce}\cdot\psi_{cv}$	0.172895	0.91299	-10063.4
4	$\overline{u} \sim \psi_{ac}+\psi_{ce}+\psi_{ev}+\psi_{cv}+\psi_{ac}\cdot\psi_{ce}+\psi_{ac}\cdot\psi_{cv}+\psi_{ce}\cdot\psi_{ev}+\psi_{ce}\cdot\psi_{cv}+\psi_{ev}\cdot\psi_{cv}$	0.171358	0.91242	-9862.31
5	$\overline{u} \sim \psi_{ac}+\psi_{ce}+\psi_{ev}+\psi_{cv}+\psi_{ac}\cdot\psi_{ce}+\psi_{ac}\cdot\psi_{cv}+\psi_{ce}\cdot\psi_{ev}+\psi_{ce}\cdot\psi_{cv}$	0.171341	0.91241	-9860.64
6	$\overline{u} \sim \psi_{ac}+\psi_{ce}+\psi_{ev}+\psi_{cv}+\psi_{ac}\cdot\psi_{ce}+\psi_{ac}\cdot\psi_{cv}+\psi_{ce}\cdot\psi_{cv}+\psi_{ev}\cdot\psi_{cv}$	0.171098	0.91238	-9850.52
7	$\overline{u} \sim \psi_{ac}+\psi_{ce}+\psi_{ev}+\psi_{cv}+\psi_{ac}\cdot\psi_{ce}+\psi_{ac}\cdot\psi_{cv}+\psi_{ce}\cdot\psi_{cv}$	0.171076	0.91237	-9848.33
8	$\overline{u} \sim \psi_{ac}+\psi_{ce}+\psi_{ev}+\psi_{cv}+\psi_{ac}\cdot\psi_{ce}+\psi_{ac}\cdot\psi_{ev}+\psi_{ce}\cdot\psi_{cv}+\psi_{ev}\cdot\psi_{cv}$	0.144824	0.90903	-8714.46
9	$\overline{u} \sim \psi_{ac}+\psi_{ce}+\psi_{ev}+\psi_{cv}+\psi_{ac}\cdot\psi_{ce}+\psi_{ac}\cdot\psi_{ev}+\psi_{ce}\cdot\psi_{ev}+\psi_{ce}\cdot\psi_{cv}+\psi_{ev}\cdot\psi_{cv}$	0.144852	0.90903	-8712.98
10	$\overline{u} \sim \psi_{ac}+\psi_{ce}+\psi_{ev}+\psi_{cv}+\psi_{ac}\cdot\psi_{ce}+\psi_{ac}\cdot\psi_{ev}+\psi_{ce}\cdot\psi_{ev}+\psi_{ce}\cdot\psi_{cv}$	0.144558	0.90888	-8661.53
11	$\overline{u} \sim \psi_{ac}+\psi_{ce}+\psi_{ev}+\psi_{cv}+\psi_{ac}\cdot\psi_{ce}+\psi_{ac}\cdot\psi_{ev}+\psi_{ce}\cdot\psi_{cv}$	0.1445	0.90887	-8661.53
12	$\overline{u} \sim \psi_{ac}+\psi_{ce}+\psi_{ev}+\psi_{cv}+\psi_{ac}\cdot\psi_{ce}+\psi_{ce}\cdot\psi_{ev}+\psi_{ce}\cdot\psi_{cv}+\psi_{ev}\cdot\psi_{cv}$	0.143412	0.90835	-8486.34
13	$\overline{u} \sim \psi_{ac}+\psi_{ce}+\psi_{ev}+\psi_{cv}+\psi_{ac}\cdot\psi_{ce}+\psi_{ce}\cdot\psi_{ev}+\psi_{ce}\cdot\psi_{cv}$	0.143401	0.90834	-8485.39
14	$\overline{u} \sim \psi_{ac}+\psi_{ce}+\psi_{ev}+\psi_{cv}+\psi_{ac}\cdot\psi_{ce}+\psi_{ce}\cdot\psi_{cv}+\psi_{ev}\cdot\psi_{cv}$	0.143251	0.90832	-8478.3
15	$\overline{u} \sim \psi_{ac}+\psi_{ce}+\psi_{ev}+\psi_{cv}+\psi_{ac}\cdot\psi_{ce}+\psi_{ce}\cdot\psi_{cv}$	0.143237	0.90831	-8476.94
16	$\overline{u} \sim \psi_{ac}+\psi_{ce}+\psi_{cv}+\psi_{ac}\cdot\psi_{ce}+\psi_{ac}\cdot\psi_{ev}+\psi_{ac}\cdot\psi_{cv}+\psi_{ce}\cdot\psi_{ev}+\psi_{ce}\cdot\psi_{cv}+\psi_{ev}\cdot\psi_{cv}$	0.163546	0.90297	-6756.41
17	$\overline{u} \sim \psi_{ac}+\psi_{ce}+\psi_{cv}+\psi_{ac}\cdot\psi_{ce}+\psi_{ac}\cdot\psi_{ev}+\psi_{ac}\cdot\psi_{cv}+\psi_{ce}\cdot\psi_{cv}+\psi_{ev}\cdot\psi_{cv}$	0.164687	0.9023	-6547.19
18	$\overline{u} \sim \psi_{ac}+\psi_{ce}+\psi_{cv}+\psi_{ac}\cdot\psi_{ce}+\psi_{ac}\cdot\psi_{ev}+\psi_{ac}\cdot\psi_{cv}+\psi_{ce}\cdot\psi_{ev}+\psi_{ce}\cdot\psi_{cv}$	0.163746	0.90224	-6528.69
19	$\overline{u} \sim \psi_{ac}+\psi_{ce}+\psi_{cv}+\psi_{ac}\cdot\psi_{ce}+\psi_{ac}\cdot\psi_{ev}+\psi_{ac}\cdot\psi_{cv}+\psi_{ce}\cdot\psi_{cv}$	0.164846	0.90164	-6345.65
20	$\overline{u} \sim \psi_{ac}+\psi_{ce}+\psi_{ev}+\psi_{ac}\cdot\psi_{ce}+\psi_{ac}\cdot\psi_{ev}+\psi_{ac}\cdot\psi_{cv}+\psi_{ce}\cdot\psi_{ev}+\psi_{ce}\cdot\psi_{cv}+\psi_{ev}\cdot\psi_{cv}$	0.137312	0.8996	-5719.06
21	$\overline{u} \sim \psi_{ac}+\psi_{ce}+\psi_{ev}+\psi_{ac}\cdot\psi_{ce}+\psi_{ac}\cdot\psi_{ev}+\psi_{ac}\cdot\psi_{cv}+\psi_{ce}\cdot\psi_{cv}+\psi_{ev}\cdot\psi_{cv}$	0.137383	0.89958	-5714.72
22	$\overline{u} \sim \psi_{ac}+\psi_{ce}+\psi_{cv}+\psi_{ac}\cdot\psi_{ce}+\psi_{ac}\cdot\psi_{ev}+\psi_{ce}\cdot\psi_{ev}+\psi_{ce}\cdot\psi_{cv}+\psi_{ev}\cdot\psi_{cv}$	0.1395	0.89913	-5578.83
23	$\overline{u} \sim \psi_{ac}+\psi_{ce}+\psi_{cv}+\psi_{ac}\cdot\psi_{ce}+\psi_{ac}\cdot\psi_{ev}+\psi_{ce}\cdot\psi_{ev}+\psi_{ce}\cdot\psi_{cv}$	0.139623	0.89841	-5363.27

24	$\overline{\boldsymbol{u}} \sim \psi_{ac}+\psi_{ce}+\psi_{cv}+\psi_{ac}\cdot\psi_{ce}+\psi_{ac}\cdot\psi_{ev}+\psi_{ce}\cdot\psi_{cv}+\psi_{ev}\cdot\psi_{cv}$	0.140419	0.89841	-5365.5
25	$\overline{\boldsymbol{u}} \sim \psi_{ac}+\psi_{ce}+\psi_{ev}+\psi_{ac}\cdot\psi_{ce}+\psi_{ac}\cdot\psi_{ev}+\psi_{ce}\cdot\psi_{ev}+\psi_{ce}\cdot\psi_{cv}+\psi_{ev}\cdot\psi_{cv}$	0.147288	0.89785	-5197.62
26	$\overline{\boldsymbol{u}} \sim \psi_{ac}+\psi_{ce}+\psi_{ev}+\psi_{ac}\cdot\psi_{ce}+\psi_{ac}\cdot\psi_{ev}+\psi_{ce}\cdot\psi_{cv}+\psi_{ev}\cdot\psi_{cv}$	0.147502	0.89781	-5185.98
27	$\overline{\boldsymbol{u}} \sim \psi_{ac}+\psi_{ce}+\psi_{cv}+\psi_{ac}\cdot\psi_{ce}+\psi_{ac}\cdot\psi_{ev}+\psi_{ce}\cdot\psi_{cv}$	0.140512	0.89777	-5175.33
28	$\overline{\boldsymbol{u}} \sim \psi_{ac}+\psi_{ce}+\psi_{ev}+\psi_{ac}\cdot\psi_{ce}+\psi_{ac}\cdot\psi_{cv}+\psi_{ce}\cdot\psi_{cv}+\psi_{ev}\cdot\psi_{cv}$	0.132749	0.89739	-5060.95
29	$\overline{\boldsymbol{u}} \sim \psi_{ac}+\psi_{ce}+\psi_{ev}+\psi_{ac}\cdot\psi_{ce}+\psi_{ac}\cdot\psi_{cv}+\psi_{ce}\cdot\psi_{ev}+\psi_{ce}\cdot\psi_{cv}+\psi_{ev}\cdot\psi_{cv}$	0.132818	0.89739	-5061
30	$\overline{\boldsymbol{u}} \sim \psi_{ac}+\psi_{ce}+\psi_{cv}+\psi_{ac}\cdot\psi_{ce}+\psi_{ac}\cdot\psi_{cv}+\psi_{ce}\cdot\psi_{ev}+\psi_{ce}\cdot\psi_{cv}+\psi_{ev}\cdot\psi_{cv}$	0.154411	0.89638	-4762.76
31	$\overline{\boldsymbol{u}} \sim \psi_{ac}+\psi_{ce}+\psi_{ev}+\psi_{ac}\cdot\psi_{ce}+\psi_{ce}\cdot\psi_{cv}+\psi_{ev}\cdot\psi_{cv}$	0.144489	0.89445	-4204.76
32	$\overline{\boldsymbol{u}} \sim \psi_{ac}+\psi_{ce}+\psi_{ev}+\psi_{ac}\cdot\psi_{ce}+\psi_{ce}\cdot\psi_{ev}+\psi_{ce}\cdot\psi_{cv}+\psi_{ev}\cdot\psi_{cv}$	0.144545	0.89445	-4203.55
33	$\overline{\boldsymbol{u}} \sim \psi_{ac}+\psi_{ce}+\psi_{ev}+\psi_{ac}\cdot\psi_{ce}+\psi_{ac}\cdot\psi_{ev}+\psi_{ac}\cdot\psi_{cv}+\psi_{ce}\cdot\psi_{cv}$	0.125726	0.89355	-3946.69
34	$\overline{\boldsymbol{u}} \sim \psi_{ac}+\psi_{ce}+\psi_{ev}+\psi_{ac}\cdot\psi_{ce}+\psi_{ac}\cdot\psi_{ev}+\psi_{ac}\cdot\psi_{cv}+\psi_{ce}\cdot\psi_{ev}+\psi_{ce}\cdot\psi_{cv}$	0.125755	0.89355	-3945.29
35	$\overline{\boldsymbol{u}} \sim \psi_{ac}+\psi_{ce}+\psi_{ev}+\psi_{ac}\cdot\psi_{ce}+\psi_{ac}\cdot\psi_{cv}+\psi_{ce}\cdot\psi_{ev}+\psi_{ce}\cdot\psi_{cv}$	0.12481	0.8931	-3817.36
36	$\overline{\boldsymbol{u}} \sim \psi_{ac}+\psi_{ce}+\psi_{ev}+\psi_{ac}\cdot\psi_{ce}+\psi_{ac}\cdot\psi_{cv}+\psi_{ce}\cdot\psi_{cv}$	0.124699	0.89308	-3813.89
37	$\overline{\boldsymbol{u}} \sim \psi_{ac}+\psi_{ce}+\psi_{cv}+\psi_{ac}\cdot\psi_{ce}+\psi_{ce}\cdot\psi_{ev}+\psi_{ce}\cdot\psi_{cv}+\psi_{ev}\cdot\psi_{cv}$	0.134305	0.89275	-3719.92
38	$\overline{\boldsymbol{u}} \sim \psi_{ac}+\psi_{ce}+\psi_{ac}\cdot\psi_{ce}+\psi_{ac}\cdot\psi_{ev}+\psi_{ac}\cdot\psi_{cv}+\psi_{ce}\cdot\psi_{ev}+\psi_{ce}\cdot\psi_{cv}+\psi_{ev}\cdot\psi_{cv}$	0.132822	0.89137	-3331.19
39	$\overline{\boldsymbol{u}} \sim \psi_{ac}+\psi_{ce}+\psi_{ac}\cdot\psi_{ce}+\psi_{ac}\cdot\psi_{ev}+\psi_{ac}\cdot\psi_{cv}+\psi_{ce}\cdot\psi_{cv}+\psi_{ev}\cdot\psi_{cv}$	0.133582	0.89046	-3079.37
40	$\overline{\boldsymbol{u}} \sim \psi_{ac}+\psi_{ce}+\psi_{ac}\cdot\psi_{ce}+\psi_{ac}\cdot\psi_{ev}+\psi_{ce}\cdot\psi_{ev}+\psi_{ce}\cdot\psi_{cv}+\psi_{ev}\cdot\psi_{cv}$	0.139993	0.89015	-2991.25
41	$\overline{\boldsymbol{u}} \sim \psi_{ac}+\psi_{ce}+\psi_{ac}\cdot\psi_{ce}+\psi_{ac}\cdot\psi_{cv}+\psi_{ce}\cdot\psi_{ev}+\psi_{ce}\cdot\psi_{cv}+\psi_{ev}\cdot\psi_{cv}$	0.133966	0.88953	-2820.92
42	$\overline{\boldsymbol{u}} \sim \psi_{ac}+\psi_{ce}+\psi_{ac}\cdot\psi_{ce}+\psi_{ce}\cdot\psi_{ev}+\psi_{ce}\cdot\psi_{cv}+\psi_{ev}\cdot\psi_{cv}$	0.137418	0.88927	-2750.23
43	$\overline{\boldsymbol{u}} \sim \psi_{ac}+\psi_{ce}+\psi_{ac}\cdot\psi_{ce}+\psi_{ac}\cdot\psi_{ev}+\psi_{ce}\cdot\psi_{cv}+\psi_{ev}\cdot\psi_{cv}$	0.140965	0.88917	-2724.74
44	$\overline{\boldsymbol{u}} \sim \psi_{ac}+\psi_{ce}+\psi_{cv}+\psi_{ac}\cdot\psi_{ce}+\psi_{ac}\cdot\psi_{cv}+\psi_{ce}\cdot\psi_{cv}+\psi_{ev}\cdot\psi_{cv}$	0.152449	0.8874	-2242.54
45	$\overline{\boldsymbol{u}} \sim \psi_{ac}+\psi_{ce}+\psi_{ev}+\psi_{cv}+\psi_{ac}\cdot\psi_{ce}+\psi_{ac}\cdot\psi_{ev}+\psi_{ac}\cdot\psi_{cv}+\psi_{ev}\cdot\psi_{cv}$	0.208463	0.88465	-1509.68
46	$\overline{\boldsymbol{u}} \sim \psi_{ac}+\psi_{ce}+\psi_{ev}+\psi_{cv}+\psi_{ac}\cdot\psi_{ce}+\psi_{ac}\cdot\psi_{ev}+\psi_{ac}\cdot\psi_{cv}+\psi_{ce}\cdot\psi_{ev}+\psi_{ev}\cdot\psi_{cv}$	0.208512	0.88465	-1508.14
47	$\overline{\boldsymbol{u}} \sim \psi_{ac}+\psi_{ce}+\psi_{ev}+\psi_{cv}+\psi_{ac}\cdot\psi_{ce}+\psi_{ac}\cdot\psi_{ev}+\psi_{ac}\cdot\psi_{cv}$	0.207967	0.88449	-1468.72
48	$\overline{\boldsymbol{u}} \sim \psi_{ac}+\psi_{ce}+\psi_{ev}+\psi_{cv}+\psi_{ac}\cdot\psi_{ce}+\psi_{ac}\cdot\psi_{ev}+\psi_{ac}\cdot\psi_{cv}+\psi_{ce}\cdot\psi_{ev}$	0.208063	0.88449	-1468.32
49	$\overline{\boldsymbol{u}} \sim \psi_{ac}+\psi_{ce}+\psi_{ev}+\psi_{cv}+\psi_{ac}\cdot\psi_{ce}+\psi_{ac}\cdot\psi_{cv}+\psi_{ce}\cdot\psi_{ev}$	0.20626	0.88396	-1329.21
50	$\overline{\boldsymbol{u}} \sim \psi_{ac}+\psi_{ce}+\psi_{ev}+\psi_{cv}+\psi_{ac}\cdot\psi_{ce}+\psi_{ac}\cdot\psi_{cv}+\psi_{ce}\cdot\psi_{ev}+\psi_{ev}\cdot\psi_{cv}$	0.206277	0.88396	-1329.52

51	$\overline{\boldsymbol{u}} \sim \psi_{ac}+\psi_{ce}+\psi_{ev}+\psi_{cv}+\psi_{ac}\cdot\psi_{ce}+\psi_{ac}\cdot\psi_{cv}+\psi_{ev}\cdot\psi_{cv}$	0.206024	0.88394	-1323.69
52	$\overline{\boldsymbol{u}} \sim \psi_{ac}+\psi_{ce}+\psi_{ev}+\psi_{cv}+\psi_{ac}\cdot\psi_{ce}+\psi_{ac}\cdot\psi_{cv}$	0.206002	0.88393	-1323.08
53	$\overline{\boldsymbol{u}} \sim \psi_{ac}+\psi_{ce}+\psi_{ac}\cdot\psi_{ce}+\psi_{ac}\cdot\psi_{cv}+\psi_{ce}\cdot\psi_{cv}+\psi_{ev}\cdot\psi_{cv}$	0.137969	0.88384	-1298.46
54	$\overline{\boldsymbol{u}} \sim \psi_{ac}+\psi_{ce}+\psi_{cv}+\psi_{ac}\cdot\psi_{ce}+\psi_{ce}\cdot\psi_{cv}+\psi_{ev}\cdot\psi_{cv}$	0.13379	0.88377	-1280.51
55	$\overline{\boldsymbol{u}} \sim \psi_{ac}+\psi_{ce}+\psi_{ac}\cdot\psi_{ce}+\psi_{ce}\cdot\psi_{cv}+\psi_{ev}\cdot\psi_{cv}$	0.134625	0.88355	-1224.27
56	$\overline{\boldsymbol{u}} \sim \psi_{ac}+\psi_{ce}+\psi_{ac}\cdot\psi_{ce}+\psi_{ac}\cdot\psi_{ev}+\psi_{ac}\cdot\psi_{cv}+\psi_{ce}\cdot\psi_{ev}+\psi_{ce}\cdot\psi_{cv}$	0.122379	0.88348	-1203.37
57	$\overline{\boldsymbol{u}} \sim \psi_{ac}+\psi_{ce}+\psi_{ac}\cdot\psi_{ce}+\psi_{ac}\cdot\psi_{ev}+\psi_{ac}\cdot\psi_{cv}+\psi_{ce}\cdot\psi_{cv}$	0.123212	0.8828	-1027.84
58	$\overline{\boldsymbol{u}} \sim \psi_{ac}+\psi_{ce}+\psi_{ev}+\psi_{ac}\cdot\psi_{ce}+\psi_{ac}\cdot\psi_{ev}+\psi_{ce}\cdot\psi_{cv}$	0.145793	0.87682	482.166
59	$\overline{\boldsymbol{u}} \sim \psi_{ac}+\psi_{ce}+\psi_{ev}+\psi_{ac}\cdot\psi_{ce}+\psi_{ac}\cdot\psi_{ev}+\psi_{ce}\cdot\psi_{ev}+\psi_{ce}\cdot\psi_{cv}$	0.145824	0.87681	483.775
60	$\overline{\boldsymbol{u}} \sim \psi_{ac}+\psi_{ce}+\psi_{ev}+\psi_{ac}\cdot\psi_{ce}+\psi_{ac}\cdot\psi_{ev}+\psi_{ac}\cdot\psi_{cv}+\psi_{ce}\cdot\psi_{ev}+\psi_{ev}\cdot\psi_{cv}$	0.171182	0.87652	558.071
61	$\overline{\boldsymbol{u}} \sim \psi_{ac}+\psi_{ce}+\psi_{ev}+\psi_{ac}\cdot\psi_{ce}+\psi_{ac}\cdot\psi_{ev}+\psi_{ac}\cdot\psi_{cv}+\psi_{ev}\cdot\psi_{cv}$	0.171235	0.87651	559.164
62	$\overline{\boldsymbol{u}} \sim \psi_{ac}+\psi_{ce}+\psi_{ev}+\psi_{ac}\cdot\psi_{ce}+\psi_{ce}\cdot\psi_{ev}+\psi_{ce}\cdot\psi_{cv}$	0.144719	0.87637	591.065
63	$\overline{\boldsymbol{u}} \sim \psi_{ac}+\psi_{ce}+\psi_{ev}+\psi_{ac}\cdot\psi_{ce}+\psi_{ce}\cdot\psi_{cv}$	0.144596	0.87636	593.264
64	$\overline{\boldsymbol{u}} \sim \psi_{ac}+\psi_{ce}+\psi_{cv}+\psi_{ac}\cdot\psi_{ce}+\psi_{ac}\cdot\psi_{cv}+\psi_{ce}\cdot\psi_{ev}+\psi_{ce}\cdot\psi_{cv}$	0.141147	0.8763	611.062
65	$\overline{\boldsymbol{u}} \sim \psi_{ac}+\psi_{ce}+\psi_{ev}+\psi_{ac}\cdot\psi_{ce}+\psi_{ac}\cdot\psi_{cv}+\psi_{ce}\cdot\psi_{ev}+\psi_{ev}\cdot\psi_{cv}$	0.166104	0.87478	980.897
66	$\overline{\boldsymbol{u}} \sim \psi_{ac}+\psi_{ce}+\psi_{ev}+\psi_{ac}\cdot\psi_{ce}+\psi_{ac}\cdot\psi_{cv}+\psi_{ev}\cdot\psi_{cv}$	0.166022	0.87477	980.781
67	$\overline{\boldsymbol{u}} \sim \psi_{ac}+\psi_{ce}+\psi_{cv}+\psi_{ac}\cdot\psi_{ce}+\psi_{ac}\cdot\psi_{ev}+\psi_{ac}\cdot\psi_{cv}+\psi_{ce}\cdot\psi_{ev}+\psi_{ev}\cdot\psi_{cv}$	0.193288	0.87459	1027.25
68	$\overline{\boldsymbol{u}} \sim \psi_{ac}+\psi_{ce}+\psi_{cv}+\psi_{ac}\cdot\psi_{ce}+\psi_{ac}\cdot\psi_{ev}+\psi_{ac}\cdot\psi_{cv}+\psi_{ce}\cdot\psi_{ev}$	0.193514	0.87395	1181.63
69	$\overline{\boldsymbol{u}} \sim \psi_{ac}+\psi_{ce}+\psi_{cv}+\psi_{ac}\cdot\psi_{ce}+\psi_{ac}\cdot\psi_{ev}+\psi_{ac}\cdot\psi_{cv}+\psi_{ev}\cdot\psi_{cv}$	0.194392	0.87393	1185.74
70	$\overline{\boldsymbol{u}} \sim \psi_{ac}+\psi_{ce}+\psi_{ev}+\psi_{ac}\cdot\psi_{ce}+\psi_{ac}\cdot\psi_{ev}+\psi_{ev}\cdot\psi_{cv}$	0.163659	0.87343	1303.59
71	$\overline{\boldsymbol{u}} \sim \psi_{ac}+\psi_{ce}+\psi_{ev}+\psi_{cv}+\psi_{ac}\cdot\psi_{ce}+\psi_{ac}\cdot\psi_{ev}+\psi_{ev}\cdot\psi_{cv}$	0.163689	0.87343	1305.51
72	$\overline{\boldsymbol{u}} \sim \psi_{ac}+\psi_{ce}+\psi_{ev}+\psi_{ac}\cdot\psi_{ce}+\psi_{ac}\cdot\psi_{ev}+\psi_{ce}\cdot\psi_{ev}+\psi_{ev}\cdot\psi_{cv}$	0.163664	0.87343	1305.57
73	$\overline{\boldsymbol{u}} \sim \psi_{ac}+\psi_{ce}+\psi_{ev}+\psi_{cv}+\psi_{ac}\cdot\psi_{ce}+\psi_{ac}\cdot\psi_{ev}+\psi_{ce}\cdot\psi_{ev}+\psi_{ev}\cdot\psi_{cv}$	0.163697	0.87343	1307.49
74	$\overline{\boldsymbol{u}} \sim \psi_{ac}+\psi_{ce}+\psi_{cv}+\psi_{ac}\cdot\psi_{ce}+\psi_{ac}\cdot\psi_{ev}+\psi_{ac}\cdot\psi_{cv}$	0.194583	0.87336	1321.32
75	$\overline{\boldsymbol{u}} \sim \psi_{ac}+\psi_{ce}+\psi_{ev}+\psi_{cv}+\psi_{ac}\cdot\psi_{ce}+\psi_{ac}\cdot\psi_{ev}$	0.163366	0.8733	1335.05
76	$\overline{\boldsymbol{u}} \sim \psi_{ac}+\psi_{ce}+\psi_{ev}+\psi_{cv}+\psi_{ac}\cdot\psi_{ce}+\psi_{ac}\cdot\psi_{ev}+\psi_{ce}\cdot\psi_{ev}$	0.163402	0.8733	1336.58
77	$\overline{\boldsymbol{u}} \sim \psi_{ac}+\psi_{ce}+\psi_{cv}+\psi_{ac}\cdot\psi_{ce}+\psi_{ce}\cdot\psi_{ev}+\psi_{ce}\cdot\psi_{cv}$	0.125665	0.87313	1377.23

78	$\overline{\boldsymbol{u}} \sim \psi_{ac}+\psi_{ce}+\psi_{ev}+\psi_{cv}+\psi_{ac}\cdot\psi_{ce}+\psi_{ce}\cdot\psi_{ev}$	0.162236	0.87285	1443.57
79	$\overline{\boldsymbol{u}} \sim \psi_{ac}+\psi_{ce}+\psi_{ev}+\psi_{cv}+\psi_{ac}\cdot\psi_{ce}+\psi_{ce}\cdot\psi_{ev}+\psi_{ev}\cdot\psi_{cv}$	0.162248	0.87285	1444.08
80	$\overline{\boldsymbol{u}} \sim \psi_{ac}+\psi_{ce}+\psi_{ev}+\psi_{cv}+\psi_{ac}\cdot\psi_{ce}$	0.162102	0.87284	1445.97
81	$\overline{\boldsymbol{u}} \sim \psi_{ac}+\psi_{ce}+\psi_{ev}+\psi_{cv}+\psi_{ac}\cdot\psi_{ce}+\psi_{ev}\cdot\psi_{cv}$	0.162117	0.87284	1446.29
82	$\overline{\boldsymbol{u}} \sim \psi_{ac}+\psi_{ce}+\psi_{ev}+\psi_{ac}\cdot\psi_{ce}+\psi_{ce}\cdot\psi_{ev}+\psi_{ev}\cdot\psi_{cv}$	0.161508	0.87277	1463.64
83	$\overline{\boldsymbol{u}} \sim \psi_{ac}+\psi_{ce}+\psi_{ev}+\psi_{ac}\cdot\psi_{ce}+\psi_{ev}\cdot\psi_{cv}$	0.161397	0.87275	1465.21
84	$\overline{\boldsymbol{u}} \sim \psi_{ac}+\psi_{ce}+\psi_{ev}+\psi_{ac}\cdot\psi_{ce}+\psi_{ac}\cdot\psi_{ev}+\psi_{ac}\cdot\psi_{cv}$	0.158216	0.87246	1536.01
85	$\overline{\boldsymbol{u}} \sim \psi_{ac}+\psi_{ce}+\psi_{ev}+\psi_{ac}\cdot\psi_{ce}+\psi_{ac}\cdot\psi_{ev}+\psi_{ac}\cdot\psi_{cv}+\psi_{ce}\cdot\psi_{ev}$	0.158247	0.87246	1537.62
86	$\overline{\boldsymbol{u}} \sim \psi_{ac}+\psi_{ce}+\psi_{ev}+\psi_{ac}\cdot\psi_{ce}+\psi_{ac}\cdot\psi_{ev}$	0.157777	0.87245	1538.46
87	$\overline{\boldsymbol{u}} \sim \psi_{ac}+\psi_{ce}+\psi_{ev}+\psi_{ac}\cdot\psi_{ce}+\psi_{ac}\cdot\psi_{ev}+\psi_{ce}\cdot\psi_{ev}$	0.157808	0.87244	1540.07
88	$\overline{\boldsymbol{u}} \sim \psi_{ac}+\psi_{ce}+\psi_{ev}+\psi_{ac}\cdot\psi_{ce}+\psi_{ac}\cdot\psi_{cv}+\psi_{ce}\cdot\psi_{ev}$	0.157146	0.87202	1641.9
89	$\overline{\boldsymbol{u}} \sim \psi_{ac}+\psi_{ce}+\psi_{ev}+\psi_{ac}\cdot\psi_{ce}+\psi_{ac}\cdot\psi_{cv}$	0.157023	0.872	1644.01
90	$\overline{\boldsymbol{u}} \sim \psi_{ac}+\psi_{ce}+\psi_{ev}+\psi_{ac}\cdot\psi_{ce}+\psi_{ce}\cdot\psi_{ev}$	0.156715	0.872	1644.21
91	$\overline{\boldsymbol{u}} \sim \psi_{ac}+\psi_{ce}+\psi_{ev}+\psi_{ac}\cdot\psi_{ce}$	0.156592	0.87199	1646.3
92	$\overline{\boldsymbol{u}} \sim \psi_{ac}+\psi_{ce}+\psi_{ac}\cdot\psi_{ce}+\psi_{ac}\cdot\psi_{ev}+\psi_{ac}\cdot\psi_{cv}+\psi_{ce}\cdot\psi_{ev}+\psi_{ev}\cdot\psi_{cv}$	0.164038	0.86797	2586.17
93	$\overline{\boldsymbol{u}} \sim \psi_{ac}+\psi_{ce}+\psi_{cv}+\psi_{ac}\cdot\psi_{ce}+\psi_{ac}\cdot\psi_{cv}+\psi_{ce}\cdot\psi_{ev}+\psi_{ev}\cdot\psi_{cv}$	0.179457	0.8675	2694.92
94	$\overline{\boldsymbol{u}} \sim \psi_{ac}+\psi_{ce}+\psi_{ac}\cdot\psi_{ce}+\psi_{ac}\cdot\psi_{ev}+\psi_{ac}\cdot\psi_{cv}+\psi_{ev}\cdot\psi_{cv}$	0.164743	0.86714	2776.01
95	$\overline{\boldsymbol{u}} \sim \psi_{ac}+\psi_{ce}+\psi_{ac}\cdot\psi_{ce}+\psi_{ac}\cdot\psi_{ev}+\psi_{ce}\cdot\psi_{ev}+\psi_{ce}\cdot\psi_{cv}$	0.140723	0.86687	2838.01
96	$\overline{\boldsymbol{u}} \sim \psi_{ce}+\psi_{ev}+\psi_{cv}+\psi_{ac}\cdot\psi_{ce}+\psi_{ce}\cdot\psi_{ev}+\psi_{ce}\cdot\psi_{cv}$	0.933017	0.86665	2888.03
97	$\overline{\boldsymbol{u}} \sim \psi_{ce}+\psi_{ev}+\psi_{cv}+\psi_{ac}\cdot\psi_{ce}+\psi_{ce}\cdot\psi_{ev}+\psi_{ce}\cdot\psi_{cv}+\psi_{ev}\cdot\psi_{cv}$	0.933017	0.86665	2887.93
98	$\overline{\boldsymbol{u}} \sim \psi_{ce}+\psi_{ev}+\psi_{cv}+\psi_{ac}\cdot\psi_{ce}+\psi_{ce}\cdot\psi_{cv}$	0.933033	0.86656	2908.32
99	$\overline{\boldsymbol{u}} \sim \psi_{ce}+\psi_{ev}+\psi_{cv}+\psi_{ac}\cdot\psi_{ce}+\psi_{ce}\cdot\psi_{cv}+\psi_{ev}\cdot\psi_{cv}$	0.933032	0.86656	2907.82
100	$\overline{\boldsymbol{u}} \sim \psi_{ac}+\psi_{ce}+\psi_{ac}\cdot\psi_{ce}+\psi_{ac}\cdot\psi_{ev}+\psi_{ce}\cdot\psi_{cv}$	0.141628	0.86621	2986.85
101	$\overline{\boldsymbol{u}} \sim \psi_{ce}+\psi_{ev}+\psi_{ac}\cdot\psi_{ce}+\psi_{ce}\cdot\psi_{ev}+\psi_{ce}\cdot\psi_{cv}+\psi_{ev}\cdot\psi_{cv}$	0.935042	0.86621	2988.81
102	$\overline{\boldsymbol{u}} \sim \psi_{ce}+\psi_{ev}+\psi_{ac}\cdot\psi_{ce}+\psi_{ce}\cdot\psi_{ev}+\psi_{ce}\cdot\psi_{cv}$	0.935011	0.86617	2996.53
103	$\overline{\boldsymbol{u}} \sim \psi_{ce}+\psi_{ev}+\psi_{ac}\cdot\psi_{ce}+\psi_{ce}\cdot\psi_{cv}+\psi_{ev}\cdot\psi_{cv}$	0.935057	0.86612	3007.98
104	$\overline{\boldsymbol{u}} \sim \psi_{ce}+\psi_{ev}+\psi_{ac}\cdot\psi_{ce}+\psi_{ce}\cdot\psi_{cv}$	0.935026	0.86607	3016.51

105	$\overline{\boldsymbol{u}} \sim \psi_{ac}+\psi_{ce}+\psi_{ac}\cdot\psi_{ce}+\psi_{ac}\cdot\psi_{cv}+\psi_{ce}\cdot\psi_{ev}+\psi_{ev}\cdot\psi_{cv}$	0.164491	0.86479	3307.69
106	$\overline{\boldsymbol{u}} \sim \psi_{ac}+\psi_{ce}+\psi_{cv}+\psi_{ac}\cdot\psi_{ce}+\psi_{ac}\cdot\psi_{ev}+\psi_{ce}\cdot\psi_{ev}+\psi_{ev}\cdot\psi_{cv}$	0.156758	0.86392	3504.23
107	$\overline{\boldsymbol{u}} \sim \psi_{ac}+\psi_{ce}+\psi_{ac}\cdot\psi_{ce}+\psi_{ac}\cdot\psi_{ev}+\psi_{ce}\cdot\psi_{ev}+\psi_{ev}\cdot\psi_{cv}$	0.157653	0.86377	3535.1
108	$\overline{\boldsymbol{u}} \sim \psi_{ac}+\psi_{ce}+\psi_{cv}+\psi_{ac}\cdot\psi_{ce}+\psi_{ac}\cdot\psi_{ev}+\psi_{ce}\cdot\psi_{ev}$	0.156853	0.8633	3639.91
109	$\overline{\boldsymbol{u}} \sim \psi_{ac}+\psi_{ce}+\psi_{cv}+\psi_{ac}\cdot\psi_{ce}+\psi_{ac}\cdot\psi_{ev}+\psi_{ev}\cdot\psi_{cv}$	0.157667	0.8632	3661.86
110	$\overline{\boldsymbol{u}} \sim \psi_{ac}+\psi_{ce}+\psi_{ac}\cdot\psi_{ce}+\psi_{ac}\cdot\psi_{ev}+\psi_{ev}\cdot\psi_{cv}$	0.158445	0.8631	3684.94
111	$\overline{\boldsymbol{u}} \sim \psi_{ac}+\psi_{ce}+\psi_{cv}+\psi_{ac}\cdot\psi_{ce}+\psi_{ac}\cdot\psi_{ev}$	0.157737	0.86266	3780.29
112	$\overline{\boldsymbol{u}} \sim \psi_{ac}+\psi_{ce}+\psi_{ac}\cdot\psi_{ce}+\psi_{ac}\cdot\psi_{ev}+\psi_{ac}\cdot\psi_{cv}+\psi_{ce}\cdot\psi_{ev}$	0.152354	0.86251	3815.73
113	$\overline{\boldsymbol{u}} \sim \psi_{ac}+\psi_{ce}+\psi_{ac}\cdot\psi_{ce}+\psi_{ac}\cdot\psi_{ev}+\psi_{ce}\cdot\psi_{ev}$	0.15198	0.8625	3817.26
114	$\overline{\boldsymbol{u}} \sim \psi_{ac}+\psi_{ce}+\psi_{ac}\cdot\psi_{ce}+\psi_{ac}\cdot\psi_{ev}+\psi_{ac}\cdot\psi_{cv}$	0.153212	0.86186	3956.8
115	$\overline{\boldsymbol{u}} \sim \psi_{ac}+\psi_{ce}+\psi_{ac}\cdot\psi_{ce}+\psi_{ac}\cdot\psi_{ev}$	0.152832	0.86185	3958.43
116	$\overline{\boldsymbol{u}} \sim \psi_{ac}+\psi_{ce}+\psi_{ac}\cdot\psi_{ce}+\psi_{ac}\cdot\psi_{cv}+\psi_{ce}\cdot\psi_{ev}+\psi_{ce}\cdot\psi_{cv}$	0.112234	0.85874	4635.21
117	$\overline{\boldsymbol{u}} \sim \psi_{ac}+\psi_{ce}+\psi_{cv}+\psi_{ac}\cdot\psi_{ce}+\psi_{ac}\cdot\psi_{cv}+\psi_{ev}\cdot\psi_{cv}$	0.175244	0.8582	4752.47
118	$\overline{\boldsymbol{u}} \sim \psi_{ac}+\psi_{ce}+\psi_{ac}\cdot\psi_{ce}+\psi_{ac}\cdot\psi_{cv}+\psi_{ev}\cdot\psi_{cv}$	0.168051	0.8575	4899.62
119	$\overline{\boldsymbol{u}} \sim \psi_{ac}+\psi_{ce}+\psi_{cv}+\psi_{ac}\cdot\psi_{ce}+\psi_{ce}\cdot\psi_{ev}+\psi_{ev}\cdot\psi_{cv}$	0.149829	0.85718	4969.29
120	$\overline{\boldsymbol{u}} \sim \psi_{ce}+\psi_{ev}+\psi_{ac}\cdot\psi_{ce}+\psi_{ac}\cdot\psi_{cv}+\psi_{ce}\cdot\psi_{ev}$	1.04907	0.85589	5240.19
121	$\overline{\boldsymbol{u}} \sim \psi_{ce}+\psi_{ev}+\psi_{ac}\cdot\psi_{ce}+\psi_{ac}\cdot\psi_{cv}$	1.04909	0.85582	5255.12
122	$\overline{\boldsymbol{u}} \sim \psi_{ac}+\psi_{ce}+\psi_{cv}+\psi_{ac}\cdot\psi_{ce}+\psi_{ev}\cdot\psi_{cv}$	0.148348	0.84789	6880.65
123	$\overline{\boldsymbol{u}} \sim \psi_{ac}+\psi_{ce}+\psi_{cv}+\psi_{ac}\cdot\psi_{ce}+\psi_{ac}\cdot\psi_{cv}+\psi_{ce}\cdot\psi_{ev}$	0.161799	0.84728	7001.67
124	$\overline{\boldsymbol{u}} \sim \psi_{ac}+\psi_{ce}+\psi_{ac}\cdot\psi_{ce}+\psi_{ce}\cdot\psi_{ev}+\psi_{ev}\cdot\psi_{cv}$	0.151445	0.84668	7120.07
125	$\overline{\boldsymbol{u}} \sim \psi_{ac}+\psi_{ce}+\psi_{ac}\cdot\psi_{ce}+\psi_{ce}\cdot\psi_{ev}+\psi_{ce}\cdot\psi_{cv}$	0.126338	0.84175	8081.48
126	$\overline{\boldsymbol{u}} \sim \psi_{ac}+\psi_{ce}+\psi_{cv}+\psi_{ac}\cdot\psi_{ce}+\psi_{ce}\cdot\psi_{ev}$	0.13911	0.83775	8838.12
127	$\overline{\boldsymbol{u}} \sim \psi_{ac}+\psi_{ce}+\psi_{ac}\cdot\psi_{ce}+\psi_{ce}\cdot\psi_{ev}$	0.135827	0.83708	8962.34
128	$\overline{\boldsymbol{u}} \sim \psi_{ac}+\psi_{ce}+\psi_{ac}\cdot\psi_{ce}+\psi_{ac}\cdot\psi_{cv}+\psi_{ce}\cdot\psi_{ev}$	0.136025	0.83708	8963
129	$\overline{\boldsymbol{u}} \sim \psi_{ce}+\psi_{ev}+\psi_{cv}+\psi_{ac}\cdot\psi_{ce}+\psi_{ce}\cdot\psi_{ev}+\psi_{ev}\cdot\psi_{cv}$	0.954812	0.83569	9222.4
130	$\overline{\boldsymbol{u}} \sim \psi_{ce}+\psi_{ev}+\psi_{cv}+\psi_{ac}\cdot\psi_{ce}+\psi_{ce}\cdot\psi_{ev}$	0.954812	0.83568	9221.72
131	$\overline{\boldsymbol{u}} \sim \psi_{ce}+\psi_{ev}+\psi_{cv}+\psi_{ac}\cdot\psi_{ce}+\psi_{ev}\cdot\psi_{cv}$	0.954825	0.83563	9232.28

132	$\overline{\boldsymbol{u}} \sim \psi_{ce}+\psi_{ev}+\psi_{cv}+\psi_{ac}\cdot\psi_{ce}$	0.954825	0.83562	9231.83
133	$\overline{\boldsymbol{u}} \sim \psi_{ce}+\psi_{ev}+\psi_{ac}\cdot\psi_{ce}+\psi_{ce}\cdot\psi_{ev}+\psi_{ev}\cdot\psi_{cv}$	0.944066	0.83266	9774.51
134	$\overline{\boldsymbol{u}} \sim \psi_{ce}+\psi_{ev}+\psi_{ac}\cdot\psi_{ce}+\psi_{ce}\cdot\psi_{ev}$	0.94332	0.8326	9785.22
135	$\overline{\boldsymbol{u}} \sim \psi_{ce}+\psi_{ev}+\psi_{ac}\cdot\psi_{ce}+\psi_{ev}\cdot\psi_{cv}$	0.944056	0.8326	9784.9
136	$\overline{\boldsymbol{u}} \sim \psi_{ce}+\psi_{ev}+\psi_{ac}\cdot\psi_{ce}$	0.943329	0.83254	9794.92
137	$\overline{\boldsymbol{u}} \sim \psi_{ce}+\psi_{ac}\cdot\psi_{ce}+\psi_{ac}\cdot\psi_{ev}+\psi_{ac}\cdot\psi_{cv}$	1.18663	0.82332	11422.6
138	$\overline{\boldsymbol{u}} \sim \psi_{ce}+\psi_{ev}+\psi_{cv}+\psi_{ac}\cdot\psi_{ce}+\psi_{ac}\cdot\psi_{ev}+\psi_{ac}\cdot\psi_{cv}+\psi_{ce}\cdot\psi_{ev}+\psi_{ce}\cdot\psi_{cv}+\psi_{ev}\cdot\psi_{cv}$	0.176657	0.80361	14635.8
139	$\overline{\boldsymbol{u}} \sim \psi_{ce}+\psi_{ev}+\psi_{cv}+\psi_{ac}\cdot\psi_{ce}+\psi_{ac}\cdot\psi_{ev}+\psi_{ac}\cdot\psi_{cv}+\psi_{ce}\cdot\psi_{cv}+\psi_{ev}\cdot\psi_{cv}$	0.177243	0.80211	14865.2
140	$\overline{\boldsymbol{u}} \sim \psi_{ce}+\psi_{cv}+\psi_{ac}\cdot\psi_{ce}+\psi_{ac}\cdot\psi_{ev}+\psi_{ce}\cdot\psi_{cv}$	1.12849	0.79923	15301
141	$\overline{\boldsymbol{u}} \sim \psi_{ce}+\psi_{ac}\cdot\psi_{ce}+\psi_{ac}\cdot\psi_{ev}+\psi_{ce}\cdot\psi_{cv}$	1.13028	0.79812	15467.4
142	$\overline{\boldsymbol{u}} \sim \psi_{ac}+\psi_{ce}+\psi_{ac}\cdot\psi_{ce}+\psi_{ev}\cdot\psi_{cv}$	0.164354	0.79398	16082.2
143	$\overline{\boldsymbol{u}} \sim \psi_{ce}+\psi_{ev}+\psi_{cv}+\psi_{ac}\cdot\psi_{ce}+\psi_{ac}\cdot\psi_{ev}+\psi_{ac}\cdot\psi_{cv}+\psi_{ce}\cdot\psi_{ev}+\psi_{ce}\cdot\psi_{cv}$	0.172924	0.79338	16175.3
144	$\overline{\boldsymbol{u}} \sim \psi_{ce}+\psi_{ev}+\psi_{cv}+\psi_{ac}\cdot\psi_{ce}+\psi_{ac}\cdot\psi_{ev}+\psi_{ac}\cdot\psi_{cv}+\psi_{ce}\cdot\psi_{cv}$	0.173237	0.79233	16327.3
145	$\overline{\boldsymbol{u}} \sim \psi_{ce}+\psi_{ev}+\psi_{cv}+\psi_{ac}\cdot\psi_{ce}+\psi_{ac}\cdot\psi_{ev}+\psi_{ac}\cdot\psi_{cv}+\psi_{ce}\cdot\psi_{ev}+\psi_{ev}\cdot\psi_{cv}$	0.17933	0.79033	16619
146	$\overline{\boldsymbol{u}} \sim \psi_{ce}+\psi_{ev}+\psi_{cv}+\psi_{ac}\cdot\psi_{ce}+\psi_{ac}\cdot\psi_{ev}+\psi_{ac}\cdot\psi_{cv}+\psi_{ev}\cdot\psi_{cv}$	0.179677	0.78897	16814.6
147	$\overline{\boldsymbol{u}} \sim \psi_{ce}+\psi_{ev}+\psi_{cv}+\psi_{ac}\cdot\psi_{ce}+\psi_{ac}\cdot\psi_{ev}+\psi_{ac}\cdot\psi_{cv}+\psi_{ce}\cdot\psi_{ev}$	0.175068	0.78127	17901.7
148	$\overline{\boldsymbol{u}} \sim \psi_{ce}+\psi_{ev}+\psi_{cv}+\psi_{ac}\cdot\psi_{ce}+\psi_{ac}\cdot\psi_{ev}+\psi_{ac}\cdot\psi_{cv}$	0.175243	0.78031	18033.7
149	$\overline{\boldsymbol{u}} \sim \psi_{ce}+\psi_{ev}+\psi_{ac}\cdot\psi_{ce}+\psi_{ac}\cdot\psi_{ev}+\psi_{ac}\cdot\psi_{cv}+\psi_{ce}\cdot\psi_{ev}+\psi_{ce}\cdot\psi_{cv}+\psi_{ev}\cdot\psi_{cv}$	0.160397	0.758	20969.9
150	$\overline{\boldsymbol{u}} \sim \psi_{ce}+\psi_{cv}+\psi_{ac}\cdot\psi_{ce}+\psi_{ce}\cdot\psi_{ev}+\psi_{ce}\cdot\psi_{cv}+\psi_{ev}\cdot\psi_{cv}$	1.03326	0.75498	21344.1
151	$\overline{\boldsymbol{u}} \sim \psi_{ce}+\psi_{ev}+\psi_{ac}\cdot\psi_{ce}+\psi_{ac}\cdot\psi_{ev}+\psi_{ac}\cdot\psi_{cv}+\psi_{ce}\cdot\psi_{cv}+\psi_{ev}\cdot\psi_{cv}$	0.162357	0.75468	21381.9
152	$\overline{\boldsymbol{u}} \sim \psi_{ce}+\psi_{ev}+\psi_{ac}\cdot\psi_{ce}+\psi_{ac}\cdot\psi_{ev}+\psi_{ac}\cdot\psi_{cv}+\psi_{ce}\cdot\psi_{ev}+\psi_{ev}\cdot\psi_{cv}$	0.162582	0.75414	21449.6
153	$\overline{\boldsymbol{u}} \sim \psi_{ce}+\psi_{ac}\cdot\psi_{ce}+\psi_{ce}\cdot\psi_{ev}+\psi_{ce}\cdot\psi_{cv}+\psi_{ev}\cdot\psi_{cv}$	1.03187	0.75216	21690.6
154	$\overline{\boldsymbol{u}} \sim \psi_{ce}+\psi_{ev}+\psi_{ac}\cdot\psi_{ce}+\psi_{ac}\cdot\psi_{ev}+\psi_{ac}\cdot\psi_{cv}+\psi_{ev}\cdot\psi_{cv}$	0.16405	0.7511	21821.1
155	$\overline{\boldsymbol{u}} \sim \psi_{ac}+\psi_{ce}+\psi_{cv}+\psi_{ac}\cdot\psi_{ce}+\psi_{ac}\cdot\psi_{cv}+\psi_{ce}\cdot\psi_{cv}$	0.117187	0.74238	22866
156	$\overline{\boldsymbol{u}} \sim \psi_{ac}+\psi_{ce}+\psi_{cv}+\psi_{ac}\cdot\psi_{ce}+\psi_{ce}\cdot\psi_{cv}$	0.101953	0.74007	23134.9
157	$\overline{\boldsymbol{u}} \sim \psi_{ac}+\psi_{ce}+\psi_{ac}\cdot\psi_{ce}+\psi_{ac}\cdot\psi_{cv}+\psi_{ce}\cdot\psi_{cv}$	0.0873924	0.72677	24649.6
158	$\overline{\boldsymbol{u}} \sim \psi_{ce}+\psi_{ev}+\psi_{cv}+\psi_{ac}\cdot\psi_{ce}+\psi_{ac}\cdot\psi_{ev}+\psi_{ce}\cdot\psi_{ev}+\psi_{ce}\cdot\psi_{cv}+\psi_{ev}\cdot\psi_{cv}$	0.163434	0.71847	25560

159	$\overline{\boldsymbol{u}} \sim \psi_{ce}+\psi_{ev}+\psi_{ac}\cdot\psi_{ce}+\psi_{ac}\cdot\psi_{ev}+\psi_{ce}\cdot\psi_{ev}+\psi_{ce}\cdot\psi_{cv}+\psi_{ev}\cdot\psi_{cv}$	0.163547	0.71824	25583.5
160	$\overline{\boldsymbol{u}} \sim \psi_{ce}+\psi_{ev}+\psi_{cv}+\psi_{ac}\cdot\psi_{ce}+\psi_{ac}\cdot\psi_{ev}+\psi_{ce}\cdot\psi_{cv}+\psi_{ev}\cdot\psi_{cv}$	0.167048	0.71451	25983.1
161	$\overline{\boldsymbol{u}} \sim \psi_{ce}+\psi_{ev}+\psi_{ac}\cdot\psi_{ce}+\psi_{ac}\cdot\psi_{ev}+\psi_{ce}\cdot\psi_{cv}+\psi_{ev}\cdot\psi_{cv}$	0.167083	0.71435	25999.2
162	$\overline{\boldsymbol{u}} \sim \psi_{ac}+\psi_{ce}+\psi_{cv}+\psi_{ac}\cdot\psi_{ce}+\psi_{ac}\cdot\psi_{cv}$	0.146584	0.71254	26189.7
163	$\overline{\boldsymbol{u}} \sim \psi_{ac}+\psi_{ce}+\psi_{ac}\cdot\psi_{ce}+\psi_{ce}\cdot\psi_{cv}$	0.101968	0.70866	26595.5
164	$\overline{\boldsymbol{u}} \sim \psi_{ac}+\psi_{ce}+\psi_{cv}+\psi_{ac}\cdot\psi_{ce}$	0.118268	0.70387	27090
165	$\overline{\boldsymbol{u}} \sim \psi_{ce}+\psi_{cv}+\psi_{ac}\cdot\psi_{ce}+\psi_{ac}\cdot\psi_{ev}+\psi_{ac}\cdot\psi_{cv}+\psi_{ce}\cdot\psi_{ev}+\psi_{ce}\cdot\psi_{cv}+\psi_{ev}\cdot\psi_{cv}$	0.123261	0.70361	27120.7
166	$\overline{\boldsymbol{u}} \sim \psi_{ac}+\psi_{ce}+\psi_{ac}\cdot\psi_{ce}$	0.114822	0.70336	27140.9
167	$\overline{\boldsymbol{u}} \sim \psi_{ac}+\psi_{ce}+\psi_{ac}\cdot\psi_{ce}+\psi_{ac}\cdot\psi_{cv}$	0.114886	0.70335	27142.8
168	$\overline{\boldsymbol{u}} \sim \psi_{ce}+\psi_{cv}+\psi_{ac}\cdot\psi_{ce}+\psi_{ac}\cdot\psi_{ev}+\psi_{ac}\cdot\psi_{cv}+\psi_{ce}\cdot\psi_{ev}+\psi_{ev}\cdot\psi_{cv}$	0.125542	0.69751	27737.5
169	$\overline{\boldsymbol{u}} \sim \psi_{ce}+\psi_{ev}+\psi_{cv}+\psi_{ac}\cdot\psi_{ce}+\psi_{ac}\cdot\psi_{ev}+\psi_{ce}\cdot\psi_{ev}+\psi_{ce}\cdot\psi_{cv}$	0.165525	0.69578	27911
170	$\overline{\boldsymbol{u}} \sim \psi_{ce}+\psi_{ev}+\psi_{cv}+\psi_{ac}\cdot\psi_{ce}+\psi_{ac}\cdot\psi_{ev}+\psi_{ce}\cdot\psi_{ev}+\psi_{ev}\cdot\psi_{cv}$	0.169938	0.6949	27998.8
171	$\overline{\boldsymbol{u}} \sim \psi_{ac}+\psi_{ev}+\psi_{cv}+\psi_{ac}\cdot\psi_{ce}+\psi_{ac}\cdot\psi_{ev}+\psi_{ac}\cdot\psi_{cv}+\psi_{ce}\cdot\psi_{ev}+\psi_{ce}\cdot\psi_{cv}+\psi_{ev}\cdot\psi_{cv}$	0.221975	0.6936	28129.3
172	$\overline{\boldsymbol{u}} \sim \psi_{ac}+\psi_{ev}+\psi_{cv}+\psi_{ac}\cdot\psi_{ce}+\psi_{ac}\cdot\psi_{ev}+\psi_{ac}\cdot\psi_{cv}+\psi_{ce}\cdot\psi_{ev}+\psi_{ce}\cdot\psi_{cv}$	0.222473	0.69351	28137
173	$\overline{\boldsymbol{u}} \sim \psi_{ac}+\psi_{ev}+\psi_{cv}+\psi_{ac}\cdot\psi_{ce}+\psi_{ac}\cdot\psi_{cv}+\psi_{ce}\cdot\psi_{ev}+\psi_{ce}\cdot\psi_{cv}$	0.223419	0.6933	28157.5
174	$\overline{\boldsymbol{u}} \sim \psi_{ac}+\psi_{ev}+\psi_{cv}+\psi_{ac}\cdot\psi_{ce}+\psi_{ac}\cdot\psi_{cv}+\psi_{ce}\cdot\psi_{ev}+\psi_{ce}\cdot\psi_{cv}+\psi_{ev}\cdot\psi_{cv}$	0.22334	0.6933	28158.2
175	$\overline{\boldsymbol{u}} \sim \psi_{ce}+\psi_{ev}+\psi_{cv}+\psi_{ac}\cdot\psi_{ce}+\psi_{ac}\cdot\psi_{ev}+\psi_{ce}\cdot\psi_{cv}$	0.168141	0.69274	28211.9
176	$\overline{\boldsymbol{u}} \sim \psi_{ce}+\psi_{ev}+\psi_{cv}+\psi_{ac}\cdot\psi_{ce}+\psi_{ac}\cdot\psi_{ev}+\psi_{ev}\cdot\psi_{cv}$	0.173055	0.69106	28377
177	$\overline{\boldsymbol{u}} \sim \psi_{ce}+\psi_{ev}+\psi_{cv}+\psi_{ac}\cdot\psi_{ce}+\psi_{ac}\cdot\psi_{cv}+\psi_{ce}\cdot\psi_{cv}$	0.126975	0.68879	28599.1
178	$\overline{\boldsymbol{u}} \sim \psi_{ce}+\psi_{ev}+\psi_{cv}+\psi_{ac}\cdot\psi_{ce}+\psi_{ac}\cdot\psi_{cv}+\psi_{ce}\cdot\psi_{ev}+\psi_{ce}\cdot\psi_{cv}$	0.126965	0.68879	28600.2
179	$\overline{\boldsymbol{u}} \sim \psi_{ce}+\psi_{ev}+\psi_{cv}+\psi_{ac}\cdot\psi_{ce}+\psi_{ac}\cdot\psi_{cv}+\psi_{ce}\cdot\psi_{cv}+\psi_{ev}\cdot\psi_{cv}$	0.126982	0.68879	28600.4
180	$\overline{\boldsymbol{u}} \sim \psi_{ce}+\psi_{ev}+\psi_{cv}+\psi_{ac}\cdot\psi_{ce}+\psi_{ac}\cdot\psi_{cv}+\psi_{ce}\cdot\psi_{ev}+\psi_{ce}\cdot\psi_{cv}+\psi_{ev}\cdot\psi_{cv}$	0.126971	0.68879	28601.4
181	$\overline{\boldsymbol{u}} \sim \psi_{ce}+\psi_{ev}+\psi_{cv}+\psi_{ac}\cdot\psi_{ce}+\psi_{ac}\cdot\psi_{cv}$	0.127839	0.68576	28892.1
182	$\overline{\boldsymbol{u}} \sim \psi_{ce}+\psi_{ev}+\psi_{cv}+\psi_{ac}\cdot\psi_{ce}+\psi_{ac}\cdot\psi_{cv}+\psi_{ce}\cdot\psi_{ev}$	0.127829	0.68576	28893.1
183	$\overline{\boldsymbol{u}} \sim \psi_{ce}+\psi_{ev}+\psi_{cv}+\psi_{ac}\cdot\psi_{ce}+\psi_{ac}\cdot\psi_{cv}+\psi_{ce}\cdot\psi_{ev}+\psi_{ev}\cdot\psi_{cv}$	0.127835	0.68576	28894.4
184	$\overline{\boldsymbol{u}} \sim \psi_{ce}+\psi_{ev}+\psi_{cv}+\psi_{ac}\cdot\psi_{ce}+\psi_{ac}\cdot\psi_{cv}+\psi_{ev}\cdot\psi_{cv}$	0.127846	0.68575	28893.5
185	$\overline{\boldsymbol{u}} \sim \psi_{ce}+\psi_{ev}+\psi_{ac}\cdot\psi_{ce}+\psi_{ac}\cdot\psi_{ev}+\psi_{ce}\cdot\psi_{ev}+\psi_{ev}\cdot\psi_{cv}$	0.17638	0.68003	29440.7

186	$\overline{\boldsymbol{u}} \sim \psi_{ce}+\psi_{ev}+\psi_{ac}\cdot\psi_{ce}+\psi_{ac}\cdot\psi_{ev}+\psi_{ac}\cdot\psi_{cv}+\psi_{ce}\cdot\psi_{ev}+\psi_{ce}\cdot\psi_{cv}$	0.161456	0.67698	29730.4
187	$\overline{\boldsymbol{u}} \sim \psi_{ce}+\psi_{ev}+\psi_{ac}\cdot\psi_{ce}+\psi_{ac}\cdot\psi_{ev}+\psi_{ev}\cdot\psi_{cv}$	0.178647	0.67689	29736.1
188	$\overline{\boldsymbol{u}} \sim \psi_{ce}+\psi_{ev}+\psi_{ac}\cdot\psi_{ce}+\psi_{ac}\cdot\psi_{ev}+\psi_{ac}\cdot\psi_{cv}+\psi_{ce}\cdot\psi_{ev}$	0.162433	0.67674	29751.1
189	$\overline{\boldsymbol{u}} \sim \psi_{ev}+\psi_{cv}+\psi_{ac}\cdot\psi_{ce}+\psi_{ac}\cdot\psi_{ev}+\psi_{ac}\cdot\psi_{cv}+\psi_{ce}\cdot\psi_{ev}+\psi_{ce}\cdot\psi_{cv}+\psi_{ev}\cdot\psi_{cv}$	0.19852	0.67528	29890.4
190	$\overline{\boldsymbol{u}} \sim \psi_{ac}+\psi_{cv}+\psi_{ac}\cdot\psi_{ce}+\psi_{ac}\cdot\psi_{ev}+\psi_{ac}\cdot\psi_{cv}+\psi_{ce}\cdot\psi_{ev}+\psi_{ce}\cdot\psi_{cv}+\psi_{ev}\cdot\psi_{cv}$	0.225544	0.67507	29909.9
191	$\overline{\boldsymbol{u}} \sim \psi_{ac}+\psi_{cv}+\psi_{ac}\cdot\psi_{ce}+\psi_{ac}\cdot\psi_{ev}+\psi_{ac}\cdot\psi_{cv}+\psi_{ce}\cdot\psi_{ev}+\psi_{ce}\cdot\psi_{cv}$	0.225675	0.67504	29911.6
192	$\overline{\boldsymbol{u}} \sim \psi_{ce}+\psi_{ev}+\psi_{ac}\cdot\psi_{ce}+\psi_{ac}\cdot\psi_{ev}+\psi_{ce}\cdot\psi_{ev}+\psi_{ce}\cdot\psi_{cv}$	0.166048	0.6749	29923.7
193	$\overline{\boldsymbol{u}} \sim \psi_{ce}+\psi_{cv}+\psi_{ac}\cdot\psi_{ce}+\psi_{ac}\cdot\psi_{cv}+\psi_{ce}\cdot\psi_{ev}+\psi_{ce}\cdot\psi_{cv}+\psi_{ev}\cdot\psi_{cv}$	0.12422	0.67485	29929.2
194	$\overline{\boldsymbol{u}} \sim \psi_{ev}+\psi_{cv}+\psi_{ac}\cdot\psi_{ce}+\psi_{ac}\cdot\psi_{ev}+\psi_{ac}\cdot\psi_{cv}+\psi_{ce}\cdot\psi_{ev}+\psi_{ce}\cdot\psi_{cv}$	0.196037	0.67418	29992
195	$\overline{\boldsymbol{u}} \sim \psi_{ce}+\psi_{ev}+\psi_{ac}\cdot\psi_{ce}+\psi_{ac}\cdot\psi_{ev}+\psi_{ac}\cdot\psi_{cv}+\psi_{ce}\cdot\psi_{cv}$	0.16337	0.67417	29991.7
196	$\overline{\boldsymbol{u}} \sim \psi_{ce}+\psi_{ev}+\psi_{ac}\cdot\psi_{ce}+\psi_{ac}\cdot\psi_{ev}+\psi_{ac}\cdot\psi_{cv}$	0.164688	0.67388	30017.9
197	$\overline{\boldsymbol{u}} \sim \psi_{ce}+\psi_{ev}+\psi_{cv}+\psi_{ac}\cdot\psi_{ce}+\psi_{ac}\cdot\psi_{ev}+\psi_{ce}\cdot\psi_{ev}$	0.17076	0.67373	30032.3
198	$\overline{\boldsymbol{u}} \sim \psi_{ce}+\psi_{ev}+\psi_{ac}\cdot\psi_{ce}+\psi_{ac}\cdot\psi_{ev}+\psi_{ce}\cdot\psi_{ev}$	0.168395	0.67283	30115.3
199	$\overline{\boldsymbol{u}} \sim \psi_{ce}+\psi_{cv}+\psi_{ac}\cdot\psi_{ce}+\psi_{ac}\cdot\psi_{ev}+\psi_{ac}\cdot\psi_{cv}+\psi_{ce}\cdot\psi_{cv}+\psi_{ev}\cdot\psi_{cv}$	0.122848	0.67236	30161
200	$\overline{\boldsymbol{u}} \sim \psi_{ce}+\psi_{ev}+\psi_{ac}\cdot\psi_{ce}+\psi_{ac}\cdot\psi_{ev}+\psi_{ce}\cdot\psi_{cv}$	0.168685	0.6719	30201.2
201	$\overline{\boldsymbol{u}} \sim \psi_{ce}+\psi_{cv}+\psi_{ac}\cdot\psi_{ce}+\psi_{ac}\cdot\psi_{cv}+\psi_{ce}\cdot\psi_{ev}+\psi_{ev}\cdot\psi_{cv}$	0.125058	0.67183	30208.9
202	$\overline{\boldsymbol{u}} \sim \psi_{ce}+\psi_{ev}+\psi_{cv}+\psi_{ac}\cdot\psi_{ce}+\psi_{ac}\cdot\psi_{ev}$	0.172988	0.67076	30306.3
203	$\overline{\boldsymbol{u}} \sim \psi_{ce}+\psi_{ev}+\psi_{ac}\cdot\psi_{ce}+\psi_{ac}\cdot\psi_{ev}$	0.170701	0.66985	30389.4
204	$\overline{\boldsymbol{u}} \sim \psi_{ce}+\psi_{cv}+\psi_{ac}\cdot\psi_{ce}+\psi_{ac}\cdot\psi_{ev}+\psi_{ac}\cdot\psi_{cv}+\psi_{ev}\cdot\psi_{cv}$	0.124199	0.66809	30553.1
205	$\overline{\boldsymbol{u}} \sim \psi_{ce}+\psi_{cv}+\psi_{ac}\cdot\psi_{ce}+\psi_{ac}\cdot\psi_{cv}+\psi_{ce}\cdot\psi_{cv}+\psi_{ev}\cdot\psi_{cv}$	0.123519	0.66476	30855.1
206	$\overline{\boldsymbol{u}} \sim \psi_{ce}+\psi_{cv}+\psi_{ac}\cdot\psi_{ce}+\psi_{ac}\cdot\psi_{cv}+\psi_{ev}\cdot\psi_{cv}$	0.124335	0.6617	31130.1
207	$\overline{\boldsymbol{u}} \sim \psi_{ce}+\psi_{cv}+\psi_{ac}\cdot\psi_{ce}+\psi_{ac}\cdot\psi_{ev}+\psi_{ac}\cdot\psi_{cv}+\psi_{ce}\cdot\psi_{ev}+\psi_{ce}\cdot\psi_{cv}$	0.120287	0.65756	31501
208	$\overline{\boldsymbol{u}} \sim \psi_{ce}+\psi_{cv}+\psi_{ac}\cdot\psi_{ce}+\psi_{ac}\cdot\psi_{cv}+\psi_{ce}\cdot\psi_{ev}+\psi_{ce}\cdot\psi_{cv}$	0.120774	0.6572	31532.1
209	$\overline{\boldsymbol{u}} \sim \psi_{ce}+\psi_{cv}+\psi_{ac}\cdot\psi_{ce}+\psi_{ac}\cdot\psi_{ev}+\psi_{ac}\cdot\psi_{cv}+\psi_{ce}\cdot\psi_{ev}$	0.121293	0.65442	31777.1
210	$\overline{\boldsymbol{u}} \sim \psi_{ce}+\psi_{cv}+\psi_{ac}\cdot\psi_{ce}+\psi_{ac}\cdot\psi_{cv}+\psi_{ce}\cdot\psi_{ev}$	0.121586	0.65423	31792.7
211	$\overline{\boldsymbol{u}} \sim \psi_{ac}+\psi_{ev}+\psi_{cv}+\psi_{ac}\cdot\psi_{ce}+\psi_{ac}\cdot\psi_{ev}+\psi_{ac}\cdot\psi_{cv}+\psi_{ce}\cdot\psi_{cv}+\psi_{ev}\cdot\psi_{cv}$	0.274939	0.65264	31935.3
212	$\overline{\boldsymbol{u}} \sim \psi_{ac}+\psi_{ev}+\psi_{cv}+\psi_{ac}\cdot\psi_{ce}+\psi_{ac}\cdot\psi_{ev}+\psi_{ac}\cdot\psi_{cv}+\psi_{ce}\cdot\psi_{cv}$	0.274888	0.65261	31936.2

213	$\overline{\boldsymbol{u}} \sim \psi_{ac}+\psi_{ev}+\psi_{cv}+\psi_{ac}\cdot\psi_{ce}+\psi_{ac}\cdot\psi_{cv}+\psi_{ce}\cdot\psi_{cv}$	0.274787	0.65257	31938.7
214	$\overline{\boldsymbol{u}} \sim \psi_{ac}+\psi_{ev}+\psi_{cv}+\psi_{ac}\cdot\psi_{ce}+\psi_{ac}\cdot\psi_{cv}+\psi_{ce}\cdot\psi_{cv}+\psi_{ev}\cdot\psi_{cv}$	0.274796	0.65257	31940
215	$\overline{\boldsymbol{u}} \sim \psi_{ac}+\psi_{ev}+\psi_{cv}+\psi_{ac}\cdot\psi_{ce}+\psi_{ac}\cdot\psi_{ev}+\psi_{ac}\cdot\psi_{cv}+\psi_{ce}\cdot\psi_{ev}+\psi_{ev}\cdot\psi_{cv}$	0.263368	0.65143	32040.1
216	$\overline{\boldsymbol{u}} \sim \psi_{ac}+\psi_{ev}+\psi_{cv}+\psi_{ac}\cdot\psi_{ce}+\psi_{ac}\cdot\psi_{ev}+\psi_{ac}\cdot\psi_{cv}+\psi_{ce}\cdot\psi_{ev}$	0.264061	0.65131	32049.7
217	$\overline{\boldsymbol{u}} \sim \psi_{ev}+\psi_{cv}+\psi_{ac}\cdot\psi_{ce}+\psi_{ac}\cdot\psi_{cv}+\psi_{ce}\cdot\psi_{ev}+\psi_{ce}\cdot\psi_{cv}$	0.160583	0.6513	32050
218	$\overline{\boldsymbol{u}} \sim \psi_{ev}+\psi_{cv}+\psi_{ac}\cdot\psi_{ce}+\psi_{ac}\cdot\psi_{cv}+\psi_{ce}\cdot\psi_{ev}+\psi_{ce}\cdot\psi_{cv}+\psi_{ev}\cdot\psi_{cv}$	0.160571	0.65129	32051.9
219	$\overline{\boldsymbol{u}} \sim \psi_{ac}+\psi_{ev}+\psi_{cv}+\psi_{ac}\cdot\psi_{ce}+\psi_{ac}\cdot\psi_{cv}+\psi_{ce}\cdot\psi_{ev}$	0.265421	0.65099	32076.8
220	$\overline{\boldsymbol{u}} \sim \psi_{ac}+\psi_{ev}+\psi_{cv}+\psi_{ac}\cdot\psi_{ce}+\psi_{ac}\cdot\psi_{cv}+\psi_{ce}\cdot\psi_{ev}+\psi_{ev}\cdot\psi_{cv}$	0.265318	0.65099	32077.5
221	$\overline{\boldsymbol{u}} \sim \psi_{ce}+\psi_{ac}\cdot\psi_{ce}+\psi_{ac}\cdot\psi_{ev}+\psi_{ac}\cdot\psi_{cv}+\psi_{ce}\cdot\psi_{ev}+\psi_{ce}\cdot\psi_{cv}+\psi_{ev}\cdot\psi_{cv}$	0.101418	0.65071	32102.4
222	$\overline{\boldsymbol{u}} \sim \psi_{ce}+\psi_{ac}\cdot\psi_{ce}+\psi_{ac}\cdot\psi_{ev}+\psi_{ac}\cdot\psi_{cv}+\psi_{ce}\cdot\psi_{ev}+\psi_{ev}\cdot\psi_{cv}$	0.102549	0.6504	32128.1
223	$\overline{\boldsymbol{u}} \sim \psi_{ac}+\psi_{ce}+\psi_{ev}+\psi_{cv}+\psi_{ac}\cdot\psi_{ev}+\psi_{ac}\cdot\psi_{cv}+\psi_{ce}\cdot\psi_{ev}+\psi_{ev}\cdot\psi_{cv}$	0.510237	0.64822	32318.8
224	$\overline{\boldsymbol{u}} \sim \psi_{ac}+\psi_{ce}+\psi_{ev}+\psi_{cv}+\psi_{ac}\cdot\psi_{ev}+\psi_{ac}\cdot\psi_{cv}+\psi_{ce}\cdot\psi_{ev}+\psi_{ce}\cdot\psi_{cv}+\psi_{ev}\cdot\psi_{cv}$	0.508718	0.64821	32320.4
225	$\overline{\boldsymbol{u}} \sim \psi_{ac}+\psi_{ce}+\psi_{ev}+\psi_{cv}+\psi_{ac}\cdot\psi_{ev}+\psi_{ac}\cdot\psi_{cv}+\psi_{ce}\cdot\psi_{ev}$	0.510069	0.6482	32319.5
226	$\overline{\boldsymbol{u}} \sim \psi_{ac}+\psi_{ce}+\psi_{ev}+\psi_{cv}+\psi_{ac}\cdot\psi_{ev}+\psi_{ac}\cdot\psi_{cv}+\psi_{ev}\cdot\psi_{cv}$	0.510392	0.64819	32319.8
227	$\overline{\boldsymbol{u}} \sim \psi_{ac}+\psi_{ce}+\psi_{ev}+\psi_{cv}+\psi_{ac}\cdot\psi_{ev}+\psi_{ac}\cdot\psi_{cv}+\psi_{ce}\cdot\psi_{ev}+\psi_{ce}\cdot\psi_{cv}$	0.508542	0.64819	32321
228	$\overline{\boldsymbol{u}} \sim \psi_{ac}+\psi_{ce}+\psi_{ev}+\psi_{cv}+\psi_{ac}\cdot\psi_{ev}+\psi_{ac}\cdot\psi_{cv}+\psi_{ce}\cdot\psi_{cv}+\psi_{ev}\cdot\psi_{cv}$	0.508877	0.64819	32321.4
229	$\overline{\boldsymbol{u}} \sim \psi_{ac}+\psi_{ce}+\psi_{ev}+\psi_{cv}+\psi_{ac}\cdot\psi_{ev}+\psi_{ac}\cdot\psi_{cv}$	0.510209	0.64818	32320
230	$\overline{\boldsymbol{u}} \sim \psi_{ac}+\psi_{ce}+\psi_{ev}+\psi_{cv}+\psi_{ac}\cdot\psi_{cv}+\psi_{ce}\cdot\psi_{ev}$	0.509614	0.64817	32321.2
231	$\overline{\boldsymbol{u}} \sim \psi_{ac}+\psi_{ce}+\psi_{ev}+\psi_{cv}+\psi_{ac}\cdot\psi_{ev}+\psi_{ac}\cdot\psi_{cv}+\psi_{ce}\cdot\psi_{cv}$	0.508687	0.64817	32321.6
232	$\overline{\boldsymbol{u}} \sim \psi_{ac}+\psi_{ce}+\psi_{ev}+\psi_{cv}+\psi_{ac}\cdot\psi_{cv}$	0.509757	0.64816	32321.1
233	$\overline{\boldsymbol{u}} \sim \psi_{ac}+\psi_{ce}+\psi_{ev}+\psi_{cv}+\psi_{ac}\cdot\psi_{cv}+\psi_{ce}\cdot\psi_{ev}+\psi_{ce}\cdot\psi_{cv}$	0.508085	0.64816	32322.8
234	$\overline{\boldsymbol{u}} \sim \psi_{ac}+\psi_{ce}+\psi_{ev}+\psi_{cv}+\psi_{ac}\cdot\psi_{cv}+\psi_{ce}\cdot\psi_{ev}+\psi_{ev}\cdot\psi_{cv}$	0.50963	0.64816	32322.6
235	$\overline{\boldsymbol{u}} \sim \psi_{ac}+\psi_{ce}+\psi_{ev}+\psi_{cv}+\psi_{ac}\cdot\psi_{cv}+\psi_{ce}\cdot\psi_{cv}$	0.508233	0.64815	32322.7
236	$\overline{\boldsymbol{u}} \sim \psi_{ac}+\psi_{ce}+\psi_{ev}+\psi_{cv}+\psi_{ac}\cdot\psi_{cv}+\psi_{ev}\cdot\psi_{cv}$	0.509778	0.64815	32322.5
237	$\overline{\boldsymbol{u}} \sim \psi_{ac}+\psi_{ce}+\psi_{ev}+\psi_{cv}+\psi_{ac}\cdot\psi_{cv}+\psi_{ce}\cdot\psi_{ev}+\psi_{ce}\cdot\psi_{cv}+\psi_{ev}\cdot\psi_{cv}$	0.508104	0.64815	32324.2
238	$\overline{\boldsymbol{u}} \sim \psi_{ac}+\psi_{ce}+\psi_{ev}+\psi_{cv}+\psi_{ac}\cdot\psi_{cv}+\psi_{ce}\cdot\psi_{cv}+\psi_{ev}\cdot\psi_{cv}$	0.508257	0.64814	32324.1
239	$\overline{\boldsymbol{u}} \sim \psi_{ev}+\psi_{cv}+\psi_{ac}\cdot\psi_{ce}+\psi_{ac}\cdot\psi_{ev}+\psi_{ac}\cdot\psi_{cv}+\psi_{ce}\cdot\psi_{cv}+\psi_{ev}\cdot\psi_{cv}$	0.234451	0.64735	32392.1

240	$\overline{\boldsymbol{u}} \sim \psi_{ev}+\psi_{cv}+\psi_{ac}\cdot\psi_{ce}+\psi_{ac}\cdot\psi_{ev}+\psi_{ac}\cdot\psi_{cv}+\psi_{ce}\cdot\psi_{cv}$	0.230844	0.64657	32458.5
241	$\overline{\boldsymbol{u}} \sim \psi_{ac}+\psi_{cv}+\psi_{ac}\cdot\psi_{ce}+\psi_{ac}\cdot\psi_{ev}+\psi_{ac}\cdot\psi_{cv}+\psi_{ce}\cdot\psi_{cv}+\psi_{ev}\cdot\psi_{cv}$	0.260597	0.64457	32630.8
242	$\overline{\boldsymbol{u}} \sim \psi_{ac}+\psi_{cv}+\psi_{ac}\cdot\psi_{ce}+\psi_{ac}\cdot\psi_{ev}+\psi_{ac}\cdot\psi_{cv}+\psi_{ce}\cdot\psi_{cv}$	0.26094	0.64437	32646.7
243	$\overline{\boldsymbol{u}} \sim \psi_{ce}+\psi_{ev}+\psi_{cv}+\psi_{ac}\cdot\psi_{ev}+\psi_{ac}\cdot\psi_{cv}+\psi_{ce}\cdot\psi_{ev}+\psi_{ce}\cdot\psi_{cv}+\psi_{ev}\cdot\psi_{cv}$	0.290019	0.64253	32805.5
244	$\overline{\boldsymbol{u}} \sim \psi_{ce}+\psi_{ev}+\psi_{cv}+\psi_{ac}\cdot\psi_{ev}+\psi_{ac}\cdot\psi_{cv}+\psi_{ce}\cdot\psi_{ev}+\psi_{ev}\cdot\psi_{cv}$	0.291019	0.6425	32806.6
245	$\overline{\boldsymbol{u}} \sim \psi_{ce}+\psi_{ev}+\psi_{cv}+\psi_{ac}\cdot\psi_{ev}+\psi_{ac}\cdot\psi_{cv}+\psi_{ce}\cdot\psi_{cv}+\psi_{ev}\cdot\psi_{cv}$	0.288505	0.64236	32819.1
246	$\overline{\boldsymbol{u}} \sim \psi_{ce}+\psi_{ev}+\psi_{cv}+\psi_{ac}\cdot\psi_{ev}+\psi_{ac}\cdot\psi_{cv}+\psi_{ev}\cdot\psi_{cv}$	0.289499	0.64233	32820.2
247	$\overline{\boldsymbol{u}} \sim \psi_{ev}+\psi_{cv}+\psi_{ac}\cdot\psi_{ce}+\psi_{ac}\cdot\psi_{ev}+\psi_{ac}\cdot\psi_{cv}+\psi_{ce}\cdot\psi_{ev}+\psi_{ev}\cdot\psi_{cv}$	0.218295	0.64225	32828.1
248	$\overline{\boldsymbol{u}} \sim \psi_{ce}+\psi_{ev}+\psi_{cv}+\psi_{ac}\cdot\psi_{ev}+\psi_{ac}\cdot\psi_{cv}+\psi_{ce}\cdot\psi_{ev}+\psi_{ce}\cdot\psi_{cv}$	0.283029	0.64199	32850.5
249	$\overline{\boldsymbol{u}} \sim \psi_{ce}+\psi_{ev}+\psi_{cv}+\psi_{ac}\cdot\psi_{ev}+\psi_{ac}\cdot\psi_{cv}+\psi_{ce}\cdot\psi_{ev}$	0.284018	0.64196	32851.6
250	$\overline{\boldsymbol{u}} \sim \psi_{ev}+\psi_{cv}+\psi_{ac}\cdot\psi_{ce}+\psi_{ac}\cdot\psi_{ev}+\psi_{ac}\cdot\psi_{cv}+\psi_{ce}\cdot\psi_{ev}$	0.21584	0.64192	32855
251	$\overline{\boldsymbol{u}} \sim \psi_{ce}+\psi_{ev}+\psi_{cv}+\psi_{ac}\cdot\psi_{ev}+\psi_{ac}\cdot\psi_{cv}+\psi_{ce}\cdot\psi_{cv}$	0.281927	0.64185	32861.1
252	$\overline{\boldsymbol{u}} \sim \psi_{ce}+\psi_{ev}+\psi_{cv}+\psi_{ac}\cdot\psi_{ev}+\psi_{ac}\cdot\psi_{cv}$	0.282911	0.64182	32862.2
253	$\overline{\boldsymbol{u}} \sim \psi_{ac}+\psi_{ev}+\psi_{cv}+\psi_{ac}\cdot\psi_{cv}+\psi_{ce}\cdot\psi_{ev}+\psi_{ce}\cdot\psi_{cv}$	0.380355	0.64014	33005.3
254	$\overline{\boldsymbol{u}} \sim \psi_{ac}+\psi_{ev}+\psi_{cv}+\psi_{ac}\cdot\psi_{ev}+\psi_{ac}\cdot\psi_{cv}+\psi_{ce}\cdot\psi_{ev}+\psi_{ce}\cdot\psi_{cv}$	0.380371	0.64013	33007.1
255	$\overline{\boldsymbol{u}} \sim \psi_{ac}+\psi_{ev}+\psi_{cv}+\psi_{ac}\cdot\psi_{cv}+\psi_{ce}\cdot\psi_{ev}+\psi_{ce}\cdot\psi_{cv}+\psi_{ev}\cdot\psi_{cv}$	0.380353	0.64013	33007.2
256	$\overline{\boldsymbol{u}} \sim \psi_{ac}+\psi_{ev}+\psi_{cv}+\psi_{ac}\cdot\psi_{ev}+\psi_{ac}\cdot\psi_{cv}+\psi_{ce}\cdot\psi_{ev}+\psi_{ce}\cdot\psi_{cv}+\psi_{ev}\cdot\psi_{cv}$	0.380369	0.64012	33009.1
257	$\overline{\boldsymbol{u}} \sim \psi_{ac}+\psi_{ce}+\psi_{cv}+\psi_{ac}\cdot\psi_{ev}+\psi_{ac}\cdot\psi_{cv}+\psi_{ce}\cdot\psi_{ev}+\psi_{ce}\cdot\psi_{cv}+\psi_{ev}\cdot\psi_{cv}$	0.362937	0.63901	33102.3
258	$\overline{\boldsymbol{u}} \sim \psi_{ac}+\psi_{ce}+\psi_{cv}+\psi_{ac}\cdot\psi_{ev}+\psi_{ac}\cdot\psi_{cv}+\psi_{ce}\cdot\psi_{ev}+\psi_{ev}\cdot\psi_{cv}$	0.364328	0.63899	33103.4
259	$\overline{\boldsymbol{u}} \sim \psi_{ac}+\psi_{ce}+\psi_{cv}+\psi_{ac}\cdot\psi_{ev}+\psi_{ac}\cdot\psi_{cv}+\psi_{ce}\cdot\psi_{ev}+\psi_{ce}\cdot\psi_{cv}$	0.363561	0.63883	33117
260	$\overline{\boldsymbol{u}} \sim \psi_{ac}+\psi_{ce}+\psi_{cv}+\psi_{ac}\cdot\psi_{ev}+\psi_{ac}\cdot\psi_{cv}+\psi_{ce}\cdot\psi_{ev}$	0.364955	0.6388	33118
261	$\overline{\boldsymbol{u}} \sim \psi_{ac}+\psi_{ce}+\psi_{cv}+\psi_{ac}\cdot\psi_{ev}+\psi_{ac}\cdot\psi_{cv}+\psi_{ce}\cdot\psi_{cv}+\psi_{ev}\cdot\psi_{cv}$	0.362549	0.63826	33165
262	$\overline{\boldsymbol{u}} \sim \psi_{ac}+\psi_{ce}+\psi_{cv}+\psi_{ac}\cdot\psi_{ev}+\psi_{ac}\cdot\psi_{cv}+\psi_{ev}\cdot\psi_{cv}$	0.363929	0.63823	33166
263	$\overline{\boldsymbol{u}} \sim \psi_{ac}+\psi_{ce}+\psi_{cv}+\psi_{ac}\cdot\psi_{ev}+\psi_{ac}\cdot\psi_{cv}+\psi_{ce}\cdot\psi_{cv}$	0.363172	0.63812	33175.7
264	$\overline{\boldsymbol{u}} \sim \psi_{ac}+\psi_{ce}+\psi_{cv}+\psi_{ac}\cdot\psi_{ev}+\psi_{ac}\cdot\psi_{cv}$	0.364554	0.63809	33176.7
265	$\overline{\boldsymbol{u}} \sim \psi_{ac}+\psi_{ev}+\psi_{cv}+\psi_{ac}\cdot\psi_{ev}+\psi_{ac}\cdot\psi_{cv}+\psi_{ce}\cdot\psi_{ev}$	0.344917	0.6361	33344.3
266	$\overline{\boldsymbol{u}} \sim \psi_{ac}+\psi_{ev}+\psi_{cv}+\psi_{ac}\cdot\psi_{ev}+\psi_{ac}\cdot\psi_{cv}+\psi_{ce}\cdot\psi_{ev}+\psi_{ev}\cdot\psi_{cv}$	0.344944	0.63609	33345.6

267	$\overline{\boldsymbol{u}} \sim \psi_{ac}+\psi_{ev}+\psi_{cv}+\psi_{ac}\cdot\psi_{cv}+\psi_{ce}\cdot\psi_{ev}$	0.344798	0.63607	33345.6
268	$\overline{\boldsymbol{u}} \sim \psi_{ac}+\psi_{ev}+\psi_{cv}+\psi_{ac}\cdot\psi_{cv}+\psi_{ce}\cdot\psi_{ev}+\psi_{ev}\cdot\psi_{cv}$	0.344799	0.63606	33347.6
269	$\overline{\boldsymbol{u}} \sim \psi_{ev}+\psi_{cv}+\psi_{ac}\cdot\psi_{ev}+\psi_{ac}\cdot\psi_{cv}+\psi_{ce}\cdot\psi_{ev}+\psi_{ce}\cdot\psi_{cv}+\psi_{ev}\cdot\psi_{cv}$	0.279578	0.63553	33392.6
270	$\overline{\boldsymbol{u}} \sim \psi_{ev}+\psi_{cv}+\psi_{ac}\cdot\psi_{ev}+\psi_{ac}\cdot\psi_{cv}+\psi_{ce}\cdot\psi_{ev}+\psi_{ce}\cdot\psi_{cv}$	0.274786	0.63519	33419.7
271	$\overline{\boldsymbol{u}} \sim \psi_{ce}+\psi_{ev}+\psi_{cv}+\psi_{ac}\cdot\psi_{cv}+\psi_{ce}\cdot\psi_{ev}+\psi_{ce}\cdot\psi_{cv}$	0.213044	0.63512	33425.9
272	$\overline{\boldsymbol{u}} \sim \psi_{ce}+\psi_{ev}+\psi_{cv}+\psi_{ac}\cdot\psi_{cv}+\psi_{ce}\cdot\psi_{ev}$	0.213701	0.63511	33425.8
273	$\overline{\boldsymbol{u}} \sim \psi_{ce}+\psi_{ev}+\psi_{cv}+\psi_{ac}\cdot\psi_{cv}+\psi_{ce}\cdot\psi_{ev}+\psi_{ce}\cdot\psi_{cv}+\psi_{ev}\cdot\psi_{cv}$	0.213051	0.63511	33427.3
274	$\overline{\boldsymbol{u}} \sim \psi_{ce}+\psi_{ev}+\psi_{cv}+\psi_{ac}\cdot\psi_{cv}+\psi_{ce}\cdot\psi_{cv}$	0.213057	0.6351	33426.3
275	$\overline{\boldsymbol{u}} \sim \psi_{ce}+\psi_{ev}+\psi_{cv}+\psi_{ac}\cdot\psi_{cv}+\psi_{ce}\cdot\psi_{ev}+\psi_{ev}\cdot\psi_{cv}$	0.213707	0.6351	33427.2
276	$\overline{\boldsymbol{u}} \sim \psi_{ce}+\psi_{ev}+\psi_{cv}+\psi_{ac}\cdot\psi_{cv}$	0.213713	0.63509	33426.2
277	$\overline{\boldsymbol{u}} \sim \psi_{ce}+\psi_{ev}+\psi_{cv}+\psi_{ac}\cdot\psi_{cv}+\psi_{ce}\cdot\psi_{cv}+\psi_{ev}\cdot\psi_{cv}$	0.213064	0.63509	33427.9
278	$\overline{\boldsymbol{u}} \sim \psi_{ce}+\psi_{ev}+\psi_{cv}+\psi_{ac}\cdot\psi_{cv}+\psi_{ev}\cdot\psi_{cv}$	0.213719	0.63508	33427.7
279	$\overline{\boldsymbol{u}} \sim \psi_{ac}+\psi_{ev}+\psi_{cv}+\psi_{ac}\cdot\psi_{ev}+\psi_{ac}\cdot\psi_{cv}+\psi_{ce}\cdot\psi_{cv}+\psi_{ev}\cdot\psi_{cv}$	0.355996	0.63487	33447.9
280	$\overline{\boldsymbol{u}} \sim \psi_{ac}+\psi_{ev}+\psi_{cv}+\psi_{ac}\cdot\psi_{ev}+\psi_{ac}\cdot\psi_{cv}+\psi_{ce}\cdot\psi_{cv}$	0.355939	0.63485	33447.8
281	$\overline{\boldsymbol{u}} \sim \psi_{ac}+\psi_{ev}+\psi_{cv}+\psi_{ac}\cdot\psi_{cv}+\psi_{ce}\cdot\psi_{cv}$	0.355838	0.63484	33448
282	$\overline{\boldsymbol{u}} \sim \psi_{ac}+\psi_{ev}+\psi_{cv}+\psi_{ac}\cdot\psi_{cv}+\psi_{ce}\cdot\psi_{cv}+\psi_{ev}\cdot\psi_{cv}$	0.35585	0.63483	33449.5
283	$\overline{\boldsymbol{u}} \sim \psi_{ev}+\psi_{cv}+\psi_{ac}\cdot\psi_{ce}+\psi_{ac}\cdot\psi_{cv}+\psi_{ce}\cdot\psi_{cv}$	0.185946	0.63447	33479.2
284	$\overline{\boldsymbol{u}} \sim \psi_{ev}+\psi_{cv}+\psi_{ac}\cdot\psi_{ce}+\psi_{ac}\cdot\psi_{cv}+\psi_{ce}\cdot\psi_{cv}+\psi_{ev}\cdot\psi_{cv}$	0.185952	0.63446	33480.6
285	$\overline{\boldsymbol{u}} \sim \psi_{ac}+\psi_{cv}+\psi_{ac}\cdot\psi_{ce}+\psi_{ac}\cdot\psi_{ev}+\psi_{ac}\cdot\psi_{cv}+\psi_{ce}\cdot\psi_{ev}$	0.262494	0.63347	33562.9
286	$\overline{\boldsymbol{u}} \sim \psi_{ac}+\psi_{cv}+\psi_{ac}\cdot\psi_{ce}+\psi_{ac}\cdot\psi_{ev}+\psi_{ac}\cdot\psi_{cv}+\psi_{ce}\cdot\psi_{ev}+\psi_{ev}\cdot\psi_{cv}$	0.262415	0.63347	33564
287	$\overline{\boldsymbol{u}} \sim \psi_{ev}+\psi_{cv}+\psi_{ac}\cdot\psi_{ce}+\psi_{ac}\cdot\psi_{cv}+\psi_{ce}\cdot\psi_{ev}$	0.17833	0.63272	33623.5
288	$\overline{\boldsymbol{u}} \sim \psi_{ev}+\psi_{cv}+\psi_{ac}\cdot\psi_{ce}+\psi_{ac}\cdot\psi_{cv}+\psi_{ce}\cdot\psi_{ev}+\psi_{ev}\cdot\psi_{cv}$	0.178294	0.63272	33625
289	$\overline{\boldsymbol{u}} \sim \psi_{ev}+\psi_{cv}+\psi_{ac}\cdot\psi_{ev}+\psi_{ac}\cdot\psi_{cv}+\psi_{ce}\cdot\psi_{cv}+\psi_{ev}\cdot\psi_{cv}$	0.282538	0.63164	33713.7
290	$\overline{\boldsymbol{u}} \sim \psi_{ev}+\psi_{cv}+\psi_{ac}\cdot\psi_{ev}+\psi_{ac}\cdot\psi_{cv}+\psi_{ce}\cdot\psi_{ev}+\psi_{ev}\cdot\psi_{cv}$	0.263288	0.63159	33718.2
291	$\overline{\boldsymbol{u}} \sim \psi_{ev}+\psi_{cv}+\psi_{ac}\cdot\psi_{ev}+\psi_{ac}\cdot\psi_{cv}+\psi_{ce}\cdot\psi_{ev}$	0.25973	0.63135	33737
292	$\overline{\boldsymbol{u}} \sim \psi_{ev}+\psi_{cv}+\psi_{ac}\cdot\psi_{ev}+\psi_{ac}\cdot\psi_{cv}+\psi_{ce}\cdot\psi_{cv}$	0.277444	0.63124	33745.7
293	$\overline{\boldsymbol{u}} \sim \psi_{ev}+\psi_{cv}+\psi_{ac}\cdot\psi_{cv}+\psi_{ce}\cdot\psi_{ev}+\psi_{ce}\cdot\psi_{cv}$	0.216533	0.62982	33862.6

294	$\overline{\boldsymbol{u}} \sim \psi_{ev}+\psi_{cv}+\psi_{ac}\cdot\psi_{cv}+\psi_{ce}\cdot\psi_{ev}+\psi_{ce}\cdot\psi_{cv}+\psi_{ev}\cdot\psi_{cv}$	0.216537	0.6298	33864.6
295	$\overline{\boldsymbol{u}} \sim \psi_{ac}+\psi_{cv}+\psi_{ac}\cdot\psi_{ce}+\psi_{ac}\cdot\psi_{cv}+\psi_{ce}\cdot\psi_{ev}+\psi_{ce}\cdot\psi_{cv}+\psi_{ev}\cdot\psi_{cv}$	0.211476	0.62975	33870.1
296	$\overline{\boldsymbol{u}} \sim \psi_{ac}+\psi_{cv}+\psi_{ac}\cdot\psi_{ev}+\psi_{ac}\cdot\psi_{cv}+\psi_{ce}\cdot\psi_{ev}+\psi_{ce}\cdot\psi_{cv}+\psi_{ev}\cdot\psi_{cv}$	0.330133	0.62923	33912.4
297	$\overline{\boldsymbol{u}} \sim \psi_{ac}+\psi_{cv}+\psi_{ac}\cdot\psi_{ev}+\psi_{ac}\cdot\psi_{cv}+\psi_{ce}\cdot\psi_{ev}+\psi_{ce}\cdot\psi_{cv}$	0.330583	0.62915	33918.5
298	$\overline{\boldsymbol{u}} \sim \psi_{ac}+\psi_{ce}+\psi_{cv}+\psi_{ac}\cdot\psi_{cv}+\psi_{ce}\cdot\psi_{ev}+\psi_{ce}\cdot\psi_{cv}+\psi_{ev}\cdot\psi_{cv}$	0.291779	0.62792	34019.7
299	$\overline{\boldsymbol{u}} \sim \psi_{ac}+\psi_{ce}+\psi_{cv}+\psi_{ac}\cdot\psi_{cv}+\psi_{ce}\cdot\psi_{ev}+\psi_{ev}\cdot\psi_{cv}$	0.293111	0.62785	34024
300	$\overline{\boldsymbol{u}} \sim \psi_{ev}+\psi_{cv}+\psi_{ac}\cdot\psi_{cv}+\psi_{ce}\cdot\psi_{ev}$	0.209154	0.62656	34127.2
301	$\overline{\boldsymbol{u}} \sim \psi_{ev}+\psi_{cv}+\psi_{ac}\cdot\psi_{cv}+\psi_{ce}\cdot\psi_{ev}+\psi_{ev}\cdot\psi_{cv}$	0.209152	0.62655	34129.2
302	$\overline{\boldsymbol{u}} \sim \psi_{ac}+\psi_{cv}+\psi_{ac}\cdot\psi_{ev}+\psi_{ac}\cdot\psi_{cv}+\psi_{ce}\cdot\psi_{cv}+\psi_{ev}\cdot\psi_{cv}$	0.321926	0.62638	34144
303	$\overline{\boldsymbol{u}} \sim \psi_{ac}+\psi_{cv}+\psi_{ac}\cdot\psi_{ev}+\psi_{ac}\cdot\psi_{cv}+\psi_{ce}\cdot\psi_{cv}$	0.322279	0.62623	34154.8
304	$\overline{\boldsymbol{u}} \sim \psi_{ev}+\psi_{cv}+\psi_{ac}\cdot\psi_{cv}+\psi_{ce}\cdot\psi_{cv}$	0.219534	0.62586	34184.4
305	$\overline{\boldsymbol{u}} \sim \psi_{ev}+\psi_{cv}+\psi_{ac}\cdot\psi_{cv}+\psi_{ce}\cdot\psi_{cv}+\psi_{ev}\cdot\psi_{cv}$	0.21954	0.62585	34185.9
306	$\overline{\boldsymbol{u}} \sim \psi_{ac}+\psi_{cv}+\psi_{ac}\cdot\psi_{ce}+\psi_{ac}\cdot\psi_{cv}+\psi_{ce}\cdot\psi_{cv}+\psi_{ev}\cdot\psi_{cv}$	0.225269	0.62517	34242.2
307	$\overline{\boldsymbol{u}} \sim \psi_{ac}+\psi_{cv}+\psi_{ac}\cdot\psi_{ev}+\psi_{ac}\cdot\psi_{cv}+\psi_{ce}\cdot\psi_{ev}+\psi_{ev}\cdot\psi_{cv}$	0.306744	0.6236	34369
308	$\overline{\boldsymbol{u}} \sim \psi_{ac}+\psi_{cv}+\psi_{ac}\cdot\psi_{ev}+\psi_{ac}\cdot\psi_{cv}+\psi_{ce}\cdot\psi_{ev}$	0.307096	0.62356	34371.2
309	$\overline{\boldsymbol{u}} \sim \psi_{ce}+\psi_{cv}+\psi_{ac}\cdot\psi_{ce}+\psi_{ac}\cdot\psi_{ev}+\psi_{ac}\cdot\psi_{cv}+\psi_{ce}\cdot\psi_{cv}$	0.120543	0.62042	34624
310	$\overline{\boldsymbol{u}} \sim \psi_{ce}+\psi_{cv}+\psi_{ac}\cdot\psi_{ev}+\psi_{ac}\cdot\psi_{cv}+\psi_{ce}\cdot\psi_{ev}+\psi_{ce}\cdot\psi_{cv}+\psi_{ev}\cdot\psi_{cv}$	0.199674	0.62027	34637
311	$\overline{\boldsymbol{u}} \sim \psi_{ce}+\psi_{cv}+\psi_{ac}\cdot\psi_{ev}+\psi_{ac}\cdot\psi_{cv}+\psi_{ce}\cdot\psi_{ev}+\psi_{ev}\cdot\psi_{cv}$	0.20031	0.62025	34637.7
312	$\overline{\boldsymbol{u}} \sim \psi_{ce}+\psi_{cv}+\psi_{ac}\cdot\psi_{cv}+\psi_{ce}\cdot\psi_{ev}+\psi_{ce}\cdot\psi_{cv}+\psi_{ev}\cdot\psi_{cv}$	0.202551	0.62021	34640.8
313	$\overline{\boldsymbol{u}} \sim \psi_{ce}+\psi_{cv}+\psi_{ac}\cdot\psi_{cv}+\psi_{ce}\cdot\psi_{ev}+\psi_{ev}\cdot\psi_{cv}$	0.203328	0.62018	34641.8
314	$\overline{\boldsymbol{u}} \sim \psi_{ce}+\psi_{cv}+\psi_{ac}\cdot\psi_{ce}+\psi_{ac}\cdot\psi_{ev}+\psi_{ac}\cdot\psi_{cv}$	0.120894	0.61879	34753.3
315	$\overline{\boldsymbol{u}} \sim \psi_{ac}+\psi_{ce}+\psi_{cv}+\psi_{ac}\cdot\psi_{cv}+\psi_{ce}\cdot\psi_{cv}+\psi_{ev}\cdot\psi_{cv}$	0.269196	0.61592	34981.3
316	$\overline{\boldsymbol{u}} \sim \psi_{ac}+\psi_{ce}+\psi_{cv}+\psi_{ac}\cdot\psi_{cv}+\psi_{ev}\cdot\psi_{cv}$	0.270493	0.61584	34986.8
317	$\overline{\boldsymbol{u}} \sim \psi_{ce}+\psi_{cv}+\psi_{ac}\cdot\psi_{ev}+\psi_{ac}\cdot\psi_{cv}+\psi_{ce}\cdot\psi_{cv}+\psi_{ev}\cdot\psi_{cv}$	0.184281	0.61248	35252.2
318	$\overline{\boldsymbol{u}} \sim \psi_{ce}+\psi_{cv}+\psi_{ac}\cdot\psi_{ev}+\psi_{ac}\cdot\psi_{cv}+\psi_{ev}\cdot\psi_{cv}$	0.184704	0.61247	35251.6
319	$\overline{\boldsymbol{u}} \sim \psi_{cv}+\psi_{ac}\cdot\psi_{ce}+\psi_{ac}\cdot\psi_{ev}+\psi_{ac}\cdot\psi_{cv}+\psi_{ce}\cdot\psi_{ev}+\psi_{ce}\cdot\psi_{cv}+\psi_{ev}\cdot\psi_{cv}$	0.165805	0.61132	35344.1
320	$\overline{\boldsymbol{u}} \sim \psi_{ce}+\psi_{cv}+\psi_{ac}\cdot\psi_{ev}+\psi_{ac}\cdot\psi_{cv}+\psi_{ce}\cdot\psi_{ev}$	0.177	0.61092	35373

321	$\overline{\boldsymbol{u}} \sim \psi_{ce}+\psi_{cv}+\psi_{ac}\cdot\psi_{ev}+\psi_{ac}\cdot\psi_{cv}+\psi_{ce}\cdot\psi_{ev}+\psi_{ce}\cdot\psi_{cv}$	0.176643	0.61092	35374
322	$\overline{\boldsymbol{u}} \sim \psi_{cv}+\psi_{ac}\cdot\psi_{ce}+\psi_{ac}\cdot\psi_{ev}+\psi_{ac}\cdot\psi_{cv}+\psi_{ce}\cdot\psi_{cv}+\psi_{ev}\cdot\psi_{cv}$	0.171474	0.61047	35408.8
323	$\overline{\boldsymbol{u}} \sim \psi_{ac}+\psi_{ev}+\psi_{cv}+\psi_{ac}\cdot\psi_{ce}+\psi_{ac}\cdot\psi_{cv}$	0.339548	0.60995	35448.5
324	$\overline{\boldsymbol{u}} \sim \psi_{ac}+\psi_{ev}+\psi_{cv}+\psi_{ac}\cdot\psi_{ce}+\psi_{ac}\cdot\psi_{ev}+\psi_{ac}\cdot\psi_{cv}$	0.339602	0.60995	35449.4
325	$\overline{\boldsymbol{u}} \sim \psi_{ac}+\psi_{ev}+\psi_{cv}+\psi_{ac}\cdot\psi_{ce}+\psi_{ac}\cdot\psi_{ev}+\psi_{ac}\cdot\psi_{cv}+\psi_{ev}\cdot\psi_{cv}$	0.339641	0.60995	35450.1
326	$\overline{\boldsymbol{u}} \sim \psi_{ac}+\psi_{ev}+\psi_{cv}+\psi_{ac}\cdot\psi_{ce}+\psi_{ac}\cdot\psi_{cv}+\psi_{ev}\cdot\psi_{cv}$	0.339558	0.60994	35450
327	$\overline{\boldsymbol{u}} \sim \psi_{cv}+\psi_{ac}\cdot\psi_{ce}+\psi_{ac}\cdot\psi_{cv}+\psi_{ce}\cdot\psi_{ev}+\psi_{ce}\cdot\psi_{cv}+\psi_{ev}\cdot\psi_{cv}$	0.17124	0.60981	35460.7
328	$\overline{\boldsymbol{u}} \sim \psi_{cv}+\psi_{ac}\cdot\psi_{ce}+\psi_{ac}\cdot\psi_{cv}+\psi_{ce}\cdot\psi_{cv}+\psi_{ev}\cdot\psi_{cv}$	0.175101	0.60935	35494.8
329	$\overline{\boldsymbol{u}} \sim \psi_{ce}+\psi_{cv}+\psi_{ac}\cdot\psi_{cv}+\psi_{ce}\cdot\psi_{cv}+\psi_{ev}\cdot\psi_{cv}$	0.198373	0.60919	35507.6
330	$\overline{\boldsymbol{u}} \sim \psi_{ce}+\psi_{cv}+\psi_{ac}\cdot\psi_{cv}+\psi_{ev}\cdot\psi_{cv}$	0.199172	0.60916	35509
331	$\overline{\boldsymbol{u}} \sim \psi_{ev}+\psi_{cv}+\psi_{ac}\cdot\psi_{ce}+\psi_{ac}\cdot\psi_{ev}+\psi_{ac}\cdot\psi_{cv}+\psi_{ev}\cdot\psi_{cv}$	0.284503	0.60816	35588.4
332	$\overline{\boldsymbol{u}} \sim \psi_{ac}+\psi_{ev}+\psi_{cv}+\psi_{ac}\cdot\psi_{ce}+\psi_{ac}\cdot\psi_{ev}+\psi_{ce}\cdot\psi_{ev}+\psi_{ce}\cdot\psi_{cv}+\psi_{ev}\cdot\psi_{cv}$	0.10349	0.60808	35596.3
333	$\overline{\boldsymbol{u}} \sim \psi_{ev}+\psi_{cv}+\psi_{ac}\cdot\psi_{ce}+\psi_{ac}\cdot\psi_{ev}+\psi_{ac}\cdot\psi_{cv}$	0.280486	0.60794	35604.5
334	$\overline{\boldsymbol{u}} \sim \psi_{ac}+\psi_{ev}+\psi_{cv}+\psi_{ac}\cdot\psi_{ce}+\psi_{ac}\cdot\psi_{ev}+\psi_{ce}\cdot\psi_{ev}+\psi_{ce}\cdot\psi_{cv}$	0.104106	0.6076	35632.6
335	$\overline{\boldsymbol{u}} \sim \psi_{ce}+\psi_{ac}\cdot\psi_{ce}+\psi_{ac}\cdot\psi_{ev}+\psi_{ac}\cdot\psi_{cv}+\psi_{ce}\cdot\psi_{cv}+\psi_{ev}\cdot\psi_{cv}$	0.100364	0.60751	35639
336	$\overline{\boldsymbol{u}} \sim \psi_{ac}+\psi_{ce}+\psi_{cv}+\psi_{ac}\cdot\psi_{cv}+\psi_{ce}\cdot\psi_{ev}+\psi_{ce}\cdot\psi_{cv}$	0.247113	0.60749	35640.1
337	$\overline{\boldsymbol{u}} \sim \psi_{ce}+\psi_{ac}\cdot\psi_{ce}+\psi_{ac}\cdot\psi_{ev}+\psi_{ac}\cdot\psi_{cv}+\psi_{ev}\cdot\psi_{cv}$	0.100038	0.60748	35639.8
338	$\overline{\boldsymbol{u}} \sim \psi_{ac}+\psi_{ce}+\psi_{cv}+\psi_{ac}\cdot\psi_{cv}+\psi_{ce}\cdot\psi_{ev}$	0.248447	0.60738	35648
339	$\overline{\boldsymbol{u}} \sim \psi_{ac}+\psi_{ev}+\psi_{cv}+\psi_{ac}\cdot\psi_{ce}+\psi_{ce}\cdot\psi_{ev}+\psi_{ce}\cdot\psi_{cv}+\psi_{ev}\cdot\psi_{cv}$	0.10472	0.60666	35705.7
340	$\overline{\boldsymbol{u}} \sim \psi_{ac}+\psi_{ev}+\psi_{cv}+\psi_{ac}\cdot\psi_{ce}+\psi_{ce}\cdot\psi_{ev}+\psi_{ce}\cdot\psi_{cv}$	0.104858	0.60659	35709.4
341	$\overline{\boldsymbol{u}} \sim \psi_{ac}+\psi_{cv}+\psi_{ac}\cdot\psi_{cv}+\psi_{ce}\cdot\psi_{ev}+\psi_{ce}\cdot\psi_{cv}+\psi_{ev}\cdot\psi_{cv}$	0.263618	0.6054	35801.2
342	$\overline{\boldsymbol{u}} \sim \psi_{ev}+\psi_{cv}+\psi_{ac}\cdot\psi_{ce}+\psi_{ac}\cdot\psi_{cv}$	0.237239	0.60537	35802
343	$\overline{\boldsymbol{u}} \sim \psi_{ev}+\psi_{cv}+\psi_{ac}\cdot\psi_{ce}+\psi_{ac}\cdot\psi_{cv}+\psi_{ev}\cdot\psi_{cv}$	0.237244	0.60536	35803.6
344	$\overline{\boldsymbol{u}} \sim \psi_{ac}+\psi_{cv}+\psi_{ac}\cdot\psi_{cv}+\psi_{ce}\cdot\psi_{cv}+\psi_{ev}\cdot\psi_{cv}$	0.261783	0.60486	35841.6
345	$\overline{\boldsymbol{u}} \sim \psi_{cv}+\psi_{ac}\cdot\psi_{ev}+\psi_{ac}\cdot\psi_{cv}+\psi_{ce}\cdot\psi_{cv}+\psi_{ev}\cdot\psi_{cv}$	0.18879	0.60381	35922.4
346	$\overline{\boldsymbol{u}} \sim \psi_{cv}+\psi_{ac}\cdot\psi_{ev}+\psi_{ac}\cdot\psi_{cv}+\psi_{ce}\cdot\psi_{ev}+\psi_{ce}\cdot\psi_{cv}+\psi_{ev}\cdot\psi_{cv}$	0.189404	0.60381	35923.1
347	$\overline{\boldsymbol{u}} \sim \psi_{ac}+\psi_{cv}+\psi_{ac}\cdot\psi_{ce}+\psi_{ac}\cdot\psi_{ev}+\psi_{ac}\cdot\psi_{cv}+\psi_{ev}\cdot\psi_{cv}$	0.310927	0.6019	36069.3

348	$\overline{\boldsymbol{u}} \sim \psi_{ce}+\psi_{cv}+\psi_{ac}\cdot\psi_{cv}+\psi_{ce}\cdot\psi_{ev}+\psi_{ce}\cdot\psi_{cv}$	0.192175	0.60181	36074.9
349	$\overline{\boldsymbol{u}} \sim \psi_{ac}+\psi_{cv}+\psi_{ac}\cdot\psi_{ce}+\psi_{ac}\cdot\psi_{ev}+\psi_{ac}\cdot\psi_{cv}$	0.311253	0.60177	36077.9
350	$\overline{\boldsymbol{u}} \sim \psi_{ce}+\psi_{cv}+\psi_{ac}\cdot\psi_{cv}+\psi_{ce}\cdot\psi_{ev}$	0.193084	0.60176	36077.6
351	$\overline{\boldsymbol{u}} \sim \psi_{cv}+\psi_{ac}\cdot\psi_{cv}+\psi_{ce}\cdot\psi_{ev}+\psi_{ce}\cdot\psi_{cv}+\psi_{ev}\cdot\psi_{cv}$	0.206521	0.6004	36182
352	$\overline{\boldsymbol{u}} \sim \psi_{cv}+\psi_{ac}\cdot\psi_{cv}+\psi_{ce}\cdot\psi_{cv}+\psi_{ev}\cdot\psi_{cv}$	0.204745	0.59977	36229.1
353	$\overline{\boldsymbol{u}} \sim \psi_{ce}+\psi_{cv}+\psi_{ac}\cdot\psi_{ev}+\psi_{ac}\cdot\psi_{cv}$	0.158044	0.59773	36383.1
354	$\overline{\boldsymbol{u}} \sim \psi_{ce}+\psi_{cv}+\psi_{ac}\cdot\psi_{ev}+\psi_{ac}\cdot\psi_{cv}+\psi_{ce}\cdot\psi_{cv}$	0.158044	0.59772	36385.1
355	$\overline{\boldsymbol{u}} \sim \psi_{ac}+\psi_{ev}+\psi_{cv}+\psi_{ac}\cdot\psi_{ev}+\psi_{ac}\cdot\psi_{cv}$	0.427621	0.59267	36763.7
356	$\overline{\boldsymbol{u}} \sim \psi_{ac}+\psi_{ev}+\psi_{cv}+\psi_{ac}\cdot\psi_{ev}+\psi_{ac}\cdot\psi_{cv}+\psi_{ev}\cdot\psi_{cv}$	0.427725	0.59267	36764.4
357	$\overline{\boldsymbol{u}} \sim \psi_{ac}+\psi_{ev}+\psi_{cv}+\psi_{ac}\cdot\psi_{cv}$	0.427416	0.59266	36763.2
358	$\overline{\boldsymbol{u}} \sim \psi_{ac}+\psi_{ev}+\psi_{cv}+\psi_{ac}\cdot\psi_{cv}+\psi_{ev}\cdot\psi_{cv}$	0.427432	0.59266	36764.7
359	$\overline{\boldsymbol{u}} \sim \psi_{cv}+\psi_{ac}\cdot\psi_{ce}+\psi_{ac}\cdot\psi_{ev}+\psi_{ac}\cdot\psi_{cv}+\psi_{ce}\cdot\psi_{ev}+\psi_{ce}\cdot\psi_{cv}$	0.157678	0.59251	36776.3
360	$\overline{\boldsymbol{u}} \sim \psi_{cv}+\psi_{ac}\cdot\psi_{ce}+\psi_{ac}\cdot\psi_{ev}+\psi_{ac}\cdot\psi_{cv}+\psi_{ce}\cdot\psi_{cv}$	0.154229	0.59219	36799.7
361	$\overline{\boldsymbol{u}} \sim \psi_{cv}+\psi_{ac}\cdot\psi_{ev}+\psi_{ac}\cdot\psi_{cv}+\psi_{ce}\cdot\psi_{ev}+\psi_{ce}\cdot\psi_{cv}$	0.167264	0.59111	36879.3
362	$\overline{\boldsymbol{u}} \sim \psi_{cv}+\psi_{ac}\cdot\psi_{ce}+\psi_{ac}\cdot\psi_{ev}+\psi_{ac}\cdot\psi_{cv}+\psi_{ce}\cdot\psi_{ev}+\psi_{ev}\cdot\psi_{cv}$	0.176229	0.59063	36916.2
363	$\overline{\boldsymbol{u}} \sim \psi_{cv}+\psi_{ac}\cdot\psi_{ev}+\psi_{ac}\cdot\psi_{cv}+\psi_{ce}\cdot\psi_{cv}$	0.162864	0.59025	36942.6
364	$\overline{\boldsymbol{u}} \sim \psi_{cv}+\psi_{ac}\cdot\psi_{ev}+\psi_{ac}\cdot\psi_{cv}+\psi_{ce}\cdot\psi_{ev}+\psi_{ev}\cdot\psi_{cv}$	0.169507	0.59009	36955.3
365	$\overline{\boldsymbol{u}} \sim \psi_{ev}+\psi_{cv}+\psi_{ac}\cdot\psi_{ev}+\psi_{ac}\cdot\psi_{cv}+\psi_{ev}\cdot\psi_{cv}$	0.272383	0.58842	37078.5
366	$\overline{\boldsymbol{u}} \sim \psi_{ev}+\psi_{cv}+\psi_{ac}\cdot\psi_{ev}+\psi_{ac}\cdot\psi_{cv}$	0.266727	0.58804	37105.3
367	$\overline{\boldsymbol{u}} \sim \psi_{cv}+\psi_{ac}\cdot\psi_{ce}+\psi_{ac}\cdot\psi_{ev}+\psi_{ac}\cdot\psi_{cv}+\psi_{ev}\cdot\psi_{cv}$	0.196423	0.58589	37264.5
368	$\overline{\boldsymbol{u}} \sim \psi_{ac}+\psi_{cv}+\psi_{ac}\cdot\psi_{ce}+\psi_{ac}\cdot\psi_{cv}+\psi_{ce}\cdot\psi_{ev}+\psi_{ev}\cdot\psi_{cv}$	0.238587	0.58519	37316.6
369	$\overline{\boldsymbol{u}} \sim \psi_{ac}+\psi_{cv}+\psi_{ac}\cdot\psi_{cv}+\psi_{ce}\cdot\psi_{ev}+\psi_{ev}\cdot\psi_{cv}$	0.236451	0.58515	37318.8
370	$\overline{\boldsymbol{u}} \sim \psi_{ac}+\psi_{cv}+\psi_{ac}\cdot\psi_{ce}+\psi_{ac}\cdot\psi_{ev}+\psi_{ce}\cdot\psi_{ev}+\psi_{ce}\cdot\psi_{cv}$	0.113848	0.58504	37327.6
371	$\overline{\boldsymbol{u}} \sim \psi_{ac}+\psi_{cv}+\psi_{ac}\cdot\psi_{ce}+\psi_{ac}\cdot\psi_{ev}+\psi_{ce}\cdot\psi_{ev}+\psi_{ce}\cdot\psi_{cv}+\psi_{ev}\cdot\psi_{cv}$	0.113846	0.58503	37329.6
372	$\overline{\boldsymbol{u}} \sim \psi_{ac}+\psi_{cv}+\psi_{ac}\cdot\psi_{ev}+\psi_{ac}\cdot\psi_{cv}+\psi_{ev}\cdot\psi_{cv}$	0.336656	0.58383	37415.1
373	$\overline{\boldsymbol{u}} \sim \psi_{ac}+\psi_{cv}+\psi_{ac}\cdot\psi_{ev}+\psi_{ac}\cdot\psi_{cv}$	0.337209	0.5837	37423.1
374	$\overline{\boldsymbol{u}} \sim \psi_{ev}+\psi_{cv}+\psi_{ac}\cdot\psi_{cv}$	0.207914	0.58328	37453.1

375	$\overline{\boldsymbol{u}} \sim \psi_{ev}+\psi_{cv}+\psi_{ac}\cdot\psi_{cv}+\psi_{ev}\cdot\psi_{cv}$	0.20792	0.58327	37454.7
376	$\overline{\boldsymbol{u}} \sim \psi_{cv}+\psi_{ac}\cdot\psi_{ce}+\psi_{ac}\cdot\psi_{cv}+\psi_{ce}\cdot\psi_{ev}+\psi_{ev}\cdot\psi_{cv}$	0.20152	0.58148	37585.8
377	$\overline{\boldsymbol{u}} \sim \psi_{cv}+\psi_{ac}\cdot\psi_{cv}+\psi_{ce}\cdot\psi_{ev}+\psi_{ev}\cdot\psi_{cv}$	0.19062	0.58056	37651.3
378	$\overline{\boldsymbol{u}} \sim \psi_{ac}+\psi_{cv}+\psi_{ac}\cdot\psi_{ce}+\psi_{ac}\cdot\psi_{cv}+\psi_{ev}\cdot\psi_{cv}$	0.255851	0.58049	37657.2
379	$\overline{\boldsymbol{u}} \sim \psi_{cv}+\psi_{ac}\cdot\psi_{ce}+\psi_{ac}\cdot\psi_{cv}+\psi_{ev}\cdot\psi_{cv}$	0.21951	0.57868	37786.9
380	$\overline{\boldsymbol{u}} \sim \psi_{cv}+\psi_{ac}\cdot\psi_{ce}+\psi_{ac}\cdot\psi_{ev}+\psi_{ac}\cdot\psi_{cv}+\psi_{ce}\cdot\psi_{ev}$	0.165825	0.57742	37878.4
381	$\overline{\boldsymbol{u}} \sim \psi_{cv}+\psi_{ac}\cdot\psi_{ce}+\psi_{ac}\cdot\psi_{ev}+\psi_{ac}\cdot\psi_{cv}$	0.173368	0.57672	37927.8
382	$\overline{\boldsymbol{u}} \sim \psi_{cv}+\psi_{ac}\cdot\psi_{ev}+\psi_{ac}\cdot\psi_{cv}+\psi_{ce}\cdot\psi_{ev}$	0.146084	0.57204	38261.6
383	$\overline{\boldsymbol{u}} \sim \psi_{ac}+\psi_{cv}+\psi_{ac}\cdot\psi_{cv}+\psi_{ev}\cdot\psi_{cv}$	0.255804	0.56211	38957.4
384	$\overline{\boldsymbol{u}} \sim \psi_{cv}+\psi_{ac}\cdot\psi_{ev}+\psi_{ac}\cdot\psi_{cv}+\psi_{ev}\cdot\psi_{cv}$	0.176444	0.56206	38960.6
385	$\overline{\boldsymbol{u}} \sim \psi_{cv}+\psi_{ac}\cdot\psi_{cv}+\psi_{ev}\cdot\psi_{cv}$	0.194597	0.55752	39272.6
386	$\overline{\boldsymbol{u}} \sim \psi_{ac}+\psi_{ev}+\psi_{ac}\cdot\psi_{ce}+\psi_{ac}\cdot\psi_{ev}+\psi_{ac}\cdot\psi_{cv}+\psi_{ce}\cdot\psi_{ev}+\psi_{ce}\cdot\psi_{cv}+\psi_{ev}\cdot\psi_{cv}$	0.119948	0.552	39653.6
387	$\overline{\boldsymbol{u}} \sim \psi_{ac}+\psi_{ev}+\psi_{ac}\cdot\psi_{ce}+\psi_{ac}\cdot\psi_{ev}+\psi_{ce}\cdot\psi_{ev}+\psi_{ce}\cdot\psi_{cv}+\psi_{ev}\cdot\psi_{cv}$	0.11269	0.55103	39718.8
388	$\overline{\boldsymbol{u}} \sim \psi_{ac}+\psi_{ev}+\psi_{ac}\cdot\psi_{ce}+\psi_{ac}\cdot\psi_{cv}+\psi_{ce}\cdot\psi_{ev}+\psi_{ce}\cdot\psi_{cv}+\psi_{ev}\cdot\psi_{cv}$	0.117383	0.55091	39726.9
389	$\overline{\boldsymbol{u}} \sim \psi_{ac}+\psi_{ev}+\psi_{ac}\cdot\psi_{ce}+\psi_{ce}\cdot\psi_{ev}+\psi_{ce}\cdot\psi_{cv}+\psi_{ev}\cdot\psi_{cv}$	0.112121	0.55033	39764.5
390	$\overline{\boldsymbol{u}} \sim \psi_{cv}+\psi_{ac}\cdot\psi_{ev}+\psi_{ac}\cdot\psi_{cv}$	0.151489	0.54972	39803
391	$\overline{\boldsymbol{u}} \sim \psi_{ac}+\psi_{cv}+\psi_{ac}\cdot\psi_{ce}+\psi_{ac}\cdot\psi_{cv}+\psi_{ce}\cdot\psi_{ev}+\psi_{ce}\cdot\psi_{cv}$	0.214561	0.54879	39868.7
392	$\overline{\boldsymbol{u}} \sim \psi_{ac}+\psi_{cv}+\psi_{ac}\cdot\psi_{cv}+\psi_{ce}\cdot\psi_{ev}+\psi_{ce}\cdot\psi_{cv}$	0.223977	0.54717	39976.2
393	$\overline{\boldsymbol{u}} \sim \psi_{cv}+\psi_{ac}\cdot\psi_{ce}+\psi_{ac}\cdot\psi_{cv}+\psi_{ce}\cdot\psi_{ev}+\psi_{ce}\cdot\psi_{cv}$	0.192547	0.54525	40104.6
394	$\overline{\boldsymbol{u}} \sim \psi_{cv}+\psi_{ac}\cdot\psi_{cv}+\psi_{ce}\cdot\psi_{ev}+\psi_{ce}\cdot\psi_{cv}$	0.197669	0.54507	40115.7
395	$\overline{\boldsymbol{u}} \sim \psi_{ac}+\psi_{ac}\cdot\psi_{ce}+\psi_{ac}\cdot\psi_{ev}+\psi_{ac}\cdot\psi_{cv}+\psi_{ce}\cdot\psi_{ev}+\psi_{ce}\cdot\psi_{cv}+\psi_{ev}\cdot\psi_{cv}$	0.126301	0.53698	40653.1
396	$\overline{\boldsymbol{u}} \sim \psi_{ac}+\psi_{ce}+\psi_{ev}+\psi_{ac}\cdot\psi_{ev}+\psi_{ac}\cdot\psi_{cv}+\psi_{ce}\cdot\psi_{ev}+\psi_{ce}\cdot\psi_{cv}+\psi_{ev}\cdot\psi_{cv}$	0.199154	0.53649	40686.3
397	$\overline{\boldsymbol{u}} \sim \psi_{ac}+\psi_{ce}+\psi_{ev}+\psi_{ac}\cdot\psi_{ev}+\psi_{ac}\cdot\psi_{cv}+\psi_{ce}\cdot\psi_{cv}+\psi_{ev}\cdot\psi_{cv}$	0.199292	0.53587	40726.2
398	$\overline{\boldsymbol{u}} \sim \psi_{ac}+\psi_{ac}\cdot\psi_{ce}+\psi_{ac}\cdot\psi_{ev}+\psi_{ce}\cdot\psi_{ev}+\psi_{ce}\cdot\psi_{cv}+\psi_{ev}\cdot\psi_{cv}$	0.117148	0.53494	40785.7
399	$\overline{\boldsymbol{u}} \sim \psi_{ce}+\psi_{ev}+\psi_{ac}\cdot\psi_{ev}+\psi_{ac}\cdot\psi_{cv}+\psi_{ce}\cdot\psi_{ev}+\psi_{ce}\cdot\psi_{cv}+\psi_{ev}\cdot\psi_{cv}$	0.18677	0.53428	40829.9
400	$\overline{\boldsymbol{u}} \sim \psi_{ac}+\psi_{ce}+\psi_{ac}\cdot\psi_{ev}+\psi_{ac}\cdot\psi_{cv}+\psi_{ce}\cdot\psi_{ev}+\psi_{ce}\cdot\psi_{cv}+\psi_{ev}\cdot\psi_{cv}$	0.192032	0.53389	40855.3
401	$\overline{\boldsymbol{u}} \sim \psi_{ce}+\psi_{ev}+\psi_{ac}\cdot\psi_{ev}+\psi_{ac}\cdot\psi_{cv}+\psi_{ce}\cdot\psi_{cv}+\psi_{ev}\cdot\psi_{cv}$	0.186077	0.53324	40896.3

402	$\overline{\boldsymbol{u}} \sim \psi_{ac}+\psi_{ce}+\psi_{ac}\cdot\psi_{cv}+\psi_{ce}\cdot\psi_{ev}+\psi_{ce}\cdot\psi_{cv}+\psi_{ev}\cdot\psi_{cv}$	0.194765	0.53254	40941.8
403	$\overline{\boldsymbol{u}} \sim \psi_{ac}+\psi_{ce}+\psi_{ev}+\psi_{ac}\cdot\psi_{cv}+\psi_{ce}\cdot\psi_{ev}+\psi_{ce}\cdot\psi_{cv}+\psi_{ev}\cdot\psi_{cv}$	0.194706	0.53253	40943.8
404	$\overline{\boldsymbol{u}} \sim \psi_{ac}+\psi_{ce}+\psi_{ac}\cdot\psi_{ev}+\psi_{ac}\cdot\psi_{cv}+\psi_{ce}\cdot\psi_{cv}+\psi_{ev}\cdot\psi_{cv}$	0.191924	0.53232	40955.9
405	$\overline{\boldsymbol{u}} \sim \psi_{ac}+\psi_{ce}+\psi_{ev}+\psi_{ac}\cdot\psi_{cv}+\psi_{ce}\cdot\psi_{cv}+\psi_{ev}\cdot\psi_{cv}$	0.194835	0.53229	40958.4
406	$\overline{\boldsymbol{u}} \sim \psi_{ac}+\psi_{ce}+\psi_{ac}\cdot\psi_{cv}+\psi_{ce}\cdot\psi_{cv}+\psi_{ev}\cdot\psi_{cv}$	0.193233	0.53212	40968.1
407	$\overline{\boldsymbol{u}} \sim \psi_{ac}+\psi_{ev}+\psi_{ac}\cdot\psi_{ce}+\psi_{ac}\cdot\psi_{ev}+\psi_{ac}\cdot\psi_{cv}+\psi_{ce}\cdot\psi_{ev}+\psi_{ev}\cdot\psi_{cv}$	0.145472	0.52981	41119.2
408	$\overline{\boldsymbol{u}} \sim \psi_{ac}+\psi_{ev}+\psi_{ac}\cdot\psi_{ce}+\psi_{ac}\cdot\psi_{cv}+\psi_{ce}\cdot\psi_{ev}+\psi_{ev}\cdot\psi_{cv}$	0.142987	0.52905	41167.3
409	$\overline{\boldsymbol{u}} \sim \psi_{ac}+\psi_{ev}+\psi_{cv}+\psi_{ac}\cdot\psi_{ce}+\psi_{ce}\cdot\psi_{cv}$	0.13004	0.5273	41279.2
410	$\overline{\boldsymbol{u}} \sim \psi_{ac}+\psi_{ev}+\psi_{cv}+\psi_{ac}\cdot\psi_{ce}+\psi_{ac}\cdot\psi_{ev}+\psi_{ce}\cdot\psi_{cv}$	0.130028	0.52728	41281
411	$\overline{\boldsymbol{u}} \sim \psi_{ac}+\psi_{ev}+\psi_{cv}+\psi_{ac}\cdot\psi_{ce}+\psi_{ce}\cdot\psi_{cv}+\psi_{ev}\cdot\psi_{cv}$	0.130046	0.52728	41281.2
412	$\overline{\boldsymbol{u}} \sim \psi_{ac}+\psi_{ev}+\psi_{cv}+\psi_{ac}\cdot\psi_{ce}+\psi_{ac}\cdot\psi_{ev}+\psi_{ce}\cdot\psi_{cv}+\psi_{ev}\cdot\psi_{cv}$	0.13003	0.52727	41283
413	$\overline{\boldsymbol{u}} \sim \psi_{ac}+\psi_{ac}\cdot\psi_{ce}+\psi_{ac}\cdot\psi_{cv}+\psi_{ce}\cdot\psi_{ev}+\psi_{ce}\cdot\psi_{cv}+\psi_{ev}\cdot\psi_{cv}$	0.136246	0.52559	41389.4
414	$\overline{\boldsymbol{u}} \sim \psi_{ev}+\psi_{ac}\cdot\psi_{ce}+\psi_{ac}\cdot\psi_{ev}+\psi_{ac}\cdot\psi_{cv}+\psi_{ce}\cdot\psi_{ev}+\psi_{ce}\cdot\psi_{cv}+\psi_{ev}\cdot\psi_{cv}$	0.142493	0.52531	41408.4
415	$\overline{\boldsymbol{u}} \sim \psi_{ce}+\psi_{cv}+\psi_{ac}\cdot\psi_{ce}+\psi_{ac}\cdot\psi_{cv}+\psi_{ce}\cdot\psi_{cv}$	0.104762	0.52399	41490.9
416	$\overline{\boldsymbol{u}} \sim \psi_{ce}+\psi_{ac}\cdot\psi_{ev}+\psi_{ac}\cdot\psi_{cv}+\psi_{ce}\cdot\psi_{ev}+\psi_{ce}\cdot\psi_{cv}+\psi_{ev}\cdot\psi_{cv}$	0.154814	0.52226	41602
417	$\overline{\boldsymbol{u}} \sim \psi_{ev}+\psi_{cv}+\psi_{ac}\cdot\psi_{ce}+\psi_{ac}\cdot\psi_{ev}+\psi_{ce}\cdot\psi_{ev}+\psi_{ce}\cdot\psi_{cv}+\psi_{ev}\cdot\psi_{cv}$	0.12892	0.52224	41604.3
418	$\overline{\boldsymbol{u}} \sim \psi_{ac}+\psi_{cv}+\psi_{ac}\cdot\psi_{ce}+\psi_{ce}\cdot\psi_{ev}+\psi_{ce}\cdot\psi_{cv}+\psi_{ev}\cdot\psi_{cv}$	0.112879	0.52149	41650.6
419	$\overline{\boldsymbol{u}} \sim \psi_{ac}+\psi_{cv}+\psi_{ac}\cdot\psi_{ce}+\psi_{ac}\cdot\psi_{ev}+\psi_{ce}\cdot\psi_{cv}+\psi_{ev}\cdot\psi_{cv}$	0.130864	0.52109	41676.3
420	$\overline{\boldsymbol{u}} \sim \psi_{ac}+\psi_{cv}+\psi_{ac}\cdot\psi_{ce}+\psi_{ac}\cdot\psi_{ev}+\psi_{ce}\cdot\psi_{cv}$	0.131007	0.521	41680.8
421	$\overline{\boldsymbol{u}} \sim \psi_{ce}+\psi_{cv}+\psi_{ac}\cdot\psi_{ce}+\psi_{ac}\cdot\psi_{cv}$	0.105833	0.52097	41681.8
422	$\overline{\boldsymbol{u}} \sim \psi_{ac}+\psi_{ce}+\psi_{ev}+\psi_{ac}\cdot\psi_{ev}+\psi_{ac}\cdot\psi_{cv}+\psi_{ce}\cdot\psi_{ev}+\psi_{ev}\cdot\psi_{cv}$	0.212538	0.51671	41953
423	$\overline{\boldsymbol{u}} \sim \psi_{ac}+\psi_{ev}+\psi_{cv}+\psi_{ac}\cdot\psi_{ce}+\psi_{ac}\cdot\psi_{ev}+\psi_{ce}\cdot\psi_{ev}+\psi_{ev}\cdot\psi_{cv}$	0.120159	0.51668	41954.9
424	$\overline{\boldsymbol{u}} \sim \psi_{ac}+\psi_{ev}+\psi_{cv}+\psi_{ac}\cdot\psi_{ce}+\psi_{ac}\cdot\psi_{ev}+\psi_{ce}\cdot\psi_{ev}$	0.121248	0.51593	42001.3
425	$\overline{\boldsymbol{u}} \sim \psi_{ac}+\psi_{ce}+\psi_{ev}+\psi_{ac}\cdot\psi_{ev}+\psi_{ac}\cdot\psi_{cv}+\psi_{ev}\cdot\psi_{cv}$	0.212941	0.51592	42001.7
426	$\overline{\boldsymbol{u}} \sim \psi_{ev}+\psi_{cv}+\psi_{ac}\cdot\psi_{ce}+\psi_{ac}\cdot\psi_{ev}+\psi_{ce}\cdot\psi_{ev}+\psi_{ce}\cdot\psi_{cv}$	0.129617	0.51577	42011
427	$\overline{\boldsymbol{u}} \sim \psi_{ac}+\psi_{ac}\cdot\psi_{ce}+\psi_{ac}\cdot\psi_{ev}+\psi_{ac}\cdot\psi_{cv}+\psi_{ce}\cdot\psi_{ev}+\psi_{ev}\cdot\psi_{cv}$	0.15145	0.51505	42056.1
428	$\overline{\boldsymbol{u}} \sim \psi_{ce}+\psi_{ev}+\psi_{ac}\cdot\psi_{ev}+\psi_{ac}\cdot\psi_{cv}+\psi_{ce}\cdot\psi_{ev}+\psi_{ev}\cdot\psi_{cv}$	0.199853	0.51476	42074.3

429	$\overline{\boldsymbol{u}} \sim \psi_{ac}+\psi_{ce}+\psi_{ac}\cdot\psi_{ev}+\psi_{ac}\cdot\psi_{cv}+\psi_{ce}\cdot\psi_{ev}+\psi_{ev}\cdot\psi_{cv}$	0.203311	0.51453	42088.8
430	$\overline{\boldsymbol{u}} \sim \psi_{ac}+\psi_{ev}+\psi_{cv}+\psi_{ac}\cdot\psi_{ce}+\psi_{ce}\cdot\psi_{ev}+\psi_{ev}\cdot\psi_{cv}$	0.1223	0.51434	42100.8
431	$\overline{\boldsymbol{u}} \sim \psi_{ac}+\psi_{ev}+\psi_{cv}+\psi_{ac}\cdot\psi_{ce}+\psi_{ce}\cdot\psi_{ev}$	0.122539	0.51425	42105.5
432	$\overline{\boldsymbol{u}} \sim \psi_{ev}+\psi_{ac}\cdot\psi_{ce}+\psi_{ac}\cdot\psi_{ev}+\psi_{ac}\cdot\psi_{cv}+\psi_{ce}\cdot\psi_{ev}+\psi_{ev}\cdot\psi_{cv}$	0.151865	0.5136	42146.9
433	$\overline{\boldsymbol{u}} \sim \psi_{ce}+\psi_{ev}+\psi_{ac}\cdot\psi_{ev}+\psi_{ac}\cdot\psi_{cv}+\psi_{ev}\cdot\psi_{cv}$	0.199278	0.51354	42149.4
434	$\overline{\boldsymbol{u}} \sim \psi_{ac}+\psi_{ce}+\psi_{ac}\cdot\psi_{ev}+\psi_{ac}\cdot\psi_{cv}+\psi_{ev}\cdot\psi_{cv}$	0.203034	0.51281	42195.1
435	$\overline{\boldsymbol{u}} \sim \psi_{ce}+\psi_{ac}\cdot\psi_{ev}+\psi_{ac}\cdot\psi_{cv}+\psi_{ce}\cdot\psi_{cv}+\psi_{ev}\cdot\psi_{cv}$	0.145806	0.51265	42205.3
436	$\overline{\boldsymbol{u}} \sim \psi_{ac}+\psi_{ac}\cdot\psi_{ce}+\psi_{ce}\cdot\psi_{ev}+\psi_{ce}\cdot\psi_{cv}+\psi_{ev}\cdot\psi_{cv}$	0.115615	0.51171	42263.7
437	$\overline{\boldsymbol{u}} \sim \psi_{ac}+\psi_{ce}+\psi_{ev}+\psi_{ac}\cdot\psi_{cv}+\psi_{ce}\cdot\psi_{ev}+\psi_{ev}\cdot\psi_{cv}$	0.208407	0.51137	42285.9
438	$\overline{\boldsymbol{u}} \sim \psi_{ac}+\psi_{ce}+\psi_{ev}+\psi_{ac}\cdot\psi_{cv}+\psi_{ev}\cdot\psi_{cv}$	0.208621	0.51107	42303.4
439	$\overline{\boldsymbol{u}} \sim \psi_{ac}+\psi_{ce}+\psi_{ac}\cdot\psi_{cv}+\psi_{ev}\cdot\psi_{cv}$	0.213145	0.51084	42316.2
440	$\overline{\boldsymbol{u}} \sim \psi_{ac}+\psi_{ce}+\psi_{ac}\cdot\psi_{cv}+\psi_{ce}\cdot\psi_{ev}+\psi_{ev}\cdot\psi_{cv}$	0.213804	0.51084	42317.7
441	$\overline{\boldsymbol{u}} \sim \psi_{ac}+\psi_{ev}+\psi_{ac}\cdot\psi_{ce}+\psi_{ac}\cdot\psi_{ev}+\psi_{ce}\cdot\psi_{ev}+\psi_{ev}\cdot\psi_{cv}$	0.121477	0.50868	42452.4
442	$\overline{\boldsymbol{u}} \sim \psi_{ce}+\psi_{cv}+\psi_{ac}\cdot\psi_{ce}+\psi_{ac}\cdot\psi_{ev}+\psi_{ce}\cdot\psi_{ev}+\psi_{ce}\cdot\psi_{cv}+\psi_{ev}\cdot\psi_{cv}$	0.0755232	0.50866	42454.7
443	$\overline{\boldsymbol{u}} \sim \psi_{ac}+\psi_{ev}+\psi_{ac}\cdot\psi_{ce}+\psi_{ce}\cdot\psi_{ev}+\psi_{ev}\cdot\psi_{cv}$	0.122219	0.50832	42473.6
444	$\overline{\boldsymbol{u}} \sim \psi_{ac}+\psi_{ev}+\psi_{ac}\cdot\psi_{ce}+\psi_{ac}\cdot\psi_{ev}+\psi_{ac}\cdot\psi_{cv}+\psi_{ce}\cdot\psi_{ev}+\psi_{ce}\cdot\psi_{cv}$	0.0887639	0.50812	42487.5
445	$\overline{\boldsymbol{u}} \sim \psi_{ac}+\psi_{ev}+\psi_{ac}\cdot\psi_{ce}+\psi_{ac}\cdot\psi_{cv}+\psi_{ce}\cdot\psi_{ev}+\psi_{ce}\cdot\psi_{cv}$	0.0897907	0.50608	42612.2
446	$\overline{\boldsymbol{u}} \sim \psi_{ce}+\psi_{ac}\cdot\psi_{ev}+\psi_{ac}\cdot\psi_{cv}+\psi_{ce}\cdot\psi_{ev}+\psi_{ev}\cdot\psi_{cv}$	0.16456	0.50445	42711.1
447	$\overline{\boldsymbol{u}} \sim \psi_{ac}+\psi_{cv}+\psi_{ac}\cdot\psi_{ce}+\psi_{ce}\cdot\psi_{cv}+\psi_{ev}\cdot\psi_{cv}$	0.123968	0.50308	42795.2
448	$\overline{\boldsymbol{u}} \sim \psi_{ac}+\psi_{cv}+\psi_{ac}\cdot\psi_{ce}+\psi_{ac}\cdot\psi_{cv}+\psi_{ce}\cdot\psi_{ev}$	0.239473	0.50265	42821.1
449	$\overline{\boldsymbol{u}} \sim \psi_{ev}+\psi_{ac}\cdot\psi_{ce}+\psi_{ac}\cdot\psi_{ev}+\psi_{ce}\cdot\psi_{ev}+\psi_{ce}\cdot\psi_{cv}+\psi_{ev}\cdot\psi_{cv}$	0.129696	0.50251	42830.6
450	$\overline{\boldsymbol{u}} \sim \psi_{cv}+\psi_{ac}\cdot\psi_{ce}+\psi_{ac}\cdot\psi_{cv}+\psi_{ce}\cdot\psi_{ev}$	0.258883	0.50217	42849.5
451	$\overline{\boldsymbol{u}} \sim \psi_{ac}+\psi_{ac}\cdot\psi_{ce}+\psi_{ac}\cdot\psi_{cv}+\psi_{ce}\cdot\psi_{ev}+\psi_{ev}\cdot\psi_{cv}$	0.162211	0.50114	42913.4
452	$\overline{\boldsymbol{u}} \sim \psi_{ac}+\psi_{ce}+\psi_{ev}+\psi_{ac}\cdot\psi_{cv}+\psi_{ce}\cdot\psi_{ev}+\psi_{ce}\cdot\psi_{cv}$	0.159203	0.49824	43089.9
453	$\overline{\boldsymbol{u}} \sim \psi_{ac}+\psi_{ce}+\psi_{ev}+\psi_{ac}\cdot\psi_{ev}+\psi_{ac}\cdot\psi_{cv}+\psi_{ce}\cdot\psi_{ev}+\psi_{ce}\cdot\psi_{cv}$	0.159162	0.49824	43090.9
454	$\overline{\boldsymbol{u}} \sim \psi_{ac}+\psi_{ce}+\psi_{ev}+\psi_{ac}\cdot\psi_{ev}+\psi_{ac}\cdot\psi_{cv}+\psi_{ce}\cdot\psi_{cv}$	0.159151	0.49813	43096.5
455	$\overline{\boldsymbol{u}} \sim \psi_{ac}+\psi_{ce}+\psi_{ev}+\psi_{ac}\cdot\psi_{cv}+\psi_{ce}\cdot\psi_{cv}$	0.159213	0.49812	43096.4

456	$\overline{\boldsymbol{u}} \sim \psi_{ac}+\psi_{ev}+\psi_{ac}\cdot\psi_{ev}+\psi_{ac}\cdot\psi_{cv}+\psi_{ce}\cdot\psi_{ev}+\psi_{ce}\cdot\psi_{cv}+\psi_{ev}\cdot\psi_{cv}$	0.189337	0.49747	43137.7
457	$\overline{\boldsymbol{u}} \sim \psi_{ac}+\psi_{ev}+\psi_{ac}\cdot\psi_{ev}+\psi_{ac}\cdot\psi_{cv}+\psi_{ce}\cdot\psi_{ev}+\psi_{ev}\cdot\psi_{cv}$	0.192209	0.49679	43177.6
458	$\overline{\boldsymbol{u}} \sim \psi_{ce}+\psi_{ev}+\psi_{ac}\cdot\psi_{ce}+\psi_{ac}\cdot\psi_{cv}+\psi_{ce}\cdot\psi_{ev}+\psi_{ce}\cdot\psi_{cv}+\psi_{ev}\cdot\psi_{cv}$	0.0921556	0.49569	43244.7
459	$\overline{\boldsymbol{u}} \sim \psi_{ce}+\psi_{ev}+\psi_{ac}\cdot\psi_{ce}+\psi_{ac}\cdot\psi_{cv}+\psi_{ce}\cdot\psi_{cv}+\psi_{ev}\cdot\psi_{cv}$	0.0923025	0.49526	43269.6
460	$\overline{\boldsymbol{u}} \sim \psi_{ce}+\psi_{ac}\cdot\psi_{ev}+\psi_{ac}\cdot\psi_{cv}+\psi_{ev}\cdot\psi_{cv}$	0.154629	0.49518	43272.7
461	$\overline{\boldsymbol{u}} \sim \psi_{ac}+\psi_{ev}+\psi_{ac}\cdot\psi_{cv}+\psi_{ce}\cdot\psi_{ev}+\psi_{ce}\cdot\psi_{cv}+\psi_{ev}\cdot\psi_{cv}$	0.186134	0.49485	43294.5
462	$\overline{\boldsymbol{u}} \sim \psi_{ac}+\psi_{cv}+\psi_{ac}\cdot\psi_{ce}+\psi_{ac}\cdot\psi_{ev}+\psi_{ce}\cdot\psi_{ev}+\psi_{ev}\cdot\psi_{cv}$	0.135217	0.49392	43350.1
463	$\overline{\boldsymbol{u}} \sim \psi_{ac}+\psi_{cv}+\psi_{ac}\cdot\psi_{ce}+\psi_{ac}\cdot\psi_{ev}+\psi_{ce}\cdot\psi_{ev}$	0.135098	0.4939	43350.6
464	$\overline{\boldsymbol{u}} \sim \psi_{ce}+\psi_{cv}+\psi_{ac}\cdot\psi_{ce}+\psi_{ac}\cdot\psi_{ev}+\psi_{ce}\cdot\psi_{ev}+\psi_{ev}\cdot\psi_{cv}$	0.0820285	0.49377	43358.9
465	$\overline{\boldsymbol{u}} \sim \psi_{ac}+\psi_{ev}+\psi_{ac}\cdot\psi_{cv}+\psi_{ce}\cdot\psi_{ev}+\psi_{ev}\cdot\psi_{cv}$	0.189672	0.49373	43360.2
466	$\overline{\boldsymbol{u}} \sim \psi_{ev}+\psi_{ac}\cdot\psi_{ev}+\psi_{ac}\cdot\psi_{cv}+\psi_{ce}\cdot\psi_{ev}+\psi_{ce}\cdot\psi_{cv}+\psi_{ev}\cdot\psi_{cv}$	0.180078	0.49341	43380.8
467	$\overline{\boldsymbol{u}} \sim \psi_{ac}+\psi_{ce}+\psi_{ac}\cdot\psi_{ev}+\psi_{ac}\cdot\psi_{cv}+\psi_{ce}\cdot\psi_{ev}+\psi_{ce}\cdot\psi_{cv}$	0.157927	0.4934	43381.2
468	$\overline{\boldsymbol{u}} \sim \psi_{ev}+\psi_{ac}\cdot\psi_{ev}+\psi_{ac}\cdot\psi_{cv}+\psi_{ce}\cdot\psi_{ev}+\psi_{ev}\cdot\psi_{cv}$	0.182243	0.49301	43403.8
469	$\overline{\boldsymbol{u}} \sim \psi_{ac}+\psi_{ce}+\psi_{ac}\cdot\psi_{ev}+\psi_{ac}\cdot\psi_{cv}+\psi_{ce}\cdot\psi_{cv}$	0.158571	0.49251	43433.6
470	$\overline{\boldsymbol{u}} \sim \psi_{ac}+\psi_{ev}+\psi_{ac}\cdot\psi_{ce}+\psi_{ac}\cdot\psi_{ev}+\psi_{ac}\cdot\psi_{cv}+\psi_{ce}\cdot\psi_{ev}$	0.111028	0.49129	43507.6
471	$\overline{\boldsymbol{u}} \sim \psi_{ac}+\psi_{ce}+\psi_{ev}+\psi_{cv}+\psi_{ce}\cdot\psi_{ev}+\psi_{ce}\cdot\psi_{cv}$	0.184237	0.4908	43536.7
472	$\overline{\boldsymbol{u}} \sim \psi_{ac}+\psi_{ce}+\psi_{ev}+\psi_{cv}+\psi_{ac}\cdot\psi_{ev}+\psi_{ce}\cdot\psi_{ev}+\psi_{ce}\cdot\psi_{cv}$	0.184177	0.4908	43537.5
473	$\overline{\boldsymbol{u}} \sim \psi_{ac}+\psi_{ce}+\psi_{ev}+\psi_{cv}+\psi_{ac}\cdot\psi_{ev}+\psi_{ce}\cdot\psi_{ev}+\psi_{ce}\cdot\psi_{cv}+\psi_{ev}\cdot\psi_{cv}$	0.184153	0.49079	43539.4
474	$\overline{\boldsymbol{u}} \sim \psi_{ac}+\psi_{ce}+\psi_{ev}+\psi_{cv}+\psi_{ce}\cdot\psi_{ev}$	0.185619	0.49078	43536.6
475	$\overline{\boldsymbol{u}} \sim \psi_{ac}+\psi_{ce}+\psi_{ev}+\psi_{cv}+\psi_{ac}\cdot\psi_{ev}+\psi_{ce}\cdot\psi_{ev}$	0.18556	0.49078	43537.5
476	$\overline{\boldsymbol{u}} \sim \psi_{ac}+\psi_{ce}+\psi_{ev}+\psi_{cv}+\psi_{ce}\cdot\psi_{ev}+\psi_{ce}\cdot\psi_{cv}+\psi_{ev}\cdot\psi_{cv}$	0.184247	0.49078	43538.6
477	$\overline{\boldsymbol{u}} \sim \psi_{ac}+\psi_{ce}+\psi_{ev}+\psi_{cv}+\psi_{ce}\cdot\psi_{ev}+\psi_{ev}\cdot\psi_{cv}$	0.185629	0.49077	43538.6
478	$\overline{\boldsymbol{u}} \sim \psi_{ac}+\psi_{ce}+\psi_{ev}+\psi_{cv}+\psi_{ac}\cdot\psi_{ev}+\psi_{ce}\cdot\psi_{ev}+\psi_{ev}\cdot\psi_{cv}$	0.185538	0.49077	43539.4
479	$\overline{\boldsymbol{u}} \sim \psi_{ac}+\psi_{ce}+\psi_{ev}+\psi_{cv}+\psi_{ac}\cdot\psi_{ev}+\psi_{ce}\cdot\psi_{cv}$	0.184156	0.49069	43543.2
480	$\overline{\boldsymbol{u}} \sim \psi_{ac}+\psi_{ce}+\psi_{ev}+\psi_{cv}+\psi_{ac}\cdot\psi_{ev}+\psi_{ce}\cdot\psi_{cv}+\psi_{ev}\cdot\psi_{cv}$	0.184105	0.49068	43544.9
481	$\overline{\boldsymbol{u}} \sim \psi_{ac}+\psi_{ce}+\psi_{ev}+\psi_{cv}+\psi_{ac}\cdot\psi_{ev}$	0.185537	0.49067	43543.2
482	$\overline{\boldsymbol{u}} \sim \psi_{ac}+\psi_{ce}+\psi_{ev}+\psi_{cv}+\psi_{ce}\cdot\psi_{cv}$	0.184248	0.49067	43543.3

483	$\overline{\boldsymbol{u}} \sim \psi_{ac}+\psi_{ce}+\psi_{ev}+\psi_{cv}+\psi_{ac}\cdot\psi_{ev}+\psi_{ev}\cdot\psi_{cv}$	0.185488	0.49066	43544.9
484	$\overline{\boldsymbol{u}} \sim \psi_{ac}+\psi_{ce}+\psi_{ev}+\psi_{cv}$	0.185627	0.49065	43543.3
485	$\overline{\boldsymbol{u}} \sim \psi_{ac}+\psi_{ce}+\psi_{ev}+\psi_{cv}+\psi_{ce}\cdot\psi_{cv}+\psi_{ev}\cdot\psi_{cv}$	0.184252	0.49065	43545.3
486	$\overline{\boldsymbol{u}} \sim \psi_{ac}+\psi_{ce}+\psi_{ev}+\psi_{cv}+\psi_{ev}\cdot\psi_{cv}$	0.185631	0.49064	43545.3
487	$\overline{\boldsymbol{u}} \sim \psi_{ac}+\psi_{ac}\cdot\psi_{ev}+\psi_{ac}\cdot\psi_{cv}+\psi_{ce}\cdot\psi_{ev}+\psi_{ce}\cdot\psi_{cv}+\psi_{ev}\cdot\psi_{cv}$	0.184097	0.49052	43553
488	$\overline{\boldsymbol{u}} \sim \psi_{ac}+\psi_{ac}\cdot\psi_{ev}+\psi_{ac}\cdot\psi_{cv}+\psi_{ce}\cdot\psi_{ev}+\psi_{ev}\cdot\psi_{cv}$	0.18512	0.49038	43560.4
489	$\overline{\boldsymbol{u}} \sim \psi_{ce}+\psi_{ac}\cdot\psi_{ce}+\psi_{ac}\cdot\psi_{cv}+\psi_{ce}\cdot\psi_{ev}+\psi_{ce}\cdot\psi_{cv}+\psi_{ev}\cdot\psi_{cv}$	0.091433	0.4899	43589.9
490	$\overline{\boldsymbol{u}} \sim \psi_{ac}+\psi_{ac}\cdot\psi_{cv}+\psi_{ce}\cdot\psi_{ev}+\psi_{ce}\cdot\psi_{cv}+\psi_{ev}\cdot\psi_{cv}$	0.182718	0.48941	43618.6
491	$\overline{\boldsymbol{u}} \sim \psi_{ac}+\psi_{ac}\cdot\psi_{cv}+\psi_{ce}\cdot\psi_{ev}+\psi_{ev}\cdot\psi_{cv}$	0.182174	0.48939	43618.3
492	$\overline{\boldsymbol{u}} \sim \psi_{ac}+\psi_{ev}+\psi_{ac}\cdot\psi_{ce}+\psi_{ac}\cdot\psi_{cv}+\psi_{ce}\cdot\psi_{ev}$	0.11231	0.48925	43627.6
493	$\overline{\boldsymbol{u}} \sim \psi_{ac}+\psi_{ac}\cdot\psi_{ce}+\psi_{ac}\cdot\psi_{ev}+\psi_{ce}\cdot\psi_{ev}+\psi_{ev}\cdot\psi_{cv}$	0.132492	0.48881	43653.7
494	$\overline{\boldsymbol{u}} \sim \psi_{ce}+\psi_{ac}\cdot\psi_{ce}+\psi_{ac}\cdot\psi_{cv}+\psi_{ce}\cdot\psi_{cv}+\psi_{ev}\cdot\psi_{cv}$	0.0907588	0.48814	43693.5
495	$\overline{\boldsymbol{u}} \sim \psi_{ac}+\psi_{ce}+\psi_{ev}+\psi_{ac}\cdot\psi_{ev}+\psi_{ce}\cdot\psi_{ev}+\psi_{ce}\cdot\psi_{cv}+\psi_{ev}\cdot\psi_{cv}$	0.173859	0.48769	43722.4
496	$\overline{\boldsymbol{u}} \sim \psi_{ac}+\psi_{ce}+\psi_{ev}+\psi_{ce}\cdot\psi_{ev}+\psi_{ce}\cdot\psi_{cv}+\psi_{ev}\cdot\psi_{cv}$	0.173104	0.48757	43728.6
497	$\overline{\boldsymbol{u}} \sim \psi_{ac}+\psi_{ce}+\psi_{ev}+\psi_{ac}\cdot\psi_{ev}+\psi_{ce}\cdot\psi_{cv}+\psi_{ev}\cdot\psi_{cv}$	0.173728	0.48747	43734.3
498	$\overline{\boldsymbol{u}} \sim \psi_{ac}+\psi_{ce}+\psi_{ev}+\psi_{ce}\cdot\psi_{cv}+\psi_{ev}\cdot\psi_{cv}$	0.173097	0.48739	43737.8
499	$\overline{\boldsymbol{u}} \sim \psi_{ac}+\psi_{ce}+\psi_{cv}+\psi_{ac}\cdot\psi_{ev}+\psi_{ce}\cdot\psi_{ev}+\psi_{ce}\cdot\psi_{cv}+\psi_{ev}\cdot\psi_{cv}$	0.179557	0.48604	43820.1
500	$\overline{\boldsymbol{u}} \sim \psi_{ac}+\psi_{ce}+\psi_{cv}+\psi_{ac}\cdot\psi_{ev}+\psi_{ce}\cdot\psi_{ev}+\psi_{ev}\cdot\psi_{cv}$	0.180835	0.48602	43820.3
501	$\overline{\boldsymbol{u}} \sim \psi_{ac}+\psi_{ce}+\psi_{cv}+\psi_{ac}\cdot\psi_{ev}+\psi_{ce}\cdot\psi_{ev}+\psi_{ce}\cdot\psi_{cv}$	0.179746	0.48599	43822.1
502	$\overline{\boldsymbol{u}} \sim \psi_{ac}+\psi_{ce}+\psi_{cv}+\psi_{ac}\cdot\psi_{ev}+\psi_{ce}\cdot\psi_{ev}$	0.181026	0.48597	43822.3
503	$\overline{\boldsymbol{u}} \sim \psi_{ac}+\psi_{ev}+\psi_{ac}\cdot\psi_{ce}+\psi_{ac}\cdot\psi_{ev}+\psi_{ce}\cdot\psi_{ev}+\psi_{ce}\cdot\psi_{cv}$	0.111412	0.48575	43836.2
504	$\overline{\boldsymbol{u}} \sim \psi_{ac}+\psi_{ev}+\psi_{ac}\cdot\psi_{ce}+\psi_{ac}\cdot\psi_{ev}+\psi_{ce}\cdot\psi_{ev}$	0.110973	0.48574	43835.5
505	$\overline{\boldsymbol{u}} \sim \psi_{ac}+\psi_{ce}+\psi_{ev}+\psi_{ac}\cdot\psi_{ev}+\psi_{ce}\cdot\psi_{ev}+\psi_{ev}\cdot\psi_{cv}$	0.180818	0.48564	43842.4
506	$\overline{\boldsymbol{u}} \sim \psi_{ac}+\psi_{ce}+\psi_{ev}+\psi_{ac}\cdot\psi_{ev}+\psi_{ev}\cdot\psi_{cv}$	0.180876	0.48535	43858.6
507	$\overline{\boldsymbol{u}} \sim \psi_{ac}+\psi_{ce}+\psi_{ev}+\psi_{ce}\cdot\psi_{ev}+\psi_{ev}\cdot\psi_{cv}$	0.180219	0.48519	43867.8
508	$\overline{\boldsymbol{u}} \sim \psi_{ac}+\psi_{ce}+\psi_{cv}+\psi_{ac}\cdot\psi_{ev}+\psi_{ce}\cdot\psi_{cv}+\psi_{ev}\cdot\psi_{cv}$	0.18034	0.48513	43872.6
509	$\overline{\boldsymbol{u}} \sim \psi_{ac}+\psi_{ce}+\psi_{cv}+\psi_{ac}\cdot\psi_{ev}+\psi_{ce}\cdot\psi_{cv}$	0.180493	0.48511	43872.9

510	$\overline{\boldsymbol{u}} \sim \psi_{ac}+\psi_{ce}+\psi_{cv}+\psi_{ac}\cdot\psi_{ev}+\psi_{ev}\cdot\psi_{cv}$	0.181595	0.48511	43872.7
511	$\overline{\boldsymbol{u}} \sim \psi_{ac}+\psi_{ce}+\psi_{cv}+\psi_{ac}\cdot\psi_{ev}$	0.18175	0.48509	43873.1
512	$\overline{\boldsymbol{u}} \sim \psi_{ac}+\psi_{ce}+\psi_{ev}+\psi_{ev}\cdot\psi_{cv}$	0.18029	0.48499	43878.6
513	$\overline{\boldsymbol{u}} \sim \psi_{ac}+\psi_{ce}+\psi_{ac}\cdot\psi_{ev}+\psi_{ce}\cdot\psi_{ev}+\psi_{ce}\cdot\psi_{cv}+\psi_{ev}\cdot\psi_{cv}$	0.170471	0.4837	43956.5
514	$\overline{\boldsymbol{u}} \sim \psi_{ac}+\psi_{ev}+\psi_{ac}\cdot\psi_{ce}+\psi_{ce}\cdot\psi_{ev}+\psi_{ce}\cdot\psi_{cv}$	0.112906	0.48364	43959.5
515	$\overline{\boldsymbol{u}} \sim \psi_{ac}+\psi_{ev}+\psi_{ac}\cdot\psi_{ce}+\psi_{ce}\cdot\psi_{ev}$	0.11243	0.48363	43959.1
516	$\overline{\boldsymbol{u}} \sim \psi_{ac}+\psi_{ce}+\psi_{ev}+\psi_{ac}\cdot\psi_{ev}+\psi_{ce}\cdot\psi_{ev}+\psi_{ce}\cdot\psi_{cv}$	0.160916	0.48356	43965.2
517	$\overline{\boldsymbol{u}} \sim \psi_{ac}+\psi_{ce}+\psi_{ev}+\psi_{ce}\cdot\psi_{ev}+\psi_{ce}\cdot\psi_{cv}$	0.160968	0.48355	43964.6
518	$\overline{\boldsymbol{u}} \sim \psi_{ac}+\psi_{ac}\cdot\psi_{ce}+\psi_{ac}\cdot\psi_{ev}+\psi_{ac}\cdot\psi_{cv}+\psi_{ce}\cdot\psi_{ev}+\psi_{ce}\cdot\psi_{cv}$	0.10309	0.48345	43971.6
519	$\overline{\boldsymbol{u}} \sim \psi_{ac}+\psi_{ce}+\psi_{ev}+\psi_{ac}\cdot\psi_{ev}+\psi_{ce}\cdot\psi_{cv}$	0.160894	0.48344	43971.1
520	$\overline{\boldsymbol{u}} \sim \psi_{ac}+\psi_{ce}+\psi_{ev}+\psi_{ce}\cdot\psi_{cv}$	0.16097	0.48341	43971.7
521	$\overline{\boldsymbol{u}} \sim \psi_{ev}+\psi_{cv}+\psi_{ac}\cdot\psi_{ce}+\psi_{ac}\cdot\psi_{ev}+\psi_{ce}\cdot\psi_{cv}+\psi_{ev}\cdot\psi_{cv}$	0.139247	0.48311	43991.2
522	$\overline{\boldsymbol{u}} \sim \psi_{ac}+\psi_{ce}+\psi_{ac}\cdot\psi_{ev}+\psi_{ce}\cdot\psi_{cv}+\psi_{ev}\cdot\psi_{cv}$	0.170941	0.48265	44017.5
523	$\overline{\boldsymbol{u}} \sim \psi_{ac}+\psi_{ce}+\psi_{ac}\cdot\psi_{ev}+\psi_{ce}\cdot\psi_{ev}+\psi_{ev}\cdot\psi_{cv}$	0.175512	0.48203	44053.7
524	$\overline{\boldsymbol{u}} \sim \psi_{ac}+\psi_{cv}+\psi_{ac}\cdot\psi_{ce}+\psi_{ac}\cdot\psi_{cv}+\psi_{ce}\cdot\psi_{cv}$	0.231635	0.48156	44081.4
525	$\overline{\boldsymbol{u}} \sim \psi_{ac}+\psi_{ce}+\psi_{ac}\cdot\psi_{ev}+\psi_{ev}\cdot\psi_{cv}$	0.17603	0.48089	44119.6
526	$\overline{\boldsymbol{u}} \sim \psi_{ac}+\psi_{ev}+\psi_{cv}+\psi_{ac}\cdot\psi_{ev}+\psi_{ce}\cdot\psi_{ev}+\psi_{ce}\cdot\psi_{cv}+\psi_{ev}\cdot\psi_{cv}$	0.184254	0.4805	44145.2
527	$\overline{\boldsymbol{u}} \sim \psi_{ac}+\psi_{ev}+\psi_{cv}+\psi_{ac}\cdot\psi_{ev}+\psi_{ce}\cdot\psi_{ev}+\psi_{ce}\cdot\psi_{cv}$	0.184472	0.48046	44146.6
528	$\overline{\boldsymbol{u}} \sim \psi_{ac}+\psi_{ev}+\psi_{cv}+\psi_{ce}\cdot\psi_{ev}+\psi_{ce}\cdot\psi_{cv}$	0.184743	0.48027	44156.7
529	$\overline{\boldsymbol{u}} \sim \psi_{ac}+\psi_{ev}+\psi_{cv}+\psi_{ce}\cdot\psi_{ev}+\psi_{ce}\cdot\psi_{cv}+\psi_{ev}\cdot\psi_{cv}$	0.18471	0.48025	44158.5
530	$\overline{\boldsymbol{u}} \sim \psi_{ac}+\psi_{ce}+\psi_{ac}\cdot\psi_{ev}+\psi_{ce}\cdot\psi_{ev}+\psi_{ce}\cdot\psi_{cv}$	0.159461	0.47887	44238.3
531	$\overline{\boldsymbol{u}} \sim \psi_{ac}+\psi_{ce}+\psi_{ac}\cdot\psi_{ev}+\psi_{ce}\cdot\psi_{cv}$	0.160184	0.47797	44289.7
532	$\overline{\boldsymbol{u}} \sim \psi_{ce}+\psi_{ev}+\psi_{ac}\cdot\psi_{ev}+\psi_{ac}\cdot\psi_{cv}+\psi_{ce}\cdot\psi_{ev}+\psi_{ce}\cdot\psi_{cv}$	0.145637	0.47793	44293.7
533	$\overline{\boldsymbol{u}} \sim \psi_{ev}+\psi_{cv}+\psi_{ac}\cdot\psi_{ce}+\psi_{ac}\cdot\psi_{ev}+\psi_{ce}\cdot\psi_{cv}$	0.139979	0.4778	44300.6
534	$\overline{\boldsymbol{u}} \sim \psi_{ac}+\psi_{ce}+\psi_{cv}+\psi_{ce}\cdot\psi_{ev}+\psi_{ce}\cdot\psi_{cv}+\psi_{ev}\cdot\psi_{cv}$	0.16274	0.4774	44324.6
535	$\overline{\boldsymbol{u}} \sim \psi_{ac}+\psi_{ce}+\psi_{cv}+\psi_{ce}\cdot\psi_{ev}+\psi_{ev}\cdot\psi_{cv}$	0.163906	0.47736	44325.7
536	$\overline{\boldsymbol{u}} \sim \psi_{ac}+\psi_{ce}+\psi_{ce}\cdot\psi_{ev}+\psi_{ev}\cdot\psi_{cv}$	0.163553	0.47735	44325.5

537	$\overline{\boldsymbol{u}} \sim \psi_{ac}+\psi_{ce}+\psi_{ce}\cdot\psi_{ev}+\psi_{ce}\cdot\psi_{cv}+\psi_{ev}\cdot\psi_{cv}$	0.163355	0.47734	44327.4
538	$\overline{\boldsymbol{u}} \sim \psi_{ce}+\psi_{ev}+\psi_{ac}\cdot\psi_{cv}+\psi_{ce}\cdot\psi_{ev}+\psi_{ce}\cdot\psi_{cv}+\psi_{ev}\cdot\psi_{cv}$	0.116415	0.47716	44338.7
539	$\overline{\boldsymbol{u}} \sim \psi_{ce}+\psi_{ev}+\psi_{ac}\cdot\psi_{ev}+\psi_{ac}\cdot\psi_{cv}+\psi_{ce}\cdot\psi_{cv}$	0.145529	0.47707	44342.6
540	$\overline{\boldsymbol{u}} \sim \psi_{ce}+\psi_{ev}+\psi_{ac}\cdot\psi_{cv}+\psi_{ce}\cdot\psi_{cv}+\psi_{ev}\cdot\psi_{cv}$	0.116492	0.47674	44361.9
541	$\overline{\boldsymbol{u}} \sim \psi_{ce}+\psi_{ev}+\psi_{ac}\cdot\psi_{ce}+\psi_{ac}\cdot\psi_{cv}+\psi_{ce}\cdot\psi_{ev}+\psi_{ev}\cdot\psi_{cv}$	0.0868669	0.47489	44470
542	$\overline{\boldsymbol{u}} \sim \psi_{ac}+\psi_{ce}+\psi_{cv}+\psi_{ac}\cdot\psi_{cv}+\psi_{ce}\cdot\psi_{cv}$	0.386441	0.47455	44488.4
543	$\overline{\boldsymbol{u}} \sim \psi_{ac}+\psi_{ce}+\psi_{cv}+\psi_{ac}\cdot\psi_{cv}$	0.389833	0.47454	44488.2
544	$\overline{\boldsymbol{u}} \sim \psi_{ce}+\psi_{ev}+\psi_{ac}\cdot\psi_{ce}+\psi_{ac}\cdot\psi_{cv}+\psi_{ev}\cdot\psi_{cv}$	0.0870926	0.47436	44499.7
545	$\overline{\boldsymbol{u}} \sim \psi_{ac}+\psi_{ev}+\psi_{cv}+\psi_{ac}\cdot\psi_{ev}+\psi_{ce}\cdot\psi_{ev}+\psi_{ev}\cdot\psi_{cv}$	0.167415	0.47404	44518.8
546	$\overline{\boldsymbol{u}} \sim \psi_{ac}+\psi_{ev}+\psi_{cv}+\psi_{ac}\cdot\psi_{ev}+\psi_{ce}\cdot\psi_{ev}$	0.167702	0.4739	44526.1
547	$\overline{\boldsymbol{u}} \sim \psi_{ce}+\psi_{ac}\cdot\psi_{cv}+\psi_{ce}\cdot\psi_{ev}+\psi_{ce}\cdot\psi_{cv}+\psi_{ev}\cdot\psi_{cv}$	0.11472	0.47373	44536.1
548	$\overline{\boldsymbol{u}} \sim \psi_{ac}+\psi_{ev}+\psi_{cv}+\psi_{ac}\cdot\psi_{ev}+\psi_{ce}\cdot\psi_{cv}$	0.186147	0.4735	44549
549	$\overline{\boldsymbol{u}} \sim \psi_{ac}+\psi_{ev}+\psi_{cv}+\psi_{ac}\cdot\psi_{ev}+\psi_{ce}\cdot\psi_{cv}+\psi_{ev}\cdot\psi_{cv}$	0.186113	0.47349	44550.7
550	$\overline{\boldsymbol{u}} \sim \psi_{ac}+\psi_{ev}+\psi_{cv}+\psi_{ce}\cdot\psi_{cv}$	0.186201	0.47348	44549.4
551	$\overline{\boldsymbol{u}} \sim \psi_{ac}+\psi_{ev}+\psi_{cv}+\psi_{ce}\cdot\psi_{cv}+\psi_{ev}\cdot\psi_{cv}$	0.186206	0.47346	44551.4
552	$\overline{\boldsymbol{u}} \sim \psi_{ac}+\psi_{ev}+\psi_{cv}+\psi_{ce}\cdot\psi_{ev}$	0.167889	0.47345	44550.8
553	$\overline{\boldsymbol{u}} \sim \psi_{ac}+\psi_{ev}+\psi_{cv}+\psi_{ce}\cdot\psi_{ev}+\psi_{ev}\cdot\psi_{cv}$	0.167825	0.47345	44552
554	$\overline{\boldsymbol{u}} \sim \psi_{ce}+\psi_{ac}\cdot\psi_{cv}+\psi_{ce}\cdot\psi_{cv}+\psi_{ev}\cdot\psi_{cv}$	0.11397	0.47303	44575.4
555	$\overline{\boldsymbol{u}} \sim \psi_{ac}+\psi_{ev}+\psi_{ac}\cdot\psi_{ce}+\psi_{ac}\cdot\psi_{ev}+\psi_{ac}\cdot\psi_{cv}+\psi_{ce}\cdot\psi_{cv}+\psi_{ev}\cdot\psi_{cv}$	0.168509	0.47291	44585.3
556	$\overline{\boldsymbol{u}} \sim \psi_{ac}+\psi_{cv}+\psi_{ac}\cdot\psi_{ev}+\psi_{ce}\cdot\psi_{ev}+\psi_{ce}\cdot\psi_{cv}$	0.183345	0.47263	44599.5
557	$\overline{\boldsymbol{u}} \sim \psi_{ac}+\psi_{cv}+\psi_{ac}\cdot\psi_{ev}+\psi_{ce}\cdot\psi_{ev}+\psi_{ce}\cdot\psi_{cv}+\psi_{ev}\cdot\psi_{cv}$	0.18327	0.47262	44600.9
558	$\overline{\boldsymbol{u}} \sim \psi_{ac}+\psi_{ac}\cdot\psi_{ce}+\psi_{ac}\cdot\psi_{ev}+\psi_{ac}\cdot\psi_{cv}+\psi_{ce}\cdot\psi_{cv}+\psi_{ev}\cdot\psi_{cv}$	0.164485	0.47063	44715
559	$\overline{\boldsymbol{u}} \sim \psi_{ac}+\psi_{ce}+\psi_{ac}\cdot\psi_{cv}+\psi_{ce}\cdot\psi_{ev}+\psi_{ce}\cdot\psi_{cv}$	0.138063	0.4694	44784.7
560	$\overline{\boldsymbol{u}} \sim \psi_{ev}+\psi_{ac}\cdot\psi_{ce}+\psi_{ac}\cdot\psi_{ev}+\psi_{ac}\cdot\psi_{cv}+\psi_{ce}\cdot\psi_{cv}+\psi_{ev}\cdot\psi_{cv}$	0.169625	0.46923	44795
561	$\overline{\boldsymbol{u}} \sim \psi_{ac}+\psi_{ev}+\psi_{ac}\cdot\psi_{ev}+\psi_{ac}\cdot\psi_{cv}+\psi_{ce}\cdot\psi_{cv}+\psi_{ev}\cdot\psi_{cv}$	0.187542	0.4687	44825.3
562	$\overline{\boldsymbol{u}} \sim \psi_{ac}+\psi_{ev}+\psi_{ac}\cdot\psi_{ev}+\psi_{ce}\cdot\psi_{ev}+\psi_{ce}\cdot\psi_{cv}+\psi_{ev}\cdot\psi_{cv}$	0.171325	0.46841	44842.2
563	$\overline{\boldsymbol{u}} \sim \psi_{ac}+\psi_{ev}+\psi_{ce}\cdot\psi_{ev}+\psi_{ce}\cdot\psi_{cv}+\psi_{ev}\cdot\psi_{cv}$	0.170684	0.46826	44849.9

564	$\overline{\boldsymbol{u}} \sim \psi_{ac}+\psi_{ac}\cdot\psi_{ce}+\psi_{ac}\cdot\psi_{ev}+\psi_{ac}\cdot\psi_{cv}+\psi_{ce}\cdot\psi_{ev}$	0.125397	0.46814	44856.6
565	$\overline{\boldsymbol{u}} \sim \psi_{ac}+\psi_{cv}+\psi_{ac}\cdot\psi_{ev}+\psi_{ce}\cdot\psi_{cv}+\psi_{ev}\cdot\psi_{cv}$	0.183371	0.46802	44863.4
566	$\overline{\boldsymbol{u}} \sim \psi_{ac}+\psi_{cv}+\psi_{ac}\cdot\psi_{ev}+\psi_{ce}\cdot\psi_{cv}$	0.183486	0.468	44863.6
567	$\overline{\boldsymbol{u}} \sim \psi_{ac}+\psi_{ev}+\psi_{ce}\cdot\psi_{ev}+\psi_{ev}\cdot\psi_{cv}$	0.165548	0.46696	44922.5
568	$\overline{\boldsymbol{u}} \sim \psi_{ac}+\psi_{ev}+\psi_{ac}\cdot\psi_{ev}+\psi_{ce}\cdot\psi_{ev}+\psi_{ev}\cdot\psi_{cv}$	0.165612	0.46696	44923.6
569	$\overline{\boldsymbol{u}} \sim \psi_{ev}+\psi_{ac}\cdot\psi_{ev}+\psi_{ac}\cdot\psi_{cv}+\psi_{ce}\cdot\psi_{cv}+\psi_{ev}\cdot\psi_{cv}$	0.182046	0.46678	44933.8
570	$\overline{\boldsymbol{u}} \sim \psi_{ac}+\psi_{ev}+\psi_{ac}\cdot\psi_{ce}+\psi_{ac}\cdot\psi_{ev}+\psi_{ce}\cdot\psi_{cv}+\psi_{ev}\cdot\psi_{cv}$	0.148447	0.46676	44935.9
571	$\overline{\boldsymbol{u}} \sim \psi_{cv}+\psi_{ac}\cdot\psi_{ce}+\psi_{ac}\cdot\psi_{cv}+\psi_{ce}\cdot\psi_{cv}$	0.164505	0.46652	44947.8
572	$\overline{\boldsymbol{u}} \sim \psi_{ac}+\psi_{ac}\cdot\psi_{ev}+\psi_{ac}\cdot\psi_{cv}+\psi_{ce}\cdot\psi_{cv}+\psi_{ev}\cdot\psi_{cv}$	0.182645	0.4663	44961.1
573	$\overline{\boldsymbol{u}} \sim \psi_{ce}+\psi_{cv}+\psi_{ac}\cdot\psi_{cv}+\psi_{ce}\cdot\psi_{cv}$	0.193054	0.46626	44962.8
574	$\overline{\boldsymbol{u}} \sim \psi_{ce}+\psi_{ac}\cdot\psi_{ce}+\psi_{ac}\cdot\psi_{ev}+\psi_{ce}\cdot\psi_{ev}+\psi_{ce}\cdot\psi_{cv}+\psi_{ev}\cdot\psi_{cv}$	0.0710264	0.46623	44966.2
575	$\overline{\boldsymbol{u}} \sim \psi_{ce}+\psi_{cv}+\psi_{ac}\cdot\psi_{cv}$	0.194267	0.46621	44964.2
576	$\overline{\boldsymbol{u}} \sim \psi_{ac}+\psi_{ev}+\psi_{ac}\cdot\psi_{ce}+\psi_{ac}\cdot\psi_{cv}+\psi_{ce}\cdot\psi_{cv}+\psi_{ev}\cdot\psi_{cv}$	0.166338	0.46616	44970.1
577	$\overline{\boldsymbol{u}} \sim \psi_{ac}+\psi_{ce}+\psi_{cv}+\psi_{ce}\cdot\psi_{cv}+\psi_{ev}\cdot\psi_{cv}$	0.156072	0.46557	45002.9
578	$\overline{\boldsymbol{u}} \sim \psi_{ac}+\psi_{ce}+\psi_{cv}+\psi_{ev}\cdot\psi_{cv}$	0.157143	0.46553	45004.2
579	$\overline{\boldsymbol{u}} \sim \psi_{ce}+\psi_{ev}+\psi_{cv}+\psi_{ac}\cdot\psi_{ev}+\psi_{ce}\cdot\psi_{ev}+\psi_{ev}\cdot\psi_{cv}$	0.166128	0.46468	45054
580	$\overline{\boldsymbol{u}} \sim \psi_{ce}+\psi_{ev}+\psi_{cv}+\psi_{ac}\cdot\psi_{ev}+\psi_{ce}\cdot\psi_{ev}+\psi_{ce}\cdot\psi_{cv}+\psi_{ev}\cdot\psi_{cv}$	0.166626	0.46468	45055.2
581	$\overline{\boldsymbol{u}} \sim \psi_{ce}+\psi_{ev}+\psi_{ac}\cdot\psi_{ev}+\psi_{ce}\cdot\psi_{ev}+\psi_{ev}\cdot\psi_{cv}$	0.166038	0.46455	45060.5
582	$\overline{\boldsymbol{u}} \sim \psi_{ce}+\psi_{ev}+\psi_{ac}\cdot\psi_{ev}+\psi_{ce}\cdot\psi_{ev}+\psi_{ce}\cdot\psi_{cv}+\psi_{ev}\cdot\psi_{cv}$	0.165551	0.46455	45061.3
583	$\overline{\boldsymbol{u}} \sim \psi_{ac}\cdot\psi_{ce}+\psi_{ac}\cdot\psi_{ev}+\psi_{ac}\cdot\psi_{cv}+\psi_{ce}\cdot\psi_{ev}+\psi_{ce}\cdot\psi_{cv}+\psi_{ev}\cdot\psi_{cv}$	0.135301	0.46444	45067.8
584	$\overline{\boldsymbol{u}} \sim \psi_{ac}+\psi_{ac}\cdot\psi_{ce}+\psi_{ac}\cdot\psi_{ev}+\psi_{ce}\cdot\psi_{cv}+\psi_{ev}\cdot\psi_{cv}$	0.145204	0.46411	45085.6
585	$\overline{\boldsymbol{u}} \sim \psi_{ac}+\psi_{cv}+\psi_{ac}\cdot\psi_{ev}+\psi_{ce}\cdot\psi_{ev}$	0.168512	0.46343	45123.2
586	$\overline{\boldsymbol{u}} \sim \psi_{ac}+\psi_{cv}+\psi_{ac}\cdot\psi_{ev}+\psi_{ce}\cdot\psi_{ev}+\psi_{ev}\cdot\psi_{cv}$	0.168527	0.46341	45125.2
587	$\overline{\boldsymbol{u}} \sim \psi_{ce}+\psi_{ev}+\psi_{cv}+\psi_{ac}\cdot\psi_{ev}+\psi_{ev}\cdot\psi_{cv}$	0.166315	0.46337	45127.6
588	$\overline{\boldsymbol{u}} \sim \psi_{ce}+\psi_{ev}+\psi_{cv}+\psi_{ac}\cdot\psi_{ev}+\psi_{ce}\cdot\psi_{cv}+\psi_{ev}\cdot\psi_{cv}$	0.166864	0.46336	45128.7
589	$\overline{\boldsymbol{u}} \sim \psi_{ce}+\psi_{ev}+\psi_{ac}\cdot\psi_{ev}+\psi_{ce}\cdot\psi_{cv}+\psi_{ev}\cdot\psi_{cv}$	0.165717	0.46321	45136.5
590	$\overline{\boldsymbol{u}} \sim \psi_{ce}+\psi_{ev}+\psi_{ac}\cdot\psi_{ev}+\psi_{ev}\cdot\psi_{cv}$	0.166252	0.4632	45135.9

591	$\overline{\boldsymbol{u}} \sim \psi_{ac}{+}\psi_{ac}{\cdot}\psi_{ce}{+}\psi_{ac}{\cdot}\psi_{cv}{+}\psi_{ce}{\cdot}\psi_{cv}{+}\psi_{ev}{\cdot}\psi_{cv}$	0.172279	0.46278	45160.6
592	$\overline{\boldsymbol{u}} \sim \psi_{ac}{+}\psi_{cv}{+}\psi_{ac}{\cdot}\psi_{cv}{+}\psi_{ce}{\cdot}\psi_{cv}$	0.309039	0.46268	45165.2
593	$\overline{\boldsymbol{u}} \sim \psi_{ce}{+}\psi_{ac}{\cdot}\psi_{ce}{+}\psi_{ac}{\cdot}\psi_{cv}{+}\psi_{ce}{\cdot}\psi_{ev}{+}\psi_{ev}{\cdot}\psi_{cv}$	0.0849304	0.4626	45170.8
594	$\overline{\boldsymbol{u}} \sim \psi_{ac}{+}\psi_{ev}{+}\psi_{ac}{\cdot}\psi_{cv}{+}\psi_{ce}{\cdot}\psi_{cv}{+}\psi_{ev}{\cdot}\psi_{cv}$	0.184387	0.46248	45177.6
595	$\overline{\boldsymbol{u}} \sim \psi_{ac}{+}\psi_{ac}{\cdot}\psi_{ce}{+}\psi_{ac}{\cdot}\psi_{ev}{+}\psi_{ce}{\cdot}\psi_{ev}{+}\psi_{ce}{\cdot}\psi_{cv}$	0.127541	0.46242	45181.2
596	$\overline{\boldsymbol{u}} \sim \psi_{ac}{+}\psi_{ac}{\cdot}\psi_{ce}{+}\psi_{ac}{\cdot}\psi_{ev}{+}\psi_{ce}{\cdot}\psi_{ev}$	0.12695	0.46236	45183.6
597	$\overline{\boldsymbol{u}} \sim \psi_{ac}{+}\psi_{ev}{+}\psi_{ac}{\cdot}\psi_{ce}{+}\psi_{ce}{\cdot}\psi_{cv}{+}\psi_{ev}{\cdot}\psi_{cv}$	0.150107	0.46225	45190.5
598	$\overline{\boldsymbol{u}} \sim \psi_{ac}{+}\psi_{ce}{+}\psi_{cv}{+}\psi_{ce}{\cdot}\psi_{ev}{+}\psi_{ce}{\cdot}\psi_{cv}$	0.14786	0.46155	45229.9
599	$\overline{\boldsymbol{u}} \sim \psi_{ac}{\cdot}\psi_{ev}{+}\psi_{ac}{\cdot}\psi_{cv}{+}\psi_{ce}{\cdot}\psi_{ev}{+}\psi_{ce}{\cdot}\psi_{cv}{+}\psi_{ev}{\cdot}\psi_{cv}$	0.145642	0.4615	45233.1
600	$\overline{\boldsymbol{u}} \sim \psi_{ac}{+}\psi_{ce}{+}\psi_{cv}{+}\psi_{ce}{\cdot}\psi_{ev}$	0.148974	0.46149	45232.2
601	$\overline{\boldsymbol{u}} \sim \psi_{ac}{+}\psi_{ac}{\cdot}\psi_{ce}{+}\psi_{ce}{\cdot}\psi_{cv}{+}\psi_{ev}{\cdot}\psi_{cv}$	0.156866	0.46144	45235.4
602	$\overline{\boldsymbol{u}} \sim \psi_{ce}{+}\psi_{ev}{+}\psi_{cv}{+}\psi_{ac}{\cdot}\psi_{ev}{+}\psi_{ce}{\cdot}\psi_{ev}{+}\psi_{ce}{\cdot}\psi_{cv}$	0.165306	0.46128	45246.3
603	$\overline{\boldsymbol{u}} \sim \psi_{ce}{+}\psi_{ev}{+}\psi_{cv}{+}\psi_{ac}{\cdot}\psi_{ev}{+}\psi_{ce}{\cdot}\psi_{ev}$	0.164668	0.46127	45245.6
604	$\overline{\boldsymbol{u}} \sim \psi_{ac}{+}\psi_{ac}{\cdot}\psi_{ev}{+}\psi_{ce}{\cdot}\psi_{ev}{+}\psi_{ce}{\cdot}\psi_{cv}{+}\psi_{ev}{\cdot}\psi_{cv}$	0.16984	0.46116	45252.1
605	$\overline{\boldsymbol{u}} \sim \psi_{ac}{+}\psi_{ce}{+}\psi_{ev}{+}\psi_{ac}{\cdot}\psi_{ev}{+}\psi_{ac}{\cdot}\psi_{cv}{+}\psi_{ce}{\cdot}\psi_{ev}$	0.168223	0.46071	45278.1
606	$\overline{\boldsymbol{u}} \sim \psi_{ac}{+}\psi_{ce}{+}\psi_{ev}{+}\psi_{ac}{\cdot}\psi_{cv}{+}\psi_{ce}{\cdot}\psi_{ev}$	0.168286	0.46069	45278.4
607	$\overline{\boldsymbol{u}} \sim \psi_{ac}{+}\psi_{ce}{+}\psi_{ev}{+}\psi_{ac}{\cdot}\psi_{ev}{+}\psi_{ac}{\cdot}\psi_{cv}$	0.16819	0.46058	45284.7
608	$\overline{\boldsymbol{u}} \sim \psi_{ac}{+}\psi_{ce}{+}\psi_{ev}{+}\psi_{ac}{\cdot}\psi_{cv}$	0.16828	0.46053	45286.3
609	$\overline{\boldsymbol{u}} \sim \psi_{ac}{+}\psi_{ce}{+}\psi_{ce}{\cdot}\psi_{cv}{+}\psi_{ev}{\cdot}\psi_{cv}$	0.160103	0.46043	45292.3
610	$\overline{\boldsymbol{u}} \sim \psi_{ce}{+}\psi_{ev}{+}\psi_{cv}{+}\psi_{ac}{\cdot}\psi_{ev}{+}\psi_{ce}{\cdot}\psi_{cv}$	0.165436	0.46014	45309.5
611	$\overline{\boldsymbol{u}} \sim \psi_{ce}{+}\psi_{ev}{+}\psi_{cv}{+}\psi_{ac}{\cdot}\psi_{ev}$	0.164758	0.46013	45309
612	$\overline{\boldsymbol{u}} \sim \psi_{ac}{+}\psi_{ac}{\cdot}\psi_{cv}{+}\psi_{ce}{\cdot}\psi_{cv}{+}\psi_{ev}{\cdot}\psi_{cv}$	0.19039	0.45999	45317
613	$\overline{\boldsymbol{u}} \sim \psi_{ac}{\cdot}\psi_{ce}{+}\psi_{ac}{\cdot}\psi_{ev}{+}\psi_{ac}{\cdot}\psi_{cv}{+}\psi_{ce}{\cdot}\psi_{ev}{+}\psi_{ev}{\cdot}\psi_{cv}$	0.140364	0.45984	45326.3
614	$\overline{\boldsymbol{u}} \sim \psi_{ac}{\cdot}\psi_{ev}{+}\psi_{ac}{\cdot}\psi_{cv}{+}\psi_{ce}{\cdot}\psi_{ev}{+}\psi_{ev}{\cdot}\psi_{cv}$	0.141808	0.4598	45327.6
615	$\overline{\boldsymbol{u}} \sim \psi_{ac}{+}\psi_{ac}{\cdot}\psi_{ev}{+}\psi_{ce}{\cdot}\psi_{ev}{+}\psi_{ev}{\cdot}\psi_{cv}$	0.164259	0.45807	45424.3
616	$\overline{\boldsymbol{u}} \sim \psi_{ce}{+}\psi_{ac}{\cdot}\psi_{ce}{+}\psi_{ac}{\cdot}\psi_{cv}{+}\psi_{ev}{\cdot}\psi_{cv}$	0.082506	0.457	45484.6
617	$\overline{\boldsymbol{u}} \sim \psi_{cv}{+}\psi_{ac}{\cdot}\psi_{cv}{+}\psi_{ce}{\cdot}\psi_{cv}$	0.201555	0.45675	45497.3

618	$\overline{\boldsymbol{u}} \sim \psi_{ac}+\psi_{ce}+\psi_{ac}\cdot\psi_{ev}+\psi_{ac}\cdot\psi_{cv}+\psi_{ce}\cdot\psi_{ev}$	0.166151	0.45624	45528.1
619	$\overline{\boldsymbol{u}} \sim \psi_{ac}+\psi_{ce}+\psi_{ac}\cdot\psi_{ev}+\psi_{ac}\cdot\psi_{cv}$	0.166843	0.45532	45577.9
620	$\overline{\boldsymbol{u}} \sim \psi_{ac}+\psi_{ce}+\psi_{ce}\cdot\psi_{ev}+\psi_{ce}\cdot\psi_{cv}$	0.137051	0.45492	45600.6
621	$\overline{\boldsymbol{u}} \sim \psi_{ce}+\psi_{ev}+\psi_{ac}\cdot\psi_{ev}+\psi_{ce}\cdot\psi_{ev}+\psi_{ce}\cdot\psi_{cv}$	0.153725	0.45437	45631.8
622	$\overline{\boldsymbol{u}} \sim \psi_{ev}+\psi_{ac}\cdot\psi_{ce}+\psi_{ac}\cdot\psi_{ev}+\psi_{ce}\cdot\psi_{ev}+\psi_{ev}\cdot\psi_{cv}$	0.139196	0.45418	45642.7
623	$\overline{\boldsymbol{u}} \sim \psi_{ev}+\psi_{cv}+\psi_{ac}\cdot\psi_{ce}+\psi_{ac}\cdot\psi_{ev}+\psi_{ce}\cdot\psi_{ev}+\psi_{ev}\cdot\psi_{cv}$	0.139257	0.45417	45644.1
624	$\overline{\boldsymbol{u}} \sim \psi_{ac}+\psi_{ce}+\psi_{ev}+\psi_{ac}\cdot\psi_{ev}+\psi_{ce}\cdot\psi_{ev}$	0.191633	0.45383	45662.1
625	$\overline{\boldsymbol{u}} \sim \psi_{ac}+\psi_{ce}+\psi_{ev}+\psi_{ce}\cdot\psi_{ev}$	0.191739	0.4538	45662.5
626	$\overline{\boldsymbol{u}} \sim \psi_{ac}+\psi_{ce}+\psi_{ev}+\psi_{ac}\cdot\psi_{ev}$	0.191589	0.45369	45668.9
627	$\overline{\boldsymbol{u}} \sim \psi_{ac}+\psi_{ce}+\psi_{ev}$	0.191741	0.45364	45670.7
628	$\overline{\boldsymbol{u}} \sim \psi_{ce}+\psi_{ev}+\psi_{ac}\cdot\psi_{ev}+\psi_{ce}\cdot\psi_{cv}$	0.15393	0.45321	45695.3
629	$\overline{\boldsymbol{u}} \sim \psi_{ac}+\psi_{ce}+\psi_{ev}\cdot\psi_{cv}$	0.150484	0.45307	45702.4
630	$\overline{\boldsymbol{u}} \sim \psi_{ac}+\psi_{ev}+\psi_{ac}\cdot\psi_{ce}+\psi_{ac}\cdot\psi_{ev}+\psi_{ac}\cdot\psi_{cv}+\psi_{ev}\cdot\psi_{cv}$	0.200134	0.45294	45712.5
631	$\overline{\boldsymbol{u}} \sim \psi_{ev}+\psi_{ac}\cdot\psi_{ce}+\psi_{ac}\cdot\psi_{ev}+\psi_{ac}\cdot\psi_{cv}+\psi_{ev}\cdot\psi_{cv}$	0.195324	0.45233	45745.3
632	$\overline{\boldsymbol{u}} \sim \psi_{ac}\cdot\psi_{ce}+\psi_{ac}\cdot\psi_{ev}+\psi_{ac}\cdot\psi_{cv}+\psi_{ce}\cdot\psi_{cv}+\psi_{ev}\cdot\psi_{cv}$	0.150238	0.45196	45765.4
633	$\overline{\boldsymbol{u}} \sim \psi_{ev}+\psi_{ac}\cdot\psi_{ce}+\psi_{ac}\cdot\psi_{ev}+\psi_{ce}\cdot\psi_{cv}+\psi_{ev}\cdot\psi_{cv}$	0.147499	0.4519	45769
634	$\overline{\boldsymbol{u}} \sim \psi_{ac}\cdot\psi_{ev}+\psi_{ac}\cdot\psi_{cv}+\psi_{ce}\cdot\psi_{cv}+\psi_{ev}\cdot\psi_{cv}$	0.152767	0.45182	45772.6
635	$\overline{\boldsymbol{u}} \sim \psi_{ev}+\psi_{cv}+\psi_{ac}\cdot\psi_{ev}+\psi_{ce}\cdot\psi_{ev}+\psi_{ce}\cdot\psi_{cv}+\psi_{ev}\cdot\psi_{cv}$	0.16913	0.45142	45796.5
636	$\overline{\boldsymbol{u}} \sim \psi_{ac}+\psi_{ev}+\psi_{ac}\cdot\psi_{ev}+\psi_{ce}\cdot\psi_{cv}+\psi_{ev}\cdot\psi_{cv}$	0.173642	0.45122	45806.7
637	$\overline{\boldsymbol{u}} \sim \psi_{ac}+\psi_{ac}\cdot\psi_{ce}+\psi_{ac}\cdot\psi_{ev}+\psi_{ac}\cdot\psi_{cv}+\psi_{ev}\cdot\psi_{cv}$	0.194574	0.45048	45847.2
638	$\overline{\boldsymbol{u}} \sim \psi_{ev}+\psi_{cv}+\psi_{ac}\cdot\psi_{ce}+\psi_{ac}\cdot\psi_{ev}+\psi_{ce}\cdot\psi_{ev}$	0.139038	0.45043	45850
639	$\overline{\boldsymbol{u}} \sim \psi_{ac}+\psi_{ce}+\psi_{ac}\cdot\psi_{ev}+\psi_{ce}\cdot\psi_{ev}$	0.18578	0.44936	45908.4
640	$\overline{\boldsymbol{u}} \sim \psi_{ac}+\psi_{ev}+\psi_{ce}\cdot\psi_{cv}+\psi_{ev}\cdot\psi_{cv}$	0.172773	0.4493	45911.7
641	$\overline{\boldsymbol{u}} \sim \psi_{ev}+\psi_{cv}+\psi_{ac}\cdot\psi_{ev}+\psi_{ce}\cdot\psi_{ev}+\psi_{ce}\cdot\psi_{cv}$	0.167622	0.44853	45955
642	$\overline{\boldsymbol{u}} \sim \psi_{ac}+\psi_{ce}+\psi_{ac}\cdot\psi_{ev}$	0.186494	0.44845	45957.3
643	$\overline{\boldsymbol{u}} \sim \psi_{ac}+\psi_{cv}+\psi_{ce}\cdot\psi_{ev}+\psi_{ce}\cdot\psi_{cv}+\psi_{ev}\cdot\psi_{cv}$	0.163453	0.44835	45964.9
644	$\overline{\boldsymbol{u}} \sim \psi_{ac}+\psi_{cv}+\psi_{ce}\cdot\psi_{cv}+\psi_{ev}\cdot\psi_{cv}$	0.162937	0.4483	45966.5

645	$\overline{\boldsymbol{u}} \sim \psi_{ac}+\psi_{ac}\cdot\psi_{ev}+\psi_{ce}\cdot\psi_{cv}+\psi_{ev}\cdot\psi_{cv}$	0.170015	0.4479	45988.5
646	$\overline{\boldsymbol{u}} \sim \psi_{ac}+\psi_{ev}+\psi_{ac}\cdot\psi_{ev}+\psi_{ac}\cdot\psi_{cv}+\psi_{ce}\cdot\psi_{ev}+\psi_{ce}\cdot\psi_{cv}$	0.151817	0.44773	45999.7
647	$\overline{\boldsymbol{u}} \sim \psi_{ac}+\psi_{ev}+\psi_{ac}\cdot\psi_{ev}+\psi_{ce}\cdot\psi_{ev}+\psi_{ce}\cdot\psi_{cv}$	0.15183	0.44771	45999.8
648	$\overline{\boldsymbol{u}} \sim \psi_{ev}+\psi_{cv}+\psi_{ac}\cdot\psi_{ev}+\psi_{ce}\cdot\psi_{cv}+\psi_{ev}\cdot\psi_{cv}$	0.170357	0.44766	46002.5
649	$\overline{\boldsymbol{u}} \sim \psi_{ac}+\psi_{ce}\cdot\psi_{ev}+\psi_{ce}\cdot\psi_{cv}+\psi_{ev}\cdot\psi_{cv}$	0.162215	0.44734	46019.1
650	$\overline{\boldsymbol{u}} \sim \psi_{ac}+\psi_{ev}+\psi_{ac}\cdot\psi_{ce}+\psi_{ac}\cdot\psi_{cv}+\psi_{ev}\cdot\psi_{cv}$	0.197838	0.44728	46023.9
651	$\overline{\boldsymbol{u}} \sim \psi_{ce}+\psi_{ev}+\psi_{ac}\cdot\psi_{cv}+\psi_{ce}\cdot\psi_{ev}+\psi_{ev}\cdot\psi_{cv}$	0.125433	0.44705	46036.1
652	$\overline{\boldsymbol{u}} \sim \psi_{ev}+\psi_{ac}\cdot\psi_{ev}+\psi_{ce}\cdot\psi_{ev}+\psi_{ce}\cdot\psi_{cv}+\psi_{ev}\cdot\psi_{cv}$	0.165346	0.44689	46045.1
653	$\overline{\boldsymbol{u}} \sim \psi_{ac}+\psi_{ev}+\psi_{ce}\cdot\psi_{ev}+\psi_{ce}\cdot\psi_{cv}$	0.152286	0.44688	46044.5
654	$\overline{\boldsymbol{u}} \sim \psi_{ac}+\psi_{ev}+\psi_{ac}\cdot\psi_{cv}+\psi_{ce}\cdot\psi_{ev}+\psi_{ce}\cdot\psi_{cv}$	0.152285	0.44688	46045.4
655	$\overline{\boldsymbol{u}} \sim \psi_{ce}+\psi_{ev}+\psi_{ac}\cdot\psi_{cv}+\psi_{ev}\cdot\psi_{cv}$	0.125591	0.44653	46064
656	$\overline{\boldsymbol{u}} \sim \psi_{ac}+\psi_{ce}\cdot\psi_{cv}+\psi_{ev}\cdot\psi_{cv}$	0.164644	0.44641	46069.3
657	$\overline{\boldsymbol{u}} \sim \psi_{ac}+\psi_{ac}\cdot\psi_{ce}+\psi_{ac}\cdot\psi_{cv}+\psi_{ev}\cdot\psi_{cv}$	0.205493	0.44559	46115.4
658	$\overline{\boldsymbol{u}} \sim \psi_{ev}+\psi_{cv}+\psi_{ac}\cdot\psi_{ev}+\psi_{ce}\cdot\psi_{cv}$	0.168952	0.44463	46167.8
659	$\overline{\boldsymbol{u}} \sim \psi_{ac}+\psi_{ev}+\psi_{ac}\cdot\psi_{ev}+\psi_{ac}\cdot\psi_{cv}+\psi_{ce}\cdot\psi_{ev}$	0.156311	0.44229	46296.3
660	$\overline{\boldsymbol{u}} \sim \psi_{ac}\cdot\psi_{ce}+\psi_{ac}\cdot\psi_{ev}+\psi_{ac}\cdot\psi_{cv}+\psi_{ev}\cdot\psi_{cv}$	0.166103	0.44225	46297.7
661	$\overline{\boldsymbol{u}} \sim \psi_{ac}+\psi_{ev}+\psi_{ac}\cdot\psi_{cv}+\psi_{ce}\cdot\psi_{ev}$	0.156532	0.44172	46326.5
662	$\overline{\boldsymbol{u}} \sim \psi_{ev}+\psi_{cv}+\psi_{ac}\cdot\psi_{ev}+\psi_{ce}\cdot\psi_{ev}+\psi_{ev}\cdot\psi_{cv}$	0.156369	0.43975	46433.9
663	$\overline{\boldsymbol{u}} \sim \psi_{ev}+\psi_{ac}\cdot\psi_{ev}+\psi_{ce}\cdot\psi_{ev}+\psi_{ev}\cdot\psi_{cv}$	0.156414	0.43944	46449.8
664	$\overline{\boldsymbol{u}} \sim \psi_{ac}+\psi_{cv}+\psi_{ac}\cdot\psi_{ce}+\psi_{ac}\cdot\psi_{cv}$	0.293556	0.43841	46505.7
665	$\overline{\boldsymbol{u}} \sim \psi_{ce}+\psi_{ac}\cdot\psi_{cv}+\psi_{ce}\cdot\psi_{ev}+\psi_{ev}\cdot\psi_{cv}$	0.127171	0.43808	46523.2
666	$\overline{\boldsymbol{u}} \sim \psi_{ev}+\psi_{ac}\cdot\psi_{ev}+\psi_{ce}\cdot\psi_{cv}+\psi_{ev}\cdot\psi_{cv}$	0.16657	0.43744	46558
667	$\overline{\boldsymbol{u}} \sim \psi_{ev}+\psi_{cv}+\psi_{ac}\cdot\psi_{ev}+\psi_{ce}\cdot\psi_{ev}$	0.155219	0.43695	46584.5
668	$\overline{\boldsymbol{u}} \sim \psi_{cv}+\psi_{ac}\cdot\psi_{ce}+\psi_{ac}\cdot\psi_{cv}$	0.220487	0.43594	46637.6
669	$\overline{\boldsymbol{u}} \sim \psi_{ac}+\psi_{ac}\cdot\psi_{ev}+\psi_{ac}\cdot\psi_{cv}+\psi_{ce}\cdot\psi_{ev}+\psi_{ce}\cdot\psi_{cv}$	0.156536	0.4352	46679.7
670	$\overline{\boldsymbol{u}} \sim \psi_{ac}+\psi_{ac}\cdot\psi_{ev}+\psi_{ce}\cdot\psi_{ev}+\psi_{ce}\cdot\psi_{cv}$	0.156662	0.43516	46680.7
671	$\overline{\boldsymbol{u}} \sim \psi_{ac}+\psi_{ev}+\psi_{ac}\cdot\psi_{ev}+\psi_{ce}\cdot\psi_{ev}$	0.170073	0.43497	46691.1

672	$\overline{\boldsymbol{u}} \sim \psi_{ce}+\psi_{ev}+\psi_{ac}\cdot\psi_{ev}+\psi_{ac}\cdot\psi_{cv}+\psi_{ce}\cdot\psi_{ev}$	0.163998	0.43468	46707.5
673	$\overline{\boldsymbol{u}} \sim \psi_{ce}+\psi_{ac}\cdot\psi_{cv}+\psi_{ev}\cdot\psi_{cv}$	0.129804	0.43458	46710.8
674	$\overline{\boldsymbol{u}} \sim \psi_{ac}+\psi_{ev}+\psi_{ce}\cdot\psi_{ev}$	0.170336	0.43437	46722
675	$\overline{\boldsymbol{u}} \sim \psi_{ce}+\psi_{ev}+\psi_{ac}\cdot\psi_{ev}+\psi_{ac}\cdot\psi_{cv}$	0.164251	0.43359	46765
676	$\overline{\boldsymbol{u}} \sim \psi_{ce}+\psi_{ev}+\psi_{ac}\cdot\psi_{ev}+\psi_{ce}\cdot\psi_{ev}$	0.170833	0.43248	46824.4
677	$\overline{\boldsymbol{u}} \sim \psi_{ac}+\psi_{ce}+\psi_{ac}\cdot\psi_{cv}+\psi_{ce}\cdot\psi_{ev}$	0.14109	0.43222	46838.3
678	$\overline{\boldsymbol{u}} \sim \psi_{ac}+\psi_{ac}\cdot\psi_{ev}+\psi_{ac}\cdot\psi_{cv}+\psi_{ce}\cdot\psi_{ev}$	0.158616	0.43195	46852.4
679	$\overline{\boldsymbol{u}} \sim \psi_{ce}+\psi_{ev}+\psi_{ac}\cdot\psi_{ev}$	0.170858	0.43148	46876.9
680	$\overline{\boldsymbol{u}} \sim \psi_{ev}+\psi_{ac}\cdot\psi_{ce}+\psi_{ac}\cdot\psi_{cv}+\psi_{ce}\cdot\psi_{ev}+\psi_{ce}\cdot\psi_{cv}+\psi_{ev}\cdot\psi_{cv}$	0.124132	0.43057	46928
681	$\overline{\boldsymbol{u}} \sim \psi_{ac}\cdot\psi_{ce}+\psi_{ac}\cdot\psi_{cv}+\psi_{ce}\cdot\psi_{ev}+\psi_{ce}\cdot\psi_{cv}+\psi_{ev}\cdot\psi_{cv}$	0.124929	0.43034	46939.6
682	$\overline{\boldsymbol{u}} \sim \psi_{ev}+\psi_{ac}\cdot\psi_{cv}+\psi_{ce}\cdot\psi_{ev}+\psi_{ce}\cdot\psi_{cv}+\psi_{ev}\cdot\psi_{cv}$	0.120617	0.43018	46948
683	$\overline{\boldsymbol{u}} \sim \psi_{ac}\cdot\psi_{cv}+\psi_{ce}\cdot\psi_{ev}+\psi_{ce}\cdot\psi_{cv}+\psi_{ev}\cdot\psi_{cv}$	0.121153	0.42986	46964
684	$\overline{\boldsymbol{u}} \sim \psi_{ev}+\psi_{ac}\cdot\psi_{ce}+\psi_{ac}\cdot\psi_{ev}+\psi_{ac}\cdot\psi_{cv}+\psi_{ce}\cdot\psi_{ev}+\psi_{ce}\cdot\psi_{cv}$	0.133067	0.42809	47060.1
685	$\overline{\boldsymbol{u}} \sim \psi_{ev}+\psi_{ac}\cdot\psi_{ce}+\psi_{ac}\cdot\psi_{cv}+\psi_{ce}\cdot\psi_{ev}+\psi_{ev}\cdot\psi_{cv}$	0.12138	0.42763	47083.5
686	$\overline{\boldsymbol{u}} \sim \psi_{ac}\cdot\psi_{ce}+\psi_{ac}\cdot\psi_{cv}+\psi_{ce}\cdot\psi_{ev}+\psi_{ev}\cdot\psi_{cv}$	0.120938	0.42761	47083.6
687	$\overline{\boldsymbol{u}} \sim \psi_{ev}+\psi_{ac}\cdot\psi_{cv}+\psi_{ce}\cdot\psi_{ev}+\psi_{ev}\cdot\psi_{cv}$	0.123397	0.42752	47088.2
688	$\overline{\boldsymbol{u}} \sim \psi_{ev}+\psi_{ac}\cdot\psi_{ce}+\psi_{ac}\cdot\psi_{ev}+\psi_{ac}\cdot\psi_{cv}+\psi_{ce}\cdot\psi_{ev}$	0.134007	0.42748	47091.5
689	$\overline{\boldsymbol{u}} \sim \psi_{ac}\cdot\psi_{cv}+\psi_{ce}\cdot\psi_{ev}+\psi_{ev}\cdot\psi_{cv}$	0.12355	0.42739	47094.4
690	$\overline{\boldsymbol{u}} \sim \psi_{ev}+\psi_{ac}\cdot\psi_{ce}+\psi_{ac}\cdot\psi_{ev}+\psi_{ce}\cdot\psi_{ev}+\psi_{ce}\cdot\psi_{cv}$	0.133821	0.42696	47118.8
691	$\overline{\boldsymbol{u}} \sim \psi_{ev}+\psi_{ac}\cdot\psi_{ce}+\psi_{ac}\cdot\psi_{ev}+\psi_{ce}\cdot\psi_{ev}$	0.133548	0.42692	47119.9
692	$\overline{\boldsymbol{u}} \sim \psi_{ac}+\psi_{cv}+\psi_{ac}\cdot\psi_{cv}+\psi_{ce}\cdot\psi_{ev}$	0.290832	0.42558	47190.8
693	$\overline{\boldsymbol{u}} \sim \psi_{ac}+\psi_{ce}+\psi_{ce}\cdot\psi_{ev}$	0.149498	0.42499	47221.3
694	$\overline{\boldsymbol{u}} \sim \psi_{ac}+\psi_{ac}\cdot\psi_{ev}+\psi_{ce}\cdot\psi_{ev}$	0.171006	0.42471	47235.8
695	$\overline{\boldsymbol{u}} \sim \psi_{ac}+\psi_{ev}+\psi_{ac}\cdot\psi_{ev}+\psi_{ac}\cdot\psi_{cv}+\psi_{ev}\cdot\psi_{cv}$	0.193249	0.42171	47395.7
696	$\overline{\boldsymbol{u}} \sim \psi_{cv}+\psi_{ac}\cdot\psi_{cv}+\psi_{ce}\cdot\psi_{ev}$	0.187354	0.42123	47418.6
697	$\overline{\boldsymbol{u}} \sim \psi_{ac}+\psi_{ev}+\psi_{cv}+\psi_{ac}\cdot\psi_{ce}+\psi_{ac}\cdot\psi_{ev}$	0.181711	0.42097	47434.6
698	$\overline{\boldsymbol{u}} \sim \psi_{ac}+\psi_{ev}+\psi_{cv}+\psi_{ac}\cdot\psi_{ce}+\psi_{ac}\cdot\psi_{ev}+\psi_{ev}\cdot\psi_{cv}$	0.181662	0.42096	47435.8

699	$\overline{\boldsymbol{u}} \sim \psi_{ac}+\psi_{ev}+\psi_{cv}+\psi_{ac}\cdot\psi_{ce}$	0.18174	0.4209	47437.1
700	$\overline{\boldsymbol{u}} \sim \psi_{ac}+\psi_{ev}+\psi_{cv}+\psi_{ac}\cdot\psi_{ce}+\psi_{ev}\cdot\psi_{cv}$	0.181738	0.42088	47439.1
701	$\overline{\boldsymbol{u}} \sim \psi_{ac}+\psi_{cv}+\psi_{ac}\cdot\psi_{cv}$	0.324621	0.42023	47471.4
702	$\overline{\boldsymbol{u}} \sim \psi_{ac}+\psi_{cv}+\psi_{ac}\cdot\psi_{ce}+\psi_{ce}\cdot\psi_{ev}+\psi_{ev}\cdot\psi_{cv}$	0.136026	0.41972	47499.9
703	$\overline{\boldsymbol{u}} \sim \psi_{ev}+\psi_{ac}\cdot\psi_{ev}+\psi_{ac}\cdot\psi_{cv}+\psi_{ev}\cdot\psi_{cv}$	0.18209	0.41965	47502.6
704	$\overline{\boldsymbol{u}} \sim \psi_{ac}+\psi_{ac}\cdot\psi_{ev}+\psi_{ac}\cdot\psi_{cv}+\psi_{ev}\cdot\psi_{cv}$	0.186128	0.41943	47514.2
705	$\overline{\boldsymbol{u}} \sim \psi_{ac}+\psi_{cv}+\psi_{ce}\cdot\psi_{ev}+\psi_{ev}\cdot\psi_{cv}$	0.143483	0.41773	47602.8
706	$\overline{\boldsymbol{u}} \sim \psi_{ac}+\psi_{cv}+\psi_{ac}\cdot\psi_{ce}+\psi_{ac}\cdot\psi_{ev}$	0.180017	0.4159	47698.1
707	$\overline{\boldsymbol{u}} \sim \psi_{ac}+\psi_{cv}+\psi_{ac}\cdot\psi_{ce}+\psi_{ac}\cdot\psi_{ev}+\psi_{ev}\cdot\psi_{cv}$	0.179943	0.41589	47699.2
708	$\overline{\boldsymbol{u}} \sim \psi_{ac}+\psi_{ev}+\psi_{ac}\cdot\psi_{cv}+\psi_{ev}\cdot\psi_{cv}$	0.190514	0.41545	47721.1
709	$\overline{\boldsymbol{u}} \sim \psi_{cv}+\psi_{ac}\cdot\psi_{cv}$	0.188692	0.41492	47746.6
710	$\overline{\boldsymbol{u}} \sim \psi_{ev}+\psi_{ac}\cdot\psi_{ev}+\psi_{ac}\cdot\psi_{cv}+\psi_{ce}\cdot\psi_{ev}+\psi_{ce}\cdot\psi_{cv}$	0.148401	0.41428	47783
711	$\overline{\boldsymbol{u}} \sim \psi_{ac}+\psi_{ev}+\psi_{ac}\cdot\psi_{ce}+\psi_{ac}\cdot\psi_{ev}+\psi_{ev}\cdot\psi_{cv}$	0.178871	0.41414	47790.1
712	$\overline{\boldsymbol{u}} \sim \psi_{ac}+\psi_{ev}+\psi_{ac}\cdot\psi_{ce}+\psi_{ev}\cdot\psi_{cv}$	0.17867	0.41381	47806.4
713	$\overline{\boldsymbol{u}} \sim \psi_{ev}+\psi_{ac}\cdot\psi_{ev}+\psi_{ce}\cdot\psi_{ev}+\psi_{ce}\cdot\psi_{cv}$	0.149877	0.41329	47833.4
714	$\overline{\boldsymbol{u}} \sim \psi_{ac}+\psi_{ac}\cdot\psi_{cv}+\psi_{ev}\cdot\psi_{cv}$	0.206548	0.41268	47863.5
715	$\overline{\boldsymbol{u}} \sim \psi_{ac}+\psi_{cv}+\psi_{ac}\cdot\psi_{ce}+\psi_{ce}\cdot\psi_{ev}+\psi_{ce}\cdot\psi_{cv}$	0.121631	0.41155	47924.2
716	$\overline{\boldsymbol{u}} \sim \psi_{ac}+\psi_{ac}\cdot\psi_{ce}+\psi_{ac}\cdot\psi_{ev}+\psi_{ev}\cdot\psi_{cv}$	0.175981	0.41025	47990
717	$\overline{\boldsymbol{u}} \sim \psi_{ev}+\psi_{ac}\cdot\psi_{ev}+\psi_{ac}\cdot\psi_{cv}+\psi_{ce}\cdot\psi_{ev}$	0.155143	0.40909	48049.6
718	$\overline{\boldsymbol{u}} \sim \psi_{ce}+\psi_{ac}\cdot\psi_{ce}+\psi_{ac}\cdot\psi_{ev}+\psi_{ac}\cdot\psi_{cv}+\psi_{ce}\cdot\psi_{ev}+\psi_{ce}\cdot\psi_{cv}$	0.0784797	0.40802	48106.6
719	$\overline{\boldsymbol{u}} \sim \psi_{ce}+\psi_{cv}+\psi_{ac}\cdot\psi_{ce}+\psi_{ac}\cdot\psi_{ev}+\psi_{ce}\cdot\psi_{cv}+\psi_{ev}\cdot\psi_{cv}$	0.0780373	0.40745	48135.8
720	$\overline{\boldsymbol{u}} \sim \psi_{ce}+\psi_{ev}+\psi_{ac}\cdot\psi_{ce}+\psi_{ac}\cdot\psi_{cv}+\psi_{ce}\cdot\psi_{ev}+\psi_{ce}\cdot\psi_{cv}$	0.087741	0.40734	48141.2
721	$\overline{\boldsymbol{u}} \sim \psi_{ev}+\psi_{ac}\cdot\psi_{ev}+\psi_{ce}\cdot\psi_{ev}$	0.158906	0.40719	48145.9
722	$\overline{\boldsymbol{u}} \sim \psi_{ce}+\psi_{ev}+\psi_{ac}\cdot\psi_{ce}+\psi_{ac}\cdot\psi_{cv}+\psi_{ce}\cdot\psi_{cv}$	0.0877116	0.40714	48150.7
723	$\overline{\boldsymbol{u}} \sim \psi_{ev}+\psi_{ac}\cdot\psi_{ce}+\psi_{ac}\cdot\psi_{cv}+\psi_{ce}\cdot\psi_{cv}+\psi_{ev}\cdot\psi_{cv}$	0.144749	0.40615	48201.2
724	$\overline{\boldsymbol{u}} \sim \psi_{ev}+\psi_{ac}\cdot\psi_{ce}+\psi_{ac}\cdot\psi_{cv}+\psi_{ev}\cdot\psi_{cv}$	0.143863	0.40611	48202.4
725	$\overline{\boldsymbol{u}} \sim \psi_{ac}\cdot\psi_{ev}+\psi_{ac}\cdot\psi_{cv}+\psi_{ev}\cdot\psi_{cv}$	0.144968	0.40534	48240.4

726	$\overline{\boldsymbol{u}} \sim \psi_{ce}+\psi_{ev}+\psi_{ac}\cdot\psi_{cv}+\psi_{ce}\cdot\psi_{ev}+\psi_{ce}\cdot\psi_{cv}$	0.100301	0.40139	48443.4
727	$\overline{\boldsymbol{u}} \sim \psi_{ce}+\psi_{ev}+\psi_{ac}\cdot\psi_{cv}+\psi_{ce}\cdot\psi_{cv}$	0.100264	0.40117	48453.4
728	$\overline{\boldsymbol{u}} \sim \psi_{ev}+\psi_{cv}+\psi_{ac}\cdot\psi_{ce}+\psi_{ac}\cdot\psi_{ev}+\psi_{ev}\cdot\psi_{cv}$	0.168776	0.3977	48629.8
729	$\overline{\boldsymbol{u}} \sim \psi_{ev}+\psi_{ac}\cdot\psi_{ce}+\psi_{ac}\cdot\psi_{ev}+\psi_{ev}\cdot\psi_{cv}$	0.168854	0.39684	48672.2
730	$\overline{\boldsymbol{u}} \sim \psi_{ce}+\psi_{cv}+\psi_{ac}\cdot\psi_{ce}+\psi_{ac}\cdot\psi_{ev}+\psi_{ev}\cdot\psi_{cv}$	0.0842095	0.39645	48692.6
731	$\overline{\boldsymbol{u}} \sim \psi_{ce}+\psi_{cv}+\psi_{ac}\cdot\psi_{ev}+\psi_{ce}\cdot\psi_{ev}+\psi_{ce}\cdot\psi_{cv}+\psi_{ev}\cdot\psi_{cv}$	0.118677	0.39645	48693.7
732	$\overline{\boldsymbol{u}} \sim \psi_{ce}+\psi_{cv}+\psi_{ac}\cdot\psi_{ev}+\psi_{ce}\cdot\psi_{ev}+\psi_{ev}\cdot\psi_{cv}$	0.11733	0.39616	48707.2
733	$\overline{\boldsymbol{u}} \sim \psi_{ac}+\psi_{cv}+\psi_{ac}\cdot\psi_{ce}+\psi_{ev}\cdot\psi_{cv}$	0.16114	0.39575	48726.9
734	$\overline{\boldsymbol{u}} \sim \psi_{ac}\cdot\psi_{ce}+\psi_{ac}\cdot\psi_{cv}+\psi_{ce}\cdot\psi_{cv}+\psi_{ev}\cdot\psi_{cv}$	0.154939	0.39563	48732.7
735	$\overline{\boldsymbol{u}} \sim \psi_{ev}+\psi_{cv}+\psi_{ac}\cdot\psi_{ce}+\psi_{ac}\cdot\psi_{ev}$	0.167654	0.39523	48753.1
736	$\overline{\boldsymbol{u}} \sim \psi_{ac}+\psi_{ev}+\psi_{ac}\cdot\psi_{ce}+\psi_{ac}\cdot\psi_{ev}+\psi_{ac}\cdot\psi_{cv}+\psi_{ce}\cdot\psi_{cv}$	0.144549	0.39477	48777.8
737	$\overline{\boldsymbol{u}} \sim \psi_{ac}+\psi_{ev}+\psi_{ac}\cdot\psi_{ce}+\psi_{ac}\cdot\psi_{cv}+\psi_{ce}\cdot\psi_{cv}$	0.144577	0.39468	48781.6
738	$\overline{\boldsymbol{u}} \sim \psi_{ac}\cdot\psi_{ce}+\psi_{ac}\cdot\psi_{cv}+\psi_{ev}\cdot\psi_{cv}$	0.153035	0.39419	48804
739	$\overline{\boldsymbol{u}} \sim \psi_{ac}+\psi_{ev}+\psi_{ac}\cdot\psi_{ev}+\psi_{ac}\cdot\psi_{cv}+\psi_{ce}\cdot\psi_{cv}$	0.153628	0.39399	48816.3
740	$\overline{\boldsymbol{u}} \sim \psi_{ac}+\psi_{ev}+\psi_{ac}\cdot\psi_{cv}+\psi_{ce}\cdot\psi_{cv}$	0.153674	0.39389	48820.3
741	$\overline{\boldsymbol{u}} \sim \psi_{ev}+\psi_{ac}\cdot\psi_{cv}+\psi_{ce}\cdot\psi_{cv}+\psi_{ev}\cdot\psi_{cv}$	0.12551	0.39076	48976.3
742	$\overline{\boldsymbol{u}} \sim \psi_{ac}+\psi_{cv}+\psi_{ce}\cdot\psi_{ev}+\psi_{ce}\cdot\psi_{cv}$	0.148372	0.39031	48998.9
743	$\overline{\boldsymbol{u}} \sim \psi_{ac}+\psi_{ac}\cdot\psi_{ce}+\psi_{ac}\cdot\psi_{ev}+\psi_{ac}\cdot\psi_{cv}+\psi_{ce}\cdot\psi_{cv}$	0.145387	0.38991	49019.5
744	$\overline{\boldsymbol{u}} \sim \psi_{ac}+\psi_{ac}\cdot\psi_{ev}+\psi_{ac}\cdot\psi_{cv}+\psi_{ce}\cdot\psi_{cv}$	0.153817	0.38916	49055.8
745	$\overline{\boldsymbol{u}} \sim \psi_{ac}+\psi_{ac}\cdot\psi_{ce}+\psi_{ce}\cdot\psi_{ev}+\psi_{ev}\cdot\psi_{cv}$	0.148523	0.3887	49078.6
746	$\overline{\boldsymbol{u}} \sim \psi_{ac}+\psi_{ac}\cdot\psi_{ce}+\psi_{ev}\cdot\psi_{cv}$	0.155044	0.38677	49173.2
747	$\overline{\boldsymbol{u}} \sim \psi_{ac}+\psi_{ev}+\psi_{cv}+\psi_{ac}\cdot\psi_{ev}$	0.167803	0.38514	49254.8
748	$\overline{\boldsymbol{u}} \sim \psi_{ac}+\psi_{ev}+\psi_{cv}+\psi_{ac}\cdot\psi_{ev}+\psi_{ev}\cdot\psi_{cv}$	0.167717	0.38514	49256
749	$\overline{\boldsymbol{u}} \sim \psi_{ac}+\psi_{ev}+\psi_{cv}$	0.167864	0.38507	49257.4
750	$\overline{\boldsymbol{u}} \sim \psi_{ac}+\psi_{ev}+\psi_{cv}+\psi_{ev}\cdot\psi_{cv}$	0.167859	0.38505	49259.4
751	$\overline{\boldsymbol{u}} \sim \psi_{ce}+\psi_{ac}\cdot\psi_{ev}+\psi_{ac}\cdot\psi_{cv}+\psi_{ce}\cdot\psi_{ev}+\psi_{ce}\cdot\psi_{cv}$	0.0992712	0.38332	49345.5
752	$\overline{\boldsymbol{u}} \sim \psi_{ac}+\psi_{ev}+\psi_{ac}\cdot\psi_{ce}+\psi_{ac}\cdot\psi_{ev}+\psi_{ac}\cdot\psi_{cv}$	0.17385	0.38299	49361.6

753	$\overline{\boldsymbol{u}} \sim \psi_{ac}+\psi_{ev}+\psi_{ac}\cdot\psi_{ce}+\psi_{ac}\cdot\psi_{cv}$	0.173872	0.38288	49366.4
754	$\overline{\boldsymbol{u}} \sim \psi_{ac}+\psi_{ce}\cdot\psi_{ev}+\psi_{ev}\cdot\psi_{cv}$	0.134649	0.3827	49374.3
755	$\overline{\boldsymbol{u}} \sim \psi_{ce}+\psi_{ac}\cdot\psi_{ev}+\psi_{ce}\cdot\psi_{ev}+\psi_{ce}\cdot\psi_{cv}+\psi_{ev}\cdot\psi_{cv}$	0.119379	0.38169	49425.8
756	$\overline{\boldsymbol{u}} \sim \psi_{ce}+\psi_{ac}\cdot\psi_{ce}+\psi_{ac}\cdot\psi_{cv}+\psi_{ce}\cdot\psi_{ev}+\psi_{ce}\cdot\psi_{cv}$	0.0852588	0.38084	49467.3
757	$\overline{\boldsymbol{u}} \sim \psi_{ce}+\psi_{ac}\cdot\psi_{ce}+\psi_{ac}\cdot\psi_{ev}+\psi_{ce}\cdot\psi_{ev}+\psi_{ev}\cdot\psi_{cv}$	0.0871649	0.38027	49495.3
758	$\overline{\boldsymbol{u}} \sim \psi_{ac}+\psi_{cv}+\psi_{ac}\cdot\psi_{ev}$	0.166764	0.38013	49500.4
759	$\overline{\boldsymbol{u}} \sim \psi_{ac}+\psi_{cv}+\psi_{ac}\cdot\psi_{ev}+\psi_{ev}\cdot\psi_{cv}$	0.166662	0.38012	49501.6
760	$\overline{\boldsymbol{u}} \sim \psi_{ac}+\psi_{ac}\cdot\psi_{ce}+\psi_{ac}\cdot\psi_{ev}+\psi_{ac}\cdot\psi_{cv}$	0.172965	0.37828	49591.5
761	$\overline{\boldsymbol{u}} \sim \psi_{ac}+\psi_{ev}+\psi_{ac}\cdot\psi_{ev}+\psi_{ev}\cdot\psi_{cv}$	0.165466	0.37797	49606.8
762	$\overline{\boldsymbol{u}} \sim \psi_{ac}+\psi_{ev}+\psi_{ev}\cdot\psi_{cv}$	0.165253	0.37762	49622.8
763	$\overline{\boldsymbol{u}} \sim \psi_{ac}+\psi_{ev}+\psi_{ac}\cdot\psi_{ce}+\psi_{ac}\cdot\psi_{ev}+\psi_{ce}\cdot\psi_{cv}$	0.189395	0.37554	49726
764	$\overline{\boldsymbol{u}} \sim \psi_{ac}+\psi_{ev}+\psi_{ac}\cdot\psi_{ce}+\psi_{ce}\cdot\psi_{cv}$	0.189429	0.37541	49731.2
765	$\overline{\boldsymbol{u}} \sim \psi_{ce}+\psi_{ac}\cdot\psi_{cv}+\psi_{ce}\cdot\psi_{ev}+\psi_{ce}\cdot\psi_{cv}$	0.0965246	0.37486	49757.7
766	$\overline{\boldsymbol{u}} \sim \psi_{ac}+\psi_{ev}+\psi_{ac}\cdot\psi_{ce}+\psi_{ac}\cdot\psi_{ev}$	0.193623	0.3748	49760.7
767	$\overline{\boldsymbol{u}} \sim \psi_{ac}+\psi_{ev}+\psi_{ac}\cdot\psi_{ce}$	0.193665	0.37468	49765.9
768	$\overline{\boldsymbol{u}} \sim \psi_{ac}+\psi_{ac}\cdot\psi_{ev}+\psi_{ev}\cdot\psi_{cv}$	0.162402	0.37417	49790.2
769	$\overline{\boldsymbol{u}} \sim \psi_{ac}+\psi_{ac}\cdot\psi_{ce}+\psi_{ac}\cdot\psi_{ev}+\psi_{ce}\cdot\psi_{cv}$	0.187241	0.37087	49951.1
770	$\overline{\boldsymbol{u}} \sim \psi_{ac}+\psi_{ac}\cdot\psi_{ce}+\psi_{ac}\cdot\psi_{ev}$	0.190921	0.37012	49986
771	$\overline{\boldsymbol{u}} \sim \psi_{ce}+\psi_{ac}\cdot\psi_{ce}+\psi_{ac}\cdot\psi_{ev}+\psi_{ac}\cdot\psi_{cv}+\psi_{ce}\cdot\psi_{ev}$	0.0610479	0.36919	50032.9
772	$\overline{\boldsymbol{u}} \sim \psi_{ac}\cdot\psi_{cv}+\psi_{ce}\cdot\psi_{cv}+\psi_{ev}\cdot\psi_{cv}$	0.139384	0.36721	50126
773	$\overline{\boldsymbol{u}} \sim \psi_{ac}+\psi_{cv}+\psi_{ac}\cdot\psi_{ce}+\psi_{ce}\cdot\psi_{cv}$	0.0924152	0.36527	50219.8
774	$\overline{\boldsymbol{u}} \sim \psi_{ev}+\psi_{ac}\cdot\psi_{ce}+\psi_{ac}\cdot\psi_{ev}+\psi_{ac}\cdot\psi_{cv}+\psi_{ce}\cdot\psi_{cv}$	0.166018	0.36307	50325.8
775	$\overline{\boldsymbol{u}} \sim \psi_{ev}+\psi_{ac}\cdot\psi_{ev}+\psi_{ac}\cdot\psi_{cv}+\psi_{ce}\cdot\psi_{cv}$	0.159528	0.36093	50426.6
776	$\overline{\boldsymbol{u}} \sim \psi_{ev}+\psi_{ac}\cdot\psi_{ce}+\psi_{ac}\cdot\psi_{ev}+\psi_{ac}\cdot\psi_{cv}$	0.170658	0.36078	50433.9
777	$\overline{\boldsymbol{u}} \sim \psi_{ac}+\psi_{cv}+\psi_{ev}\cdot\psi_{cv}$	0.145874	0.36023	50458.8
778	$\overline{\boldsymbol{u}} \sim \psi_{ce}+\psi_{ac}\cdot\psi_{ev}+\psi_{ce}\cdot\psi_{ev}+\psi_{ev}\cdot\psi_{cv}$	0.106982	0.35919	50509.1
779	$\overline{\boldsymbol{u}} \sim \psi_{ev}+\psi_{ac}\cdot\psi_{ce}+\psi_{ac}\cdot\psi_{ev}+\psi_{ce}\cdot\psi_{cv}$	0.176706	0.358	50565.4

780	$\overline{\boldsymbol{u}} \sim \psi_{ev}+\psi_{ac}\cdot\psi_{ce}+\psi_{ac}\cdot\psi_{ev}$	0.179324	0.35739	50593
781	$\overline{\boldsymbol{u}} \sim \psi_{ac}+\psi_{ev}+\psi_{ac}\cdot\psi_{ev}+\psi_{ce}\cdot\psi_{cv}$	0.219957	0.35394	50756.8
782	$\overline{\boldsymbol{u}} \sim \psi_{ac}+\psi_{ev}+\psi_{ce}\cdot\psi_{cv}$	0.220154	0.35381	50761.8
783	$\overline{\boldsymbol{u}} \sim \psi_{ce}+\psi_{cv}+\psi_{ac}\cdot\psi_{ce}+\psi_{ac}\cdot\psi_{ev}+\psi_{ce}\cdot\psi_{ev}+\psi_{ce}\cdot\psi_{cv}$	0.0824632	0.35272	50815.9
784	$\overline{\boldsymbol{u}} \sim \psi_{ac}+\psi_{ev}\cdot\psi_{cv}$	0.13725	0.3522	50836.1
785	$\overline{\boldsymbol{u}} \sim \psi_{ev}+\psi_{cv}+\psi_{ac}\cdot\psi_{ev}+\psi_{ev}\cdot\psi_{cv}$	0.154472	0.35049	50918.3
786	$\overline{\boldsymbol{u}} \sim \psi_{ev}+\psi_{ac}\cdot\psi_{ev}+\psi_{ev}\cdot\psi_{cv}$	0.154545	0.35017	50932
787	$\overline{\boldsymbol{u}} \sim \psi_{ce}+\psi_{cv}+\psi_{ac}\cdot\psi_{ev}+\psi_{ce}\cdot\psi_{cv}+\psi_{ev}\cdot\psi_{cv}$	0.116007	0.34961	50960.4
788	$\overline{\boldsymbol{u}} \sim \psi_{cv}+\psi_{ac}\cdot\psi_{ce}+\psi_{ac}\cdot\psi_{ev}+\psi_{ce}\cdot\psi_{ev}+\psi_{ce}\cdot\psi_{cv}+\psi_{ev}\cdot\psi_{cv}$	0.117353	0.34934	50974
789	$\overline{\boldsymbol{u}} \sim \psi_{ac}+\psi_{ac}\cdot\psi_{ev}+\psi_{ce}\cdot\psi_{cv}$	0.20884	0.3492	50977.1
790	$\overline{\boldsymbol{u}} \sim \psi_{ce}+\psi_{cv}+\psi_{ac}\cdot\psi_{ev}+\psi_{ev}\cdot\psi_{cv}$	0.114017	0.3487	51001.5
791	$\overline{\boldsymbol{u}} \sim \psi_{cv}+\psi_{ac}\cdot\psi_{ev}+\psi_{ce}\cdot\psi_{ev}+\psi_{ce}\cdot\psi_{cv}+\psi_{ev}\cdot\psi_{cv}$	0.125066	0.34693	51085.2
792	$\overline{\boldsymbol{u}} \sim \psi_{ac}\cdot\psi_{ce}+\psi_{ac}\cdot\psi_{ev}+\psi_{ce}\cdot\psi_{ev}+\psi_{ce}\cdot\psi_{cv}+\psi_{ev}\cdot\psi_{cv}$	0.116437	0.34686	51088
793	$\overline{\boldsymbol{u}} \sim \psi_{ev}+\psi_{cv}+\psi_{ac}\cdot\psi_{ev}$	0.153468	0.34659	51099
794	$\overline{\boldsymbol{u}} \sim \psi_{ce}+\psi_{cv}+\psi_{ac}\cdot\psi_{ce}+\psi_{ac}\cdot\psi_{ev}+\psi_{ce}\cdot\psi_{ev}$	0.0876742	0.34487	51180.7
795	$\overline{\boldsymbol{u}} \sim \psi_{ac}+\psi_{ev}+\psi_{ac}\cdot\psi_{ev}+\psi_{ac}\cdot\psi_{cv}$	0.16106	0.34453	51195.1
796	$\overline{\boldsymbol{u}} \sim \psi_{ce}+\psi_{ac}\cdot\psi_{ce}+\psi_{ac}\cdot\psi_{ev}+\psi_{ce}\cdot\psi_{ev}+\psi_{ce}\cdot\psi_{cv}$	0.083531	0.34453	51196.2
797	$\overline{\boldsymbol{u}} \sim \psi_{ac}+\psi_{ev}+\psi_{ac}\cdot\psi_{cv}$	0.161112	0.34441	51199.9
798	$\overline{\boldsymbol{u}} \sim \psi_{ev}+\psi_{ac}\cdot\psi_{cv}+\psi_{ev}\cdot\psi_{cv}$	0.110753	0.34421	51209
799	$\overline{\boldsymbol{u}} \sim \psi_{ce}+\psi_{ac}\cdot\psi_{ce}+\psi_{ac}\cdot\psi_{ev}+\psi_{ce}\cdot\psi_{ev}$	0.0856966	0.34416	51212.4
800	$\overline{\boldsymbol{u}} \sim \psi_{ac}\cdot\psi_{ev}+\psi_{ce}\cdot\psi_{ev}+\psi_{ce}\cdot\psi_{cv}+\psi_{ev}\cdot\psi_{cv}$	0.124763	0.34414	51213.2
801	$\overline{\boldsymbol{u}} \sim \psi_{cv}+\psi_{ac}\cdot\psi_{ce}+\psi_{ac}\cdot\psi_{ev}+\psi_{ce}\cdot\psi_{cv}+\psi_{ev}\cdot\psi_{cv}$	0.111964	0.34377	51231.6
802	$\overline{\boldsymbol{u}} \sim \psi_{ac}+\psi_{ac}\cdot\psi_{ev}+\psi_{ac}\cdot\psi_{cv}$	0.160836	0.33988	51408.8
803	$\overline{\boldsymbol{u}} \sim \psi_{cv}+\psi_{ac}\cdot\psi_{ev}+\psi_{ce}\cdot\psi_{cv}+\psi_{ev}\cdot\psi_{cv}$	0.121643	0.33787	51502
804	$\overline{\boldsymbol{u}} \sim \psi_{ac}+\psi_{ev}+\psi_{ac}\cdot\psi_{ev}$	0.191275	0.33643	51566.8
805	$\overline{\boldsymbol{u}} \sim \psi_{ac}+\psi_{ev}$	0.191399	0.3363	51571.9
806	$\overline{\boldsymbol{u}} \sim \psi_{ce}+\psi_{ac}\cdot\psi_{ce}+\psi_{ac}\cdot\psi_{ev}+\psi_{ce}\cdot\psi_{cv}+\psi_{ev}\cdot\psi_{cv}$	0.0822287	0.33603	51587

807	$\overline{\boldsymbol{u}} \sim \psi_{ac}+\psi_{ce}+\psi_{ac}\cdot\psi_{cv}+\psi_{ce}\cdot\psi_{cv}$	0.103611	0.33549	51610.8
808	$\overline{\boldsymbol{u}} \sim \psi_{ev}+\psi_{ac}\cdot\psi_{ce}+\psi_{ac}\cdot\psi_{cv}+\psi_{ce}\cdot\psi_{ev}+\psi_{ce}\cdot\psi_{cv}$	0.128886	0.3346	51652.4
809	$\overline{\boldsymbol{u}} \sim \psi_{ce}+\psi_{ev}+\psi_{ac}\cdot\psi_{cv}+\psi_{ce}\cdot\psi_{ev}$	0.182315	0.33216	51762.5
810	$\overline{\boldsymbol{u}} \sim \psi_{ev}+\psi_{ac}\cdot\psi_{ev}+\psi_{ce}\cdot\psi_{cv}$	0.188302	0.33213	51762.7
811	$\overline{\boldsymbol{u}} \sim \psi_{ce}+\psi_{ev}+\psi_{ac}\cdot\psi_{cv}$	0.182031	0.33185	51775.5
812	$\overline{\boldsymbol{u}} \sim \psi_{ac}+\psi_{ac}\cdot\psi_{ev}$	0.186596	0.33181	51776.5
813	$\overline{\boldsymbol{u}} \sim \psi_{ac}\cdot\psi_{ce}+\psi_{ac}\cdot\psi_{ev}+\psi_{ce}\cdot\psi_{cv}+\psi_{ev}\cdot\psi_{cv}$	0.102395	0.32768	51965.2
814	$\overline{\boldsymbol{u}} \sim \psi_{ac}+\psi_{ce}+\psi_{cv}+\psi_{ce}\cdot\psi_{cv}$	0.108891	0.32719	51987.2
815	$\overline{\boldsymbol{u}} \sim \psi_{ac}+\psi_{ce}+\psi_{cv}$	0.111235	0.32705	51992.6
816	$\overline{\boldsymbol{u}} \sim \psi_{ce}+\psi_{cv}+\psi_{ac}\cdot\psi_{ev}+\psi_{ce}\cdot\psi_{ev}+\psi_{ce}\cdot\psi_{cv}$	0.112917	0.32574	52053.7
817	$\overline{\boldsymbol{u}} \sim \psi_{ce}+\psi_{cv}+\psi_{ac}\cdot\psi_{ev}+\psi_{ce}\cdot\psi_{ev}$	0.110626	0.32424	52120.1
818	$\overline{\boldsymbol{u}} \sim \psi_{ce}+\psi_{ac}\cdot\psi_{ev}+\psi_{ac}\cdot\psi_{cv}+\psi_{ce}\cdot\psi_{ev}$	0.114676	0.32197	52222
819	$\overline{\boldsymbol{u}} \sim \psi_{ev}+\psi_{ac}\cdot\psi_{cv}+\psi_{ce}\cdot\psi_{ev}+\psi_{ce}\cdot\psi_{cv}$	0.116582	0.32171	52233.7
820	$\overline{\boldsymbol{u}} \sim \psi_{ac}+\psi_{ce}+\psi_{ce}\cdot\psi_{cv}$	0.0974744	0.32122	52254.6
821	$\overline{\boldsymbol{u}} \sim \psi_{ce}+\psi_{ac}\cdot\psi_{ev}+\psi_{ce}\cdot\psi_{ev}+\psi_{ce}\cdot\psi_{cv}$	0.110244	0.32025	52299
822	$\overline{\boldsymbol{u}} \sim \psi_{ce}+\psi_{ev}+\psi_{cv}+\psi_{ce}\cdot\psi_{ev}+\psi_{ce}\cdot\psi_{cv}$	0.291928	0.31981	52319.5
823	$\overline{\boldsymbol{u}} \sim \psi_{ce}+\psi_{ev}+\psi_{cv}+\psi_{ce}\cdot\psi_{ev}+\psi_{ce}\cdot\psi_{cv}+\psi_{ev}\cdot\psi_{cv}$	0.291945	0.31978	52321.5
824	$\overline{\boldsymbol{u}} \sim \psi_{ce}+\psi_{ev}+\psi_{cv}+\psi_{ce}\cdot\psi_{cv}$	0.291436	0.31948	52333.3
825	$\overline{\boldsymbol{u}} \sim \psi_{ce}+\psi_{ev}+\psi_{cv}+\psi_{ce}\cdot\psi_{cv}+\psi_{ev}\cdot\psi_{cv}$	0.291414	0.31945	52335.3
826	$\overline{\boldsymbol{u}} \sim \psi_{ac}\cdot\psi_{cv}+\psi_{ev}\cdot\psi_{cv}$	0.113343	0.31865	52368
827	$\overline{\boldsymbol{u}} \sim \psi_{ce}+\psi_{ev}+\psi_{ce}\cdot\psi_{ev}+\psi_{ce}\cdot\psi_{cv}+\psi_{ev}\cdot\psi_{cv}$	0.290398	0.31734	52429.4
828	$\overline{\boldsymbol{u}} \sim \psi_{ce}+\psi_{ac}\cdot\psi_{ce}+\psi_{ac}\cdot\psi_{cv}+\psi_{ce}\cdot\psi_{ev}$	0.0882046	0.31702	52442.8
829	$\overline{\boldsymbol{u}} \sim \psi_{ce}+\psi_{ev}+\psi_{ce}\cdot\psi_{cv}+\psi_{ev}\cdot\psi_{cv}$	0.290139	0.31696	52445.4
830	$\overline{\boldsymbol{u}} \sim \psi_{ce}+\psi_{ac}\cdot\psi_{ev}+\psi_{ce}\cdot\psi_{ev}$	0.113755	0.31661	52459.6
831	$\overline{\boldsymbol{u}} \sim \psi_{ce}+\psi_{ev}+\psi_{cv}+\psi_{ce}\cdot\psi_{ev}$	0.296786	0.31554	52508.2
832	$\overline{\boldsymbol{u}} \sim \psi_{ce}+\psi_{ev}+\psi_{cv}+\psi_{ce}\cdot\psi_{ev}+\psi_{ev}\cdot\psi_{cv}$	0.296797	0.31552	52510.2
833	$\overline{\boldsymbol{u}} \sim \psi_{ce}+\psi_{ev}+\psi_{ce}\cdot\psi_{ev}+\psi_{ev}\cdot\psi_{cv}$	0.293213	0.31535	52516.6

834	$\overline{\boldsymbol{u}} \sim \psi_{ce}+\psi_{ev}+\psi_{cv}$	0.296277	0.3152	52522.3
835	$\overline{\boldsymbol{u}} \sim \psi_{ce}+\psi_{ev}+\psi_{cv}+\psi_{ev}\cdot\psi_{cv}$	0.296244	0.31518	52524.3
836	$\overline{\boldsymbol{u}} \sim \psi_{ce}+\psi_{ev}+\psi_{ev}\cdot\psi_{cv}$	0.292648	0.315	52531.3
837	$\overline{\boldsymbol{u}} \sim \psi_{ce}+\psi_{ev}+\psi_{ce}\cdot\psi_{ev}$	0.291669	0.31432	52561.5
838	$\overline{\boldsymbol{u}} \sim \psi_{ce}+\psi_{ev}+\psi_{ce}\cdot\psi_{ev}+\psi_{ce}\cdot\psi_{cv}$	0.291533	0.31429	52563.5
839	$\overline{\boldsymbol{u}} \sim \psi_{ce}+\psi_{ev}$	0.291174	0.31397	52575.6
840	$\overline{\boldsymbol{u}} \sim \psi_{ce}+\psi_{ev}+\psi_{ce}\cdot\psi_{cv}$	0.291033	0.31395	52577.6
841	$\overline{\boldsymbol{u}} \sim \psi_{ev}+\psi_{cv}+\psi_{ac}\cdot\psi_{ce}+\psi_{ce}\cdot\psi_{ev}+\psi_{ce}\cdot\psi_{cv}$	0.21085	0.31347	52600.7
842	$\overline{\boldsymbol{u}} \sim \psi_{ev}+\psi_{cv}+\psi_{ac}\cdot\psi_{ce}+\psi_{ce}\cdot\psi_{ev}+\psi_{ce}\cdot\psi_{cv}+\psi_{ev}\cdot\psi_{cv}$	0.210823	0.31345	52602.6
843	$\overline{\boldsymbol{u}} \sim \psi_{ev}+\psi_{cv}+\psi_{ce}\cdot\psi_{ev}+\psi_{ce}\cdot\psi_{cv}$	0.223264	0.31335	52605.1
844	$\overline{\boldsymbol{u}} \sim \psi_{ev}+\psi_{cv}+\psi_{ce}\cdot\psi_{ev}+\psi_{ce}\cdot\psi_{cv}+\psi_{ev}\cdot\psi_{cv}$	0.223219	0.31333	52606.9
845	$\overline{\boldsymbol{u}} \sim \psi_{ce}+\psi_{ac}\cdot\psi_{ev}+\psi_{ce}\cdot\psi_{cv}+\psi_{ev}\cdot\psi_{cv}$	0.121098	0.31218	52656.9
846	$\overline{\boldsymbol{u}} \sim \psi_{ev}+\psi_{cv}+\psi_{ac}\cdot\psi_{ce}+\psi_{ce}\cdot\psi_{cv}$	0.195094	0.31211	52659.9
847	$\overline{\boldsymbol{u}} \sim \psi_{ev}+\psi_{cv}+\psi_{ac}\cdot\psi_{ce}+\psi_{ce}\cdot\psi_{cv}+\psi_{ev}\cdot\psi_{cv}$	0.195081	0.31209	52661.9
848	$\overline{\boldsymbol{u}} \sim \psi_{ev}+\psi_{cv}+\psi_{ce}\cdot\psi_{cv}$	0.209781	0.31089	52712.6
849	$\overline{\boldsymbol{u}} \sim \psi_{ev}+\psi_{cv}+\psi_{ce}\cdot\psi_{cv}+\psi_{ev}\cdot\psi_{cv}$	0.209768	0.31087	52714.6
850	$\overline{\boldsymbol{u}} \sim \psi_{ac}\cdot\psi_{ev}+\psi_{ce}\cdot\psi_{cv}+\psi_{ev}\cdot\psi_{cv}$	0.12036	0.31077	52718
851	$\overline{\boldsymbol{u}} \sim \psi_{ev}+\psi_{ac}\cdot\psi_{ev}+\psi_{ac}\cdot\psi_{cv}$	0.1603	0.31048	52730.9
852	$\overline{\boldsymbol{u}} \sim \psi_{ac}+\psi_{cv}+\psi_{ce}\cdot\psi_{cv}$	0.128419	0.30929	52782.9
853	$\overline{\boldsymbol{u}} \sim \psi_{ev}+\psi_{ac}\cdot\psi_{ce}+\psi_{ac}\cdot\psi_{cv}+\psi_{ce}\cdot\psi_{ev}$	0.161938	0.30914	52790.6
854	$\overline{\boldsymbol{u}} \sim \psi_{ev}+\psi_{ac}\cdot\psi_{ev}$	0.167926	0.30815	52832.2
855	$\overline{\boldsymbol{u}} \sim \psi_{ev}+\psi_{ac}\cdot\psi_{cv}+\psi_{ce}\cdot\psi_{ev}$	0.154573	0.30748	52862.4
856	$\overline{\boldsymbol{u}} \sim \psi_{ev}+\psi_{ac}\cdot\psi_{ce}+\psi_{ce}\cdot\psi_{ev}+\psi_{ce}\cdot\psi_{cv}+\psi_{ev}\cdot\psi_{cv}$	0.191502	0.30658	52903.8
857	$\overline{\boldsymbol{u}} \sim \psi_{ce}+\psi_{cv}+\psi_{ce}\cdot\psi_{ev}+\psi_{ce}\cdot\psi_{cv}+\psi_{ev}\cdot\psi_{cv}$	0.194392	0.30632	52915.2
858	$\overline{\boldsymbol{u}} \sim \psi_{ce}+\psi_{ce}\cdot\psi_{ev}+\psi_{ce}\cdot\psi_{cv}+\psi_{ev}\cdot\psi_{cv}$	0.191486	0.30611	52923.2
859	$\overline{\boldsymbol{u}} \sim \psi_{ce}+\psi_{ac}\cdot\psi_{cv}+\psi_{ce}\cdot\psi_{ev}$	0.133302	0.30546	52950.6
860	$\overline{\boldsymbol{u}} \sim \psi_{ev}+\psi_{ce}\cdot\psi_{ev}+\psi_{ce}\cdot\psi_{cv}+\psi_{ev}\cdot\psi_{cv}$	0.213585	0.30518	52964

861	$\overline{\boldsymbol{u}} \sim \psi_{ev}{+}\psi_{ac}{\cdot}\psi_{ce}{+}\psi_{ac}{\cdot}\psi_{cv}{+}\psi_{ce}{\cdot}\psi_{cv}$	0.159298	0.30448	52994.6
862	$\overline{\boldsymbol{u}} \sim \psi_{ce}{+}\psi_{ac}{\cdot}\psi_{ev}{+}\psi_{ac}{\cdot}\psi_{cv}{+}\psi_{ce}{\cdot}\psi_{cv}$	0.0847255	0.30381	53023.8
863	$\overline{\boldsymbol{u}} \sim \psi_{ce}{+}\psi_{ac}{\cdot}\psi_{ce}{+}\psi_{ac}{\cdot}\psi_{ev}{+}\psi_{ac}{\cdot}\psi_{cv}{+}\psi_{ce}{\cdot}\psi_{cv}$	0.0846588	0.30379	53025.8
864	$\overline{\boldsymbol{u}} \sim \psi_{ev}{+}\psi_{ac}{\cdot}\psi_{ce}{+}\psi_{ce}{\cdot}\psi_{cv}{+}\psi_{ev}{\cdot}\psi_{cv}$	0.181099	0.30345	53039.6
865	$\overline{\boldsymbol{u}} \sim \psi_{cv}{+}\psi_{ac}{\cdot}\psi_{ce}{+}\psi_{ac}{\cdot}\psi_{ev}{+}\psi_{ce}{\cdot}\psi_{ev}{+}\psi_{ev}{\cdot}\psi_{cv}$	0.131982	0.30249	53082
866	$\overline{\boldsymbol{u}} \sim \psi_{cv}{+}\psi_{ac}{\cdot}\psi_{ce}{+}\psi_{ac}{\cdot}\psi_{ev}{+}\psi_{ev}{\cdot}\psi_{cv}$	0.130289	0.30225	53091.5
867	$\overline{\boldsymbol{u}} \sim \psi_{ce}{+}\psi_{cv}{+}\psi_{ac}{\cdot}\psi_{ce}{+}\psi_{ce}{\cdot}\psi_{ev}{+}\psi_{ev}{\cdot}\psi_{cv}$	0.20719	0.30209	53099.5
868	$\overline{\boldsymbol{u}} \sim \psi_{ce}{+}\psi_{cv}{+}\psi_{ce}{\cdot}\psi_{ev}{+}\psi_{ev}{\cdot}\psi_{cv}$	0.194648	0.30204	53100.7
869	$\overline{\boldsymbol{u}} \sim \psi_{ev}{+}\psi_{cv}{+}\psi_{ac}{\cdot}\psi_{ce}{+}\psi_{ce}{\cdot}\psi_{ev}$	0.17247	0.30143	53127.2
870	$\overline{\boldsymbol{u}} \sim \psi_{ev}{+}\psi_{cv}{+}\psi_{ac}{\cdot}\psi_{ce}{+}\psi_{ce}{\cdot}\psi_{ev}{+}\psi_{ev}{\cdot}\psi_{cv}$	0.172425	0.30142	53128.8
871	$\overline{\boldsymbol{u}} \sim \psi_{ev}{+}\psi_{ac}{\cdot}\psi_{ce}{+}\psi_{ce}{\cdot}\psi_{ev}{+}\psi_{ev}{\cdot}\psi_{cv}$	0.172998	0.30054	53165.7
872	$\overline{\boldsymbol{u}} \sim \psi_{ac}{+}\psi_{cv}{+}\psi_{ac}{\cdot}\psi_{ce}{+}\psi_{ce}{\cdot}\psi_{ev}$	0.156068	0.29912	53227.5
873	$\overline{\boldsymbol{u}} \sim \psi_{ac}{+}\psi_{ce}{+}\psi_{ac}{\cdot}\psi_{cv}$	0.101014	0.29822	53265.5
874	$\overline{\boldsymbol{u}} \sim \psi_{ev}{+}\psi_{ce}{\cdot}\psi_{cv}{+}\psi_{ev}{\cdot}\psi_{cv}$	0.197356	0.29646	53341.5
875	$\overline{\boldsymbol{u}} \sim \psi_{ev}{+}\psi_{ac}{\cdot}\psi_{ce}{+}\psi_{ce}{\cdot}\psi_{ev}$	0.176905	0.29645	53341.9
876	$\overline{\boldsymbol{u}} \sim \psi_{ev}{+}\psi_{ac}{\cdot}\psi_{ce}{+}\psi_{ce}{\cdot}\psi_{ev}{+}\psi_{ce}{\cdot}\psi_{cv}$	0.176317	0.29643	53343.6
877	$\overline{\boldsymbol{u}} \sim \psi_{ev}{+}\psi_{cv}{+}\psi_{ac}{\cdot}\psi_{ce}$	0.168049	0.29633	53347.1
878	$\overline{\boldsymbol{u}} \sim \psi_{ev}{+}\psi_{cv}{+}\psi_{ac}{\cdot}\psi_{ce}{+}\psi_{ev}{\cdot}\psi_{cv}$	0.168036	0.2963	53349.1
879	$\overline{\boldsymbol{u}} \sim \psi_{ev}{+}\psi_{ac}{\cdot}\psi_{ce}{+}\psi_{ev}{\cdot}\psi_{cv}$	0.168541	0.295	53404.3
880	$\overline{\boldsymbol{u}} \sim \psi_{ce}{+}\psi_{ac}{\cdot}\psi_{ce}{+}\psi_{ce}{\cdot}\psi_{ev}{+}\psi_{ev}{\cdot}\psi_{cv}$	0.169183	0.29458	53423.3
881	$\overline{\boldsymbol{u}} \sim \psi_{ce}{+}\psi_{ce}{\cdot}\psi_{ev}{+}\psi_{ev}{\cdot}\psi_{cv}$	0.178976	0.29426	53436.3
882	$\overline{\boldsymbol{u}} \sim \psi_{ce}{+}\psi_{cv}{+}\psi_{ac}{\cdot}\psi_{ce}{+}\psi_{ce}{\cdot}\psi_{cv}{+}\psi_{ev}{\cdot}\psi_{cv}$	0.206658	0.2939	53453.6
883	$\overline{\boldsymbol{u}} \sim \psi_{ce}{+}\psi_{cv}{+}\psi_{ce}{\cdot}\psi_{cv}{+}\psi_{ev}{\cdot}\psi_{cv}$	0.176735	0.29317	53484
884	$\overline{\boldsymbol{u}} \sim \psi_{ev}{+}\psi_{ac}{\cdot}\psi_{ce}{+}\psi_{ac}{\cdot}\psi_{cv}$	0.17455	0.29246	53513.5
885	$\overline{\boldsymbol{u}} \sim \psi_{ce}{+}\psi_{cv}{+}\psi_{ac}{\cdot}\psi_{ce}{+}\psi_{ce}{\cdot}\psi_{ev}{+}\psi_{ce}{\cdot}\psi_{cv}$	0.175904	0.29202	53534.2
886	$\overline{\boldsymbol{u}} \sim \psi_{ce}{+}\psi_{cv}{+}\psi_{ce}{\cdot}\psi_{ev}{+}\psi_{ce}{\cdot}\psi_{cv}$	0.163935	0.29151	53555.2
887	$\overline{\boldsymbol{u}} \sim \psi_{ev}{+}\psi_{ce}{\cdot}\psi_{ev}{+}\psi_{ce}{\cdot}\psi_{cv}$	0.204908	0.29061	53592.8

888	$\overline{\boldsymbol{u}} \sim \psi_{ac}+\psi_{ce}$	0.106244	0.29056	53593.9
889	$\overline{\boldsymbol{u}} \sim \psi_{cv}+\psi_{ac}\cdot\psi_{ce}+\psi_{ce}\cdot\psi_{ev}+\psi_{ce}\cdot\psi_{cv}+\psi_{ev}\cdot\psi_{cv}$	0.163551	0.29009	53616.8
890	$\overline{\boldsymbol{u}} \sim \psi_{ac}\cdot\psi_{ce}+\psi_{ce}\cdot\psi_{ev}+\psi_{ce}\cdot\psi_{cv}+\psi_{ev}\cdot\psi_{cv}$	0.163853	0.29003	53618.5
891	$\overline{\boldsymbol{u}} \sim \psi_{ev}+\psi_{cv}+\psi_{ce}\cdot\psi_{ev}+\psi_{ev}\cdot\psi_{cv}$	0.194069	0.28885	53668.8
892	$\overline{\boldsymbol{u}} \sim \psi_{ce}+\psi_{cv}+\psi_{ev}\cdot\psi_{cv}$	0.1767	0.28884	53668.3
893	$\overline{\boldsymbol{u}} \sim \psi_{ev}+\psi_{cv}+\psi_{ce}\cdot\psi_{ev}$	0.194237	0.28884	53668.2
894	$\overline{\boldsymbol{u}} \sim \psi_{ce}+\psi_{cv}+\psi_{ac}\cdot\psi_{ce}+\psi_{ev}\cdot\psi_{cv}$	0.179251	0.28883	53669.7
895	$\overline{\boldsymbol{u}} \sim \psi_{ev}+\psi_{ce}\cdot\psi_{ev}+\psi_{ev}\cdot\psi_{cv}$	0.19391	0.28852	53681.8
896	$\overline{\boldsymbol{u}} \sim \psi_{ce}+\psi_{ac}\cdot\psi_{ce}+\psi_{ce}\cdot\psi_{cv}+\psi_{ev}\cdot\psi_{cv}$	0.179853	0.28752	53725.3
897	$\overline{\boldsymbol{u}} \sim \psi_{ev}+\psi_{ce}\cdot\psi_{ev}$	0.192721	0.28751	53723.7
898	$\overline{\boldsymbol{u}} \sim \psi_{ce}+\psi_{cv}+\psi_{ce}\cdot\psi_{ev}$	0.163751	0.2873	53733.7
899	$\overline{\boldsymbol{u}} \sim \psi_{ce}+\psi_{cv}+\psi_{ac}\cdot\psi_{ce}+\psi_{ce}\cdot\psi_{ev}$	0.164184	0.28728	53735.7
900	$\overline{\boldsymbol{u}} \sim \psi_{ev}+\psi_{ac}\cdot\psi_{ce}+\psi_{ce}\cdot\psi_{cv}$	0.172374	0.28716	53739.6
901	$\overline{\boldsymbol{u}} \sim \psi_{ev}+\psi_{ac}\cdot\psi_{ce}$	0.174098	0.28706	53742.8
902	$\overline{\boldsymbol{u}} \sim \psi_{cv}+\psi_{ac}\cdot\psi_{ce}+\psi_{ce}\cdot\psi_{cv}+\psi_{ev}\cdot\psi_{cv}$	0.163015	0.28688	53752.8
903	$\overline{\boldsymbol{u}} \sim \psi_{cv}+\psi_{ce}\cdot\psi_{ev}+\psi_{ce}\cdot\psi_{cv}+\psi_{ev}\cdot\psi_{cv}$	0.167726	0.28687	53753.1
904	$\overline{\boldsymbol{u}} \sim \psi_{ce}+\psi_{ce}\cdot\psi_{cv}+\psi_{ev}\cdot\psi_{cv}$	0.165139	0.28673	53758.1
905	$\overline{\boldsymbol{u}} \sim \psi_{ce}\cdot\psi_{ev}+\psi_{ce}\cdot\psi_{cv}+\psi_{ev}\cdot\psi_{cv}$	0.16907	0.28652	53767.1
906	$\overline{\boldsymbol{u}} \sim \psi_{ce}+\psi_{ac}\cdot\psi_{ce}+\psi_{ce}\cdot\psi_{ev}+\psi_{ce}\cdot\psi_{cv}$	0.172244	0.28647	53770.1
907	$\overline{\boldsymbol{u}} \sim \psi_{ce}+\psi_{ac}\cdot\psi_{ce}+\psi_{ce}\cdot\psi_{ev}$	0.169505	0.28643	53770.9
908	$\overline{\boldsymbol{u}} \sim \psi_{ce}+\psi_{ce}\cdot\psi_{ev}$	0.16251	0.28618	53780.3
909	$\overline{\boldsymbol{u}} \sim \psi_{ce}+\psi_{ce}\cdot\psi_{ev}+\psi_{ce}\cdot\psi_{cv}$	0.162515	0.28616	53782.1
910	$\overline{\boldsymbol{u}} \sim \psi_{cv}+\psi_{ce}\cdot\psi_{cv}+\psi_{ev}\cdot\psi_{cv}$	0.16716	0.28546	53812.1
911	$\overline{\boldsymbol{u}} \sim \psi_{ac}\cdot\psi_{ce}+\psi_{ce}\cdot\psi_{cv}+\psi_{ev}\cdot\psi_{cv}$	0.162525	0.28533	53817.4
912	$\overline{\boldsymbol{u}} \sim \psi_{ce}\cdot\psi_{cv}+\psi_{ev}\cdot\psi_{cv}$	0.164957	0.28508	53827.1
913	$\overline{\boldsymbol{u}} \sim \psi_{cv}+\psi_{ac}\cdot\psi_{ev}+\psi_{ce}\cdot\psi_{ev}+\psi_{ev}\cdot\psi_{cv}$	0.113129	0.27207	54376.4
914	$\overline{\boldsymbol{u}} \sim \psi_{cv}+\psi_{ac}\cdot\psi_{ce}+\psi_{ce}\cdot\psi_{ev}+\psi_{ev}\cdot\psi_{cv}$	0.154108	0.27189	54383.8

915	$\overline{\boldsymbol{u}} \sim \psi_{cv}+\psi_{ac}\cdot\psi_{ce}+\psi_{ev}\cdot\psi_{cv}$	0.153107	0.27144	54401.5
916	$\overline{\boldsymbol{u}} \sim \psi_{ac}+\psi_{ac}\cdot\psi_{ce}+\psi_{ac}\cdot\psi_{cv}+\psi_{ce}\cdot\psi_{ev}+\psi_{ce}\cdot\psi_{cv}$	0.13265	0.26905	54502.9
917	$\overline{\boldsymbol{u}} \sim \psi_{ac}+\psi_{ac}\cdot\psi_{cv}+\psi_{ce}\cdot\psi_{ev}+\psi_{ce}\cdot\psi_{cv}$	0.120802	0.26729	54574.8
918	$\overline{\boldsymbol{u}} \sim \psi_{ac}\cdot\psi_{ce}+\psi_{ac}\cdot\psi_{ev}+\psi_{ce}\cdot\psi_{ev}+\psi_{ev}\cdot\psi_{cv}$	0.14067	0.26374	54721.6
919	$\overline{\boldsymbol{u}} \sim \psi_{ac}\cdot\psi_{ce}+\psi_{ce}\cdot\psi_{ev}+\psi_{ev}\cdot\psi_{cv}$	0.149501	0.25797	54957.4
920	$\overline{\boldsymbol{u}} \sim \psi_{ac}+\psi_{cv}+\psi_{ac}\cdot\psi_{ce}$	0.127698	0.25752	54975.7
921	$\overline{\boldsymbol{u}} \sim \psi_{ac}+\psi_{ac}\cdot\psi_{ce}+\psi_{ac}\cdot\psi_{cv}+\psi_{ce}\cdot\psi_{ev}$	0.153443	0.25737	54982.8
922	$\overline{\boldsymbol{u}} \sim \psi_{cv}+\psi_{ac}\cdot\psi_{ce}+\psi_{ac}\cdot\psi_{ev}+\psi_{ce}\cdot\psi_{ev}+\psi_{ce}\cdot\psi_{cv}$	0.14365	0.25466	55094.4
923	$\overline{\boldsymbol{u}} \sim \psi_{cv}+\psi_{ac}\cdot\psi_{ce}+\psi_{ce}\cdot\psi_{ev}+\psi_{ce}\cdot\psi_{cv}$	0.147793	0.25402	55119.4
924	$\overline{\boldsymbol{u}} \sim \psi_{ce}+\psi_{ac}\cdot\psi_{ce}+\psi_{ac}\cdot\psi_{cv}+\psi_{ce}\cdot\psi_{cv}$	0.0477756	0.2529	55165.1
925	$\overline{\boldsymbol{u}} \sim \psi_{ac}+\psi_{ac}\cdot\psi_{ce}+\psi_{ce}\cdot\psi_{ev}+\psi_{ce}\cdot\psi_{cv}$	0.163527	0.24894	55325.1
926	$\overline{\boldsymbol{u}} \sim \psi_{ac}+\psi_{ac}\cdot\psi_{ce}+\psi_{ce}\cdot\psi_{ev}$	0.165446	0.24775	55372.4
927	$\overline{\boldsymbol{u}} \sim \psi_{ce}+\psi_{ac}\cdot\psi_{cv}+\psi_{ce}\cdot\psi_{cv}$	0.0636652	0.24474	55493.5
928	$\overline{\boldsymbol{u}} \sim \psi_{ev}+\psi_{ac}\cdot\psi_{cv}+\psi_{ce}\cdot\psi_{cv}$	0.383295	0.24225	55593.2
929	$\overline{\boldsymbol{u}} \sim \psi_{cv}+\psi_{ac}\cdot\psi_{ev}+\psi_{ce}\cdot\psi_{ev}+\psi_{ce}\cdot\psi_{cv}$	0.130973	0.24137	55629.4
930	$\overline{\boldsymbol{u}} \sim \psi_{ev}+\psi_{ce}\cdot\psi_{cv}$	0.414758	0.24125	55632.5
931	$\overline{\boldsymbol{u}} \sim \psi_{cv}+\psi_{ce}\cdot\psi_{ev}+\psi_{ce}\cdot\psi_{cv}$	0.139488	0.23997	55684.5
932	$\overline{\boldsymbol{u}} \sim \psi_{ce}+\psi_{ac}\cdot\psi_{ce}+\psi_{ev}\cdot\psi_{cv}$	0.137574	0.23881	55730.9
933	$\overline{\boldsymbol{u}} \sim \psi_{ce}+\psi_{ac}\cdot\psi_{ce}+\psi_{ac}\cdot\psi_{ev}+\psi_{ev}\cdot\psi_{cv}$	0.135737	0.23881	55731.9
934	$\overline{\boldsymbol{u}} \sim \psi_{ac}\cdot\psi_{ce}+\psi_{ac}\cdot\psi_{ev}+\psi_{ev}\cdot\psi_{cv}$	0.138205	0.2351	55878.2
935	$\overline{\boldsymbol{u}} \sim \psi_{ac}\cdot\psi_{ce}+\psi_{ev}\cdot\psi_{cv}$	0.136621	0.23509	55877.8
936	$\overline{\boldsymbol{u}} \sim \psi_{ac}+\psi_{ac}\cdot\psi_{ce}+\psi_{ac}\cdot\psi_{cv}+\psi_{ce}\cdot\psi_{cv}$	0.0928318	0.23408	55919.8
937	$\overline{\boldsymbol{u}} \sim \psi_{ac}\cdot\psi_{ce}+\psi_{ac}\cdot\psi_{ev}+\psi_{ac}\cdot\psi_{cv}+\psi_{ce}\cdot\psi_{ev}+\psi_{ce}\cdot\psi_{cv}$	0.150138	0.23276	55972.8
938	$\overline{\boldsymbol{u}} \sim \psi_{ce}+\psi_{ac}\cdot\psi_{ev}+\psi_{ev}\cdot\psi_{cv}$	0.147118	0.2327	55973.3
939	$\overline{\boldsymbol{u}} \sim \psi_{ac}+\psi_{ac}\cdot\psi_{cv}+\psi_{ce}\cdot\psi_{cv}$	0.10445	0.23251	55980.9
940	$\overline{\boldsymbol{u}} \sim \psi_{ce}+\psi_{ev}\cdot\psi_{cv}$	0.168784	0.23234	55986.6
941	$\overline{\boldsymbol{u}} \sim \psi_{ac}\cdot\psi_{ce}+\psi_{ac}\cdot\psi_{ev}+\psi_{ac}\cdot\psi_{cv}+\psi_{ce}\cdot\psi_{cv}$	0.144785	0.23084	56047.9

942	$\overline{\boldsymbol{u}} \sim \psi_{cv}+\psi_{ce}\cdot\psi_{ev}+\psi_{ev}\cdot\psi_{cv}$	0.15375	0.22491	56279.6
943	$\overline{\boldsymbol{u}} \sim \psi_{cv}+\psi_{ac}\cdot\psi_{ce}+\psi_{ac}\cdot\psi_{ev}+\psi_{ce}\cdot\psi_{ev}$	0.152417	0.22485	56283.1
944	$\overline{\boldsymbol{u}} \sim \psi_{ac}+\psi_{cv}$	0.0959482	0.22351	56333.3
945	$\overline{\boldsymbol{u}} \sim \psi_{ac}+\psi_{cv}+\psi_{ce}\cdot\psi_{ev}$	0.0959747	0.22349	56335.3
946	$\overline{\boldsymbol{u}} \sim \psi_{ce}+\psi_{ac}\cdot\psi_{ev}+\psi_{ac}\cdot\psi_{cv}$	0.177933	0.22324	56345.1
947	$\overline{\boldsymbol{u}} \sim \psi_{cv}+\psi_{ac}\cdot\psi_{ce}+\psi_{ce}\cdot\psi_{ev}$	0.148263	0.22266	56367.7
948	$\overline{\boldsymbol{u}} \sim \psi_{ac}+\psi_{ac}\cdot\psi_{ce}+\psi_{ac}\cdot\psi_{cv}$	0.124596	0.22032	56459
949	$\overline{\boldsymbol{u}} \sim \psi_{cv}+\psi_{ac}\cdot\psi_{ev}+\psi_{ev}\cdot\psi_{cv}$	0.106093	0.21979	56479.6
950	$\overline{\boldsymbol{u}} \sim \psi_{ac}\cdot\psi_{ce}+\psi_{ac}\cdot\psi_{ev}+\psi_{ac}\cdot\psi_{cv}+\psi_{ce}\cdot\psi_{ev}$	0.166079	0.2142	56697.1
951	$\overline{\boldsymbol{u}} \sim \psi_{ac}+\psi_{ac}\cdot\psi_{ce}+\psi_{ce}\cdot\psi_{cv}$	0.136574	0.21216	56774.7
952	$\overline{\boldsymbol{u}} \sim \psi_{ac}+\psi_{ac}\cdot\psi_{ce}$	0.13645	0.21132	56806.2
953	$\overline{\boldsymbol{u}} \sim \psi_{ce}+\psi_{cv}+\psi_{ac}\cdot\psi_{ev}+\psi_{ce}\cdot\psi_{cv}$	0.813298	0.21083	56826.8
954	$\overline{\boldsymbol{u}} \sim \psi_{ac}\cdot\psi_{ce}+\psi_{ac}\cdot\psi_{ev}+\psi_{ce}\cdot\psi_{ev}+\psi_{ce}\cdot\psi_{cv}$	0.160895	0.21022	56850.4
955	$\overline{\boldsymbol{u}} \sim \psi_{ce}+\psi_{ac}\cdot\psi_{ev}+\psi_{ce}\cdot\psi_{cv}$	0.817423	0.21021	56849.7
956	$\overline{\boldsymbol{u}} \sim \psi_{ac}\cdot\psi_{ce}+\psi_{ac}\cdot\psi_{ev}+\psi_{ce}\cdot\psi_{ev}$	0.162711	0.20987	56862.8
957	$\overline{\boldsymbol{u}} \sim \psi_{ac}\cdot\psi_{ce}+\psi_{ac}\cdot\psi_{cv}+\psi_{ce}\cdot\psi_{ev}+\psi_{ce}\cdot\psi_{cv}$	0.153923	0.20628	57001.3
958	$\overline{\boldsymbol{u}} \sim \psi_{ce}+\psi_{cv}+\psi_{ac}\cdot\psi_{ev}$	0.818547	0.2059	57014.8
959	$\overline{\boldsymbol{u}} \sim \psi_{ce}+\psi_{ac}\cdot\psi_{ev}$	0.81568	0.2057	57021.4
960	$\overline{\boldsymbol{u}} \sim \psi_{ac}\cdot\psi_{ce}+\psi_{ce}\cdot\psi_{ev}+\psi_{ce}\cdot\psi_{cv}$	0.154125	0.20207	57160.7
961	$\overline{\boldsymbol{u}} \sim \psi_{ac}\cdot\psi_{ce}+\psi_{ac}\cdot\psi_{cv}+\psi_{ce}\cdot\psi_{ev}$	0.156453	0.2018	57170.9
962	$\overline{\boldsymbol{u}} \sim \psi_{ce}+\psi_{ac}\cdot\psi_{ce}+\psi_{ac}\cdot\psi_{cv}$	0.00713173	0.20174	57173.3
963	$\overline{\boldsymbol{u}} \sim \psi_{ac}\cdot\psi_{ce}+\psi_{ce}\cdot\psi_{ev}$	0.155705	0.20142	57184.4
964	$\overline{\boldsymbol{u}} \sim \psi_{ac}\cdot\psi_{ce}+\psi_{ac}\cdot\psi_{ev}+\psi_{ac}\cdot\psi_{cv}$	0.171885	0.19985	57245
965	$\overline{\boldsymbol{u}} \sim \psi_{ac}+\psi_{ce}\cdot\psi_{ev}+\psi_{ce}\cdot\psi_{cv}$	0.125537	0.19528	57418
966	$\overline{\boldsymbol{u}} \sim \psi_{cv}+\psi_{ac}\cdot\psi_{ce}+\psi_{ac}\cdot\psi_{ev}+\psi_{ce}\cdot\psi_{cv}$	0.199698	0.19473	57439.5
967	$\overline{\boldsymbol{u}} \sim \psi_{ce}+\psi_{cv}+\psi_{ac}\cdot\psi_{ce}+\psi_{ac}\cdot\psi_{ev}$	0.20579	0.19182	57549
968	$\overline{\boldsymbol{u}} \sim \psi_{ce}+\psi_{ac}\cdot\psi_{ce}+\psi_{ac}\cdot\psi_{ev}$	0.230964	0.19124	57569.7

969	$\overline{\boldsymbol{u}} \sim \psi_{ac}+\psi_{ce}\cdot\psi_{cv}$	0.112139	0.19077	57586.5
970	$\overline{\boldsymbol{u}} \sim \psi_{cv}+\psi_{ac}\cdot\psi_{ev}+\psi_{ce}\cdot\psi_{cv}$	0.274025	0.18849	57672.7
971	$\overline{\boldsymbol{u}} \sim \psi_{cv}+\psi_{ac}\cdot\psi_{ce}+\psi_{ac}\cdot\psi_{ev}$	0.173186	0.18745	57711.8
972	$\overline{\boldsymbol{u}} \sim \psi_{ev}+\psi_{ac}\cdot\psi_{cv}$	0.427141	0.18698	57728.4
973	$\overline{\boldsymbol{u}} \sim \psi_{ac}+\psi_{ac}\cdot\psi_{cv}+\psi_{ce}\cdot\psi_{ev}$	0.0924475	0.18381	57847.2
974	$\overline{\boldsymbol{u}} \sim \psi_{ac}\cdot\psi_{ce}+\psi_{ac}\cdot\psi_{ev}$	0.177793	0.18375	57848.6
975	$\overline{\boldsymbol{u}} \sim \psi_{ac}\cdot\psi_{ce}+\psi_{ac}\cdot\psi_{ev}+\psi_{ce}\cdot\psi_{cv}$	0.176787	0.18375	57849.6
976	$\overline{\boldsymbol{u}} \sim \psi_{ac}+\psi_{ac}\cdot\psi_{cv}$	0.0932158	0.18367	57851.5
977	$\overline{\boldsymbol{u}} \sim \psi_{ac}+\psi_{ce}\cdot\psi_{ev}$	0.0972367	0.17476	58180.7
978	$\overline{\boldsymbol{u}} \sim \psi_{ac}$	0.098304	0.17458	58186.3
979	$\overline{\boldsymbol{u}} \sim \psi_{ce}+\psi_{ac}\cdot\psi_{cv}$	0.0495366	0.17398	58209.6
980	$\overline{\boldsymbol{u}} \sim \psi_{ev}+\psi_{cv}$	3.51367	0.17205	58280.2
981	$\overline{\boldsymbol{u}} \sim \psi_{ev}+\psi_{cv}+\psi_{ev}\cdot\psi_{cv}$	3.51375	0.17203	58282.2
982	$\overline{\boldsymbol{u}} \sim \psi_{ev}$	3.86362	0.16994	58356.5
983	$\overline{\boldsymbol{u}} \sim \psi_{ev}+\psi_{ev}\cdot\psi_{cv}$	0.656728	0.16957	58370.9
984	$\overline{\boldsymbol{u}} \sim \psi_{ce}+\psi_{cv}+\psi_{ac}\cdot\psi_{ce}+\psi_{ce}\cdot\psi_{cv}$	0.0691333	0.15748	58811.4
985	$\overline{\boldsymbol{u}} \sim \psi_{ce}+\psi_{cv}+\psi_{ce}\cdot\psi_{cv}$	0.0847726	0.15697	58829
986	$\overline{\boldsymbol{u}} \sim \psi_{ce}+\psi_{cv}$	0.0814312	0.15286	58975.5
987	$\overline{\boldsymbol{u}} \sim \psi_{ce}+\psi_{cv}+\psi_{ac}\cdot\psi_{ce}$	0.0817576	0.15283	58977.4
988	$\overline{\boldsymbol{u}} \sim \psi_{ce}+\psi_{ac}\cdot\psi_{ce}+\psi_{ce}\cdot\psi_{cv}$	0.0673505	0.15212	59002.8
989	$\overline{\boldsymbol{u}} \sim \psi_{ce}+\psi_{ac}\cdot\psi_{ce}$	0.0725336	0.15205	59004.3
990	$\overline{\boldsymbol{u}} \sim \psi_{ce}$	0.07859	0.15186	59010.4
991	$\overline{\boldsymbol{u}} \sim \psi_{ce}+\psi_{ce}\cdot\psi_{cv}$	0.0786708	0.15183	59012.3
992	$\overline{\boldsymbol{u}} \sim \psi_{cv}+\psi_{ac}\cdot\psi_{ce}+\psi_{ce}\cdot\psi_{cv}$	0.106415	0.1506	59057.2
993	$\overline{\boldsymbol{u}} \sim \psi_{cv}+\psi_{ce}\cdot\psi_{cv}$	0.0993006	0.14917	59107.2
994	$\overline{\boldsymbol{u}} \sim \psi_{ac}\cdot\psi_{ce}+\psi_{ac}\cdot\psi_{cv}+\psi_{ce}\cdot\psi_{cv}$	0.121663	0.14366	59304.1
995	$\overline{\boldsymbol{u}} \sim \psi_{cv}+\psi_{ev}\cdot\psi_{cv}$	0.217962	0.13875	59476.6

996	$\overline{\boldsymbol{u}} \sim \psi_{ac}{\cdot}\psi_{ev}{+}\psi_{ac}{\cdot}\psi_{cv}{+}\psi_{ce}{\cdot}\psi_{ev}{+}\psi_{ce}{\cdot}\psi_{cv}$	0.61229	0.13835	59492.5
997	$\overline{\boldsymbol{u}} \sim \psi_{ac}{\cdot}\psi_{ev}{+}\psi_{ce}{\cdot}\psi_{ev}{+}\psi_{ce}{\cdot}\psi_{cv}$	0.596037	0.13742	59524.4
998	$\overline{\boldsymbol{u}} \sim \psi_{cv}{+}\psi_{ac}{\cdot}\psi_{ce}$	0.117083	0.13556	59588.7
999	$\overline{\boldsymbol{u}} \sim \psi_{ac}{\cdot}\psi_{ev}{+}\psi_{ac}{\cdot}\psi_{cv}{+}\psi_{ce}{\cdot}\psi_{cv}$	0.558446	0.13533	59597.9
1000	$\overline{\boldsymbol{u}} \sim \psi_{ac}{\cdot}\psi_{ev}{+}\psi_{ce}{\cdot}\psi_{cv}$	0.554436	0.13531	59597.3
1001	$\overline{\boldsymbol{u}} \sim \psi_{ac}{\cdot}\psi_{ce}{+}\psi_{ac}{\cdot}\psi_{cv}$	0.119779	0.13136	59735.8
1002	$\overline{\boldsymbol{u}} \sim \psi_{ac}{\cdot}\psi_{ce}{+}\psi_{ce}{\cdot}\psi_{cv}$	0.122492	0.12619	59915.8
1003	$\overline{\boldsymbol{u}} \sim \psi_{ac}{\cdot}\psi_{ce}$	0.122775	0.12602	59920.6
1004	$\overline{\boldsymbol{u}} \sim \psi_{ac}{\cdot}\psi_{ev}{+}\psi_{ce}{\cdot}\psi_{ev}{+}\psi_{ev}{\cdot}\psi_{cv}$	0.246792	0.1161	60265
1005	$\overline{\boldsymbol{u}} \sim \psi_{ce}{\cdot}\psi_{ev}{+}\psi_{ev}{\cdot}\psi_{cv}$	0.176952	0.11488	60306.1
1006	$\overline{\boldsymbol{u}} \sim \psi_{ac}{\cdot}\psi_{ev}{+}\psi_{ac}{\cdot}\psi_{cv}{+}\psi_{ce}{\cdot}\psi_{ev}$	0.336188	0.11441	60323
1007	$\overline{\boldsymbol{u}} \sim \psi_{ac}{\cdot}\psi_{cv}{+}\psi_{ce}{\cdot}\psi_{ev}{+}\psi_{ce}{\cdot}\psi_{cv}$	0.216871	0.10002	60812
1008	$\overline{\boldsymbol{u}} \sim \psi_{ce}{\cdot}\psi_{ev}{+}\psi_{ce}{\cdot}\psi_{cv}$	0.159679	0.08992	61149.6
1009	$\overline{\boldsymbol{u}} \sim \psi_{ac}{\cdot}\psi_{ev}{+}\psi_{ev}{\cdot}\psi_{cv}$	0.120845	0.0898	61153.6
1010	$\overline{\boldsymbol{u}} \sim \psi_{ev}{\cdot}\psi_{cv}$	0.241946	0.0872	61239.3
1011	$\overline{\boldsymbol{u}} \sim \psi_{ac}{\cdot}\psi_{ev}{+}\psi_{ce}{\cdot}\psi_{ev}$	0.663992	0.08166	61423.7
1012	$\overline{\boldsymbol{u}} \sim \psi_{cv}{+}\psi_{ac}{\cdot}\psi_{ev}{+}\psi_{ce}{\cdot}\psi_{ev}$	0.665223	0.08164	61425.4
1013	$\overline{\boldsymbol{u}} \sim \psi_{ac}{\cdot}\psi_{cv}{+}\psi_{ce}{\cdot}\psi_{cv}$	0.111631	0.07704	61575.8
1014	$\overline{\boldsymbol{u}} \sim \psi_{ce}{\cdot}\psi_{cv}$	0.0848485	0.07666	61587.4
1015	$\overline{\boldsymbol{u}} \sim \psi_{ac}{\cdot}\psi_{ev}{+}\psi_{ac}{\cdot}\psi_{cv}$	0.530039	0.0746	61656.2
1016	$\overline{\boldsymbol{u}} \sim \psi_{cv}{+}\psi_{ac}{\cdot}\psi_{ev}$	0.845785	0.06346	62019
1017	$\overline{\boldsymbol{u}} \sim \psi_{ac}{\cdot}\psi_{ev}$	0.841342	0.06258	62046.6
1018	$\overline{\boldsymbol{u}} \sim \psi_{ac}{\cdot}\psi_{cv}{+}\psi_{ce}{\cdot}\psi_{ev}$	0.0104698	0.03565	62906.9
1019	$\overline{\boldsymbol{u}} \sim \psi_{ac}{\cdot}\psi_{cv}$	0.0117169	0.03266	62999.9
1020	$\overline{\boldsymbol{u}} \sim \psi_{cv}{+}\psi_{ce}{\cdot}\psi_{ev}$	0.00362754	0.00604	63824.4
1021	$\overline{\boldsymbol{u}} \sim \psi_{ce}{\cdot}\psi_{ev}$	0.00159754	0.00439	63873.7
1022	$\overline{\boldsymbol{u}} \sim \psi_{cv}$	0.155392	0.00221	63940.2
