# Supplementary material for: Influence of interaction of cerebral fluids on ventricular deformation: A mathematical approach
Source: PLoS One. 2022 Feb 28;17(2):e0264395. doi: 10.1371/journal.pone.0264395 (PMC8884699; doi:10.1371/journal.pone.0264395)
Supplement: S4 File — The file provides information about linear regression models for volunteer 1, which were constructed when searching for bopt. For each regression model, the regression formula, the value of bopt, Radj.2 and the value of the Akaike information criterion (AIC) are given. (PDF) [file pone.0264395.s005.pdf]

Table 1: Regression results for volunteer 4

| N | Formula | Optimal b | $R^2_{adj.}$ | AIC |
|---|---|---|---|---|
| 0 | DisplMean˜ $\psi_{ac}+\psi_{ce}+\psi_{ev}+\psi_{cv}+\psi_{ac}:\psi_{ce}+\psi_{ac}:\psi_{ev}+\psi_{ac}:\psi_{cv}+\psi_{ce}:\psi_{ev}+\psi_{ce}:\psi_{cv}+\psi_{ev}:\psi_{cv}$ | 0.189656 | 0.93053 | -9770.01 |
| 1 | DisplMean˜ $\psi_{ac}+\psi_{ce}+\psi_{ev}+\psi_{cv}+\psi_{ac}:\psi_{ce}+\psi_{ac}:\psi_{ev}+\psi_{ac}:\psi_{cv}+\psi_{ce}:\psi_{cv}+\psi_{ev}:\psi_{cv}$ | 0.189543 | 0.93052 | -9768.5 |
| 2 | DisplMean˜ $\psi_{ac}+\psi_{ce}+\psi_{ev}+\psi_{cv}+\psi_{ac}:\psi_{ce}+\psi_{ac}:\psi_{ev}+\psi_{ac}:\psi_{cv}+\psi_{ce}:\psi_{ev}+\psi_{ce}:\psi_{cv}$ | 0.188814 | 0.93035 | -9693.45 |
| 3 | DisplMean˜ $\psi_{ac}+\psi_{ce}+\psi_{ev}+\psi_{cv}+\psi_{ac}:\psi_{ce}+\psi_{ac}:\psi_{ev}+\psi_{ac}:\psi_{cv}+\psi_{ce}:\psi_{cv}$ | 0.188624 | 0.93034 | -9687.53 |
| 4 | DisplMean˜ $\psi_{ac}+\psi_{ce}+\psi_{ev}+\psi_{cv}+\psi_{ac}:\psi_{ce}+\psi_{ac}:\psi_{cv}+\psi_{ce}:\psi_{ev}+\psi_{ce}:\psi_{cv}+\psi_{ev}:\psi_{cv}$ | 0.184923 | 0.9297 | -9409.28 |
| 5 | DisplMean˜ $\psi_{ac}+\psi_{ce}+\psi_{ev}+\psi_{cv}+\psi_{ac}:\psi_{ce}+\psi_{ac}:\psi_{cv}+\psi_{ce}:\psi_{ev}+\psi_{ce}:\psi_{cv}$ | 0.184893 | 0.92969 | -9406.14 |
| 6 | DisplMean˜ $\psi_{ac}+\psi_{ce}+\psi_{ev}+\psi_{cv}+\psi_{ac}:\psi_{ce}+\psi_{ac}:\psi_{cv}+\psi_{ce}:\psi_{cv}+\psi_{ev}:\psi_{cv}$ | 0.184316 | 0.92963 | -9378.77 |
| 7 | DisplMean˜ $\psi_{ac}+\psi_{ce}+\psi_{ev}+\psi_{cv}+\psi_{ac}:\psi_{ce}+\psi_{ac}:\psi_{cv}+\psi_{ce}:\psi_{cv}$ | 0.184275 | 0.92962 | -9374.58 |
| 8 | DisplMean˜ $\psi_{ac}+\psi_{ce}+\psi_{ev}+\psi_{cv}+\psi_{ac}:\psi_{ce}+\psi_{ac}:\psi_{ev}+\psi_{ce}:\psi_{cv}+\psi_{ev}:\psi_{cv}$ | 0.160616 | 0.92665 | -8116 |
| 9 | DisplMean˜ $\psi_{ac}+\psi_{ce}+\psi_{ev}+\psi_{cv}+\psi_{ac}:\psi_{ce}+\psi_{ac}:\psi_{ev}+\psi_{ce}:\psi_{ev}+\psi_{ce}:\psi_{cv}+\psi_{ev}:\psi_{cv}$ | 0.160675 | 0.92665 | -8116.15 |
| 10 | DisplMean˜ $\psi_{ac}+\psi_{ce}+\psi_{ev}+\psi_{cv}+\psi_{ac}:\psi_{ce}+\psi_{ac}:\psi_{ev}+\psi_{ce}:\psi_{ev}+\psi_{ce}:\psi_{cv}$ | 0.16018 | 0.9265 | -8054.79 |
| 11 | DisplMean˜ $\psi_{ac}+\psi_{ce}+\psi_{ev}+\psi_{cv}+\psi_{ac}:\psi_{ce}+\psi_{ac}:\psi_{ev}+\psi_{ce}:\psi_{cv}$ | 0.160073 | 0.92649 | -8051.41 |
| 12 | DisplMean˜ $\psi_{ac}+\psi_{ce}+\psi_{ev}+\psi_{cv}+\psi_{ac}:\psi_{ce}+\psi_{ce}:\psi_{ev}+\psi_{ce}:\psi_{cv}+\psi_{ev}:\psi_{cv}$ | 0.157885 | 0.92596 | -7830.28 |
| 13 | DisplMean˜ $\psi_{ac}+\psi_{ce}+\psi_{ev}+\psi_{cv}+\psi_{ac}:\psi_{ce}+\psi_{ce}:\psi_{ev}+\psi_{ce}:\psi_{cv}$ | 0.157867 | 0.92595 | -7827.99 |
| 14 | DisplMean˜ $\psi_{ac}+\psi_{ce}+\psi_{ev}+\psi_{cv}+\psi_{ac}:\psi_{ce}+\psi_{ce}:\psi_{cv}+\psi_{ev}:\psi_{cv}$ | 0.157529 | 0.9259 | -7808.3 |
| 15 | DisplMean˜ $\psi_{ac}+\psi_{ce}+\psi_{ev}+\psi_{cv}+\psi_{ac}:\psi_{ce}+\psi_{ce}:\psi_{cv}$ | 0.157505 | 0.92589 | -7805.17 |
| 16 | DisplMean˜ $\psi_{ac}+\psi_{ce}+\psi_{cv}+\psi_{ac}:\psi_{ce}+\psi_{ac}:\psi_{ev}+\psi_{ac}:\psi_{cv}+\psi_{ce}:\psi_{ev}+\psi_{ce}:\psi_{cv}+\psi_{ev}:\psi_{cv}$ | 0.171092 | 0.92099 | -5852.28 |
| 17 | DisplMean˜ $\psi_{ac}+\psi_{ce}+\psi_{cv}+\psi_{ac}:\psi_{ce}+\psi_{ac}:\psi_{ev}+\psi_{ac}:\psi_{cv}+\psi_{ce}:\psi_{cv}+\psi_{ev}:\psi_{cv}$ | 0.171782 | 0.92049 | -5658.67 |
| 18 | DisplMean˜ $\psi_{ac}+\psi_{ce}+\psi_{cv}+\psi_{ac}:\psi_{ce}+\psi_{ac}:\psi_{ev}+\psi_{ac}:\psi_{cv}+\psi_{ce}:\psi_{ev}+\psi_{ce}:\psi_{cv}$ | 0.169555 | 0.91997 | -5460.85 |
| 19 | DisplMean˜ $\psi_{ac}+\psi_{ce}+\psi_{cv}+\psi_{ac}:\psi_{ce}+\psi_{ac}:\psi_{ev}+\psi_{ac}:\psi_{cv}+\psi_{ce}:\psi_{cv}$ | 0.170282 | 0.91954 | -5300.77 |
| 20 | DisplMean˜ $\psi_{ac}+\psi_{ce}+\psi_{ev}+\psi_{ac}:\psi_{ce}+\psi_{ac}:\psi_{ev}+\psi_{ac}:\psi_{cv}+\psi_{ce}:\psi_{ev}+\psi_{ce}:\psi_{cv}+\psi_{ev}:\psi_{cv}$ | 0.15583 | 0.91847 | -4895.19 |
| 21 | DisplMean˜ $\psi_{ac}+\psi_{ce}+\psi_{ev}+\psi_{ac}:\psi_{ce}+\psi_{ac}:\psi_{ev}+\psi_{ac}:\psi_{cv}+\psi_{ce}:\psi_{cv}+\psi_{ev}:\psi_{cv}$ | 0.155892 | 0.91846 | -4891.96 |
| 22 | DisplMean˜ $\psi_{ac}+\psi_{ce}+\psi_{cv}+\psi_{ac}:\psi_{ce}+\psi_{ac}:\psi_{ev}+\psi_{ce}:\psi_{ev}+\psi_{ce}:\psi_{cv}+\psi_{ev}:\psi_{cv}$ | 0.149933 | 0.91761 | -4575.78 |
| 23 | DisplMean˜ $\psi_{ac}+\psi_{ce}+\psi_{ev}+\psi_{ac}:\psi_{ce}+\psi_{ac}:\psi_{ev}+\psi_{ce}:\psi_{ev}+\psi_{ce}:\psi_{cv}+\psi_{ev}:\psi_{cv}$ | 0.165621 | 0.91726 | -4448.71 |

| | | | | |
|---|---|---|---|---|
| 24 | DisplMean~ $\psi_{ac}$+$\psi_{ce}$+$\psi_{ev}$+$\psi_{ac}$:$\psi_{ce}$+$\psi_{ac}$:$\psi_{ev}$+$\psi_{ce}$:$\psi_{cv}$+$\psi_{ev}$:$\psi_{cv}$ | 0.165834 | 0.91723 | -4438.47 |
| 25 | DisplMean~ $\psi_{ac}$+$\psi_{ce}$+$\psi_{cv}$+$\psi_{ac}$:$\psi_{ce}$+$\psi_{ac}$:$\psi_{ev}$+$\psi_{ce}$:$\psi_{cv}$+$\psi_{ev}$:$\psi_{cv}$ | 0.150526 | 0.91709 | -4383.81 |
| 26 | DisplMean~ $\psi_{ac}$+$\psi_{ce}$+$\psi_{cv}$+$\psi_{ac}$:$\psi_{ce}$+$\psi_{ac}$:$\psi_{ev}$+$\psi_{ce}$:$\psi_{ev}$+$\psi_{ce}$:$\psi_{cv}$ | 0.148948 | 0.91664 | -4221.89 |
| 27 | DisplMean~ $\psi_{ac}$+$\psi_{ce}$+$\psi_{ev}$+$\psi_{ac}$:$\psi_{ce}$+$\psi_{ac}$:$\psi_{cv}$+$\psi_{ce}$:$\psi_{ev}$+$\psi_{ce}$:$\psi_{cv}$+$\psi_{ev}$:$\psi_{cv}$ | 0.149769 | 0.91647 | -4159.48 |
| 28 | DisplMean~ $\psi_{ac}$+$\psi_{ce}$+$\psi_{ev}$+$\psi_{ac}$:$\psi_{ce}$+$\psi_{ac}$:$\psi_{cv}$+$\psi_{ce}$:$\psi_{cv}$+$\psi_{ev}$:$\psi_{cv}$ | 0.149566 | 0.91645 | -4152.77 |
| 29 | DisplMean~ $\psi_{ac}$+$\psi_{ce}$+$\psi_{cv}$+$\psi_{ac}$:$\psi_{ce}$+$\psi_{ac}$:$\psi_{cv}$+$\psi_{ce}$:$\psi_{ev}$+$\psi_{ce}$:$\psi_{cv}$+$\psi_{ev}$:$\psi_{cv}$ | 0.166057 | 0.91625 | -4076.89 |
| 30 | DisplMean~ $\psi_{ac}$+$\psi_{ce}$+$\psi_{cv}$+$\psi_{ac}$:$\psi_{ce}$+$\psi_{ac}$:$\psi_{ev}$+$\psi_{ce}$:$\psi_{cv}$ | 0.149561 | 0.9162 | -4061.34 |
| 31 | DisplMean~ $\psi_{ac}$+$\psi_{ce}$+$\psi_{ev}$+$\psi_{ac}$:$\psi_{ce}$+$\psi_{ce}$:$\psi_{ev}$+$\psi_{ce}$:$\psi_{cv}$+$\psi_{ev}$:$\psi_{cv}$ | 0.160987 | 0.91436 | -3398.08 |
| 32 | DisplMean~ $\psi_{ac}$+$\psi_{ce}$+$\psi_{ev}$+$\psi_{ac}$:$\psi_{ce}$+$\psi_{ce}$:$\psi_{cv}$+$\psi_{ev}$:$\psi_{cv}$ | 0.160772 | 0.91434 | -3393.92 |
| 33 | DisplMean~ $\psi_{ac}$+$\psi_{ce}$+$\psi_{cv}$+$\psi_{ac}$:$\psi_{ce}$+$\psi_{ce}$:$\psi_{ev}$+$\psi_{ce}$:$\psi_{cv}$+$\psi_{ev}$:$\psi_{cv}$ | 0.146926 | 0.91294 | -2897.24 |
| 34 | DisplMean~ $\psi_{ac}$+$\psi_{ce}$+$\psi_{ev}$+$\psi_{ac}$:$\psi_{ce}$+$\psi_{ac}$:$\psi_{ev}$+$\psi_{ac}$:$\psi_{cv}$+$\psi_{ce}$:$\psi_{ev}$+$\psi_{ce}$:$\psi_{cv}$ | 0.143454 | 0.91284 | -2861.79 |
| 35 | DisplMean~ $\psi_{ac}$+$\psi_{ce}$+$\psi_{ev}$+$\psi_{ac}$:$\psi_{ce}$+$\psi_{ac}$:$\psi_{ev}$+$\psi_{ac}$:$\psi_{cv}$+$\psi_{ce}$:$\psi_{cv}$ | 0.143381 | 0.91283 | -2860.73 |
| 36 | DisplMean~ $\psi_{ac}$+$\psi_{ce}$+$\psi_{ev}$+$\psi_{ac}$:$\psi_{ce}$+$\psi_{ac}$:$\psi_{cv}$+$\psi_{ce}$:$\psi_{ev}$+$\psi_{ce}$:$\psi_{cv}$ | 0.141763 | 0.91239 | -2705.4 |
| 37 | DisplMean~ $\psi_{ac}$+$\psi_{ce}$+$\psi_{ev}$+$\psi_{ac}$:$\psi_{ce}$+$\psi_{ac}$:$\psi_{cv}$+$\psi_{ce}$:$\psi_{cv}$ | 0.141503 | 0.91235 | -2691.65 |
| 38 | DisplMean~ $\psi_{ac}$+$\psi_{ce}$+$\psi_{ac}$:$\psi_{ce}$+$\psi_{ac}$:$\psi_{ev}$+$\psi_{ac}$:$\psi_{cv}$+$\psi_{ce}$:$\psi_{ev}$+$\psi_{ce}$:$\psi_{cv}$+$\psi_{ev}$:$\psi_{cv}$ | 0.147417 | 0.91139 | -2358.88 |
| 39 | DisplMean~ $\psi_{ac}$+$\psi_{ce}$+$\psi_{ac}$:$\psi_{ce}$+$\psi_{ac}$:$\psi_{ev}$+$\psi_{ac}$:$\psi_{cv}$+$\psi_{ce}$:$\psi_{cv}$+$\psi_{ev}$:$\psi_{cv}$ | 0.147892 | 0.91072 | -2131.7 |
| 40 | DisplMean~ $\psi_{ac}$+$\psi_{ce}$+$\psi_{ac}$:$\psi_{ce}$+$\psi_{ac}$:$\psi_{ev}$+$\psi_{ce}$:$\psi_{ev}$+$\psi_{ce}$:$\psi_{cv}$+$\psi_{ev}$:$\psi_{cv}$ | 0.153888 | 0.91066 | -2109.69 |
| 41 | DisplMean~ $\psi_{ac}$+$\psi_{ce}$+$\psi_{ac}$:$\psi_{ce}$+$\psi_{ac}$:$\psi_{cv}$+$\psi_{ce}$:$\psi_{ev}$+$\psi_{ce}$:$\psi_{cv}$+$\psi_{ev}$:$\psi_{cv}$ | 0.148558 | 0.9102 | -1955.49 |
| 42 | DisplMean~ $\psi_{ac}$+$\psi_{ce}$+$\psi_{ac}$:$\psi_{ce}$+$\psi_{ce}$:$\psi_{ev}$+$\psi_{ce}$:$\psi_{cv}$+$\psi_{ev}$:$\psi_{cv}$ | 0.151556 | 0.91006 | -1907.62 |
| 43 | DisplMean~ $\psi_{ac}$+$\psi_{ce}$+$\psi_{ac}$:$\psi_{ce}$+$\psi_{ac}$:$\psi_{ev}$+$\psi_{ce}$:$\psi_{cv}$+$\psi_{ev}$:$\psi_{cv}$ | 0.154567 | 0.90995 | -1869.18 |
| 44 | DisplMean~ $\psi_{ac}$+$\psi_{ce}$+$\psi_{cv}$+$\psi_{ac}$:$\psi_{ce}$+$\psi_{ac}$:$\psi_{cv}$+$\psi_{ce}$:$\psi_{cv}$+$\psi_{ev}$:$\psi_{cv}$ | 0.165116 | 0.90951 | -1722.01 |
| 45 | DisplMean~ $\psi_{ac}$+$\psi_{ce}$+$\psi_{ev}$+$\psi_{cv}$+$\psi_{ac}$:$\psi_{ce}$+$\psi_{ac}$:$\psi_{ev}$+$\psi_{ac}$:$\psi_{cv}$+$\psi_{ev}$:$\psi_{cv}$ | 0.228802 | 0.90729 | -980.254 |
| 46 | DisplMean~ $\psi_{ac}$+$\psi_{ce}$+$\psi_{ev}$+$\psi_{cv}$+$\psi_{ac}$:$\psi_{ce}$+$\psi_{ac}$:$\psi_{ev}$+$\psi_{ac}$:$\psi_{cv}$+$\psi_{ce}$:$\psi_{ev}$+$\psi_{ev}$:$\psi_{cv}$ | 0.228945 | 0.90729 | -981.107 |
| 47 | DisplMean~ $\psi_{ac}$+$\psi_{ce}$+$\psi_{ev}$+$\psi_{cv}$+$\psi_{ac}$:$\psi_{ce}$+$\psi_{ac}$:$\psi_{ev}$+$\psi_{ac}$:$\psi_{cv}$+$\psi_{ce}$:$\psi_{ev}$ | 0.227925 | 0.90712 | -924.775 |
| 48 | DisplMean~ $\psi_{ac}$+$\psi_{ce}$+$\psi_{ev}$+$\psi_{cv}$+$\psi_{ac}$:$\psi_{ce}$+$\psi_{ac}$:$\psi_{ev}$+$\psi_{ac}$:$\psi_{cv}$ | 0.227689 | 0.9071 | -920.581 |
| 49 | DisplMean~ $\psi_{ac}$+$\psi_{ce}$+$\psi_{ev}$+$\psi_{cv}$+$\psi_{ac}$:$\psi_{ce}$+$\psi_{ac}$:$\psi_{cv}$+$\psi_{ce}$:$\psi_{ev}$+$\psi_{ev}$:$\psi_{cv}$ | 0.223145 | 0.90646 | -708.697 |
| 50 | DisplMean~ $\psi_{ac}$+$\psi_{ce}$+$\psi_{ev}$+$\psi_{cv}$+$\psi_{ac}$:$\psi_{ce}$+$\psi_{ac}$:$\psi_{cv}$+$\psi_{ce}$:$\psi_{ev}$ | 0.223108 | 0.90645 | -706.928 |

| | | | | |
|---|---|---|---|---|
| 51 | DisplMean~ $\psi_{ac}+\psi_{ce}+\psi_{ev}+\psi_{cv}+\psi_{ac}:\psi_{ce}+\psi_{ac}:\psi_{cv}+\psi_{ev}:\psi_{cv}$ | 0.222399 | 0.90638 | -685.524 |
| 52 | DisplMean~ $\psi_{ac}+\psi_{ce}+\psi_{ev}+\psi_{cv}+\psi_{ac}:\psi_{ce}+\psi_{ac}:\psi_{cv}$ | 0.222349 | 0.90637 | -682.981 |
| 53 | DisplMean~ $\psi_{ac}+\psi_{ce}+\psi_{cv}+\psi_{ac}:\psi_{ce}+\psi_{ce}:\psi_{cv}+\psi_{ev}:\psi_{cv}$ | 0.146861 | 0.90619 | -622.844 |
| 54 | DisplMean~ $\psi_{ac}+\psi_{ce}+\psi_{ac}:\psi_{ce}+\psi_{ac}:\psi_{cv}+\psi_{ce}:\psi_{cv}+\psi_{ev}:\psi_{cv}$ | 0.151897 | 0.90618 | -622.416 |
| 55 | DisplMean~ $\psi_{ac}+\psi_{ce}+\psi_{ac}:\psi_{ce}+\psi_{ce}:\psi_{cv}+\psi_{ev}:\psi_{cv}$ | 0.148242 | 0.90594 | -543.658 |
| 56 | DisplMean~ $\psi_{ac}+\psi_{ce}+\psi_{ac}:\psi_{ce}+\psi_{ac}:\psi_{ev}+\psi_{ac}:\psi_{cv}+\psi_{ce}:\psi_{ev}+\psi_{ce}:\psi_{cv}$ | 0.135248 | 0.90347 | 248.07 |
| 57 | DisplMean~ $\psi_{ac}+\psi_{ce}+\psi_{ac}:\psi_{ce}+\psi_{ac}:\psi_{ev}+\psi_{ac}:\psi_{cv}+\psi_{ce}:\psi_{cv}$ | 0.135836 | 0.90301 | 391.237 |
| 58 | DisplMean~ $\psi_{ac}+\psi_{ce}+\psi_{ev}+\psi_{ac}:\psi_{ce}+\psi_{ac}:\psi_{ev}+\psi_{ac}:\psi_{cv}+\psi_{ev}:\psi_{cv}$ | 0.191964 | 0.89967 | 1423.06 |
| 59 | DisplMean~ $\psi_{ac}+\psi_{ce}+\psi_{ev}+\psi_{ac}:\psi_{ce}+\psi_{ac}:\psi_{ev}+\psi_{ac}:\psi_{cv}+\psi_{ce}:\psi_{ev}+\psi_{ev}:\psi_{cv}$ | 0.191923 | 0.89967 | 1423.47 |
| 60 | DisplMean~ $\psi_{ac}+\psi_{ce}+\psi_{ev}+\psi_{ac}:\psi_{ce}+\psi_{ac}:\psi_{ev}+\psi_{ce}:\psi_{cv}$ | 0.166115 | 0.89922 | 1560.37 |
| 61 | DisplMean~ $\psi_{ac}+\psi_{ce}+\psi_{ev}+\psi_{ac}:\psi_{ce}+\psi_{ac}:\psi_{ev}+\psi_{ce}:\psi_{ev}+\psi_{ce}:\psi_{cv}$ | 0.166212 | 0.89922 | 1559.35 |
| 62 | DisplMean~ $\psi_{ac}+\psi_{ce}+\psi_{cv}+\psi_{ac}:\psi_{ce}+\psi_{ac}:\psi_{cv}+\psi_{ce}:\psi_{ev}+\psi_{ce}:\psi_{cv}$ | 0.150505 | 0.89881 | 1684 |
| 63 | DisplMean~ $\psi_{ac}+\psi_{ce}+\psi_{ev}+\psi_{ac}:\psi_{ce}+\psi_{ce}:\psi_{ev}+\psi_{ce}:\psi_{cv}$ | 0.164019 | 0.89875 | 1702.05 |
| 64 | DisplMean~ $\psi_{ac}+\psi_{ce}+\psi_{ev}+\psi_{ac}:\psi_{ce}+\psi_{ce}:\psi_{cv}$ | 0.16368 | 0.8987 | 1714.96 |
| 65 | DisplMean~ $\psi_{ac}+\psi_{ce}+\psi_{ev}+\psi_{ac}:\psi_{ce}+\psi_{ac}:\psi_{cv}+\psi_{ce}:\psi_{ev}+\psi_{ev}:\psi_{cv}$ | 0.184543 | 0.89796 | 1939.18 |
| 66 | DisplMean~ $\psi_{ac}+\psi_{ce}+\psi_{ev}+\psi_{ac}:\psi_{ce}+\psi_{ac}:\psi_{cv}+\psi_{ev}:\psi_{cv}$ | 0.184256 | 0.89793 | 1946.97 |
| 67 | DisplMean~ $\psi_{ac}+\psi_{ce}+\psi_{cv}+\psi_{ac}:\psi_{ce}+\psi_{ac}:\psi_{ev}+\psi_{ac}:\psi_{cv}+\psi_{ce}:\psi_{ev}+\psi_{ev}:\psi_{cv}$ | 0.202169 | 0.89754 | 2065.86 |
| 68 | DisplMean~ $\psi_{ac}+\psi_{ce}+\psi_{cv}+\psi_{ac}:\psi_{ce}+\psi_{ac}:\psi_{ev}+\psi_{ac}:\psi_{cv}+\psi_{ev}:\psi_{cv}$ | 0.202844 | 0.89705 | 2207.77 |
| 69 | DisplMean~ $\psi_{ac}+\psi_{ce}+\psi_{ev}+\psi_{ac}:\psi_{ce}+\psi_{ac}:\psi_{ev}+\psi_{ev}:\psi_{cv}$ | 0.183347 | 0.89667 | 2321.07 |
| 70 | DisplMean~ $\psi_{ac}+\psi_{ce}+\psi_{ev}+\psi_{ac}:\psi_{ce}+\psi_{ac}:\psi_{ev}+\psi_{ce}:\psi_{ev}+\psi_{ev}:\psi_{cv}$ | 0.183397 | 0.89667 | 2321.84 |
| 71 | DisplMean~ $\psi_{ac}+\psi_{ce}+\psi_{ev}+\psi_{cv}+\psi_{ac}:\psi_{ce}+\psi_{ac}:\psi_{ev}+\psi_{ev}:\psi_{cv}$ | 0.183363 | 0.89666 | 2323.04 |
| 72 | DisplMean~ $\psi_{ac}+\psi_{ce}+\psi_{ev}+\psi_{cv}+\psi_{ac}:\psi_{ce}+\psi_{ac}:\psi_{ev}+\psi_{ce}:\psi_{ev}+\psi_{ev}:\psi_{cv}$ | 0.183423 | 0.89666 | 2323.75 |
| 73 | DisplMean~ $\psi_{ac}+\psi_{ce}+\psi_{cv}+\psi_{ac}:\psi_{ce}+\psi_{ac}:\psi_{ev}+\psi_{ac}:\psi_{cv}+\psi_{ce}:\psi_{ev}$ | 0.20037 | 0.89654 | 2358.18 |
| 74 | DisplMean~ $\psi_{ac}+\psi_{ce}+\psi_{ev}+\psi_{cv}+\psi_{ac}:\psi_{ce}+\psi_{ac}:\psi_{ev}+\psi_{ce}:\psi_{ev}$ | 0.182907 | 0.89653 | 2361.93 |
| 75 | DisplMean~ $\psi_{ac}+\psi_{ce}+\psi_{ev}+\psi_{cv}+\psi_{ac}:\psi_{ce}+\psi_{ac}:\psi_{ev}$ | 0.182799 | 0.89652 | 2363.2 |
| 76 | DisplMean~ $\psi_{ac}+\psi_{ce}+\psi_{cv}+\psi_{ac}:\psi_{ce}+\psi_{ac}:\psi_{ev}+\psi_{ac}:\psi_{cv}$ | 0.201101 | 0.89614 | 2475.19 |
| 77 | DisplMean~ $\psi_{ac}+\psi_{ce}+\psi_{ev}+\psi_{cv}+\psi_{ac}:\psi_{ce}+\psi_{ce}:\psi_{ev}+\psi_{ev}:\psi_{cv}$ | 0.180522 | 0.89604 | 2506.68 |

| | | | | |
|---|---|---|---|---|
| 78 | DisplMean~ $\psi_{ac}+\psi_{ce}+\psi_{ev}+\psi_{cv}+\psi_{ac}:\psi_{ce}+\psi_{ce}:\psi_{ev}$ | 0.180503 | 0.89603 | 2507.39 |
| 79 | DisplMean~ $\psi_{ac}+\psi_{ce}+\psi_{ev}+\psi_{cv}+\psi_{ac}:\psi_{ce}+\psi_{ev}:\psi_{cv}$ | 0.180155 | 0.89599 | 2519.82 |
| 80 | DisplMean~ $\psi_{ac}+\psi_{ce}+\psi_{ev}+\psi_{cv}+\psi_{ac}:\psi_{ce}$ | 0.180128 | 0.89598 | 2521.05 |
| 81 | DisplMean~ $\psi_{ac}+\psi_{ce}+\psi_{ev}+\psi_{ac}:\psi_{ce}+\psi_{ce}:\psi_{ev}+\psi_{ev}:\psi_{cv}$ | 0.179779 | 0.89596 | 2528.8 |
| 82 | DisplMean~ $\psi_{ac}+\psi_{ce}+\psi_{cv}+\psi_{ac}:\psi_{ce}+\psi_{ce}:\psi_{ev}+\psi_{ce}:\psi_{cv}$ | 0.136247 | 0.89593 | 2536.3 |
| 83 | DisplMean~ $\psi_{ac}+\psi_{ce}+\psi_{ev}+\psi_{ac}:\psi_{ce}+\psi_{ev}:\psi_{cv}$ | 0.179449 | 0.89592 | 2540.53 |
| 84 | DisplMean~ $\psi_{ac}+\psi_{ce}+\psi_{ev}+\psi_{ac}:\psi_{ce}+\psi_{ac}:\psi_{ev}+\psi_{ac}:\psi_{cv}$ | 0.177352 | 0.89568 | 2612.05 |
| 85 | DisplMean~ $\psi_{ac}+\psi_{ce}+\psi_{ev}+\psi_{ac}:\psi_{ce}+\psi_{ac}:\psi_{ev}+\psi_{ac}:\psi_{cv}+\psi_{ce}:\psi_{ev}$ | 0.177451 | 0.89568 | 2610.98 |
| 86 | DisplMean~ $\psi_{ac}+\psi_{ce}+\psi_{ev}+\psi_{ac}:\psi_{ce}+\psi_{ac}:\psi_{ev}+\psi_{ce}:\psi_{ev}$ | 0.177025 | 0.89567 | 2614.58 |
| 87 | DisplMean~ $\psi_{ac}+\psi_{ce}+\psi_{ev}+\psi_{ac}:\psi_{ce}+\psi_{ac}:\psi_{ev}$ | 0.176927 | 0.89566 | 2615.63 |
| 88 | DisplMean~ $\psi_{ac}+\psi_{ce}+\psi_{ev}+\psi_{ac}:\psi_{ce}+\psi_{ac}:\psi_{cv}+\psi_{ce}:\psi_{ev}$ | 0.175232 | 0.8952 | 2751.33 |
| 89 | DisplMean~ $\psi_{ac}+\psi_{ce}+\psi_{ev}+\psi_{ac}:\psi_{ce}+\psi_{ce}:\psi_{ev}$ | 0.174815 | 0.89518 | 2754.76 |
| 90 | DisplMean~ $\psi_{ac}+\psi_{ce}+\psi_{ev}+\psi_{ac}:\psi_{ce}+\psi_{ac}:\psi_{cv}$ | 0.174887 | 0.89515 | 2764.24 |
| 91 | DisplMean~ $\psi_{ac}+\psi_{ce}+\psi_{ev}+\psi_{ac}:\psi_{ce}$ | 0.174472 | 0.89513 | 2767.63 |
| 92 | DisplMean~ $\psi_{ac}+\psi_{ce}+\psi_{cv}+\psi_{ac}:\psi_{ce}+\psi_{ac}:\psi_{cv}+\psi_{ce}:\psi_{ev}+\psi_{ev}:\psi_{cv}$ | 0.193538 | 0.89245 | 3541.96 |
| 93 | DisplMean~ $\psi_{ac}+\psi_{ce}+\psi_{ac}:\psi_{ce}+\psi_{ac}:\psi_{ev}+\psi_{ac}:\psi_{cv}+\psi_{ce}:\psi_{ev}+\psi_{ev}:\psi_{cv}$ | 0.178981 | 0.89197 | 3677.23 |
| 94 | DisplMean~ $\psi_{ac}+\psi_{ce}+\psi_{ac}:\psi_{ce}+\psi_{ac}:\psi_{ev}+\psi_{ac}:\psi_{cv}+\psi_{ev}:\psi_{cv}$ | 0.179397 | 0.89136 | 3845.53 |
| 95 | DisplMean~ $\psi_{ac}+\psi_{ce}+\psi_{ac}:\psi_{ce}+\psi_{ac}:\psi_{cv}+\psi_{ce}:\psi_{ev}+\psi_{ev}:\psi_{cv}$ | 0.179813 | 0.88985 | 4266.07 |
| 96 | DisplMean~ $\psi_{ac}+\psi_{ce}+\psi_{ac}:\psi_{ce}+\psi_{ac}:\psi_{ev}+\psi_{ce}:\psi_{ev}+\psi_{ce}:\psi_{cv}$ | 0.154518 | 0.88971 | 4305.66 |
| 97 | DisplMean~ $\psi_{ac}+\psi_{ce}+\psi_{ac}:\psi_{ce}+\psi_{ac}:\psi_{ev}+\psi_{ce}:\psi_{cv}$ | 0.155152 | 0.88927 | 4425.25 |
| 98 | DisplMean~ $\psi_{ac}+\psi_{ce}+\psi_{cv}+\psi_{ac}:\psi_{ce}+\psi_{ac}:\psi_{ev}+\psi_{ce}:\psi_{ev}+\psi_{ev}:\psi_{cv}$ | 0.170174 | 0.88776 | 4840.09 |
| 99 | DisplMean~ $\psi_{ac}+\psi_{ce}+\psi_{ac}:\psi_{ce}+\psi_{ac}:\psi_{ev}+\psi_{ce}:\psi_{ev}+\psi_{ev}:\psi_{cv}$ | 0.170831 | 0.88749 | 4913.35 |
| 100 | DisplMean~ $\psi_{ce}+\psi_{ev}+\psi_{cv}+\psi_{ac}:\psi_{ce}+\psi_{ce}:\psi_{ev}+\psi_{ce}:\psi_{cv}+\psi_{ev}:\psi_{cv}$ | 0.9333 | 0.88747 | 4918.96 |
| 101 | DisplMean~ $\psi_{ce}+\psi_{ev}+\psi_{cv}+\psi_{ac}:\psi_{ce}+\psi_{ce}:\psi_{ev}+\psi_{ce}:\psi_{cv}$ | 0.933301 | 0.88746 | 4922.03 |
| 102 | DisplMean~ $\psi_{ac}+\psi_{ce}+\psi_{cv}+\psi_{ac}:\psi_{ce}+\psi_{ac}:\psi_{ev}+\psi_{ev}:\psi_{cv}$ | 0.170766 | 0.88726 | 4974.67 |
| 103 | DisplMean~ $\psi_{ce}+\psi_{ev}+\psi_{cv}+\psi_{ac}:\psi_{ce}+\psi_{ce}:\psi_{cv}+\psi_{ev}:\psi_{cv}$ | 0.933312 | 0.8872 | 4992.47 |
| 104 | DisplMean~ $\psi_{ce}+\psi_{ev}+\psi_{cv}+\psi_{ac}:\psi_{ce}+\psi_{ce}:\psi_{cv}$ | 0.933312 | 0.88718 | 4996.88 |

| | | | | |
|---|---|---|---|---|
| 105 | DisplMean~ $\psi_{ce}+\psi_{ev}+\psi_{ac}:\psi_{ce}+\psi_{ce}:\psi_{ev}+\psi_{ce}:\psi_{cv}+\psi_{ev}:\psi_{cv}$ | 0.935119 | 0.88717 | 4998.32 |
| 106 | DisplMean~ $\psi_{ce}+\psi_{ev}+\psi_{ac}:\psi_{ce}+\psi_{ce}:\psi_{ev}+\psi_{ce}:\psi_{cv}$ | 0.935072 | 0.88713 | 5010.4 |
| 107 | DisplMean~ $\psi_{ac}+\psi_{ce}+\psi_{ac}:\psi_{ce}+\psi_{ac}:\psi_{ev}+\psi_{ev}:\psi_{cv}$ | 0.171367 | 0.88703 | 5036.69 |
| 108 | DisplMean~ $\psi_{ce}+\psi_{ev}+\psi_{ac}:\psi_{ce}+\psi_{ce}:\psi_{cv}+\psi_{ev}:\psi_{cv}$ | 0.93513 | 0.8869 | 5070.5 |
| 109 | DisplMean~ $\psi_{ac}+\psi_{ce}+\psi_{cv}+\psi_{ac}:\psi_{ce}+\psi_{ac}:\psi_{ev}+\psi_{ce}:\psi_{ev}$ | 0.169123 | 0.88687 | 5080.03 |
| 110 | DisplMean~ $\psi_{ce}+\psi_{ev}+\psi_{ac}:\psi_{ce}+\psi_{ce}:\psi_{cv}$ | 0.935083 | 0.88685 | 5084.7 |
| 111 | DisplMean~ $\psi_{ac}+\psi_{ce}+\psi_{cv}+\psi_{ac}:\psi_{ce}+\psi_{ac}:\psi_{ev}$ | 0.169741 | 0.88645 | 5193 |
| 112 | DisplMean~ $\psi_{ac}+\psi_{ce}+\psi_{ac}:\psi_{ce}+\psi_{ac}:\psi_{ev}+\psi_{ac}:\psi_{cv}+\psi_{ce}:\psi_{ev}$ | 0.164864 | 0.8861 | 5287.17 |
| 113 | DisplMean~ $\psi_{ac}+\psi_{ce}+\psi_{ac}:\psi_{ce}+\psi_{ac}:\psi_{ev}+\psi_{ce}:\psi_{ev}$ | 0.164518 | 0.88609 | 5289.37 |
| 114 | DisplMean~ $\psi_{ac}+\psi_{ce}+\psi_{ac}:\psi_{ce}+\psi_{ac}:\psi_{ev}+\psi_{ac}:\psi_{cv}$ | 0.165466 | 0.88567 | 5400.35 |
| 115 | DisplMean~ $\psi_{ac}+\psi_{ce}+\psi_{ac}:\psi_{ce}+\psi_{ac}:\psi_{ev}$ | 0.165118 | 0.88566 | 5402.63 |
| 116 | DisplMean~ $\psi_{ac}+\psi_{ce}+\psi_{cv}+\psi_{ac}:\psi_{ce}+\psi_{ac}:\psi_{cv}+\psi_{ev}:\psi_{cv}$ | 0.190654 | 0.88554 | 5436.69 |
| 117 | DisplMean~ $\psi_{ac}+\psi_{ce}+\psi_{ac}:\psi_{ce}+\psi_{ac}:\psi_{cv}+\psi_{ev}:\psi_{cv}$ | 0.182949 | 0.8847 | 5657.77 |
| 118 | DisplMean~ $\psi_{ac}+\psi_{ce}+\psi_{ac}:\psi_{ce}+\psi_{ac}:\psi_{cv}+\psi_{ce}:\psi_{ev}+\psi_{ce}:\psi_{cv}$ | 0.125182 | 0.88306 | 6090.13 |
| 119 | DisplMean~ $\psi_{ac}+\psi_{ce}+\psi_{cv}+\psi_{ac}:\psi_{ce}+\psi_{ce}:\psi_{ev}+\psi_{ev}:\psi_{cv}$ | 0.165456 | 0.8828 | 6157.55 |
| 120 | DisplMean~ $\psi_{ce}+\psi_{ev}+\psi_{ac}:\psi_{ce}+\psi_{ac}:\psi_{cv}+\psi_{ce}:\psi_{ev}$ | 1.05232 | 0.87918 | 7083.19 |
| 121 | DisplMean~ $\psi_{ce}+\psi_{ev}+\psi_{ac}:\psi_{ce}+\psi_{ac}:\psi_{cv}$ | 1.05232 | 0.87889 | 7154.16 |
| 122 | DisplMean~ $\psi_{ac}+\psi_{ce}+\psi_{cv}+\psi_{ac}:\psi_{ce}+\psi_{ev}:\psi_{cv}$ | 0.164391 | 0.87588 | 7902.73 |
| 123 | DisplMean~ $\psi_{ac}+\psi_{ce}+\psi_{cv}+\psi_{ac}:\psi_{ce}+\psi_{ac}:\psi_{cv}+\psi_{ce}:\psi_{ev}$ | 0.172972 | 0.87461 | 8213.61 |
| 124 | DisplMean~ $\psi_{ac}+\psi_{ce}+\psi_{ac}:\psi_{ce}+\psi_{ce}:\psi_{ev}+\psi_{ev}:\psi_{cv}$ | 0.165456 | 0.87381 | 8408.29 |
| 125 | DisplMean~ $\psi_{ac}+\psi_{ce}+\psi_{ac}:\psi_{ce}+\psi_{ce}:\psi_{ev}+\psi_{ce}:\psi_{cv}$ | 0.140329 | 0.86888 | 9573.96 |
| 126 | DisplMean~ $\psi_{ac}+\psi_{ce}+\psi_{cv}+\psi_{ac}:\psi_{ce}+\psi_{ce}:\psi_{ev}$ | 0.152196 | 0.86569 | 10306.3 |
| 127 | DisplMean~ $\psi_{ev}+\psi_{cv}+\psi_{ac}:\psi_{ce}+\psi_{ce}:\psi_{ev}+\psi_{ce}:\psi_{cv}+\psi_{ev}:\psi_{cv}$ | 1.01734 | 0.8651 | 10442.4 |
| 128 | DisplMean~ $\psi_{ev}+\psi_{cv}+\psi_{ac}:\psi_{ce}+\psi_{ce}:\psi_{ev}+\psi_{ce}:\psi_{cv}$ | 1.01735 | 0.86508 | 10446.2 |
| 129 | DisplMean~ $\psi_{ac}+\psi_{ce}+\psi_{ac}:\psi_{ce}+\psi_{ce}:\psi_{ev}$ | 0.148946 | 0.86502 | 10458.6 |
| 130 | DisplMean~ $\psi_{ac}+\psi_{ce}+\psi_{ac}:\psi_{ce}+\psi_{ac}:\psi_{cv}+\psi_{ce}:\psi_{ev}$ | 0.149151 | 0.86502 | 10458.7 |
| 131 | DisplMean~ $\psi_{ce}+\psi_{ev}+\psi_{cv}+\psi_{ac}:\psi_{ce}+\psi_{ce}:\psi_{ev}+\psi_{ev}:\psi_{cv}$ | 0.955735 | 0.86241 | 11043.4 |

| | | | | |
|---|---|---|---|---|
| 132 | DisplMean~ $\psi_{ce}$+$\psi_{ev}$+$\psi_{cv}$+$\psi_{ac}$:$\psi_{ce}$+$\psi_{ce}$:$\psi_{ev}$ | 0.955736 | 0.8624 | 11045.1 |
| 133 | DisplMean~ $\psi_{ce}$+$\psi_{ev}$+$\psi_{cv}$+$\psi_{ac}$:$\psi_{ce}$+$\psi_{ev}$:$\psi_{cv}$ | 0.95575 | 0.86218 | 11092.9 |
| 134 | DisplMean~ $\psi_{ce}$+$\psi_{ev}$+$\psi_{cv}$+$\psi_{ac}$:$\psi_{ce}$ | 0.95575 | 0.86216 | 11095.5 |
| 135 | DisplMean~ $\psi_{ce}$+$\psi_{ev}$+$\psi_{ac}$:$\psi_{ce}$+$\psi_{ce}$:$\psi_{ev}$+$\psi_{ev}$:$\psi_{cv}$ | 0.944024 | 0.85976 | 11623.1 |
| 136 | DisplMean~ $\psi_{ce}$+$\psi_{ev}$+$\psi_{ac}$:$\psi_{ce}$+$\psi_{ce}$:$\psi_{ev}$ | 0.943358 | 0.85973 | 11629.5 |
| 137 | DisplMean~ $\psi_{ce}$+$\psi_{ev}$+$\psi_{ac}$:$\psi_{ce}$+$\psi_{ev}$:$\psi_{cv}$ | 0.943979 | 0.85952 | 11673.4 |
| 138 | DisplMean~ $\psi_{ce}$+$\psi_{ev}$+$\psi_{ac}$:$\psi_{ce}$ | 0.943365 | 0.8595 | 11678.4 |
| 139 | DisplMean~ $\psi_{ce}$+$\psi_{ac}$:$\psi_{ce}$+$\psi_{ac}$:$\psi_{ev}$+$\psi_{ac}$:$\psi_{cv}$ | 1.19059 | 0.85604 | 12418.8 |
| 140 | DisplMean~ $\psi_{ce}$+$\psi_{cv}$+$\psi_{ac}$:$\psi_{ce}$+$\psi_{ac}$:$\psi_{ev}$+$\psi_{ce}$:$\psi_{cv}$ | 1.13022 | 0.83624 | 16345.6 |
| 141 | DisplMean~ $\psi_{ce}$+$\psi_{ac}$:$\psi_{ce}$+$\psi_{ac}$:$\psi_{ev}$+$\psi_{ce}$:$\psi_{cv}$ | 1.13196 | 0.83523 | 16531.7 |
| 142 | DisplMean~ $\psi_{ac}$+$\psi_{ce}$+$\psi_{ac}$:$\psi_{ce}$+$\psi_{ev}$:$\psi_{cv}$ | 0.181041 | 0.83313 | 16917.5 |
| 143 | DisplMean~ $\psi_{ce}$+$\psi_{ev}$+$\psi_{cv}$+$\psi_{ac}$:$\psi_{ce}$+$\psi_{ac}$:$\psi_{ev}$+$\psi_{ac}$:$\psi_{cv}$+$\psi_{ce}$:$\psi_{ev}$+$\psi_{ce}$:$\psi_{cv}$+$\psi_{ev}$:$\psi_{cv}$ | 0.206842 | 0.82865 | 17730.4 |
| 144 | DisplMean~ $\psi_{ce}$+$\psi_{ev}$+$\psi_{cv}$+$\psi_{ac}$:$\psi_{ce}$+$\psi_{ac}$:$\psi_{ev}$+$\psi_{ac}$:$\psi_{cv}$+$\psi_{ce}$:$\psi_{cv}$+$\psi_{ev}$:$\psi_{cv}$ | 0.206747 | 0.82659 | 18094.2 |
| 145 | DisplMean~ $\psi_{ce}$+$\psi_{ac}$:$\psi_{ce}$+$\psi_{ac}$:$\psi_{cv}$+$\psi_{ce}$:$\psi_{ev}$ | 1.15105 | 0.82277 | 18753.2 |
| 146 | DisplMean~ $\psi_{ce}$+$\psi_{cv}$+$\psi_{ac}$:$\psi_{ce}$+$\psi_{ac}$:$\psi_{ev}$ | 1.14293 | 0.82086 | 19080.2 |
| 147 | DisplMean~ $\psi_{ce}$+$\psi_{ev}$+$\psi_{cv}$+$\psi_{ac}$:$\psi_{ce}$+$\psi_{ac}$:$\psi_{ev}$+$\psi_{ac}$:$\psi_{cv}$+$\psi_{ce}$:$\psi_{ev}$+$\psi_{ce}$:$\psi_{cv}$ | 0.203416 | 0.82012 | 19209.7 |
| 148 | DisplMean~ $\psi_{ce}$+$\psi_{ev}$+$\psi_{cv}$+$\psi_{ac}$:$\psi_{ce}$+$\psi_{ac}$:$\psi_{ev}$+$\psi_{ac}$:$\psi_{cv}$+$\psi_{ce}$:$\psi_{cv}$ | 0.203212 | 0.81861 | 19463.3 |
| 149 | DisplMean~ $\psi_{ce}$+$\psi_{ev}$+$\psi_{cv}$+$\psi_{ac}$:$\psi_{ce}$+$\psi_{ac}$:$\psi_{ev}$+$\psi_{ac}$:$\psi_{cv}$+$\psi_{ce}$:$\psi_{ev}$+$\psi_{ev}$:$\psi_{cv}$ | 0.2074 | 0.81789 | 19583.8 |
| 150 | DisplMean~ $\psi_{ce}$+$\psi_{ev}$+$\psi_{cv}$+$\psi_{ac}$:$\psi_{ce}$+$\psi_{ac}$:$\psi_{ev}$+$\psi_{ac}$:$\psi_{cv}$+$\psi_{ev}$:$\psi_{cv}$ | 0.207075 | 0.81603 | 19893.7 |
| 151 | DisplMean~ $\psi_{ce}$+$\psi_{ev}$+$\psi_{cv}$+$\psi_{ac}$:$\psi_{ce}$+$\psi_{ac}$:$\psi_{ev}$+$\psi_{ac}$:$\psi_{cv}$+$\psi_{ce}$:$\psi_{ev}$ | 0.203572 | 0.8102 | 20844.2 |
| 152 | DisplMean~ $\psi_{ce}$+$\psi_{ac}$:$\psi_{ce}$+$\psi_{ac}$:$\psi_{ev}$ | 1.12828 | 0.80902 | 21027.5 |
| 153 | DisplMean~ $\psi_{ce}$+$\psi_{ev}$+$\psi_{cv}$+$\psi_{ac}$:$\psi_{ce}$+$\psi_{ac}$:$\psi_{ev}$+$\psi_{ac}$:$\psi_{cv}$ | 0.203239 | 0.80882 | 21062.6 |
| 154 | DisplMean~ $\psi_{ce}$+$\psi_{cv}$+$\psi_{ac}$:$\psi_{ce}$+$\psi_{ce}$:$\psi_{ev}$+$\psi_{ce}$:$\psi_{cv}$+$\psi_{ev}$:$\psi_{cv}$ | 1.03724 | 0.80101 | 22282 |
| 155 | DisplMean~ $\psi_{ce}$+$\psi_{ac}$:$\psi_{ce}$+$\psi_{ce}$:$\psi_{ev}$+$\psi_{ce}$:$\psi_{cv}$+$\psi_{ev}$:$\psi_{cv}$ | 1.03587 | 0.79862 | 22645.1 |
| 156 | DisplMean~ $\psi_{ac}$+$\psi_{ce}$+$\psi_{cv}$+$\psi_{ac}$:$\psi_{ce}$+$\psi_{ac}$:$\psi_{cv}$+$\psi_{ce}$:$\psi_{cv}$ | 0.136707 | 0.79206 | 23622.7 |
| 157 | DisplMean~ $\psi_{ac}$+$\psi_{ce}$+$\psi_{cv}$+$\psi_{ac}$:$\psi_{ce}$+$\psi_{ce}$:$\psi_{cv}$ | 0.120533 | 0.78963 | 23975.6 |
| 158 | DisplMean~ $\psi_{ce}$+$\psi_{cv}$+$\psi_{ac}$:$\psi_{ce}$+$\psi_{ce}$:$\psi_{ev}$+$\psi_{ev}$:$\psi_{cv}$ | 1.05438 | 0.78736 | 24303.2 |

| | | | | |
|---|---|---|---|---|
| 159 | DisplMean~ $\psi_{ce}+\psi_{ac}:\psi_{ce}+\psi_{ce}:\psi_{ev}+\psi_{ev}:\psi_{cv}$ | 1.05509 | 0.78686 | 24373.2 |
| 160 | DisplMean~ $\psi_{ce}+\psi_{ev}+\psi_{ac}:\psi_{ce}+\psi_{ac}:\psi_{ev}+\psi_{ac}:\psi_{cv}+\psi_{ce}:\psi_{ev}+\psi_{ce}:\psi_{cv}+\psi_{ev}:\psi_{cv}$ | 0.190833 | 0.78203 | 25060.7 |
| 161 | DisplMean~ $\psi_{ce}+\psi_{cv}+\psi_{ac}:\psi_{ce}+\psi_{ce}:\psi_{ev}+\psi_{ce}:\psi_{cv}$ | 1.02533 | 0.7816 | 25117.7 |
| 162 | DisplMean~ $\psi_{ce}+\psi_{ac}:\psi_{ce}+\psi_{ce}:\psi_{ev}+\psi_{ce}:\psi_{cv}$ | 1.0241 | 0.78152 | 25127.2 |
| 163 | DisplMean~ $\psi_{ce}+\psi_{ev}+\psi_{ac}:\psi_{ce}+\psi_{ac}:\psi_{ev}+\psi_{ac}:\psi_{cv}+\psi_{ce}:\psi_{ev}+\psi_{ev}:\psi_{cv}$ | 0.190901 | 0.77947 | 25414.9 |
| 164 | DisplMean~ $\psi_{ce}+\psi_{ev}+\psi_{ac}:\psi_{ce}+\psi_{ac}:\psi_{ev}+\psi_{ac}:\psi_{cv}+\psi_{ce}:\psi_{cv}+\psi_{ev}:\psi_{cv}$ | 0.191519 | 0.77734 | 25706.8 |
| 165 | DisplMean~ $\psi_{ac}+\psi_{ce}+\psi_{ac}:\psi_{ce}+\psi_{ac}:\psi_{cv}+\psi_{ce}:\psi_{cv}$ | 0.107603 | 0.77725 | 25717.5 |
| 166 | DisplMean~ $\psi_{ce}+\psi_{ev}+\psi_{ac}:\psi_{ce}+\psi_{ac}:\psi_{ev}+\psi_{ac}:\psi_{cv}+\psi_{ev}:\psi_{cv}$ | 0.19122 | 0.77512 | 26008.6 |
| 167 | DisplMean~ $\psi_{ac}+\psi_{ce}+\psi_{cv}+\psi_{ac}:\psi_{ce}+\psi_{ac}:\psi_{cv}$ | 0.169366 | 0.76751 | 27021.5 |
| 168 | DisplMean~ $\psi_{ce}+\psi_{cv}+\psi_{ac}:\psi_{ce}+\psi_{ce}:\psi_{ev}$ | 1.05013 | 0.76719 | 27062.9 |
| 169 | DisplMean~ $\psi_{ce}+\psi_{cv}+\psi_{ac}:\psi_{ce}+\psi_{ce}:\psi_{cv}+\psi_{ev}:\psi_{cv}$ | 1.05175 | 0.76564 | 27265.8 |
| 170 | DisplMean~ $\psi_{ac}+\psi_{ce}+\psi_{ac}:\psi_{ce}+\psi_{ce}:\psi_{cv}$ | 0.124994 | 0.76243 | 27678.9 |
| 171 | DisplMean~ $\psi_{ce}+\psi_{ac}:\psi_{ce}+\psi_{ce}:\psi_{cv}+\psi_{ev}:\psi_{cv}$ | 1.0279 | 0.75911 | 28101.8 |
| 172 | DisplMean~ $\psi_{ac}+\psi_{ce}+\psi_{cv}+\psi_{ac}:\psi_{ce}$ | 0.140697 | 0.75889 | 28129.1 |
| 173 | DisplMean~ $\psi_{ac}+\psi_{ce}+\psi_{ac}:\psi_{ce}$ | 0.136778 | 0.75829 | 28203.6 |
| 174 | DisplMean~ $\psi_{ac}+\psi_{ce}+\psi_{ac}:\psi_{ce}+\psi_{ac}:\psi_{cv}$ | 0.136966 | 0.75829 | 28205.1 |
| 175 | DisplMean~ $\psi_{ce}+\psi_{ac}:\psi_{ce}+\psi_{ce}:\psi_{ev}$ | 1.01711 | 0.75477 | 28644.3 |
| 176 | DisplMean~ $\psi_{ce}+\psi_{cv}+\psi_{ac}:\psi_{ce}+\psi_{ev}:\psi_{cv}$ | 1.0765 | 0.7537 | 28778.7 |
| 177 | DisplMean~ $\psi_{ce}+\psi_{cv}+\psi_{ac}:\psi_{ce}+\psi_{ce}:\psi_{cv}$ | 0.933243 | 0.74042 | 30377.5 |
| 178 | DisplMean~ $\psi_{ce}+\psi_{ev}+\psi_{cv}+\psi_{ac}:\psi_{ce}+\psi_{ac}:\psi_{ev}+\psi_{ce}:\psi_{ev}+\psi_{ce}:\psi_{cv}+\psi_{ev}:\psi_{cv}$ | 0.207474 | 0.74018 | 30409.8 |
| 179 | DisplMean~ $\psi_{ce}+\psi_{ac}:\psi_{ce}+\psi_{ce}:\psi_{cv}$ | 0.934987 | 0.74011 | 30412.9 |
| 180 | DisplMean~ $\psi_{ce}+\psi_{ev}+\psi_{ac}:\psi_{ce}+\psi_{ac}:\psi_{ev}+\psi_{ce}:\psi_{ev}+\psi_{ce}:\psi_{cv}+\psi_{ev}:\psi_{cv}$ | 0.208076 | 0.74005 | 30424.1 |
| 181 | DisplMean~ $\psi_{ce}+\psi_{cv}+\psi_{ac}:\psi_{ce}+\psi_{ac}:\psi_{ev}+\psi_{ac}:\psi_{cv}+\psi_{ce}:\psi_{ev}+\psi_{ce}:\psi_{cv}+\psi_{ev}:\psi_{cv}$ | 0.144457 | 0.73902 | 30545.5 |
| 182 | DisplMean~ $\psi_{ce}+\psi_{ev}+\psi_{cv}+\psi_{ac}:\psi_{ce}+\psi_{ac}:\psi_{ev}+\psi_{ce}:\psi_{cv}+\psi_{ev}:\psi_{cv}$ | 0.210724 | 0.73427 | 31093.7 |
| 183 | DisplMean~ $\psi_{ce}+\psi_{ev}+\psi_{ac}:\psi_{ce}+\psi_{ac}:\psi_{ev}+\psi_{ce}:\psi_{cv}+\psi_{ev}:\psi_{cv}$ | 0.211138 | 0.73421 | 31100.4 |
| 184 | DisplMean~ $\psi_{ce}+\psi_{cv}+\psi_{ac}:\psi_{ce}+\psi_{ac}:\psi_{ev}+\psi_{ac}:\psi_{cv}+\psi_{ce}:\psi_{ev}+\psi_{ev}:\psi_{cv}$ | 0.146056 | 0.73394 | 31131.9 |
| 185 | DisplMean~ $\psi_{ac}+\psi_{ev}+\psi_{cv}+\psi_{ac}:\psi_{ce}+\psi_{ac}:\psi_{ev}+\psi_{ac}:\psi_{cv}+\psi_{ce}:\psi_{ev}+\psi_{ce}:\psi_{cv}+\psi_{ev}:\psi_{cv}$ | 0.279858 | 0.73324 | 31214.3 |

| | | | | |
|---|---|---|---|---|
| 186 | DisplMean~ $\psi_{ac}+\psi_{ev}+\psi_{cv}+\psi_{ac}:\psi_{ce}+\psi_{ac}:\psi_{ev}+\psi_{ac}:\psi_{cv}+\psi_{ce}:\psi_{ev}+\psi_{ce}:\psi_{cv}$ | 0.280694 | 0.7331 | 31229.2 |
| 187 | DisplMean~ $\psi_{ac}+\psi_{ev}+\psi_{cv}+\psi_{ac}:\psi_{ce}+\psi_{ac}:\psi_{cv}+\psi_{ce}:\psi_{ev}+\psi_{ce}:\psi_{cv}+\psi_{ev}:\psi_{cv}$ | 0.283154 | 0.73261 | 31285.1 |
| 188 | DisplMean~ $\psi_{ac}+\psi_{ev}+\psi_{cv}+\psi_{ac}:\psi_{ce}+\psi_{ac}:\psi_{cv}+\psi_{ce}:\psi_{ev}+\psi_{ce}:\psi_{cv}$ | 0.283235 | 0.7326 | 31284.7 |
| 189 | DisplMean~ $\psi_{ce}+\psi_{ev}+\psi_{cv}+\psi_{ac}:\psi_{ce}+\psi_{ac}:\psi_{ev}+\psi_{ce}:\psi_{ev}+\psi_{ce}:\psi_{cv}$ | 0.217285 | 0.7203 | 32655.4 |
| 190 | DisplMean~ $\psi_{ce}+\psi_{ev}+\psi_{cv}+\psi_{ac}:\psi_{ce}+\psi_{ac}:\psi_{cv}+\psi_{ce}:\psi_{cv}$ | 0.145544 | 0.71799 | 32904.3 |
| 191 | DisplMean~ $\psi_{ce}+\psi_{ev}+\psi_{cv}+\psi_{ac}:\psi_{ce}+\psi_{ac}:\psi_{cv}+\psi_{ce}:\psi_{cv}+\psi_{ev}:\psi_{cv}$ | 0.145545 | 0.71799 | 32905.3 |
| 192 | DisplMean~ $\psi_{ce}+\psi_{ev}+\psi_{cv}+\psi_{ac}:\psi_{ce}+\psi_{ac}:\psi_{cv}+\psi_{ce}:\psi_{ev}+\psi_{ce}:\psi_{cv}$ | 0.145542 | 0.71798 | 32906.3 |
| 193 | DisplMean~ $\psi_{ce}+\psi_{ev}+\psi_{cv}+\psi_{ac}:\psi_{ce}+\psi_{ac}:\psi_{cv}+\psi_{ce}:\psi_{ev}+\psi_{ce}:\psi_{cv}+\psi_{ev}:\psi_{cv}$ | 0.145542 | 0.71798 | 32907.3 |
| 194 | DisplMean~ $\psi_{ce}+\psi_{ev}+\psi_{cv}+\psi_{ac}:\psi_{ce}+\psi_{ac}:\psi_{ev}+\psi_{ce}:\psi_{ev}+\psi_{ev}:\psi_{cv}$ | 0.206698 | 0.71784 | 32922.2 |
| 195 | DisplMean~ $\psi_{ac}+\psi_{cv}+\psi_{ac}:\psi_{ce}+\psi_{ac}:\psi_{ev}+\psi_{ac}:\psi_{cv}+\psi_{ce}:\psi_{ev}+\psi_{ce}:\psi_{cv}+\psi_{ev}:\psi_{cv}$ | 0.268603 | 0.71624 | 33095.2 |
| 196 | DisplMean~ $\psi_{ac}+\psi_{cv}+\psi_{ac}:\psi_{ce}+\psi_{ac}:\psi_{ev}+\psi_{ac}:\psi_{cv}+\psi_{ce}:\psi_{ev}+\psi_{ce}:\psi_{cv}$ | 0.268369 | 0.71614 | 33104.9 |
| 197 | DisplMean~ $\psi_{ce}+\psi_{cv}+\psi_{ac}:\psi_{ce}$ | 0.955704 | 0.71587 | 33129.8 |
| 198 | DisplMean~ $\psi_{ce}+\psi_{ev}+\psi_{cv}+\psi_{ac}:\psi_{ce}+\psi_{ac}:\psi_{cv}$ | 0.145951 | 0.71578 | 33140.9 |
| 199 | DisplMean~ $\psi_{ce}+\psi_{ev}+\psi_{cv}+\psi_{ac}:\psi_{ce}+\psi_{ac}:\psi_{cv}+\psi_{ev}:\psi_{cv}$ | 0.145952 | 0.71578 | 33142 |
| 200 | DisplMean~ $\psi_{ce}+\psi_{ev}+\psi_{cv}+\psi_{ac}:\psi_{ce}+\psi_{ac}:\psi_{cv}+\psi_{ce}:\psi_{ev}$ | 0.145948 | 0.71577 | 33142.9 |
| 201 | DisplMean~ $\psi_{ce}+\psi_{ev}+\psi_{cv}+\psi_{ac}:\psi_{ce}+\psi_{ac}:\psi_{cv}+\psi_{ce}:\psi_{ev}+\psi_{ev}:\psi_{cv}$ | 0.145948 | 0.71577 | 33143.9 |
| 202 | DisplMean~ $\psi_{ce}+\psi_{ev}+\psi_{cv}+\psi_{ac}:\psi_{ce}+\psi_{ac}:\psi_{ev}+\psi_{ce}:\psi_{cv}$ | 0.21943 | 0.71555 | 33167.2 |
| 203 | DisplMean~ $\psi_{ce}+\psi_{ac}:\psi_{ce}$ | 0.943263 | 0.71324 | 33409.8 |
| 204 | DisplMean~ $\psi_{ce}+\psi_{ev}+\psi_{cv}+\psi_{ac}:\psi_{ce}+\psi_{ac}:\psi_{ev}+\psi_{ev}:\psi_{cv}$ | 0.208584 | 0.71206 | 33538.3 |
| 205 | DisplMean~ $\psi_{ce}+\psi_{cv}+\psi_{ac}:\psi_{ce}+\psi_{ac}:\psi_{ev}+\psi_{ac}:\psi_{cv}+\psi_{ce}:\psi_{cv}+\psi_{ev}:\psi_{cv}$ | 0.142041 | 0.7093 | 33829.6 |
| 206 | DisplMean~ $\psi_{ev}+\psi_{cv}+\psi_{ac}:\psi_{ce}+\psi_{ac}:\psi_{ev}+\psi_{ac}:\psi_{cv}+\psi_{ce}:\psi_{ev}+\psi_{ce}:\psi_{cv}+\psi_{ev}:\psi_{cv}$ | 0.227693 | 0.70825 | 33940.7 |
| 207 | DisplMean~ $\psi_{ev}+\psi_{cv}+\psi_{ac}:\psi_{ce}+\psi_{ac}:\psi_{ev}+\psi_{ac}:\psi_{cv}+\psi_{ce}:\psi_{ev}+\psi_{ce}:\psi_{cv}$ | 0.224948 | 0.7072 | 34049.1 |
| 208 | DisplMean~ $\psi_{ce}+\psi_{cv}+\psi_{ac}:\psi_{ce}+\psi_{ac}:\psi_{cv}+\psi_{ce}:\psi_{ev}+\psi_{ce}:\psi_{cv}+\psi_{ev}:\psi_{cv}$ | 0.141705 | 0.70633 | 34139.2 |
| 209 | DisplMean~ $\psi_{ce}+\psi_{cv}+\psi_{ac}:\psi_{ce}+\psi_{ac}:\psi_{ev}+\psi_{ac}:\psi_{cv}+\psi_{ev}:\psi_{cv}$ | 0.142869 | 0.70585 | 34188.1 |
| 210 | DisplMean~ $\psi_{ce}+\psi_{ev}+\psi_{ac}:\psi_{ce}+\psi_{ac}:\psi_{ev}+\psi_{ce}:\psi_{ev}+\psi_{ev}:\psi_{cv}$ | 0.216765 | 0.70458 | 34319.9 |
| 211 | DisplMean~ $\psi_{ce}+\psi_{cv}+\psi_{ac}:\psi_{ce}+\psi_{ac}:\psi_{cv}+\psi_{ce}:\psi_{ev}+\psi_{ev}:\psi_{cv}$ | 0.142136 | 0.70412 | 34366.8 |
| 212 | DisplMean~ $\psi_{ce}+\psi_{ev}+\psi_{ac}:\psi_{ce}+\psi_{ac}:\psi_{ev}+\psi_{ac}:\psi_{cv}+\psi_{ce}:\psi_{ev}+\psi_{ce}:\psi_{cv}$ | 0.207028 | 0.70354 | 34427.3 |

| | | | | |
|---|---|---|---|---|
| 213 | DisplMean~ $\psi_{ce}+\psi_{ev}+\psi_{ac}:\psi_{ce}+\psi_{ac}:\psi_{ev}+\psi_{ac}:\psi_{cv}+\psi_{ce}:\psi_{ev}$ | 0.213709 | 0.70309 | 34473 |
| 214 | DisplMean~ $\psi_{ce}+\psi_{ev}+\psi_{ac}:\psi_{ce}+\psi_{ac}:\psi_{ev}+\psi_{ce}:\psi_{ev}+\psi_{ce}:\psi_{cv}$ | 0.220337 | 0.7005 | 34737.7 |
| 215 | DisplMean~ $\psi_{ce}+\psi_{ev}+\psi_{ac}:\psi_{ce}+\psi_{ac}:\psi_{ev}+\psi_{ev}:\psi_{cv}$ | 0.217452 | 0.69966 | 34822.2 |
| 216 | DisplMean~ $\psi_{ce}+\psi_{ev}+\psi_{ac}:\psi_{ce}+\psi_{ac}:\psi_{ev}+\psi_{ac}:\psi_{cv}+\psi_{ce}:\psi_{cv}$ | 0.207265 | 0.69931 | 34858.6 |
| 217 | DisplMean~ $\psi_{ce}+\psi_{ev}+\psi_{cv}+\psi_{ac}:\psi_{ce}+\psi_{ac}:\psi_{ev}+\psi_{ce}:\psi_{ev}$ | 0.212907 | 0.69886 | 34903.8 |
| 218 | DisplMean~ $\psi_{ce}+\psi_{ev}+\psi_{ac}:\psi_{ce}+\psi_{ac}:\psi_{ev}+\psi_{ac}:\psi_{cv}$ | 0.215493 | 0.69871 | 34917.9 |
| 219 | DisplMean~ $\psi_{ce}+\psi_{cv}+\psi_{ac}:\psi_{ce}+\psi_{ac}:\psi_{cv}+\psi_{ce}:\psi_{cv}+\psi_{ev}:\psi_{cv}$ | 0.141004 | 0.69869 | 34921.5 |
| 220 | DisplMean~ $\psi_{ce}+\psi_{ev}+\psi_{ac}:\psi_{ce}+\psi_{ac}:\psi_{ev}+\psi_{ce}:\psi_{ev}$ | 0.212771 | 0.6981 | 34979.5 |
| 221 | DisplMean~ $\psi_{ce}+\psi_{cv}+\psi_{ac}:\psi_{ce}+\psi_{ac}:\psi_{cv}+\psi_{ev}:\psi_{cv}$ | 0.141421 | 0.69646 | 35144.4 |
| 222 | DisplMean~ $\psi_{ce}+\psi_{ev}+\psi_{ac}:\psi_{ce}+\psi_{ac}:\psi_{ev}+\psi_{ce}:\psi_{cv}$ | 0.222513 | 0.69585 | 35206.2 |
| 223 | DisplMean~ $\psi_{ce}+\psi_{ev}+\psi_{cv}+\psi_{ac}:\psi_{ce}+\psi_{ac}:\psi_{ev}$ | 0.213872 | 0.6942 | 35370.9 |
| 224 | DisplMean~ $\psi_{ce}+\psi_{ev}+\psi_{ac}:\psi_{ce}+\psi_{ac}:\psi_{ev}$ | 0.213945 | 0.69344 | 35444.8 |
| 225 | DisplMean~ $\psi_{ac}+\psi_{ev}+\psi_{cv}+\psi_{ac}:\psi_{ce}+\psi_{ac}:\psi_{ev}+\psi_{ac}:\psi_{cv}+\psi_{ce}:\psi_{ev}+\psi_{ev}:\psi_{cv}$ | 0.326488 | 0.69332 | 35460.9 |
| 226 | DisplMean~ $\psi_{ac}+\psi_{ev}+\psi_{cv}+\psi_{ac}:\psi_{ce}+\psi_{ac}:\psi_{ev}+\psi_{ac}:\psi_{cv}+\psi_{ce}:\psi_{ev}$ | 0.32758 | 0.69313 | 35478.9 |
| 227 | DisplMean~ $\psi_{ac}+\psi_{ev}+\psi_{cv}+\psi_{ac}:\psi_{ce}+\psi_{ac}:\psi_{cv}+\psi_{ce}:\psi_{ev}+\psi_{ev}:\psi_{cv}$ | 0.330792 | 0.69242 | 35549.7 |
| 228 | DisplMean~ $\psi_{ac}+\psi_{ev}+\psi_{cv}+\psi_{ac}:\psi_{ce}+\psi_{ac}:\psi_{cv}+\psi_{ce}:\psi_{ev}$ | 0.330896 | 0.69241 | 35549.4 |
| 229 | DisplMean~ $\psi_{ce}+\psi_{cv}+\psi_{ac}:\psi_{ce}+\psi_{ac}:\psi_{ev}+\psi_{ac}:\psi_{cv}+\psi_{ce}:\psi_{ev}+\psi_{ce}:\psi_{cv}$ | 0.136699 | 0.69227 | 35564.1 |
| 230 | DisplMean~ $\psi_{ac}+\psi_{ev}+\psi_{cv}+\psi_{ac}:\psi_{ce}+\psi_{ac}:\psi_{ev}+\psi_{ac}:\psi_{cv}+\psi_{ce}:\psi_{cv}+\psi_{ev}:\psi_{cv}$ | 0.335734 | 0.69109 | 35681.7 |
| 231 | DisplMean~ $\psi_{ac}+\psi_{ev}+\psi_{cv}+\psi_{ac}:\psi_{ce}+\psi_{ac}:\psi_{ev}+\psi_{ac}:\psi_{cv}+\psi_{ce}:\psi_{cv}$ | 0.335573 | 0.69107 | 35683 |
| 232 | DisplMean~ $\psi_{ac}+\psi_{ev}+\psi_{cv}+\psi_{ac}:\psi_{ce}+\psi_{ac}:\psi_{cv}+\psi_{ce}:\psi_{cv}$ | 0.335038 | 0.69102 | 35686.4 |
| 233 | DisplMean~ $\psi_{ac}+\psi_{ev}+\psi_{cv}+\psi_{ac}:\psi_{ce}+\psi_{ac}:\psi_{cv}+\psi_{ce}:\psi_{cv}+\psi_{ev}:\psi_{cv}$ | 0.335048 | 0.69102 | 35687.5 |
| 234 | DisplMean~ $\psi_{ce}+\psi_{cv}+\psi_{ac}:\psi_{ce}+\psi_{ac}:\psi_{cv}+\psi_{ce}:\psi_{ev}+\psi_{ce}:\psi_{cv}$ | 0.137287 | 0.69096 | 35693.1 |
| 235 | DisplMean~ $\psi_{ac}+\psi_{ce}+\psi_{ev}+\psi_{cv}+\psi_{ac}:\psi_{ev}+\psi_{ac}:\psi_{cv}+\psi_{ev}:\psi_{cv}$ | 0.919596 | 0.69001 | 35786.7 |
| 236 | DisplMean~ $\psi_{ac}+\psi_{ce}+\psi_{ev}+\psi_{cv}+\psi_{ac}:\psi_{ev}+\psi_{ac}:\psi_{cv}+\psi_{ce}:\psi_{cv}+\psi_{ev}:\psi_{cv}$ | 0.915956 | 0.69001 | 35788.3 |
| 237 | DisplMean~ $\psi_{ac}+\psi_{ce}+\psi_{ev}+\psi_{cv}+\psi_{ac}:\psi_{ev}+\psi_{ac}:\psi_{cv}+\psi_{ce}:\psi_{ev}+\psi_{ev}:\psi_{cv}$ | 0.919581 | 0.69 | 35788.7 |
| 238 | DisplMean~ $\psi_{ac}+\psi_{ce}+\psi_{ev}+\psi_{cv}+\psi_{ac}:\psi_{ev}+\psi_{ac}:\psi_{cv}+\psi_{ce}:\psi_{ev}+\psi_{ce}:\psi_{cv}+\psi_{ev}:\psi_{cv}$ | 0.91594 | 0.69 | 35790.3 |
| 239 | DisplMean~ $\psi_{ac}+\psi_{ce}+\psi_{ev}+\psi_{cv}+\psi_{ac}:\psi_{ev}+\psi_{ac}:\psi_{cv}$ | 0.917082 | 0.68998 | 35788.7 |

| | | | | |
|---|---|---|---|---|
| 240 | DisplMean~ $\psi_{ac}+\psi_{ce}+\psi_{ev}+\psi_{cv}+\psi_{ac}:\psi_{ev}+\psi_{ac}:\psi_{cv}+\psi_{ce}:\psi_{cv}$ | 0.91342 | 0.68998 | 35790.3 |
| 241 | DisplMean~ $\psi_{ac}+\psi_{ce}+\psi_{ev}+\psi_{cv}+\psi_{ac}:\psi_{ev}+\psi_{ac}:\psi_{cv}+\psi_{ce}:\psi_{ev}$ | 0.917265 | 0.68997 | 35790.6 |
| 242 | DisplMean~ $\psi_{ac}+\psi_{ce}+\psi_{ev}+\psi_{cv}+\psi_{ac}:\psi_{ev}+\psi_{ac}:\psi_{cv}+\psi_{ce}:\psi_{ev}+\psi_{ce}:\psi_{cv}$ | 0.913603 | 0.68997 | 35792.3 |
| 243 | DisplMean~ $\psi_{ac}+\psi_{ce}+\psi_{ev}+\psi_{cv}+\psi_{ac}:\psi_{cv}$ | 0.907405 | 0.68991 | 35795.2 |
| 244 | DisplMean~ $\psi_{ac}+\psi_{ce}+\psi_{ev}+\psi_{cv}+\psi_{ac}:\psi_{cv}+\psi_{ev}:\psi_{cv}$ | 0.90758 | 0.68991 | 35796.3 |
| 245 | DisplMean~ $\psi_{ac}+\psi_{ce}+\psi_{ev}+\psi_{cv}+\psi_{ac}:\psi_{cv}+\psi_{ce}:\psi_{ev}$ | 0.908477 | 0.6899 | 35796.8 |
| 246 | DisplMean~ $\psi_{ac}+\psi_{ce}+\psi_{ev}+\psi_{cv}+\psi_{ac}:\psi_{cv}+\psi_{ce}:\psi_{cv}$ | 0.903687 | 0.6899 | 35796.8 |
| 247 | DisplMean~ $\psi_{ac}+\psi_{ce}+\psi_{ev}+\psi_{cv}+\psi_{ac}:\psi_{cv}+\psi_{ce}:\psi_{ev}+\psi_{ce}:\psi_{cv}$ | 0.904765 | 0.6899 | 35798.4 |
| 248 | DisplMean~ $\psi_{ac}+\psi_{ce}+\psi_{ev}+\psi_{cv}+\psi_{ac}:\psi_{cv}+\psi_{ce}:\psi_{ev}+\psi_{ev}:\psi_{cv}$ | 0.908604 | 0.6899 | 35797.9 |
| 249 | DisplMean~ $\psi_{ac}+\psi_{ce}+\psi_{ev}+\psi_{cv}+\psi_{ac}:\psi_{cv}+\psi_{ce}:\psi_{cv}+\psi_{ev}:\psi_{cv}$ | 0.903866 | 0.6899 | 35797.9 |
| 250 | DisplMean~ $\psi_{ac}+\psi_{ce}+\psi_{ev}+\psi_{cv}+\psi_{ac}:\psi_{cv}+\psi_{ce}:\psi_{ev}+\psi_{ce}:\psi_{cv}+\psi_{ev}:\psi_{cv}$ | 0.904895 | 0.68989 | 35799.5 |
| 251 | DisplMean~ $\psi_{ce}+\psi_{cv}+\psi_{ac}:\psi_{ce}+\psi_{ac}:\psi_{ev}+\psi_{ac}:\psi_{cv}+\psi_{ce}:\psi_{ev}$ | 0.137354 | 0.68976 | 35811.2 |
| 252 | DisplMean~ $\psi_{ce}+\psi_{cv}+\psi_{ac}:\psi_{ce}+\psi_{ac}:\psi_{cv}+\psi_{ce}:\psi_{ev}$ | 0.137743 | 0.68876 | 35908.1 |
| 253 | DisplMean~ $\psi_{ce}+\psi_{ac}:\psi_{ce}+\psi_{ac}:\psi_{ev}+\psi_{ac}:\psi_{cv}+\psi_{ce}:\psi_{ev}+\psi_{ce}:\psi_{cv}+\psi_{ev}:\psi_{cv}$ | 0.124668 | 0.68805 | 35978.7 |
| 254 | DisplMean~ $\psi_{ce}+\psi_{ac}:\psi_{ce}+\psi_{ac}:\psi_{ev}+\psi_{ac}:\psi_{cv}+\psi_{ce}:\psi_{ev}+\psi_{ev}:\psi_{cv}$ | 0.125336 | 0.6879 | 35992.7 |
| 255 | DisplMean~ $\psi_{ac}+\psi_{cv}+\psi_{ac}:\psi_{ce}+\psi_{ac}:\psi_{ev}+\psi_{ac}:\psi_{cv}+\psi_{ce}:\psi_{cv}+\psi_{ev}:\psi_{cv}$ | 0.307398 | 0.68327 | 36442.5 |
| 256 | DisplMean~ $\psi_{ac}+\psi_{cv}+\psi_{ac}:\psi_{ce}+\psi_{ac}:\psi_{ev}+\psi_{ac}:\psi_{cv}+\psi_{ce}:\psi_{cv}$ | 0.306852 | 0.6829 | 36476.6 |
| 257 | DisplMean~ $\psi_{ev}+\psi_{cv}+\psi_{ac}:\psi_{ce}+\psi_{ac}:\psi_{cv}+\psi_{ce}:\psi_{ev}+\psi_{ce}:\psi_{cv}$ | 0.178954 | 0.68228 | 36536.5 |
| 258 | DisplMean~ $\psi_{ev}+\psi_{cv}+\psi_{ac}:\psi_{ce}+\psi_{ac}:\psi_{cv}+\psi_{ce}:\psi_{ev}+\psi_{ce}:\psi_{cv}+\psi_{ev}:\psi_{cv}$ | 0.178944 | 0.68227 | 36538.3 |
| 259 | DisplMean~ $\psi_{ev}+\psi_{cv}+\psi_{ac}:\psi_{ce}+\psi_{ac}:\psi_{ev}+\psi_{ac}:\psi_{cv}+\psi_{ce}:\psi_{cv}+\psi_{ev}:\psi_{cv}$ | 0.262722 | 0.68192 | 36571.7 |
| 260 | DisplMean~ $\psi_{ev}+\psi_{cv}+\psi_{ac}:\psi_{ce}+\psi_{ac}:\psi_{ev}+\psi_{ac}:\psi_{cv}+\psi_{ce}:\psi_{cv}$ | 0.258698 | 0.681 | 36658.8 |
| 261 | DisplMean~ $\psi_{ac}+\psi_{ev}+\psi_{cv}+\psi_{ac}:\psi_{ev}+\psi_{ac}:\psi_{cv}+\psi_{ce}:\psi_{ev}+\psi_{ce}:\psi_{cv}$ | 0.465337 | 0.67905 | 36846 |
| 262 | DisplMean~ $\psi_{ac}+\psi_{ev}+\psi_{cv}+\psi_{ac}:\psi_{ev}+\psi_{ac}:\psi_{cv}+\psi_{ce}:\psi_{ev}+\psi_{ce}:\psi_{cv}+\psi_{ev}:\psi_{cv}$ | 0.465345 | 0.67904 | 36847.9 |
| 263 | DisplMean~ $\psi_{ac}+\psi_{ev}+\psi_{cv}+\psi_{ac}:\psi_{cv}+\psi_{ce}:\psi_{ev}+\psi_{ce}:\psi_{cv}$ | 0.465358 | 0.67903 | 36846.7 |
| 264 | DisplMean~ $\psi_{ac}+\psi_{ev}+\psi_{cv}+\psi_{ac}:\psi_{cv}+\psi_{ce}:\psi_{ev}+\psi_{ce}:\psi_{cv}+\psi_{ev}:\psi_{cv}$ | 0.465352 | 0.67902 | 36848.6 |
| 265 | DisplMean~ $\psi_{ev}+\psi_{cv}+\psi_{ac}:\psi_{ce}+\psi_{ac}:\psi_{ev}+\psi_{ac}:\psi_{cv}+\psi_{ce}:\psi_{ev}+\psi_{ev}:\psi_{cv}$ | 0.242464 | 0.67787 | 36957.3 |
| 266 | DisplMean~ $\psi_{ev}+\psi_{cv}+\psi_{ac}:\psi_{ce}+\psi_{ac}:\psi_{ev}+\psi_{ac}:\psi_{cv}+\psi_{ce}:\psi_{ev}$ | 0.239902 | 0.6775 | 36991.5 |

| | | | | |
|---|---|---|---|---|
| 267 | DisplMean~ $\psi_{ac}+\psi_{ce}+\psi_{cv}+\psi_{ac}:\psi_{ev}+\psi_{ac}:\psi_{cv}+\psi_{ce}:\psi_{ev}+\psi_{ce}:\psi_{cv}+\psi_{ev}:\psi_{cv}$ | 0.43141 | 0.67739 | 37003.7 |
| 268 | DisplMean~ $\psi_{ac}+\psi_{ce}+\psi_{cv}+\psi_{ac}:\psi_{ev}+\psi_{ac}:\psi_{cv}+\psi_{ce}:\psi_{ev}+\psi_{ev}:\psi_{cv}$ | 0.433574 | 0.67729 | 37011.8 |
| 269 | DisplMean~ $\psi_{ac}+\psi_{ce}+\psi_{cv}+\psi_{ac}:\psi_{ev}+\psi_{ac}:\psi_{cv}+\psi_{ce}:\psi_{ev}+\psi_{ce}:\psi_{cv}$ | 0.430389 | 0.67701 | 37038.2 |
| 270 | DisplMean~ $\psi_{ac}+\psi_{ce}+\psi_{cv}+\psi_{ac}:\psi_{ev}+\psi_{ac}:\psi_{cv}+\psi_{ce}:\psi_{cv}+\psi_{ev}:\psi_{cv}$ | 0.431595 | 0.67698 | 37041.8 |
| 271 | DisplMean~ $\psi_{ac}+\psi_{ce}+\psi_{cv}+\psi_{ac}:\psi_{ev}+\psi_{ac}:\psi_{cv}+\psi_{ce}:\psi_{ev}$ | 0.432566 | 0.67692 | 37046.3 |
| 272 | DisplMean~ $\psi_{ac}+\psi_{ce}+\psi_{cv}+\psi_{ac}:\psi_{ev}+\psi_{ac}:\psi_{cv}+\psi_{ev}:\psi_{cv}$ | 0.433762 | 0.67688 | 37049.9 |
| 273 | DisplMean~ $\psi_{ce}+\psi_{ev}+\psi_{cv}+\psi_{ac}:\psi_{ev}+\psi_{ac}:\psi_{cv}+\psi_{ce}:\psi_{ev}+\psi_{ce}:\psi_{cv}+\psi_{ev}:\psi_{cv}$ | 0.311182 | 0.67667 | 37072 |
| 274 | DisplMean~ $\psi_{ac}+\psi_{ce}+\psi_{cv}+\psi_{ac}:\psi_{ev}+\psi_{ac}:\psi_{cv}+\psi_{ce}:\psi_{cv}$ | 0.43076 | 0.67665 | 37071.1 |
| 275 | DisplMean~ $\psi_{ac}+\psi_{ce}+\psi_{cv}+\psi_{ac}:\psi_{ev}+\psi_{ac}:\psi_{cv}$ | 0.432939 | 0.67656 | 37079.2 |
| 276 | DisplMean~ $\psi_{ce}+\psi_{ev}+\psi_{cv}+\psi_{ac}:\psi_{ev}+\psi_{ac}:\psi_{cv}+\psi_{ce}:\psi_{ev}+\psi_{ev}:\psi_{cv}$ | 0.312869 | 0.67654 | 37082.6 |
| 277 | DisplMean~ $\psi_{ac}+\psi_{cv}+\psi_{ac}:\psi_{ce}+\psi_{ac}:\psi_{ev}+\psi_{ac}:\psi_{cv}+\psi_{ce}:\psi_{ev}+\psi_{ev}:\psi_{cv}$ | 0.308427 | 0.67646 | 37090.2 |
| 278 | DisplMean~ $\psi_{ce}+\psi_{ev}+\psi_{cv}+\psi_{ac}:\psi_{ev}+\psi_{ac}:\psi_{cv}+\psi_{ce}:\psi_{cv}+\psi_{ev}:\psi_{cv}$ | 0.309225 | 0.67646 | 37090.1 |
| 279 | DisplMean~ $\psi_{ac}+\psi_{cv}+\psi_{ac}:\psi_{ce}+\psi_{ac}:\psi_{ev}+\psi_{ac}:\psi_{cv}+\psi_{ce}:\psi_{ev}$ | 0.308275 | 0.67642 | 37093.6 |
| 280 | DisplMean~ $\psi_{ce}+\psi_{ev}+\psi_{cv}+\psi_{ac}:\psi_{ev}+\psi_{ac}:\psi_{cv}+\psi_{ev}:\psi_{cv}$ | 0.310907 | 0.67634 | 37100.8 |
| 281 | DisplMean~ $\psi_{ce}+\psi_{ev}+\psi_{cv}+\psi_{ac}:\psi_{ev}+\psi_{ac}:\psi_{cv}+\psi_{ce}:\psi_{ev}+\psi_{ce}:\psi_{cv}$ | 0.304638 | 0.67599 | 37134.2 |
| 282 | DisplMean~ $\psi_{ce}+\psi_{ev}+\psi_{cv}+\psi_{ac}:\psi_{ev}+\psi_{ac}:\psi_{cv}+\psi_{ce}:\psi_{ev}$ | 0.306313 | 0.67587 | 37144.9 |
| 283 | DisplMean~ $\psi_{ce}+\psi_{ev}+\psi_{cv}+\psi_{ac}:\psi_{ev}+\psi_{ac}:\psi_{cv}+\psi_{ce}:\psi_{cv}$ | 0.303191 | 0.67584 | 37147.8 |
| 284 | DisplMean~ $\psi_{ce}+\psi_{ev}+\psi_{cv}+\psi_{ac}:\psi_{ev}+\psi_{ac}:\psi_{cv}$ | 0.304862 | 0.67571 | 37158.5 |
| 285 | DisplMean~ $\psi_{ac}+\psi_{ev}+\psi_{cv}+\psi_{ac}:\psi_{ev}+\psi_{ac}:\psi_{cv}+\psi_{ce}:\psi_{ev}+\psi_{ev}:\psi_{cv}$ | 0.427769 | 0.67561 | 37170.2 |
| 286 | DisplMean~ $\psi_{ac}+\psi_{ev}+\psi_{cv}+\psi_{ac}:\psi_{ev}+\psi_{ac}:\psi_{cv}+\psi_{ce}:\psi_{ev}$ | 0.427712 | 0.6756 | 37170 |
| 287 | DisplMean~ $\psi_{ac}+\psi_{ev}+\psi_{cv}+\psi_{ac}:\psi_{cv}+\psi_{ce}:\psi_{ev}$ | 0.42757 | 0.67549 | 37179.4 |
| 288 | DisplMean~ $\psi_{ac}+\psi_{ev}+\psi_{cv}+\psi_{ac}:\psi_{cv}+\psi_{ce}:\psi_{ev}+\psi_{ev}:\psi_{cv}$ | 0.427574 | 0.67548 | 37181.4 |
| 289 | DisplMean~ $\psi_{ac}+\psi_{cv}+\psi_{ac}:\psi_{ce}+\psi_{ac}:\psi_{cv}+\psi_{ce}:\psi_{ev}+\psi_{ce}:\psi_{cv}+\psi_{ev}:\psi_{cv}$ | 0.251585 | 0.67377 | 37343 |
| 290 | DisplMean~ $\psi_{ac}+\psi_{ev}+\psi_{cv}+\psi_{ac}:\psi_{ev}+\psi_{ac}:\psi_{cv}+\psi_{ce}:\psi_{cv}+\psi_{ev}:\psi_{cv}$ | 0.428441 | 0.67264 | 37448.4 |
| 291 | DisplMean~ $\psi_{ac}+\psi_{ev}+\psi_{cv}+\psi_{ac}:\psi_{ev}+\psi_{ac}:\psi_{cv}+\psi_{ce}:\psi_{cv}$ | 0.428293 | 0.67262 | 37448.4 |
| 292 | DisplMean~ $\psi_{ac}+\psi_{ev}+\psi_{cv}+\psi_{ac}:\psi_{cv}+\psi_{ce}:\psi_{cv}$ | 0.427904 | 0.67261 | 37448.5 |
| 293 | DisplMean~ $\psi_{ac}+\psi_{ev}+\psi_{cv}+\psi_{ac}:\psi_{cv}+\psi_{ce}:\psi_{cv}+\psi_{ev}:\psi_{cv}$ | 0.427917 | 0.67261 | 37449.8 |

| | | | | |
|---|---|---|---|---|
| 294 | DisplMean~ $\psi_{ev}+\psi_{cv}+\psi_{ac}:\psi_{ev}+\psi_{ac}:\psi_{cv}+\psi_{ce}:\psi_{ev}+\psi_{ce}:\psi_{cv}+\psi_{ev}:\psi_{cv}$ | 0.29999 | 0.66943 | 37745.1 |
| 295 | DisplMean~ $\psi_{ev}+\psi_{cv}+\psi_{ac}:\psi_{ev}+\psi_{ac}:\psi_{cv}+\psi_{ce}:\psi_{ev}+\psi_{ce}:\psi_{cv}$ | 0.295431 | 0.66901 | 37783.2 |
| 296 | DisplMean~ $\psi_{ac}+\psi_{cv}+\psi_{ac}:\psi_{ev}+\psi_{ac}:\psi_{cv}+\psi_{ce}:\psi_{ev}+\psi_{ce}:\psi_{cv}+\psi_{ev}:\psi_{cv}$ | 0.387636 | 0.66778 | 37897.2 |
| 297 | DisplMean~ $\psi_{ac}+\psi_{cv}+\psi_{ac}:\psi_{ev}+\psi_{ac}:\psi_{cv}+\psi_{ce}:\psi_{ev}+\psi_{ce}:\psi_{cv}$ | 0.387424 | 0.66757 | 37915 |
| 298 | DisplMean~ $\psi_{ac}+\psi_{cv}+\psi_{ac}:\psi_{ce}+\psi_{ac}:\psi_{cv}+\psi_{ce}:\psi_{cv}+\psi_{ev}:\psi_{cv}$ | 0.269358 | 0.66718 | 37950.5 |
| 299 | DisplMean~ $\psi_{ac}+\psi_{ce}+\psi_{cv}+\psi_{ac}:\psi_{cv}+\psi_{ce}:\psi_{ev}+\psi_{ce}:\psi_{cv}+\psi_{ev}:\psi_{cv}$ | 0.350772 | 0.6669 | 37977.6 |
| 300 | DisplMean~ $\psi_{ac}+\psi_{ce}+\psi_{cv}+\psi_{ac}:\psi_{cv}+\psi_{ce}:\psi_{ev}+\psi_{ev}:\psi_{cv}$ | 0.352802 | 0.66673 | 37991.9 |
| 301 | DisplMean~ $\psi_{ce}+\psi_{ev}+\psi_{cv}+\psi_{ac}:\psi_{cv}+\psi_{ce}:\psi_{cv}$ | 0.227046 | 0.66652 | 38010 |
| 302 | DisplMean~ $\psi_{ce}+\psi_{ev}+\psi_{cv}+\psi_{ac}:\psi_{cv}+\psi_{ce}:\psi_{ev}+\psi_{ce}:\psi_{cv}$ | 0.227031 | 0.66652 | 38011.6 |
| 303 | DisplMean~ $\psi_{ce}+\psi_{ev}+\psi_{cv}+\psi_{ac}:\psi_{cv}+\psi_{ce}:\psi_{cv}+\psi_{ev}:\psi_{cv}$ | 0.227048 | 0.66652 | 38011.3 |
| 304 | DisplMean~ $\psi_{ce}+\psi_{ev}+\psi_{cv}+\psi_{ac}:\psi_{cv}+\psi_{ce}:\psi_{ev}+\psi_{ce}:\psi_{cv}+\psi_{ev}:\psi_{cv}$ | 0.227032 | 0.66651 | 38012.9 |
| 305 | DisplMean~ $\psi_{ce}+\psi_{ev}+\psi_{cv}+\psi_{ac}:\psi_{cv}$ | 0.228387 | 0.66642 | 38018.2 |
| 306 | DisplMean~ $\psi_{ce}+\psi_{ev}+\psi_{cv}+\psi_{ac}:\psi_{cv}+\psi_{ev}:\psi_{cv}$ | 0.228388 | 0.66642 | 38019.5 |
| 307 | DisplMean~ $\psi_{ce}+\psi_{ev}+\psi_{cv}+\psi_{ac}:\psi_{cv}+\psi_{ce}:\psi_{ev}$ | 0.228372 | 0.66641 | 38019.8 |
| 308 | DisplMean~ $\psi_{ce}+\psi_{ev}+\psi_{cv}+\psi_{ac}:\psi_{cv}+\psi_{ce}:\psi_{ev}+\psi_{ev}:\psi_{cv}$ | 0.228373 | 0.66641 | 38021.1 |
| 309 | DisplMean~ $\psi_{ev}+\psi_{cv}+\psi_{ac}:\psi_{ev}+\psi_{ac}:\psi_{cv}+\psi_{ce}:\psi_{ev}+\psi_{ev}:\psi_{cv}$ | 0.285949 | 0.66627 | 38034.1 |
| 310 | DisplMean~ $\psi_{ev}+\psi_{cv}+\psi_{ac}:\psi_{ce}+\psi_{ac}:\psi_{cv}+\psi_{ce}:\psi_{ev}$ | 0.194312 | 0.66608 | 38050.3 |
| 311 | DisplMean~ $\psi_{ev}+\psi_{cv}+\psi_{ac}:\psi_{ce}+\psi_{ac}:\psi_{cv}+\psi_{ce}:\psi_{ev}+\psi_{ev}:\psi_{cv}$ | 0.194285 | 0.66608 | 38051.7 |
| 312 | DisplMean~ $\psi_{ev}+\psi_{cv}+\psi_{ac}:\psi_{ev}+\psi_{ac}:\psi_{cv}+\psi_{ce}:\psi_{ev}$ | 0.282397 | 0.66595 | 38062.1 |
| 313 | DisplMean~ $\psi_{ev}+\psi_{cv}+\psi_{ac}:\psi_{ev}+\psi_{ac}:\psi_{cv}+\psi_{ce}:\psi_{cv}+\psi_{ev}:\psi_{cv}$ | 0.304292 | 0.66559 | 38096 |
| 314 | DisplMean~ $\psi_{ev}+\psi_{cv}+\psi_{ac}:\psi_{ce}+\psi_{ac}:\psi_{cv}+\psi_{ce}:\psi_{cv}$ | 0.202495 | 0.66547 | 38106.3 |
| 315 | DisplMean~ $\psi_{ev}+\psi_{cv}+\psi_{ac}:\psi_{ce}+\psi_{ac}:\psi_{cv}+\psi_{ce}:\psi_{cv}+\psi_{ev}:\psi_{cv}$ | 0.202496 | 0.66546 | 38107.5 |
| 316 | DisplMean~ $\psi_{ev}+\psi_{cv}+\psi_{ac}:\psi_{ev}+\psi_{ac}:\psi_{cv}+\psi_{ce}:\psi_{cv}$ | 0.299284 | 0.66507 | 38142.5 |
| 317 | DisplMean~ $\psi_{ac}+\psi_{cv}+\psi_{ac}:\psi_{ev}+\psi_{ac}:\psi_{cv}+\psi_{ce}:\psi_{cv}+\psi_{ev}:\psi_{cv}$ | 0.374915 | 0.66405 | 38236.3 |
| 318 | DisplMean~ $\psi_{ac}+\psi_{cv}+\psi_{ac}:\psi_{ev}+\psi_{ac}:\psi_{cv}+\psi_{ce}:\psi_{cv}$ | 0.374296 | 0.66374 | 38262.8 |
| 319 | DisplMean~ $\psi_{ac}+\psi_{cv}+\psi_{ac}:\psi_{ev}+\psi_{ac}:\psi_{cv}+\psi_{ce}:\psi_{ev}+\psi_{ev}:\psi_{cv}$ | 0.364534 | 0.66346 | 38289.1 |
| 320 | DisplMean~ $\psi_{ac}+\psi_{cv}+\psi_{ac}:\psi_{ev}+\psi_{ac}:\psi_{cv}+\psi_{ce}:\psi_{ev}$ | 0.364512 | 0.66333 | 38300.4 |

| | | | | |
|---|---|---|---|---|
| 321 | DisplMean~ $\psi_{ev}+\psi_{cv}+\psi_{ac}:\psi_{cv}+\psi_{ce}:\psi_{ev}+\psi_{ce}:\psi_{cv}$ | 0.230143 | 0.66147 | 38468 |
| 322 | DisplMean~ $\psi_{ev}+\psi_{cv}+\psi_{ac}:\psi_{cv}+\psi_{ce}:\psi_{ev}+\psi_{ce}:\psi_{cv}+\psi_{ev}:\psi_{cv}$ | 0.230145 | 0.66146 | 38469.9 |
| 323 | DisplMean~ $\psi_{ev}+\psi_{cv}+\psi_{ac}:\psi_{cv}+\psi_{ce}:\psi_{ev}$ | 0.224404 | 0.65919 | 38671.7 |
| 324 | DisplMean~ $\psi_{ev}+\psi_{cv}+\psi_{ac}:\psi_{cv}+\psi_{ce}:\psi_{ev}+\psi_{ev}:\psi_{cv}$ | 0.224404 | 0.65918 | 38673.7 |
| 325 | DisplMean~ $\psi_{ac}+\psi_{ce}+\psi_{cv}+\psi_{ac}:\psi_{cv}+\psi_{ce}:\psi_{cv}+\psi_{ev}:\psi_{cv}$ | 0.326408 | 0.65726 | 38845.7 |
| 326 | DisplMean~ $\psi_{ev}+\psi_{cv}+\psi_{ac}:\psi_{cv}+\psi_{ce}:\psi_{cv}$ | 0.232598 | 0.6572 | 38849.1 |
| 327 | DisplMean~ $\psi_{ev}+\psi_{cv}+\psi_{ac}:\psi_{cv}+\psi_{ce}:\psi_{cv}+\psi_{ev}:\psi_{cv}$ | 0.2326 | 0.65719 | 38850.4 |
| 328 | DisplMean~ $\psi_{ac}+\psi_{ce}+\psi_{cv}+\psi_{ac}:\psi_{cv}+\psi_{ev}:\psi_{cv}$ | 0.328441 | 0.65706 | 38862.2 |
| 329 | DisplMean~ $\psi_{ce}+\psi_{cv}+\psi_{ac}:\psi_{ce}+\psi_{ac}:\psi_{ev}+\psi_{ac}:\psi_{cv}+\psi_{ce}:\psi_{cv}$ | 0.134731 | 0.65618 | 38941.4 |
| 330 | DisplMean~ $\psi_{ce}+\psi_{cv}+\psi_{ac}:\psi_{ce}+\psi_{ac}:\psi_{ev}+\psi_{ac}:\psi_{cv}$ | 0.134889 | 0.655 | 39045.2 |
| 331 | DisplMean~ $\psi_{ce}+\psi_{cv}+\psi_{ac}:\psi_{ev}+\psi_{ac}:\psi_{cv}+\psi_{ce}:\psi_{ev}+\psi_{ce}:\psi_{cv}+\psi_{ev}:\psi_{cv}$ | 0.221297 | 0.65397 | 39137.7 |
| 332 | DisplMean~ $\psi_{ce}+\psi_{cv}+\psi_{ac}:\psi_{cv}+\psi_{ce}:\psi_{ev}+\psi_{ce}:\psi_{cv}+\psi_{ev}:\psi_{cv}$ | 0.217584 | 0.65388 | 39144.9 |
| 333 | DisplMean~ $\psi_{ce}+\psi_{cv}+\psi_{ac}:\psi_{ev}+\psi_{ac}:\psi_{cv}+\psi_{ce}:\psi_{ev}+\psi_{ev}:\psi_{cv}$ | 0.222603 | 0.65383 | 39148.5 |
| 334 | DisplMean~ $\psi_{ce}+\psi_{cv}+\psi_{ac}:\psi_{cv}+\psi_{ce}:\psi_{ev}+\psi_{ev}:\psi_{cv}$ | 0.218977 | 0.65375 | 39155 |
| 335 | DisplMean~ $\psi_{ac}+\psi_{ev}+\psi_{cv}+\psi_{ac}:\psi_{ce}+\psi_{ac}:\psi_{cv}$ | 0.413877 | 0.64882 | 39586 |
| 336 | DisplMean~ $\psi_{ac}+\psi_{ev}+\psi_{cv}+\psi_{ac}:\psi_{ce}+\psi_{ac}:\psi_{ev}+\psi_{ac}:\psi_{cv}$ | 0.414174 | 0.64882 | 39586.9 |
| 337 | DisplMean~ $\psi_{ac}+\psi_{ev}+\psi_{cv}+\psi_{ac}:\psi_{ce}+\psi_{ac}:\psi_{ev}+\psi_{ac}:\psi_{cv}+\psi_{ev}:\psi_{cv}$ | 0.414312 | 0.64882 | 39587.5 |
| 338 | DisplMean~ $\psi_{ac}+\psi_{ev}+\psi_{cv}+\psi_{ac}:\psi_{ce}+\psi_{ac}:\psi_{cv}+\psi_{ev}:\psi_{cv}$ | 0.413889 | 0.64881 | 39587.3 |
| 339 | DisplMean~ $\psi_{ac}+\psi_{ce}+\psi_{cv}+\psi_{ac}:\psi_{cv}+\psi_{ce}:\psi_{ev}+\psi_{ce}:\psi_{cv}$ | 0.292582 | 0.64738 | 39711.5 |
| 340 | DisplMean~ $\psi_{ac}+\psi_{ce}+\psi_{cv}+\psi_{ac}:\psi_{cv}+\psi_{ce}:\psi_{ev}$ | 0.294572 | 0.64714 | 39731.1 |
| 341 | DisplMean~ $\psi_{ce}+\psi_{cv}+\psi_{ac}:\psi_{ev}+\psi_{ac}:\psi_{cv}+\psi_{ce}:\psi_{cv}+\psi_{ev}:\psi_{cv}$ | 0.204787 | 0.64675 | 39765.2 |
| 342 | DisplMean~ $\psi_{ce}+\psi_{cv}+\psi_{ac}:\psi_{ev}+\psi_{ac}:\psi_{cv}+\psi_{ev}:\psi_{cv}$ | 0.2059 | 0.64665 | 39773.6 |
| 343 | DisplMean~ $\psi_{cv}+\psi_{ac}:\psi_{ce}+\psi_{ac}:\psi_{ev}+\psi_{ac}:\psi_{cv}+\psi_{ce}:\psi_{ev}+\psi_{ce}:\psi_{cv}+\psi_{ev}:\psi_{cv}$ | 0.183571 | 0.64644 | 39793 |
| 344 | DisplMean~ $\psi_{ac}+\psi_{ev}+\psi_{cv}+\psi_{ac}:\psi_{ce}+\psi_{ac}:\psi_{ev}+\psi_{ce}:\psi_{ev}+\psi_{ce}:\psi_{cv}+\psi_{ev}:\psi_{cv}$ | 0.138894 | 0.6464 | 39797.8 |
| 345 | DisplMean~ $\psi_{ce}+\psi_{ac}:\psi_{ce}+\psi_{ac}:\psi_{ev}+\psi_{ac}:\psi_{cv}+\psi_{ce}:\psi_{cv}+\psi_{ev}:\psi_{cv}$ | 0.120947 | 0.64622 | 39811.6 |
| 346 | DisplMean~ $\psi_{ce}+\psi_{ac}:\psi_{ce}+\psi_{ac}:\psi_{ev}+\psi_{ac}:\psi_{cv}+\psi_{ev}:\psi_{cv}$ | 0.12047 | 0.64611 | 39819.9 |
| 347 | DisplMean~ $\psi_{cv}+\psi_{ac}:\psi_{ce}+\psi_{ac}:\psi_{cv}+\psi_{ce}:\psi_{ev}+\psi_{ce}:\psi_{cv}+\psi_{ev}:\psi_{cv}$ | 0.186843 | 0.64608 | 39823.5 |

| | | | | |
|---|---|---|---|---|
| 348 | DisplMean~ $\psi_{ac}$+$\psi_{cv}$+$\psi_{ac}$:$\psi_{cv}$+$\psi_{ce}$:$\psi_{ev}$+$\psi_{ce}$:$\psi_{cv}$+$\psi_{ev}$:$\psi_{cv}$ | 0.312252 | 0.64575 | 39851.8 |
| 349 | DisplMean~ $\psi_{ac}$+$\psi_{ev}$+$\psi_{cv}$+$\psi_{ac}$:$\psi_{ce}$+$\psi_{ac}$:$\psi_{ev}$+$\psi_{ce}$:$\psi_{ev}$+$\psi_{ce}$:$\psi_{cv}$ | 0.139403 | 0.64575 | 39853 |
| 350 | DisplMean~ $\psi_{ac}$+$\psi_{cv}$+$\psi_{ac}$:$\psi_{cv}$+$\psi_{ce}$:$\psi_{cv}$+$\psi_{ev}$:$\psi_{cv}$ | 0.311358 | 0.6456 | 39863.6 |
| 351 | DisplMean~ $\psi_{ce}$+$\psi_{cv}$+$\psi_{ac}$:$\psi_{cv}$+$\psi_{ce}$:$\psi_{cv}$+$\psi_{ev}$:$\psi_{cv}$ | 0.214459 | 0.6455 | 39872.3 |
| 352 | DisplMean~ $\psi_{ce}$+$\psi_{cv}$+$\psi_{ac}$:$\psi_{cv}$+$\psi_{ev}$:$\psi_{cv}$ | 0.215871 | 0.64536 | 39883.1 |
| 353 | DisplMean~ $\psi_{cv}$+$\psi_{ac}$:$\psi_{ce}$+$\psi_{ac}$:$\psi_{ev}$+$\psi_{ac}$:$\psi_{cv}$+$\psi_{ce}$:$\psi_{cv}$+$\psi_{ev}$:$\psi_{cv}$ | 0.190651 | 0.64522 | 39896.8 |
| 354 | DisplMean~ $\psi_{cv}$+$\psi_{ac}$:$\psi_{ce}$+$\psi_{ac}$:$\psi_{cv}$+$\psi_{ce}$:$\psi_{cv}$+$\psi_{ev}$:$\psi_{cv}$ | 0.192249 | 0.6451 | 39906.6 |
| 355 | DisplMean~ $\psi_{ev}$+$\psi_{cv}$+$\psi_{ac}$:$\psi_{ce}$+$\psi_{ac}$:$\psi_{ev}$+$\psi_{ac}$:$\psi_{cv}$+$\psi_{ev}$:$\psi_{cv}$ | 0.303467 | 0.64379 | 40019.4 |
| 356 | DisplMean~ $\psi_{ac}$+$\psi_{ev}$+$\psi_{cv}$+$\psi_{ac}$:$\psi_{ce}$+$\psi_{ce}$:$\psi_{ev}$+$\psi_{ce}$:$\psi_{cv}$+$\psi_{ev}$:$\psi_{cv}$ | 0.139538 | 0.64371 | 40027.3 |
| 357 | DisplMean~ $\psi_{ac}$+$\psi_{ev}$+$\psi_{cv}$+$\psi_{ac}$:$\psi_{ce}$+$\psi_{ce}$:$\psi_{ev}$+$\psi_{ce}$:$\psi_{cv}$ | 0.139634 | 0.64366 | 40031 |
| 358 | DisplMean~ $\psi_{ev}$+$\psi_{cv}$+$\psi_{ac}$:$\psi_{ce}$+$\psi_{ac}$:$\psi_{ev}$+$\psi_{ac}$:$\psi_{cv}$ | 0.299201 | 0.64344 | 40048.5 |
| 359 | DisplMean~ $\psi_{ce}$+$\psi_{cv}$+$\psi_{ac}$:$\psi_{ev}$+$\psi_{ac}$:$\psi_{cv}$+$\psi_{ce}$:$\psi_{ev}$+$\psi_{ce}$:$\psi_{cv}$ | 0.193996 | 0.64338 | 40054.7 |
| 360 | DisplMean~ $\psi_{ce}$+$\psi_{cv}$+$\psi_{ac}$:$\psi_{ev}$+$\psi_{ac}$:$\psi_{cv}$+$\psi_{ce}$:$\psi_{ev}$ | 0.195001 | 0.64329 | 40061.7 |
| 361 | DisplMean~ $\psi_{ac}$+$\psi_{cv}$+$\psi_{ac}$:$\psi_{ce}$+$\psi_{ac}$:$\psi_{ev}$+$\psi_{ac}$:$\psi_{cv}$+$\psi_{ev}$:$\psi_{cv}$ | 0.362858 | 0.6407 | 40282.9 |
| 362 | DisplMean~ $\psi_{ac}$+$\psi_{cv}$+$\psi_{ac}$:$\psi_{ce}$+$\psi_{ac}$:$\psi_{ev}$+$\psi_{ac}$:$\psi_{cv}$ | 0.362296 | 0.64042 | 40305.3 |
| 363 | DisplMean~ $\psi_{ac}$+$\psi_{ev}$+$\psi_{cv}$+$\psi_{ac}$:$\psi_{ev}$+$\psi_{ac}$:$\psi_{cv}$+$\psi_{ev}$:$\psi_{cv}$ | 0.733939 | 0.64011 | 40333.1 |
| 364 | DisplMean~ $\psi_{ac}$+$\psi_{ev}$+$\psi_{cv}$+$\psi_{ac}$:$\psi_{ev}$+$\psi_{ac}$:$\psi_{cv}$ | 0.732256 | 0.64008 | 40334.3 |
| 365 | DisplMean~ $\psi_{ac}$+$\psi_{ev}$+$\psi_{cv}$+$\psi_{ac}$:$\psi_{cv}$ | 0.726014 | 0.64002 | 40338.8 |
| 366 | DisplMean~ $\psi_{ac}$+$\psi_{ev}$+$\psi_{cv}$+$\psi_{ac}$:$\psi_{cv}$+$\psi_{ev}$:$\psi_{cv}$ | 0.72613 | 0.64001 | 40340 |
| 367 | DisplMean~ $\psi_{ev}$+$\psi_{cv}$+$\psi_{ac}$:$\psi_{ce}$+$\psi_{ac}$:$\psi_{cv}$ | 0.245567 | 0.63876 | 40444.7 |
| 368 | DisplMean~ $\psi_{ev}$+$\psi_{cv}$+$\psi_{ac}$:$\psi_{ce}$+$\psi_{ac}$:$\psi_{cv}$+$\psi_{ev}$:$\psi_{cv}$ | 0.245568 | 0.63876 | 40446.1 |
| 369 | DisplMean~ $\psi_{cv}$+$\psi_{ac}$:$\psi_{ev}$+$\psi_{ac}$:$\psi_{cv}$+$\psi_{ce}$:$\psi_{cv}$+$\psi_{ev}$:$\psi_{cv}$ | 0.208791 | 0.63771 | 40534.7 |
| 370 | DisplMean~ $\psi_{cv}$+$\psi_{ac}$:$\psi_{ev}$+$\psi_{ac}$:$\psi_{cv}$+$\psi_{ce}$:$\psi_{ev}$+$\psi_{ce}$:$\psi_{cv}$+$\psi_{ev}$:$\psi_{cv}$ | 0.208829 | 0.63769 | 40536.7 |
| 371 | DisplMean~ $\psi_{ce}$+$\psi_{cv}$+$\psi_{ac}$:$\psi_{cv}$+$\psi_{ce}$:$\psi_{ev}$+$\psi_{ce}$:$\psi_{cv}$ | 0.207806 | 0.63763 | 40540.7 |
| 372 | DisplMean~ $\psi_{ce}$+$\psi_{cv}$+$\psi_{ac}$:$\psi_{cv}$+$\psi_{ce}$:$\psi_{ev}$ | 0.209253 | 0.63748 | 40552.8 |
| 373 | DisplMean~ $\psi_{cv}$+$\psi_{ac}$:$\psi_{cv}$+$\psi_{ce}$:$\psi_{ev}$+$\psi_{ce}$:$\psi_{cv}$+$\psi_{ev}$:$\psi_{cv}$ | 0.220957 | 0.63623 | 40658.3 |
| 374 | DisplMean~ $\psi_{cv}$+$\psi_{ac}$:$\psi_{cv}$+$\psi_{ce}$:$\psi_{cv}$+$\psi_{ev}$:$\psi_{cv}$ | 0.22006 | 0.63603 | 40674.4 |

| | | | | |
|---|---|---|---|---|
| 375 | DisplMean~ $\psi_{ce}+\psi_{cv}+\psi_{ac}:\psi_{ev}+\psi_{ac}:\psi_{cv}+\psi_{ce}:\psi_{cv}$ | 0.174161 | 0.631 | 41093 |
| 376 | DisplMean~ $\psi_{ce}+\psi_{cv}+\psi_{ac}:\psi_{ev}+\psi_{ac}:\psi_{cv}$ | 0.174831 | 0.63096 | 41095.7 |
| 377 | DisplMean~ $\psi_{ac}+\psi_{cv}+\psi_{ac}:\psi_{ce}+\psi_{ac}:\psi_{cv}+\psi_{ce}:\psi_{ev}+\psi_{ev}:\psi_{cv}$ | 0.279667 | 0.62985 | 41188.8 |
| 378 | DisplMean~ $\psi_{ac}+\psi_{cv}+\psi_{ac}:\psi_{cv}+\psi_{ce}:\psi_{ev}+\psi_{ev}:\psi_{cv}$ | 0.284174 | 0.62964 | 41205.2 |
| 379 | DisplMean~ $\psi_{ev}+\psi_{cv}+\psi_{ac}:\psi_{ev}+\psi_{ac}:\psi_{cv}+\psi_{ev}:\psi_{cv}$ | 0.297282 | 0.62913 | 41247.2 |
| 380 | DisplMean~ $\psi_{ac}+\psi_{cv}+\psi_{ac}:\psi_{ev}+\psi_{ac}:\psi_{cv}+\psi_{ev}:\psi_{cv}$ | 0.411252 | 0.62861 | 41289.7 |
| 381 | DisplMean~ $\psi_{ev}+\psi_{cv}+\psi_{ac}:\psi_{ev}+\psi_{ac}:\psi_{cv}$ | 0.291598 | 0.62859 | 41290.4 |
| 382 | DisplMean~ $\psi_{ac}+\psi_{cv}+\psi_{ac}:\psi_{ev}+\psi_{ac}:\psi_{cv}$ | 0.410289 | 0.62829 | 41315.3 |
| 383 | DisplMean~ $\psi_{ac}+\psi_{cv}+\psi_{ac}:\psi_{ce}+\psi_{ac}:\psi_{ev}+\psi_{ce}:\psi_{ev}+\psi_{ce}:\psi_{cv}$ | 0.148648 | 0.62713 | 41412.5 |
| 384 | DisplMean~ $\psi_{ac}+\psi_{cv}+\psi_{ac}:\psi_{ce}+\psi_{ac}:\psi_{ev}+\psi_{ce}:\psi_{ev}+\psi_{ce}:\psi_{cv}+\psi_{ev}:\psi_{cv}$ | 0.148633 | 0.62711 | 41414.4 |
| 385 | DisplMean~ $\psi_{cv}+\psi_{ac}:\psi_{ce}+\psi_{ac}:\psi_{ev}+\psi_{ac}:\psi_{cv}+\psi_{ce}:\psi_{ev}+\psi_{ev}:\psi_{cv}$ | 0.191979 | 0.62656 | 41459 |
| 386 | DisplMean~ $\psi_{cv}+\psi_{ac}:\psi_{ev}+\psi_{ac}:\psi_{cv}+\psi_{ce}:\psi_{ev}+\psi_{ev}:\psi_{cv}$ | 0.189284 | 0.62648 | 41463.9 |
| 387 | DisplMean~ $\psi_{cv}+\psi_{ac}:\psi_{ce}+\psi_{ac}:\psi_{ev}+\psi_{ac}:\psi_{cv}+\psi_{ce}:\psi_{ev}+\psi_{ce}:\psi_{cv}$ | 0.171564 | 0.62561 | 41535.8 |
| 388 | DisplMean~ $\psi_{cv}+\psi_{ac}:\psi_{ce}+\psi_{ac}:\psi_{ev}+\psi_{ac}:\psi_{cv}+\psi_{ce}:\psi_{cv}$ | 0.169136 | 0.62547 | 41546.6 |
| 389 | DisplMean~ $\psi_{cv}+\psi_{ac}:\psi_{ev}+\psi_{ac}:\psi_{cv}+\psi_{ce}:\psi_{ev}+\psi_{ce}:\psi_{cv}$ | 0.182723 | 0.6237 | 41690.5 |
| 390 | DisplMean~ $\psi_{cv}+\psi_{ac}:\psi_{ev}+\psi_{ac}:\psi_{cv}+\psi_{ce}:\psi_{cv}$ | 0.178754 | 0.62307 | 41740.4 |
| 391 | DisplMean~ $\psi_{ac}+\psi_{cv}+\psi_{ac}:\psi_{ce}+\psi_{ac}:\psi_{cv}+\psi_{ev}:\psi_{cv}$ | 0.302736 | 0.6225 | 41787.4 |
| 392 | DisplMean~ $\psi_{cv}+\psi_{ac}:\psi_{ce}+\psi_{ac}:\psi_{cv}+\psi_{ce}:\psi_{ev}+\psi_{ev}:\psi_{cv}$ | 0.2126 | 0.62119 | 41892.9 |
| 393 | DisplMean~ $\psi_{ev}+\psi_{cv}+\psi_{ac}:\psi_{cv}$ | 0.221993 | 0.62107 | 41900.2 |
| 394 | DisplMean~ $\psi_{ev}+\psi_{cv}+\psi_{ac}:\psi_{cv}+\psi_{ev}:\psi_{cv}$ | 0.221995 | 0.62107 | 41901.6 |
| 395 | DisplMean~ $\psi_{cv}+\psi_{ac}:\psi_{cv}+\psi_{ce}:\psi_{ev}+\psi_{ev}:\psi_{cv}$ | 0.207398 | 0.62096 | 41910.4 |
| 396 | DisplMean~ $\psi_{cv}+\psi_{ac}:\psi_{ce}+\psi_{ac}:\psi_{ev}+\psi_{ac}:\psi_{cv}+\psi_{ev}:\psi_{cv}$ | 0.214322 | 0.62077 | 41926.3 |
| 397 | DisplMean~ $\psi_{cv}+\psi_{ac}:\psi_{ce}+\psi_{ac}:\psi_{cv}+\psi_{ev}:\psi_{cv}$ | 0.231537 | 0.61721 | 42210.1 |
| 398 | DisplMean~ $\psi_{cv}+\psi_{ac}:\psi_{ce}+\psi_{ac}:\psi_{ev}+\psi_{ac}:\psi_{cv}+\psi_{ce}:\psi_{ev}$ | 0.178249 | 0.61127 | 42679.7 |
| 399 | DisplMean~ $\psi_{cv}+\psi_{ac}:\psi_{ce}+\psi_{ac}:\psi_{ev}+\psi_{ac}:\psi_{cv}$ | 0.187505 | 0.6102 | 42762.5 |
| 400 | DisplMean~ $\psi_{ac}+\psi_{cv}+\psi_{ac}:\psi_{cv}+\psi_{ev}:\psi_{cv}$ | 0.315221 | 0.60963 | 42807 |
| 401 | DisplMean~ $\psi_{cv}+\psi_{ac}:\psi_{ev}+\psi_{ac}:\psi_{cv}+\psi_{ce}:\psi_{ev}$ | 0.161835 | 0.60776 | 42953.2 |

| | | | | |
|---|---|---|---|---|
| 402 | DisplMean~ $\psi_{cv}$+$\psi_{ac}$:$\psi_{ev}$+$\psi_{ac}$:$\psi_{cv}$+$\psi_{ev}$:$\psi_{cv}$ | 0.197892 | 0.60207 | 43391.6 |
| 403 | DisplMean~ $\psi_{cv}$+$\psi_{ac}$:$\psi_{cv}$+$\psi_{ev}$:$\psi_{cv}$ | 0.210634 | 0.60019 | 43534.2 |
| 404 | DisplMean~ $\psi_{ac}$+$\psi_{cv}$+$\psi_{ac}$:$\psi_{ce}$+$\psi_{ac}$:$\psi_{cv}$+$\psi_{ce}$:$\psi_{ev}$+$\psi_{ce}$:$\psi_{cv}$ | 0.248205 | 0.59697 | 43781.3 |
| 405 | DisplMean~ $\psi_{ac}$+$\psi_{cv}$+$\psi_{ac}$:$\psi_{cv}$+$\psi_{ce}$:$\psi_{ev}$+$\psi_{ce}$:$\psi_{cv}$ | 0.262904 | 0.59317 | 44066.3 |
| 406 | DisplMean~ $\psi_{cv}$+$\psi_{ac}$:$\psi_{ce}$+$\psi_{ac}$:$\psi_{cv}$+$\psi_{ce}$:$\psi_{ev}$+$\psi_{ce}$:$\psi_{cv}$ | 0.204838 | 0.58842 | 44419.8 |
| 407 | DisplMean~ $\psi_{cv}$+$\psi_{ac}$:$\psi_{ev}$+$\psi_{ac}$:$\psi_{cv}$ | 0.168174 | 0.58834 | 44423.7 |
| 408 | DisplMean~ $\psi_{cv}$+$\psi_{ac}$:$\psi_{cv}$+$\psi_{ce}$:$\psi_{ev}$+$\psi_{ce}$:$\psi_{cv}$ | 0.212678 | 0.58791 | 44456.5 |
| 409 | DisplMean~ $\psi_{ac}$+$\psi_{ev}$+$\psi_{ac}$:$\psi_{ce}$+$\psi_{ac}$:$\psi_{ev}$+$\psi_{ac}$:$\psi_{cv}$+$\psi_{ce}$:$\psi_{ev}$+$\psi_{ce}$:$\psi_{cv}$+$\psi_{ev}$:$\psi_{cv}$ | 0.151358 | 0.58717 | 44515.7 |
| 410 | DisplMean~ $\psi_{ac}$+$\psi_{ev}$+$\psi_{ac}$:$\psi_{ce}$+$\psi_{ac}$:$\psi_{cv}$+$\psi_{ce}$:$\psi_{ev}$+$\psi_{ce}$:$\psi_{cv}$+$\psi_{ev}$:$\psi_{cv}$ | 0.150968 | 0.587 | 44526.7 |
| 411 | DisplMean~ $\psi_{ac}$+$\psi_{ev}$+$\psi_{ac}$:$\psi_{ce}$+$\psi_{ac}$:$\psi_{ev}$+$\psi_{ce}$:$\psi_{ev}$+$\psi_{ce}$:$\psi_{cv}$+$\psi_{ev}$:$\psi_{cv}$ | 0.143341 | 0.58609 | 44594.1 |
| 412 | DisplMean~ $\psi_{ac}$+$\psi_{ev}$+$\psi_{ac}$:$\psi_{ce}$+$\psi_{ce}$:$\psi_{ev}$+$\psi_{ce}$:$\psi_{cv}$+$\psi_{ev}$:$\psi_{cv}$ | 0.143489 | 0.58606 | 44595 |
| 413 | DisplMean~ $\psi_{ce}$+$\psi_{cv}$+$\psi_{ac}$:$\psi_{ce}$+$\psi_{ac}$:$\psi_{cv}$+$\psi_{ce}$:$\psi_{cv}$ | 0.127917 | 0.5843 | 44723.4 |
| 414 | DisplMean~ $\psi_{ce}$+$\psi_{cv}$+$\psi_{ac}$:$\psi_{ce}$+$\psi_{ac}$:$\psi_{cv}$ | 0.128534 | 0.58209 | 44883.8 |
| 415 | DisplMean~ $\psi_{ac}$+$\psi_{ac}$:$\psi_{ce}$+$\psi_{ac}$:$\psi_{ev}$+$\psi_{ac}$:$\psi_{cv}$+$\psi_{ce}$:$\psi_{ev}$+$\psi_{ce}$:$\psi_{cv}$+$\psi_{ev}$:$\psi_{cv}$ | 0.159197 | 0.57723 | 45239.3 |
| 416 | DisplMean~ $\psi_{ac}$+$\psi_{ac}$:$\psi_{ce}$+$\psi_{ac}$:$\psi_{ev}$+$\psi_{ce}$:$\psi_{ev}$+$\psi_{ce}$:$\psi_{cv}$+$\psi_{ev}$:$\psi_{cv}$ | 0.149339 | 0.57507 | 45393.7 |
| 417 | DisplMean~ $\psi_{ac}$+$\psi_{ac}$:$\psi_{ce}$+$\psi_{ac}$:$\psi_{cv}$+$\psi_{ce}$:$\psi_{ev}$+$\psi_{ce}$:$\psi_{cv}$+$\psi_{ev}$:$\psi_{cv}$ | 0.165557 | 0.56618 | 46024.2 |
| 418 | DisplMean~ $\psi_{ac}$+$\psi_{ce}$+$\psi_{ev}$+$\psi_{ac}$:$\psi_{ev}$+$\psi_{ac}$:$\psi_{cv}$+$\psi_{ce}$:$\psi_{ev}$+$\psi_{ce}$:$\psi_{cv}$+$\psi_{ev}$:$\psi_{cv}$ | 0.241922 | 0.56496 | 46111.5 |
| 419 | DisplMean~ $\psi_{ac}$+$\psi_{cv}$+$\psi_{ac}$:$\psi_{ce}$+$\psi_{ce}$:$\psi_{ev}$+$\psi_{ce}$:$\psi_{cv}$+$\psi_{ev}$:$\psi_{cv}$ | 0.140928 | 0.56467 | 46129.8 |
| 420 | DisplMean~ $\psi_{ac}$+$\psi_{ce}$+$\psi_{ev}$+$\psi_{ac}$:$\psi_{ev}$+$\psi_{ac}$:$\psi_{cv}$+$\psi_{ce}$:$\psi_{cv}$+$\psi_{ev}$:$\psi_{cv}$ | 0.241989 | 0.56442 | 46148.8 |
| 421 | DisplMean~ $\psi_{ac}$+$\psi_{ev}$+$\psi_{ac}$:$\psi_{ce}$+$\psi_{ac}$:$\psi_{ev}$+$\psi_{ac}$:$\psi_{cv}$+$\psi_{ce}$:$\psi_{ev}$+$\psi_{ev}$:$\psi_{cv}$ | 0.175215 | 0.56421 | 46163.3 |
| 422 | DisplMean~ $\psi_{ac}$+$\psi_{ev}$+$\psi_{ac}$:$\psi_{ce}$+$\psi_{ac}$:$\psi_{cv}$+$\psi_{ce}$:$\psi_{ev}$+$\psi_{ev}$:$\psi_{cv}$ | 0.175001 | 0.56418 | 46164.3 |
| 423 | DisplMean~ $\psi_{ac}$+$\psi_{ce}$+$\psi_{ac}$:$\psi_{ev}$+$\psi_{ac}$:$\psi_{cv}$+$\psi_{ce}$:$\psi_{ev}$+$\psi_{ce}$:$\psi_{cv}$+$\psi_{ev}$:$\psi_{cv}$ | 0.236967 | 0.56395 | 46181.7 |
| 424 | DisplMean~ $\psi_{ac}$+$\psi_{ce}$+$\psi_{ac}$:$\psi_{ev}$+$\psi_{ac}$:$\psi_{cv}$+$\psi_{ce}$:$\psi_{cv}$+$\psi_{ev}$:$\psi_{cv}$ | 0.236711 | 0.56294 | 46251 |
| 425 | DisplMean~ $\psi_{ac}$+$\psi_{ce}$+$\psi_{ev}$+$\psi_{ac}$:$\psi_{cv}$+$\psi_{ce}$:$\psi_{ev}$+$\psi_{ce}$:$\psi_{cv}$+$\psi_{ev}$:$\psi_{cv}$ | 0.241672 | 0.56213 | 46308.3 |
| 426 | DisplMean~ $\psi_{ac}$+$\psi_{ce}$+$\psi_{ev}$+$\psi_{ac}$:$\psi_{cv}$+$\psi_{ce}$:$\psi_{cv}$+$\psi_{ev}$:$\psi_{cv}$ | 0.241722 | 0.56196 | 46319.2 |
| 427 | DisplMean~ $\psi_{ac}$+$\psi_{ce}$+$\psi_{ac}$:$\psi_{cv}$+$\psi_{ce}$:$\psi_{cv}$+$\psi_{ev}$:$\psi_{cv}$ | 0.245118 | 0.56185 | 46325.9 |
| 428 | DisplMean~ $\psi_{ac}$+$\psi_{ce}$+$\psi_{ac}$:$\psi_{cv}$+$\psi_{ce}$:$\psi_{ev}$+$\psi_{ce}$:$\psi_{cv}$+$\psi_{ev}$:$\psi_{cv}$ | 0.245775 | 0.56184 | 46327.3 |

| | | | | |
|---|---|---|---|---|
| 429 | DisplMean~ $\psi_{ac}$+$\psi_{ev}$+$\psi_{cv}$+$\psi_{ac}$:$\psi_{ce}$+$\psi_{ac}$:$\psi_{ev}$+$\psi_{ce}$:$\psi_{cv}$ | 0.165164 | 0.56045 | 46423.5 |
| 430 | DisplMean~ $\psi_{ac}$+$\psi_{ev}$+$\psi_{cv}$+$\psi_{ac}$:$\psi_{ce}$+$\psi_{ac}$:$\psi_{ev}$+$\psi_{ce}$:$\psi_{cv}$+$\psi_{ev}$:$\psi_{cv}$ | 0.165171 | 0.56044 | 46425.4 |
| 431 | DisplMean~ $\psi_{ac}$+$\psi_{ev}$+$\psi_{cv}$+$\psi_{ac}$:$\psi_{ce}$+$\psi_{ce}$:$\psi_{cv}$ | 0.16503 | 0.5604 | 46426.1 |
| 432 | DisplMean~ $\psi_{ac}$+$\psi_{ev}$+$\psi_{cv}$+$\psi_{ac}$:$\psi_{ce}$+$\psi_{ce}$:$\psi_{cv}$+$\psi_{ev}$:$\psi_{cv}$ | 0.165028 | 0.56039 | 46427.9 |
| 433 | DisplMean~ $\psi_{ce}$+$\psi_{ev}$+$\psi_{ac}$:$\psi_{ev}$+$\psi_{ac}$:$\psi_{cv}$+$\psi_{ce}$:$\psi_{ev}$+$\psi_{ce}$:$\psi_{cv}$+$\psi_{ev}$:$\psi_{cv}$ | 0.206713 | 0.55965 | 46480.4 |
| 434 | DisplMean~ $\psi_{ce}$+$\psi_{ev}$+$\psi_{ac}$:$\psi_{ev}$+$\psi_{ac}$:$\psi_{cv}$+$\psi_{ce}$:$\psi_{cv}$+$\psi_{ev}$:$\psi_{cv}$ | 0.2045 | 0.55828 | 46573.6 |
| 435 | DisplMean~ $\psi_{ac}$+$\psi_{cv}$+$\psi_{ac}$:$\psi_{ce}$+$\psi_{ac}$:$\psi_{ev}$+$\psi_{ce}$:$\psi_{cv}$+$\psi_{ev}$:$\psi_{cv}$ | 0.165061 | 0.55586 | 46740.1 |
| 436 | DisplMean~ $\psi_{ac}$+$\psi_{cv}$+$\psi_{ac}$:$\psi_{ce}$+$\psi_{ac}$:$\psi_{ev}$+$\psi_{ce}$:$\psi_{cv}$ | 0.165266 | 0.55576 | 46746.1 |
| 437 | DisplMean~ $\psi_{ac}$+$\psi_{ac}$:$\psi_{ce}$+$\psi_{ac}$:$\psi_{ev}$+$\psi_{ac}$:$\psi_{cv}$+$\psi_{ce}$:$\psi_{ev}$+$\psi_{ev}$:$\psi_{cv}$ | 0.182727 | 0.55427 | 46849.3 |
| 438 | DisplMean~ $\psi_{ac}$+$\psi_{ac}$:$\psi_{ce}$+$\psi_{ce}$:$\psi_{ev}$+$\psi_{ce}$:$\psi_{cv}$+$\psi_{ev}$:$\psi_{cv}$ | 0.143109 | 0.55279 | 46948.8 |
| 439 | DisplMean~ $\psi_{ac}$+$\psi_{cv}$+$\psi_{ac}$:$\psi_{ce}$+$\psi_{ac}$:$\psi_{cv}$+$\psi_{ce}$:$\psi_{cv}$ | 0.300003 | 0.55203 | 47001 |
| 440 | DisplMean~ $\psi_{ac}$+$\psi_{ev}$+$\psi_{cv}$+$\psi_{ac}$:$\psi_{ce}$+$\psi_{ac}$:$\psi_{ev}$+$\psi_{ce}$:$\psi_{ev}$+$\psi_{ev}$:$\psi_{cv}$ | 0.153614 | 0.55137 | 47047.8 |
| 441 | DisplMean~ $\psi_{ac}$+$\psi_{ev}$+$\psi_{cv}$+$\psi_{ac}$:$\psi_{ce}$+$\psi_{ac}$:$\psi_{ev}$+$\psi_{ce}$:$\psi_{ev}$ | 0.15417 | 0.55029 | 47119.7 |
| 442 | DisplMean~ $\psi_{ce}$+$\psi_{ac}$:$\psi_{ev}$+$\psi_{ac}$:$\psi_{cv}$+$\psi_{ce}$:$\psi_{ev}$+$\psi_{ce}$:$\psi_{cv}$+$\psi_{ev}$:$\psi_{cv}$ | 0.175831 | 0.55012 | 47131.2 |
| 443 | DisplMean~ $\psi_{ac}$+$\psi_{cv}$+$\psi_{ac}$:$\psi_{ce}$+$\psi_{ac}$:$\psi_{cv}$+$\psi_{ce}$:$\psi_{ev}$ | 0.275621 | 0.54976 | 47155.1 |
| 444 | DisplMean~ $\psi_{cv}$+$\psi_{ac}$:$\psi_{ce}$+$\psi_{ac}$:$\psi_{cv}$+$\psi_{ce}$:$\psi_{ev}$ | 0.259467 | 0.54949 | 47172.1 |
| 445 | DisplMean~ $\psi_{ac}$+$\psi_{ce}$+$\psi_{cv}$+$\psi_{ac}$:$\psi_{cv}$ | 0.72105 | 0.54793 | 47277.4 |
| 446 | DisplMean~ $\psi_{ac}$+$\psi_{ce}$+$\psi_{cv}$+$\psi_{ac}$:$\psi_{cv}$+$\psi_{ce}$:$\psi_{cv}$ | 0.714423 | 0.54793 | 47278.2 |
| 447 | DisplMean~ $\psi_{ev}$+$\psi_{ac}$:$\psi_{ce}$+$\psi_{ac}$:$\psi_{ev}$+$\psi_{ac}$:$\psi_{cv}$+$\psi_{ce}$:$\psi_{ev}$+$\psi_{ce}$:$\psi_{cv}$+$\psi_{ev}$:$\psi_{cv}$ | 0.157443 | 0.54772 | 47294.5 |
| 448 | DisplMean~ $\psi_{ac}$+$\psi_{ev}$+$\psi_{cv}$+$\psi_{ac}$:$\psi_{ce}$+$\psi_{ce}$:$\psi_{ev}$+$\psi_{ev}$:$\psi_{cv}$ | 0.152791 | 0.54679 | 47356 |
| 449 | DisplMean~ $\psi_{ac}$+$\psi_{ev}$+$\psi_{cv}$+$\psi_{ac}$:$\psi_{ce}$+$\psi_{ce}$:$\psi_{ev}$ | 0.15294 | 0.5467 | 47361 |
| 450 | DisplMean~ $\psi_{ac}$+$\psi_{ce}$+$\psi_{ev}$+$\psi_{ac}$:$\psi_{ev}$+$\psi_{ac}$:$\psi_{cv}$+$\psi_{ce}$:$\psi_{ev}$+$\psi_{ev}$:$\psi_{cv}$ | 0.258261 | 0.54196 | 47679.9 |
| 451 | DisplMean~ $\psi_{ac}$+$\psi_{ev}$+$\psi_{ac}$:$\psi_{ce}$+$\psi_{ac}$:$\psi_{ev}$+$\psi_{ce}$:$\psi_{ev}$+$\psi_{ev}$:$\psi_{cv}$ | 0.15091 | 0.54192 | 47681.9 |
| 452 | DisplMean~ $\psi_{ce}$+$\psi_{ac}$:$\psi_{ev}$+$\psi_{ac}$:$\psi_{cv}$+$\psi_{ce}$:$\psi_{cv}$+$\psi_{ev}$:$\psi_{cv}$ | 0.164302 | 0.54142 | 47713.7 |
| 453 | DisplMean~ $\psi_{ac}$+$\psi_{ce}$+$\psi_{ac}$:$\psi_{ev}$+$\psi_{ac}$:$\psi_{cv}$+$\psi_{ce}$:$\psi_{ev}$+$\psi_{ev}$:$\psi_{cv}$ | 0.25274 | 0.54139 | 47717.2 |
| 454 | DisplMean~ $\psi_{ac}$+$\psi_{ce}$+$\psi_{ev}$+$\psi_{ac}$:$\psi_{ev}$+$\psi_{ac}$:$\psi_{cv}$+$\psi_{ev}$:$\psi_{cv}$ | 0.258581 | 0.54121 | 47728.6 |
| 455 | DisplMean~ $\psi_{ac}$+$\psi_{ev}$+$\psi_{ac}$:$\psi_{ce}$+$\psi_{ac}$:$\psi_{ev}$+$\psi_{ac}$:$\psi_{cv}$+$\psi_{ce}$:$\psi_{ev}$+$\psi_{ce}$:$\psi_{cv}$ | 0.117898 | 0.54092 | 47749 |

| | | | | |
|---|---|---|---|---|
| 456 | DisplMean~ $\psi_{ac}$+$\psi_{cv}$+$\psi_{ac}$:$\psi_{ce}$+$\psi_{ce}$:$\psi_{cv}$+$\psi_{ev}$:$\psi_{cv}$ | 0.153786 | 0.54079 | 47755.6 |
| 457 | DisplMean~ $\psi_{ce}$+$\psi_{cv}$+$\psi_{ac}$:$\psi_{ce}$+$\psi_{ac}$:$\psi_{ev}$+$\psi_{ce}$:$\psi_{ev}$+$\psi_{ce}$:$\psi_{cv}$+$\psi_{ev}$:$\psi_{cv}$ | 0.0976055 | 0.54044 | 47780.7 |
| 458 | DisplMean~ $\psi_{ac}$+$\psi_{ev}$+$\psi_{ac}$:$\psi_{ce}$+$\psi_{ce}$:$\psi_{ev}$+$\psi_{ev}$:$\psi_{cv}$ | 0.150324 | 0.54037 | 47783.8 |
| 459 | DisplMean~ $\psi_{ac}$+$\psi_{ac}$:$\psi_{ce}$+$\psi_{ac}$:$\psi_{cv}$+$\psi_{ce}$:$\psi_{ev}$+$\psi_{ev}$:$\psi_{cv}$ | 0.188951 | 0.54031 | 47787.7 |
| 460 | DisplMean~ $\psi_{ac}$+$\psi_{ce}$+$\psi_{ac}$:$\psi_{ev}$+$\psi_{ac}$:$\psi_{cv}$+$\psi_{ev}$:$\psi_{cv}$ | 0.252193 | 0.54024 | 47792.1 |
| 461 | DisplMean~ $\psi_{ev}$+$\psi_{cv}$+$\psi_{ac}$:$\psi_{ce}$+$\psi_{ac}$:$\psi_{ev}$+$\psi_{ce}$:$\psi_{ev}$+$\psi_{ce}$:$\psi_{cv}$+$\psi_{ev}$:$\psi_{cv}$ | 0.143638 | 0.53929 | 47856.9 |
| 462 | DisplMean~ $\psi_{ac}$+$\psi_{ce}$+$\psi_{ev}$+$\psi_{ac}$:$\psi_{cv}$+$\psi_{ce}$:$\psi_{ev}$+$\psi_{ev}$:$\psi_{cv}$ | 0.260526 | 0.53818 | 47929.2 |
| 463 | DisplMean~ $\psi_{ac}$+$\psi_{ce}$+$\psi_{ev}$+$\psi_{ac}$:$\psi_{cv}$+$\psi_{ev}$:$\psi_{cv}$ | 0.2606 | 0.53794 | 47944.2 |
| 464 | DisplMean~ $\psi_{ev}$+$\psi_{ac}$:$\psi_{ce}$+$\psi_{ac}$:$\psi_{ev}$+$\psi_{ac}$:$\psi_{cv}$+$\psi_{ce}$:$\psi_{ev}$+$\psi_{ev}$:$\psi_{cv}$ | 0.163907 | 0.53704 | 48004.6 |
| 465 | DisplMean~ $\psi_{ac}$+$\psi_{ev}$+$\psi_{ac}$:$\psi_{ce}$+$\psi_{ac}$:$\psi_{cv}$+$\psi_{ce}$:$\psi_{ev}$+$\psi_{ce}$:$\psi_{cv}$ | 0.116374 | 0.5369 | 48013.5 |
| 466 | DisplMean~ $\psi_{ce}$+$\psi_{ev}$+$\psi_{ac}$:$\psi_{ev}$+$\psi_{ac}$:$\psi_{cv}$+$\psi_{ce}$:$\psi_{ev}$+$\psi_{ev}$:$\psi_{cv}$ | 0.218851 | 0.53658 | 48034.9 |
| 467 | DisplMean~ $\psi_{ac}$+$\psi_{ce}$+$\psi_{ac}$:$\psi_{cv}$+$\psi_{ce}$:$\psi_{ev}$+$\psi_{ev}$:$\psi_{cv}$ | 0.281829 | 0.53646 | 48041.6 |
| 468 | DisplMean~ $\psi_{ac}$+$\psi_{ce}$+$\psi_{ac}$:$\psi_{cv}$+$\psi_{ev}$:$\psi_{cv}$ | 0.29075 | 0.53629 | 48051.7 |
| 469 | DisplMean~ $\psi_{ce}$+$\psi_{ev}$+$\psi_{ac}$:$\psi_{ev}$+$\psi_{ac}$:$\psi_{cv}$+$\psi_{ev}$:$\psi_{cv}$ | 0.216394 | 0.53489 | 48144.6 |
| 470 | DisplMean~ $\psi_{ac}$+$\psi_{cv}$+$\psi_{ac}$:$\psi_{cv}$+$\psi_{ce}$:$\psi_{cv}$ | 0.39475 | 0.53289 | 48274.2 |
| 471 | DisplMean~ $\psi_{ev}$+$\psi_{cv}$+$\psi_{ac}$:$\psi_{ce}$+$\psi_{ac}$:$\psi_{ev}$+$\psi_{ce}$:$\psi_{ev}$+$\psi_{ce}$:$\psi_{cv}$ | 0.145173 | 0.53289 | 48276.3 |
| 472 | DisplMean~ $\psi_{ac}$+$\psi_{cv}$+$\psi_{ac}$:$\psi_{ce}$+$\psi_{ac}$:$\psi_{ev}$+$\psi_{ce}$:$\psi_{ev}$+$\psi_{ev}$:$\psi_{cv}$ | 0.169754 | 0.53199 | 48334.8 |
| 473 | DisplMean~ $\psi_{ac}$+$\psi_{cv}$+$\psi_{ac}$:$\psi_{ce}$+$\psi_{ac}$:$\psi_{ev}$+$\psi_{ce}$:$\psi_{ev}$ | 0.169521 | 0.53196 | 48335.9 |
| 474 | DisplMean~ $\psi_{ce}$+$\psi_{cv}$+$\psi_{ac}$:$\psi_{cv}$+$\psi_{ce}$:$\psi_{cv}$ | 0.213313 | 0.53025 | 48446.3 |
| 475 | DisplMean~ $\psi_{ce}$+$\psi_{cv}$+$\psi_{ac}$:$\psi_{cv}$ | 0.215008 | 0.53011 | 48454 |
| 476 | DisplMean~ $\psi_{cv}$+$\psi_{ac}$:$\psi_{ce}$+$\psi_{ac}$:$\psi_{cv}$+$\psi_{ce}$:$\psi_{cv}$ | 0.187007 | 0.52979 | 48475.7 |
| 477 | DisplMean~ $\psi_{ce}$+$\psi_{ac}$:$\psi_{ev}$+$\psi_{ac}$:$\psi_{cv}$+$\psi_{ce}$:$\psi_{ev}$+$\psi_{ev}$:$\psi_{cv}$ | 0.186605 | 0.52892 | 48533.1 |
| 478 | DisplMean~ $\psi_{ac}$+$\psi_{ac}$:$\psi_{ce}$+$\psi_{ac}$:$\psi_{ev}$+$\psi_{ce}$:$\psi_{ev}$+$\psi_{ev}$:$\psi_{cv}$ | 0.164033 | 0.52633 | 48700.4 |
| 479 | DisplMean~ $\psi_{ce}$+$\psi_{cv}$+$\psi_{ac}$:$\psi_{ce}$+$\psi_{ac}$:$\psi_{ev}$+$\psi_{ce}$:$\psi_{ev}$+$\psi_{ev}$:$\psi_{cv}$ | 0.102918 | 0.5248 | 48799.6 |
| 480 | DisplMean~ $\psi_{ac}$+$\psi_{ev}$+$\psi_{ac}$:$\psi_{ev}$+$\psi_{ac}$:$\psi_{cv}$+$\psi_{ce}$:$\psi_{ev}$+$\psi_{ce}$:$\psi_{cv}$+$\psi_{ev}$:$\psi_{cv}$ | 0.219045 | 0.52377 | 48866.5 |
| 481 | DisplMean~ $\psi_{ac}$+$\psi_{ev}$+$\psi_{ac}$:$\psi_{ce}$+$\psi_{ac}$:$\psi_{ev}$+$\psi_{ac}$:$\psi_{cv}$+$\psi_{ce}$:$\psi_{ev}$ | 0.139764 | 0.52348 | 48883.8 |
| 482 | DisplMean~ $\psi_{ac}$+$\psi_{ev}$+$\psi_{ac}$:$\psi_{cv}$+$\psi_{ce}$:$\psi_{ev}$+$\psi_{ce}$:$\psi_{cv}$+$\psi_{ev}$:$\psi_{cv}$ | 0.219595 | 0.52265 | 48936.6 |

| | | | | |
|---|---|---|---|---|
| 483 | DisplMean~ $\psi_{ac}$+$\psi_{ev}$+$\psi_{ac}$:$\psi_{ev}$+$\psi_{ac}$:$\psi_{cv}$+$\psi_{ce}$:$\psi_{ev}$+$\psi_{ev}$:$\psi_{cv}$ | 0.224069 | 0.52235 | 48955.9 |
| 484 | DisplMean~ $\psi_{ac}$+$\psi_{ce}$+$\psi_{ev}$+$\psi_{ac}$:$\psi_{ev}$+$\psi_{ac}$:$\psi_{cv}$+$\psi_{ce}$:$\psi_{ev}$+$\psi_{ce}$:$\psi_{cv}$ | 0.199194 | 0.52228 | 48961.5 |
| 485 | DisplMean~ $\psi_{ac}$+$\psi_{ce}$+$\psi_{ev}$+$\psi_{ac}$:$\psi_{ev}$+$\psi_{ac}$:$\psi_{cv}$+$\psi_{ce}$:$\psi_{cv}$ | 0.199198 | 0.52225 | 48962.1 |
| 486 | DisplMean~ $\psi_{ac}$+$\psi_{ce}$+$\psi_{ev}$+$\psi_{ac}$:$\psi_{cv}$+$\psi_{ce}$:$\psi_{ev}$+$\psi_{ce}$:$\psi_{cv}$ | 0.19846 | 0.52213 | 48970.3 |
| 487 | DisplMean~ $\psi_{ac}$+$\psi_{ce}$+$\psi_{ev}$+$\psi_{ac}$:$\psi_{cv}$+$\psi_{ce}$:$\psi_{cv}$ | 0.198373 | 0.52206 | 48973.2 |
| 488 | DisplMean~ $\psi_{ac}$+$\psi_{ev}$+$\psi_{ac}$:$\psi_{cv}$+$\psi_{ce}$:$\psi_{ev}$+$\psi_{ev}$:$\psi_{cv}$ | 0.225821 | 0.52081 | 49052.8 |
| 489 | DisplMean~ $\psi_{cv}$+$\psi_{ac}$:$\psi_{cv}$+$\psi_{ce}$:$\psi_{cv}$ | 0.219976 | 0.52079 | 49052.6 |
| 490 | DisplMean~ $\psi_{ac}$+$\psi_{ac}$:$\psi_{ce}$+$\psi_{ac}$:$\psi_{ev}$+$\psi_{ac}$:$\psi_{cv}$+$\psi_{ce}$:$\psi_{ev}$+$\psi_{ce}$:$\psi_{cv}$ | 0.135052 | 0.52049 | 49074.7 |
| 491 | DisplMean~ $\psi_{ce}$+$\psi_{ac}$:$\psi_{ev}$+$\psi_{ac}$:$\psi_{cv}$+$\psi_{ev}$:$\psi_{cv}$ | 0.173845 | 0.52043 | 49076.5 |
| 492 | DisplMean~ $\psi_{ac}$+$\psi_{ac}$:$\psi_{ev}$+$\psi_{ac}$:$\psi_{cv}$+$\psi_{ce}$:$\psi_{ev}$+$\psi_{ce}$:$\psi_{cv}$+$\psi_{ev}$:$\psi_{cv}$ | 0.217313 | 0.52042 | 49079.2 |
| 493 | DisplMean~ $\psi_{ac}$+$\psi_{ac}$:$\psi_{cv}$+$\psi_{ce}$:$\psi_{ev}$+$\psi_{ce}$:$\psi_{cv}$+$\psi_{ev}$:$\psi_{cv}$ | 0.214967 | 0.51983 | 49115.5 |
| 494 | DisplMean~ $\psi_{ac}$+$\psi_{ac}$:$\psi_{ev}$+$\psi_{ac}$:$\psi_{cv}$+$\psi_{ce}$:$\psi_{ev}$+$\psi_{ev}$:$\psi_{cv}$ | 0.220472 | 0.51962 | 49128.4 |
| 495 | DisplMean~ $\psi_{ac}$+$\psi_{ac}$:$\psi_{cv}$+$\psi_{ce}$:$\psi_{ev}$+$\psi_{ev}$:$\psi_{cv}$ | 0.218152 | 0.5195 | 49135.3 |
| 496 | DisplMean~ $\psi_{ac}$+$\psi_{ev}$+$\psi_{ac}$:$\psi_{ce}$+$\psi_{ac}$:$\psi_{cv}$+$\psi_{ce}$:$\psi_{ev}$ | 0.13804 | 0.51926 | 49151.8 |
| 497 | DisplMean~ $\psi_{ac}$+$\psi_{ce}$+$\psi_{ac}$:$\psi_{ev}$+$\psi_{ac}$:$\psi_{cv}$+$\psi_{ce}$:$\psi_{ev}$+$\psi_{ce}$:$\psi_{cv}$ | 0.198091 | 0.51872 | 49186.8 |
| 498 | DisplMean~ $\psi_{ac}$+$\psi_{ce}$+$\psi_{ac}$:$\psi_{ev}$+$\psi_{ac}$:$\psi_{cv}$+$\psi_{ce}$:$\psi_{cv}$ | 0.198664 | 0.5183 | 49212 |
| 499 | DisplMean~ $\psi_{ev}$+$\psi_{ac}$:$\psi_{ce}$+$\psi_{ac}$:$\psi_{ev}$+$\psi_{ce}$:$\psi_{ev}$+$\psi_{ce}$:$\psi_{cv}$+$\psi_{ev}$:$\psi_{cv}$ | 0.139932 | 0.51809 | 49226.7 |
| 500 | DisplMean~ $\psi_{ac}$+$\psi_{ev}$+$\psi_{ac}$:$\psi_{ce}$+$\psi_{ac}$:$\psi_{ev}$+$\psi_{ce}$:$\psi_{ev}$ | 0.142384 | 0.51797 | 49233.3 |
| 501 | DisplMean~ $\psi_{ac}$+$\psi_{ev}$+$\psi_{ac}$:$\psi_{ce}$+$\psi_{ac}$:$\psi_{ev}$+$\psi_{ce}$:$\psi_{ev}$+$\psi_{ce}$:$\psi_{cv}$ | 0.142458 | 0.51796 | 49234.9 |
| 502 | DisplMean~ $\psi_{ce}$+$\psi_{ev}$+$\psi_{ac}$:$\psi_{ce}$+$\psi_{ac}$:$\psi_{cv}$+$\psi_{ce}$:$\psi_{ev}$+$\psi_{ce}$:$\psi_{cv}$+$\psi_{ev}$:$\psi_{cv}$ | 0.106959 | 0.51785 | 49242.5 |
| 503 | DisplMean~ $\psi_{ce}$+$\psi_{ev}$+$\psi_{ac}$:$\psi_{ce}$+$\psi_{ac}$:$\psi_{cv}$+$\psi_{ce}$:$\psi_{cv}$+$\psi_{ev}$:$\psi_{cv}$ | 0.106999 | 0.51752 | 49262.8 |
| 504 | DisplMean~ $\psi_{ev}$+$\psi_{ac}$:$\psi_{ev}$+$\psi_{ac}$:$\psi_{cv}$+$\psi_{ce}$:$\psi_{ev}$+$\psi_{ce}$:$\psi_{cv}$+$\psi_{ev}$:$\psi_{cv}$ | 0.190245 | 0.51449 | 49453.5 |
| 505 | DisplMean~ $\psi_{ac}$+$\psi_{ce}$+$\psi_{ev}$+$\psi_{cv}$+$\psi_{ac}$:$\psi_{ev}$+$\psi_{ce}$:$\psi_{ev}$ | 0.259699 | 0.51381 | 49495.9 |
| 506 | DisplMean~ $\psi_{ac}$+$\psi_{ce}$+$\psi_{ev}$+$\psi_{cv}$+$\psi_{ac}$:$\psi_{ev}$+$\psi_{ce}$:$\psi_{ev}$+$\psi_{ce}$:$\psi_{cv}$ | 0.258199 | 0.51381 | 49496.8 |
| 507 | DisplMean~ $\psi_{ac}$+$\psi_{ce}$+$\psi_{ev}$+$\psi_{cv}$+$\psi_{ac}$:$\psi_{ev}$+$\psi_{ce}$:$\psi_{ev}$+$\psi_{ev}$:$\psi_{cv}$ | 0.259902 | 0.51381 | 49496.7 |
| 508 | DisplMean~ $\psi_{ac}$+$\psi_{ce}$+$\psi_{ev}$+$\psi_{cv}$+$\psi_{ac}$:$\psi_{ev}$+$\psi_{ce}$:$\psi_{ev}$+$\psi_{ce}$:$\psi_{cv}$+$\psi_{ev}$:$\psi_{cv}$ | 0.258403 | 0.51381 | 49497.6 |
| 509 | DisplMean~ $\psi_{ac}$+$\psi_{ce}$+$\psi_{ev}$+$\psi_{cv}$+$\psi_{ac}$:$\psi_{ev}$+$\psi_{ev}$:$\psi_{cv}$ | 0.259939 | 0.51379 | 49497.1 |

| | | | | |
|---|---|---|---|---|
| 510 | DisplMean~ $\psi_{ac}+\psi_{ce}+\psi_{ev}+\psi_{cv}+\psi_{ac}:\psi_{ev}+\psi_{ce}:\psi_{cv}+\psi_{ev}:\psi_{cv}$ | 0.258439 | 0.51379 | 49498.1 |
| 511 | DisplMean~ $\psi_{ac}+\psi_{ce}+\psi_{ev}+\psi_{cv}+\psi_{ac}:\psi_{ev}$ | 0.259715 | 0.51378 | 49496.8 |
| 512 | DisplMean~ $\psi_{ac}+\psi_{ce}+\psi_{ev}+\psi_{cv}+\psi_{ac}:\psi_{ev}+\psi_{ce}:\psi_{cv}$ | 0.258212 | 0.51378 | 49497.7 |
| 513 | DisplMean~ $\psi_{ev}+\psi_{ac}:\psi_{ev}+\psi_{ac}:\psi_{cv}+\psi_{ce}:\psi_{ev}+\psi_{ev}:\psi_{cv}$ | 0.193563 | 0.51365 | 49504.7 |
| 514 | DisplMean~ $\psi_{ac}+\psi_{ev}+\psi_{ac}:\psi_{ce}+\psi_{ce}:\psi_{ev}$ | 0.140621 | 0.5136 | 49507.3 |
| 515 | DisplMean~ $\psi_{ac}+\psi_{ev}+\psi_{ac}:\psi_{ce}+\psi_{ce}:\psi_{ev}+\psi_{ce}:\psi_{cv}$ | 0.140713 | 0.51359 | 49508.8 |
| 516 | DisplMean~ $\psi_{ac}+\psi_{ce}+\psi_{ev}+\psi_{cv}+\psi_{ce}:\psi_{ev}$ | 0.257681 | 0.51358 | 49509.5 |
| 517 | DisplMean~ $\psi_{ac}+\psi_{ce}+\psi_{ev}+\psi_{cv}+\psi_{ce}:\psi_{ev}+\psi_{ce}:\psi_{cv}$ | 0.25615 | 0.51358 | 49510.4 |
| 518 | DisplMean~ $\psi_{ac}+\psi_{ce}+\psi_{ev}+\psi_{cv}+\psi_{ce}:\psi_{ev}+\psi_{ev}:\psi_{cv}$ | 0.257674 | 0.51356 | 49511.4 |
| 519 | DisplMean~ $\psi_{ac}+\psi_{ce}+\psi_{ev}+\psi_{cv}+\psi_{ce}:\psi_{ev}+\psi_{ce}:\psi_{cv}+\psi_{ev}:\psi_{cv}$ | 0.256143 | 0.51356 | 49512.3 |
| 520 | DisplMean~ $\psi_{ac}+\psi_{ce}+\psi_{ev}+\psi_{cv}$ | 0.257471 | 0.5135 | 49513.5 |
| 521 | DisplMean~ $\psi_{ac}+\psi_{ce}+\psi_{ev}+\psi_{cv}+\psi_{ce}:\psi_{cv}$ | 0.255933 | 0.5135 | 49514.3 |
| 522 | DisplMean~ $\psi_{ac}+\psi_{ce}+\psi_{ev}+\psi_{cv}+\psi_{ev}:\psi_{cv}$ | 0.257467 | 0.51348 | 49515.4 |
| 523 | DisplMean~ $\psi_{ac}+\psi_{ce}+\psi_{ev}+\psi_{cv}+\psi_{ce}:\psi_{cv}+\psi_{ev}:\psi_{cv}$ | 0.255929 | 0.51348 | 49516.3 |
| 524 | DisplMean~ $\psi_{ac}+\psi_{ce}+\psi_{cv}+\psi_{ac}:\psi_{ev}+\psi_{ce}:\psi_{ev}+\psi_{ev}:\psi_{cv}$ | 0.24759 | 0.51037 | 49710.7 |
| 525 | DisplMean~ $\psi_{ac}+\psi_{ce}+\psi_{cv}+\psi_{ac}:\psi_{ev}+\psi_{ce}:\psi_{ev}+\psi_{ce}:\psi_{cv}+\psi_{ev}:\psi_{cv}$ | 0.246304 | 0.51037 | 49711.4 |
| 526 | DisplMean~ $\psi_{ac}+\psi_{ce}+\psi_{cv}+\psi_{ac}:\psi_{ev}+\psi_{ce}:\psi_{ev}+\psi_{ce}:\psi_{cv}$ | 0.246636 | 0.51035 | 49712 |
| 527 | DisplMean~ $\psi_{ac}+\psi_{ce}+\psi_{cv}+\psi_{ac}:\psi_{ev}+\psi_{ce}:\psi_{ev}$ | 0.247913 | 0.51034 | 49711.3 |
| 528 | DisplMean~ $\psi_{ac}+\psi_{ce}+\psi_{cv}+\psi_{ac}:\psi_{ev}+\psi_{ev}:\psi_{cv}$ | 0.248271 | 0.50996 | 49735.4 |
| 529 | DisplMean~ $\psi_{ac}+\psi_{ce}+\psi_{cv}+\psi_{ac}:\psi_{ev}+\psi_{ce}:\psi_{cv}+\psi_{ev}:\psi_{cv}$ | 0.247002 | 0.50996 | 49736.1 |
| 530 | DisplMean~ $\psi_{ac}+\psi_{ce}+\psi_{cv}+\psi_{ac}:\psi_{ev}+\psi_{ce}:\psi_{cv}$ | 0.247274 | 0.50995 | 49735.8 |
| 531 | DisplMean~ $\psi_{ac}+\psi_{ce}+\psi_{cv}+\psi_{ac}:\psi_{ev}$ | 0.248535 | 0.50994 | 49735.1 |
| 532 | DisplMean~ $\psi_{ac}+\psi_{ce}+\psi_{ev}+\psi_{ce}:\psi_{ev}+\psi_{ce}:\psi_{cv}+\psi_{ev}:\psi_{cv}$ | 0.228007 | 0.50963 | 49756.9 |
| 533 | DisplMean~ $\psi_{ac}+\psi_{ce}+\psi_{ev}+\psi_{ac}:\psi_{ev}+\psi_{ce}:\psi_{ev}+\psi_{ce}:\psi_{cv}+\psi_{ev}:\psi_{cv}$ | 0.227982 | 0.50961 | 49758.8 |
| 534 | DisplMean~ $\psi_{ac}+\psi_{ce}+\psi_{ev}+\psi_{ce}:\psi_{cv}+\psi_{ev}:\psi_{cv}$ | 0.227733 | 0.50951 | 49763.2 |
| 535 | DisplMean~ $\psi_{ac}+\psi_{ce}+\psi_{ev}+\psi_{ac}:\psi_{ev}+\psi_{ce}:\psi_{cv}+\psi_{ev}:\psi_{cv}$ | 0.227767 | 0.50949 | 49765.1 |
| 536 | DisplMean~ $\psi_{ac}+\psi_{cv}+\psi_{ac}:\psi_{ce}+\psi_{ac}:\psi_{cv}$ | 0.376339 | 0.50945 | 49766.1 |

| | | | | |
|---|---|---|---|---|
| 537 | DisplMean~ $\psi_{ce}$+$\psi_{ac}$:$\psi_{ce}$+$\psi_{ac}$:$\psi_{cv}$+$\psi_{ce}$:$\psi_{ev}$+$\psi_{ce}$:$\psi_{cv}$+$\psi_{ev}$:$\psi_{cv}$ | 0.105981 | 0.5076 | 49882.7 |
| 538 | DisplMean~ $\psi_{ac}$+$\psi_{ce}$+$\psi_{ac}$:$\psi_{ev}$+$\psi_{ce}$:$\psi_{ev}$+$\psi_{ce}$:$\psi_{cv}$+$\psi_{ev}$:$\psi_{cv}$ | 0.224644 | 0.5072 | 49907.4 |
| 539 | DisplMean~ $\psi_{ac}$+$\psi_{ce}$+$\psi_{ev}$+$\psi_{ac}$:$\psi_{ev}$+$\psi_{ce}$:$\psi_{ev}$+$\psi_{ev}$:$\psi_{cv}$ | 0.233311 | 0.50711 | 49912.9 |
| 540 | DisplMean~ $\psi_{ac}$+$\psi_{ce}$+$\psi_{ev}$+$\psi_{ce}$:$\psi_{ev}$+$\psi_{ev}$:$\psi_{cv}$ | 0.233935 | 0.50702 | 49917.3 |
| 541 | DisplMean~ $\psi_{ac}$+$\psi_{ce}$+$\psi_{ev}$+$\psi_{ac}$:$\psi_{ev}$+$\psi_{ev}$:$\psi_{cv}$ | 0.233223 | 0.50692 | 49923.7 |
| 542 | DisplMean~ $\psi_{ac}$+$\psi_{ce}$+$\psi_{ev}$+$\psi_{ev}$:$\psi_{cv}$ | 0.233718 | 0.50687 | 49925.4 |
| 543 | DisplMean~ $\psi_{ac}$+$\psi_{ce}$+$\psi_{ac}$:$\psi_{ev}$+$\psi_{ce}$:$\psi_{cv}$+$\psi_{ev}$:$\psi_{cv}$ | 0.224954 | 0.50666 | 49939.8 |
| 544 | DisplMean~ $\psi_{ac}$+$\psi_{ce}$+$\psi_{ac}$:$\psi_{ev}$+$\psi_{ce}$:$\psi_{ev}$+$\psi_{ev}$:$\psi_{cv}$ | 0.228673 | 0.50515 | 50032.6 |
| 545 | DisplMean~ $\psi_{ac}$+$\psi_{ce}$+$\psi_{ev}$+$\psi_{ac}$:$\psi_{ev}$+$\psi_{ce}$:$\psi_{ev}$+$\psi_{ce}$:$\psi_{cv}$ | 0.2076 | 0.50489 | 50049.8 |
| 546 | DisplMean~ $\psi_{ac}$+$\psi_{ce}$+$\psi_{ev}$+$\psi_{ac}$:$\psi_{ev}$+$\psi_{ce}$:$\psi_{cv}$ | 0.20759 | 0.50486 | 50050.7 |
| 547 | DisplMean~ $\psi_{ac}$+$\psi_{ce}$+$\psi_{ev}$+$\psi_{ce}$:$\psi_{ev}$+$\psi_{ce}$:$\psi_{cv}$ | 0.206304 | 0.50467 | 50062.3 |
| 548 | DisplMean~ $\psi_{ac}$+$\psi_{ce}$+$\psi_{ev}$+$\psi_{ce}$:$\psi_{cv}$ | 0.206135 | 0.50459 | 50066.2 |
| 549 | DisplMean~ $\psi_{ac}$+$\psi_{ce}$+$\psi_{ac}$:$\psi_{ev}$+$\psi_{ev}$:$\psi_{cv}$ | 0.228972 | 0.50454 | 50069.4 |
| 550 | DisplMean~ $\psi_{ac}$+$\psi_{ac}$:$\psi_{ce}$+$\psi_{ac}$:$\psi_{ev}$+$\psi_{ac}$:$\psi_{cv}$+$\psi_{ce}$:$\psi_{ev}$ | 0.156297 | 0.50415 | 50094.4 |
| 551 | DisplMean~ $\psi_{ac}$+$\psi_{ev}$+$\psi_{cv}$+$\psi_{ac}$:$\psi_{ev}$+$\psi_{ce}$:$\psi_{ev}$+$\psi_{ce}$:$\psi_{cv}$+$\psi_{ev}$:$\psi_{cv}$ | 0.237213 | 0.50395 | 50108.6 |
| 552 | DisplMean~ $\psi_{ac}$+$\psi_{ev}$+$\psi_{cv}$+$\psi_{ac}$:$\psi_{ev}$+$\psi_{ce}$:$\psi_{ev}$+$\psi_{ce}$:$\psi_{cv}$ | 0.236872 | 0.50383 | 50115 |
| 553 | DisplMean~ $\psi_{ac}$+$\psi_{ev}$+$\psi_{cv}$+$\psi_{ce}$:$\psi_{ev}$+$\psi_{ce}$:$\psi_{cv}$ | 0.234103 | 0.50304 | 50162.2 |
| 554 | DisplMean~ $\psi_{ac}$+$\psi_{ev}$+$\psi_{cv}$+$\psi_{ce}$:$\psi_{ev}$+$\psi_{ce}$:$\psi_{cv}$+$\psi_{ev}$:$\psi_{cv}$ | 0.234104 | 0.50303 | 50164.1 |
| 555 | DisplMean~ $\psi_{ce}$+$\psi_{ac}$:$\psi_{ce}$+$\psi_{ac}$:$\psi_{cv}$+$\psi_{ce}$:$\psi_{cv}$+$\psi_{ev}$:$\psi_{cv}$ | 0.105789 | 0.50285 | 50173.9 |
| 556 | DisplMean~ $\psi_{cv}$+$\psi_{ac}$:$\psi_{ce}$+$\psi_{ac}$:$\psi_{cv}$ | 0.233821 | 0.50214 | 50215.4 |
| 557 | DisplMean~ $\psi_{ac}$+$\psi_{ce}$+$\psi_{ce}$:$\psi_{ev}$+$\psi_{ce}$:$\psi_{cv}$+$\psi_{ev}$:$\psi_{cv}$ | 0.20951 | 0.50181 | 50237.5 |
| 558 | DisplMean~ $\psi_{ac}$+$\psi_{ce}$+$\psi_{cv}$+$\psi_{ce}$:$\psi_{ev}$+$\psi_{ce}$:$\psi_{cv}$+$\psi_{ev}$:$\psi_{cv}$ | 0.209675 | 0.5018 | 50239.4 |
| 559 | DisplMean~ $\psi_{ac}$+$\psi_{ce}$+$\psi_{cv}$+$\psi_{ce}$:$\psi_{ev}$+$\psi_{ev}$:$\psi_{cv}$ | 0.210959 | 0.50177 | 50240.1 |
| 560 | DisplMean~ $\psi_{ac}$+$\psi_{ce}$+$\psi_{ce}$:$\psi_{ev}$+$\psi_{ev}$:$\psi_{cv}$ | 0.211277 | 0.50171 | 50242.6 |
| 561 | DisplMean~ $\psi_{ac}$+$\psi_{ce}$+$\psi_{ac}$:$\psi_{ev}$+$\psi_{ce}$:$\psi_{ev}$+$\psi_{ce}$:$\psi_{cv}$ | 0.20617 | 0.50161 | 50250.1 |
| 562 | DisplMean~ $\psi_{ac}$+$\psi_{ce}$+$\psi_{ac}$:$\psi_{ev}$+$\psi_{ce}$:$\psi_{cv}$ | 0.206823 | 0.50119 | 50274.2 |
| 563 | DisplMean~ $\psi_{ev}$+$\psi_{cv}$+$\psi_{ac}$:$\psi_{ce}$+$\psi_{ac}$:$\psi_{ev}$+$\psi_{ce}$:$\psi_{cv}$+$\psi_{ev}$:$\psi_{cv}$ | 0.152767 | 0.50106 | 50284.7 |

| | | | | |
|---|---|---|---|---|
| 564 | DisplMean~ $\psi_{ac}$+$\psi_{cv}$+$\psi_{ac}$:$\psi_{cv}$+$\psi_{ce}$:$\psi_{ev}$ | 0.448367 | 0.50041 | 50322.1 |
| 565 | DisplMean~ $\psi_{ac}$+$\psi_{ev}$+$\psi_{ac}$:$\psi_{ce}$+$\psi_{ac}$:$\psi_{ev}$+$\psi_{ac}$:$\psi_{cv}$+$\psi_{ce}$:$\psi_{cv}$+$\psi_{ev}$:$\psi_{cv}$ | 0.192263 | 0.49916 | 50401 |
| 566 | DisplMean~ $\psi_{ac}$+$\psi_{ac}$:$\psi_{ce}$+$\psi_{ac}$:$\psi_{ev}$+$\psi_{ac}$:$\psi_{cv}$+$\psi_{ce}$:$\psi_{cv}$+$\psi_{ev}$:$\psi_{cv}$ | 0.191269 | 0.49875 | 50425.3 |
| 567 | DisplMean~ $\psi_{ac}$+$\psi_{cv}$+$\psi_{ac}$:$\psi_{cv}$ | 0.573883 | 0.4986 | 50431.1 |
| 568 | DisplMean~ $\psi_{ac}$+$\psi_{ac}$:$\psi_{ce}$+$\psi_{ac}$:$\psi_{ev}$+$\psi_{ce}$:$\psi_{ev}$+$\psi_{ce}$:$\psi_{cv}$ | 0.160985 | 0.49851 | 50438.7 |
| 569 | DisplMean~ $\psi_{ac}$+$\psi_{ac}$:$\psi_{ce}$+$\psi_{ac}$:$\psi_{ev}$+$\psi_{ce}$:$\psi_{ev}$ | 0.161032 | 0.4985 | 50438 |
| 570 | DisplMean~ $\psi_{ac}$+$\psi_{cv}$+$\psi_{ac}$:$\psi_{ev}$+$\psi_{ce}$:$\psi_{ev}$+$\psi_{ce}$:$\psi_{cv}$ | 0.233265 | 0.49829 | 50452.1 |
| 571 | DisplMean~ $\psi_{ac}$+$\psi_{cv}$+$\psi_{ac}$:$\psi_{ev}$+$\psi_{ce}$:$\psi_{ev}$+$\psi_{ce}$:$\psi_{cv}$+$\psi_{ev}$:$\psi_{cv}$ | 0.233185 | 0.49828 | 50453.9 |
| 572 | DisplMean~ $\psi_{ac}$+$\psi_{ev}$+$\psi_{cv}$+$\psi_{ac}$:$\psi_{ev}$+$\psi_{ce}$:$\psi_{ev}$+$\psi_{ev}$:$\psi_{cv}$ | 0.211965 | 0.49789 | 50477.4 |
| 573 | DisplMean~ $\psi_{ac}$+$\psi_{ev}$+$\psi_{cv}$+$\psi_{ac}$:$\psi_{ev}$+$\psi_{ce}$:$\psi_{ev}$ | 0.21144 | 0.49763 | 50492 |
| 574 | DisplMean~ $\psi_{ac}$+$\psi_{ce}$+$\psi_{ac}$:$\psi_{cv}$+$\psi_{ce}$:$\psi_{ev}$+$\psi_{ce}$:$\psi_{cv}$ | 0.168784 | 0.49692 | 50535.1 |
| 575 | DisplMean~ $\psi_{ce}$+$\psi_{ev}$+$\psi_{ac}$:$\psi_{cv}$+$\psi_{ce}$:$\psi_{ev}$+$\psi_{ce}$:$\psi_{cv}$+$\psi_{ev}$:$\psi_{cv}$ | 0.127352 | 0.49641 | 50567.2 |
| 576 | DisplMean~ $\psi_{ac}$+$\psi_{ev}$+$\psi_{cv}$+$\psi_{ce}$:$\psi_{ev}$ | 0.207701 | 0.49632 | 50570.3 |
| 577 | DisplMean~ $\psi_{ac}$+$\psi_{ev}$+$\psi_{cv}$+$\psi_{ce}$:$\psi_{ev}$+$\psi_{ev}$:$\psi_{cv}$ | 0.207707 | 0.49632 | 50571.7 |
| 578 | DisplMean~ $\psi_{ac}$+$\psi_{ev}$+$\psi_{cv}$+$\psi_{ac}$:$\psi_{ev}$+$\psi_{ce}$:$\psi_{cv}$+$\psi_{ev}$:$\psi_{cv}$ | 0.230403 | 0.49624 | 50577.5 |
| 579 | DisplMean~ $\psi_{ac}$+$\psi_{ev}$+$\psi_{cv}$+$\psi_{ac}$:$\psi_{ev}$+$\psi_{ce}$:$\psi_{cv}$ | 0.23028 | 0.49623 | 50577.1 |
| 580 | DisplMean~ $\psi_{ce}$+$\psi_{ev}$+$\psi_{ac}$:$\psi_{cv}$+$\psi_{ce}$:$\psi_{cv}$+$\psi_{ev}$:$\psi_{cv}$ | 0.127316 | 0.49607 | 50586.5 |
| 581 | DisplMean~ $\psi_{ac}$+$\psi_{ev}$+$\psi_{cv}$+$\psi_{ce}$:$\psi_{cv}$ | 0.229083 | 0.49594 | 50593.7 |
| 582 | DisplMean~ $\psi_{ac}$+$\psi_{ev}$+$\psi_{cv}$+$\psi_{ce}$:$\psi_{cv}$+$\psi_{ev}$:$\psi_{cv}$ | 0.22908 | 0.49592 | 50595.6 |
| 583 | DisplMean~ $\psi_{ce}$+$\psi_{ac}$:$\psi_{ce}$+$\psi_{ac}$:$\psi_{ev}$+$\psi_{ce}$:$\psi_{ev}$+$\psi_{ce}$:$\psi_{cv}$+$\psi_{ev}$:$\psi_{cv}$ | 0.0928543 | 0.49573 | 50608 |
| 584 | DisplMean~ $\psi_{ev}$+$\psi_{cv}$+$\psi_{ac}$:$\psi_{ce}$+$\psi_{ac}$:$\psi_{ev}$+$\psi_{ce}$:$\psi_{cv}$ | 0.15399 | 0.49535 | 50630.1 |
| 585 | DisplMean~ $\psi_{ac}$+$\psi_{ev}$+$\psi_{ac}$:$\psi_{ce}$+$\psi_{ac}$:$\psi_{cv}$+$\psi_{ce}$:$\psi_{cv}$+$\psi_{ev}$:$\psi_{cv}$ | 0.194396 | 0.49416 | 50702.7 |
| 586 | DisplMean~ $\psi_{ce}$+$\psi_{ev}$+$\psi_{ac}$:$\psi_{ev}$+$\psi_{ac}$:$\psi_{cv}$+$\psi_{ce}$:$\psi_{ev}$+$\psi_{ce}$:$\psi_{cv}$ | 0.15823 | 0.49409 | 50706.7 |
| 587 | DisplMean~ $\psi_{ce}$+$\psi_{ev}$+$\psi_{ac}$:$\psi_{ce}$+$\psi_{ac}$:$\psi_{cv}$+$\psi_{ce}$:$\psi_{ev}$+$\psi_{ev}$:$\psi_{cv}$ | 0.103936 | 0.4935 | 50742.3 |
| 588 | DisplMean~ $\psi_{ce}$+$\psi_{ev}$+$\psi_{ac}$:$\psi_{ce}$+$\psi_{ac}$:$\psi_{cv}$+$\psi_{ev}$:$\psi_{cv}$ | 0.104003 | 0.49305 | 50768.4 |
| 589 | DisplMean~ $\psi_{ce}$+$\psi_{ev}$+$\psi_{ac}$:$\psi_{ev}$+$\psi_{ac}$:$\psi_{cv}$+$\psi_{ce}$:$\psi_{cv}$ | 0.157164 | 0.49298 | 50772.8 |
| 590 | DisplMean~ $\psi_{ac}$:$\psi_{ce}$+$\psi_{ac}$:$\psi_{ev}$+$\psi_{ac}$:$\psi_{cv}$+$\psi_{ce}$:$\psi_{ev}$+$\psi_{ce}$:$\psi_{cv}$+$\psi_{ev}$:$\psi_{cv}$ | 0.14668 | 0.49291 | 50778 |

| | | | | |
|---|---|---|---|---|
| 591 | DisplMean~ $\psi_{ac}$+$\psi_{ev}$+$\psi_{ac}$:$\psi_{ce}$+$\psi_{ac}$:$\psi_{ev}$+$\psi_{ce}$:$\psi_{cv}$+$\psi_{ev}$:$\psi_{cv}$ | 0.172741 | 0.49275 | 50787.5 |
| 592 | DisplMean~ $\psi_{ac}$+$\psi_{cv}$+$\psi_{ac}$:$\psi_{ev}$+$\psi_{ce}$:$\psi_{cv}$+$\psi_{ev}$:$\psi_{cv}$ | 0.226993 | 0.49257 | 50797.5 |
| 593 | DisplMean~ $\psi_{ac}$+$\psi_{cv}$+$\psi_{ac}$:$\psi_{ev}$+$\psi_{ce}$:$\psi_{cv}$ | 0.227171 | 0.49256 | 50797 |
| 594 | DisplMean~ $\psi_{ac}$+$\psi_{ce}$+$\psi_{cv}$+$\psi_{ce}$:$\psi_{cv}$+$\psi_{ev}$:$\psi_{cv}$ | 0.196794 | 0.49243 | 50806 |
| 595 | DisplMean~ $\psi_{ac}$+$\psi_{ev}$+$\psi_{ac}$:$\psi_{ev}$+$\psi_{ac}$:$\psi_{cv}$+$\psi_{ce}$:$\psi_{cv}$+$\psi_{ev}$:$\psi_{cv}$ | 0.212703 | 0.49242 | 50807.4 |
| 596 | DisplMean~ $\psi_{ac}$+$\psi_{ce}$+$\psi_{cv}$+$\psi_{ev}$:$\psi_{cv}$ | 0.198041 | 0.49239 | 50807.3 |
| 597 | DisplMean~ $\psi_{ac}$+$\psi_{ac}$:$\psi_{ce}$+$\psi_{ac}$:$\psi_{ev}$+$\psi_{ce}$:$\psi_{cv}$+$\psi_{ev}$:$\psi_{cv}$ | 0.171809 | 0.49203 | 50829.6 |
| 598 | DisplMean~ $\psi_{ac}$+$\psi_{ac}$:$\psi_{ev}$+$\psi_{ac}$:$\psi_{cv}$+$\psi_{ce}$:$\psi_{cv}$+$\psi_{ev}$:$\psi_{cv}$ | 0.211332 | 0.49197 | 50833.5 |
| 599 | DisplMean~ $\psi_{ev}$+$\psi_{ac}$:$\psi_{ce}$+$\psi_{ac}$:$\psi_{ev}$+$\psi_{ac}$:$\psi_{cv}$+$\psi_{ce}$:$\psi_{cv}$+$\psi_{ev}$:$\psi_{cv}$ | 0.181434 | 0.49037 | 50930 |
| 600 | DisplMean~ $\psi_{ac}$+$\psi_{cv}$+$\psi_{ac}$:$\psi_{ev}$+$\psi_{ce}$:$\psi_{ev}$ | 0.211703 | 0.49022 | 50937.3 |
| 601 | DisplMean~ $\psi_{ac}$+$\psi_{cv}$+$\psi_{ac}$:$\psi_{ev}$+$\psi_{ce}$:$\psi_{ev}$+$\psi_{ev}$:$\psi_{cv}$ | 0.211794 | 0.4902 | 50939.1 |
| 602 | DisplMean~ $\psi_{ac}$+$\psi_{ev}$+$\psi_{ce}$:$\psi_{ev}$+$\psi_{ce}$:$\psi_{cv}$+$\psi_{ev}$:$\psi_{cv}$ | 0.204668 | 0.49006 | 50947.7 |
| 603 | DisplMean~ $\psi_{ac}$+$\psi_{ev}$+$\psi_{ac}$:$\psi_{ev}$+$\psi_{ce}$:$\psi_{ev}$+$\psi_{ce}$:$\psi_{cv}$+$\psi_{ev}$:$\psi_{cv}$ | 0.204827 | 0.49006 | 50948.9 |
| 604 | DisplMean~ $\psi_{ac}$+$\psi_{ce}$+$\psi_{ce}$:$\psi_{cv}$+$\psi_{ev}$:$\psi_{cv}$ | 0.201643 | 0.4897 | 50968.2 |
| 605 | DisplMean~ $\psi_{ac}$+$\psi_{ev}$+$\psi_{ac}$:$\psi_{ce}$+$\psi_{ce}$:$\psi_{cv}$+$\psi_{ev}$:$\psi_{cv}$ | 0.176951 | 0.48954 | 50978.6 |
| 606 | DisplMean~ $\psi_{ce}$+$\psi_{ac}$:$\psi_{cv}$+$\psi_{ce}$:$\psi_{ev}$+$\psi_{ce}$:$\psi_{cv}$+$\psi_{ev}$:$\psi_{cv}$ | 0.124452 | 0.48928 | 50994.6 |
| 607 | DisplMean~ $\psi_{ac}$+$\psi_{ev}$+$\psi_{ac}$:$\psi_{ev}$+$\psi_{ce}$:$\psi_{ev}$+$\psi_{ev}$:$\psi_{cv}$ | 0.199601 | 0.48909 | 51005.3 |
| 608 | DisplMean~ $\psi_{ac}$+$\psi_{ev}$+$\psi_{ce}$:$\psi_{ev}$+$\psi_{ev}$:$\psi_{cv}$ | 0.198695 | 0.48898 | 51011.2 |
| 609 | DisplMean~ $\psi_{cv}$+$\psi_{ac}$:$\psi_{cv}$+$\psi_{ce}$:$\psi_{ev}$ | 0.206952 | 0.48885 | 51017.7 |
| 610 | DisplMean~ $\psi_{ac}$:$\psi_{ce}$+$\psi_{ac}$:$\psi_{ev}$+$\psi_{ac}$:$\psi_{cv}$+$\psi_{ce}$:$\psi_{ev}$+$\psi_{ev}$:$\psi_{cv}$ | 0.15038 | 0.48809 | 51065 |
| 611 | DisplMean~ $\psi_{ac}$+$\psi_{ev}$+$\psi_{ac}$:$\psi_{cv}$+$\psi_{ce}$:$\psi_{cv}$+$\psi_{ev}$:$\psi_{cv}$ | 0.214182 | 0.48804 | 51068.2 |
| 612 | DisplMean~ $\psi_{ac}$:$\psi_{ev}$+$\psi_{ac}$:$\psi_{cv}$+$\psi_{ce}$:$\psi_{ev}$+$\psi_{ce}$:$\psi_{cv}$+$\psi_{ev}$:$\psi_{cv}$ | 0.158832 | 0.48774 | 51085.9 |
| 613 | DisplMean~ $\psi_{ac}$+$\psi_{ce}$+$\psi_{cv}$+$\psi_{ce}$:$\psi_{ev}$+$\psi_{ce}$:$\psi_{cv}$ | 0.186737 | 0.48757 | 51096.1 |
| 614 | DisplMean~ $\psi_{ac}$+$\psi_{ce}$+$\psi_{cv}$+$\psi_{ce}$:$\psi_{ev}$ | 0.187917 | 0.48752 | 51098 |
| 615 | DisplMean~ $\psi_{ac}$:$\psi_{ev}$+$\psi_{ac}$:$\psi_{cv}$+$\psi_{ce}$:$\psi_{ev}$+$\psi_{ev}$:$\psi_{cv}$ | 0.156166 | 0.48711 | 51122.5 |
| 616 | DisplMean~ $\psi_{ev}$+$\psi_{ac}$:$\psi_{ev}$+$\psi_{ac}$:$\psi_{cv}$+$\psi_{ce}$:$\psi_{cv}$+$\psi_{ev}$:$\psi_{cv}$ | 0.193215 | 0.48699 | 51130.4 |
| 617 | DisplMean~ $\psi_{ce}$+$\psi_{ac}$:$\psi_{cv}$+$\psi_{ce}$:$\psi_{cv}$+$\psi_{ev}$:$\psi_{cv}$ | 0.123417 | 0.48639 | 51165.2 |

| | | | | |
|---|---|---|---|---|
| 618 | DisplMean~ $\psi_{ac}$+$\psi_{ce}$+$\psi_{ev}$:$\psi_{cv}$ | 0.198181 | 0.48633 | 51167.5 |
| 619 | DisplMean~ $\psi_{ac}$+$\psi_{ac}$:$\psi_{ce}$+$\psi_{ac}$:$\psi_{cv}$+$\psi_{ce}$:$\psi_{cv}$+$\psi_{ev}$:$\psi_{cv}$ | 0.210102 | 0.48581 | 51200.6 |
| 620 | DisplMean~ $\psi_{ac}$+$\psi_{ac}$:$\psi_{ev}$+$\psi_{ce}$:$\psi_{ev}$+$\psi_{ce}$:$\psi_{cv}$+$\psi_{ev}$:$\psi_{cv}$ | 0.205869 | 0.48578 | 51202 |
| 621 | DisplMean~ $\psi_{ac}$+$\psi_{ac}$:$\psi_{ce}$+$\psi_{ce}$:$\psi_{cv}$+$\psi_{ev}$:$\psi_{cv}$ | 0.197568 | 0.48531 | 51228.9 |
| 622 | DisplMean~ $\psi_{cv}$+$\psi_{ac}$:$\psi_{cv}$ | 0.208715 | 0.48495 | 51248.2 |
| 623 | DisplMean~ $\psi_{ac}$+$\psi_{ac}$:$\psi_{ev}$+$\psi_{ce}$:$\psi_{ev}$+$\psi_{ev}$:$\psi_{cv}$ | 0.200416 | 0.48371 | 51323.6 |
| 624 | DisplMean~ $\psi_{ac}$+$\psi_{ac}$:$\psi_{cv}$+$\psi_{ce}$:$\psi_{cv}$+$\psi_{ev}$:$\psi_{cv}$ | 0.231223 | 0.48135 | 51462.5 |
| 625 | DisplMean~ $\psi_{ac}$+$\psi_{ce}$+$\psi_{ce}$:$\psi_{ev}$+$\psi_{ce}$:$\psi_{cv}$ | 0.169874 | 0.47969 | 51559.9 |
| 626 | DisplMean~ $\psi_{ac}$+$\psi_{ce}$+$\psi_{ev}$+$\psi_{ac}$:$\psi_{ev}$+$\psi_{ac}$:$\psi_{cv}$+$\psi_{ce}$:$\psi_{ev}$ | 0.2168 | 0.47871 | 51619 |
| 627 | DisplMean~ $\psi_{ac}$+$\psi_{ce}$+$\psi_{ev}$+$\psi_{ac}$:$\psi_{ev}$+$\psi_{ac}$:$\psi_{cv}$ | 0.21677 | 0.47867 | 51620.5 |
| 628 | DisplMean~ $\psi_{ac}$+$\psi_{ce}$+$\psi_{ev}$+$\psi_{ac}$:$\psi_{cv}$+$\psi_{ce}$:$\psi_{ev}$ | 0.214885 | 0.4784 | 51636.3 |
| 629 | DisplMean~ $\psi_{ac}$+$\psi_{ce}$+$\psi_{ev}$+$\psi_{ac}$:$\psi_{cv}$ | 0.214624 | 0.4783 | 51641.4 |
| 630 | DisplMean~ $\psi_{ac}$:$\psi_{ce}$+$\psi_{ac}$:$\psi_{ev}$+$\psi_{ac}$:$\psi_{cv}$+$\psi_{ce}$:$\psi_{cv}$+$\psi_{ev}$:$\psi_{cv}$ | 0.162404 | 0.47656 | 51743.8 |
| 631 | DisplMean~ $\psi_{ac}$:$\psi_{ev}$+$\psi_{ac}$:$\psi_{cv}$+$\psi_{ce}$:$\psi_{cv}$+$\psi_{ev}$:$\psi_{cv}$ | 0.167176 | 0.47585 | 51783.7 |
| 632 | DisplMean~ $\psi_{ac}$+$\psi_{ce}$+$\psi_{ac}$:$\psi_{ev}$+$\psi_{ac}$:$\psi_{cv}$+$\psi_{ce}$:$\psi_{ev}$ | 0.215106 | 0.47574 | 51791.2 |
| 633 | DisplMean~ $\psi_{ac}$+$\psi_{ev}$+$\psi_{ac}$:$\psi_{ce}$+$\psi_{ac}$:$\psi_{ev}$+$\psi_{ac}$:$\psi_{cv}$+$\psi_{ev}$:$\psi_{cv}$ | 0.218883 | 0.47557 | 51802.1 |
| 634 | DisplMean~ $\psi_{ac}$+$\psi_{ce}$+$\psi_{ac}$:$\psi_{ev}$+$\psi_{ac}$:$\psi_{cv}$ | 0.215735 | 0.47533 | 51814 |
| 635 | DisplMean~ $\psi_{ce}$+$\psi_{ev}$+$\psi_{cv}$+$\psi_{ac}$:$\psi_{ev}$+$\psi_{ce}$:$\psi_{ev}$+$\psi_{ev}$:$\psi_{cv}$ | 0.173716 | 0.47519 | 51824.4 |
| 636 | DisplMean~ $\psi_{ce}$+$\psi_{ev}$+$\psi_{cv}$+$\psi_{ac}$:$\psi_{ev}$+$\psi_{ce}$:$\psi_{ev}$+$\psi_{ce}$:$\psi_{cv}$+$\psi_{ev}$:$\psi_{cv}$ | 0.174185 | 0.47518 | 51825.8 |
| 637 | DisplMean~ $\psi_{ac}$+$\psi_{cv}$+$\psi_{ce}$:$\psi_{cv}$+$\psi_{ev}$:$\psi_{cv}$ | 0.195279 | 0.47517 | 51823.5 |
| 638 | DisplMean~ $\psi_{ac}$+$\psi_{cv}$+$\psi_{ce}$:$\psi_{ev}$+$\psi_{ce}$:$\psi_{cv}$+$\psi_{ev}$:$\psi_{cv}$ | 0.195167 | 0.47515 | 51825.3 |
| 639 | DisplMean~ $\psi_{ac}$+$\psi_{ac}$:$\psi_{ce}$+$\psi_{ac}$:$\psi_{ev}$+$\psi_{ac}$:$\psi_{cv}$+$\psi_{ev}$:$\psi_{cv}$ | 0.217409 | 0.47511 | 51827.9 |
| 640 | DisplMean~ $\psi_{ce}$+$\psi_{ev}$+$\psi_{ac}$:$\psi_{ev}$+$\psi_{ce}$:$\psi_{ev}$+$\psi_{ev}$:$\psi_{cv}$ | 0.173448 | 0.47505 | 51831.6 |
| 641 | DisplMean~ $\psi_{ce}$+$\psi_{ev}$+$\psi_{ac}$:$\psi_{ev}$+$\psi_{ce}$:$\psi_{ev}$+$\psi_{ce}$:$\psi_{cv}$+$\psi_{ev}$:$\psi_{cv}$ | 0.172969 | 0.47505 | 51832.2 |
| 642 | DisplMean~ $\psi_{ac}$+$\psi_{ce}$:$\psi_{ev}$+$\psi_{ce}$:$\psi_{cv}$+$\psi_{ev}$:$\psi_{cv}$ | 0.191931 | 0.47351 | 51919.7 |
| 643 | DisplMean~ $\psi_{ce}$+$\psi_{ac}$:$\psi_{ce}$+$\psi_{ac}$:$\psi_{cv}$+$\psi_{ce}$:$\psi_{ev}$+$\psi_{ev}$:$\psi_{cv}$ | 0.103384 | 0.47346 | 51923.7 |
| 644 | DisplMean~ $\psi_{ce}$+$\psi_{ev}$+$\psi_{cv}$+$\psi_{ac}$:$\psi_{ev}$+$\psi_{ev}$:$\psi_{cv}$ | 0.172086 | 0.47324 | 51936.1 |

| | | | | |
|---|---|---|---|---|
| 645 | DisplMean~ $\psi_{ce}$+$\psi_{ev}$+$\psi_{cv}$+$\psi_{ac}$:$\psi_{ev}$+$\psi_{ce}$:$\psi_{cv}$+$\psi_{ev}$:$\psi_{cv}$ | 0.172603 | 0.47324 | 51937.3 |
| 646 | DisplMean~ $\psi_{ce}$+$\psi_{ev}$+$\psi_{ac}$:$\psi_{ev}$+$\psi_{ce}$:$\psi_{cv}$+$\psi_{ev}$:$\psi_{cv}$ | 0.171265 | 0.47307 | 51946 |
| 647 | DisplMean~ $\psi_{ce}$+$\psi_{ev}$+$\psi_{ac}$:$\psi_{ev}$+$\psi_{ev}$:$\psi_{cv}$ | 0.171804 | 0.47306 | 51945.8 |
| 648 | DisplMean~ $\psi_{ev}$+$\psi_{ac}$:$\psi_{ce}$+$\psi_{ac}$:$\psi_{ev}$+$\psi_{ac}$:$\psi_{cv}$+$\psi_{ev}$:$\psi_{cv}$ | 0.201139 | 0.47246 | 51981.2 |
| 649 | DisplMean~ $\psi_{ac}$+$\psi_{ce}$+$\psi_{ev}$+$\psi_{ac}$:$\psi_{ev}$+$\psi_{ce}$:$\psi_{ev}$ | 0.276567 | 0.47231 | 51990.1 |
| 650 | DisplMean~ $\psi_{ac}$+$\psi_{ce}$+$\psi_{ev}$+$\psi_{ac}$:$\psi_{ev}$ | 0.276539 | 0.47227 | 51991.6 |
| 651 | DisplMean~ $\psi_{ac}$+$\psi_{ce}$+$\psi_{ev}$+$\psi_{ce}$:$\psi_{ev}$ | 0.272755 | 0.47193 | 52010.8 |
| 652 | DisplMean~ $\psi_{ac}$+$\psi_{ce}$+$\psi_{ev}$ | 0.272304 | 0.47182 | 52016.4 |
| 653 | DisplMean~ $\psi_{ac}$+$\psi_{ev}$+$\psi_{ac}$:$\psi_{ce}$+$\psi_{ac}$:$\psi_{cv}$+$\psi_{ev}$:$\psi_{cv}$ | 0.220807 | 0.47156 | 52033.4 |
| 654 | DisplMean~ $\psi_{ac}$+$\psi_{ev}$+$\psi_{ac}$:$\psi_{ev}$+$\psi_{ce}$:$\psi_{cv}$+$\psi_{ev}$:$\psi_{cv}$ | 0.199187 | 0.4714 | 52042.3 |
| 655 | DisplMean~ $\psi_{ce}$+$\psi_{ev}$+$\psi_{cv}$+$\psi_{ac}$:$\psi_{ev}$+$\psi_{ce}$:$\psi_{ev}$+$\psi_{ce}$:$\psi_{cv}$ | 0.173643 | 0.4714 | 52043.6 |
| 656 | DisplMean~ $\psi_{ce}$+$\psi_{ev}$+$\psi_{cv}$+$\psi_{ac}$:$\psi_{ev}$+$\psi_{ce}$:$\psi_{ev}$ | 0.173017 | 0.47139 | 52042.9 |
| 657 | DisplMean~ $\psi_{ac}$+$\psi_{ev}$+$\psi_{ce}$:$\psi_{cv}$+$\psi_{ev}$:$\psi_{cv}$ | 0.200559 | 0.47052 | 52092.4 |
| 658 | DisplMean~ $\psi_{ac}$+$\psi_{ac}$:$\psi_{ev}$+$\psi_{ce}$:$\psi_{cv}$+$\psi_{ev}$:$\psi_{cv}$ | 0.198504 | 0.47016 | 52112.9 |
| 659 | DisplMean~ $\psi_{ac}$+$\psi_{ce}$:$\psi_{cv}$+$\psi_{ev}$:$\psi_{cv}$ | 0.196164 | 0.47003 | 52119.4 |
| 660 | DisplMean~ $\psi_{ce}$+$\psi_{ev}$+$\psi_{cv}$+$\psi_{ac}$:$\psi_{ev}$+$\psi_{ce}$:$\psi_{cv}$ | 0.172123 | 0.46971 | 52139.8 |
| 661 | DisplMean~ $\psi_{ce}$+$\psi_{ev}$+$\psi_{cv}$+$\psi_{ac}$:$\psi_{ev}$ | 0.171461 | 0.4697 | 52139.3 |
| 662 | DisplMean~ $\psi_{ac}$+$\psi_{ce}$+$\psi_{ac}$:$\psi_{ev}$+$\psi_{ce}$:$\psi_{ev}$ | 0.261641 | 0.46926 | 52164.5 |
| 663 | DisplMean~ $\psi_{ac}$+$\psi_{ev}$+$\psi_{ac}$:$\psi_{ev}$+$\psi_{ac}$:$\psi_{cv}$+$\psi_{ce}$:$\psi_{ev}$+$\psi_{ce}$:$\psi_{cv}$ | 0.180595 | 0.46924 | 52167.8 |
| 664 | DisplMean~ $\psi_{ac}$+$\psi_{ev}$+$\psi_{ac}$:$\psi_{ev}$+$\psi_{ce}$:$\psi_{ev}$+$\psi_{ce}$:$\psi_{cv}$ | 0.180863 | 0.46896 | 52182.9 |
| 665 | DisplMean~ $\psi_{ac}$+$\psi_{ce}$+$\psi_{ac}$:$\psi_{ev}$ | 0.262165 | 0.46886 | 52186.4 |
| 666 | DisplMean~ $\psi_{ev}$+$\psi_{ac}$:$\psi_{ce}$+$\psi_{ac}$:$\psi_{ev}$+$\psi_{ce}$:$\psi_{cv}$+$\psi_{ev}$:$\psi_{cv}$ | 0.154333 | 0.46721 | 52283 |
| 667 | DisplMean~ $\psi_{ac}$+$\psi_{ev}$+$\psi_{ac}$:$\psi_{cv}$+$\psi_{ce}$:$\psi_{ev}$+$\psi_{ce}$:$\psi_{cv}$ | 0.177364 | 0.46704 | 52292.5 |
| 668 | DisplMean~ $\psi_{ac}$+$\psi_{ev}$+$\psi_{ce}$:$\psi_{ev}$+$\psi_{ce}$:$\psi_{cv}$ | 0.177565 | 0.46686 | 52301.8 |
| 669 | DisplMean~ $\psi_{ac}$+$\psi_{ac}$:$\psi_{ce}$+$\psi_{ac}$:$\psi_{cv}$+$\psi_{ev}$:$\psi_{cv}$ | 0.238978 | 0.46587 | 52358.6 |
| 670 | DisplMean~ $\psi_{ac}$:$\psi_{ce}$+$\psi_{ac}$:$\psi_{ev}$+$\psi_{ac}$:$\psi_{cv}$+$\psi_{ev}$:$\psi_{cv}$ | 0.177052 | 0.4651 | 52402.1 |
| 671 | DisplMean~ $\psi_{ev}$+$\psi_{ac}$:$\psi_{ce}$+$\psi_{ac}$:$\psi_{ev}$+$\psi_{ce}$:$\psi_{ev}$+$\psi_{ev}$:$\psi_{cv}$ | 0.141225 | 0.46509 | 52404.1 |

| | | | | |
|---|---|---|---|---|
| 672 | DisplMean~ $\psi_{ev}$+$\psi_{cv}$+$\psi_{ac}$:$\psi_{ce}$+$\psi_{ac}$:$\psi_{ev}$+$\psi_{ce}$:$\psi_{ev}$+$\psi_{ev}$:$\psi_{cv}$ | 0.141324 | 0.46508 | 52405.4 |
| 673 | DisplMean~ $\psi_{ce}$+$\psi_{ev}$+$\psi_{ac}$:$\psi_{ev}$+$\psi_{ce}$:$\psi_{ev}$+$\psi_{ce}$:$\psi_{cv}$ | 0.16134 | 0.46375 | 52480.1 |
| 674 | DisplMean~ $\psi_{ce}$+$\psi_{ev}$+$\psi_{ac}$:$\psi_{ev}$+$\psi_{ce}$:$\psi_{cv}$ | 0.160083 | 0.46208 | 52574.2 |
| 675 | DisplMean~ $\psi_{ce}$+$\psi_{ac}$:$\psi_{ce}$+$\psi_{ac}$:$\psi_{cv}$+$\psi_{ev}$:$\psi_{cv}$ | 0.107253 | 0.46181 | 52589.2 |
| 676 | DisplMean~ $\psi_{ac}$+$\psi_{ev}$+$\psi_{ac}$:$\psi_{ev}$+$\psi_{ac}$:$\psi_{cv}$+$\psi_{ce}$:$\psi_{ev}$ | 0.189248 | 0.46118 | 52625.8 |
| 677 | DisplMean~ $\psi_{ev}$+$\psi_{cv}$+$\psi_{ac}$:$\psi_{ce}$+$\psi_{ac}$:$\psi_{ev}$+$\psi_{ce}$:$\psi_{ev}$ | 0.141844 | 0.46114 | 52628.3 |
| 678 | DisplMean~ $\psi_{ce}$+$\psi_{ev}$+$\psi_{ac}$:$\psi_{cv}$+$\psi_{ce}$:$\psi_{ev}$+$\psi_{ev}$:$\psi_{cv}$ | 0.135824 | 0.46065 | 52655.9 |
| 679 | DisplMean~ $\psi_{ev}$+$\psi_{cv}$+$\psi_{ac}$:$\psi_{ev}$+$\psi_{ce}$:$\psi_{ev}$+$\psi_{ce}$:$\psi_{cv}$+$\psi_{ev}$:$\psi_{cv}$ | 0.172495 | 0.46061 | 52658.8 |
| 680 | DisplMean~ $\psi_{ce}$+$\psi_{ev}$+$\psi_{ac}$:$\psi_{cv}$+$\psi_{ev}$:$\psi_{cv}$ | 0.13579 | 0.46017 | 52681.9 |
| 681 | DisplMean~ $\psi_{ac}$+$\psi_{ac}$:$\psi_{ev}$+$\psi_{ac}$:$\psi_{cv}$+$\psi_{ce}$:$\psi_{ev}$+$\psi_{ce}$:$\psi_{cv}$ | 0.187878 | 0.45984 | 52701.6 |
| 682 | DisplMean~ $\psi_{ac}$+$\psi_{ac}$:$\psi_{ev}$+$\psi_{ce}$:$\psi_{ev}$+$\psi_{ce}$:$\psi_{cv}$ | 0.187841 | 0.45982 | 52701.7 |
| 683 | DisplMean~ $\psi_{ac}$+$\psi_{ev}$+$\psi_{ac}$:$\psi_{cv}$+$\psi_{ce}$:$\psi_{ev}$ | 0.185459 | 0.4596 | 52714.2 |
| 684 | DisplMean~ $\psi_{ac}$+$\psi_{cv}$+$\psi_{ac}$:$\psi_{ce}$+$\psi_{ce}$:$\psi_{ev}$+$\psi_{ce}$:$\psi_{cv}$ | 0.145636 | 0.45785 | 52813.5 |
| 685 | DisplMean~ $\psi_{ev}$+$\psi_{cv}$+$\psi_{ac}$:$\psi_{ev}$+$\psi_{ce}$:$\psi_{cv}$+$\psi_{ev}$:$\psi_{cv}$ | 0.17472 | 0.45755 | 52830.6 |
| 686 | DisplMean~ $\psi_{ev}$+$\psi_{cv}$+$\psi_{ac}$:$\psi_{ev}$+$\psi_{ce}$:$\psi_{ev}$+$\psi_{ce}$:$\psi_{cv}$ | 0.171824 | 0.45743 | 52837 |
| 687 | DisplMean~ $\psi_{ev}$+$\psi_{ac}$:$\psi_{ev}$+$\psi_{ce}$:$\psi_{ev}$+$\psi_{ce}$:$\psi_{cv}$+$\psi_{ev}$:$\psi_{cv}$ | 0.166805 | 0.45573 | 52932.7 |
| 688 | DisplMean~ $\psi_{ac}$+$\psi_{cv}$+$\psi_{ac}$:$\psi_{ce}$+$\psi_{ce}$:$\psi_{ev}$+$\psi_{ev}$:$\psi_{cv}$ | 0.155556 | 0.45429 | 53013.1 |
| 689 | DisplMean~ $\psi_{ev}$+$\psi_{cv}$+$\psi_{ac}$:$\psi_{ev}$+$\psi_{ce}$:$\psi_{cv}$ | 0.17409 | 0.45419 | 53017.4 |
| 690 | DisplMean~ $\psi_{ac}$+$\psi_{ac}$:$\psi_{ev}$+$\psi_{ac}$:$\psi_{cv}$+$\psi_{ce}$:$\psi_{ev}$ | 0.193229 | 0.45406 | 53024.5 |
| 691 | DisplMean~ $\psi_{ac}$+$\psi_{ev}$+$\psi_{ac}$:$\psi_{ev}$+$\psi_{ce}$:$\psi_{ev}$ | 0.21362 | 0.4538 | 53039.4 |
| 692 | DisplMean~ $\psi_{ac}$+$\psi_{ce}$+$\psi_{ac}$:$\psi_{cv}$+$\psi_{ce}$:$\psi_{ev}$ | 0.174396 | 0.45363 | 53048.8 |
| 693 | DisplMean~ $\psi_{ac}$+$\psi_{ev}$+$\psi_{ce}$:$\psi_{ev}$ | 0.208459 | 0.45209 | 53133.5 |
| 694 | DisplMean~ $\psi_{ac}$+$\psi_{ev}$+$\psi_{ac}$:$\psi_{ev}$+$\psi_{ac}$:$\psi_{cv}$+$\psi_{ev}$:$\psi_{cv}$ | 0.225442 | 0.45141 | 53173.1 |
| 695 | DisplMean~ $\psi_{ac}$+$\psi_{ac}$:$\psi_{ev}$+$\psi_{ac}$:$\psi_{cv}$+$\psi_{ev}$:$\psi_{cv}$ | 0.222701 | 0.45096 | 53197.3 |
| 696 | DisplMean~ $\psi_{ac}$+$\psi_{cv}$+$\psi_{ce}$:$\psi_{ev}$+$\psi_{ev}$:$\psi_{cv}$ | 0.167974 | 0.44804 | 53358.7 |
| 697 | DisplMean~ $\psi_{ev}$+$\psi_{cv}$+$\psi_{ac}$:$\psi_{ev}$+$\psi_{ce}$:$\psi_{ev}$+$\psi_{ev}$:$\psi_{cv}$ | 0.156224 | 0.44794 | 53365.2 |
| 698 | DisplMean~ $\psi_{ev}$+$\psi_{ac}$:$\psi_{ev}$+$\psi_{ce}$:$\psi_{ev}$+$\psi_{ev}$:$\psi_{cv}$ | 0.156016 | 0.4476 | 53383.1 |

| | | | | |
|---|---|---|---|---|
| 699 | DisplMean~ $\psi_{ev}+\psi_{ac}:\psi_{ev}+\psi_{ce}:\psi_{cv}+\psi_{ev}:\psi_{cv}$ | 0.168984 | 0.44737 | 53395.8 |
| 700 | DisplMean~ $\psi_{ac}+\psi_{ev}+\psi_{ac}:\psi_{cv}+\psi_{ev}:\psi_{cv}$ | 0.228703 | 0.44683 | 53425.4 |
| 701 | DisplMean~ $\psi_{ac}+\psi_{ac}:\psi_{ev}+\psi_{ce}:\psi_{ev}$ | 0.215238 | 0.44678 | 53427.2 |
| 702 | DisplMean~ $\psi_{ac}+\psi_{ce}+\psi_{ce}:\psi_{ev}$ | 0.190662 | 0.44621 | 53458.8 |
| 703 | DisplMean~ $\psi_{ev}+\psi_{ac}:\psi_{ce}+\psi_{ac}:\psi_{cv}+\psi_{ce}:\psi_{ev}+\psi_{ce}:\psi_{cv}+\psi_{ev}:\psi_{cv}$ | 0.129364 | 0.44617 | 53463.7 |
| 704 | DisplMean~ $\psi_{ac}:\psi_{ce}+\psi_{ac}:\psi_{cv}+\psi_{ce}:\psi_{ev}+\psi_{ce}:\psi_{cv}+\psi_{ev}:\psi_{cv}$ | 0.128795 | 0.44608 | 53467.7 |
| 705 | DisplMean~ $\psi_{ev}+\psi_{ac}:\psi_{cv}+\psi_{ce}:\psi_{ev}+\psi_{ce}:\psi_{cv}+\psi_{ev}:\psi_{cv}$ | 0.127465 | 0.44601 | 53471.7 |
| 706 | DisplMean~ $\psi_{ac}:\psi_{cv}+\psi_{ce}:\psi_{ev}+\psi_{ce}:\psi_{cv}+\psi_{ev}:\psi_{cv}$ | 0.12713 | 0.44595 | 53474.1 |
| 707 | DisplMean~ $\psi_{ev}+\psi_{ac}:\psi_{ev}+\psi_{ac}:\psi_{cv}+\psi_{ev}:\psi_{cv}$ | 0.19289 | 0.44574 | 53485.4 |
| 708 | DisplMean~ $\psi_{ev}+\psi_{cv}+\psi_{ac}:\psi_{ev}+\psi_{ce}:\psi_{ev}$ | 0.155926 | 0.44487 | 53533.2 |
| 709 | DisplMean~ $\psi_{ce}+\psi_{ac}:\psi_{cv}+\psi_{ce}:\psi_{ev}+\psi_{ev}:\psi_{cv}$ | 0.136904 | 0.44441 | 53558.4 |
| 710 | DisplMean~ $\psi_{ev}+\psi_{ac}:\psi_{ce}+\psi_{ac}:\psi_{cv}+\psi_{ce}:\psi_{ev}+\psi_{ev}:\psi_{cv}$ | 0.127274 | 0.44225 | 53677.4 |
| 711 | DisplMean~ $\psi_{ev}+\psi_{ac}:\psi_{cv}+\psi_{ce}:\psi_{ev}+\psi_{ev}:\psi_{cv}$ | 0.131061 | 0.44165 | 53709.7 |
| 712 | DisplMean~ $\psi_{ce}+\psi_{ev}+\psi_{ac}:\psi_{ev}+\psi_{ac}:\psi_{cv}+\psi_{ce}:\psi_{ev}$ | 0.172905 | 0.44123 | 53733.6 |
| 713 | DisplMean~ $\psi_{ac}:\psi_{ce}+\psi_{ac}:\psi_{cv}+\psi_{ce}:\psi_{ev}+\psi_{ev}:\psi_{cv}$ | 0.124863 | 0.44074 | 53759.2 |
| 714 | DisplMean~ $\psi_{ac}+\psi_{ac}:\psi_{cv}+\psi_{ev}:\psi_{cv}$ | 0.285358 | 0.44008 | 53794.2 |
| 715 | DisplMean~ $\psi_{ac}+\psi_{ev}+\psi_{cv}+\psi_{ac}:\psi_{ce}+\psi_{ac}:\psi_{ev}+\psi_{ev}:\psi_{cv}$ | 0.206206 | 0.44003 | 53799.7 |
| 716 | DisplMean~ $\psi_{ac}+\psi_{ev}+\psi_{cv}+\psi_{ac}:\psi_{ce}+\psi_{ac}:\psi_{ev}$ | 0.206032 | 0.44001 | 53800 |
| 717 | DisplMean~ $\psi_{ce}+\psi_{ev}+\psi_{ac}:\psi_{ev}+\psi_{ac}:\psi_{cv}$ | 0.171572 | 0.43967 | 53817.3 |
| 718 | DisplMean~ $\psi_{ac}+\psi_{ev}+\psi_{cv}+\psi_{ac}:\psi_{ce}$ | 0.204524 | 0.43961 | 53820.3 |
| 719 | DisplMean~ $\psi_{ac}+\psi_{ev}+\psi_{cv}+\psi_{ac}:\psi_{ce}+\psi_{ev}:\psi_{cv}$ | 0.204521 | 0.4396 | 53822.3 |
| 720 | DisplMean~ $\psi_{ce}+\psi_{ev}+\psi_{ac}:\psi_{ev}+\psi_{ce}:\psi_{ev}$ | 0.18121 | 0.43954 | 53824.3 |
| 721 | DisplMean~ $\psi_{ac}:\psi_{cv}+\psi_{ce}:\psi_{ev}+\psi_{ev}:\psi_{cv}$ | 0.131762 | 0.43856 | 53876.4 |
| 722 | DisplMean~ $\psi_{ce}+\psi_{ev}+\psi_{ac}:\psi_{ev}$ | 0.179381 | 0.4381 | 53901.6 |
| 723 | DisplMean~ $\psi_{ac}+\psi_{cv}+\psi_{ac}:\psi_{ce}+\psi_{ac}:\psi_{ev}$ | 0.206246 | 0.43676 | 53975 |
| 724 | DisplMean~ $\psi_{ac}+\psi_{cv}+\psi_{ac}:\psi_{ce}+\psi_{ac}:\psi_{ev}+\psi_{ev}:\psi_{cv}$ | 0.206109 | 0.43675 | 53976.4 |
| 725 | DisplMean~ $\psi_{ev}+\psi_{ac}:\psi_{ce}+\psi_{ac}:\psi_{ev}+\psi_{ac}:\psi_{cv}+\psi_{ce}:\psi_{ev}+\psi_{ce}:\psi_{cv}$ | 0.136684 | 0.43655 | 53988.5 |

| | | | | |
|---|---|---|---|---|
| 726 | DisplMean~ $\psi_{ev}$+$\psi_{ac}$:$\psi_{ce}$+$\psi_{ac}$:$\psi_{ev}$+$\psi_{ac}$:$\psi_{cv}$+$\psi_{ce}$:$\psi_{ev}$ | 0.136968 | 0.43627 | 54002.5 |
| 727 | DisplMean~ $\psi_{ev}$+$\psi_{ac}$:$\psi_{ce}$+$\psi_{ac}$:$\psi_{ev}$+$\psi_{ce}$:$\psi_{ev}$ | 0.137158 | 0.43609 | 54011 |
| 728 | DisplMean~ $\psi_{ev}$+$\psi_{ac}$:$\psi_{ce}$+$\psi_{ac}$:$\psi_{ev}$+$\psi_{ce}$:$\psi_{ev}$+$\psi_{ce}$:$\psi_{cv}$ | 0.137145 | 0.43609 | 54012.5 |
| 729 | DisplMean~ $\psi_{ce}$+$\psi_{ac}$:$\psi_{cv}$+$\psi_{ev}$:$\psi_{cv}$ | 0.144381 | 0.43586 | 54022.7 |
| 730 | DisplMean~ $\psi_{ac}$:$\psi_{ev}$+$\psi_{ac}$:$\psi_{cv}$+$\psi_{ev}$:$\psi_{cv}$ | 0.160431 | 0.435 | 54069.2 |
| 731 | DisplMean~ $\psi_{ce}$+$\psi_{cv}$+$\psi_{ac}$:$\psi_{ce}$+$\psi_{ac}$:$\psi_{ev}$+$\psi_{ce}$:$\psi_{cv}$+$\psi_{ev}$:$\psi_{cv}$ | 0.0937592 | 0.43423 | 54113.4 |
| 732 | DisplMean~ $\psi_{ce}$+$\psi_{ac}$:$\psi_{ce}$+$\psi_{ac}$:$\psi_{ev}$+$\psi_{ac}$:$\psi_{cv}$+$\psi_{ce}$:$\psi_{ev}$+$\psi_{ce}$:$\psi_{cv}$ | 0.0965674 | 0.43237 | 54213.5 |
| 733 | DisplMean~ $\psi_{ac}$+$\psi_{ev}$+$\psi_{ac}$:$\psi_{ce}$+$\psi_{ac}$:$\psi_{ev}$+$\psi_{ev}$:$\psi_{cv}$ | 0.196179 | 0.43158 | 54255 |
| 734 | DisplMean~ $\psi_{ac}$+$\psi_{ev}$+$\psi_{ac}$:$\psi_{ce}$+$\psi_{ev}$:$\psi_{cv}$ | 0.196634 | 0.43154 | 54255.9 |
| 735 | DisplMean~ $\psi_{ac}$+$\psi_{ac}$:$\psi_{ce}$+$\psi_{ac}$:$\psi_{ev}$+$\psi_{ev}$:$\psi_{cv}$ | 0.196379 | 0.42976 | 54351.3 |
| 736 | DisplMean~ $\psi_{ac}$+$\psi_{cv}$+$\psi_{ac}$:$\psi_{ce}$+$\psi_{ce}$:$\psi_{cv}$ | 0.134228 | 0.42807 | 54441.6 |
| 737 | DisplMean~ $\psi_{ac}$+$\psi_{ac}$:$\psi_{ce}$+$\psi_{ce}$:$\psi_{ev}$+$\psi_{ev}$:$\psi_{cv}$ | 0.173196 | 0.42464 | 54623.8 |
| 738 | DisplMean~ $\psi_{ac}$+$\psi_{cv}$+$\psi_{ce}$:$\psi_{ev}$+$\psi_{ce}$:$\psi_{cv}$ | 0.174973 | 0.42401 | 54656.8 |
| 739 | DisplMean~ $\psi_{ac}$+$\psi_{ce}$:$\psi_{ev}$+$\psi_{ev}$:$\psi_{cv}$ | 0.170336 | 0.42374 | 54670.3 |
| 740 | DisplMean~ $\psi_{ce}$+$\psi_{cv}$+$\psi_{ac}$:$\psi_{ce}$+$\psi_{ac}$:$\psi_{ev}$+$\psi_{ev}$:$\psi_{cv}$ | 0.0982267 | 0.42194 | 54767.2 |
| 741 | DisplMean~ $\psi_{ev}$+$\psi_{ac}$:$\psi_{ev}$+$\psi_{ac}$:$\psi_{cv}$+$\psi_{ce}$:$\psi_{ev}$+$\psi_{ce}$:$\psi_{cv}$ | 0.148077 | 0.42116 | 54808.2 |
| 742 | DisplMean~ $\psi_{ac}$+$\psi_{cv}$+$\psi_{ac}$:$\psi_{ce}$+$\psi_{ev}$:$\psi_{cv}$ | 0.180815 | 0.41931 | 54904.3 |
| 743 | DisplMean~ $\psi_{ev}$+$\psi_{ac}$:$\psi_{ev}$+$\psi_{ce}$:$\psi_{ev}$+$\psi_{ce}$:$\psi_{cv}$ | 0.149228 | 0.4188 | 54931.1 |
| 744 | DisplMean~ $\psi_{ev}$+$\psi_{ac}$:$\psi_{ce}$+$\psi_{ac}$:$\psi_{cv}$+$\psi_{ce}$:$\psi_{cv}$+$\psi_{ev}$:$\psi_{cv}$ | 0.146269 | 0.41729 | 55011.2 |
| 745 | DisplMean~ $\psi_{ev}$+$\psi_{ac}$:$\psi_{ce}$+$\psi_{ac}$:$\psi_{cv}$+$\psi_{ev}$:$\psi_{cv}$ | 0.145281 | 0.41717 | 55016.7 |
| 746 | DisplMean~ $\psi_{ac}$+$\psi_{ac}$:$\psi_{ce}$+$\psi_{ev}$:$\psi_{cv}$ | 0.179332 | 0.41591 | 55081.3 |
| 747 | DisplMean~ $\psi_{ce}$+$\psi_{ev}$+$\psi_{ac}$:$\psi_{ce}$+$\psi_{ac}$:$\psi_{cv}$+$\psi_{ce}$:$\psi_{ev}$+$\psi_{ce}$:$\psi_{cv}$ | 0.101203 | 0.4147 | 55147.2 |
| 748 | DisplMean~ $\psi_{ce}$+$\psi_{ev}$+$\psi_{ac}$:$\psi_{ce}$+$\psi_{ac}$:$\psi_{cv}$+$\psi_{ce}$:$\psi_{cv}$ | 0.101054 | 0.41459 | 55151.9 |
| 749 | DisplMean~ $\psi_{ev}$+$\psi_{ac}$:$\psi_{ev}$+$\psi_{ac}$:$\psi_{cv}$+$\psi_{ce}$:$\psi_{ev}$ | 0.155522 | 0.41335 | 55215.8 |
| 750 | DisplMean~ $\psi_{ev}$+$\psi_{ac}$:$\psi_{ev}$+$\psi_{ce}$:$\psi_{ev}$ | 0.159813 | 0.41198 | 55285.7 |
| 751 | DisplMean~ $\psi_{ac}$+$\psi_{ev}$+$\psi_{cv}$+$\psi_{ac}$:$\psi_{ev}$+$\psi_{ev}$:$\psi_{cv}$ | 0.217193 | 0.41115 | 55330.5 |
| 752 | DisplMean~ $\psi_{ac}$+$\psi_{ev}$+$\psi_{cv}$+$\psi_{ac}$:$\psi_{ev}$ | 0.216921 | 0.41113 | 55330.4 |

| | | | | |
|---|---|---|---|---|
| 753 | DisplMean~ $\psi_{ac}$+$\psi_{ev}$+$\psi_{cv}$ | 0.214485 | 0.41077 | 55348.2 |
| 754 | DisplMean~ $\psi_{ac}$+$\psi_{ev}$+$\psi_{cv}$+$\psi_{ev}$:$\psi_{cv}$ | 0.214481 | 0.41075 | 55350.1 |
| 755 | DisplMean~ $\psi_{ac}$+$\psi_{ev}$+$\psi_{ac}$:$\psi_{ce}$+$\psi_{ac}$:$\psi_{ev}$+$\psi_{ac}$:$\psi_{cv}$+$\psi_{ce}$:$\psi_{cv}$ | 0.164605 | 0.41073 | 55353.3 |
| 756 | DisplMean~ $\psi_{ac}$+$\psi_{ev}$+$\psi_{ac}$:$\psi_{ce}$+$\psi_{ac}$:$\psi_{cv}$+$\psi_{ce}$:$\psi_{cv}$ | 0.16308 | 0.41029 | 55374.8 |
| 757 | DisplMean~ $\psi_{ac}$+$\psi_{ev}$+$\psi_{ac}$:$\psi_{ev}$+$\psi_{ac}$:$\psi_{cv}$+$\psi_{ce}$:$\psi_{cv}$ | 0.17673 | 0.40857 | 55463.9 |
| 758 | DisplMean~ $\psi_{ac}$+$\psi_{ev}$+$\psi_{ac}$:$\psi_{cv}$+$\psi_{ce}$:$\psi_{cv}$ | 0.175139 | 0.40809 | 55487.6 |
| 759 | DisplMean~ $\psi_{ac}$+$\psi_{cv}$+$\psi_{ac}$:$\psi_{ev}$ | 0.214984 | 0.40777 | 55503.1 |
| 760 | DisplMean~ $\psi_{ce}$+$\psi_{cv}$+$\psi_{ac}$:$\psi_{ev}$+$\psi_{ce}$:$\psi_{ev}$+$\psi_{ce}$:$\psi_{cv}$+$\psi_{ev}$:$\psi_{cv}$ | 0.126004 | 0.40777 | 55505.9 |
| 761 | DisplMean~ $\psi_{ac}$+$\psi_{cv}$+$\psi_{ac}$:$\psi_{ev}$+$\psi_{ev}$:$\psi_{cv}$ | 0.214751 | 0.40776 | 55504.4 |
| 762 | DisplMean~ $\psi_{ac}$+$\psi_{ac}$:$\psi_{ce}$+$\psi_{ac}$:$\psi_{ev}$+$\psi_{ac}$:$\psi_{cv}$+$\psi_{ce}$:$\psi_{cv}$ | 0.167482 | 0.4077 | 55508.3 |
| 763 | DisplMean~ $\psi_{ce}$+$\psi_{cv}$+$\psi_{ac}$:$\psi_{ev}$+$\psi_{ce}$:$\psi_{ev}$+$\psi_{ev}$:$\psi_{cv}$ | 0.12468 | 0.40752 | 55518 |
| 764 | DisplMean~ $\psi_{ce}$+$\psi_{ev}$+$\psi_{ac}$:$\psi_{cv}$+$\psi_{ce}$:$\psi_{ev}$+$\psi_{ce}$:$\psi_{cv}$ | 0.109043 | 0.40695 | 55547 |
| 765 | DisplMean~ $\psi_{ce}$+$\psi_{ev}$+$\psi_{ac}$:$\psi_{cv}$+$\psi_{ce}$:$\psi_{cv}$ | 0.108891 | 0.40683 | 55552.2 |
| 766 | DisplMean~ $\psi_{ac}$+$\psi_{ac}$:$\psi_{ev}$+$\psi_{ac}$:$\psi_{cv}$+$\psi_{ce}$:$\psi_{cv}$ | 0.178966 | 0.40563 | 55613.6 |
| 767 | DisplMean~ $\psi_{ev}$+$\psi_{cv}$+$\psi_{ac}$:$\psi_{ce}$+$\psi_{ac}$:$\psi_{ev}$+$\psi_{ev}$:$\psi_{cv}$ | 0.162174 | 0.40352 | 55722.6 |
| 768 | DisplMean~ $\psi_{ev}$+$\psi_{ac}$:$\psi_{ce}$+$\psi_{ac}$:$\psi_{ev}$+$\psi_{ev}$:$\psi_{cv}$ | 0.161907 | 0.40267 | 55765.2 |
| 769 | DisplMean~ $\psi_{ac}$+$\psi_{ev}$+$\psi_{ac}$:$\psi_{ev}$+$\psi_{ev}$:$\psi_{cv}$ | 0.19891 | 0.40236 | 55780.9 |
| 770 | DisplMean~ $\psi_{ac}$+$\psi_{ev}$+$\psi_{ev}$:$\psi_{cv}$ | 0.199689 | 0.4023 | 55783 |
| 771 | DisplMean~ $\psi_{ev}$+$\psi_{ac}$:$\psi_{cv}$+$\psi_{ce}$:$\psi_{cv}$+$\psi_{ev}$:$\psi_{cv}$ | 0.130588 | 0.40224 | 55786.8 |
| 772 | DisplMean~ $\psi_{ev}$+$\psi_{cv}$+$\psi_{ac}$:$\psi_{ce}$+$\psi_{ac}$:$\psi_{ev}$ | 0.161842 | 0.40057 | 55872 |
| 773 | DisplMean~ $\psi_{ac}$+$\psi_{ac}$:$\psi_{ev}$+$\psi_{ev}$:$\psi_{cv}$ | 0.198486 | 0.40048 | 55875.4 |
| 774 | DisplMean~ $\psi_{ce}$+$\psi_{ac}$:$\psi_{ce}$+$\psi_{ac}$:$\psi_{ev}$+$\psi_{ce}$:$\psi_{ev}$+$\psi_{ev}$:$\psi_{cv}$ | 0.103414 | 0.40021 | 55891.1 |
| 775 | DisplMean~ $\psi_{ce}$+$\psi_{ac}$:$\psi_{ev}$+$\psi_{ac}$:$\psi_{cv}$+$\psi_{ce}$:$\psi_{ev}$+$\psi_{ce}$:$\psi_{cv}$ | 0.110712 | 0.39859 | 55973.2 |
| 776 | DisplMean~ $\psi_{ac}$+$\psi_{ev}$+$\psi_{ac}$:$\psi_{ce}$+$\psi_{ac}$:$\psi_{ev}$+$\psi_{ac}$:$\psi_{cv}$ | 0.18912 | 0.39559 | 56124.9 |
| 777 | DisplMean~ $\psi_{ac}$+$\psi_{ev}$+$\psi_{ac}$:$\psi_{ce}$+$\psi_{ac}$:$\psi_{cv}$ | 0.18734 | 0.39506 | 56151 |
| 778 | DisplMean~ $\psi_{ac}$:$\psi_{ce}$+$\psi_{ac}$:$\psi_{cv}$+$\psi_{ce}$:$\psi_{cv}$+$\psi_{ev}$:$\psi_{cv}$ | 0.154374 | 0.39423 | 56192.7 |
| 779 | DisplMean~ $\psi_{ac}$+$\psi_{ac}$:$\psi_{ce}$+$\psi_{ac}$:$\psi_{ev}$+$\psi_{ac}$:$\psi_{cv}$ | 0.190915 | 0.39277 | 56266 |

| | | | | |
|---|---|---|---|---|
| 780 | DisplMean~ $\psi_{ce}$+$\psi_{ac}$:$\psi_{ce}$+$\psi_{ac}$:$\psi_{cv}$+$\psi_{ce}$:$\psi_{ev}$+$\psi_{ce}$:$\psi_{cv}$ | 0.0991456 | 0.39224 | 56293.3 |
| 781 | DisplMean~ $\psi_{ce}$+$\psi_{ac}$:$\psi_{ev}$+$\psi_{ce}$:$\psi_{ev}$+$\psi_{ce}$:$\psi_{cv}$+$\psi_{ev}$:$\psi_{cv}$ | 0.127116 | 0.39224 | 56293.6 |
| 782 | DisplMean~ $\psi_{ac}$:$\psi_{ce}$+$\psi_{ac}$:$\psi_{cv}$+$\psi_{ev}$:$\psi_{cv}$ | 0.153863 | 0.39105 | 56351.1 |
| 783 | DisplMean~ $\psi_{ac}$+$\psi_{cv}$+$\psi_{ev}$:$\psi_{cv}$ | 0.176358 | 0.39045 | 56381.2 |
| 784 | DisplMean~ $\psi_{ac}$+$\psi_{ce}$+$\psi_{ac}$:$\psi_{cv}$+$\psi_{ce}$:$\psi_{cv}$ | 0.155805 | 0.38937 | 56436.1 |
| 785 | DisplMean~ $\psi_{ce}$+$\psi_{ac}$:$\psi_{ce}$+$\psi_{ac}$:$\psi_{ev}$+$\psi_{ac}$:$\psi_{cv}$+$\psi_{ce}$:$\psi_{ev}$ | 0.0869779 | 0.38803 | 56503.5 |
| 786 | DisplMean~ $\psi_{ac}$+$\psi_{ev}$:$\psi_{cv}$ | 0.173721 | 0.38748 | 56528.3 |
| 787 | DisplMean~ $\psi_{ac}$+$\psi_{ev}$+$\psi_{ac}$:$\psi_{ce}$+$\psi_{ac}$:$\psi_{ev}$+$\psi_{ce}$:$\psi_{cv}$ | 0.208312 | 0.38726 | 56542 |
| 788 | DisplMean~ $\psi_{ac}$+$\psi_{ev}$+$\psi_{ac}$:$\psi_{ce}$+$\psi_{ac}$:$\psi_{ev}$ | 0.214381 | 0.38685 | 56561.4 |
| 789 | DisplMean~ $\psi_{ac}$+$\psi_{ev}$+$\psi_{ac}$:$\psi_{ce}$+$\psi_{ce}$:$\psi_{cv}$ | 0.206137 | 0.38667 | 56570.5 |
| 790 | DisplMean~ $\psi_{ac}$+$\psi_{ev}$+$\psi_{ac}$:$\psi_{ce}$ | 0.212081 | 0.38624 | 56590.5 |
| 791 | DisplMean~ $\psi_{ce}$+$\psi_{ac}$:$\psi_{cv}$+$\psi_{ce}$:$\psi_{ev}$+$\psi_{ce}$:$\psi_{cv}$ | 0.106498 | 0.38455 | 56675.4 |
| 792 | DisplMean~ $\psi_{ac}$+$\psi_{ac}$:$\psi_{ce}$+$\psi_{ac}$:$\psi_{ev}$+$\psi_{ce}$:$\psi_{cv}$ | 0.209152 | 0.38452 | 56677.1 |
| 793 | DisplMean~ $\psi_{ac}$+$\psi_{ac}$:$\psi_{ce}$+$\psi_{ac}$:$\psi_{ev}$ | 0.214826 | 0.3841 | 56696.5 |
| 794 | DisplMean~ $\psi_{ac}$+$\psi_{ce}$+$\psi_{cv}$+$\psi_{ce}$:$\psi_{cv}$ | 0.182783 | 0.3798 | 56909.8 |
| 795 | DisplMean~ $\psi_{ac}$+$\psi_{ce}$+$\psi_{cv}$ | 0.185301 | 0.37975 | 56911.3 |
| 796 | DisplMean~ $\psi_{ac}$+$\psi_{ce}$+$\psi_{ce}$:$\psi_{cv}$ | 0.154328 | 0.37211 | 57284.1 |
| 797 | DisplMean~ $\psi_{ac}$+$\psi_{ev}$+$\psi_{ac}$:$\psi_{ev}$+$\psi_{ce}$:$\psi_{cv}$ | 0.301747 | 0.37119 | 57329.8 |
| 798 | DisplMean~ $\psi_{ac}$+$\psi_{ev}$+$\psi_{ce}$:$\psi_{cv}$ | 0.293422 | 0.37049 | 57362.3 |
| 799 | DisplMean~ $\psi_{ce}$+$\psi_{cv}$+$\psi_{ac}$:$\psi_{ce}$+$\psi_{ac}$:$\psi_{ev}$+$\psi_{ce}$:$\psi_{ev}$+$\psi_{ce}$:$\psi_{cv}$ | 0.103336 | 0.36977 | 57400.1 |
| 800 | DisplMean~ $\psi_{ce}$+$\psi_{ac}$:$\psi_{ev}$+$\psi_{ce}$:$\psi_{ev}$+$\psi_{ev}$:$\psi_{cv}$ | 0.114689 | 0.36906 | 57432.6 |
| 801 | DisplMean~ $\psi_{ac}$+$\psi_{ac}$:$\psi_{ev}$+$\psi_{ce}$:$\psi_{cv}$ | 0.280159 | 0.36824 | 57471.3 |
| 802 | DisplMean~ $\psi_{ac}$+$\psi_{ev}$+$\psi_{ac}$:$\psi_{ev}$+$\psi_{ac}$:$\psi_{cv}$ | 0.191419 | 0.36398 | 57677 |
| 803 | DisplMean~ $\psi_{ac}$+$\psi_{ev}$+$\psi_{ac}$:$\psi_{cv}$ | 0.188908 | 0.36348 | 57699.8 |
| 804 | DisplMean~ $\psi_{ev}$+$\psi_{ac}$:$\psi_{ce}$+$\psi_{ac}$:$\psi_{ev}$+$\psi_{ac}$:$\psi_{cv}$+$\psi_{ce}$:$\psi_{cv}$ | 0.160443 | 0.36307 | 57721.3 |
| 805 | DisplMean~ $\psi_{ac}$+$\psi_{cv}$+$\psi_{ce}$:$\psi_{cv}$ | 0.185431 | 0.36262 | 57741 |
| 806 | DisplMean~ $\psi_{cv}$+$\psi_{ac}$:$\psi_{ce}$+$\psi_{ac}$:$\psi_{ev}$+$\psi_{ce}$:$\psi_{ev}$+$\psi_{ce}$:$\psi_{cv}$+$\psi_{ev}$:$\psi_{cv}$ | 0.11892 | 0.36253 | 57748.3 |

| | | | | |
|---|---|---|---|---|
| 807 | DisplMean~ $\psi_{ev}+\psi_{cv}+\psi_{ac}:\psi_{ev}+\psi_{ev}:\psi_{cv}$ | 0.148653 | 0.3615 | 57795.3 |
| 808 | DisplMean~ $\psi_{ev}+\psi_{ac}:\psi_{ev}+\psi_{ac}:\psi_{cv}+\psi_{ce}:\psi_{cv}$ | 0.156473 | 0.36136 | 57802.3 |
| 809 | DisplMean~ $\psi_{ev}+\psi_{ac}:\psi_{ev}+\psi_{ev}:\psi_{cv}$ | 0.148446 | 0.36117 | 57810.3 |
| 810 | DisplMean~ $\psi_{ac}+\psi_{ac}:\psi_{ev}+\psi_{ac}:\psi_{cv}$ | 0.193517 | 0.36106 | 57815.3 |
| 811 | DisplMean~ $\psi_{ev}+\psi_{ac}:\psi_{cv}+\psi_{ev}:\psi_{cv}$ | 0.116048 | 0.36072 | 57831.7 |
| 812 | DisplMean~ $\psi_{ev}+\psi_{ac}:\psi_{ce}+\psi_{ac}:\psi_{ev}+\psi_{ac}:\psi_{cv}$ | 0.163162 | 0.36069 | 57833.9 |
| 813 | DisplMean~ $\psi_{ce}+\psi_{cv}+\psi_{ac}:\psi_{ce}+\psi_{ac}:\psi_{ev}+\psi_{ce}:\psi_{ev}$ | 0.106209 | 0.36021 | 57858.1 |
| 814 | DisplMean~ $\psi_{ce}+\psi_{ac}:\psi_{ce}+\psi_{ac}:\psi_{ev}+\psi_{ce}:\psi_{ev}+\psi_{ce}:\psi_{cv}$ | 0.104487 | 0.36008 | 57864.2 |
| 815 | DisplMean~ $\psi_{ac}:\psi_{ce}+\psi_{ac}:\psi_{ev}+\psi_{ce}:\psi_{ev}+\psi_{ce}:\psi_{cv}+\psi_{ev}:\psi_{cv}$ | 0.118604 | 0.36003 | 57866.5 |
| 816 | DisplMean~ $\psi_{ac}:\psi_{cv}+\psi_{ce}:\psi_{cv}+\psi_{ev}:\psi_{cv}$ | 0.145639 | 0.35995 | 57868.4 |
| 817 | DisplMean~ $\psi_{ce}+\psi_{ac}:\psi_{ce}+\psi_{ac}:\psi_{ev}+\psi_{ce}:\psi_{ev}$ | 0.105563 | 0.35951 | 57890.3 |
| 818 | DisplMean~ $\psi_{ce}+\psi_{cv}+\psi_{ac}:\psi_{ev}+\psi_{ce}:\psi_{cv}+\psi_{ev}:\psi_{cv}$ | 0.11779 | 0.35936 | 57898.3 |
| 819 | DisplMean~ $\psi_{ce}+\psi_{cv}+\psi_{ac}:\psi_{ev}+\psi_{ev}:\psi_{cv}$ | 0.115807 | 0.35855 | 57936 |
| 820 | DisplMean~ $\psi_{ev}+\psi_{ac}:\psi_{ce}+\psi_{ac}:\psi_{ev}+\psi_{ce}:\psi_{cv}$ | 0.168771 | 0.35821 | 57952.2 |
| 821 | DisplMean~ $\psi_{cv}+\psi_{ac}:\psi_{ce}+\psi_{ac}:\psi_{ev}+\psi_{ce}:\psi_{cv}+\psi_{ev}:\psi_{cv}$ | 0.114376 | 0.35787 | 57969 |
| 822 | DisplMean~ $\psi_{ev}+\psi_{ac}:\psi_{ce}+\psi_{ac}:\psi_{ev}$ | 0.171255 | 0.35781 | 57969.8 |
| 823 | DisplMean~ $\psi_{ev}+\psi_{cv}+\psi_{ac}:\psi_{ev}$ | 0.148779 | 0.35709 | 58004.3 |
| 824 | DisplMean~ $\psi_{cv}+\psi_{ac}:\psi_{ev}+\psi_{ce}:\psi_{ev}+\psi_{ce}:\psi_{cv}+\psi_{ev}:\psi_{cv}$ | 0.127376 | 0.35666 | 58026.5 |
| 825 | DisplMean~ $\psi_{ce}+\psi_{ac}:\psi_{ce}+\psi_{ac}:\psi_{ev}+\psi_{ce}:\psi_{cv}+\psi_{ev}:\psi_{cv}$ | 0.0942962 | 0.35641 | 58038.5 |
| 826 | DisplMean~ $\psi_{ac}+\psi_{ev}+\psi_{ac}:\psi_{ev}$ | 0.248788 | 0.35578 | 58065.9 |
| 827 | DisplMean~ $\psi_{ac}+\psi_{ev}$ | 0.243464 | 0.35518 | 58093.3 |
| 828 | DisplMean~ $\psi_{ac}:\psi_{ev}+\psi_{ce}:\psi_{ev}+\psi_{ce}:\psi_{cv}+\psi_{ev}:\psi_{cv}$ | 0.12765 | 0.35362 | 58168.9 |
| 829 | DisplMean~ $\psi_{ac}+\psi_{ac}:\psi_{ev}$ | 0.242663 | 0.35291 | 58200.5 |
| 830 | DisplMean~ $\psi_{cv}+\psi_{ac}:\psi_{ev}+\psi_{ce}:\psi_{cv}+\psi_{ev}:\psi_{cv}$ | 0.122576 | 0.34721 | 58469.9 |
| 831 | DisplMean~ $\psi_{ac}+\psi_{ce}+\psi_{ac}:\psi_{cv}$ | 0.160793 | 0.34591 | 58529.2 |
| 832 | DisplMean~ $\psi_{ac}:\psi_{ce}+\psi_{ac}:\psi_{ev}+\psi_{ce}:\psi_{cv}+\psi_{ev}:\psi_{cv}$ | 0.108308 | 0.34313 | 58659.4 |
| 833 | DisplMean~ $\psi_{ac}+\psi_{ce}$ | 0.189764 | 0.33833 | 58879.1 |

| | | | | |
|---|---|---|---|---|
| 834 | DisplMean~ $\psi_{ev}+\psi_{ac}:\psi_{ev}+\psi_{ce}:\psi_{cv}$ | 0.181624 | 0.33582 | 58995.8 |
| 835 | DisplMean~ $\psi_{ce}+\psi_{cv}+\psi_{ac}:\psi_{ev}+\psi_{ce}:\psi_{ev}+\psi_{ce}:\psi_{cv}$ | 0.120443 | 0.33037 | 59246.5 |
| 836 | DisplMean~ $\psi_{ac}+\psi_{cv}+\psi_{ac}:\psi_{ce}+\psi_{ce}:\psi_{ev}$ | 0.168743 | 0.32994 | 59265 |
| 837 | DisplMean~ $\psi_{ev}+\psi_{ac}:\psi_{ce}+\psi_{ac}:\psi_{cv}+\psi_{ce}:\psi_{ev}+\psi_{ce}:\psi_{cv}$ | 0.122086 | 0.3291 | 59304.3 |
| 838 | DisplMean~ $\psi_{ce}+\psi_{cv}+\psi_{ac}:\psi_{ev}+\psi_{ce}:\psi_{ev}$ | 0.118124 | 0.32885 | 59314.8 |
| 839 | DisplMean~ $\psi_{ce}+\psi_{ac}:\psi_{ev}+\psi_{ac}:\psi_{cv}+\psi_{ce}:\psi_{ev}$ | 0.126021 | 0.32479 | 59498.5 |
| 840 | DisplMean~ $\psi_{ce}+\psi_{ac}:\psi_{ev}+\psi_{ce}:\psi_{ev}+\psi_{ce}:\psi_{cv}$ | 0.117647 | 0.32394 | 59536.8 |
| 841 | DisplMean~ $\psi_{ce}+\psi_{ev}+\psi_{ac}:\psi_{cv}+\psi_{ce}:\psi_{ev}$ | 0.219392 | 0.32061 | 59686.5 |
| 842 | DisplMean~ $\psi_{ce}+\psi_{ev}+\psi_{ac}:\psi_{cv}$ | 0.216764 | 0.32033 | 59698 |
| 843 | DisplMean~ $\psi_{ce}+\psi_{ac}:\psi_{ev}+\psi_{ce}:\psi_{cv}+\psi_{ev}:\psi_{cv}$ | 0.122212 | 0.32001 | 59713.2 |
| 844 | DisplMean~ $\psi_{ce}+\psi_{ac}:\psi_{ce}+\psi_{ac}:\psi_{ev}+\psi_{ac}:\psi_{cv}+\psi_{ce}:\psi_{cv}$ | 0.0924368 | 0.31966 | 59729.7 |
| 845 | DisplMean~ $\psi_{ce}+\psi_{ac}:\psi_{ev}+\psi_{ac}:\psi_{cv}+\psi_{ce}:\psi_{cv}$ | 0.0953899 | 0.31891 | 59762.6 |
| 846 | DisplMean~ $\psi_{ce}+\psi_{ac}:\psi_{ev}+\psi_{ce}:\psi_{ev}$ | 0.122395 | 0.31889 | 59762.4 |
| 847 | DisplMean~ $\psi_{ac}:\psi_{ev}+\psi_{ce}:\psi_{cv}+\psi_{ev}:\psi_{cv}$ | 0.121918 | 0.31849 | 59780.4 |
| 848 | DisplMean~ $\psi_{ev}+\psi_{ac}:\psi_{cv}+\psi_{ce}:\psi_{ev}+\psi_{ce}:\psi_{cv}$ | 0.113372 | 0.31621 | 59883.2 |
| 849 | DisplMean~ $\psi_{ev}+\psi_{ac}:\psi_{ev}+\psi_{ac}:\psi_{cv}$ | 0.152351 | 0.31548 | 59914.3 |
| 850 | DisplMean~ $\psi_{ac}:\psi_{cv}+\psi_{ev}:\psi_{cv}$ | 0.112957 | 0.31532 | 59920.4 |
| 851 | DisplMean~ $\psi_{ev}+\psi_{ac}:\psi_{ev}$ | 0.159152 | 0.31355 | 59999.4 |
| 852 | DisplMean~ $\psi_{ac}+\psi_{cv}+\psi_{ac}:\psi_{ce}$ | 0.166433 | 0.30744 | 60270.1 |
| 853 | DisplMean~ $\psi_{cv}+\psi_{ac}:\psi_{ce}+\psi_{ac}:\psi_{ev}+\psi_{ev}:\psi_{cv}$ | 0.122618 | 0.30495 | 60380.4 |
| 854 | DisplMean~ $\psi_{cv}+\psi_{ac}:\psi_{ce}+\psi_{ac}:\psi_{ev}+\psi_{ce}:\psi_{ev}+\psi_{ev}:\psi_{cv}$ | 0.122633 | 0.30493 | 60382.4 |
| 855 | DisplMean~ $\psi_{ce}+\psi_{ev}+\psi_{cv}+\psi_{ce}:\psi_{ev}+\psi_{ce}:\psi_{cv}$ | 0.387123 | 0.30288 | 60472.2 |
| 856 | DisplMean~ $\psi_{ce}+\psi_{ev}+\psi_{cv}+\psi_{ce}:\psi_{ev}+\psi_{ce}:\psi_{cv}+\psi_{ev}:\psi_{cv}$ | 0.387116 | 0.30286 | 60474.2 |
| 857 | DisplMean~ $\psi_{ce}+\psi_{ev}+\psi_{cv}+\psi_{ce}:\psi_{cv}$ | 0.380075 | 0.3025 | 60487.7 |
| 858 | DisplMean~ $\psi_{ce}+\psi_{ev}+\psi_{cv}+\psi_{ce}:\psi_{cv}+\psi_{ev}:\psi_{cv}$ | 0.380114 | 0.30248 | 60489.7 |
| 859 | DisplMean~ $\psi_{ce}+\psi_{ev}+\psi_{ce}:\psi_{ev}+\psi_{ce}:\psi_{cv}+\psi_{ev}:\psi_{cv}$ | 0.374733 | 0.30001 | 60597.2 |
| 860 | DisplMean~ $\psi_{ce}+\psi_{ev}+\psi_{ce}:\psi_{cv}+\psi_{ev}:\psi_{cv}$ | 0.368209 | 0.29957 | 60615.2 |

| | | | | |
|---|---|---|---|---|
| 861 | DisplMean~ $\psi_{ce}+\psi_{ac}:\psi_{cv}+\psi_{ce}:\psi_{ev}$ | 0.156514 | 0.29819 | 60674.2 |
| 862 | DisplMean~ $\psi_{ac}+\psi_{ac}:\psi_{cv}+\psi_{ce}:\psi_{ev}+\psi_{ce}:\psi_{cv}$ | 0.141406 | 0.29799 | 60684 |
| 863 | DisplMean~ $\psi_{ac}+\psi_{ac}:\psi_{ce}+\psi_{ac}:\psi_{cv}+\psi_{ce}:\psi_{ev}+\psi_{ce}:\psi_{cv}$ | 0.142205 | 0.29798 | 60685.5 |
| 864 | DisplMean~ $\psi_{ce}+\psi_{ev}+\psi_{cv}+\psi_{ce}:\psi_{ev}$ | 0.395268 | 0.29785 | 60690.1 |
| 865 | DisplMean~ $\psi_{ce}+\psi_{ev}+\psi_{cv}+\psi_{ce}:\psi_{ev}+\psi_{ev}:\psi_{cv}$ | 0.395265 | 0.29783 | 60692.1 |
| 866 | DisplMean~ $\psi_{ce}+\psi_{ev}+\psi_{ce}:\psi_{ev}+\psi_{ev}:\psi_{cv}$ | 0.380049 | 0.29761 | 60700.6 |
| 867 | DisplMean~ $\psi_{ce}+\psi_{ev}+\psi_{cv}$ | 0.387655 | 0.29746 | 60705.9 |
| 868 | DisplMean~ $\psi_{ce}+\psi_{ev}+\psi_{cv}+\psi_{ev}:\psi_{cv}$ | 0.387694 | 0.29744 | 60707.9 |
| 869 | DisplMean~ $\psi_{ce}+\psi_{ev}+\psi_{ev}:\psi_{cv}$ | 0.373102 | 0.2972 | 60717.1 |
| 870 | DisplMean~ $\psi_{ce}+\psi_{ev}+\psi_{ce}:\psi_{ev}+\psi_{ce}:\psi_{cv}$ | 0.415364 | 0.29657 | 60745.4 |
| 871 | DisplMean~ $\psi_{ce}+\psi_{ev}+\psi_{ce}:\psi_{ev}$ | 0.388743 | 0.29655 | 60745.4 |
| 872 | DisplMean~ $\psi_{ce}+\psi_{ev}+\psi_{ce}:\psi_{cv}$ | 0.402219 | 0.29618 | 60761.5 |
| 873 | DisplMean~ $\psi_{ce}+\psi_{ev}$ | 0.381258 | 0.29616 | 60761.2 |
| 874 | DisplMean~ $\psi_{ev}+\psi_{cv}+\psi_{ce}:\psi_{ev}+\psi_{ce}:\psi_{cv}$ | 0.23836 | 0.29559 | 60788 |
| 875 | DisplMean~ $\psi_{ev}+\psi_{cv}+\psi_{ce}:\psi_{ev}+\psi_{ce}:\psi_{cv}+\psi_{ev}:\psi_{cv}$ | 0.2384 | 0.29557 | 60789.9 |
| 876 | DisplMean~ $\psi_{ev}+\psi_{ac}:\psi_{ce}+\psi_{ac}:\psi_{cv}+\psi_{ce}:\psi_{ev}$ | 0.156903 | 0.2951 | 60809.1 |
| 877 | DisplMean~ $\psi_{ev}+\psi_{ac}:\psi_{cv}+\psi_{ce}:\psi_{ev}$ | 0.154716 | 0.29447 | 60835.3 |
| 878 | DisplMean~ $\psi_{ev}+\psi_{cv}+\psi_{ac}:\psi_{ce}+\psi_{ce}:\psi_{cv}$ | 0.204627 | 0.29336 | 60884.3 |
| 879 | DisplMean~ $\psi_{ev}+\psi_{cv}+\psi_{ac}:\psi_{ce}+\psi_{ce}:\psi_{cv}+\psi_{ev}:\psi_{cv}$ | 0.20463 | 0.29334 | 60886.3 |
| 880 | DisplMean~ $\psi_{ev}+\psi_{cv}+\psi_{ce}:\psi_{cv}$ | 0.221971 | 0.293 | 60899 |
| 881 | DisplMean~ $\psi_{ev}+\psi_{cv}+\psi_{ce}:\psi_{cv}+\psi_{ev}:\psi_{cv}$ | 0.221975 | 0.29297 | 60900.9 |
| 882 | DisplMean~ $\psi_{ce}+\psi_{ce}:\psi_{ev}+\psi_{ce}:\psi_{cv}+\psi_{ev}:\psi_{cv}$ | 0.22442 | 0.29139 | 60969.3 |
| 883 | DisplMean~ $\psi_{ce}+\psi_{cv}+\psi_{ce}:\psi_{ev}+\psi_{ce}:\psi_{cv}+\psi_{ev}:\psi_{cv}$ | 0.22563 | 0.29139 | 60970.3 |
| 884 | DisplMean~ $\psi_{ev}+\psi_{ac}:\psi_{ce}+\psi_{ac}:\psi_{cv}+\psi_{ce}:\psi_{cv}$ | 0.14552 | 0.28944 | 61052.7 |
| 885 | DisplMean~ $\psi_{ce}+\psi_{ac}:\psi_{ce}+\psi_{ac}:\psi_{cv}+\psi_{ce}:\psi_{cv}$ | 0.0733916 | 0.28823 | 61104.5 |
| 886 | DisplMean~ $\psi_{ev}+\psi_{ac}:\psi_{ce}+\psi_{ce}:\psi_{ev}+\psi_{ce}:\psi_{cv}+\psi_{ev}:\psi_{cv}$ | 0.195016 | 0.28711 | 61153.6 |
| 887 | DisplMean~ $\psi_{ev}+\psi_{ce}:\psi_{ev}+\psi_{ce}:\psi_{cv}+\psi_{ev}:\psi_{cv}$ | 0.219195 | 0.28662 | 61173.6 |

| | | | | |
|---|---|---|---|---|
| 888 | DisplMean~ $\psi_{ce}$+$\psi_{cv}$+$\psi_{ce}$:$\psi_{ev}$+$\psi_{ev}$:$\psi_{cv}$ | 0.226355 | 0.28632 | 61186.1 |
| 889 | DisplMean~ $\psi_{ev}$+$\psi_{ac}$:$\psi_{ce}$+$\psi_{ce}$:$\psi_{cv}$+$\psi_{ev}$:$\psi_{cv}$ | 0.174193 | 0.28297 | 61329.1 |
| 890 | DisplMean~ $\psi_{ac}$+$\psi_{ac}$:$\psi_{ce}$+$\psi_{ac}$:$\psi_{cv}$+$\psi_{ce}$:$\psi_{ev}$ | 0.160812 | 0.2823 | 61357.4 |
| 891 | DisplMean~ $\psi_{cv}$+$\psi_{ac}$:$\psi_{ev}$+$\psi_{ce}$:$\psi_{ev}$+$\psi_{ev}$:$\psi_{cv}$ | 0.108442 | 0.28069 | 61425.4 |
| 892 | DisplMean~ $\psi_{ce}$+$\psi_{cv}$+$\psi_{ce}$:$\psi_{cv}$+$\psi_{ev}$:$\psi_{cv}$ | 0.197187 | 0.28064 | 61427.5 |
| 893 | DisplMean~ $\psi_{ce}$+$\psi_{ce}$:$\psi_{ev}$+$\psi_{ev}$:$\psi_{cv}$ | 0.211199 | 0.28011 | 61449 |
| 894 | DisplMean~ $\psi_{ac}$+$\psi_{ac}$:$\psi_{ce}$+$\psi_{ac}$:$\psi_{cv}$+$\psi_{ce}$:$\psi_{cv}$ | 0.127752 | 0.27999 | 61455.1 |
| 895 | DisplMean~ $\psi_{ev}$+$\psi_{cv}$+$\psi_{ac}$:$\psi_{ce}$+$\psi_{ce}$:$\psi_{ev}$ | 0.160948 | 0.2798 | 61463.1 |
| 896 | DisplMean~ $\psi_{ev}$+$\psi_{cv}$+$\psi_{ac}$:$\psi_{ce}$+$\psi_{ce}$:$\psi_{ev}$+$\psi_{ev}$:$\psi_{cv}$ | 0.160975 | 0.27979 | 61464.8 |
| 897 | DisplMean~ $\psi_{ce}$+$\psi_{ac}$:$\psi_{cv}$+$\psi_{ce}$:$\psi_{cv}$ | 0.0846793 | 0.27973 | 61465.2 |
| 898 | DisplMean~ $\psi_{ac}$+$\psi_{cv}$+$\psi_{ce}$:$\psi_{ev}$ | 0.151216 | 0.27913 | 61490.8 |
| 899 | DisplMean~ $\psi_{ev}$+$\psi_{ac}$:$\psi_{ce}$+$\psi_{ce}$:$\psi_{ev}$+$\psi_{ev}$:$\psi_{cv}$ | 0.161194 | 0.27894 | 61499.5 |
| 900 | DisplMean~ $\psi_{ce}$+$\psi_{cv}$+$\psi_{ce}$:$\psi_{ev}$+$\psi_{ce}$:$\psi_{cv}$ | 0.191643 | 0.27876 | 61507.4 |
| 901 | DisplMean~ $\psi_{ac}$+$\psi_{cv}$ | 0.15047 | 0.27868 | 61508.6 |
| 902 | DisplMean~ $\psi_{ev}$+$\psi_{ce}$:$\psi_{cv}$+$\psi_{ev}$:$\psi_{cv}$ | 0.200071 | 0.27753 | 61558.3 |
| 903 | DisplMean~ $\psi_{ac}$+$\psi_{ac}$:$\psi_{cv}$+$\psi_{ce}$:$\psi_{cv}$ | 0.141371 | 0.2773 | 61567.9 |
| 904 | DisplMean~ $\psi_{ce}$+$\psi_{ce}$:$\psi_{cv}$+$\psi_{ev}$:$\psi_{cv}$ | 0.185676 | 0.27693 | 61583.4 |
| 905 | DisplMean~ $\psi_{ce}$+$\psi_{cv}$+$\psi_{ev}$:$\psi_{cv}$ | 0.197426 | 0.27555 | 61641.6 |
| 906 | DisplMean~ $\psi_{cv}$+$\psi_{ac}$:$\psi_{ce}$+$\psi_{ce}$:$\psi_{ev}$+$\psi_{ce}$:$\psi_{cv}$+$\psi_{ev}$:$\psi_{cv}$ | 0.166586 | 0.27499 | 61666.9 |
| 907 | DisplMean~ $\psi_{ev}$+$\psi_{ac}$:$\psi_{ce}$+$\psi_{ce}$:$\psi_{ev}$ | 0.166564 | 0.27487 | 61670.2 |
| 908 | DisplMean~ $\psi_{ev}$+$\psi_{ac}$:$\psi_{ce}$+$\psi_{ce}$:$\psi_{ev}$+$\psi_{ce}$:$\psi_{cv}$ | 0.168073 | 0.27487 | 61671.1 |
| 909 | DisplMean~ $\psi_{ac}$:$\psi_{ce}$+$\psi_{ce}$:$\psi_{ev}$+$\psi_{ce}$:$\psi_{cv}$+$\psi_{ev}$:$\psi_{cv}$ | 0.166021 | 0.27453 | 61685.4 |
| 910 | DisplMean~ $\psi_{ce}$+$\psi_{cv}$+$\psi_{ce}$:$\psi_{ev}$ | 0.191988 | 0.27373 | 61718.1 |
| 911 | DisplMean~ $\psi_{cv}$+$\psi_{ce}$:$\psi_{ev}$+$\psi_{ce}$:$\psi_{cv}$+$\psi_{ev}$:$\psi_{cv}$ | 0.178081 | 0.27342 | 61731.9 |
| 912 | DisplMean~ $\psi_{cv}$+$\psi_{ac}$:$\psi_{ce}$+$\psi_{ce}$:$\psi_{cv}$+$\psi_{ev}$:$\psi_{cv}$ | 0.169298 | 0.27332 | 61736.2 |
| 913 | DisplMean~ $\psi_{ac}$:$\psi_{ce}$+$\psi_{ce}$:$\psi_{cv}$+$\psi_{ev}$:$\psi_{cv}$ | 0.170371 | 0.27328 | 61736.6 |
| 914 | DisplMean~ $\psi_{ev}$+$\psi_{cv}$+$\psi_{ac}$:$\psi_{ce}$ | 0.152739 | 0.27309 | 61744.6 |

| | | | | |
|---|---|---|---|---|
| 915 | DisplMean~ $\psi_{ev}$+$\psi_{cv}$+$\psi_{ac}$:$\psi_{ce}$+$\psi_{ev}$:$\psi_{cv}$ | 0.15274 | 0.27307 | 61746.6 |
| 916 | DisplMean~ $\psi_{ev}$+$\psi_{ac}$:$\psi_{ce}$+$\psi_{ac}$:$\psi_{cv}$ | 0.161183 | 0.27278 | 61757.7 |
| 917 | DisplMean~ $\psi_{ac}$+$\psi_{ac}$:$\psi_{ce}$+$\psi_{ce}$:$\psi_{ev}$+$\psi_{ce}$:$\psi_{cv}$ | 0.172542 | 0.27278 | 61758.9 |
| 918 | DisplMean~ $\psi_{cv}$+$\psi_{ce}$:$\psi_{cv}$+$\psi_{ev}$:$\psi_{cv}$ | 0.177716 | 0.27267 | 61762.3 |
| 919 | DisplMean~ $\psi_{ce}$:$\psi_{ev}$+$\psi_{ce}$:$\psi_{cv}$+$\psi_{ev}$:$\psi_{cv}$ | 0.179185 | 0.27258 | 61766.1 |
| 920 | DisplMean~ $\psi_{ce}$:$\psi_{cv}$+$\psi_{ev}$:$\psi_{cv}$ | 0.178597 | 0.27256 | 61766 |
| 921 | DisplMean~ $\psi_{ce}$+$\psi_{ce}$:$\psi_{ev}$ | 0.19098 | 0.27251 | 61767.9 |
| 922 | DisplMean~ $\psi_{ce}$+$\psi_{ce}$:$\psi_{ev}$+$\psi_{ce}$:$\psi_{cv}$ | 0.190982 | 0.27249 | 61769.9 |
| 923 | DisplMean~ $\psi_{ac}$+$\psi_{ac}$:$\psi_{ce}$+$\psi_{ce}$:$\psi_{ev}$ | 0.175945 | 0.27199 | 61790.6 |
| 924 | DisplMean~ $\psi_{ev}$+$\psi_{ac}$:$\psi_{ce}$+$\psi_{ev}$:$\psi_{cv}$ | 0.15284 | 0.27175 | 61801 |
| 925 | DisplMean~ $\psi_{ev}$+$\psi_{ce}$:$\psi_{ev}$+$\psi_{ce}$:$\psi_{cv}$ | 0.206439 | 0.27041 | 61857 |
| 926 | DisplMean~ $\psi_{ev}$+$\psi_{cv}$+$\psi_{ce}$:$\psi_{ev}$+$\psi_{ev}$:$\psi_{cv}$ | 0.190993 | 0.26818 | 61950.8 |
| 927 | DisplMean~ $\psi_{ev}$+$\psi_{cv}$+$\psi_{ce}$:$\psi_{ev}$ | 0.190959 | 0.26817 | 61950.2 |
| 928 | DisplMean~ $\psi_{ev}$+$\psi_{ce}$:$\psi_{ev}$+$\psi_{ev}$:$\psi_{cv}$ | 0.190271 | 0.26784 | 61964.1 |
| 929 | DisplMean~ $\psi_{ev}$+$\psi_{ce}$:$\psi_{ev}$ | 0.189712 | 0.26678 | 62007.2 |
| 930 | DisplMean~ $\psi_{ac}$+$\psi_{ac}$:$\psi_{ce}$+$\psi_{ac}$:$\psi_{cv}$ | 0.154816 | 0.26388 | 62128.1 |
| 931 | DisplMean~ $\psi_{ev}$+$\psi_{ac}$:$\psi_{ce}$ | 0.158424 | 0.26317 | 62156.8 |
| 932 | DisplMean~ $\psi_{ev}$+$\psi_{ac}$:$\psi_{ce}$+$\psi_{ce}$:$\psi_{cv}$ | 0.157574 | 0.26317 | 62157.8 |
| 933 | DisplMean~ $\psi_{ac}$:$\psi_{ce}$+$\psi_{ac}$:$\psi_{ev}$+$\psi_{ce}$:$\psi_{ev}$+$\psi_{ev}$:$\psi_{cv}$ | 0.125706 | 0.25754 | 62390.5 |
| 934 | DisplMean~ $\psi_{ac}$+$\psi_{ac}$:$\psi_{ce}$+$\psi_{ce}$:$\psi_{cv}$ | 0.170463 | 0.25498 | 62494.5 |
| 935 | DisplMean~ $\psi_{ac}$+$\psi_{ac}$:$\psi_{ce}$ | 0.174469 | 0.2544 | 62517.1 |
| 936 | DisplMean~ $\psi_{cv}$+$\psi_{ac}$:$\psi_{ce}$+$\psi_{ev}$:$\psi_{cv}$ | 0.142719 | 0.25375 | 62544.5 |
| 937 | DisplMean~ $\psi_{cv}$+$\psi_{ac}$:$\psi_{ce}$+$\psi_{ce}$:$\psi_{ev}$+$\psi_{ev}$:$\psi_{cv}$ | 0.142737 | 0.25374 | 62546.1 |
| 938 | DisplMean~ $\psi_{cv}$+$\psi_{ac}$:$\psi_{ce}$+$\psi_{ac}$:$\psi_{ev}$+$\psi_{ce}$:$\psi_{ev}$+$\psi_{ce}$:$\psi_{cv}$ | 0.136687 | 0.24922 | 62730.8 |
| 939 | DisplMean~ $\psi_{cv}$+$\psi_{ac}$:$\psi_{ce}$+$\psi_{ce}$:$\psi_{ev}$+$\psi_{ce}$:$\psi_{cv}$ | 0.147234 | 0.24324 | 62971.5 |
| 940 | DisplMean~ $\psi_{ac}$:$\psi_{ce}$+$\psi_{ce}$:$\psi_{ev}$+$\psi_{ev}$:$\psi_{cv}$ | 0.137195 | 0.24153 | 63039.3 |
| 941 | DisplMean~ $\psi_{cv}$+$\psi_{ac}$:$\psi_{ev}$+$\psi_{ce}$:$\psi_{ev}$+$\psi_{ce}$:$\psi_{cv}$ | 0.13134 | 0.24072 | 63073.2 |

| | | | | |
|---|---|---|---|---|
| 942 | DisplMean~ $\psi_{ce}$+$\psi_{ac}$:$\psi_{ce}$+$\psi_{ac}$:$\psi_{ev}$+$\psi_{ev}$:$\psi_{cv}$ | 0.121611 | 0.2397 | 63113.8 |
| 943 | DisplMean~ $\psi_{ac}$+$\psi_{ce}$:$\psi_{ev}$+$\psi_{ce}$:$\psi_{cv}$ | 0.198872 | 0.23839 | 63165.5 |
| 944 | DisplMean~ $\psi_{ce}$+$\psi_{ac}$:$\psi_{ev}$+$\psi_{ev}$:$\psi_{cv}$ | 0.13158 | 0.23753 | 63199.8 |
| 945 | DisplMean~ $\psi_{ac}$+$\psi_{ce}$:$\psi_{cv}$ | 0.194949 | 0.23739 | 63204.1 |
| 946 | DisplMean~ $\psi_{ce}$+$\psi_{ac}$:$\psi_{ce}$+$\psi_{ev}$:$\psi_{cv}$ | 0.145708 | 0.23498 | 63301.2 |
| 947 | DisplMean~ $\psi_{ac}$+$\psi_{ac}$:$\psi_{cv}$+$\psi_{ce}$:$\psi_{ev}$ | 0.138804 | 0.23391 | 63344.1 |
| 948 | DisplMean~ $\psi_{cv}$+$\psi_{ce}$:$\psi_{ev}$+$\psi_{ce}$:$\psi_{cv}$ | 0.150736 | 0.23342 | 63363.6 |
| 949 | DisplMean~ $\psi_{ac}$+$\psi_{ac}$:$\psi_{cv}$ | 0.138761 | 0.23255 | 63397 |
| 950 | DisplMean~ $\psi_{ce}$+$\psi_{ev}$:$\psi_{cv}$ | 0.21471 | 0.23199 | 63419.3 |
| 951 | DisplMean~ $\psi_{ac}$:$\psi_{ce}$+$\psi_{ac}$:$\psi_{ev}$+$\psi_{ev}$:$\psi_{cv}$ | 0.12038 | 0.23097 | 63460.5 |
| 952 | DisplMean~ $\psi_{cv}$+$\psi_{ac}$:$\psi_{ev}$+$\psi_{ev}$:$\psi_{cv}$ | 0.0981562 | 0.22897 | 63539.7 |
| 953 | DisplMean~ $\psi_{ac}$:$\psi_{ce}$+$\psi_{ev}$:$\psi_{cv}$ | 0.13082 | 0.22801 | 63576.5 |
| 954 | DisplMean~ $\psi_{ac}$+$\psi_{ce}$:$\psi_{ev}$ | 0.164251 | 0.22453 | 63713.5 |
| 955 | DisplMean~ $\psi_{ac}$ | 0.16379 | 0.22304 | 63771.3 |
| 956 | DisplMean~ $\psi_{ce}$+$\psi_{ac}$:$\psi_{ev}$+$\psi_{ac}$:$\psi_{cv}$ | 0.232356 | 0.22185 | 63819.6 |
| 957 | DisplMean~ $\psi_{ac}$:$\psi_{ce}$+$\psi_{ac}$:$\psi_{ev}$+$\psi_{ac}$:$\psi_{cv}$+$\psi_{ce}$:$\psi_{ev}$+$\psi_{ce}$:$\psi_{cv}$ | 0.136189 | 0.22018 | 63887.1 |
| 958 | DisplMean~ $\psi_{ac}$:$\psi_{ce}$+$\psi_{ac}$:$\psi_{ev}$+$\psi_{ac}$:$\psi_{cv}$+$\psi_{ce}$:$\psi_{cv}$ | 0.133807 | 0.21956 | 63910.4 |
| 959 | DisplMean~ $\psi_{ev}$+$\psi_{ce}$:$\psi_{cv}$ | 0.431753 | 0.21755 | 63986.7 |
| 960 | DisplMean~ $\psi_{ev}$+$\psi_{ac}$:$\psi_{cv}$+$\psi_{ce}$:$\psi_{cv}$ | 0.424265 | 0.21755 | 63987.5 |
| 961 | DisplMean~ $\psi_{ce}$+$\psi_{cv}$+$\psi_{ac}$:$\psi_{ev}$+$\psi_{ce}$:$\psi_{cv}$ | 0.865017 | 0.21567 | 64061.7 |
| 962 | DisplMean~ $\psi_{ce}$+$\psi_{ac}$:$\psi_{ev}$+$\psi_{ce}$:$\psi_{cv}$ | 0.865954 | 0.21517 | 64080.2 |
| 963 | DisplMean~ $\psi_{ce}$+$\psi_{ac}$:$\psi_{ce}$+$\psi_{ac}$:$\psi_{cv}$ | 0.0152733 | 0.21514 | 64081.2 |
| 964 | DisplMean~ $\psi_{cv}$+$\psi_{ce}$:$\psi_{ev}$+$\psi_{ev}$:$\psi_{cv}$ | 0.150745 | 0.21098 | 64242.2 |
| 965 | DisplMean~ $\psi_{ce}$+$\psi_{cv}$+$\psi_{ac}$:$\psi_{ev}$ | 0.868753 | 0.21052 | 64260.2 |
| 966 | DisplMean~ $\psi_{ce}$+$\psi_{ac}$:$\psi_{ev}$ | 0.865209 | 0.20979 | 64287.2 |
| 967 | DisplMean~ $\psi_{cv}$+$\psi_{ac}$:$\psi_{ce}$+$\psi_{ce}$:$\psi_{ev}$ | 0.136206 | 0.20894 | 64320.8 |
| 968 | DisplMean~ $\psi_{cv}$+$\psi_{ac}$:$\psi_{ce}$+$\psi_{ac}$:$\psi_{ev}$+$\psi_{ce}$:$\psi_{ev}$ | 0.136187 | 0.20892 | 64322.8 |

| | | | | |
|---|---|---|---|---|
| 969 | DisplMean~ $\psi_{ac}{:}\psi_{ce}+\psi_{ac}{:}\psi_{cv}+\psi_{ce}{:}\psi_{ev}+\psi_{ce}{:}\psi_{cv}$ | 0.140964 | 0.20052 | 64644.4 |
| 970 | DisplMean~ $\psi_{ac}{:}\psi_{ce}+\psi_{ac}{:}\psi_{ev}+\psi_{ac}{:}\psi_{cv}+\psi_{ce}{:}\psi_{ev}$ | 0.151146 | 0.19739 | 64763.6 |
| 971 | DisplMean~ $\psi_{ac}{:}\psi_{ce}+\psi_{ac}{:}\psi_{cv}+\psi_{ce}{:}\psi_{ev}$ | 0.145707 | 0.19151 | 64984.8 |
| 972 | DisplMean~ $\psi_{ce}+\psi_{ac}{:}\psi_{cv}$ | 0.0922687 | 0.19139 | 64988.5 |
| 973 | DisplMean~ $\psi_{ac}{:}\psi_{ce}+\psi_{ac}{:}\psi_{ev}+\psi_{ce}{:}\psi_{ev}+\psi_{ce}{:}\psi_{cv}$ | 0.144511 | 0.19094 | 65007.5 |
| 974 | DisplMean~ $\psi_{ac}{:}\psi_{ce}+\psi_{ac}{:}\psi_{ev}+\psi_{ce}{:}\psi_{ev}$ | 0.14597 | 0.19075 | 65013.6 |
| 975 | DisplMean~ $\psi_{ac}{:}\psi_{ce}+\psi_{ce}{:}\psi_{ev}+\psi_{ce}{:}\psi_{cv}$ | 0.141867 | 0.18898 | 65080 |
| 976 | DisplMean~ $\psi_{ac}{:}\psi_{ce}+\psi_{ce}{:}\psi_{ev}$ | 0.143334 | 0.18869 | 65090.1 |
| 977 | DisplMean~ $\psi_{ac}{:}\psi_{ce}+\psi_{ac}{:}\psi_{ev}+\psi_{ac}{:}\psi_{cv}$ | 0.156314 | 0.18592 | 65194.8 |
| 978 | DisplMean~ $\psi_{cv}+\psi_{ac}{:}\psi_{ce}+\psi_{ac}{:}\psi_{ev}+\psi_{ce}{:}\psi_{cv}$ | 0.195721 | 0.18497 | 65231.4 |
| 979 | DisplMean~ $\psi_{cv}+\psi_{ac}{:}\psi_{ev}+\psi_{ce}{:}\psi_{cv}$ | 0.263365 | 0.18255 | 65320.8 |
| 980 | DisplMean~ $\psi_{cv}+\psi_{ac}{:}\psi_{ce}+\psi_{ac}{:}\psi_{ev}$ | 0.151198 | 0.17195 | 65713 |
| 981 | DisplMean~ $\psi_{ce}+\psi_{cv}+\psi_{ce}{:}\psi_{cv}$ | 0.174951 | 0.17068 | 65759.7 |
| 982 | DisplMean~ $\psi_{ac}{:}\psi_{ce}+\psi_{ac}{:}\psi_{ev}$ | 0.156604 | 0.16639 | 65916 |
| 983 | DisplMean~ $\psi_{ac}{:}\psi_{ce}+\psi_{ac}{:}\psi_{ev}+\psi_{ce}{:}\psi_{cv}$ | 0.156399 | 0.16636 | 65917.9 |
| 984 | DisplMean~ $\psi_{ce}+\psi_{cv}$ | 0.175345 | 0.16566 | 65942.7 |
| 985 | DisplMean~ $\psi_{ce}$ | 0.170984 | 0.16445 | 65985.6 |
| 986 | DisplMean~ $\psi_{ce}+\psi_{ce}{:}\psi_{cv}$ | 0.170968 | 0.16443 | 65987.6 |
| 987 | DisplMean~ $\psi_{cv}+\psi_{ac}{:}\psi_{ce}+\psi_{ce}{:}\psi_{cv}$ | 0.138559 | 0.16384 | 66010.1 |
| 988 | DisplMean~ $\psi_{ev}+\psi_{ac}{:}\psi_{cv}$ | 0.193801 | 0.16343 | 66023.8 |
| 989 | DisplMean~ $\psi_{cv}+\psi_{ce}{:}\psi_{cv}$ | 0.14749 | 0.163 | 66039.7 |
| 990 | DisplMean~ $\psi_{ac}{:}\psi_{ce}+\psi_{ac}{:}\psi_{cv}+\psi_{ce}{:}\psi_{cv}$ | 0.122739 | 0.16122 | 66105.3 |
| 991 | DisplMean~ $\psi_{cv}+\psi_{ac}{:}\psi_{ce}$ | 0.120653 | 0.14516 | 66681.9 |
| 992 | DisplMean~ $\psi_{ac}{:}\psi_{ce}+\psi_{ac}{:}\psi_{cv}$ | 0.126821 | 0.14435 | 66710.9 |
| 993 | DisplMean~ $\psi_{ev}+\psi_{cv}$ | 1.78855 | 0.13607 | 67004.3 |
| 994 | DisplMean~ $\psi_{ev}+\psi_{cv}+\psi_{ev}{:}\psi_{cv}$ | 1.79015 | 0.13604 | 67006.2 |
| 995 | DisplMean~ $\psi_{ac}{:}\psi_{ce}+\psi_{ce}{:}\psi_{cv}$ | 0.125866 | 0.13506 | 67040 |

| | | | | |
|---|---|---|---|---|
| 996 | DisplMean~ $\psi_{ac}$:$\psi_{ce}$ | 0.126605 | 0.13502 | 67040.3 |
| 997 | DisplMean~ $\psi_{ev}$+$\psi_{ev}$:$\psi_{cv}$ | 0.58104 | 0.13444 | 67061.7 |
| 998 | DisplMean~ $\psi_{ev}$ | 1.9017 | 0.13372 | 67085.8 |
| 999 | DisplMean~ $\psi_{ac}$:$\psi_{ev}$+$\psi_{ac}$:$\psi_{cv}$+$\psi_{ce}$:$\psi_{ev}$+$\psi_{ce}$:$\psi_{cv}$ | 0.650448 | 0.12703 | 67323.3 |
| 1000 | DisplMean~ $\psi_{ac}$:$\psi_{ev}$+$\psi_{ce}$:$\psi_{ev}$+$\psi_{ce}$:$\psi_{cv}$ | 0.624814 | 0.12353 | 67444.2 |
| 1001 | DisplMean~ $\psi_{ac}$:$\psi_{ev}$+$\psi_{ac}$:$\psi_{cv}$+$\psi_{ce}$:$\psi_{cv}$ | 0.593327 | 0.12092 | 67534.9 |
| 1002 | DisplMean~ $\psi_{ac}$:$\psi_{ev}$+$\psi_{ce}$:$\psi_{cv}$ | 0.569204 | 0.12011 | 67562 |
| 1003 | DisplMean~ $\psi_{ac}$:$\psi_{ev}$+$\psi_{ce}$:$\psi_{ev}$+$\psi_{ev}$:$\psi_{cv}$ | 0.11298 | 0.11402 | 67772.9 |
| 1004 | DisplMean~ $\psi_{ce}$:$\psi_{ev}$+$\psi_{ev}$:$\psi_{cv}$ | 0.170477 | 0.11276 | 67815.3 |
| 1005 | DisplMean~ $\psi_{cv}$+$\psi_{ev}$:$\psi_{cv}$ | 0.202254 | 0.11134 | 67864 |
| 1006 | DisplMean~ $\psi_{ac}$:$\psi_{ev}$+$\psi_{ac}$:$\psi_{cv}$+$\psi_{ce}$:$\psi_{ev}$ | 0.338201 | 0.10459 | 68095.4 |
| 1007 | DisplMean~ $\psi_{ac}$:$\psi_{cv}$+$\psi_{ce}$:$\psi_{ev}$+$\psi_{ce}$:$\psi_{cv}$ | 0.249439 | 0.09423 | 68445.9 |
| 1008 | DisplMean~ $\psi_{ac}$:$\psi_{ev}$+$\psi_{ev}$:$\psi_{cv}$ | 0.0456605 | 0.09166 | 68531.1 |
| 1009 | DisplMean~ $\psi_{ce}$:$\psi_{ev}$+$\psi_{ce}$:$\psi_{cv}$ | 0.232927 | 0.09144 | 68538.6 |
| 1010 | DisplMean~ $\psi_{ac}$:$\psi_{cv}$+$\psi_{ce}$:$\psi_{cv}$ | 0.276107 | 0.08509 | 68750.8 |
| 1011 | DisplMean~ $\psi_{ce}$:$\psi_{cv}$ | 0.25772 | 0.08502 | 68752 |
| 1012 | DisplMean~ $\psi_{ev}$:$\psi_{cv}$ | 0.218108 | 0.07237 | 69170.3 |
| 1013 | DisplMean~ $\psi_{cv}$+$\psi_{ac}$:$\psi_{ev}$+$\psi_{ce}$:$\psi_{ev}$ | 0.690469 | 0.06431 | 69436 |
| 1014 | DisplMean~ $\psi_{ac}$:$\psi_{ev}$+$\psi_{ce}$:$\psi_{ev}$ | 0.685719 | 0.06416 | 69439.6 |
| 1015 | DisplMean~ $\psi_{ac}$:$\psi_{ev}$+$\psi_{ac}$:$\psi_{cv}$ | 0.0773651 | 0.05895 | 69608.8 |
| 1016 | DisplMean~ $\psi_{cv}$+$\psi_{ac}$:$\psi_{ev}$ | 0.892158 | 0.05276 | 69808.6 |
| 1017 | DisplMean~ $\psi_{ac}$:$\psi_{ev}$ | 0.887498 | 0.05076 | 69871.9 |
| 1018 | DisplMean~ $\psi_{ac}$:$\psi_{cv}$+$\psi_{ce}$:$\psi_{ev}$ | 0.0192884 | 0.048 | 69961.3 |
| 1019 | DisplMean~ $\psi_{ac}$:$\psi_{cv}$ | 0.0185322 | 0.04112 | 70179.5 |
| 1020 | DisplMean~ $\psi_{cv}$+$\psi_{ce}$:$\psi_{ev}$ | 0.0235014 | 0.01113 | 71118.9 |
| 1021 | DisplMean~ $\psi_{ce}$:$\psi_{ev}$ | 0.0212925 | 0.00892 | 71185.8 |
| 1022 | DisplMean~ $\psi_{cv}$ | 0.0134067 | 0.00255 | 71380.8 |
