# Supplementary material for: Influence of interaction of cerebral fluids on ventricular deformation: A mathematical approach
Source: PLoS One. 2022 Feb 28;17(2):e0264395. doi: 10.1371/journal.pone.0264395 (PMC8884699; doi:10.1371/journal.pone.0264395)

The Residuals vs. Fitted values plot and Density vs. Residuals plot for Volunteer 1 (A), Volunteer 2 (B), Volunteer 3 (C) and Volunteer 4 (D).

Results for volunteer 1 and volunteer 2

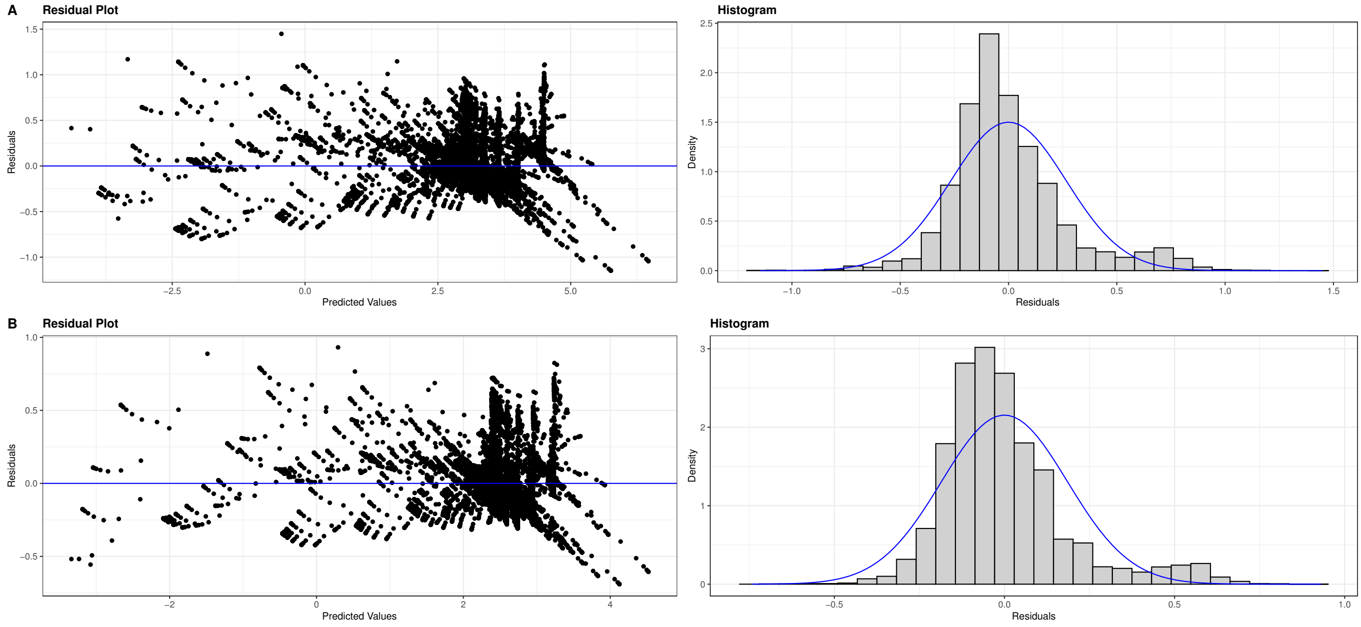

Results for volunteer 3 and volunteer 4

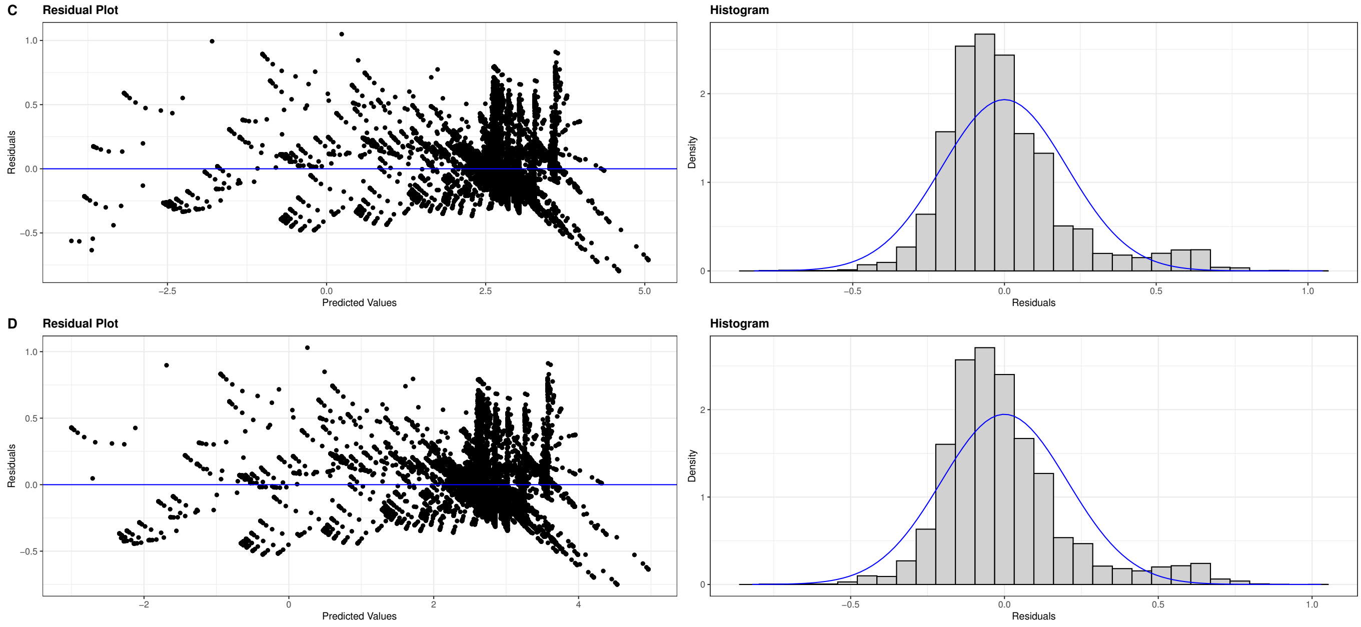

Supplement: S5 File — The Residuals vs. Fitted values plot and Density vs. Residuals plot for all volunteers. (PDF) [file pone.0264395.s006.pdf]
