# Supplementary material for: Influence of interaction of cerebral fluids on ventricular deformation: A mathematical approach
Source: PLoS One. 2022 Feb 28;17(2):e0264395. doi: 10.1371/journal.pone.0264395 (PMC8884699; doi:10.1371/journal.pone.0264395)
Supplement: S6 File — The file provides the numerical results: dependence of capillary pressure (pc) and mean wall ventricular displacement (u¯) on the logarithm of the interaction parameters (gxy) for all four volunteers. (PDF) [file pone.0264395.s007.pdf]

The plots below show the numerical results: dependence of capillary pressure (p_c) on the logarithm of the interaction coefficients (g_{xy}) for all four volunteers.

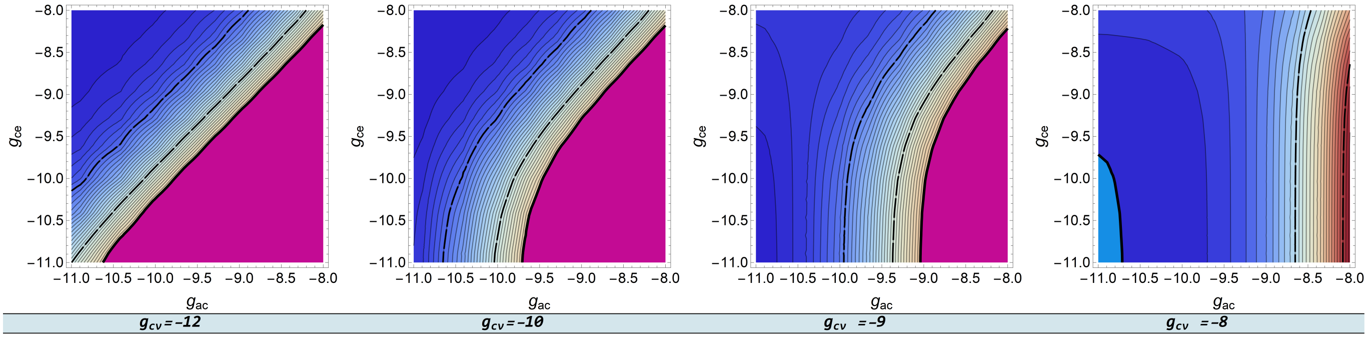

Capillary pressure results for volunteer 1

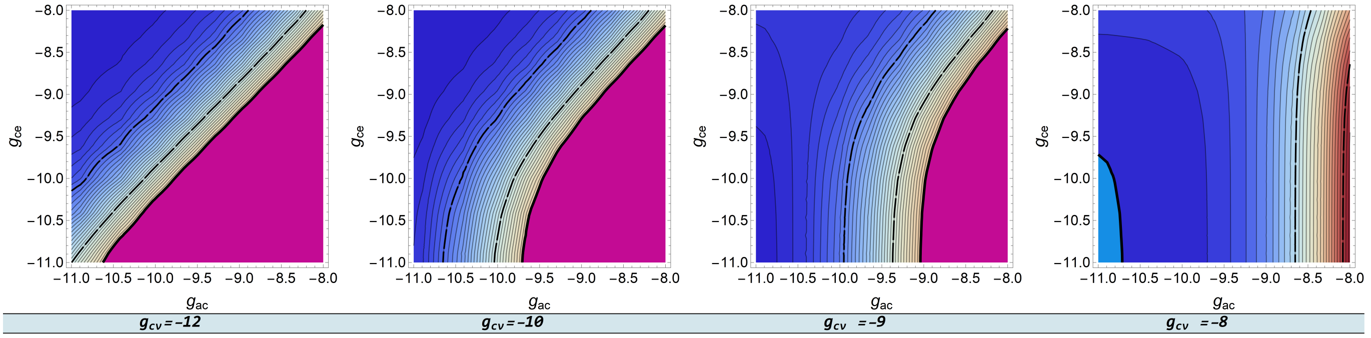

Capillary pressure results for volunteer 2

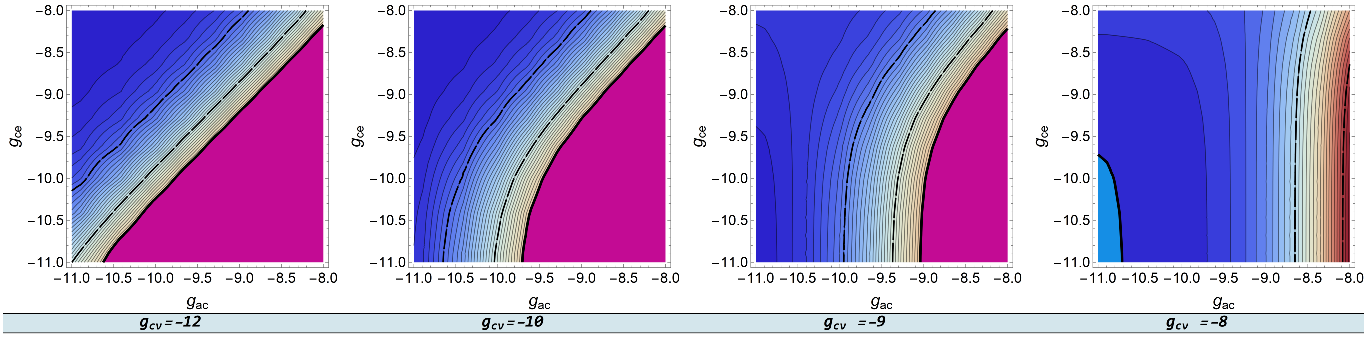

Capillary pressure results for volunteer 3

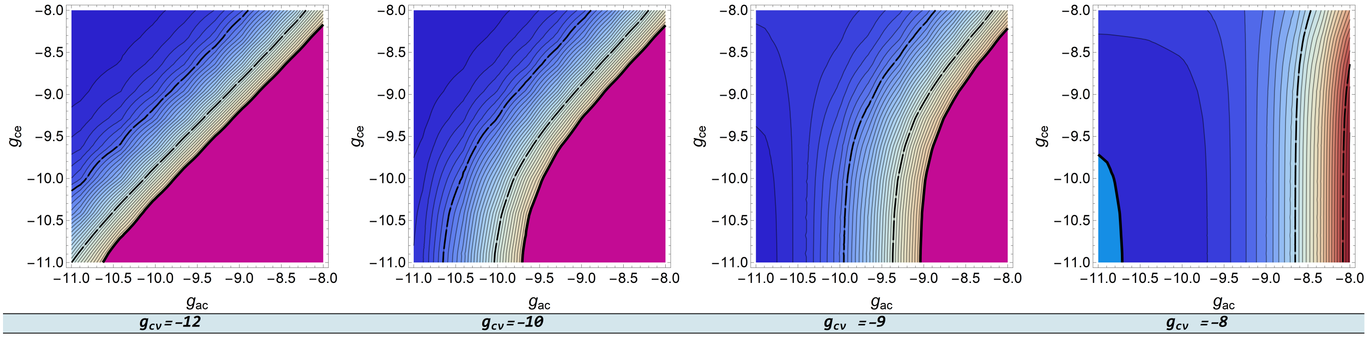

Capillary pressure results for volunteer 4

The plots below show the numerical results: dependence of mean displacement ($\overline{\boldsymbol{u}}$) on the logarithm of the interaction coefficients (g_{xy}) for all four volunteers.

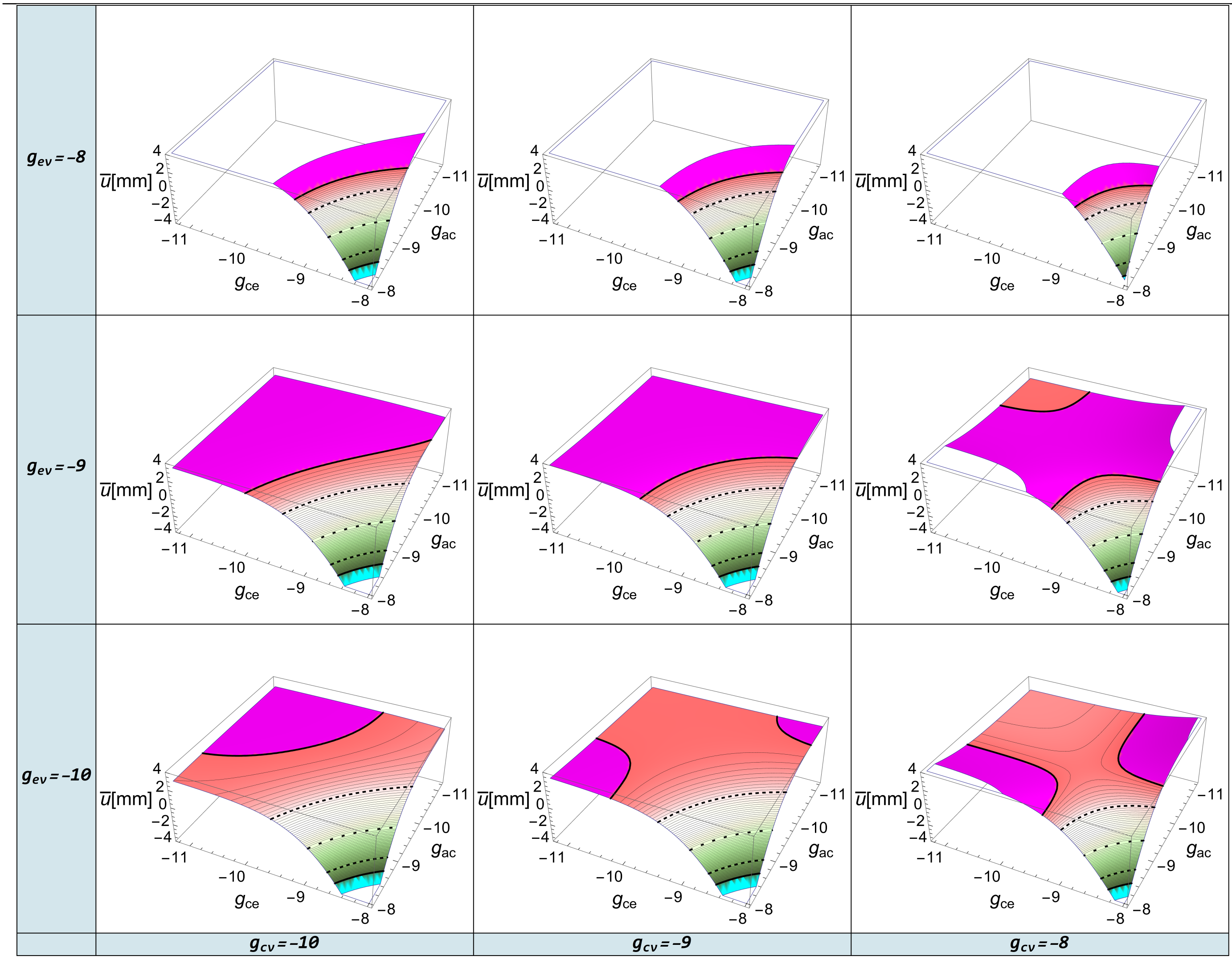

Mean displacement results for volunteer 1

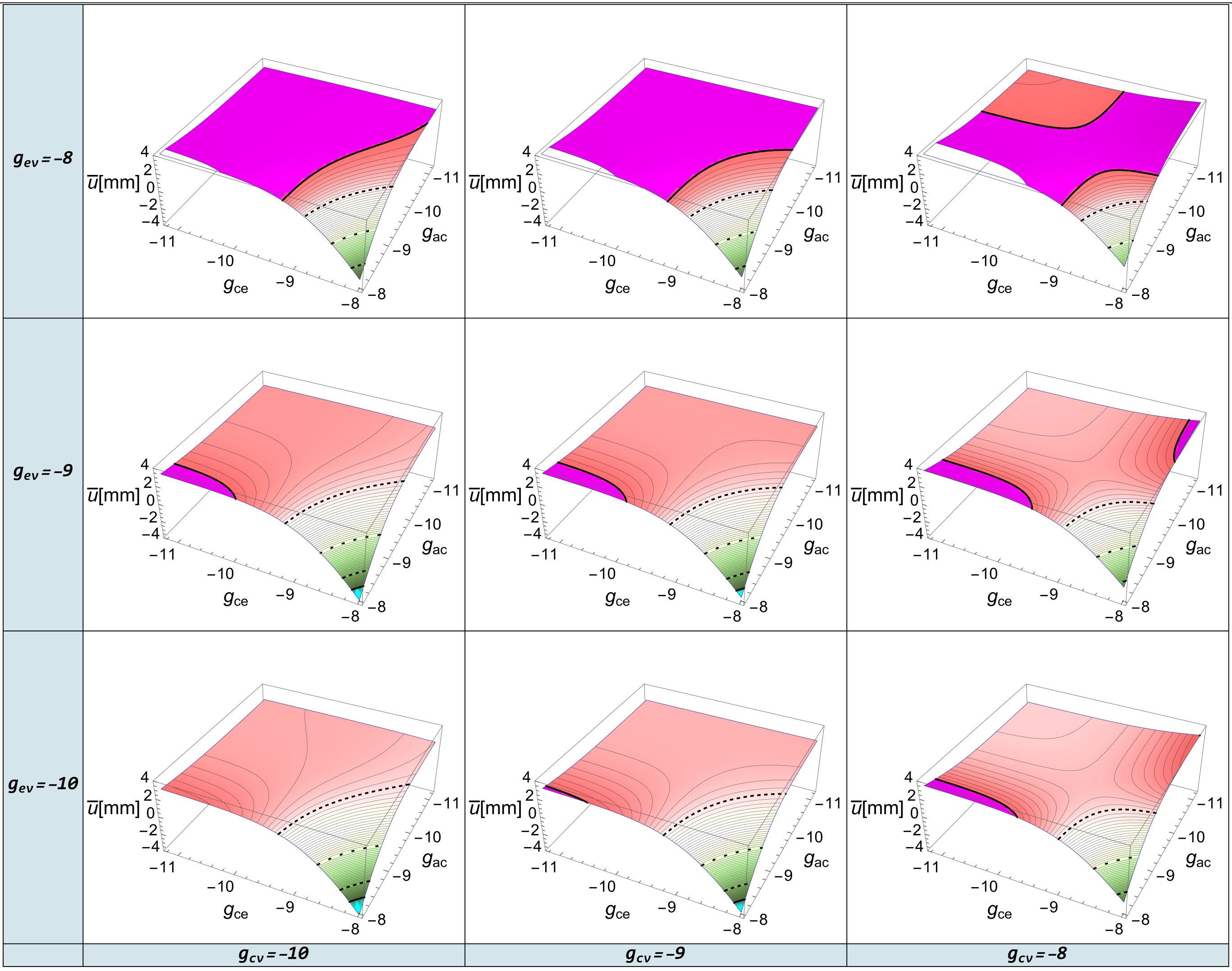

Mean displacement results for volunteer 2

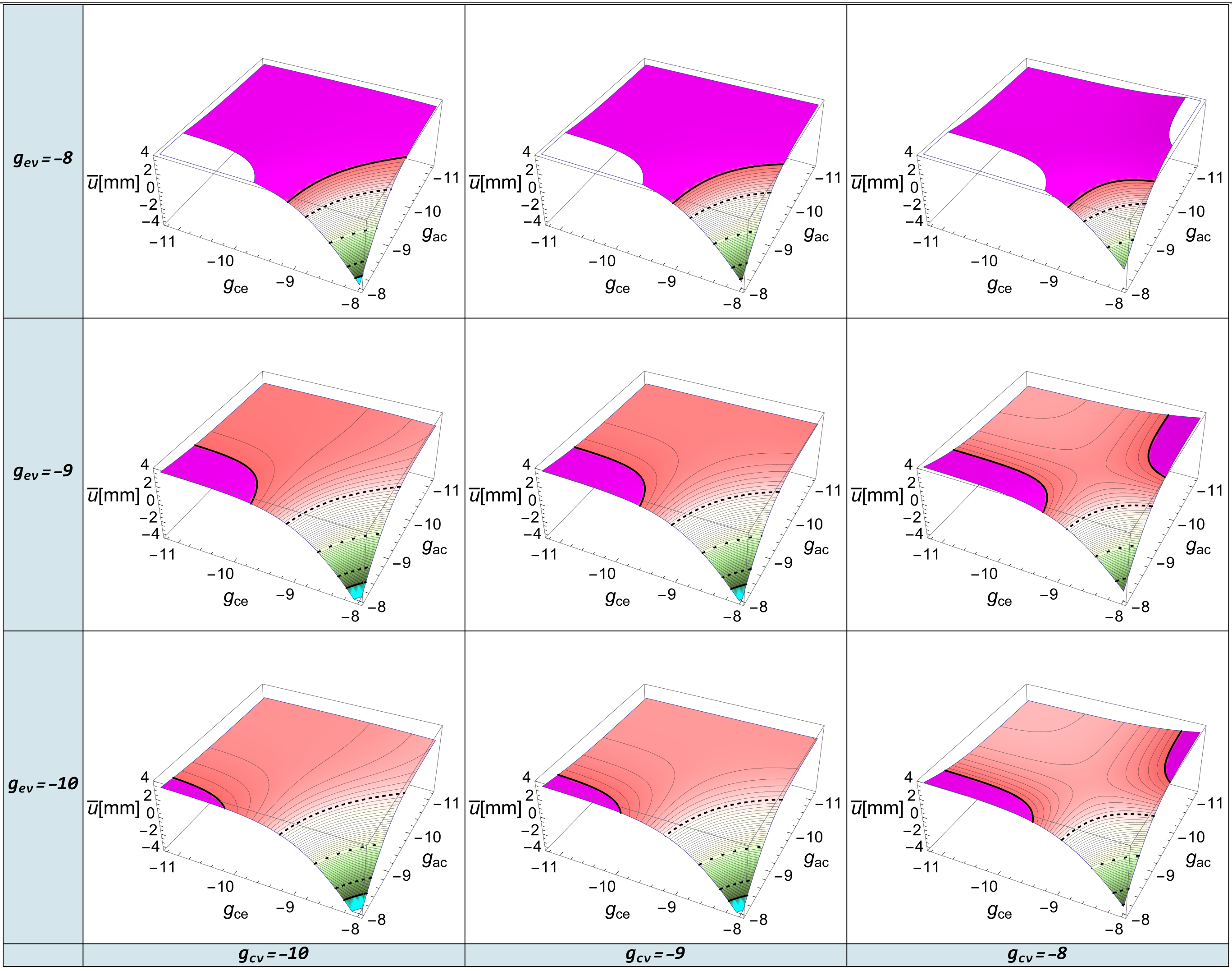

Mean displacement results for volunteer 3

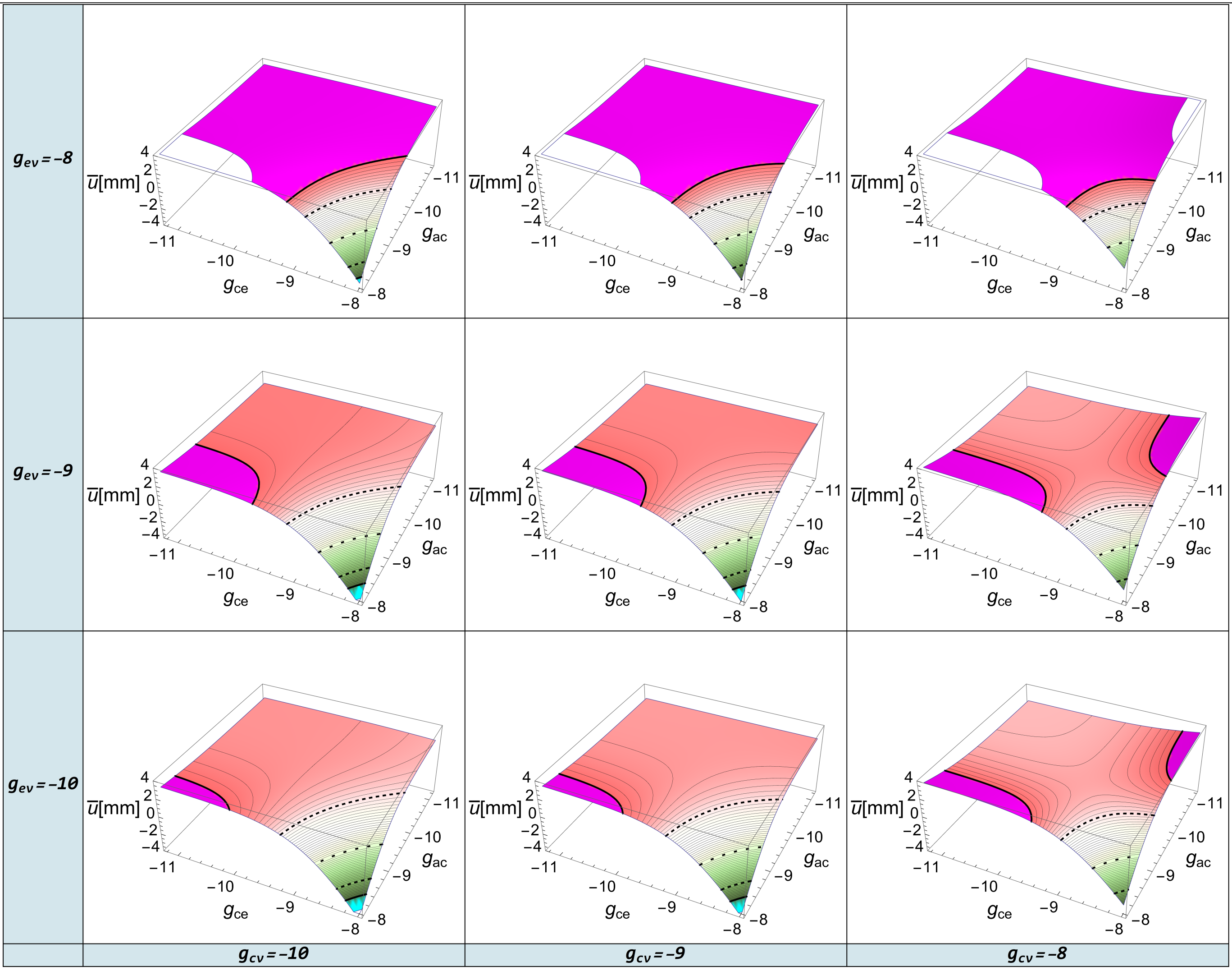

Mean displacement results for volunteer 4

—l—
